# Preferences for multi-cancer tests (MCTs) in primary care: discrete choice experiments of general practitioners and the general public in England

**DOI:** 10.1038/s41416-025-03063-9

**Published:** 2025-06-02

**Authors:** John Buckell, Nomalanga Madyiwa, Gail Hayward, Mark R. Middleton, FD Richard Hobbs, James Buchanan, Apostolos Tsiachristas, Brian D. Nicholson

**Affiliations:** 1 Health Economics Research Centre, Oxford Population Health, University of Oxford, Oxford, England; 2 Buckinghamshire Healthcare NHS Trust, Ayelsbury, England; 3 Nuffield Department of Primary Health Care Sciences, University of Oxford, Oxford, England; 4 Department of Oncology, University of Oxford, Oxford, England; 5 Health Economics and Policy Research Unit, Centre for Evaluation and Methods, Wolfson Institute of Population Health, Queen Mary University, London, England

**Keywords:** Cancer, Economics

## Abstract

**Background:**

Multi-Cancer tests (MCTs) hold potential to detect cancer across multiple sites and some predict the origin of the cancer signal. Understanding stakeholder preferences for MCTs could help to develop appealing MCTs, encouraging their adoption.

**Methods:**

Discrete Choice Experiments (DCEs) conducted online in England.

**Results:**

GPs (*n* = 251) and the general public (*n* = 1005) preferred MCTs that maximised negative predictive value, positive predictive value, and could test for a larger number of cancer sites. A reduction of the NPV of 4.0% was balanced by a 12.5% increase in the PPV for people and a 32.5% increase in PPV for GPs. People from ethnic minority backgrounds placed less importance on whether MCTs can detect multiple cancers. People with more knowledge and experience of cancer placed substantial importance on the MCT being able to detect cancer at an early stage. Both GPs and members of the public preferred the MCT reported in the SYMPLIFY study to FIT, PSA, and CA125, and preferred the SYMPLIFY MCT to 91% (GPs) and 95% (people) of 2048 simulated MCTs.

**Conclusions:**

These findings provide a basis for designing clinical implementation strategies for MCTs, according to their performance characteristics.

## Introduction

Cancers have moved from fifth to third place of global causes of morbidity and mortality across all ages [1]. Consequently, global effort is now focused on early diagnosis as early detection allows for timely intervention, widens the range of available curative treatments, and improves health outcomes [2–4]. In line with this international trend, cancer screening and early diagnosis is a high priority in England’s National Health Service (NHS). This is driven not just by the high cancer prevalence and mortality, but also the associated health inequalities [5–7]. Three national screening programs (breast, cervical, and bowel) and targeted lung health checks for people at high-risk of lung cancer have increased early-stage diagnosis in recent years [8]. However, approximately 90% of cancers are detected outside the screening programs, with most diagnosed following symptomatic presentation to NHS primary care [9].

In 2021 the NHS implemented the Faster Diagnosis Standard where patients with suspected cancer should have a diagnosis or cancer ruled out in 28 days [10]. General Practitioners (GPs) may refer patients with symptoms and signs meeting the National Institute for Health and Care Excellence (NICE) Suspected Cancer guideline criteria for urgent cancer investigation. GPs use a combination of clinical judgement and tests to decide which symptomatic patients to refer. The current standard-of-care for patient triage include some cancer-specific tests [11–13] (e.g. faecal immunochemical testing (FIT) for colorectal cancer; CA-125 for ovarian cancer; PSA for prostate cancer; serum protein electrophoresis for myeloma). Other non-specific tests (e.g. haemoglobin, platelets, and inflammatory markers) may indirectly indicate cancer but also indicate non-cancer conditions [14]. Improved triage testing could therefore expedite referral for symptomatic patients at high risk of cancer whilst avoiding unnecessary and potentially invasive diagnostic procedures for patients with benign or no disease [15].

Multi-Cancer tests (MCTs) hold great potential to detect cancer across multiple sites and some also predict the origin of the cancer signal [16, 17]. They are a broad class of technologies, commonly analysing sequenced circulating tumour DNA but also a broad range of biomolecules such as circulating tumour cells, extra-cellular vesicles, proteins, and metabolites [18]. The optimal use for MCTs is yet to be determined. With multiple MCT approaches, indicative performance varies by cancer type and cancer stage. There are also many possible use cases (e.g. screening asymptomatic people, triage in symptomatic patients, detection of residual disease or recurrence following cancer treatment), and very few large-scale prospective studies have been performed to understand efficacy and effectiveness in clinical practice [19]. In the UK, the NHS-Galleri trial is an on-going prospective randomised controlled trial (RCT) investigating if screening with an MCT reduces the incidence of late-stage cancer when used in an asymptomatic population [20]. The observational prospective SYMPLIFY study investigated the efficacy in patients referred with signs and symptoms of cancer for urgent cancer investigation in England and Wales [21]. 5461 participants enrolled in the trial across 44 hospitals in England. The study used Galleri, a MCT (blood test), which tested for over 50 types of cancer. This MCT had good positive (76%) and negative (98%) predictive values, returned results in 1–2 weeks, and was able to identify the cancer site. Modelling of SYMPLIFY data showed the potential for the MCT to be used as a triage test in primary care.

Implementation of MCTs will necessarily take into consideration the perspectives of key stakeholder groups: members of the general public, general practitioners (GPs), device manufacturers and health care payers [22] (NHS commissioners and private insurance companies). Understanding preferences early on could steer innovation and uptake, as illustrated by the development of Target Product Profiles [23]. Indeed, a recent study [24] noted, “...a major need for more rigorous data regarding MCDs [multicancer detection tests] to inform the development of guidelines for use as cancer screening tools.”

This study used a discrete choice experiment (DCE) to evaluate preferences of GPs and members of the public for MCT characteristics when used to investigate symptomatic patients in primary care, to ascertain the trade-offs that they are willing to make to arrive at a testing decision. We measure the value of test accuracy, and the relative value of NPV and PPV. We used hypothetical MCTs and compared them to tests currently used in practice and reported in the literature.

## Methods

Ethical approval was granted from OxTREC ethics committee at Oxford, REF: R90824/RE001. Main analyses were pre-registered prior to collection of the data [25]; full details in Supplement 4.

### Sampling

To elicit preferences of GPs, 251 GPs were recruited by M3 Group, who recruit medical professionals for surveys. They used email lists that GPs had signed up to. We included GPs that were currently practising in England. Quotas (age, gender, and region), based on the NHS General Practice Workforce data, were used to increase representativeness. 6757 GPs were invited to participate in the survey. 812 started the survey, 66 failed eligibility, 104 exceeded the maximum time limit, and 391 were in excess of the quotas (and did not complete the survey); see Supplement 3 for further details. GPs were rewarded with credits on M3’s platform equivalent to a GP’s hourly rate pro-rated to the expected survey time of 15 min.

With regards to public preferences, 1005 members of the general public were recruited by SurveyEngine, a survey company that provides panels in the UK. They used email lists that individuals signed up to. We included adults (18 years or older) living in England. Quotas (age, gender, and region) based on the UK census, were used to promote representativeness. 1465 individuals were invited to participate in the survey. 1413 started the survey, 70 failed eligibility, 158 exceeded the maximum time limit, 7 failed the minimum time limit (less that 1/3 of the median time taken in the pilot study), 4 failed open-ended sense response checks (i.e. gave erratic responses), and 169 were in excess of the quotas (and did not complete the survey); see Supplement 3 for further details. Individuals were rewarded with credits on SurveyEngine’s platform equivalent to the UK minimum wage pro-rated to the expected survey time of 15 min.

### Discrete choice experiment (DCE)

A DCE is a technique used widely in health to understand preferences by asking participants to make specific choices [26, 27]. Here, participants were presented with a choice between two MCTs. Each test was defined by a set of attributes, such as whether the test can identify the cancer site, and the variation in each attribute (in this example, “yes” or “no”) is referred to as a level. By making a series of choices between a hypothetical MCT with one set of attributes and levels and another MCT with alternative attributes and levels, participants implicitly reveal the degree to which each attribute is important to them and the value they place on each level of the attribute.

The two DCEs, one for each stakeholder group, were designed according to experimental best practices, ranging from design efficiency to participant-centred aspects such as checking experimental tasks were clear to participants [28, 29]. Individuals made 12 choices between two hypothetical MCTs. The alternatives were described by attributes and levels (below) representing different hypothetical MCTs.

#### Attributes and levels

Seven attributes described the characteristics of MCTs in the choice tasks, summarised in Table 1. The full descriptions, as presented to respondents, are provided in supplement 1. Attributes and levels were selected based on several sources of evidence: a scoping review of choice experiments including diagnostic technologies (see Supplement 12 for details); collaborative working with clinical experts on the study team; and a group interview with members of the public (*n* = 4).

**Table 1:** Descriptive system: attributes and levels used in the discrete choice experiment.

	GPs	General public
Risk of cancer after a positive test (GPs); Test gets it wrong when it tells us there is a cancer (General public)	20%	Gets it wrong 8 out of 10 times
	40%	Gets it wrong 6 out of 10 times
	60%	Gets it wrong 4 out of 10 times
	80%	Gets it wrong 2 out of 10 times
Risk of cancer after a negative test (GPs); Test gets it wrong when it tells us there is not a cancer (General public)	0.1%	Almost never (1 out of 1000 times)
	0.5%	Incredibly rarely (5 out of 1000 times)
	1%	Very, very rarely (10 out of 1000 times)
	4%	Rarely (40 out of 1000 times)
Waiting time for test results	1 week	1 week
	1 to 2 weeks	1 to 2 weeks
Number of cancer sites tested (GPs); Number of cancers tested for (General public)	1	1
	5	5
	10	10
	25	25
Can the test identify the site? (GPs); Test detects type of cancer? (General public)	Yes	Yes
	No	No
Form of the test	Blood test	Blood test
	Faecal test	Faecal test
	Urine test	Urine test
	Breath test	Breath test
Can the test detect the stage of cancer? (GPs); Test can detect cancer at an early stage? (General public)	Yes	Yes
	No	No

#### PPIE input into the design

A focus group with 4 members of the public helped to maximise understanding of the experiment by discussing drafts and refining the descriptions of the attributes. For example, the attribute of number of cancer sites tested for was reworded to “number of cancers tested for” for members of the general public as this was clearer to focus group participants (though not for GPs since this terminology is routine to them). This group agreed that communicating test accuracy in three ways (rhetorically, numerically, and graphically), rather than just numerically, would maximise respondents’ understanding. In addition, PPIE contributors found the concepts of sensitivity and specificity difficult to understand despite various options being presented to them. They found explanations of predictive values easier to comprehend when expressed as the accuracy of a positive or negative test. We therefore opted to include attributes about positive and negative test accuracy in both patient and GP DCEs to allow comparison between the two groups. All information was framed to increase understanding, drawing on this qualitative work and public input.

#### Pilot phase with members of the public

We conducted a pilot study in members of the public (*n* = 62), in which we asked respondents to report misunderstandings and/or difficulties, and adjusted questions as required. None of the respondents reported difficulties in understanding and none reported any discomfort in taking the survey. The attribute levels were altered from the analysis plan following the analyses of the pilot data. These alterations were intended to improve understanding and efficiency: (i) The form of the test attribute levels “saliva” and “oral swab” were removed. In the pilot data, there was no evidence to suggest that the levels “saliva” or “oral swab” were impactful on test preferences therefore these levels were removed from the final design. (ii) The negative test accuracy attribute levels for GPs were changed from 6 to 4 levels. The attribute for negative test accuracy for GPs had one level removed to simplify and balance the design so that each level for each attribute appears the same number of times. (iii) Randomisation of members of the public to positive or negative predictive value attributes. From the PPIE focus groups and pilot data, it became clear that expressing test accuracy both positively and negatively simultaneously was confusing. Keeping both of these attributes in a single DCE would likely result in poor quality data because comprehension would be low. We switched to a split design (Hess et al., 2017), where half of the public sample would be randomly allocated to a design with PPV, and the other half to NPV; all other attributes and levels were the same in both arms. Hence, each patient’s choices were described by 6 attributes, and preferences for the full set of 7 attributes were subsequently modelled by combining the arms and applying appropriate statistical corrections (see later section).

#### Experimental design

A Bayesian D-efficient design was generated that contained 24 choice tasks. Priors were obtained from the pilot study. For GPs, the tasks included 7 attributes. For members of the public, two versions of choice tasks (where respondents were randomised to either a positive or a negative predictive value) were generated from the same design. This design used two utility functions, one each for each choice task version, and averaged over those versions. In both samples, individuals were randomized to two blocks of 12 choice tasks, balancing concerns of learning and respondent fatigue [29]; that is, to keep the number of tasks manageable for respondents. Examples of choice tasks, and the schematic for randomization through the survey, are presented in Supplement 2.

### Survey

The online survey was distributed through email lists, accompanied by an introductory letter and instruction sheet. The questionnaire, tailored to each sample, collected sociodemographic information (both samples), experience and knowledge of cancer (general public sample), practice characteristics (GP sample), and validation questions at the end of the survey to assess the quality of their responses. Public cancer knowledge and experiences were assessed using a set of questions in the survey, including how many symptoms of cancer individuals could identify from a list, whether the individual had ever had cancer, whether the individual had ever been tested for cancer, and whether the individual had ever been screened for cancer. They were asked further questions on their health behaviours (smoking, exercise, etc.).

### Data quality

Respondents were given narrative and visual information describing the alternatives, attributes, and levels. A practice choice scenario was presented prior to the main experiment. “Forced responses” prevented respondents from skipping past questions in the survey; and a minimum time threshold of 2.5 min, based on pilot data, removed respondents who rushed through. Duplicate survey responses were rejected. Open-ended text responses were examined and suspicious responses (such as “October” given as an answer to a cancer estimate question) were identified and removed [30]. A post-experiment question asked respondents which attribute was most important. This allowed us to assess internal validity by checking for consistency with the model estimates.

#### Statistical analyses

Our sample size was sufficient to ensure statistical power based on the pilot parameter estimates [31].

Primary analyses used mixed multinomial logit models to analyse the impact of attribute levels on experimental choices. The dependent variable in the regression models was the selection of the hypothetical tests, “MCT 1”, or “MCT 2”. Attributes were independent variables. Attributes were dummy-coded [32]. Odds ratios were computed by exponentiating the estimated coefficients.

Data from the GP and public samples were pooled and modelled jointly. A first scale parameter adjusted for the public randomisation to either the positive or negative predictive value. A second scale parameter adjusted for differences between the GP and public experimental layout (e.g. wording differences of the same attributes between the two designs). Sample weighting was applied in the regressions to account for the unequal size of the two samples (the sample of public was four times larger than the sample of GPs). A set of interaction terms were specified using a “sample” dummy (i.e. GP or public sample) and the attributes.

Marginal rates of substitution (MRSs) were computed for NPV and PPV for both GPs and the general public, these being the ratios of estimated parameters (delta method applied to compute confidence intervals). MRSs reflect the relative value of PPV and NPV. More specifically, MRSs give the amount of PPV that would be needed to compensate for a lower NPV between two MCTs, such that they are considered as good as one another (all else being equal).

Secondary analyses assessed preference variation in the overall sample across a set of demographics using interaction terms: age, gender, ethnicity, education, and urbanicity. For deterministic heterogeneity, a refining procedure specified models with all interactions and iteratively removed non-significant parameters from each model. A factor analysis was performed to investigate patterns of correlation between public cancer knowledge and experiences, and the results of this analysis guided the construction of a latent variable of cancer knowledge and experience. In measurement equations of the latent variable, each question was treated as an outcome and the latent variable was the explanatory variable. Using a structural equation, the latent variable was regressed on a set of individual covariates (i.e. age, gender, ethnicity, education, and rurality) to understand how cancer experience varies across demographics. Finally, the latent variable of cancer knowledge and experience entered the choice model in a similar way to patient demographics, to relate attribute preference variation with patient knowledge and experience of cancer. This model extends the mixed multinomial logit using a system of equations that is simultaneously estimated, and known as Integrated Choice and Latent Variable (ICLV) model [33]. See supplement 4 for details and a diagram of the system of equations.

Statistical significance was examined with t-ratios (i.e. two-tailed t-tests). Models were estimated using the Apollo package in R [34]. Code scripts are available on request.

#### Simulations for ranking MCTs

It is possible to simulate choices for any combination of attributes by setting the attribute values in the data and applying these to the model. In this way, we predicted probabilities of choosing all possible MCTs based on all the combinations of attribute levels in the design (*n* = 2048). By taking any one MCT and using it as a comparator (i.e., benchmark), we can rank the entire set of MCTs relative to that comparator. In this case, we used the characteristics of GRAIL’s Galleri MCT as reported in the SYMPLIFY study for comparison as, at the time of writing, it was only MCT with published data from a symptomatic population (hereafter named the ‘SYMPLIFY MCT’) [21]. In addition, we compared preferences for simulated MCTs with the features of cancer diagnostics currently used in primary care practice: the faecal immunochemical test (FIT); prostate-specific antigen (PSA) test; and the CA125 blood test. Technical details, and details of the MCTs, are provided in Supplements 4 and 5.

## Results

A summary of the characteristics of the public and GP samples is presented in Table 2.

**Table 2:** Respondent characteristics.

	Public	General practitioners
*n*	1005	251
Age (median [IQR])	44 [32.0, 63.0]	43 [39.0, 49.5]
Gender (%)		
Male	493 (49.1)	106 (42.2)
Female	509 (50.6)	145 (57.8)
Non-binary	2 (0.2)	–
Prefer not to say	1 (0.1)	–
Ethnicity (%)		
Afro-Caribbean	95 (9.5)	8 (3.2)
Asian	83 (8.3)	76 (30.3)
Mixed race	19 (1.9)	10 (4.0)
White	805 (80.1)	151 (60.2)
Other	3 (0.3)	6 (2.4)
Qualifications		
Higher education: degree or higher	433 (43.1)	–
Secondary education: a levels	337 (33.5)	–
Vocational/work-related	179 (17.8)	–
None of the above	56 (5.6)	–
Region (%)		
East of England	109 (10.8)	27 (10.8)
London	163 (16.2)	39 (15.5)
Midlands	198 (19.7)	48 (19.1)
North East	48 (4.8)	–
North East and Yorkshire	–	39 (15.5)
North West	125 (12.4)	32 (12.7)
South East	159 (15.8)	38 (15.1)
South West	108 (10.7)	28 (11.2)
Yorkshire and Humber	97 (9.7)	–
Midlands	–	48 (19.1)
Household size (median [IQR])	2 [2.0, 4.0)	–
Household income (median [IQR])	35 000 [25 000, 55 000]	–
Employment (%)		
Yes	629 (62.6)	–
No	369 (36.7)	–
Prefer not to answer	7 (0.7)	–
Number of GPs in practice (%)		
<5	–	46 (18.3)
5–10	–	144 (57.4)
11–15	–	36 (14.3)
16–20	–	13 (5.2)
>20	–	12 (4.8)
Years in practice (%)		
Less than 1 year	–	3 (1.2)
1–2 years	–	8 (3.2)
3–5 years	–	25 (10.0)
6–10 years	–	45 (17.9)
More than 10 years	–	170 (67.7)
Number of individuals (%)		
<1000	–	3 (1.2)
1000–5000	–	14 (5.6)
5000–10,000	–	76 (30.3)
10,000–15,000	–	93 (37.1)
15,000–20,000	–	32 (12.7)
>20,000	–	33 (13.1)
Area (%)		
Rural	228 (22.7)	41 (16.3)
Suburban	459 (45.7)	134 (53.4)
Urban	318 (31.6)	76 (30.3)
Previous cancer test arranged by GP (%)		
Yes	334 (33.2)	–
No	671 (66.8)	–
Ever had cancer (%)		–
Yes	9 (10.0)	–
No	904 (90.0)	–
Frequency of MCT use (%)		
Never	–	11 (4.4)
Rarely	–	19 (7.6)
Occasionally	–	77 (30.7)
Regularly	–	101 (40.2)
Frequently	–	43 (17.1)
Participation in cancer screening (%)		
Yes	350 (34.8)	–
No	577 (57.7)	–
Ineligible due to age	78 (77.6)	–

### Members of the public

The sample characteristics of the 1005 members of the public included in the analysis were reflective of the latest population estimates for age, gender and ethnicity. The median household income was at the current UK median income of £34,500 [35]. The median age was 44 years, and 50.6% (*n* = 509) of participants were female. About 4 in 10 participants were educated to degree level or higher and the majority were employed (62.6%, *n* = 629). 33.2% (*n* = 334) of participants reported that they have had cancer screening tests arranged by their GP and 34.8% (*n* = 350) reported participation in cancer screening programmes.

### General practitioners

251 GP responses were included in the analysis. Most of the GPs were UK trained (96%, *n* = 241) with about two thirds of them having more than 10 years of experience (*n* = 170). GP practices surveyed were distributed across England with just over half of them situated in suburban areas. 40.2% (*n* = 101) of GPs reported that they would use an MCT regularly while 4.4% (*n* = 11) indicated that they would never use an MCT.

#### MCT preferences for the general public and GPs

**Fig. 1:**
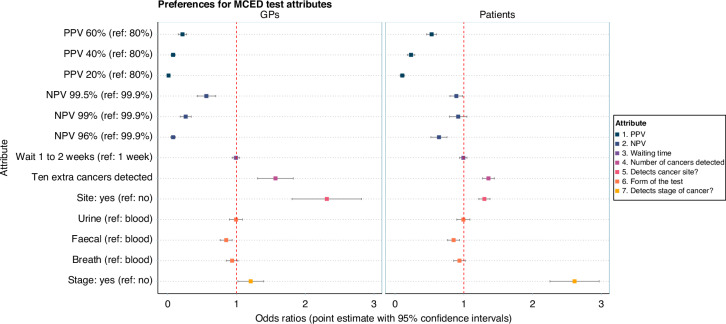
Odds ratios for MCT attributes. Point estimates and 95% Krinsky–Robb confidence intervals are shown. These show the probability of choosing an MCT for the attribute level, relative to the reference (omitted) category, all else being equal. For continuous attributes, preferences are estimated at the margin (i.e. denote the effect on MCT choice of a one-unit change in that attribute). Accordingly, we express preferences in multiple units: Number of Cancers Tested for (10 extra). PPV and NPV are scientific measures for which positive and negative test accuracies were used as proxies in the survey.

Figure 1 presents the estimated odds ratios (ORs) for MCT attributes (Supplement 8 for mixed multinomial logit estimates and diagnostics; Supplement 4 for testing and robustness analyses). These reflect the sample level effect of each attribute level on the probability of choosing an MCT.

#### GP preferences for MCTs

GPs were less likely to choose MCTs with lower PPV and NPV. The OR of choosing an MCT with PPV of 60% (versus a PPV of 80%, the reference) was 0.21 (95% CI: 0.15–0.28). The OR of choosing an MCT with NPV of 99.5% (versus a NPV of 99.9%, the reference) was 0.56 (95% CI: 0.43–0.70). ORs continued to decline towards 0 as PPV and NPV decreased to the bottom of their respective ranges, namely 20% PPV and 96% NPV. GPs were more likely to choose MCTs that detected more cancer sites (OR for an MCT that can detect 10 extra cancers 1.57; 95% CI: 1.31–1.83), can detect the cancer site versus not detecting the site (OR 2.31; 95% CI: 1.81–2.82), and can detect cancer at an early stage versus not detecting early stage (OR 1.21; 95% CI: 1.02–1.39). GPs’ MCT preferences were unrelated to waiting time and the form of the test, with the exception of a slight negative preference for a faecal (vs. blood) test (OR 0.85; 95% CI: 0.76–0.94).

#### Public preferences for MCTs

General public respondents were less likely to choose MCTs with lower PPV. The OR of choosing an MCT with PPV of 60% (versus a PPV of 80%, the reference) was 0.53 (95% CI: 0.46–0.60). ORs continued to decline towards 0 as PPV decreased to the bottom of its range, 20% PPV. Lower NPV reduced the chances of MCT choices only at the lower end of its range, OR for NPV of 96% (versus 99.9%) was 0.64 (95% CI: 0.52–0.76). General public respondents were more likely to choose MCTs that detected more cancer sites (OR for an MCT that can detect 10 extra cancers 1.36; 95% CI: 1.27–1.44), can detect the cancer site (OR 1.30; 95% CI: 1.22–1.38), and can detect cancer at an early stage (OR 2.61; 95% CI: 2.25–2.97). Public MCT preferences were unrelated to waiting time and the form of the test, with the exception of a slight negative preference for a faecal (vs. blood) test (OR 0.85; 95% CI: 0.76–0.94).

#### Marginal rates of substitution (MRSs) between NPV and PPV

**Fig. 2:**
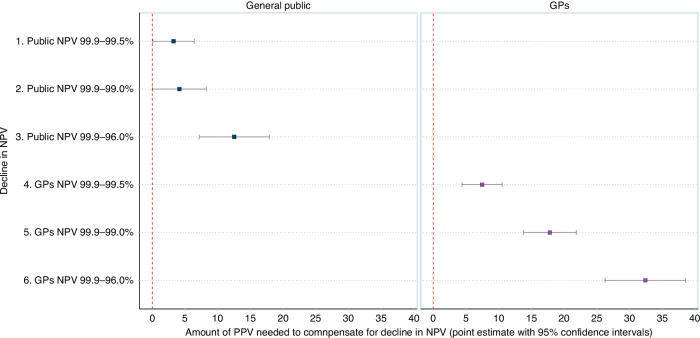
Marginal rates of substitution (MRSs) between NPV and PPV. Reduction in NPV is listed on the y-axis. The x-axis shows the gain in PPV needed to compensate for the decline in NPV such that the MCT is as good. The left pane shows the general public and the right pane shows GPs. Row 3 shows that for the general public, two MCTs with NPVs of 99.9% and 96.0% would be considered equal if the latter had a higher PPV by 12.5% (95% CI: 7.2–17.9%). Row 6 shows that for the GPs, two MCTs with NPVs of 99.9% and 96.0% would be considered equal if the latter had a higher PPV by 32.4% (26.3–38.6%).

Figure 2 shows the MRSs for GPs and for the general public. Row 3 shows that for the general public, two MCTs with NPVs of 99.9% and 96.0% would be as good as each other if the latter had a higher PPV of 12.5% (95% CI: 7.2–17.9%). Row 6 shows that for the GPs, two MCTs with NPVs of 99.9% and 96.0% would be as good as each other if the latter had an increased PPV of 32.4% (95% CI: 26.3–38.6%). The amount of PPV increase did not depend on the starting point of PPV (Supplement 12).

#### Comparison of MCTs with other cancer diagnostics

**Fig. 3:**
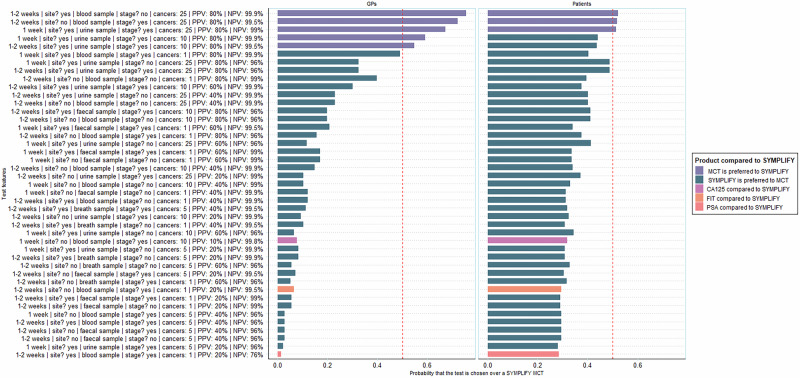
Comparison of MCTs to SYMPLIFY. Shown is a subset of 40 hypothetical MCTs from the experiment randomly drawn from the full set of 2048 and the three tests in clinical practice: CA-125, FIT, and PSA. Each row shows an individual simulated MCT with its features listed on the y-axis. For each, the predicted probability of choosing that MCT over the SYMPLIFY MCT is presented. Purple bars are MCTs that are preferred to the SYMPLIFY MCT; blue bars denote that the SYMPLIFY MCT is preferred; pink and orange bars compare tests used in clinical practice to the SYMPLIFY MCT. The top row, for example, shows an MCT that: takes 1–2 weeks to return its results, does identify the cancer site, requires a faecal sample, does detect early stage cancer, tests for 25 different cancers, has a PPV of 80%, and a NPV of 99.9%. For GPs, there is around a 77% probability that this test would be chosen over the SYMPLIFY MCT; for patients, there is around a 52% chance that this test would be chosen over the SYMPLIFY MCT.

All 2048 simulated choice probabilities are presented in Supplement 12. Compared to 2048 simulated MCTs, the SYMPLIFY MCT was preferred in 91% (GPs) and 95% (people) of cases. MCTs with higher probabilities had higher values for the more valued attributes (high NPV, high PPV, and the detection of more cancer sites). Both GPs and members of the public preferred the SYMPLIFY MCT to FIT, PSA, and CA125. Figure 3 presents a random subset of 40 simulations (one per row) compared to the SYMPLIFY MCT, and including PSA, FIT, and CA125.

### Preference variation by patient characteristics

**Fig. 4:**
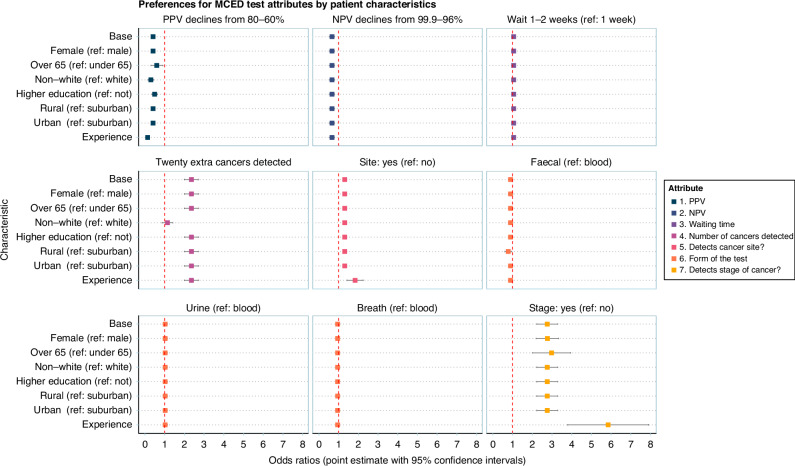
MCT attribute preferences by patient characteristics. “Base” is the base coefficient, meaning the reference categories for each characteristic. Each characteristic was tested in model refinement and non-significant interaction terms were removed for parsimony. All enter as binary variables (except for cancer knowledge and experience, “Experience”), with the reference category shown in parentheses. “Experience” here is a combination of: the individual has undergone cancer screening (versus has not), the individual has previously had cancer (versus has not), and the individual can identify 8 out of 16 cancer symptoms.

Figure 4 shows preferences for MCT attribute levels by public characteristics. The full Integrated Choice and Latent Variable (ICLV) model results are presented in Supplement 9. Results are based on significant interactions in the model (Fig. 4 shows the overall effect for each demographic).

Those over 65 yeas old (OR: 0.60, 95% CI: 0.30–0.89) were more likely to select an MCT if PPV declined from 80% to 60%, compared to those under 65 years old (0.41, 0.32–0.50); i.e. they were less put off by less accurate MCTs. Those from ethnic minority backgrounds (0.30, 0.15–0.45) were more sensitive to PPV than respondents of white ethnicity (0.41, 0.32–0.50). These respondents’ choices, unlike for white respondents, were not impacted by the MCT being able to detect more cancers (1.14, 0.87–1.41). Respondents in rural settings (0.78, 0.61–0.96) disliked faecal tests to a greater extent than those in suburban settings (0.89, 0.79–1.01). In four (of nine) cases (NPV, waiting time, urine test (vs. blood test), and breath test (versus blood test)) there was no evidence of preference variation across demographics.

Cancer knowledge and experience was related to MCT preferences. Individuals with a higher score on the latent variable for cancer knowledge and experience were able to identify more potential symptoms of cancer, were more likely to have been screened for cancer, and were more likely to have previously had cancer. These individuals were more likely to be female, over 65 years old, have higher education, and less likely to be from an ethnic minority background. These individuals were much less likely to select an MCT if PPV declined from 80% to 60% (OR: 0.13, 95% CI: 0.06–0.20), compared to those without cancer experience (0.41, 0.32–0.50). They preferred MCTs that identify the cancer site (1.83, 1.42–2.24 vs. 1.31, 1.20–1.41), and placed considerable importance on the MCT being able to detect cancer at an early stage (5.85, 3.80–7.90 vs. 2.76, 2.23–3.29).

## Discussion

GPs and the public preferred MCTs that maximised NPV, PPV, and could test for a larger number of cancers. Isolating the cancer site and detecting cancers at an early stage were important, but to a lesser degree. Both groups were indifferent between blood, urine, and breath tests; and preferred these three to faecal tests. Neither group’s preferences were influenced by waiting time. GPs placed substantial importance on NPV, requiring an almost three times greater increase in PPV to compensate for a reduction in MCT NPV compared to the general public: GPs, above all else, do not want to renege on a negative diagnosis. People placed substantial weight on the MCT being able detect early stage cancers. Respondents from ethnic minority backgrounds placed less importance on whether MCTs can detect multiple cancers. Respondents with more knowledge and experience of cancer placed more importance on PPV and on the MCT being able to identify the cancer site, and placed substantial importance on the MCT being able to detect cancer at an early stage. These individuals were more likely to be female, older, having higher education, and of white ethnicity.

### Strengths and limitations

Strengths of this study include the use of two key stakeholder groups. This facilitates comparisons between populations due to use of the same study design. We used large samples that applied quotas to increase representativeness. Our approach of randomising the general public to PPV and NPV arms avoided issues of confusing them by giving them both and, arguably, increased the reliability of the estimated preferences. Our sensitivity analyses confirmed that other preferences were consistent across these arms. Our data collection and modelling allowed us to investigate heterogeneity within samples based on our analysis plan. The use of the latent variable allowed us to ask whether observed differences in patient behaviour were linked to demographics or to cancer knowledge and experience (which itself was linked to patient demographics); that is, gives insight into the mechanisms through which demographics were related to MCT preferences. The latter was the dominant effect, underscoring the importance of doing so. A further strength of the study design is that it allowed us to rank preferences for a large number of potential MCTs (in excess of 2000). A further advantage is being able to use the model to simulate preferences for existing tests and compare these against the MCTs covered in the design.

Our study is subject to a set of limitations. Discrete choice experiments are subject to hypothetical bias [36]. However, given that very few MCTs are commonplace in clinical settings, any preference elicitation at this stage must be hypothetical. We took steps to mitigate any effects by relying heavily on patient input during the design – particularly in the wording and form of the experiment, the PPV-NPV randomisation being an example. We also instructed respondents that their responses would inform a scientific study, which has been shown to help to mitigate this bias [37]. This notwithstanding, the DCE allows for preferences to be measured. The size and direction of coefficients estimated was consistent with a priori expectations. In the open-ended questions section some respondents highlighted that accuracy was very important to them and therefore they consistently selected the most accurate tests; and some highlighted the challenging decision between prioritizing accuracy over early detection. These findings provide evidence on face validity. A further limitation is that we did not estimate preferences for sensitivity and specificity; only PPV and NPV. This was to maximise public understanding (they were better able to grasp PPV and NPV as concepts compared to sensitivity and specificity), and for GPs, it was important to align the study design with that of the general public. Another limitation of our design was that we used the number of cancer sites detected, but did not specify which these were. This could have been interpreted differently by respondents, and we are unable to observe this. A further limitation was in piloting members of the public only and not GPs. Our sample size of GPs is smaller than average for health-based choice experiments [31]. However, given the much smaller population size of GPs than members of the general public, in relative terms the sample is much larger. We used less granular quotas for GPs than for the general public to avoid quota bins with <5 individuals. We are at risk of other forms of bias. Given the response rates, our results are at risk of non-response. There is potential for selection bias if those who chose to take the online surveys have preferences that are systematically different to those in the wider populations. However, we use large samples with quotas to mitigate this risk, as per previous such studies [38].

### Comparison with the existing literature

A few studies have explored the perceptions of members of the general public regarding MCT use for screening of cancer [38–40]. In the UK, one study [39] carried out a quantitative, cross-sectional study of MCTs that evaluated intention to test as a surrogate for future uptake. Whilst important, this study has several limitations. First, the authors only ask about MCT uptake, without decomposing preferences for MCT characteristics (which may vary between both tests and across individuals). Second, the authors only ask about blood-based MCTs, though other forms of test are available. Third, the study focuses on just a patient population. However, given the dyadic nature of clinical encounters, clinician preferences are also likely to be central to decision-making about MCT use (as are commissioners, though outside of the scope of this study). There was no evidence of differences across demographics in MCT uptake intention. In contrast, we find substantial heterogeneity. This may be due to the high reported MCT uptake intention (94%) and hence limited variation in the outcome. However, that study did find associations of intention with both previous screening and cancer attitudes, which aligns with our findings on cancer knowledge and experience. Our findings on the importance of PPV and NPV align with those of Gelhorn et al. [38]; though the number of cancers was more important to individuals in our findings. That study did not include detecting cancer at an early stage, which was important to individuals in our study, particularly those who have knowledge and experience of cancer. A focus group study in the US [24] explored perceptions of clinicians and the general public. They found similar result to ours in that test accuracy was important (to avoid malpractice in the case of clinicians, and anxiety in the case of patients), as was early detection of cancer. The authors report that ease of use was important to both clinicians and the public, which accords with our finding of a negative preference for a faecal test.

### Implications for research and practice

Incorporating public and GP preferences in the development of MCTs could reduce subsequent obstacles to adoption and uptake of MCTs in clinical practice, and improve cost-effectiveness in real-world settings, which could be appealing to device manufacturers and healthcare system payers [2, 41]. We initially planned to sample the preferences of these two groups. However, following consultation with subject experts, it was deemed that there would likely be insufficient respondents to yield reliable estimates (*n* < 10). Future work should elicit the preferences of these groups, for example as expert stakeholders contributing to MCT Target Product Profile development.

Our elicited differences in preference noted by demographic characteristic may inform efforts to promote equitable access to early cancer detection tests. For example, those who reported their ethnicity as non-white had no extra preference for MCTs that could detect more (versus fewer) cancer sites. For these groups, the evidence generated may lead to the development of MCTs that are not only efficacious but acceptable, possibly leading to increased uptake. Knowing features valued by members of the general public and primary care physicians may also aid in decisions on where to place MCTs in the cancer diagnosis pathway, motivating further research into the combination of MCT features that could aid GPs in making decisions about referral and patient management. For example, we have that GPs place a much higher value than the public on avoiding missed cancers than incorrectly investigating people without cancer. Our findings may therefore inform decisions by device manufacturers, NHS commissioners, and private insurers concerning adoption of MCTs in clinical practice.

GP and public preferences for MCTs are similar regarding preferred test attributes, but differ in magnitude, leading to more pronounced preferences on the part of GPs. For the general public, we found substantial preference variation by ethnicity and experience of cancer. These findings provide a basis for designing clinical implementation strategies for MCTs, according to their performance characteristics.

## Supplementary information

Supplementary materials

## Data Availability

The data that support the findings of this study are available from the authors upon reasonable request subject to the permission of funding bodies.
