# Correction: Preferences for multi-cancer tests (MCTs) in primary care: discrete choice experiments of general practitioners and the general public in England

**DOI:** 10.1038/s41416-025-03213-z

**Published:** 2025-12-01

**Authors:** John Buckell, Nomalanga Madyiwa, Gail Hayward, Mark R. Middleton, FD Richard Hobbs, James Buchanan, Apostolos Tsiachristas, Brian D. Nicholson

**Affiliations:** 1 Health Economics Research Centre, Oxford Population Health, University of Oxford, Oxford, England; 2 Buckinghamshire Healthcare NHS Trust, Ayelsbury, England; 3 Nuffield Department of Primary Health Care Sciences, University of Oxford, Oxford, England; 4 Department of Oncology, University of Oxford, Oxford, England; 5 Health Economics and Policy Research Unit, Centre for Evaluation and Methods, Wolfson Institute of Population Health, Queen Mary University, London, England

**Keywords:** Cancer, Economics

Correction to: *British Journal of Cancer* 10.1038/s41416-025-03063-9, Article published online 2 June 2025

Following publication of the article, errors were found in Fig 3. During the production process for the article, the correct version of the figure (which had been present throughout the editorial assessment and peer review process) was inadvertently replaced with an incorrect figure file.

The article has been corrected; the correction does to affect the results or conclusions or the article.

Correct figure:
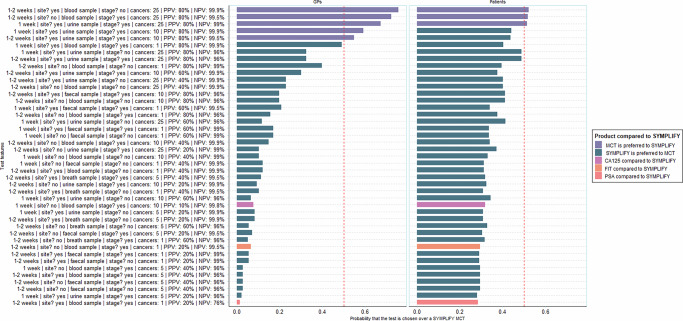


Original figure:
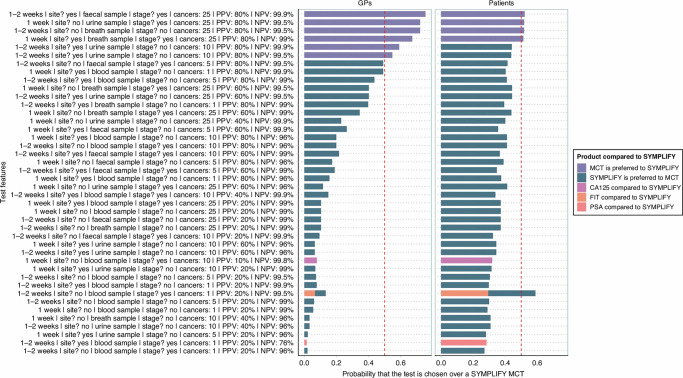


The original article has been corrected.

