# Supplementary material for: Preferences for multi-cancer tests (MCTs) in primary care: discrete choice experiments of general practitioners and the general public in England
Source: Br J Cancer. 2025 Jun 2;133(3):394–403. doi: 10.1038/s41416-025-03063-9 (PMC12322097; doi:10.1038/s41416-025-03063-9)
Supplement: Supplementary file 1 — Supplementary materials [file 41416_2025_3063_MOESM1_ESM.docx]

SUPPLEMENTS

Supplement 1: Descriptions of attributes and levels presented in the survey to respondents

**//////////////////////////////// GPs**

*Multi-cancer early detection tests have different features – for example, the risk of cancer after a positive test, the risk of cancer after a negative test, uncertainty in the evidence, waiting time for results, number of cancer sites tested for, ability of the test to indicate cancer site and form of the test.*

*We would like you to answer 12 choice questions with options of multi-cancer early detection tests*, *where each test is described by 6 features.*

*Please read the information below about each of the features carefully - you will use this information when you answer the choice questions.*

1. **Risk of cancer after a positive test.** The number of people who have cancer when they have a positive MCED test result. A lower risk percentage, such as 20%, indicates that 2 in 10 people who test positive have cancer. A higher risk percentage, like 80%, indicates that 8 in 10 people who test positive have cancer.

*The options for this characteristic are:*

*- 20%: 2 in 10 people who test positive have cancer*

*- 40%: 4 in 10 people who test positive have cancer*

*- 60%: 6 in 10 people who test positive have cancer*

*- 80%: 8 in 10 people who test positive have cancer*

1. **Risk of cancer after a negative test.** The number of people who have cancer when they have a negative MCED test result. At 0%, nobody who tests negative has cancer. Higher risk percentages, like 4%, suggest 4 in 100 people who test negative have cancer.

*The options for this characteristic are:*

*- 0.1%: 1 in 1000 people testing negative have cancer*

*- 0.5%: 1 in 200 people testing negative have cancer*

*- 1%: 1 in 100 people testing negative have cancer*

*- 4%: 4 in 100 people testing negative have cancer*

1. **Waiting time for results.** The number of days it takes for the GP to receive the results of the MCED test.

*The options for this characteristic are:*

*· within 1 week*

*· 1 to 2 weeks*

1. **Number of cancer sites tested.** The number of specific cancer sites that the test is designed to detect.

*The options for this characteristic are:*

*- 1 site*

*- 5 sites*

*- 10 sites*

*- 25 sites*

1. **Can the test identify the site?** Can the MCED test pinpoint the location or organ from which the cancer signal originates (for example the lung or bowel)?

*The options for this characteristic are:*

- *Yes*
- *No*

1. **Form of the test.** The type of sample that the MCED test requires.

*The options for this characteristic are:*

*- Blood*

*- Breath*

*- Faeces*

*- Urine*

1. **Can the test detect the stage of cancer?** Can the MCED test distinguish between early (stage 1 or 2) or late (stages 3 or 4) cancers?

*The options for this characteristic are:*

*· Yes.*

*· No.*

**//////////////////////////////// PATIENTS**

*Multi-cancer early detection tests have different features – for example, how accurate the test is, waiting time for results, number of cancer sites tested for, ability of the test to indicate cancer site and form of the test.*

*We would like you to answer 12 choice questions with the option of choosing between two multi-cancer early detection tests, where each test is described by 5 features.*

*Please read the information below about each of the features carefully - you will use this information when you answer the choice questions.*

1a **Test gets it wrong when it tells us there is a cancer.** The test is uncertain if you have cancer. The result from these cancer tests can sometimes be wrong because they only pick up a signal for cancer. So if the test tells you that you have cancer, it may turn out to be wrong when you have further tests for cancer at the hospital.

*The options for this feature are:*

*· Gets it wrong OFTEN (8 out of ten times)*

*
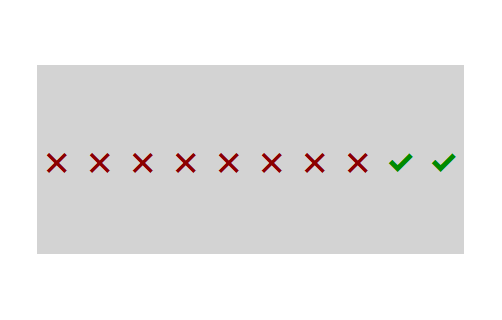
*

*· Gets it wrong QUITE OFTEN (6 out of ten times)*

*
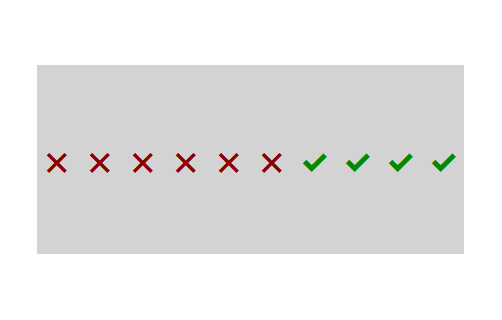
*

*· Gets it wrong SOMETIMES (4 out of ten times)*

*
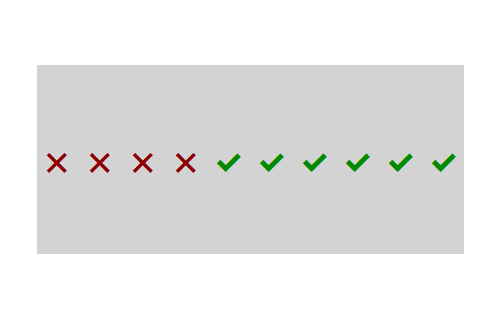
*

*· Gets it wrong RARELY (2 out of ten times)*

*
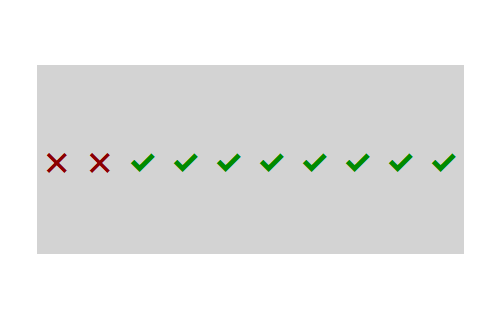
*

1b **Test gets it wrong when it tells us there is not a cancer.** Test is uncertain if you do not have cancer. The result from these cancer tests can be wrong because they only pick up a signal for the cancer. So it may tell you that you do not have cancer, when in fact you do. It is rare that the test result of “no cancer” is wrong, but it does happen.

*The options for this feature are:*

*· Gets it wrong ALMOST NEVER (1 in 1000 times)*

*
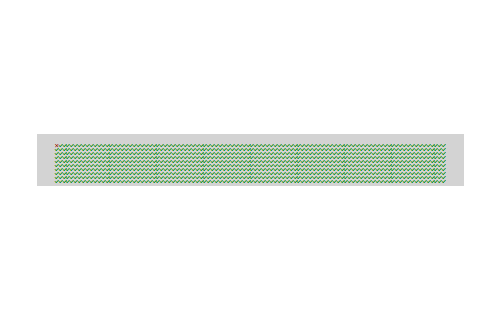
*

*· Gets it wrong INCREDIBLY RARELY (5 in 1000 times)*

*
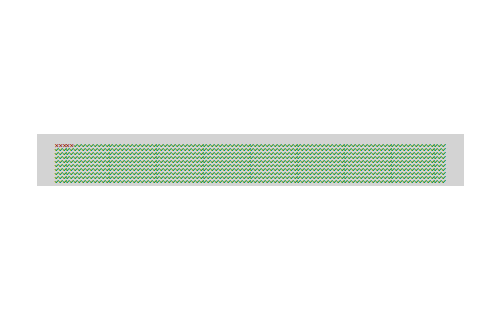
*

*· Gets it wrong VERY, VERY RARELY (10 in 1000 times)*

*
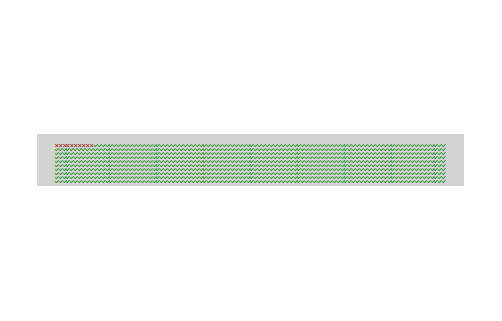
*

*· Gets it wrong RARELY (40 in 1000 times)*

*
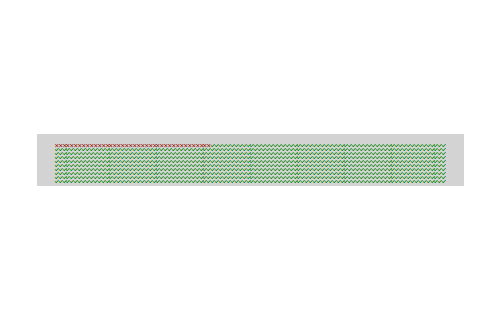
*

1. **Waiting time for results.** The number of days it takes for the GP to receive the results of the MCED test.

*The options for this feature are:*

*· within 1 week*

*· 1 to 2 weeks*

1. **Number of cancers (e.g. bowel, skin, or lung) tested for.** The number of specific cancer types that the test is designed to detect.

*The options for this feature are:*

*· 1*

*· 5*

*· 10*

*· 25*

1. **Test detects type of cancer (e.g. bowel, skin, or lung)?** Can the MCED test pinpoint the location or organ from which the cancer signal originates? For example the lung or bowel.

*The options for this feature are:*

*· Yes. The test is able to tell you the type of cancer.*

*· No. The test is not able to tell you the type of cancer.*

1. **Form of the test.** The type of sample that the MCED test requires.

*The options for this feature are:*

*· Blood test. The test will need a blood sample to be taken from you.*

*· Faecal test. The test will need a faecal (poo) sample from you.*

*· Urine test. The test will need a urine (wee) sample from you.*

*· Breath.* *The test will need a breath sample from you.*

**6 Test detects cancer at an early stage?**If cancer is detected at an earlier stage, (called stage 1 or 2) people are more likely to survive than if it is detected at a later stage (called stage 3 or 4).

*The options for this feature are:*

*· Yes. The test can tell you if the cancer is early or late*

*· No. The test cannot tell you if the cancer is early or late*

Supplement 2: example of experimental choice tasks and randomization schematic.


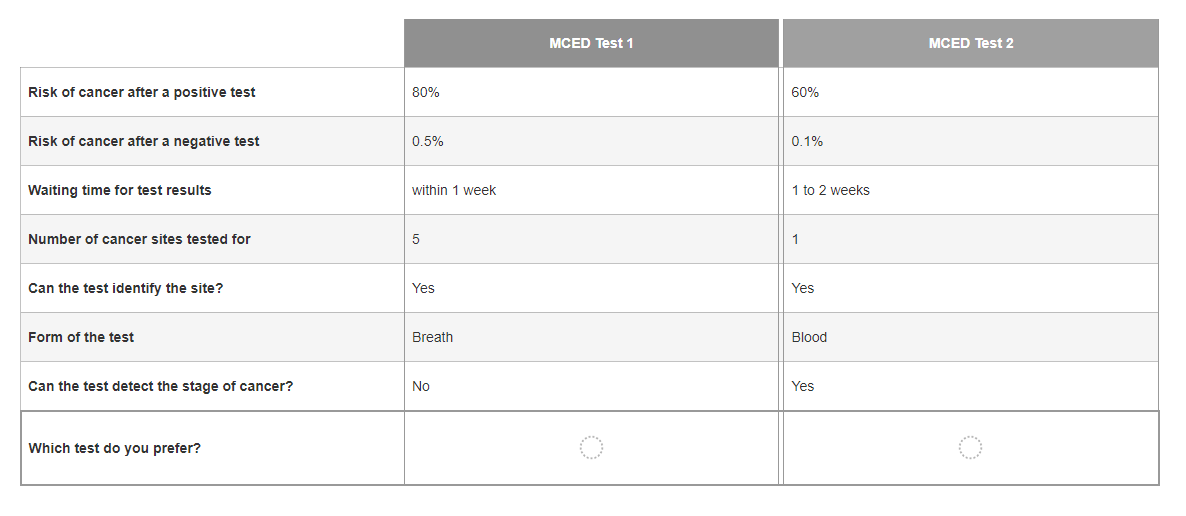


Fig S1: Example choice task from GP survey


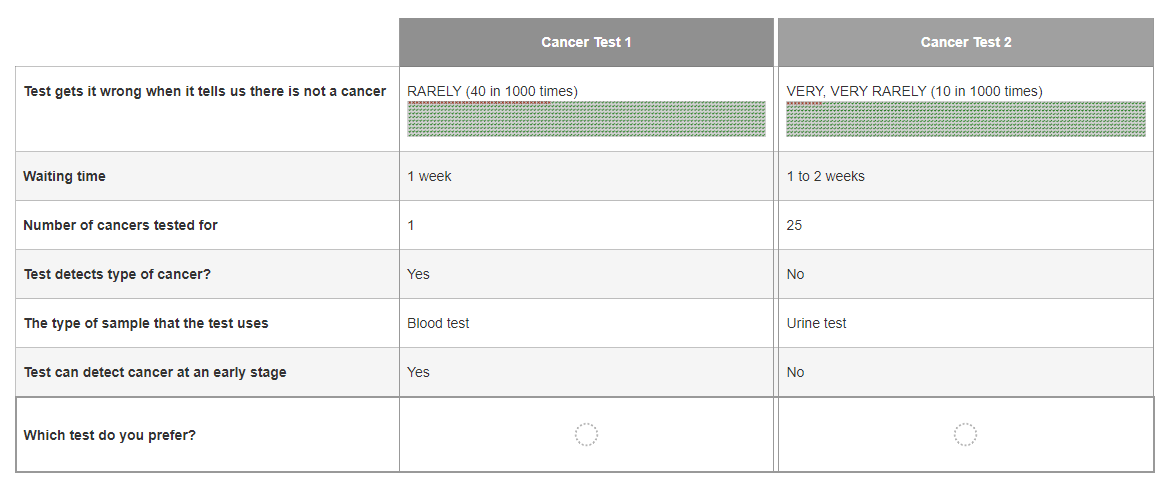


Fig S2: Example choice task from patient survey, negative risk attribute


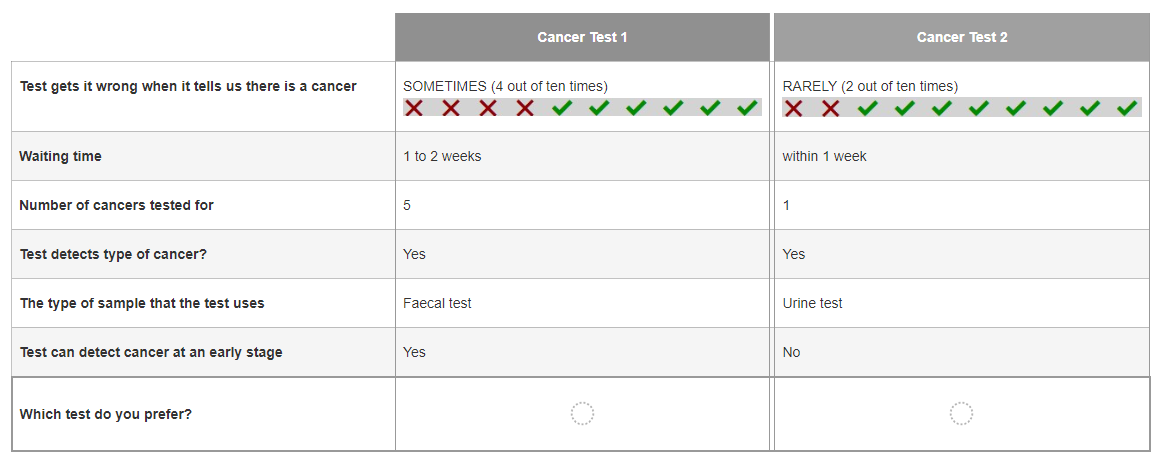


Fig S3: Example choice task from patient survey, positive risk attribute


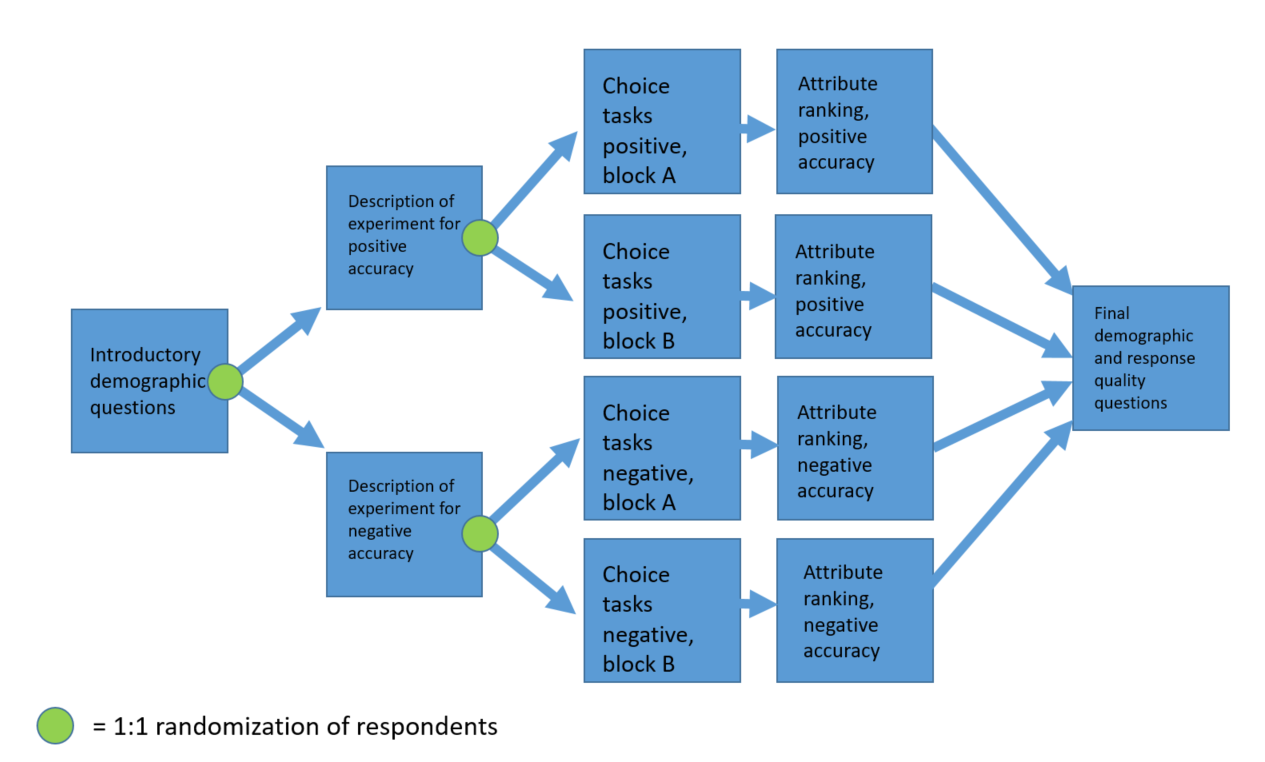


Fig S4: Patient survey flow schematic

Supplement 3: Breakdown of survey response quality checks

|  |  | GPs | Patients |
| --- | --- | --- | --- |
|  |  |  |  |
| *Invited participants* |  |  |  |
| Total |  | 6757 | 1465 |
|  |  |  |  |
| *Started the survey* |  |  |  |
| Total |  | 812 | 1413 |
|  |  |  |  |
| *Participants removed* |  |  |  |
| Failed eligibility |  | 66 | 70 |
| Timed out |  | 104 | 158 |
| Failed speeding checks |  | 0 | 7 |
| Duplicate IDs |  | 0 | 0 |
| Failed open-ended sense check |  | 0 | 4 |
|  |  |  |  |
| *Exceed quotas* |  |  |  |
| Over quota |  | 391 | 169 |
|  |  |  |  |
| Final sample |  | 251 | 1005 |
|  |  |  |  |

There were 812 GPs who started the survey, 561 surveys were excluded (n=66 were not eligible and were screened out, n=391 were over the predetermined quota, and n=104 were timed out). Thus, a total of 251 GP responses were included in the analysis.

A total of 1413 respondents started the general public survey. Out of these, 404 respondents were excluded from the final sample (n=169 were above the required quotas, n=70 were ineligible, n=158 were timed out, n=7 failed the speeder check, and n=4 were excluded due to illogical responses.

Supplement 4: choice modelling

Based on McFadden (1974), respondents were assumed to maximize their utility in making DCE choices. In this formulation, individuals reconcile their attribute preferences for each of the available MCED tests and choose that which maximizes their utility; that is, choosing their preferred MCED test as they are described by the attributes in each choice task. Respondents’ utility is a linearly-additive function of attribute preferences.

$U_{ni}= V_{ni}+\varepsilon_{ni}=\sum_{m}^{M} \beta_{m}\cdot x_{im}+\varepsilon_{ni}$ (1)

$U_{ni}$ is the utility for decision-maker *n* of MCED test $i$, comprising deterministic and random utility$; V_{ni}$ is the deterministic component of utility; $\varepsilon_{ni}$ is the random component of utility; $x_{im}$ is the *m*th attribute-level of alternative $i$; and $\beta_{m}$ is the $m$th preference parameter to be estimated. The deterministic component of utility comprises preferences for MCED test attributes, and survey artefacts (left-to-right bias):

$$V_{ni}+\varepsilon_{ni}$$

(2)

ASC denotes an alternative-specific constant. is an indicator variable taking the value of 1 if the MCED test is the left-hand alternative, 0 otherwise; accounts for a tendency to choose alternatives presented on the left more often than those presented on the right. $\beta$ are attribute-specific preference parameters. , , , and are categorical variables. For these attributes, the levels are dummy-coded meaning that one is set to zero as a reference category against which preferences for the other levels are measured. There are then $L-1$ estimated parameters for each attribute, where $L$ is the number of levels for that attribute. For example, for “form of the test” has 4 levels, “blood”, “faecal”, “urine” and “breath”. We set “blood” to zero and estimate three parameters (i.e., $L-1=4-1=3$) for the levels, “faecal”, “urine” and “breath”. These measure, respectively, the preference for a faecal MCED test versus a blood-sample MCED test, a urine MCED test versus a blood-sample MCED test, and a breath MCED test versus a blood-sample MCED test. , , and are continuous variables. Parameters, e.g. , measure the change in utility of a one-unit change in that variable.

Estimation is operationalized by assuming a type-I extreme value error distribution on the error term and estimating choice probabilities for each alternative with a multinomial logit (MNL) model.

$P_{ni}=\frac{exp(V_{ni})}{\sum_{j=1}^{n} exp(V_{nj})}$ (3)

where $P_{ni}$ is the probability that respondent *n* chooses MCED test $i$ from choice task $J$. In our data, every choice task had two MCED tests, thus $J=2 \forall n$.

Scale corrections

Given that different sections of the sample had different elements of the designs, a correction to the scale is made to account for any unobserved differences between groups (Buckell and Hess, 2019). He scale enters through a multiplication of $V_{ni}$:

$$P_{ni}=\frac{exp(\sigma V_{ni})}{\sum_{j=1}^{n} exp({\sigma V}_{nj})}$$

Where

$$\sigma= \sigma_{negative}*design\_negative+ \sigma_{GP}*GP\_sample$$

$design\_negative$ is a binary variable taking the value of 1 if the individual was assigned to the design with the negative test accuracy attribute; 0 otherwise. $GP\_sample$ is a binary variable taking the value of 1 if the individual is a GP; 0 if the individual is a patient. $\sigma_{negative}$ and $\sigma_{GP}$ are estimated, capturing scale differences between the two patient designs and the GP versus the patient design, respectively.

Preference heterogeneity

Preference variation for MCED test attributes was modelled deterministically and randomly. For deterministic heterogeneity, the attributes were interacted with individuals’ characteristics of age, gender, ethnicity, education, and rurality; a latent variable capturing patient knowledge and experiences of cancer; and preferences of GPs. These parameters indicate MCED test attribute preference variation according to those characteristics. Random preference heterogeneity was modelled using mixing distributions (Train, 2009). Parameters were treated as a triangular distributions, thus each has a midpoint and a range to be estimated.

Combining the forms of heterogeneity, taking attribute (level) “test can detect stage of cancer? (yes, reference category: no)” as an example, we have,

$\theta_{stage}+{\gamma_{stage}'Z}_{i}+\tau_{stage}.{LV}_{experirence}$ (5)

Where $\theta_{stage}$ is a symmetrical triangular distribution with the midpoint, $\mu_{stage}$, and range, ${range}_{stage}$: $\theta_{stage}\sim T[\mu_{stage},{range}_{stage}]$. Taking two uniform draws, the range of the distribution is computed as ${range}_{stage}*(d_{U1}-d_{U2})$ where $d_{U1}$ and $d_{U2}$ are draws from independent uniform distributions; $\mu_{stage},{range}_{stage}$ are parameters to be estimated. Triangular distributions were specified for all attributes, and 500 draws were taken using the Modified Latin Hypercube Sampling algorithm (Hess et al., 2006). $Z_{i}$ are three individual characteristics and $\gamma_{stage}$ are deterministic heterogeneity parameters measuring if preferences vary for MCED tests; for example, if women’s preferences for MCED tests that detect cancer at an early stage are different to men’s. ${LV}_{experirence}$ is a latent variable for cancer knowledge and experience (see below for details); $\tau_{stage}$ is a parameter that measures preference variation by cancer experience; for example, whether people with more knowledge and experience of cancer have stronger preferences for MCED tests that detect cancer at an early stage than those with less knowledge and experience. Models were specified with all interactions on covariates and all mixing distributions. Models were then refined to remove non-significant parameters for a parsimonious specification.

Latent variable for cancer knowledge and experience

Whereas interacting individual characteristics (age, gender, etc.) is straightforward and considered standard practice in a choice model, interacting the responses to attitudinal/experiential survey questions raises a number of issues (Ben-Akiva et al., 2022). In this case, several questions were asked about patients’ knowledge and experiences of cancer. It is not clear which of the variables should enter the utility function. Using all of them together may induce collinearity (since they are likely to be correlated); yet using a subset means information in the data may be lost. This approach proliferates parameters, and models quickly become unwieldy. It is also not clear how these questions are able to capture the underlying concept of interest; that is, they are indirect, imperfect measures, e.g., of patients’ experience of cancer. They may also be subject to varying interpretation (one person’s score of 6 on a scale of 1 to 10 may be another person’s 7 or 8), which may result in measurement error.

Using latent variables within an integrated choice and latent variable framework (ICLV; Walker, 2001) obviates the issue that the variables are not direct measures of the concept of interest, by treating the attitudinal/experiential variables as indicators of an underlying latent variable. Their function is to explain utility (and ultimately choices) and to explain all of the attitudinal/experiential variables at the same time (by doing so, they are able to use all of the information in the data). They thus reduce the dimensionality of the choice model because only one variable is inserted into the utility function for each latent variable (as opposed to multiple questions individually). In addition, they avoid issues of collinearity that would likely arise if the attitudinal/experiential variables would enter together in the utility function. In addition, it is possible to explain the latent variables with individual characteristics, which is useful behavioural information.

Indicators of latent variables took one of three forms: binary variables (e.g., yes/no for “Have you ever had cancer?”), ordered variables (e.g., Likert scale of 1 to 4 for “I am anxious or distressed about cancer”), or continuous variables (e.g., scalar response from 0 to 11 for “How many of the following symptoms do you think indicates a possible cancer?”). Taking these three as examples of indicators of the knowledge and experiences of cancer latent variable, they are specified in measurement equations to estimate the relationship between the latent variable and its indicator variables.

Binary variables use a logit,

$P_{had cancer}=\frac{exp(\delta_{had cancer}+\zeta_{had cancer} \cdot{LV}_{experience})}{1+exp(\delta_{had cancer}+\zeta_{had cancer} \cdot{LV}_{experience})}$ (7)

Where $\delta_{had cancer}$ is a constant term to be estimated, ${LV}_{experience}$ is the latent variable for knowledge and experiences of cancer, and $\zeta_{had cancer}$ measures the relationship between the latent variable and the response to the question, in this case whether the patient has ever had cancer.

Ordered variables use an ordered logit,

$P_{cancer anxiety}= \sum_{s=1}^{S} \left. \delta(I_{cancer anxiety}==s \right)\left[ \frac{exp(\tau_{cancer anxiety, s}-\zeta_{cancer anxiety}\cdot{LV}_{experience})}{1+exp(\tau_{cancer anxiety, s}-\zeta_{cancer anxiety}\cdot{LV}_{experience})}-\frac{exp(\tau_{cancer anxiety, s-1}-\zeta_{cancer anxiety}\cdot{LV}_{experience})}{1+exp(\tau_{cancer anxiety, s-1}-\zeta_{cancer anxiety}\cdot{LV}_{experience})} \right]$ (8)

Where $\tau_{cancer anxiety, s}$ are estimated threshold parameters for threshold *s* of the climate change worry indicator, $I_{cancer anxiety}$. ${LV}_{experience}$ is the latent variable for knowledge and experiences of cancer, and $\zeta_{cancer anxiety}$ measures the relationship between the latent variable and the response to the question, in this case how anxious about cancer the patient feels.

Continuous variables use a linear model,

$P_{symptoms}=\frac{1}{\sqrt{2\pi\sigma_{symptoms}^{2}}} \cdot exp-\left( \frac{I_{symptoms}-\bar{I_{symptoms}}-\zeta_{symptoms}\cdot{LV}_{experience}}{2\sigma_{symptoms}^{2}} \right)$ (9)

Where using the demeaned variable (operationalised by subtracting $\bar{I_{cc responsibility}}$) avoids estimating a constant, and $\sigma_{symptoms}^{2}$ is the estimated error variance. ${LV}_{experience}$ is the latent variable for knowledge and experiences of cancer, and $\zeta_{symptoms}$ measures the relationship between the latent variable and the response to the question. In this case how many symptoms the individual recorded as being indicative of cancer.

Next, a structural equation for the latent variable measures the relationship between the latent variable and individual characteristics.

${LV}_{experience}=\gamma_{experience}\cdot Z_{n}+\xi_{experience}$ (10)

where $Z_{n}$ are individual characteristics, $\gamma_{experience}$ capture the relationships between the latent variable for knowledge and experiences of cancer and individual characteristics. $\xi_{experience}$ captures random heterogeneity following a standard normal distribution with zero mean and unit variance, $\xi_{experience}\sim N(0,1)$.


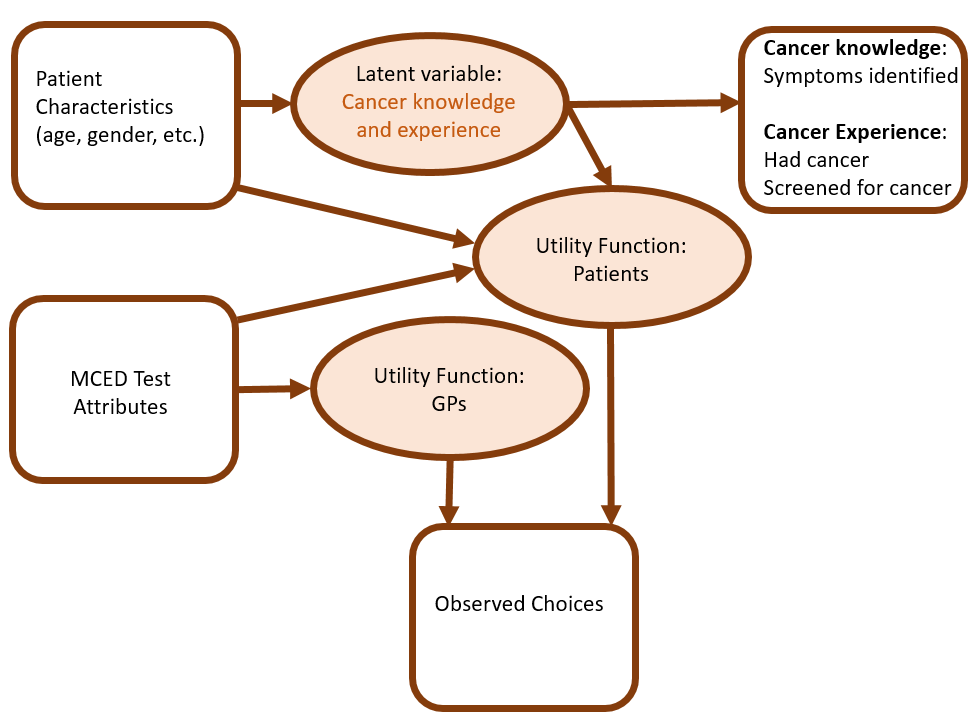


Figure S5: Schematic of ICLV model. Boxes are observed in the data; ellipses are inferred in analyses. The experiment varied the MCED test attributes which shape the utility for both patients and GPs (cf. equation (2)). In turn, utilities explain choices (equation (3)). Patient utilities are further explained by patient characteristics and the latent variable for cancer knowledge and experience (equation (5)). Patient characteristics also explain the latent variable for cancer knowledge and experience (equation (10)). Finally, the latent variable for cancer knowledge and experience explains the indicator measures of it (i.e. survey questions; equations (7)-(9)).

Joint estimation

With the addition of the latent variables, the models for the DCE, the measurement equations, and the structural equations are combined in a single system of equations. In this setup, the latent variable explains each of the indicators. Each latent variable is explained by individuals’ characteristics. The latent variable enters the utility function to explain how cancer knowledge and experiences shape preferences for MCED tests.

Given both the mixing distributions and error terms on the latent variables, the resulting likelihood function no longer has a closed form solution. To facilitate estimation, we use simulations and integrate over both the mixing distributions and the latent variable to approximate the likelihood function.

$SLL=w_{n}\sum_{nt} \log\int_{\beta} \int_{LV} P_{ni}P_{binary}P_{ordered}P_{linear}\phi(\zeta)m(\beta)d\beta dLV$(11)

where SLL is the simulated log-likelihood. $w_{n}$is an individual-specific, post-stratification weighting, the equalises the contribution of patients and GPs to the likelihood function. $P_{ni}$ is the choice probabilities for the experimental MCED choices. $P_{binary}$, $P_{ordered}$, and $P_{linear}$ are probabilities for the measurement equations of binary, ordered and continuous indicator variables. 500 draws were taken using the Modified Latin Hypercube Sampling algorithm (Hess et al., 2006). Robust standard errors are computed using the sandwich estimator. All models were estimated using simulated maximum likelihood with Bunch, Gay and Welch (1993), BGW, optimisation in the Apollo package for R (Hess and Palma, 2019).

Simulations for ranking MCED tests

To operationalise each simulation, we set the attributes for “MCED 2” to be those of the FIT, then based on equation 2, we would have $V_{FIT}$. We can then compare any other policy to that by changing “MCED 1” attributes computing a V. If we define the MCED of interest to $V_{MCED}$, then we can compute the probability that this is preferred to the SDIL by using equation 4, such that,

$P_{health policy}=\frac{exp(V_{MCED})}{\sum_{j=1}^{J} exp(V_{mced,nj})}=\frac{exp(V_{MCED})}{\exp\left( V_{MCED} \right)+exp(V_{FIT})}$ (12)

It follows that,

$P_{FIT}=\frac{exp(V_{FIT})}{\exp\left( V_{FIT} \right)+exp(V_{FIT})}= \frac{1}{2}=0.5$ (13)

That is, the probability of choosing the FIT compared to the FIT is exactly 50% since it is the same thing. So, if a policy is more popular than the FIT, $V_{MCED}> V_{FIT}$, and the probability is higher than 0.5. If a policy is less popular than the FIT, $V_{MCED}< V_{FIT}$, and the probability is lower than 0.5.

Note, however, that using FIT as a benchmark is irrelevant because in a choice model, only relative differences matter for identification. The implication of this is that the order of predicted probabilities of the MCEDs is independent of the choice of comparator.

Simulations used sample enumeration (15) with 95% Krinsky-Robb confidence intervals (16).

Robustness and Sensitivity analyses

Our estimated preferences aligned with the reported ranking of preferred attributes in follow-up questions. Mixed multinomial logit models were preferred to more basic multinomial logit models on the basis of improvements in model fit (Vuong test for non-nested models), allowing for heterogeneity in respondents’ preferences, and mitigating the risk of parameter bias. Triangular mixing distributions were preferred to normal mixing distributions, though the estimated means of distributions in both cases was very similar.

In preliminary modelling, preferences were tested across the randomization of patients to positive or negative predictive value attributes. We modelled the data separately for both arms. We then computed marginal rates of substitution (which is the ratio of any two parameters) for all attributes using number of cancers detected as the denominator. Such a computation is needed to make appropriate (i.e. to avoid the parameter-scale confound inherent in these models^36^) comparisons across separate MNL models. No differences were found, that is, patient preferences were stable irrespective of randomisation to PPV of NPV. See supplement 6.

In pilot data, we found evidence that some individuals preferred lower PPV to higher. We investigated this in the full data using a latent class model with four classes. We found no evidence of this behaviour in the full data. See supplement 7.

A model interacted GP preferences for MCTs with their characteristics: gender, ethnicity, years of practice, practice size and urbanicity. After a process of refinement (removing non-significant parameters), we found that GPs in small practices had a lower preference for faecal tests, versus blood tests, than those in larger practices. No other preference heterogeneity was found. See supplement 11.

We tested for level effects in MRSs for the general public and GPs. No evidence was found. See supplement 12.

Supplement 5: figures used for simulation of currently used cancer tests

| **Characteristic** | **Galleri** | **CA125** | **FIT** | **PSA** |
| --- | --- | --- | --- | --- |
| PPV | 75.5% | 10.1% | 19.4% | 19% |
| NPV | 97.6% | 99.8% | 99.5% | 76% |
| Waiting time | 1-2 weeks | 1 week | 1-2 weeks | 1-2 weeks |
| Number of sites detected | 25 | 10 | 1 | 1 |
| Test can identify site | Yes | no | yes | yes |
| Form of the test | Blood | blood | faecal | blood |
| Can detect early stage | no | yes | yes | yes |

Supplement 6: testing for consistency in preferences across PPV and NPV randomization for patients.

PPV


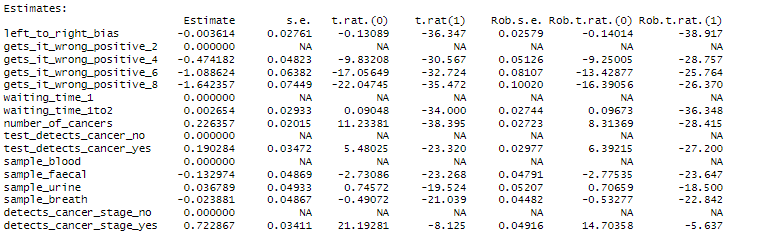


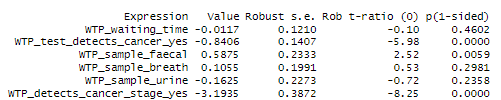


NPV


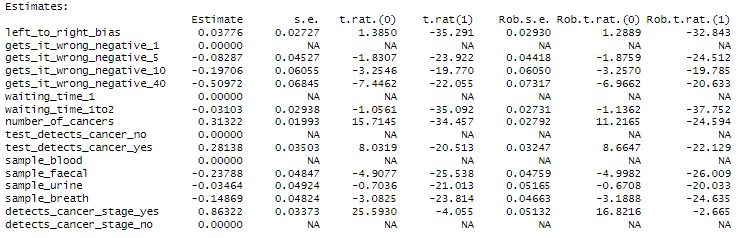


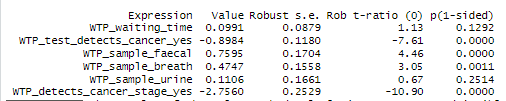


Supplement 7: Latent class model for testing directionality of PPV attribute preferences


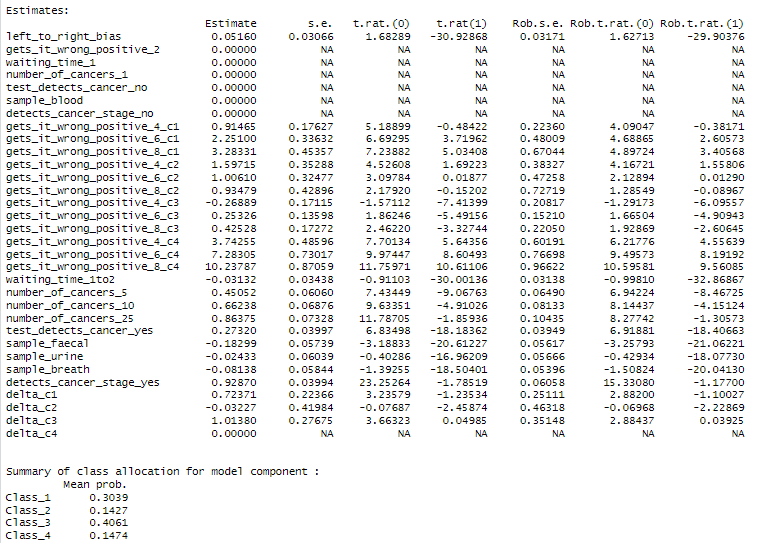


Supplement 8: Mixed logit model including interactions with GPs

|  |  | Estimate | Rob.std.err. | Rob.t-ratio(0) |
| --- | --- | --- | --- | --- |
|  |  |
|  |  |  |  |  |
| Left To Right Bias |  | -0.06 | 0.03 | -1.80 |
|  |  |  |  |  |
| PPV: Midpoint |  | -0.50 | 0.04 | -12.94 |
| NPV: Midpoint |  | -0.01 | 0.00 | -4.57 |
| Waiting Time 1 |  | 0.00 | NA | NA |
| Waiting Time 1To2: Midpoint |  | 0.04 | 0.03 | 1.33 |
| Number Of Cancers: Midpoint |  | 0.04 | 0.00 | 9.98 |
| Test Detects Cancer No |  | 0.00 | NA | NA |
| Test Detects Cancer Yes: Midpoint |  | 0.30 | 0.04 | 8.25 |
| Blood |  | 0.00 | NA | NA |
| Faecal: Midpoint |  | -0.15 | 0.06 | -2.59 |
| Urine: Midpoint |  | 0.01 | 0.05 | 0.23 |
| Breath: Midpoint |  | -0.05 | 0.05 | -1.07 |
| Detects Cancer Stage No |  | 0.00 | NA | NA |
| Detects Cancer Stage Yes: Midpoint |  | 1.13 | 0.08 | 14.35 |
|  |  |  |  |  |
| PPV: Range |  | -0.40 | 0.03 | -13.51 |
| NPV: Range |  | 0.03 | 0.00 | 10.28 |
| Waiting Time 1To2: Range |  | 0.10 | 0.06 | 1.70 |
| Number Of Cancers: Range |  | 0.04 | 0.00 | 10.14 |
| Test Detects Cancer Yes: Range |  | -0.34 | 0.07 | -5.13 |
| Faecal: Range |  | 0.39 | 0.06 | 5.98 |
| Urine: Range |  | -0.23 | 0.07 | -3.30 |
| Breath: Range |  | -0.21 | 0.08 | -2.51 |
| Detects Cancer Stage Yes: Range |  | 0.76 | 0.05 | 15.47 |
|  |  |  |  |  |
| Mu: GP |  | 1.00 | NA | NA |
| Mu: Positive |  | 1.00 | NA | NA |
| Mu: Negative |  | 1.11 | 0.10 | 11.58 |
|  |  |  |  |  |
| PPV: GP |  | -0.52 | 0.07 | -7.82 |
| NPV: GP |  | -0.06 | 0.01 | -9.69 |
| Waiting Time 1To2: GP |  | 0.00 | NA | NA |
| Number Of Cancers: GP |  | 0.04 | 0.01 | 4.32 |
| Test Detects Cancer Site Yes: GP |  | 0.42 | 0.11 | 3.88 |
| Faecal: GP |  | 0.00 | NA | NA |
| Urine: GP |  | 0.00 | NA | NA |
| Breath: GP |  | 0.00 | NA | NA |
| Detects Cancer Stage Yes: GP |  | -0.69 | 0.12 | -5.80 |
|  |  |  |  |  |
|  |  |  |  |  |
| Individuals |  | 1256 | | |
| Observations |  | 15072 | | |
| Number of Model Components |  | 2 | | |
| Number of Estimated Parameters |  | 25 | | |
| MLHS draws |  |  | 500 |  |
| Log-Likelihood |  | -6619.39 | | |
| AIC |  | 13288.78 | | |
|  |  |  |  |  |

Mixed logit model including interactions with GPs. Rob.std.err - robust standard error, Rob t-ratio(0) – robust t-ratio versus 0. Parameters “midpoint” are the estimated means of triangular distributions; parameters “range” are the estimated ranges of the triangular distributions. MLHS draws – number of modified Latin hypercube sampling draws. Parameters “mu” are parameters capturing differences in scale between PPV/NPV randomisation for patients and patients and GP samples.

GP-specific preferences:

| Expression | Value | Robust s.e. | Rob t-ratio (0) | p(1-sided) | LCB | UCB |
| --- | --- | --- | --- | --- | --- | --- |
|  |  |  |  |  |  |  |
| PPV: GP | -1.0128 | 0.0666 | -15.21 | 0 | -1.143336 | -0.882264 |
| NPV: GP | -0.0719 | 0.0062 | -11.61 | 0 | -0.084052 | -0.059748 |
| Number Of Cancers: GP | 0.0725 | 0.0085 | 8.55 | 0 | 0.05584 | 0.08916 |
| Test Detects Cancer Site Yes: GP | 0.7179 | 0.107 | 6.71 | 0 | 0.50818 | 0.92762 |
| Detects Cancer Stage Yes: GP | 0.4401 | 0.0968 | 4.55 | 0 | 0.250372 | 0.629828 |
|  |  |  |  |  |  |  |

Supplement 9: Integrated choice and latent variable (ICLV) model

|  |  | Estimate | Rob.std.err. | Rob.t-ratio(0) |
| --- | --- | --- | --- | --- |
|  |  |
|  |  |  |  |  |
| Left To Right Bias |  | -0.06 | 0.03 | -1.79 |
|  |  |  |  |  |
| PPV Midpoint |  | -0.45 | 0.06 | -7.93 |
| PPV Range |  | 1.20 | 0.12 | 9.90 |
| PPV Female |  | 0.00 | NA | NA |
| PPV Older |  | 0.19 | 0.12 | 1.58 |
| PPV Ethnic minority background |  | -0.16 | 0.13 | -1.27 |
| PPV Higher Education |  | 0.09 | 0.07 | 1.32 |
| PPV Rural |  | 0.00 | NA | NA |
| PPV Urban |  | 0.00 | NA | NA |
| PPV Experience |  | -0.32 | 0.07 | -4.69 |
|  |  |  |  |  |
| NPV Midpoint |  | -0.01 | 0.00 | -4.28 |
| NPV Range |  | -0.10 | 0.01 | -9.19 |
| NPV Female |  | 0.00 | NA | NA |
| NPV Older |  | 0.00 | NA | NA |
| NPV Ethnic minority background |  | 0.00 | NA | NA |
| NPV Higher Education |  | 0.00 | NA | NA |
| NPV Rural |  | 0.00 | NA | NA |
| NPV Urban |  | 0.00 | NA | NA |
| NPV Experience |  | 0.00 | NA | NA |
|  |  |  |  |  |
| Waiting Time 1 |  | 0.00 | NA | NA |
| Waiting Time 1To2 Midpoint |  | 0.05 | 0.03 | 1.69 |
| Waiting Time 1To2 Range |  | -0.07 | 0.53 | -0.14 |
| Waiting Time 1To2 Female |  | 0.00 | NA | NA |
| Waiting Time 1To2 Older |  | 0.00 | NA | NA |
| Waiting Time 1To2 Ethnic minority background |  | 0.00 | NA | NA |
| Waiting Time 1To2 Higher Education |  | 0.00 | NA | NA |
| Waiting Time 1To2 Rural |  | 0.00 | NA | NA |
| Waiting Time 1To2 Urban |  | 0.00 | NA | NA |
| Waiting Time 1To2 Experience |  | 0.00 | NA | NA |
|  |  |  |  |  |
| Number Of Cancers Midpoint |  | 0.04 | 0.00 | 10.96 |
| Number Of Cancers Range |  | 0.14 | 0.01 | 10.69 |
| Number Of Cancers Female |  | 0.00 | NA | NA |
| Number Of Cancers Older |  | 0.00 | NA | NA |
| Number Of Cancers Ethnic minority background |  | -0.04 | 0.01 | -5.03 |
| Number Of Cancers Higher Education |  | 0.00 | NA | NA |
| Number Of Cancers Rural |  | 0.00 | NA | NA |
| Number Of Cancers Urban |  | 0.00 | NA | NA |
| Number Of Cancers Experience |  | 0.00 | NA | NA |
|  |  |  |  |  |
| Test Detects Cancer: No |  | 0.00 | NA | NA |
| Test Detects Cancer: Yes Midpoint |  | 0.27 | 0.04 | 6.62 |
| Test Detects Cancer: Yes Range |  | 1.10 | 0.21 | 5.22 |
| Test Detects Cancer: Yes Female |  | 0.00 | NA | NA |
| Test Detects Cancer: Yes Older |  | 0.00 | NA | NA |
| Test Detects Cancer: Yes Ethnic minority background |  | 0.00 | NA | NA |
| Test Detects Cancer: Yes Higher Education |  | 0.00 | NA | NA |
| Test Detects Cancer: Yes Rural |  | 0.00 | NA | NA |
| Test Detects Cancer: Yes Urban |  | 0.00 | NA | NA |
| Test Detects Cancer: Yes Experience |  | 0.19 | 0.06 | 3.08 |
|  |  |  |  |  |
| Blood Test |  | 0.00 | NA | NA |
| Faecal Test Midpoint |  | -0.11 | 0.06 | -1.73 |
| Faecal Test Range |  | 1.08 | 0.30 | 3.56 |
| Faecal Test Female |  | 0.00 | NA | NA |
| Faecal Test Older |  | 0.00 | NA | NA |
| Faecal Ethnic minority background |  | 0.00 | NA | NA |
| Faecal Test Higher Education |  | 0.00 | NA | NA |
| Faecal Test Rural |  | -0.14 | 0.12 | -1.15 |
| Faecal Test Urban |  | 0.00 | NA | NA |
| Faecal Test Experience |  | 0.00 | NA | NA |
| Urine Test Midpoint |  | 0.02 | 0.05 | 0.45 |
| Urine Test Range |  | 0.76 | 0.25 | 3.03 |
| Urine Test Female |  | 0.00 | NA | NA |
| Urine Test Older |  | 0.00 | NA | NA |
| Urine Ethnic minority background |  | 0.00 | NA | NA |
| Urine Test Higher Education |  | 0.00 | NA | NA |
| Urine Test Rural |  | 0.00 | NA | NA |
| Urine Test Urban |  | 0.00 | NA | NA |
| Urine Test Experience |  | 0.00 | NA | NA |
| Breath Test Midpoint |  | -0.05 | 0.05 | -1.05 |
| Breath Test Range |  | 0.73 | 0.33 | 2.22 |
| Breath Test Female |  | 0.00 | NA | NA |
| Breath Test Older |  | 0.00 | NA | NA |
| Breath Ethnic minority background |  | 0.00 | NA | NA |
| Breath Test Higher Education |  | 0.00 | NA | NA |
| Breath Test Rural |  | 0.00 | NA | NA |
| Breath Test Urban |  | 0.00 | NA | NA |
| Breath Test Experience |  | 0.00 | NA | NA |
|  |  |  |  |  |
| Detects Cancer Stage: No |  | 0.00 | NA | NA |
| Detects Cancer Stage: Yes Midpoint |  | 1.02 | 0.10 | 10.30 |
| Detects Cancer Stage: Yes Range |  | -2.30 | 0.20 | -11.77 |
| Detects Cancer Stage: Yes Female |  | 0.00 | 0.10 | 0.03 |
| Detects Cancer Stage: Yes Older |  | 0.08 | 0.16 | 0.48 |
| Detects Cancer Stage: Yes Ethnic minority background |  | 0.00 | NA | NA |
| Detects Cancer Stage: Yes Higher Education |  | 0.00 | NA | NA |
| Detects Cancer Stage: Yes Rural |  | 0.00 | NA | NA |
| Detects Cancer Stage: Yes Urban |  | 0.00 | NA | NA |
| Detects Cancer Stage: Yes Experience |  | 0.43 | 0.07 | 5.91 |
|  |  |  |  |  |
| Mu: GP |  | 1.10 | 0.15 | 7.26 |
| Mu: Positive |  | 1.00 | NA | NA |
| Mu: Negative |  | 1.11 | 0.09 | 11.93 |
|  |  |  |  |  |
| PPV: GP |  | -0.46 | 0.10 | -4.71 |
| NPV: GP |  | -0.06 | 0.01 | -7.00 |
| Waiting Time 1To2: GP |  | 0.00 | NA | NA |
| Number Of Cancers: GP |  | 0.02 | 0.01 | 2.52 |
| Test Detects Cancer: Yes: GP |  | 0.37 | 0.11 | 3.37 |
| Faecal: GP |  | 0.00 | NA | NA |
| Urine: GP |  | 0.00 | NA | NA |
| Breath: GP |  | 0.00 | NA | NA |
| Detects Cancer Stage: Yes: GP |  | -0.60 | 0.12 | -4.80 |
|  |  |  |  |  |
| Latent Variable: Female |  | 0.35 | 0.14 | 2.52 |
| Latent Variable: Older |  | 1.03 | 0.23 | 4.42 |
| Latent Variable: Ethnic minority background |  | -1.01 | 0.16 | -6.27 |
| Latent Variable: Higher Education |  | 0.21 | 0.12 | 1.77 |
| Latent Variable: Rural |  | 0.00 | NA | NA |
| Latent Variable: Urban |  | 0.00 | NA | NA |
|  |  |  |  |  |
| Zeta Warning Sign Score |  | 1.30 | 0.28 | 4.63 |
| Tau1 Warning Sign |  | -5.46 | 0.51 | -10.65 |
| Tau2 Warning Sign |  | -0.39 | 0.16 | -2.43 |
| Tau3 Warning Sign |  | 1.46 | 0.20 | 7.34 |
|  |  |  |  |  |
| Zeta Had Cancer |  | 0.44 | 0.17 | 2.60 |
| Delta Had Cancer |  | -2.54 | 0.20 | -12.80 |
|  |  |  |  |  |
| Zeta Cancer Screening |  | 0.49 | 0.11 | 4.59 |
| Delta Cancer Screening |  | -0.83 | 0.12 | -6.89 |
|  |  |  |  |  |
|  |  |  |  |  |
| Individuals |  |  | 1256 | |
| Observations |  |  | 19092 | |
| Number of Model Components |  |  | 6 | |
| Number of Estimated Parameters |  |  | 45 | |
| MLHS draws |  |  | 500 | |
| Simulated Log-Likelihood |  |  | -8147.04 | |
| AIC |  |  | 16390.09 | |
|  |  |  |  |  |

Mixed logit model including interactions with GPs. Rob.std.err - robust standard error, Rob t-ratio(0) – robust t-ratio versus 0. Parameters “midpoint” are the estimated means of triangular distributions; parameters “range” are the estimated ranges of the triangular distributions. MLHS draws – number of modified Latin hypercube sampling draws. Parameters “mu” are parameters capturing differences in scale between PPV/NPV randomisation for patients and patients and GP samples. “Latent variable” are parameters of the structural equation showing the relationship between the latent variable and individual characteristics. For example, “Latent Variable: Female” is positive and significant, denoting that on average female patients had higher values of the latent variable than male patients. “Zeta” measure the association of the latent variable and each indicator variable. For example, “Zeta Had Cancer” is positive and significant, denoting that patients that have a higher latent variable were more likely to have had cancer.

Supplement 10: Estimated attribute preference distributions from the mixed logit model


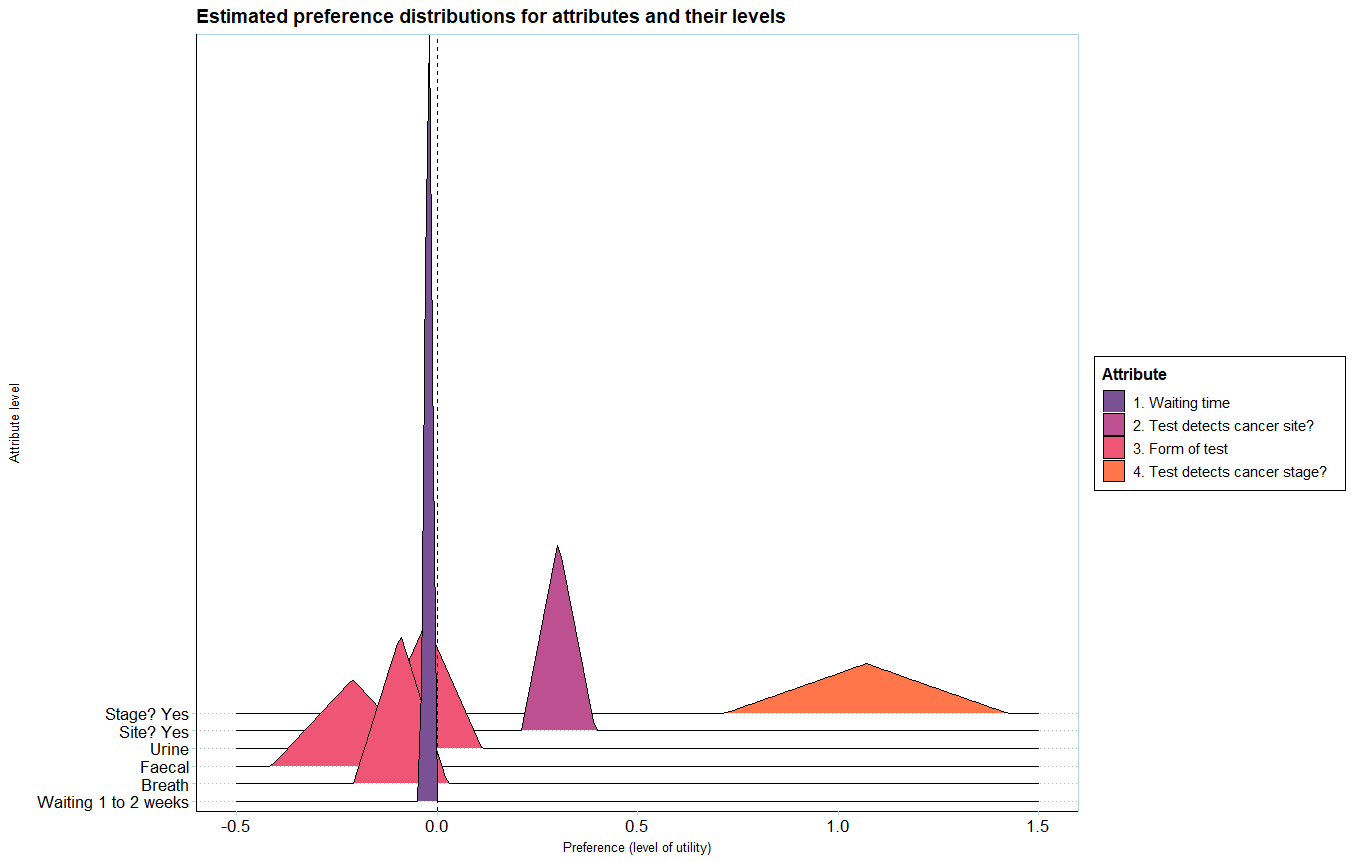


Estimated attribute preference distributions from the mixed logit model. For each attribute level, the probability density function (PDF) is plotted using the estimated midpoint and range from the mixed logit model.

Supplement 11: GP model with preference variation across demographics


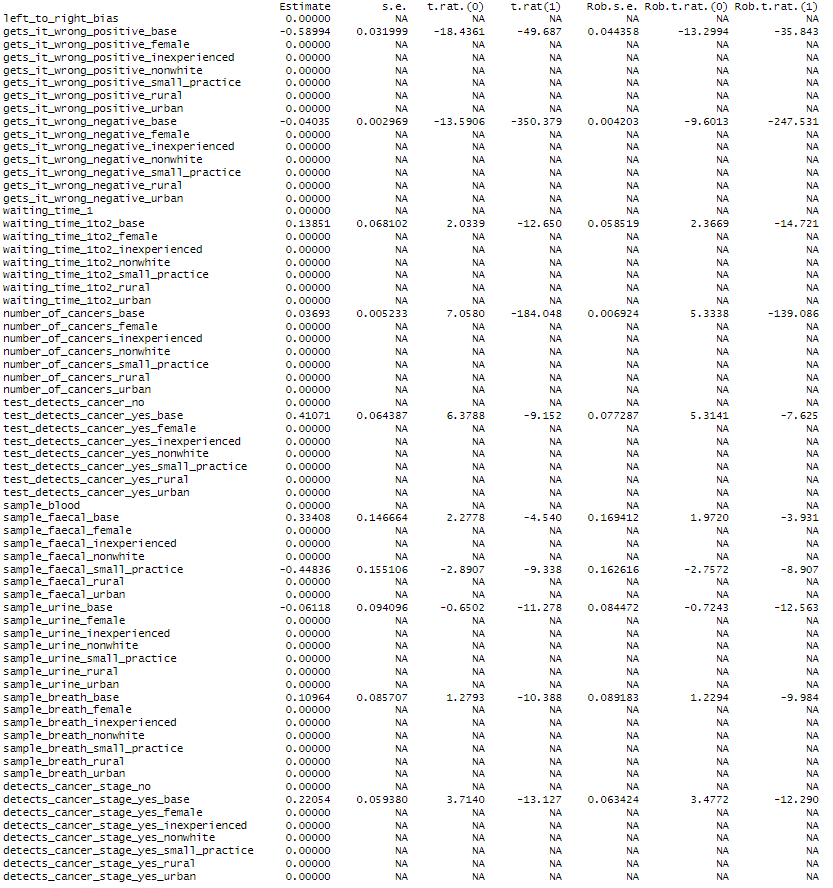


Supplement 12: Size and Level effects for MRSs

| Index | Parameter | Estimate | Robust s.e. | Rob t-ratio (0) | p(1-sided) | LCB | UCB | sample | Comparison |
| --- | --- | --- | --- | --- | --- | --- | --- | --- | --- |
| 1 | NPV 99.9% to 99.5% | PPV 40% to 20% | 1.7342 | 0.8227 | 2.11 | 0.0174 | 0.121708 | 3.346692 | General Public | Size |
| 2 | NPV 99.9% to 99.0% | PPV 40% to 20% | 3.3117 | 1.0711 | 3.09 | 0.001 | 1.212344 | 5.411056 | General Public | Size |
| 3 | NPV 99.9% to 96.0% | PPV 40% to 20% | 8.6332 | 1.6034 | 5.38 | 0 | 5.490536 | 11.77586 | General Public | Size |
| 4 | NPV 99.9% to 99.5% | PPV 60% to 20% | 0.7668 | 0.3596 | 2.13 | 0.0166 | 0.061984 | 1.471616 | General Public | Size |
| 5 | NPV 99.9% to 99.0% | PPV 60% to 20% | 1.4643 | 0.4555 | 3.21 | 7.00E-04 | 0.57152 | 2.35708 | General Public | Size |
| 6 | NPV 99.9% to 96.0% | PPV 60% to 20% | 3.8172 | 0.6231 | 6.13 | 0 | 2.595924 | 5.038476 | General Public | Size |
| 7 | NPV 99.9% to 99.5% | PPV 80% to 20% | 0.5055 | 0.2368 | 2.13 | 0.0166 | 0.041372 | 0.969628 | General Public | Size |
| 8 | NPV 99.9% to 99.0% | PPV 80% to 20% | 0.9654 | 0.2984 | 3.23 | 6.00E-04 | 0.380536 | 1.550264 | General Public | Size |
| 9 | NPV 99.9% to 96.0% | PPV 80% to 20% | 2.5167 | 0.4037 | 6.23 | 0 | 1.725448 | 3.307952 | General Public | Size |
| 10 | NPV 99.9% to 99.5% | PPV 40% to 20% | 1.7342 | 0.8227 | 2.11 | 0.0174 | 0.121708 | 3.346692 | General Public | Level |
| 11 | NPV 99.9% to 99.0% | PPV 40% to 20% | 3.3117 | 1.0711 | 3.09 | 0.001 | 1.212344 | 5.411056 | General Public | Level |
| 12 | NPV 99.9% to 96.0% | PPV 40% to 20% | 8.6332 | 1.6034 | 5.38 | 0 | 5.490536 | 11.77586 | General Public | Level |
| 13 | NPV 99.9% to 99.5% | PPV 60% to 40% | 1.3745 | 0.6505 | 2.11 | 0.0174 | 0.09952 | 2.64948 | General Public | Level |
| 14 | NPV 99.9% to 99.0% | PPV 60% to 40% | 2.6249 | 0.824 | 3.19 | 7.00E-04 | 1.00986 | 4.23994 | General Public | Level |
| 15 | NPV 99.9% to 96.0% | PPV 60% to 40% | 6.8426 | 1.1531 | 5.93 | 0 | 4.582524 | 9.102676 | General Public | Level |
| 16 | NPV 99.9% to 99.5% | PPV 80% to 60% | 1.4838 | 0.7071 | 2.1 | 0.0179 | 0.097884 | 2.869716 | General Public | Level |
| 17 | NPV 99.9% to 99.0% | PPV 80% to 60% | 2.8336 | 0.9053 | 3.13 | 9.00E-04 | 1.059212 | 4.607988 | General Public | Level |
| 18 | NPV 99.9% to 96.0% | PPV 80% to 60% | 7.3868 | 1.3373 | 5.52 | 0 | 4.765692 | 10.00791 | General Public | Level |
| 19 | test_level_high_largest_difference | -1.7906 | 1.1179 | -1.6 | 0.0548 | -3.98168 | 0.400484 | General Public | Level test |
| 20 | test_level_medium_largest_difference | 0.6869 | 0.4657 | 1.47 | 0.0708 | -0.22587 | 1.599672 | General Public | Level test |
| 21 | test_level_low_largest_difference | 0.3597 | 0.2695 | 1.33 | 0.0918 | -0.16852 | 0.88792 | General Public | Level test |
| 22 | NPV 99.9% to 99.5% | PPV 40% to 20% | 0.5921 | 0.8887 | 0.67 | 0.2514 | -1.14975 | 2.333952 | GPs | Size |
| 23 | NPV 99.9% to 99.0% | PPV 40% to 20% | 1.5948 | 1.2127 | 1.32 | 0.0934 | -0.78209 | 3.971692 | GPs | Size |
| 24 | NPV 99.9% to 96.0% | PPV 40% to 20% | 6.4076 | 1.6679 | 3.84 | 1.00E-04 | 3.138516 | 9.676684 | GPs | Size |
| 25 | NPV 99.9% to 99.5% | PPV 60% to 20% | 0.2587 | 0.3875 | 0.67 | 0.2514 | -0.5008 | 1.0182 | GPs | Size |
| 26 | NPV 99.9% to 99.0% | PPV 60% to 20% | 0.6968 | 0.5248 | 1.33 | 0.0918 | -0.33181 | 1.725408 | GPs | Size |
| 27 | NPV 99.9% to 96.0% | PPV 60% to 20% | 2.7995 | 0.6822 | 4.1 | 0 | 1.462388 | 4.136612 | GPs | Size |
| 28 | NPV 99.9% to 99.5% | PPV 80% to 20% | 0.174 | 0.2609 | 0.67 | 0.2514 | -0.33736 | 0.685364 | GPs | Size |
| 29 | NPV 99.9% to 99.0% | PPV 80% to 20% | 0.4687 | 0.3533 | 1.33 | 0.0918 | -0.22377 | 1.161168 | GPs | Size |
| 30 | NPV 99.9% to 96.0% | PPV 80% to 20% | 1.883 | 0.4572 | 4.12 | 0 | 0.986888 | 2.779112 | GPs | Size |
| 31 | NPV 99.9% to 99.5% | PPV 40% to 20% | 0.5921 | 0.8887 | 0.67 | 0.2514 | -1.14975 | 2.333952 | GPs | Level |
| 32 | NPV 99.9% to 99.0% | PPV 40% to 20% | 1.5948 | 1.2127 | 1.32 | 0.0934 | -0.78209 | 3.971692 | GPs | Level |
| 33 | NPV 99.9% to 96.0% | PPV 40% to 20% | 6.4076 | 1.6679 | 3.84 | 1.00E-04 | 3.138516 | 9.676684 | GPs | Level |
| 34 | NPV 99.9% to 99.5% | PPV 60% to 40% | 0.4594 | 0.6881 | 0.67 | 0.2514 | -0.88928 | 1.808076 | GPs | Level |
| 35 | NPV 99.9% to 99.0% | PPV 60% to 40% | 1.2374 | 0.9305 | 1.33 | 0.0918 | -0.58638 | 3.06118 | GPs | Level |
| 36 | NPV 99.9% to 96.0% | PPV 60% to 40% | 4.9717 | 1.2154 | 4.09 | 0 | 2.589516 | 7.353884 | GPs | Level |
| 37 | NPV 99.9% to 99.5% | PPV 80% to 60% | 0.5315 | 0.7999 | 0.66 | 0.2546 | -1.0363 | 2.099304 | GPs | Level |
| 38 | NPV 99.9% to 99.0% | PPV 80% to 60% | 1.4315 | 1.0905 | 1.31 | 0.0951 | -0.70588 | 3.56888 | GPs | Level |
| 39 | NPV 99.9% to 96.0% | PPV 80% to 60% | 5.7514 | 1.4991 | 3.84 | 1.00E-04 | 2.813164 | 8.689636 | GPs | Level |
| 40 | test_level_high_largest_difference | -1.436 | 0.836 | -1.72 | 0.0427 | -3.07456 | 0.20256 | GPs | Level test |
| 41 | test_level_medium_largest_difference | 0.3574 | 0.3326 | 1.07 | 0.1423 | -0.2945 | 1.009296 | GPs | Level test |
| 42 | test_level_low_largest_difference | 0.1327 | 0.2109 | 0.63 | 0.2643 | -0.28066 | 0.546064 | GPs | Level test |

Size effects: lines 3,6,9 – do bigger changes matter more than smaller changes?

Line 3 shows us the relative value of a shift from 99.9%NPV to 96.0% NPV and a shift of 40% to 20% PPV. The value is 8.6 – so a 4% change in NPV is worth 8.6 times a 20% change in PPV.

Line 6 shows us the relative value of a shift from 99.9%NPV to 96.0% NPV and a shift of 60% to 20% PPV. The value is 3.8 – so a 4% change in NPV is worth 3.8 times a 40% change in PPV.

Line 9 shows us the relative value of a shift from 99.9%NPV to 96.0% NPV and a shift of 80% to 20% PPV. The value is 2.5 – so a 4% change in NPV is worth 2.5 times a 60% change in PPV.

Here, bigger changes in PPV matter more because the relative value of the same amount of NPV decreases. But that amount of NPV change is still more important than a large swing in PPV.

Level effects: lines 12,15,18 – does the same amount of NPV change matter up or down the range of the PPV?

Line 12 shows us the relative value of a shift from 99.9%NPV to 96.0% NPV and a shift of 40% to 20% PPV. The value is 8.6 – so a 4% change in NPV is worth 8.6 times a 20% change in PPV.

Line 15 shows us the relative value of a shift from 99.9%NPV to 96.0% NPV and a shift of 60% to 40% PPV. The value is 6.8 – so a 4% change in NPV is worth 6.8 times a 20% change in PPV.

Line 18 shows us the relative value of a shift from 99.9%NPV to 96.0% NPV and a shift of 80% to 60% PPV. The value is 7.3 – so a 4% change in NPV is worth 7.3 times a 20% change in PPV.

Here, a 20% shift in PPV is of the same magnitude moving up/down the range of PPV (not statistically significantly different). Testing of this in lines 19-21 for the general public and 40-42 for GPs. We find no evidence of level effects.

Supplement 13: Scoping review of DCEs

This supplement summarises the results of a scoping literature review that was undertaken to inform the development of a discrete choice experiment (DCE). The aim of the DCE is to investigate stakeholder preferences for the use of multi-cancer early detection (MCED) tests in symptomatic patients in the UK NHS. The stakeholders in question are general practitioners and members of the public. The purpose of undertaking the literature review was to elicit a list of attributes that could potentially influence the preferences of these stakeholders when deciding whether to use/undergo a MCED test.

**Search Strategy**

The search strategy involved combining terms related to cancer, terms related to the form of testing (e.g. multi-cancer early detection), and terms related to preferences or DCEs. Search terms were identified by reviewing key known papers in the literature. Four databases were searched: PubMed, Embase, Econlit and PsycInfo. Although the earliest mention of either MCED or “multi-cancer early detection” in PubMed is in 2020, articles highlighting the potential of liquid biopsy tests to guide early detection in cancer start to appear around 2015. Given this, the search start date was 1^st^ January 2015. The search end date was 20^th^ June 2022. All searches were restricted to humans and the English language. Detailed search strategies are presented in the appendix for each database.

Articles were included in the review if they considered the preferences, opinions or beliefs of any stakeholders concerning the use of MCED tests. The preferences of patients were considered to be of interest as there is some overlap with the preferences of members of the public.

**Search results**

The number of hits in each database is listed below:

| **Database** | **Date range searched** | **Hits** | **Duplicates removed** | **After reviewing…** | | |
| --- | --- | --- | --- | --- | --- | --- |
|  |  |  |  | **Titles** | **Abstracts** | **Full-text** |
| **PubMed** | 01/01/15 to 20/06/22 | 30 | 28 | 4 | 2 | 2 |
| **Embase** |  | 83 | 57 | 5 | 3 | 2 |
| **Econlit** |  | 2 | 2 | 0 | 0 | 0 |
| **PsycInfo** |  | 0 | 0 | 0 | 0 | 0 |
| **Added from other sources** | - | 1 | 1 | 1 | 1 | 1 |
| **Total** | - | 116 | 88 | 10 | 6 | 5 |

Four papers were identified in the literature search that met the inclusion criteria [1-4]. One was subsequently added that was already known to the researchers [5]. In total, five papers were included in the review. Appendix 2 presents a brief summary of each paper.

All papers were reviewed by JB. Information was extracted from each paper on potential DCE attributes for our study, including broad constructs that could be converted into an attribute. Twenty-two potential attributes were identified in the literature search. The following table categorises and references these attributes. No potential attributes were identified that were important to GPs; all studies only considered the public or patient perspective.

| **Attribute** | **Count** | **Important to which stakeholders** | | | | **Source** | **Notes** |
| --- | --- | --- | --- | --- | --- | --- | --- |
|  |  | **GPs** | **Public** | **Patients** | **Other** |  |  |
| Waiting time for results | 3 |  | Y | YY |  | [1, 3, 5] | From study comparing tissue vs liquid biopsies [3], study in asymptomatic population [5], and HTA of testing for a treatment resistance mutation in lung cancer [1] |
| False positives / probability of overtreatment or overdiagnosis | 2 |  | YY |  |  | [2, 5] | From study in asymptomatic population |
| Greater certainty on likelihood of recurrence (Relton *et al.*) or on test result more generally (Lee *et al.*) | 2 |  |  | YY |  | [3, 4] | From study of endometrial cancer follow-up [4] and study comparing tissue vs liquid biopsies [3] |
| Invasiveness of testing / pain of undergoing test | 2 |  |  | YY |  | [1, 4] | From study of endometrial cancer follow-up [4] and HTA of testing for a treatment resistance mutation in lung cancer [1] |
| Number of cancers tested for / scope of test | 2 |  | YY |  |  | [2, 5] | From study in asymptomatic population |
| Test location | 2 |  | Y | Y |  | [1, 5] | From study in asymptomatic population [5] and HTA of testing for a treatment resistance mutation in lung cancer [1] |
| Chance of cancer diagnosis | 1 |  | Y |  |  | [5] | From study in asymptomatic population |
| Clinician recommendation to undergo testing or not | 1 |  | Y |  |  | [2] | Not an attribute used in the DCE, but suggested to potentially be important by the authors |
| False negatives | 1 |  | Y |  |  | [2] |  |
| Healthcare provider characteristics | 1 |  | Y |  |  | [5] | From study in asymptomatic population |
| Likelihood that the cancer type is unknown | 1 |  | Y |  |  | [2] |  |
| Mortality risk reduction | 1 |  | Y |  |  | [5] | From study in asymptomatic population |
| Need (or not) for multiple tests | 1 |  | Y |  |  | [5] | From study in asymptomatic population |
| Pre-test support / information | 1 |  | Y |  |  | [5] | From study in asymptomatic population |
| Results delivery | 1 |  | Y |  |  | [5] | From study in asymptomatic population |
| Sensitivity | 1 |  | Y |  |  | [5] | From study in asymptomatic population |
| Specificity | 1 |  | Y |  |  | [5] | From study in asymptomatic population |
| Test procedure e.g. blood test versus tissue sample | 1 |  | Y |  |  | [5] | From study in asymptomatic population |
| Test reputation | 1 |  | Y |  |  | [5] | From study in asymptomatic population |
| Travel time | 1 |  | Y |  |  | [5] | From study in asymptomatic population |
| True positives | 1 |  | Y |  |  | [2] |  |
| Type of healthcare provider | 1 |  | Y |  |  | [5] | From study in asymptomatic population |

**Attributes identified via interviews**

To supplement the literature review, JB interviewed three experts (a GP, a clinical specialist, and a patient representative) to identify additional attributes not captured in the review. Six additional attributes were identified:

1. Accuracy of cancer signal origin prediction
2. Individual characteristics (e.g. age, previous own/family experience of cancer, comorbidities)
3. Positive predictive value / negative predictive value
4. Symptoms
5. Test organisation (e.g. invitation sent to individual, or individual responsible for organising own appointment)
6. Test time of day

**Final longlist of attributes**

The following table lists all attributes identified in the literature review and interviews with experts, along with notes containing further information, and the final decision as to which attributes would be included in the shortlist to be presented in the stakeholder interviews (decision made via consultation between JBuch and BDN).

|  | **Attribute** | **Notes** | **Include in shortlist?** |
| --- | --- | --- | --- |
| 1 | Accuracy of cancer signal origin prediction | What if multiple possible tissues of origin are identified? Does accuracy have implications for the number of follow up tests i.e. the complexity of the patient pathway from this point onwards? | Yes |
| 2 | Chance of cancer diagnosis | What about other non-cancer diagnoses identified after further investigations? Patients are not necessarily thinking solely of cancer when they present with symptoms. | No |
| 3 | Clinician recommendation to undergo testing or not | i.e. clinician has recommended a specific way forward (whether this is an MCED test or another pathway), versus clinician is neutral on the next steps | No |
| 4 | False negatives | i.e. test says no cancer when individual has cancer. Risk must be explained in a very simple way, ideally using graphics with a constant denominator. This applies to all risk attributes in this longlist. | Yes |
| 5 | False positives / probability of overtreatment or over-diagnosis | i.e. test says cancer is present when it is not present. | Yes |
| 6 | Greater certainty on likelihood of recurrence or on test result more generally | Related to some of the other attributes but could perhaps be framed in different ways | No |
| 7 | Healthcare provider characteristics | e.g. breadth of knowledge about cancer, known to patient, experience taking samples. This could be the person who orders the test, who takes the sample, or who returns the results. | Yes |
| 8 | Individual characteristics | e.g. age, previous own/family experience of cancer, comorbidities, ethnicity, index of deprivation, previous experience of screening. More likely to be collected as part of DCE survey rather than featuring as DCE attribute. | Yes |
| 9 | Invasiveness of testing / pain of undergoing test | e.g. whether a blood draw or tissues sample is required for the initial test (and if it is a fasting blood test). The invasiveness of any follow-up tests may also be important e.g. endoscopic investigations. | Yes |
| 10 | Likelihood that the cancer type is unknown | - | Yes |
| 11 | Mortality risk reduction | From the overall MCED testing programme | No |
| 12 | Need (or not) for multiple tests | Strong overlap with other attributes in this list. | Excluded from main list. |
| 13 | Number of cancers tested for / scope of test | Could be anything in the range 1-50 cancers. Also important to note which specific cancers are included e.g. test might be more appealing if it identifies cancers that are difficult to diagnose and generally diagnosed late e.g. pancreas, or cancers there are not currently part of screening programmes. Test might be less appealing if it identifies cancers where it is not helpful to pick them up early. | Yes |
| 14 | Positive predictive value / negative predictive value | Ratio of individuals truly diagnosed as positive to all those who had positive test results / ratio of individuals truly diagnosed as negative to all those who had negative test results. | Yes |
| 15 | Pre-test support / information | e.g. counselling provided, further support signposted, extent of information provided. Linked to #7, who the information comes from is also important e.g. GP (more trusted) or National Screening body (greater expertise), and the format for this support e.g. leaflet versus phone contact versus f2f appointment. | Yes |
| 16 | Results delivery | e.g. amount of information provided, how accessible this information is (language used, quantity of information, whether further information is signposted). | Yes |
| 17 | Sensitivity | How often a test correctly generates a positive result for people who have the condition that’s being tested for | Yes |
| 18 | Specificity | Ability of a test to correctly generate a negative result for people who don’t have the condition that’s being tested for | Yes |
| 19 | Symptoms | The symptoms that the presenting individual is exhibiting | Yes |
| 20 | Test location | GP surgery? Community setting? Secondary care e.g. hospital? Logistics of getting to location also important e.g. is there parking, is it on a bus route and near a bus stop, is the location familiar? | No |
| 21 | Test organisation | Invitation sent to individual (how easy is this to change, is the invitation followed up if there is no response?), or individual responsible for organising own appointment. | No |
| 22 | Test procedure | e.g. blood test versus tissue sample, quantity of blood taken. | No |
| 23 | Test reputation | Somewhat subjective e.g. has test received favourable write-ups in the media, is there robust evidence in the scientific literature, has the test been endorsed or used by trusted people (not experts) e.g. by celebrities on social media, or by actors in widely watched tv shows? | No |
| 24 | Test time of day | Does testing only happen during working hours or during evenings and weekends? | No |
| 25 | Travel time | To get to test location, or to the location where test results will be delivered. | No |
| 26 | True positives | Related to other attributes but could be framed in different ways | Yes |
| 27 | Type of healthcare provider | e.g. GP, hospital consultant, nurse. Strong overlap with #7. | No |
| 28 | Waiting time for results | In days/weeks. Target for turnaround time is 1 week but the range to consider in the DCE might be from 1 week to 3 weeks. | Yes |

Decisions

We selected 16 attributes for the final shortlist to be presented in the stakeholder interviews. These attributes were described in detail during the interviews. The other 12 attributes were retained and presented to participants at the end of the interviews, to check if we have missed anything important.

**References**

1. Anonymous. Cell-free circulating tumour DNA blood testing to detect EGFR t790m mutation in people with advanced non-small cell lung cancer: A health technology assessment. *Ontario Health Technology Assessment Series* 2020;**20(5)**:1-176.

2. Gelhorn H, Ross M, Kansal AR, et al. Patient Preferences for Attributes of a Multi-Cancer Early Detection Test: A Discrete Choice Experiment (DCE) Quantitative Pilot Study. *Value in Health* 2022;**25(1 Supplement)**:S4.

3. Lee MJ, Hueniken K, Kuehne N, et al. Cancer Patient-Reported Preferences and Knowledge for Liquid Biopsies and Blood Biomarkers at a Comprehensive Cancer Center. *Cancer Manag Res* 2020;**12**:1163-73.

4. Relton A, Collins A, Guttery DS, et al. Patient acceptability of circulating tumour DNA testing in endometrial cancer follow-up. *Eur J Cancer Care (Engl)* 2021;**30**(4):e13429.

5. Hall R, Medina-Lara A, Hamilton W, et al. Attributes Used for Cancer Screening Discrete Choice Experiments: A Systematic Review. *Patient* 2022;**15**(3):269-85.

**Search strategies**

PubMed

Run on standard PubMed database on 20^th^ June

(cancer*[Title/Abstract] OR neoplasm*[Title/Abstract] OR carcinoma*[Title/Abstract] OR malignan*[Title/Abstract] OR lesion*[Title/Abstract] OR tumor*[Title/Abstract] OR tumour*[Title/Abstract]) AND (“multi-cancer early detection” [Title/Abstract] OR mced[Title/Abstract] OR “earlier detection”[Title/Abstract] OR “liquid biopsy*”[Title/Abstract] OR “multi-cancer screening”[Title/Abstract] OR “circulating tumor DNA”[Title/Abstract] or “circulating tumour DNA”[Title/Abstract] OR ctDNA[Title/Abstract] OR “cell free DNA”[Title/Abstract] OR grail[Title/Abstract] OR galleri[Title/Abstract]) AND (preference*[Title/Abstract] OR DCE[Title/Abstract] OR conjoint*[Title/Abstract] OR best-worst*[Title/Abstract] OR BWS[Title/Abstract] OR discrete choice*[Title/Abstract])

Restricted to 2015-current and English language and human.

Embase

cancer*.mp

neoplasm*.mp

carcinoma*.mp

malignan*.mp

lesion*.mp

tumo?r*.mp

1 or 2 or 3 or 4 or 5 or 6

multi-cancer early detection.mp

mced.mp

earlier detection.mp

liquid biopsy*.mp

multi-cancer screening.mp

circulating tumo?r DNA.mp

ctDNA.mp

cell free DNA.mp

grail.mp

galleri.mp

8 or 9 or 10 or 11 or 12 or 13 or 14 or 15 or 16 or 17

preference*.mp

DCE.mp

conjoint*.mp

best-worst*.mp

BWS.mp

discrete choice*.mp

19 or 20 or 21 or 22 or 23 or 24

7 and 18 and 25

limit 26 to (human and english language and yr="2015 -Current")

Econlit

noft(cancer* OR neoplasm* OR carcinoma* OR malignan* OR lesion* OR tumo?r*) AND noft(multi-cancer early detection OR mced OR earlier detection OR liquid biopsy* OR multi-cancer screening OR circulating tumo?r DNA OR ctDNA OR cell free DNA OR grail OR galleri) AND noft(preference* OR DCE OR conjoint* OR best-worst* OR BWS OR discrete choice*)

Restricted to 2015-current and English language and human.

Psycinfo

Using PsycINFO 1806 to present

cancer*.mp

neoplasm*.mp

carcinoma*.mp

malignan*.mp

lesion*.mp

tumo?r*.mp

1 or 2 or 3 or 4 or 5 or 6

multi-cancer early detection.mp

mced.mp

earlier detection.mp

liquid biopsy*.mp

multi-cancer screening.mp

circulating tumo?r DNA.mp

ctDNA.mp

cell free DNA.mp

grail.mp

galleri.mp

8 or 9 or 10 or 11 or 12 or 13 or 14 or 15 or 16 or 17

preference*.mp

DCE.mp

conjoint*.mp

best-worst*.mp

BWS.mp

discrete choice*.mp

19 or 20 or 21 or 22 or 23 or 24

7 and 18 and 25

limit 26 to (human and english language and yr="2015 -Current")

Supplement 12: Full set of simulations for MCED tests

| lcb | mean | ucb | mced_test_id | sample | product |
| --- | --- | --- | --- | --- | --- |
| 0.121739 | 0.355593 | 0.697001 | pred_wait_1_detect_1_form1_stage_1_cancers_1_posrisk_8_negrisk_40 | patients | hypothetical |
| 0.182846 | 0.418716 | 0.657271 | pred_wait_1_detect_1_form1_stage_1_cancers_5_posrisk_2_negrisk_1 | Patients | hypothetical |
| 0.191994 | 0.441408 | 0.692158 | pred_wait_1_detect_1_form1_stage_1_cancers_10_posrisk_2_negrisk_1 | Patients | hypothetical |
| 0.246904 | 0.52136 | 0.783577 | pred_wait_1_detect_1_form1_stage_1_cancers_25_posrisk_2_negrisk_1 | Patients | hypothetical |
| 0.172999 | 0.39881 | 0.628372 | pred_wait_1_detect_1_form1_stage_1_cancers_1_posrisk_2_negrisk_5 | Patients | hypothetical |
| 0.177665 | 0.414849 | 0.655619 | pred_wait_1_detect_1_form1_stage_1_cancers_5_posrisk_2_negrisk_5 | Patients | hypothetical |
| 0.186554 | 0.437535 | 0.690041 | pred_wait_1_detect_1_form1_stage_1_cancers_10_posrisk_2_negrisk_5 | Patients | hypothetical |
| 0.240509 | 0.518117 | 0.783733 | pred_wait_1_detect_1_form1_stage_1_cancers_25_posrisk_2_negrisk_5 | Patients | hypothetical |
| 0.166163 | 0.394238 | 0.625924 | pred_wait_1_detect_1_form1_stage_1_cancers_1_posrisk_2_negrisk_10 | Patients | hypothetical |
| 0.169755 | 0.410138 | 0.652845 | pred_wait_1_detect_1_form1_stage_1_cancers_5_posrisk_2_negrisk_10 | Patients | hypothetical |
| 0.177252 | 0.432739 | 0.688226 | pred_wait_1_detect_1_form1_stage_1_cancers_10_posrisk_2_negrisk_10 | Patients | hypothetical |
| 0.228034 | 0.513809 | 0.782569 | pred_wait_1_detect_1_form1_stage_1_cancers_25_posrisk_2_negrisk_10 | Patients | hypothetical |
| 0.147697 | 0.375481 | 0.619559 | pred_wait_1_detect_1_form1_stage_1_cancers_1_posrisk_2_negrisk_40 | Patients | hypothetical |
| 0.150532 | 0.39012 | 0.646715 | pred_wait_1_detect_1_form1_stage_1_cancers_5_posrisk_2_negrisk_40 | Patients | hypothetical |
| 0.155941 | 0.411075 | 0.681777 | pred_wait_1_detect_1_form1_stage_1_cancers_10_posrisk_2_negrisk_40 | Patients | hypothetical |
| 0.198626 | 0.488042 | 0.774579 | pred_wait_1_detect_1_form1_stage_1_cancers_25_posrisk_2_negrisk_40 | Patients | hypothetical |
| 0.110715 | 0.343384 | 0.620335 | pred_wait_1_detect_1_form1_stage_1_cancers_1_posrisk_4_negrisk_1 | Patients | hypothetical |
| 0.111862 | 0.356254 | 0.647194 | pred_wait_1_detect_1_form1_stage_1_cancers_5_posrisk_4_negrisk_1 | Patients | hypothetical |
| 0.115167 | 0.374947 | 0.681878 | pred_wait_1_detect_1_form1_stage_1_cancers_10_posrisk_4_negrisk_1 | Patients | hypothetical |
| 0.147273 | 0.446215 | 0.774045 | pred_wait_1_detect_1_form1_stage_1_cancers_25_posrisk_4_negrisk_1 | Patients | hypothetical |
| 0.110715 | 0.33959 | 0.615487 | pred_wait_1_detect_1_form1_stage_1_cancers_1_posrisk_4_negrisk_5 | Patients | hypothetical |
| 0.111862 | 0.352387 | 0.643274 | pred_wait_1_detect_1_form1_stage_1_cancers_5_posrisk_4_negrisk_5 | Patients | hypothetical |
| 0.115167 | 0.371075 | 0.679174 | pred_wait_1_detect_1_form1_stage_1_cancers_10_posrisk_4_negrisk_5 | Patients | hypothetical |
| 0.147273 | 0.442972 | 0.774488 | pred_wait_1_detect_1_form1_stage_1_cancers_25_posrisk_4_negrisk_5 | Patients | hypothetical |
| 0.110715 | 0.335018 | 0.60839 | pred_wait_1_detect_1_form1_stage_1_cancers_1_posrisk_4_negrisk_10 | Patients | hypothetical |
| 0.111862 | 0.347676 | 0.636821 | pred_wait_1_detect_1_form1_stage_1_cancers_5_posrisk_4_negrisk_10 | Patients | hypothetical |
| 0.115167 | 0.366279 | 0.673792 | pred_wait_1_detect_1_form1_stage_1_cancers_10_posrisk_4_negrisk_10 | Patients | hypothetical |
| 0.147273 | 0.438664 | 0.772624 | pred_wait_1_detect_1_form1_stage_1_cancers_25_posrisk_4_negrisk_10 | Patients | hypothetical |
| 0.110618 | 0.316262 | 0.555857 | pred_wait_1_detect_1_form1_stage_1_cancers_1_posrisk_4_negrisk_40 | Patients | hypothetical |
| 0.111767 | 0.327657 | 0.579561 | pred_wait_1_detect_1_form1_stage_1_cancers_5_posrisk_4_negrisk_40 | Patients | hypothetical |
| 0.115153 | 0.344615 | 0.611993 | pred_wait_1_detect_1_form1_stage_1_cancers_10_posrisk_4_negrisk_40 | Patients | hypothetical |
| 0.147273 | 0.412897 | 0.707796 | pred_wait_1_detect_1_form1_stage_1_cancers_25_posrisk_4_negrisk_40 | Patients | hypothetical |
| 0.089891 | 0.31147 | 0.620335 | pred_wait_1_detect_1_form1_stage_1_cancers_1_posrisk_6_negrisk_1 | Patients | hypothetical |
| 0.091778 | 0.322679 | 0.647194 | pred_wait_1_detect_1_form1_stage_1_cancers_5_posrisk_6_negrisk_1 | Patients | hypothetical |
| 0.095358 | 0.338942 | 0.681878 | pred_wait_1_detect_1_form1_stage_1_cancers_10_posrisk_6_negrisk_1 | Patients | hypothetical |
| 0.120116 | 0.40078 | 0.774045 | pred_wait_1_detect_1_form1_stage_1_cancers_25_posrisk_6_negrisk_1 | Patients | hypothetical |
| 0.089891 | 0.307676 | 0.615487 | pred_wait_1_detect_1_form1_stage_1_cancers_1_posrisk_6_negrisk_5 | Patients | hypothetical |
| 0.091778 | 0.318812 | 0.643274 | pred_wait_1_detect_1_form1_stage_1_cancers_5_posrisk_6_negrisk_5 | Patients | hypothetical |
| 0.095358 | 0.335069 | 0.679174 | pred_wait_1_detect_1_form1_stage_1_cancers_10_posrisk_6_negrisk_5 | Patients | hypothetical |
| 0.120116 | 0.397537 | 0.774488 | pred_wait_1_detect_1_form1_stage_1_cancers_25_posrisk_6_negrisk_5 | Patients | hypothetical |
| 0.089891 | 0.303104 | 0.60839 | pred_wait_1_detect_1_form1_stage_1_cancers_1_posrisk_6_negrisk_10 | Patients | hypothetical |
| 0.091778 | 0.314101 | 0.636821 | pred_wait_1_detect_1_form1_stage_1_cancers_5_posrisk_6_negrisk_10 | Patients | hypothetical |
| 0.095358 | 0.330273 | 0.673792 | pred_wait_1_detect_1_form1_stage_1_cancers_10_posrisk_6_negrisk_10 | Patients | hypothetical |
| 0.120116 | 0.393229 | 0.772624 | pred_wait_1_detect_1_form1_stage_1_cancers_25_posrisk_6_negrisk_10 | Patients | hypothetical |
| 0.089891 | 0.284348 | 0.555857 | pred_wait_1_detect_1_form1_stage_1_cancers_1_posrisk_6_negrisk_40 | Patients | hypothetical |
| 0.091778 | 0.294083 | 0.579561 | pred_wait_1_detect_1_form1_stage_1_cancers_5_posrisk_6_negrisk_40 | Patients | hypothetical |
| 0.095358 | 0.308609 | 0.611993 | pred_wait_1_detect_1_form1_stage_1_cancers_10_posrisk_6_negrisk_40 | Patients | hypothetical |
| 0.120116 | 0.367462 | 0.707796 | pred_wait_1_detect_1_form1_stage_1_cancers_25_posrisk_6_negrisk_40 | Patients | hypothetical |
| 0.089238 | 0.297922 | 0.620335 | pred_wait_1_detect_1_form1_stage_1_cancers_1_posrisk_8_negrisk_1 | Patients | hypothetical |
| 0.091829 | 0.308532 | 0.647194 | pred_wait_1_detect_1_form1_stage_1_cancers_5_posrisk_8_negrisk_1 | Patients | hypothetical |
| 0.095875 | 0.323731 | 0.681878 | pred_wait_1_detect_1_form1_stage_1_cancers_10_posrisk_8_negrisk_1 | Patients | hypothetical |
| 0.117168 | 0.379898 | 0.774045 | pred_wait_1_detect_1_form1_stage_1_cancers_25_posrisk_8_negrisk_1 | Patients | hypothetical |
| 0.089238 | 0.294128 | 0.615487 | pred_wait_1_detect_1_form1_stage_1_cancers_1_posrisk_8_negrisk_5 | Patients | hypothetical |
| 0.091829 | 0.304665 | 0.643274 | pred_wait_1_detect_1_form1_stage_1_cancers_5_posrisk_8_negrisk_5 | Patients | hypothetical |
| 0.095875 | 0.319858 | 0.679174 | pred_wait_1_detect_1_form1_stage_1_cancers_10_posrisk_8_negrisk_5 | Patients | hypothetical |
| 0.117168 | 0.376655 | 0.774488 | pred_wait_1_detect_1_form1_stage_1_cancers_25_posrisk_8_negrisk_5 | Patients | hypothetical |
| 0.089238 | 0.289556 | 0.60839 | pred_wait_1_detect_1_form1_stage_1_cancers_1_posrisk_8_negrisk_10 | Patients | hypothetical |
| 0.091829 | 0.299954 | 0.636821 | pred_wait_1_detect_1_form1_stage_1_cancers_5_posrisk_8_negrisk_10 | Patients | hypothetical |
| 0.095875 | 0.315062 | 0.673792 | pred_wait_1_detect_1_form1_stage_1_cancers_10_posrisk_8_negrisk_10 | Patients | hypothetical |
| 0.117168 | 0.372347 | 0.772624 | pred_wait_1_detect_1_form1_stage_1_cancers_25_posrisk_8_negrisk_10 | Patients | hypothetical |
| 0.089238 | 0.2708 | 0.555857 | pred_wait_1_detect_1_form1_stage_1_cancers_1_posrisk_8_negrisk_40 | Patients | hypothetical |
| 0.091829 | 0.279935 | 0.579561 | pred_wait_1_detect_1_form1_stage_1_cancers_5_posrisk_8_negrisk_40 | Patients | hypothetical |
| 0.095875 | 0.293398 | 0.611993 | pred_wait_1_detect_1_form1_stage_1_cancers_10_posrisk_8_negrisk_40 | Patients | hypothetical |
| 0.117168 | 0.34658 | 0.707796 | pred_wait_1_detect_1_form1_stage_1_cancers_25_posrisk_8_negrisk_40 | Patients | hypothetical |
| 0.177685 | 0.402604 | 0.630238 | pred_wait_1_detect_1_form1_stage_2_cancers_1_posrisk_2_negrisk_1 | Patients | hypothetical |
| 0.182846 | 0.418716 | 0.657271 | pred_wait_1_detect_1_form1_stage_2_cancers_5_posrisk_2_negrisk_1 | Patients | hypothetical |
| 0.191994 | 0.441408 | 0.692158 | pred_wait_1_detect_1_form1_stage_2_cancers_10_posrisk_2_negrisk_1 | Patients | hypothetical |
| 0.246904 | 0.52136 | 0.783577 | pred_wait_1_detect_1_form1_stage_2_cancers_25_posrisk_2_negrisk_1 | Patients | hypothetical |
| 0.172999 | 0.39881 | 0.628372 | pred_wait_1_detect_1_form1_stage_2_cancers_1_posrisk_2_negrisk_5 | Patients | hypothetical |
| 0.177665 | 0.414849 | 0.655619 | pred_wait_1_detect_1_form1_stage_2_cancers_5_posrisk_2_negrisk_5 | Patients | hypothetical |
| 0.186554 | 0.437535 | 0.690041 | pred_wait_1_detect_1_form1_stage_2_cancers_10_posrisk_2_negrisk_5 | Patients | hypothetical |
| 0.240509 | 0.518117 | 0.783733 | pred_wait_1_detect_1_form1_stage_2_cancers_25_posrisk_2_negrisk_5 | Patients | hypothetical |
| 0.166163 | 0.394238 | 0.625924 | pred_wait_1_detect_1_form1_stage_2_cancers_1_posrisk_2_negrisk_10 | Patients | hypothetical |
| 0.169755 | 0.410138 | 0.652845 | pred_wait_1_detect_1_form1_stage_2_cancers_5_posrisk_2_negrisk_10 | Patients | hypothetical |
| 0.177252 | 0.432739 | 0.688226 | pred_wait_1_detect_1_form1_stage_2_cancers_10_posrisk_2_negrisk_10 | Patients | hypothetical |
| 0.228034 | 0.513809 | 0.782569 | pred_wait_1_detect_1_form1_stage_2_cancers_25_posrisk_2_negrisk_10 | Patients | hypothetical |
| 0.147697 | 0.375481 | 0.619559 | pred_wait_1_detect_1_form1_stage_2_cancers_1_posrisk_2_negrisk_40 | Patients | hypothetical |
| 0.150532 | 0.39012 | 0.646715 | pred_wait_1_detect_1_form1_stage_2_cancers_5_posrisk_2_negrisk_40 | Patients | hypothetical |
| 0.155941 | 0.411075 | 0.681777 | pred_wait_1_detect_1_form1_stage_2_cancers_10_posrisk_2_negrisk_40 | Patients | hypothetical |
| 0.198626 | 0.488042 | 0.774579 | pred_wait_1_detect_1_form1_stage_2_cancers_25_posrisk_2_negrisk_40 | Patients | hypothetical |
| 0.110715 | 0.343384 | 0.620335 | pred_wait_1_detect_1_form1_stage_2_cancers_1_posrisk_4_negrisk_1 | Patients | hypothetical |
| 0.111862 | 0.356254 | 0.647194 | pred_wait_1_detect_1_form1_stage_2_cancers_5_posrisk_4_negrisk_1 | Patients | hypothetical |
| 0.115167 | 0.374947 | 0.681878 | pred_wait_1_detect_1_form1_stage_2_cancers_10_posrisk_4_negrisk_1 | Patients | hypothetical |
| 0.147273 | 0.446215 | 0.774045 | pred_wait_1_detect_1_form1_stage_2_cancers_25_posrisk_4_negrisk_1 | Patients | hypothetical |
| 0.110715 | 0.33959 | 0.615487 | pred_wait_1_detect_1_form1_stage_2_cancers_1_posrisk_4_negrisk_5 | Patients | hypothetical |
| 0.111862 | 0.352387 | 0.643274 | pred_wait_1_detect_1_form1_stage_2_cancers_5_posrisk_4_negrisk_5 | Patients | hypothetical |
| 0.115167 | 0.371075 | 0.679174 | pred_wait_1_detect_1_form1_stage_2_cancers_10_posrisk_4_negrisk_5 | Patients | hypothetical |
| 0.147273 | 0.442972 | 0.774488 | pred_wait_1_detect_1_form1_stage_2_cancers_25_posrisk_4_negrisk_5 | Patients | hypothetical |
| 0.110715 | 0.335018 | 0.60839 | pred_wait_1_detect_1_form1_stage_2_cancers_1_posrisk_4_negrisk_10 | Patients | hypothetical |
| 0.111862 | 0.347676 | 0.636821 | pred_wait_1_detect_1_form1_stage_2_cancers_5_posrisk_4_negrisk_10 | Patients | hypothetical |
| 0.115167 | 0.366279 | 0.673792 | pred_wait_1_detect_1_form1_stage_2_cancers_10_posrisk_4_negrisk_10 | Patients | hypothetical |
| 0.147273 | 0.438664 | 0.772624 | pred_wait_1_detect_1_form1_stage_2_cancers_25_posrisk_4_negrisk_10 | Patients | hypothetical |
| 0.110618 | 0.316262 | 0.555857 | pred_wait_1_detect_1_form1_stage_2_cancers_1_posrisk_4_negrisk_40 | Patients | hypothetical |
| 0.111767 | 0.327657 | 0.579561 | pred_wait_1_detect_1_form1_stage_2_cancers_5_posrisk_4_negrisk_40 | Patients | hypothetical |
| 0.115153 | 0.344615 | 0.611993 | pred_wait_1_detect_1_form1_stage_2_cancers_10_posrisk_4_negrisk_40 | Patients | hypothetical |
| 0.147273 | 0.412897 | 0.707796 | pred_wait_1_detect_1_form1_stage_2_cancers_25_posrisk_4_negrisk_40 | Patients | hypothetical |
| 0.089891 | 0.31147 | 0.620335 | pred_wait_1_detect_1_form1_stage_2_cancers_1_posrisk_6_negrisk_1 | Patients | hypothetical |
| 0.091778 | 0.322679 | 0.647194 | pred_wait_1_detect_1_form1_stage_2_cancers_5_posrisk_6_negrisk_1 | Patients | hypothetical |
| 0.095358 | 0.338942 | 0.681878 | pred_wait_1_detect_1_form1_stage_2_cancers_10_posrisk_6_negrisk_1 | Patients | hypothetical |
| 0.120116 | 0.40078 | 0.774045 | pred_wait_1_detect_1_form1_stage_2_cancers_25_posrisk_6_negrisk_1 | Patients | hypothetical |
| 0.089891 | 0.307676 | 0.615487 | pred_wait_1_detect_1_form1_stage_2_cancers_1_posrisk_6_negrisk_5 | Patients | hypothetical |
| 0.091778 | 0.318812 | 0.643274 | pred_wait_1_detect_1_form1_stage_2_cancers_5_posrisk_6_negrisk_5 | Patients | hypothetical |
| 0.095358 | 0.335069 | 0.679174 | pred_wait_1_detect_1_form1_stage_2_cancers_10_posrisk_6_negrisk_5 | Patients | hypothetical |
| 0.120116 | 0.397537 | 0.774488 | pred_wait_1_detect_1_form1_stage_2_cancers_25_posrisk_6_negrisk_5 | Patients | hypothetical |
| 0.089891 | 0.303104 | 0.60839 | pred_wait_1_detect_1_form1_stage_2_cancers_1_posrisk_6_negrisk_10 | Patients | hypothetical |
| 0.091778 | 0.314101 | 0.636821 | pred_wait_1_detect_1_form1_stage_2_cancers_5_posrisk_6_negrisk_10 | Patients | hypothetical |
| 0.095358 | 0.330273 | 0.673792 | pred_wait_1_detect_1_form1_stage_2_cancers_10_posrisk_6_negrisk_10 | Patients | hypothetical |
| 0.120116 | 0.393229 | 0.772624 | pred_wait_1_detect_1_form1_stage_2_cancers_25_posrisk_6_negrisk_10 | Patients | hypothetical |
| 0.089891 | 0.284348 | 0.555857 | pred_wait_1_detect_1_form1_stage_2_cancers_1_posrisk_6_negrisk_40 | Patients | hypothetical |
| 0.091778 | 0.294083 | 0.579561 | pred_wait_1_detect_1_form1_stage_2_cancers_5_posrisk_6_negrisk_40 | Patients | hypothetical |
| 0.095358 | 0.308609 | 0.611993 | pred_wait_1_detect_1_form1_stage_2_cancers_10_posrisk_6_negrisk_40 | Patients | hypothetical |
| 0.120116 | 0.367462 | 0.707796 | pred_wait_1_detect_1_form1_stage_2_cancers_25_posrisk_6_negrisk_40 | Patients | hypothetical |
| 0.089238 | 0.297922 | 0.620335 | pred_wait_1_detect_1_form1_stage_2_cancers_1_posrisk_8_negrisk_1 | Patients | hypothetical |
| 0.091829 | 0.308532 | 0.647194 | pred_wait_1_detect_1_form1_stage_2_cancers_5_posrisk_8_negrisk_1 | Patients | hypothetical |
| 0.095875 | 0.323731 | 0.681878 | pred_wait_1_detect_1_form1_stage_2_cancers_10_posrisk_8_negrisk_1 | Patients | hypothetical |
| 0.117168 | 0.379898 | 0.774045 | pred_wait_1_detect_1_form1_stage_2_cancers_25_posrisk_8_negrisk_1 | Patients | hypothetical |
| 0.089238 | 0.294128 | 0.615487 | pred_wait_1_detect_1_form1_stage_2_cancers_1_posrisk_8_negrisk_5 | Patients | hypothetical |
| 0.091829 | 0.304665 | 0.643274 | pred_wait_1_detect_1_form1_stage_2_cancers_5_posrisk_8_negrisk_5 | Patients | hypothetical |
| 0.095875 | 0.319858 | 0.679174 | pred_wait_1_detect_1_form1_stage_2_cancers_10_posrisk_8_negrisk_5 | Patients | hypothetical |
| 0.117168 | 0.376655 | 0.774488 | pred_wait_1_detect_1_form1_stage_2_cancers_25_posrisk_8_negrisk_5 | Patients | hypothetical |
| 0.089238 | 0.289556 | 0.60839 | pred_wait_1_detect_1_form1_stage_2_cancers_1_posrisk_8_negrisk_10 | Patients | hypothetical |
| 0.091829 | 0.299954 | 0.636821 | pred_wait_1_detect_1_form1_stage_2_cancers_5_posrisk_8_negrisk_10 | Patients | hypothetical |
| 0.095875 | 0.315062 | 0.673792 | pred_wait_1_detect_1_form1_stage_2_cancers_10_posrisk_8_negrisk_10 | Patients | hypothetical |
| 0.117168 | 0.372347 | 0.772624 | pred_wait_1_detect_1_form1_stage_2_cancers_25_posrisk_8_negrisk_10 | Patients | hypothetical |
| 0.089238 | 0.2708 | 0.555857 | pred_wait_1_detect_1_form1_stage_2_cancers_1_posrisk_8_negrisk_40 | Patients | hypothetical |
| 0.091829 | 0.279935 | 0.579561 | pred_wait_1_detect_1_form1_stage_2_cancers_5_posrisk_8_negrisk_40 | Patients | hypothetical |
| 0.095875 | 0.293398 | 0.611993 | pred_wait_1_detect_1_form1_stage_2_cancers_10_posrisk_8_negrisk_40 | Patients | hypothetical |
| 0.117168 | 0.34658 | 0.707796 | pred_wait_1_detect_1_form1_stage_2_cancers_25_posrisk_8_negrisk_40 | Patients | hypothetical |
| 0.177685 | 0.402604 | 0.630238 | pred_wait_1_detect_1_form2_stage_1_cancers_1_posrisk_2_negrisk_1 | Patients | hypothetical |
| 0.182846 | 0.418716 | 0.657271 | pred_wait_1_detect_1_form2_stage_1_cancers_5_posrisk_2_negrisk_1 | Patients | hypothetical |
| 0.191994 | 0.441408 | 0.692158 | pred_wait_1_detect_1_form2_stage_1_cancers_10_posrisk_2_negrisk_1 | Patients | hypothetical |
| 0.246904 | 0.52136 | 0.783577 | pred_wait_1_detect_1_form2_stage_1_cancers_25_posrisk_2_negrisk_1 | Patients | hypothetical |
| 0.172999 | 0.39881 | 0.628372 | pred_wait_1_detect_1_form2_stage_1_cancers_1_posrisk_2_negrisk_5 | Patients | hypothetical |
| 0.177665 | 0.414849 | 0.655619 | pred_wait_1_detect_1_form2_stage_1_cancers_5_posrisk_2_negrisk_5 | Patients | hypothetical |
| 0.186554 | 0.437535 | 0.690041 | pred_wait_1_detect_1_form2_stage_1_cancers_10_posrisk_2_negrisk_5 | Patients | hypothetical |
| 0.240509 | 0.518117 | 0.783733 | pred_wait_1_detect_1_form2_stage_1_cancers_25_posrisk_2_negrisk_5 | Patients | hypothetical |
| 0.166163 | 0.394238 | 0.625924 | pred_wait_1_detect_1_form2_stage_1_cancers_1_posrisk_2_negrisk_10 | Patients | hypothetical |
| 0.169755 | 0.410138 | 0.652845 | pred_wait_1_detect_1_form2_stage_1_cancers_5_posrisk_2_negrisk_10 | Patients | hypothetical |
| 0.177252 | 0.432739 | 0.688226 | pred_wait_1_detect_1_form2_stage_1_cancers_10_posrisk_2_negrisk_10 | Patients | hypothetical |
| 0.228034 | 0.513809 | 0.782569 | pred_wait_1_detect_1_form2_stage_1_cancers_25_posrisk_2_negrisk_10 | Patients | hypothetical |
| 0.147697 | 0.375481 | 0.619559 | pred_wait_1_detect_1_form2_stage_1_cancers_1_posrisk_2_negrisk_40 | Patients | hypothetical |
| 0.150532 | 0.39012 | 0.646715 | pred_wait_1_detect_1_form2_stage_1_cancers_5_posrisk_2_negrisk_40 | Patients | hypothetical |
| 0.155941 | 0.411075 | 0.681777 | pred_wait_1_detect_1_form2_stage_1_cancers_10_posrisk_2_negrisk_40 | Patients | hypothetical |
| 0.198626 | 0.488042 | 0.774579 | pred_wait_1_detect_1_form2_stage_1_cancers_25_posrisk_2_negrisk_40 | Patients | hypothetical |
| 0.110715 | 0.343384 | 0.620335 | pred_wait_1_detect_1_form2_stage_1_cancers_1_posrisk_4_negrisk_1 | Patients | hypothetical |
| 0.111862 | 0.356254 | 0.647194 | pred_wait_1_detect_1_form2_stage_1_cancers_5_posrisk_4_negrisk_1 | Patients | hypothetical |
| 0.115167 | 0.374947 | 0.681878 | pred_wait_1_detect_1_form2_stage_1_cancers_10_posrisk_4_negrisk_1 | Patients | hypothetical |
| 0.147273 | 0.446215 | 0.774045 | pred_wait_1_detect_1_form2_stage_1_cancers_25_posrisk_4_negrisk_1 | Patients | hypothetical |
| 0.110715 | 0.33959 | 0.615487 | pred_wait_1_detect_1_form2_stage_1_cancers_1_posrisk_4_negrisk_5 | Patients | hypothetical |
| 0.111862 | 0.352387 | 0.643274 | pred_wait_1_detect_1_form2_stage_1_cancers_5_posrisk_4_negrisk_5 | Patients | hypothetical |
| 0.115167 | 0.371075 | 0.679174 | pred_wait_1_detect_1_form2_stage_1_cancers_10_posrisk_4_negrisk_5 | Patients | hypothetical |
| 0.147273 | 0.442972 | 0.774488 | pred_wait_1_detect_1_form2_stage_1_cancers_25_posrisk_4_negrisk_5 | Patients | hypothetical |
| 0.110715 | 0.335018 | 0.60839 | pred_wait_1_detect_1_form2_stage_1_cancers_1_posrisk_4_negrisk_10 | Patients | hypothetical |
| 0.111862 | 0.347676 | 0.636821 | pred_wait_1_detect_1_form2_stage_1_cancers_5_posrisk_4_negrisk_10 | Patients | hypothetical |
| 0.115167 | 0.366279 | 0.673792 | pred_wait_1_detect_1_form2_stage_1_cancers_10_posrisk_4_negrisk_10 | Patients | hypothetical |
| 0.147273 | 0.438664 | 0.772624 | pred_wait_1_detect_1_form2_stage_1_cancers_25_posrisk_4_negrisk_10 | Patients | hypothetical |
| 0.110618 | 0.316262 | 0.555857 | pred_wait_1_detect_1_form2_stage_1_cancers_1_posrisk_4_negrisk_40 | Patients | hypothetical |
| 0.111767 | 0.327657 | 0.579561 | pred_wait_1_detect_1_form2_stage_1_cancers_5_posrisk_4_negrisk_40 | Patients | hypothetical |
| 0.115153 | 0.344615 | 0.611993 | pred_wait_1_detect_1_form2_stage_1_cancers_10_posrisk_4_negrisk_40 | Patients | hypothetical |
| 0.147273 | 0.412897 | 0.707796 | pred_wait_1_detect_1_form2_stage_1_cancers_25_posrisk_4_negrisk_40 | Patients | hypothetical |
| 0.089891 | 0.31147 | 0.620335 | pred_wait_1_detect_1_form2_stage_1_cancers_1_posrisk_6_negrisk_1 | Patients | hypothetical |
| 0.091778 | 0.322679 | 0.647194 | pred_wait_1_detect_1_form2_stage_1_cancers_5_posrisk_6_negrisk_1 | Patients | hypothetical |
| 0.095358 | 0.338942 | 0.681878 | pred_wait_1_detect_1_form2_stage_1_cancers_10_posrisk_6_negrisk_1 | Patients | hypothetical |
| 0.120116 | 0.40078 | 0.774045 | pred_wait_1_detect_1_form2_stage_1_cancers_25_posrisk_6_negrisk_1 | Patients | hypothetical |
| 0.089891 | 0.307676 | 0.615487 | pred_wait_1_detect_1_form2_stage_1_cancers_1_posrisk_6_negrisk_5 | Patients | hypothetical |
| 0.091778 | 0.318812 | 0.643274 | pred_wait_1_detect_1_form2_stage_1_cancers_5_posrisk_6_negrisk_5 | Patients | hypothetical |
| 0.095358 | 0.335069 | 0.679174 | pred_wait_1_detect_1_form2_stage_1_cancers_10_posrisk_6_negrisk_5 | Patients | hypothetical |
| 0.120116 | 0.397537 | 0.774488 | pred_wait_1_detect_1_form2_stage_1_cancers_25_posrisk_6_negrisk_5 | Patients | hypothetical |
| 0.089891 | 0.303104 | 0.60839 | pred_wait_1_detect_1_form2_stage_1_cancers_1_posrisk_6_negrisk_10 | Patients | hypothetical |
| 0.091778 | 0.314101 | 0.636821 | pred_wait_1_detect_1_form2_stage_1_cancers_5_posrisk_6_negrisk_10 | Patients | hypothetical |
| 0.095358 | 0.330273 | 0.673792 | pred_wait_1_detect_1_form2_stage_1_cancers_10_posrisk_6_negrisk_10 | Patients | hypothetical |
| 0.120116 | 0.393229 | 0.772624 | pred_wait_1_detect_1_form2_stage_1_cancers_25_posrisk_6_negrisk_10 | Patients | hypothetical |
| 0.089891 | 0.284348 | 0.555857 | pred_wait_1_detect_1_form2_stage_1_cancers_1_posrisk_6_negrisk_40 | Patients | hypothetical |
| 0.091778 | 0.294083 | 0.579561 | pred_wait_1_detect_1_form2_stage_1_cancers_5_posrisk_6_negrisk_40 | Patients | hypothetical |
| 0.095358 | 0.308609 | 0.611993 | pred_wait_1_detect_1_form2_stage_1_cancers_10_posrisk_6_negrisk_40 | Patients | hypothetical |
| 0.120116 | 0.367462 | 0.707796 | pred_wait_1_detect_1_form2_stage_1_cancers_25_posrisk_6_negrisk_40 | Patients | hypothetical |
| 0.089238 | 0.297922 | 0.620335 | pred_wait_1_detect_1_form2_stage_1_cancers_1_posrisk_8_negrisk_1 | Patients | hypothetical |
| 0.091829 | 0.308532 | 0.647194 | pred_wait_1_detect_1_form2_stage_1_cancers_5_posrisk_8_negrisk_1 | Patients | hypothetical |
| 0.095875 | 0.323731 | 0.681878 | pred_wait_1_detect_1_form2_stage_1_cancers_10_posrisk_8_negrisk_1 | Patients | hypothetical |
| 0.117168 | 0.379898 | 0.774045 | pred_wait_1_detect_1_form2_stage_1_cancers_25_posrisk_8_negrisk_1 | Patients | hypothetical |
| 0.089238 | 0.294128 | 0.615487 | pred_wait_1_detect_1_form2_stage_1_cancers_1_posrisk_8_negrisk_5 | Patients | hypothetical |
| 0.091829 | 0.304665 | 0.643274 | pred_wait_1_detect_1_form2_stage_1_cancers_5_posrisk_8_negrisk_5 | Patients | hypothetical |
| 0.095875 | 0.319858 | 0.679174 | pred_wait_1_detect_1_form2_stage_1_cancers_10_posrisk_8_negrisk_5 | Patients | hypothetical |
| 0.117168 | 0.376655 | 0.774488 | pred_wait_1_detect_1_form2_stage_1_cancers_25_posrisk_8_negrisk_5 | Patients | hypothetical |
| 0.089238 | 0.289556 | 0.60839 | pred_wait_1_detect_1_form2_stage_1_cancers_1_posrisk_8_negrisk_10 | Patients | hypothetical |
| 0.091829 | 0.299954 | 0.636821 | pred_wait_1_detect_1_form2_stage_1_cancers_5_posrisk_8_negrisk_10 | Patients | hypothetical |
| 0.095875 | 0.315062 | 0.673792 | pred_wait_1_detect_1_form2_stage_1_cancers_10_posrisk_8_negrisk_10 | Patients | hypothetical |
| 0.117168 | 0.372347 | 0.772624 | pred_wait_1_detect_1_form2_stage_1_cancers_25_posrisk_8_negrisk_10 | Patients | hypothetical |
| 0.089238 | 0.2708 | 0.555857 | pred_wait_1_detect_1_form2_stage_1_cancers_1_posrisk_8_negrisk_40 | Patients | hypothetical |
| 0.091829 | 0.279935 | 0.579561 | pred_wait_1_detect_1_form2_stage_1_cancers_5_posrisk_8_negrisk_40 | Patients | hypothetical |
| 0.095875 | 0.293398 | 0.611993 | pred_wait_1_detect_1_form2_stage_1_cancers_10_posrisk_8_negrisk_40 | Patients | hypothetical |
| 0.117168 | 0.34658 | 0.707796 | pred_wait_1_detect_1_form2_stage_1_cancers_25_posrisk_8_negrisk_40 | Patients | hypothetical |
| 0.177685 | 0.402604 | 0.630238 | pred_wait_1_detect_1_form2_stage_2_cancers_1_posrisk_2_negrisk_1 | Patients | hypothetical |
| 0.182846 | 0.418716 | 0.657271 | pred_wait_1_detect_1_form2_stage_2_cancers_5_posrisk_2_negrisk_1 | Patients | hypothetical |
| 0.191994 | 0.441408 | 0.692158 | pred_wait_1_detect_1_form2_stage_2_cancers_10_posrisk_2_negrisk_1 | Patients | hypothetical |
| 0.246904 | 0.52136 | 0.783577 | pred_wait_1_detect_1_form2_stage_2_cancers_25_posrisk_2_negrisk_1 | Patients | hypothetical |
| 0.172999 | 0.39881 | 0.628372 | pred_wait_1_detect_1_form2_stage_2_cancers_1_posrisk_2_negrisk_5 | Patients | hypothetical |
| 0.177665 | 0.414849 | 0.655619 | pred_wait_1_detect_1_form2_stage_2_cancers_5_posrisk_2_negrisk_5 | Patients | hypothetical |
| 0.186554 | 0.437535 | 0.690041 | pred_wait_1_detect_1_form2_stage_2_cancers_10_posrisk_2_negrisk_5 | Patients | hypothetical |
| 0.240509 | 0.518117 | 0.783733 | pred_wait_1_detect_1_form2_stage_2_cancers_25_posrisk_2_negrisk_5 | Patients | hypothetical |
| 0.166163 | 0.394238 | 0.625924 | pred_wait_1_detect_1_form2_stage_2_cancers_1_posrisk_2_negrisk_10 | Patients | hypothetical |
| 0.169755 | 0.410138 | 0.652845 | pred_wait_1_detect_1_form2_stage_2_cancers_5_posrisk_2_negrisk_10 | Patients | hypothetical |
| 0.177252 | 0.432739 | 0.688226 | pred_wait_1_detect_1_form2_stage_2_cancers_10_posrisk_2_negrisk_10 | Patients | hypothetical |
| 0.228034 | 0.513809 | 0.782569 | pred_wait_1_detect_1_form2_stage_2_cancers_25_posrisk_2_negrisk_10 | Patients | hypothetical |
| 0.147697 | 0.375481 | 0.619559 | pred_wait_1_detect_1_form2_stage_2_cancers_1_posrisk_2_negrisk_40 | Patients | hypothetical |
| 0.150532 | 0.39012 | 0.646715 | pred_wait_1_detect_1_form2_stage_2_cancers_5_posrisk_2_negrisk_40 | Patients | hypothetical |
| 0.155941 | 0.411075 | 0.681777 | pred_wait_1_detect_1_form2_stage_2_cancers_10_posrisk_2_negrisk_40 | Patients | hypothetical |
| 0.198626 | 0.488042 | 0.774579 | pred_wait_1_detect_1_form2_stage_2_cancers_25_posrisk_2_negrisk_40 | Patients | hypothetical |
| 0.110715 | 0.343384 | 0.620335 | pred_wait_1_detect_1_form2_stage_2_cancers_1_posrisk_4_negrisk_1 | Patients | hypothetical |
| 0.111862 | 0.356254 | 0.647194 | pred_wait_1_detect_1_form2_stage_2_cancers_5_posrisk_4_negrisk_1 | Patients | hypothetical |
| 0.115167 | 0.374947 | 0.681878 | pred_wait_1_detect_1_form2_stage_2_cancers_10_posrisk_4_negrisk_1 | Patients | hypothetical |
| 0.147273 | 0.446215 | 0.774045 | pred_wait_1_detect_1_form2_stage_2_cancers_25_posrisk_4_negrisk_1 | Patients | hypothetical |
| 0.110715 | 0.33959 | 0.615487 | pred_wait_1_detect_1_form2_stage_2_cancers_1_posrisk_4_negrisk_5 | Patients | hypothetical |
| 0.111862 | 0.352387 | 0.643274 | pred_wait_1_detect_1_form2_stage_2_cancers_5_posrisk_4_negrisk_5 | Patients | hypothetical |
| 0.115167 | 0.371075 | 0.679174 | pred_wait_1_detect_1_form2_stage_2_cancers_10_posrisk_4_negrisk_5 | Patients | hypothetical |
| 0.147273 | 0.442972 | 0.774488 | pred_wait_1_detect_1_form2_stage_2_cancers_25_posrisk_4_negrisk_5 | Patients | hypothetical |
| 0.110715 | 0.335018 | 0.60839 | pred_wait_1_detect_1_form2_stage_2_cancers_1_posrisk_4_negrisk_10 | Patients | hypothetical |
| 0.111862 | 0.347676 | 0.636821 | pred_wait_1_detect_1_form2_stage_2_cancers_5_posrisk_4_negrisk_10 | Patients | hypothetical |
| 0.115167 | 0.366279 | 0.673792 | pred_wait_1_detect_1_form2_stage_2_cancers_10_posrisk_4_negrisk_10 | Patients | hypothetical |
| 0.147273 | 0.438664 | 0.772624 | pred_wait_1_detect_1_form2_stage_2_cancers_25_posrisk_4_negrisk_10 | Patients | hypothetical |
| 0.110618 | 0.316262 | 0.555857 | pred_wait_1_detect_1_form2_stage_2_cancers_1_posrisk_4_negrisk_40 | Patients | hypothetical |
| 0.111767 | 0.327657 | 0.579561 | pred_wait_1_detect_1_form2_stage_2_cancers_5_posrisk_4_negrisk_40 | Patients | hypothetical |
| 0.115153 | 0.344615 | 0.611993 | pred_wait_1_detect_1_form2_stage_2_cancers_10_posrisk_4_negrisk_40 | Patients | hypothetical |
| 0.147273 | 0.412897 | 0.707796 | pred_wait_1_detect_1_form2_stage_2_cancers_25_posrisk_4_negrisk_40 | Patients | hypothetical |
| 0.089891 | 0.31147 | 0.620335 | pred_wait_1_detect_1_form2_stage_2_cancers_1_posrisk_6_negrisk_1 | Patients | hypothetical |
| 0.091778 | 0.322679 | 0.647194 | pred_wait_1_detect_1_form2_stage_2_cancers_5_posrisk_6_negrisk_1 | Patients | hypothetical |
| 0.095358 | 0.338942 | 0.681878 | pred_wait_1_detect_1_form2_stage_2_cancers_10_posrisk_6_negrisk_1 | Patients | hypothetical |
| 0.120116 | 0.40078 | 0.774045 | pred_wait_1_detect_1_form2_stage_2_cancers_25_posrisk_6_negrisk_1 | Patients | hypothetical |
| 0.089891 | 0.307676 | 0.615487 | pred_wait_1_detect_1_form2_stage_2_cancers_1_posrisk_6_negrisk_5 | Patients | hypothetical |
| 0.091778 | 0.318812 | 0.643274 | pred_wait_1_detect_1_form2_stage_2_cancers_5_posrisk_6_negrisk_5 | Patients | hypothetical |
| 0.095358 | 0.335069 | 0.679174 | pred_wait_1_detect_1_form2_stage_2_cancers_10_posrisk_6_negrisk_5 | Patients | hypothetical |
| 0.120116 | 0.397537 | 0.774488 | pred_wait_1_detect_1_form2_stage_2_cancers_25_posrisk_6_negrisk_5 | Patients | hypothetical |
| 0.089891 | 0.303104 | 0.60839 | pred_wait_1_detect_1_form2_stage_2_cancers_1_posrisk_6_negrisk_10 | Patients | hypothetical |
| 0.091778 | 0.314101 | 0.636821 | pred_wait_1_detect_1_form2_stage_2_cancers_5_posrisk_6_negrisk_10 | Patients | hypothetical |
| 0.095358 | 0.330273 | 0.673792 | pred_wait_1_detect_1_form2_stage_2_cancers_10_posrisk_6_negrisk_10 | Patients | hypothetical |
| 0.120116 | 0.393229 | 0.772624 | pred_wait_1_detect_1_form2_stage_2_cancers_25_posrisk_6_negrisk_10 | Patients | hypothetical |
| 0.089891 | 0.284348 | 0.555857 | pred_wait_1_detect_1_form2_stage_2_cancers_1_posrisk_6_negrisk_40 | Patients | hypothetical |
| 0.091778 | 0.294083 | 0.579561 | pred_wait_1_detect_1_form2_stage_2_cancers_5_posrisk_6_negrisk_40 | Patients | hypothetical |
| 0.095358 | 0.308609 | 0.611993 | pred_wait_1_detect_1_form2_stage_2_cancers_10_posrisk_6_negrisk_40 | Patients | hypothetical |
| 0.120116 | 0.367462 | 0.707796 | pred_wait_1_detect_1_form2_stage_2_cancers_25_posrisk_6_negrisk_40 | Patients | hypothetical |
| 0.089238 | 0.297922 | 0.620335 | pred_wait_1_detect_1_form2_stage_2_cancers_1_posrisk_8_negrisk_1 | Patients | hypothetical |
| 0.091829 | 0.308532 | 0.647194 | pred_wait_1_detect_1_form2_stage_2_cancers_5_posrisk_8_negrisk_1 | Patients | hypothetical |
| 0.095875 | 0.323731 | 0.681878 | pred_wait_1_detect_1_form2_stage_2_cancers_10_posrisk_8_negrisk_1 | Patients | hypothetical |
| 0.117168 | 0.379898 | 0.774045 | pred_wait_1_detect_1_form2_stage_2_cancers_25_posrisk_8_negrisk_1 | Patients | hypothetical |
| 0.089238 | 0.294128 | 0.615487 | pred_wait_1_detect_1_form2_stage_2_cancers_1_posrisk_8_negrisk_5 | Patients | hypothetical |
| 0.091829 | 0.304665 | 0.643274 | pred_wait_1_detect_1_form2_stage_2_cancers_5_posrisk_8_negrisk_5 | Patients | hypothetical |
| 0.095875 | 0.319858 | 0.679174 | pred_wait_1_detect_1_form2_stage_2_cancers_10_posrisk_8_negrisk_5 | Patients | hypothetical |
| 0.117168 | 0.376655 | 0.774488 | pred_wait_1_detect_1_form2_stage_2_cancers_25_posrisk_8_negrisk_5 | Patients | hypothetical |
| 0.089238 | 0.289556 | 0.60839 | pred_wait_1_detect_1_form2_stage_2_cancers_1_posrisk_8_negrisk_10 | Patients | hypothetical |
| 0.091829 | 0.299954 | 0.636821 | pred_wait_1_detect_1_form2_stage_2_cancers_5_posrisk_8_negrisk_10 | Patients | hypothetical |
| 0.095875 | 0.315062 | 0.673792 | pred_wait_1_detect_1_form2_stage_2_cancers_10_posrisk_8_negrisk_10 | Patients | hypothetical |
| 0.117168 | 0.372347 | 0.772624 | pred_wait_1_detect_1_form2_stage_2_cancers_25_posrisk_8_negrisk_10 | Patients | hypothetical |
| 0.089238 | 0.2708 | 0.555857 | pred_wait_1_detect_1_form2_stage_2_cancers_1_posrisk_8_negrisk_40 | Patients | hypothetical |
| 0.091829 | 0.279935 | 0.579561 | pred_wait_1_detect_1_form2_stage_2_cancers_5_posrisk_8_negrisk_40 | Patients | hypothetical |
| 0.095875 | 0.293398 | 0.611993 | pred_wait_1_detect_1_form2_stage_2_cancers_10_posrisk_8_negrisk_40 | Patients | hypothetical |
| 0.117168 | 0.34658 | 0.707796 | pred_wait_1_detect_1_form2_stage_2_cancers_25_posrisk_8_negrisk_40 | Patients | hypothetical |
| 0.177685 | 0.402604 | 0.630238 | pred_wait_1_detect_1_form3_stage_1_cancers_1_posrisk_2_negrisk_1 | Patients | hypothetical |
| 0.182846 | 0.418716 | 0.657271 | pred_wait_1_detect_1_form3_stage_1_cancers_5_posrisk_2_negrisk_1 | Patients | hypothetical |
| 0.191994 | 0.441408 | 0.692158 | pred_wait_1_detect_1_form3_stage_1_cancers_10_posrisk_2_negrisk_1 | Patients | hypothetical |
| 0.246904 | 0.52136 | 0.783577 | pred_wait_1_detect_1_form3_stage_1_cancers_25_posrisk_2_negrisk_1 | Patients | hypothetical |
| 0.172999 | 0.39881 | 0.628372 | pred_wait_1_detect_1_form3_stage_1_cancers_1_posrisk_2_negrisk_5 | Patients | hypothetical |
| 0.177665 | 0.414849 | 0.655619 | pred_wait_1_detect_1_form3_stage_1_cancers_5_posrisk_2_negrisk_5 | Patients | hypothetical |
| 0.186554 | 0.437535 | 0.690041 | pred_wait_1_detect_1_form3_stage_1_cancers_10_posrisk_2_negrisk_5 | Patients | hypothetical |
| 0.240509 | 0.518117 | 0.783733 | pred_wait_1_detect_1_form3_stage_1_cancers_25_posrisk_2_negrisk_5 | Patients | hypothetical |
| 0.166163 | 0.394238 | 0.625924 | pred_wait_1_detect_1_form3_stage_1_cancers_1_posrisk_2_negrisk_10 | Patients | hypothetical |
| 0.169755 | 0.410138 | 0.652845 | pred_wait_1_detect_1_form3_stage_1_cancers_5_posrisk_2_negrisk_10 | Patients | hypothetical |
| 0.177252 | 0.432739 | 0.688226 | pred_wait_1_detect_1_form3_stage_1_cancers_10_posrisk_2_negrisk_10 | Patients | hypothetical |
| 0.228034 | 0.513809 | 0.782569 | pred_wait_1_detect_1_form3_stage_1_cancers_25_posrisk_2_negrisk_10 | Patients | hypothetical |
| 0.147697 | 0.375481 | 0.619559 | pred_wait_1_detect_1_form3_stage_1_cancers_1_posrisk_2_negrisk_40 | Patients | hypothetical |
| 0.150532 | 0.39012 | 0.646715 | pred_wait_1_detect_1_form3_stage_1_cancers_5_posrisk_2_negrisk_40 | Patients | hypothetical |
| 0.155941 | 0.411075 | 0.681777 | pred_wait_1_detect_1_form3_stage_1_cancers_10_posrisk_2_negrisk_40 | Patients | hypothetical |
| 0.198626 | 0.488042 | 0.774579 | pred_wait_1_detect_1_form3_stage_1_cancers_25_posrisk_2_negrisk_40 | Patients | hypothetical |
| 0.110715 | 0.343384 | 0.620335 | pred_wait_1_detect_1_form3_stage_1_cancers_1_posrisk_4_negrisk_1 | Patients | hypothetical |
| 0.111862 | 0.356254 | 0.647194 | pred_wait_1_detect_1_form3_stage_1_cancers_5_posrisk_4_negrisk_1 | Patients | hypothetical |
| 0.115167 | 0.374947 | 0.681878 | pred_wait_1_detect_1_form3_stage_1_cancers_10_posrisk_4_negrisk_1 | Patients | hypothetical |
| 0.147273 | 0.446215 | 0.774045 | pred_wait_1_detect_1_form3_stage_1_cancers_25_posrisk_4_negrisk_1 | Patients | hypothetical |
| 0.110715 | 0.33959 | 0.615487 | pred_wait_1_detect_1_form3_stage_1_cancers_1_posrisk_4_negrisk_5 | Patients | hypothetical |
| 0.111862 | 0.352387 | 0.643274 | pred_wait_1_detect_1_form3_stage_1_cancers_5_posrisk_4_negrisk_5 | Patients | hypothetical |
| 0.115167 | 0.371075 | 0.679174 | pred_wait_1_detect_1_form3_stage_1_cancers_10_posrisk_4_negrisk_5 | Patients | hypothetical |
| 0.147273 | 0.442972 | 0.774488 | pred_wait_1_detect_1_form3_stage_1_cancers_25_posrisk_4_negrisk_5 | Patients | hypothetical |
| 0.110715 | 0.335018 | 0.60839 | pred_wait_1_detect_1_form3_stage_1_cancers_1_posrisk_4_negrisk_10 | Patients | hypothetical |
| 0.111862 | 0.347676 | 0.636821 | pred_wait_1_detect_1_form3_stage_1_cancers_5_posrisk_4_negrisk_10 | Patients | hypothetical |
| 0.115167 | 0.366279 | 0.673792 | pred_wait_1_detect_1_form3_stage_1_cancers_10_posrisk_4_negrisk_10 | Patients | hypothetical |
| 0.147273 | 0.438664 | 0.772624 | pred_wait_1_detect_1_form3_stage_1_cancers_25_posrisk_4_negrisk_10 | Patients | hypothetical |
| 0.110618 | 0.316262 | 0.555857 | pred_wait_1_detect_1_form3_stage_1_cancers_1_posrisk_4_negrisk_40 | Patients | hypothetical |
| 0.111767 | 0.327657 | 0.579561 | pred_wait_1_detect_1_form3_stage_1_cancers_5_posrisk_4_negrisk_40 | Patients | hypothetical |
| 0.115153 | 0.344615 | 0.611993 | pred_wait_1_detect_1_form3_stage_1_cancers_10_posrisk_4_negrisk_40 | Patients | hypothetical |
| 0.147273 | 0.412897 | 0.707796 | pred_wait_1_detect_1_form3_stage_1_cancers_25_posrisk_4_negrisk_40 | Patients | hypothetical |
| 0.089891 | 0.31147 | 0.620335 | pred_wait_1_detect_1_form3_stage_1_cancers_1_posrisk_6_negrisk_1 | Patients | hypothetical |
| 0.091778 | 0.322679 | 0.647194 | pred_wait_1_detect_1_form3_stage_1_cancers_5_posrisk_6_negrisk_1 | Patients | hypothetical |
| 0.095358 | 0.338942 | 0.681878 | pred_wait_1_detect_1_form3_stage_1_cancers_10_posrisk_6_negrisk_1 | Patients | hypothetical |
| 0.120116 | 0.40078 | 0.774045 | pred_wait_1_detect_1_form3_stage_1_cancers_25_posrisk_6_negrisk_1 | Patients | hypothetical |
| 0.089891 | 0.307676 | 0.615487 | pred_wait_1_detect_1_form3_stage_1_cancers_1_posrisk_6_negrisk_5 | Patients | hypothetical |
| 0.091778 | 0.318812 | 0.643274 | pred_wait_1_detect_1_form3_stage_1_cancers_5_posrisk_6_negrisk_5 | Patients | hypothetical |
| 0.095358 | 0.335069 | 0.679174 | pred_wait_1_detect_1_form3_stage_1_cancers_10_posrisk_6_negrisk_5 | Patients | hypothetical |
| 0.120116 | 0.397537 | 0.774488 | pred_wait_1_detect_1_form3_stage_1_cancers_25_posrisk_6_negrisk_5 | Patients | hypothetical |
| 0.089891 | 0.303104 | 0.60839 | pred_wait_1_detect_1_form3_stage_1_cancers_1_posrisk_6_negrisk_10 | Patients | hypothetical |
| 0.091778 | 0.314101 | 0.636821 | pred_wait_1_detect_1_form3_stage_1_cancers_5_posrisk_6_negrisk_10 | Patients | hypothetical |
| 0.095358 | 0.330273 | 0.673792 | pred_wait_1_detect_1_form3_stage_1_cancers_10_posrisk_6_negrisk_10 | Patients | hypothetical |
| 0.120116 | 0.393229 | 0.772624 | pred_wait_1_detect_1_form3_stage_1_cancers_25_posrisk_6_negrisk_10 | Patients | hypothetical |
| 0.089891 | 0.284348 | 0.555857 | pred_wait_1_detect_1_form3_stage_1_cancers_1_posrisk_6_negrisk_40 | Patients | hypothetical |
| 0.091778 | 0.294083 | 0.579561 | pred_wait_1_detect_1_form3_stage_1_cancers_5_posrisk_6_negrisk_40 | Patients | hypothetical |
| 0.095358 | 0.308609 | 0.611993 | pred_wait_1_detect_1_form3_stage_1_cancers_10_posrisk_6_negrisk_40 | Patients | hypothetical |
| 0.120116 | 0.367462 | 0.707796 | pred_wait_1_detect_1_form3_stage_1_cancers_25_posrisk_6_negrisk_40 | Patients | hypothetical |
| 0.089238 | 0.297922 | 0.620335 | pred_wait_1_detect_1_form3_stage_1_cancers_1_posrisk_8_negrisk_1 | Patients | hypothetical |
| 0.091829 | 0.308532 | 0.647194 | pred_wait_1_detect_1_form3_stage_1_cancers_5_posrisk_8_negrisk_1 | Patients | hypothetical |
| 0.095875 | 0.323731 | 0.681878 | pred_wait_1_detect_1_form3_stage_1_cancers_10_posrisk_8_negrisk_1 | Patients | hypothetical |
| 0.117168 | 0.379898 | 0.774045 | pred_wait_1_detect_1_form3_stage_1_cancers_25_posrisk_8_negrisk_1 | Patients | hypothetical |
| 0.089238 | 0.294128 | 0.615487 | pred_wait_1_detect_1_form3_stage_1_cancers_1_posrisk_8_negrisk_5 | Patients | hypothetical |
| 0.091829 | 0.304665 | 0.643274 | pred_wait_1_detect_1_form3_stage_1_cancers_5_posrisk_8_negrisk_5 | Patients | hypothetical |
| 0.095875 | 0.319858 | 0.679174 | pred_wait_1_detect_1_form3_stage_1_cancers_10_posrisk_8_negrisk_5 | Patients | hypothetical |
| 0.117168 | 0.376655 | 0.774488 | pred_wait_1_detect_1_form3_stage_1_cancers_25_posrisk_8_negrisk_5 | Patients | hypothetical |
| 0.089238 | 0.289556 | 0.60839 | pred_wait_1_detect_1_form3_stage_1_cancers_1_posrisk_8_negrisk_10 | Patients | hypothetical |
| 0.091829 | 0.299954 | 0.636821 | pred_wait_1_detect_1_form3_stage_1_cancers_5_posrisk_8_negrisk_10 | Patients | hypothetical |
| 0.095875 | 0.315062 | 0.673792 | pred_wait_1_detect_1_form3_stage_1_cancers_10_posrisk_8_negrisk_10 | Patients | hypothetical |
| 0.117168 | 0.372347 | 0.772624 | pred_wait_1_detect_1_form3_stage_1_cancers_25_posrisk_8_negrisk_10 | Patients | hypothetical |
| 0.089238 | 0.2708 | 0.555857 | pred_wait_1_detect_1_form3_stage_1_cancers_1_posrisk_8_negrisk_40 | Patients | hypothetical |
| 0.091829 | 0.279935 | 0.579561 | pred_wait_1_detect_1_form3_stage_1_cancers_5_posrisk_8_negrisk_40 | Patients | hypothetical |
| 0.095875 | 0.293398 | 0.611993 | pred_wait_1_detect_1_form3_stage_1_cancers_10_posrisk_8_negrisk_40 | Patients | hypothetical |
| 0.117168 | 0.34658 | 0.707796 | pred_wait_1_detect_1_form3_stage_1_cancers_25_posrisk_8_negrisk_40 | Patients | hypothetical |
| 0.177685 | 0.402604 | 0.630238 | pred_wait_1_detect_1_form3_stage_2_cancers_1_posrisk_2_negrisk_1 | Patients | hypothetical |
| 0.182846 | 0.418716 | 0.657271 | pred_wait_1_detect_1_form3_stage_2_cancers_5_posrisk_2_negrisk_1 | Patients | hypothetical |
| 0.191994 | 0.441408 | 0.692158 | pred_wait_1_detect_1_form3_stage_2_cancers_10_posrisk_2_negrisk_1 | Patients | hypothetical |
| 0.246904 | 0.52136 | 0.783577 | pred_wait_1_detect_1_form3_stage_2_cancers_25_posrisk_2_negrisk_1 | Patients | hypothetical |
| 0.172999 | 0.39881 | 0.628372 | pred_wait_1_detect_1_form3_stage_2_cancers_1_posrisk_2_negrisk_5 | Patients | hypothetical |
| 0.177665 | 0.414849 | 0.655619 | pred_wait_1_detect_1_form3_stage_2_cancers_5_posrisk_2_negrisk_5 | Patients | hypothetical |
| 0.186554 | 0.437535 | 0.690041 | pred_wait_1_detect_1_form3_stage_2_cancers_10_posrisk_2_negrisk_5 | Patients | hypothetical |
| 0.240509 | 0.518117 | 0.783733 | pred_wait_1_detect_1_form3_stage_2_cancers_25_posrisk_2_negrisk_5 | Patients | hypothetical |
| 0.166163 | 0.394238 | 0.625924 | pred_wait_1_detect_1_form3_stage_2_cancers_1_posrisk_2_negrisk_10 | Patients | hypothetical |
| 0.169755 | 0.410138 | 0.652845 | pred_wait_1_detect_1_form3_stage_2_cancers_5_posrisk_2_negrisk_10 | Patients | hypothetical |
| 0.177252 | 0.432739 | 0.688226 | pred_wait_1_detect_1_form3_stage_2_cancers_10_posrisk_2_negrisk_10 | Patients | hypothetical |
| 0.228034 | 0.513809 | 0.782569 | pred_wait_1_detect_1_form3_stage_2_cancers_25_posrisk_2_negrisk_10 | Patients | hypothetical |
| 0.147697 | 0.375481 | 0.619559 | pred_wait_1_detect_1_form3_stage_2_cancers_1_posrisk_2_negrisk_40 | Patients | hypothetical |
| 0.150532 | 0.39012 | 0.646715 | pred_wait_1_detect_1_form3_stage_2_cancers_5_posrisk_2_negrisk_40 | Patients | hypothetical |
| 0.155941 | 0.411075 | 0.681777 | pred_wait_1_detect_1_form3_stage_2_cancers_10_posrisk_2_negrisk_40 | Patients | hypothetical |
| 0.198626 | 0.488042 | 0.774579 | pred_wait_1_detect_1_form3_stage_2_cancers_25_posrisk_2_negrisk_40 | Patients | hypothetical |
| 0.110715 | 0.343384 | 0.620335 | pred_wait_1_detect_1_form3_stage_2_cancers_1_posrisk_4_negrisk_1 | Patients | hypothetical |
| 0.111862 | 0.356254 | 0.647194 | pred_wait_1_detect_1_form3_stage_2_cancers_5_posrisk_4_negrisk_1 | Patients | hypothetical |
| 0.115167 | 0.374947 | 0.681878 | pred_wait_1_detect_1_form3_stage_2_cancers_10_posrisk_4_negrisk_1 | Patients | hypothetical |
| 0.147273 | 0.446215 | 0.774045 | pred_wait_1_detect_1_form3_stage_2_cancers_25_posrisk_4_negrisk_1 | Patients | hypothetical |
| 0.110715 | 0.33959 | 0.615487 | pred_wait_1_detect_1_form3_stage_2_cancers_1_posrisk_4_negrisk_5 | Patients | hypothetical |
| 0.111862 | 0.352387 | 0.643274 | pred_wait_1_detect_1_form3_stage_2_cancers_5_posrisk_4_negrisk_5 | Patients | hypothetical |
| 0.115167 | 0.371075 | 0.679174 | pred_wait_1_detect_1_form3_stage_2_cancers_10_posrisk_4_negrisk_5 | Patients | hypothetical |
| 0.147273 | 0.442972 | 0.774488 | pred_wait_1_detect_1_form3_stage_2_cancers_25_posrisk_4_negrisk_5 | Patients | hypothetical |
| 0.110715 | 0.335018 | 0.60839 | pred_wait_1_detect_1_form3_stage_2_cancers_1_posrisk_4_negrisk_10 | Patients | hypothetical |
| 0.111862 | 0.347676 | 0.636821 | pred_wait_1_detect_1_form3_stage_2_cancers_5_posrisk_4_negrisk_10 | Patients | hypothetical |
| 0.115167 | 0.366279 | 0.673792 | pred_wait_1_detect_1_form3_stage_2_cancers_10_posrisk_4_negrisk_10 | Patients | hypothetical |
| 0.147273 | 0.438664 | 0.772624 | pred_wait_1_detect_1_form3_stage_2_cancers_25_posrisk_4_negrisk_10 | Patients | hypothetical |
| 0.110618 | 0.316262 | 0.555857 | pred_wait_1_detect_1_form3_stage_2_cancers_1_posrisk_4_negrisk_40 | Patients | hypothetical |
| 0.111767 | 0.327657 | 0.579561 | pred_wait_1_detect_1_form3_stage_2_cancers_5_posrisk_4_negrisk_40 | Patients | hypothetical |
| 0.115153 | 0.344615 | 0.611993 | pred_wait_1_detect_1_form3_stage_2_cancers_10_posrisk_4_negrisk_40 | Patients | hypothetical |
| 0.147273 | 0.412897 | 0.707796 | pred_wait_1_detect_1_form3_stage_2_cancers_25_posrisk_4_negrisk_40 | Patients | hypothetical |
| 0.089891 | 0.31147 | 0.620335 | pred_wait_1_detect_1_form3_stage_2_cancers_1_posrisk_6_negrisk_1 | Patients | hypothetical |
| 0.091778 | 0.322679 | 0.647194 | pred_wait_1_detect_1_form3_stage_2_cancers_5_posrisk_6_negrisk_1 | Patients | hypothetical |
| 0.095358 | 0.338942 | 0.681878 | pred_wait_1_detect_1_form3_stage_2_cancers_10_posrisk_6_negrisk_1 | Patients | hypothetical |
| 0.120116 | 0.40078 | 0.774045 | pred_wait_1_detect_1_form3_stage_2_cancers_25_posrisk_6_negrisk_1 | Patients | hypothetical |
| 0.089891 | 0.307676 | 0.615487 | pred_wait_1_detect_1_form3_stage_2_cancers_1_posrisk_6_negrisk_5 | Patients | hypothetical |
| 0.091778 | 0.318812 | 0.643274 | pred_wait_1_detect_1_form3_stage_2_cancers_5_posrisk_6_negrisk_5 | Patients | hypothetical |
| 0.095358 | 0.335069 | 0.679174 | pred_wait_1_detect_1_form3_stage_2_cancers_10_posrisk_6_negrisk_5 | Patients | hypothetical |
| 0.120116 | 0.397537 | 0.774488 | pred_wait_1_detect_1_form3_stage_2_cancers_25_posrisk_6_negrisk_5 | Patients | hypothetical |
| 0.089891 | 0.303104 | 0.60839 | pred_wait_1_detect_1_form3_stage_2_cancers_1_posrisk_6_negrisk_10 | Patients | hypothetical |
| 0.091778 | 0.314101 | 0.636821 | pred_wait_1_detect_1_form3_stage_2_cancers_5_posrisk_6_negrisk_10 | Patients | hypothetical |
| 0.095358 | 0.330273 | 0.673792 | pred_wait_1_detect_1_form3_stage_2_cancers_10_posrisk_6_negrisk_10 | Patients | hypothetical |
| 0.120116 | 0.393229 | 0.772624 | pred_wait_1_detect_1_form3_stage_2_cancers_25_posrisk_6_negrisk_10 | Patients | hypothetical |
| 0.089891 | 0.284348 | 0.555857 | pred_wait_1_detect_1_form3_stage_2_cancers_1_posrisk_6_negrisk_40 | Patients | hypothetical |
| 0.091778 | 0.294083 | 0.579561 | pred_wait_1_detect_1_form3_stage_2_cancers_5_posrisk_6_negrisk_40 | Patients | hypothetical |
| 0.095358 | 0.308609 | 0.611993 | pred_wait_1_detect_1_form3_stage_2_cancers_10_posrisk_6_negrisk_40 | Patients | hypothetical |
| 0.120116 | 0.367462 | 0.707796 | pred_wait_1_detect_1_form3_stage_2_cancers_25_posrisk_6_negrisk_40 | Patients | hypothetical |
| 0.089238 | 0.297922 | 0.620335 | pred_wait_1_detect_1_form3_stage_2_cancers_1_posrisk_8_negrisk_1 | Patients | hypothetical |
| 0.091829 | 0.308532 | 0.647194 | pred_wait_1_detect_1_form3_stage_2_cancers_5_posrisk_8_negrisk_1 | Patients | hypothetical |
| 0.095875 | 0.323731 | 0.681878 | pred_wait_1_detect_1_form3_stage_2_cancers_10_posrisk_8_negrisk_1 | Patients | hypothetical |
| 0.117168 | 0.379898 | 0.774045 | pred_wait_1_detect_1_form3_stage_2_cancers_25_posrisk_8_negrisk_1 | Patients | hypothetical |
| 0.089238 | 0.294128 | 0.615487 | pred_wait_1_detect_1_form3_stage_2_cancers_1_posrisk_8_negrisk_5 | Patients | hypothetical |
| 0.091829 | 0.304665 | 0.643274 | pred_wait_1_detect_1_form3_stage_2_cancers_5_posrisk_8_negrisk_5 | Patients | hypothetical |
| 0.095875 | 0.319858 | 0.679174 | pred_wait_1_detect_1_form3_stage_2_cancers_10_posrisk_8_negrisk_5 | Patients | hypothetical |
| 0.117168 | 0.376655 | 0.774488 | pred_wait_1_detect_1_form3_stage_2_cancers_25_posrisk_8_negrisk_5 | Patients | hypothetical |
| 0.089238 | 0.289556 | 0.60839 | pred_wait_1_detect_1_form3_stage_2_cancers_1_posrisk_8_negrisk_10 | Patients | hypothetical |
| 0.091829 | 0.299954 | 0.636821 | pred_wait_1_detect_1_form3_stage_2_cancers_5_posrisk_8_negrisk_10 | Patients | hypothetical |
| 0.095875 | 0.315062 | 0.673792 | pred_wait_1_detect_1_form3_stage_2_cancers_10_posrisk_8_negrisk_10 | Patients | hypothetical |
| 0.117168 | 0.372347 | 0.772624 | pred_wait_1_detect_1_form3_stage_2_cancers_25_posrisk_8_negrisk_10 | Patients | hypothetical |
| 0.089238 | 0.2708 | 0.555857 | pred_wait_1_detect_1_form3_stage_2_cancers_1_posrisk_8_negrisk_40 | Patients | hypothetical |
| 0.091829 | 0.279935 | 0.579561 | pred_wait_1_detect_1_form3_stage_2_cancers_5_posrisk_8_negrisk_40 | Patients | hypothetical |
| 0.095875 | 0.293398 | 0.611993 | pred_wait_1_detect_1_form3_stage_2_cancers_10_posrisk_8_negrisk_40 | Patients | hypothetical |
| 0.117168 | 0.34658 | 0.707796 | pred_wait_1_detect_1_form3_stage_2_cancers_25_posrisk_8_negrisk_40 | Patients | hypothetical |
| 0.177685 | 0.402604 | 0.630238 | pred_wait_1_detect_1_form4_stage_1_cancers_1_posrisk_2_negrisk_1 | Patients | hypothetical |
| 0.182846 | 0.418716 | 0.657271 | pred_wait_1_detect_1_form4_stage_1_cancers_5_posrisk_2_negrisk_1 | Patients | hypothetical |
| 0.191994 | 0.441408 | 0.692158 | pred_wait_1_detect_1_form4_stage_1_cancers_10_posrisk_2_negrisk_1 | Patients | hypothetical |
| 0.246904 | 0.52136 | 0.783577 | pred_wait_1_detect_1_form4_stage_1_cancers_25_posrisk_2_negrisk_1 | Patients | hypothetical |
| 0.172999 | 0.39881 | 0.628372 | pred_wait_1_detect_1_form4_stage_1_cancers_1_posrisk_2_negrisk_5 | Patients | hypothetical |
| 0.177665 | 0.414849 | 0.655619 | pred_wait_1_detect_1_form4_stage_1_cancers_5_posrisk_2_negrisk_5 | Patients | hypothetical |
| 0.186554 | 0.437535 | 0.690041 | pred_wait_1_detect_1_form4_stage_1_cancers_10_posrisk_2_negrisk_5 | Patients | hypothetical |
| 0.240509 | 0.518117 | 0.783733 | pred_wait_1_detect_1_form4_stage_1_cancers_25_posrisk_2_negrisk_5 | Patients | hypothetical |
| 0.166163 | 0.394238 | 0.625924 | pred_wait_1_detect_1_form4_stage_1_cancers_1_posrisk_2_negrisk_10 | Patients | hypothetical |
| 0.169755 | 0.410138 | 0.652845 | pred_wait_1_detect_1_form4_stage_1_cancers_5_posrisk_2_negrisk_10 | Patients | hypothetical |
| 0.177252 | 0.432739 | 0.688226 | pred_wait_1_detect_1_form4_stage_1_cancers_10_posrisk_2_negrisk_10 | Patients | hypothetical |
| 0.228034 | 0.513809 | 0.782569 | pred_wait_1_detect_1_form4_stage_1_cancers_25_posrisk_2_negrisk_10 | Patients | hypothetical |
| 0.147697 | 0.375481 | 0.619559 | pred_wait_1_detect_1_form4_stage_1_cancers_1_posrisk_2_negrisk_40 | Patients | hypothetical |
| 0.150532 | 0.39012 | 0.646715 | pred_wait_1_detect_1_form4_stage_1_cancers_5_posrisk_2_negrisk_40 | Patients | hypothetical |
| 0.155941 | 0.411075 | 0.681777 | pred_wait_1_detect_1_form4_stage_1_cancers_10_posrisk_2_negrisk_40 | Patients | hypothetical |
| 0.198626 | 0.488042 | 0.774579 | pred_wait_1_detect_1_form4_stage_1_cancers_25_posrisk_2_negrisk_40 | Patients | hypothetical |
| 0.110715 | 0.343384 | 0.620335 | pred_wait_1_detect_1_form4_stage_1_cancers_1_posrisk_4_negrisk_1 | Patients | hypothetical |
| 0.111862 | 0.356254 | 0.647194 | pred_wait_1_detect_1_form4_stage_1_cancers_5_posrisk_4_negrisk_1 | Patients | hypothetical |
| 0.115167 | 0.374947 | 0.681878 | pred_wait_1_detect_1_form4_stage_1_cancers_10_posrisk_4_negrisk_1 | Patients | hypothetical |
| 0.147273 | 0.446215 | 0.774045 | pred_wait_1_detect_1_form4_stage_1_cancers_25_posrisk_4_negrisk_1 | Patients | hypothetical |
| 0.110715 | 0.33959 | 0.615487 | pred_wait_1_detect_1_form4_stage_1_cancers_1_posrisk_4_negrisk_5 | Patients | hypothetical |
| 0.111862 | 0.352387 | 0.643274 | pred_wait_1_detect_1_form4_stage_1_cancers_5_posrisk_4_negrisk_5 | Patients | hypothetical |
| 0.115167 | 0.371075 | 0.679174 | pred_wait_1_detect_1_form4_stage_1_cancers_10_posrisk_4_negrisk_5 | Patients | hypothetical |
| 0.147273 | 0.442972 | 0.774488 | pred_wait_1_detect_1_form4_stage_1_cancers_25_posrisk_4_negrisk_5 | Patients | hypothetical |
| 0.110715 | 0.335018 | 0.60839 | pred_wait_1_detect_1_form4_stage_1_cancers_1_posrisk_4_negrisk_10 | Patients | hypothetical |
| 0.111862 | 0.347676 | 0.636821 | pred_wait_1_detect_1_form4_stage_1_cancers_5_posrisk_4_negrisk_10 | Patients | hypothetical |
| 0.115167 | 0.366279 | 0.673792 | pred_wait_1_detect_1_form4_stage_1_cancers_10_posrisk_4_negrisk_10 | Patients | hypothetical |
| 0.147273 | 0.438664 | 0.772624 | pred_wait_1_detect_1_form4_stage_1_cancers_25_posrisk_4_negrisk_10 | Patients | hypothetical |
| 0.110618 | 0.316262 | 0.555857 | pred_wait_1_detect_1_form4_stage_1_cancers_1_posrisk_4_negrisk_40 | Patients | hypothetical |
| 0.111767 | 0.327657 | 0.579561 | pred_wait_1_detect_1_form4_stage_1_cancers_5_posrisk_4_negrisk_40 | Patients | hypothetical |
| 0.115153 | 0.344615 | 0.611993 | pred_wait_1_detect_1_form4_stage_1_cancers_10_posrisk_4_negrisk_40 | Patients | hypothetical |
| 0.147273 | 0.412897 | 0.707796 | pred_wait_1_detect_1_form4_stage_1_cancers_25_posrisk_4_negrisk_40 | Patients | hypothetical |
| 0.089891 | 0.31147 | 0.620335 | pred_wait_1_detect_1_form4_stage_1_cancers_1_posrisk_6_negrisk_1 | Patients | hypothetical |
| 0.091778 | 0.322679 | 0.647194 | pred_wait_1_detect_1_form4_stage_1_cancers_5_posrisk_6_negrisk_1 | Patients | hypothetical |
| 0.095358 | 0.338942 | 0.681878 | pred_wait_1_detect_1_form4_stage_1_cancers_10_posrisk_6_negrisk_1 | Patients | hypothetical |
| 0.120116 | 0.40078 | 0.774045 | pred_wait_1_detect_1_form4_stage_1_cancers_25_posrisk_6_negrisk_1 | Patients | hypothetical |
| 0.089891 | 0.307676 | 0.615487 | pred_wait_1_detect_1_form4_stage_1_cancers_1_posrisk_6_negrisk_5 | Patients | hypothetical |
| 0.091778 | 0.318812 | 0.643274 | pred_wait_1_detect_1_form4_stage_1_cancers_5_posrisk_6_negrisk_5 | Patients | hypothetical |
| 0.095358 | 0.335069 | 0.679174 | pred_wait_1_detect_1_form4_stage_1_cancers_10_posrisk_6_negrisk_5 | Patients | hypothetical |
| 0.120116 | 0.397537 | 0.774488 | pred_wait_1_detect_1_form4_stage_1_cancers_25_posrisk_6_negrisk_5 | Patients | hypothetical |
| 0.089891 | 0.303104 | 0.60839 | pred_wait_1_detect_1_form4_stage_1_cancers_1_posrisk_6_negrisk_10 | Patients | hypothetical |
| 0.091778 | 0.314101 | 0.636821 | pred_wait_1_detect_1_form4_stage_1_cancers_5_posrisk_6_negrisk_10 | Patients | hypothetical |
| 0.095358 | 0.330273 | 0.673792 | pred_wait_1_detect_1_form4_stage_1_cancers_10_posrisk_6_negrisk_10 | Patients | hypothetical |
| 0.120116 | 0.393229 | 0.772624 | pred_wait_1_detect_1_form4_stage_1_cancers_25_posrisk_6_negrisk_10 | Patients | hypothetical |
| 0.089891 | 0.284348 | 0.555857 | pred_wait_1_detect_1_form4_stage_1_cancers_1_posrisk_6_negrisk_40 | Patients | hypothetical |
| 0.091778 | 0.294083 | 0.579561 | pred_wait_1_detect_1_form4_stage_1_cancers_5_posrisk_6_negrisk_40 | Patients | hypothetical |
| 0.095358 | 0.308609 | 0.611993 | pred_wait_1_detect_1_form4_stage_1_cancers_10_posrisk_6_negrisk_40 | Patients | hypothetical |
| 0.120116 | 0.367462 | 0.707796 | pred_wait_1_detect_1_form4_stage_1_cancers_25_posrisk_6_negrisk_40 | Patients | hypothetical |
| 0.089238 | 0.297922 | 0.620335 | pred_wait_1_detect_1_form4_stage_1_cancers_1_posrisk_8_negrisk_1 | Patients | hypothetical |
| 0.091829 | 0.308532 | 0.647194 | pred_wait_1_detect_1_form4_stage_1_cancers_5_posrisk_8_negrisk_1 | Patients | hypothetical |
| 0.095875 | 0.323731 | 0.681878 | pred_wait_1_detect_1_form4_stage_1_cancers_10_posrisk_8_negrisk_1 | Patients | hypothetical |
| 0.117168 | 0.379898 | 0.774045 | pred_wait_1_detect_1_form4_stage_1_cancers_25_posrisk_8_negrisk_1 | Patients | hypothetical |
| 0.089238 | 0.294128 | 0.615487 | pred_wait_1_detect_1_form4_stage_1_cancers_1_posrisk_8_negrisk_5 | Patients | hypothetical |
| 0.091829 | 0.304665 | 0.643274 | pred_wait_1_detect_1_form4_stage_1_cancers_5_posrisk_8_negrisk_5 | Patients | hypothetical |
| 0.095875 | 0.319858 | 0.679174 | pred_wait_1_detect_1_form4_stage_1_cancers_10_posrisk_8_negrisk_5 | Patients | hypothetical |
| 0.117168 | 0.376655 | 0.774488 | pred_wait_1_detect_1_form4_stage_1_cancers_25_posrisk_8_negrisk_5 | Patients | hypothetical |
| 0.089238 | 0.289556 | 0.60839 | pred_wait_1_detect_1_form4_stage_1_cancers_1_posrisk_8_negrisk_10 | Patients | hypothetical |
| 0.091829 | 0.299954 | 0.636821 | pred_wait_1_detect_1_form4_stage_1_cancers_5_posrisk_8_negrisk_10 | Patients | hypothetical |
| 0.095875 | 0.315062 | 0.673792 | pred_wait_1_detect_1_form4_stage_1_cancers_10_posrisk_8_negrisk_10 | Patients | hypothetical |
| 0.117168 | 0.372347 | 0.772624 | pred_wait_1_detect_1_form4_stage_1_cancers_25_posrisk_8_negrisk_10 | Patients | hypothetical |
| 0.089238 | 0.2708 | 0.555857 | pred_wait_1_detect_1_form4_stage_1_cancers_1_posrisk_8_negrisk_40 | Patients | hypothetical |
| 0.091829 | 0.279935 | 0.579561 | pred_wait_1_detect_1_form4_stage_1_cancers_5_posrisk_8_negrisk_40 | Patients | hypothetical |
| 0.095875 | 0.293398 | 0.611993 | pred_wait_1_detect_1_form4_stage_1_cancers_10_posrisk_8_negrisk_40 | Patients | hypothetical |
| 0.117168 | 0.34658 | 0.707796 | pred_wait_1_detect_1_form4_stage_1_cancers_25_posrisk_8_negrisk_40 | Patients | hypothetical |
| 0.177685 | 0.402604 | 0.630238 | pred_wait_1_detect_1_form4_stage_2_cancers_1_posrisk_2_negrisk_1 | Patients | hypothetical |
| 0.182846 | 0.418716 | 0.657271 | pred_wait_1_detect_1_form4_stage_2_cancers_5_posrisk_2_negrisk_1 | Patients | hypothetical |
| 0.191994 | 0.441408 | 0.692158 | pred_wait_1_detect_1_form4_stage_2_cancers_10_posrisk_2_negrisk_1 | Patients | hypothetical |
| 0.246904 | 0.52136 | 0.783577 | pred_wait_1_detect_1_form4_stage_2_cancers_25_posrisk_2_negrisk_1 | Patients | hypothetical |
| 0.172999 | 0.39881 | 0.628372 | pred_wait_1_detect_1_form4_stage_2_cancers_1_posrisk_2_negrisk_5 | Patients | hypothetical |
| 0.177665 | 0.414849 | 0.655619 | pred_wait_1_detect_1_form4_stage_2_cancers_5_posrisk_2_negrisk_5 | Patients | hypothetical |
| 0.186554 | 0.437535 | 0.690041 | pred_wait_1_detect_1_form4_stage_2_cancers_10_posrisk_2_negrisk_5 | Patients | hypothetical |
| 0.240509 | 0.518117 | 0.783733 | pred_wait_1_detect_1_form4_stage_2_cancers_25_posrisk_2_negrisk_5 | Patients | hypothetical |
| 0.166163 | 0.394238 | 0.625924 | pred_wait_1_detect_1_form4_stage_2_cancers_1_posrisk_2_negrisk_10 | Patients | hypothetical |
| 0.169755 | 0.410138 | 0.652845 | pred_wait_1_detect_1_form4_stage_2_cancers_5_posrisk_2_negrisk_10 | Patients | hypothetical |
| 0.177252 | 0.432739 | 0.688226 | pred_wait_1_detect_1_form4_stage_2_cancers_10_posrisk_2_negrisk_10 | Patients | hypothetical |
| 0.228034 | 0.513809 | 0.782569 | pred_wait_1_detect_1_form4_stage_2_cancers_25_posrisk_2_negrisk_10 | Patients | hypothetical |
| 0.147697 | 0.375481 | 0.619559 | pred_wait_1_detect_1_form4_stage_2_cancers_1_posrisk_2_negrisk_40 | Patients | hypothetical |
| 0.150532 | 0.39012 | 0.646715 | pred_wait_1_detect_1_form4_stage_2_cancers_5_posrisk_2_negrisk_40 | Patients | hypothetical |
| 0.155941 | 0.411075 | 0.681777 | pred_wait_1_detect_1_form4_stage_2_cancers_10_posrisk_2_negrisk_40 | Patients | hypothetical |
| 0.198626 | 0.488042 | 0.774579 | pred_wait_1_detect_1_form4_stage_2_cancers_25_posrisk_2_negrisk_40 | Patients | hypothetical |
| 0.110715 | 0.343384 | 0.620335 | pred_wait_1_detect_1_form4_stage_2_cancers_1_posrisk_4_negrisk_1 | Patients | hypothetical |
| 0.111862 | 0.356254 | 0.647194 | pred_wait_1_detect_1_form4_stage_2_cancers_5_posrisk_4_negrisk_1 | Patients | hypothetical |
| 0.115167 | 0.374947 | 0.681878 | pred_wait_1_detect_1_form4_stage_2_cancers_10_posrisk_4_negrisk_1 | Patients | hypothetical |
| 0.147273 | 0.446215 | 0.774045 | pred_wait_1_detect_1_form4_stage_2_cancers_25_posrisk_4_negrisk_1 | Patients | hypothetical |
| 0.110715 | 0.33959 | 0.615487 | pred_wait_1_detect_1_form4_stage_2_cancers_1_posrisk_4_negrisk_5 | Patients | hypothetical |
| 0.111862 | 0.352387 | 0.643274 | pred_wait_1_detect_1_form4_stage_2_cancers_5_posrisk_4_negrisk_5 | Patients | hypothetical |
| 0.115167 | 0.371075 | 0.679174 | pred_wait_1_detect_1_form4_stage_2_cancers_10_posrisk_4_negrisk_5 | Patients | hypothetical |
| 0.147273 | 0.442972 | 0.774488 | pred_wait_1_detect_1_form4_stage_2_cancers_25_posrisk_4_negrisk_5 | Patients | hypothetical |
| 0.110715 | 0.335018 | 0.60839 | pred_wait_1_detect_1_form4_stage_2_cancers_1_posrisk_4_negrisk_10 | Patients | hypothetical |
| 0.111862 | 0.347676 | 0.636821 | pred_wait_1_detect_1_form4_stage_2_cancers_5_posrisk_4_negrisk_10 | Patients | hypothetical |
| 0.115167 | 0.366279 | 0.673792 | pred_wait_1_detect_1_form4_stage_2_cancers_10_posrisk_4_negrisk_10 | Patients | hypothetical |
| 0.147273 | 0.438664 | 0.772624 | pred_wait_1_detect_1_form4_stage_2_cancers_25_posrisk_4_negrisk_10 | Patients | hypothetical |
| 0.110618 | 0.316262 | 0.555857 | pred_wait_1_detect_1_form4_stage_2_cancers_1_posrisk_4_negrisk_40 | Patients | hypothetical |
| 0.111767 | 0.327657 | 0.579561 | pred_wait_1_detect_1_form4_stage_2_cancers_5_posrisk_4_negrisk_40 | Patients | hypothetical |
| 0.115153 | 0.344615 | 0.611993 | pred_wait_1_detect_1_form4_stage_2_cancers_10_posrisk_4_negrisk_40 | Patients | hypothetical |
| 0.147273 | 0.412897 | 0.707796 | pred_wait_1_detect_1_form4_stage_2_cancers_25_posrisk_4_negrisk_40 | Patients | hypothetical |
| 0.089891 | 0.31147 | 0.620335 | pred_wait_1_detect_1_form4_stage_2_cancers_1_posrisk_6_negrisk_1 | Patients | hypothetical |
| 0.091778 | 0.322679 | 0.647194 | pred_wait_1_detect_1_form4_stage_2_cancers_5_posrisk_6_negrisk_1 | Patients | hypothetical |
| 0.095358 | 0.338942 | 0.681878 | pred_wait_1_detect_1_form4_stage_2_cancers_10_posrisk_6_negrisk_1 | Patients | hypothetical |
| 0.120116 | 0.40078 | 0.774045 | pred_wait_1_detect_1_form4_stage_2_cancers_25_posrisk_6_negrisk_1 | Patients | hypothetical |
| 0.089891 | 0.307676 | 0.615487 | pred_wait_1_detect_1_form4_stage_2_cancers_1_posrisk_6_negrisk_5 | Patients | hypothetical |
| 0.091778 | 0.318812 | 0.643274 | pred_wait_1_detect_1_form4_stage_2_cancers_5_posrisk_6_negrisk_5 | Patients | hypothetical |
| 0.095358 | 0.335069 | 0.679174 | pred_wait_1_detect_1_form4_stage_2_cancers_10_posrisk_6_negrisk_5 | Patients | hypothetical |
| 0.120116 | 0.397537 | 0.774488 | pred_wait_1_detect_1_form4_stage_2_cancers_25_posrisk_6_negrisk_5 | Patients | hypothetical |
| 0.089891 | 0.303104 | 0.60839 | pred_wait_1_detect_1_form4_stage_2_cancers_1_posrisk_6_negrisk_10 | Patients | hypothetical |
| 0.091778 | 0.314101 | 0.636821 | pred_wait_1_detect_1_form4_stage_2_cancers_5_posrisk_6_negrisk_10 | Patients | hypothetical |
| 0.095358 | 0.330273 | 0.673792 | pred_wait_1_detect_1_form4_stage_2_cancers_10_posrisk_6_negrisk_10 | Patients | hypothetical |
| 0.120116 | 0.393229 | 0.772624 | pred_wait_1_detect_1_form4_stage_2_cancers_25_posrisk_6_negrisk_10 | Patients | hypothetical |
| 0.089891 | 0.284348 | 0.555857 | pred_wait_1_detect_1_form4_stage_2_cancers_1_posrisk_6_negrisk_40 | Patients | hypothetical |
| 0.091778 | 0.294083 | 0.579561 | pred_wait_1_detect_1_form4_stage_2_cancers_5_posrisk_6_negrisk_40 | Patients | hypothetical |
| 0.095358 | 0.308609 | 0.611993 | pred_wait_1_detect_1_form4_stage_2_cancers_10_posrisk_6_negrisk_40 | Patients | hypothetical |
| 0.120116 | 0.367462 | 0.707796 | pred_wait_1_detect_1_form4_stage_2_cancers_25_posrisk_6_negrisk_40 | Patients | hypothetical |
| 0.089238 | 0.297922 | 0.620335 | pred_wait_1_detect_1_form4_stage_2_cancers_1_posrisk_8_negrisk_1 | Patients | hypothetical |
| 0.091829 | 0.308532 | 0.647194 | pred_wait_1_detect_1_form4_stage_2_cancers_5_posrisk_8_negrisk_1 | Patients | hypothetical |
| 0.095875 | 0.323731 | 0.681878 | pred_wait_1_detect_1_form4_stage_2_cancers_10_posrisk_8_negrisk_1 | Patients | hypothetical |
| 0.117168 | 0.379898 | 0.774045 | pred_wait_1_detect_1_form4_stage_2_cancers_25_posrisk_8_negrisk_1 | Patients | hypothetical |
| 0.089238 | 0.294128 | 0.615487 | pred_wait_1_detect_1_form4_stage_2_cancers_1_posrisk_8_negrisk_5 | Patients | hypothetical |
| 0.091829 | 0.304665 | 0.643274 | pred_wait_1_detect_1_form4_stage_2_cancers_5_posrisk_8_negrisk_5 | Patients | hypothetical |
| 0.095875 | 0.319858 | 0.679174 | pred_wait_1_detect_1_form4_stage_2_cancers_10_posrisk_8_negrisk_5 | Patients | hypothetical |
| 0.117168 | 0.376655 | 0.774488 | pred_wait_1_detect_1_form4_stage_2_cancers_25_posrisk_8_negrisk_5 | Patients | hypothetical |
| 0.089238 | 0.289556 | 0.60839 | pred_wait_1_detect_1_form4_stage_2_cancers_1_posrisk_8_negrisk_10 | Patients | hypothetical |
| 0.091829 | 0.299954 | 0.636821 | pred_wait_1_detect_1_form4_stage_2_cancers_5_posrisk_8_negrisk_10 | Patients | hypothetical |
| 0.095875 | 0.315062 | 0.673792 | pred_wait_1_detect_1_form4_stage_2_cancers_10_posrisk_8_negrisk_10 | Patients | hypothetical |
| 0.117168 | 0.372347 | 0.772624 | pred_wait_1_detect_1_form4_stage_2_cancers_25_posrisk_8_negrisk_10 | Patients | hypothetical |
| 0.089238 | 0.2708 | 0.555857 | pred_wait_1_detect_1_form4_stage_2_cancers_1_posrisk_8_negrisk_40 | Patients | hypothetical |
| 0.091829 | 0.279935 | 0.579561 | pred_wait_1_detect_1_form4_stage_2_cancers_5_posrisk_8_negrisk_40 | Patients | hypothetical |
| 0.095875 | 0.293398 | 0.611993 | pred_wait_1_detect_1_form4_stage_2_cancers_10_posrisk_8_negrisk_40 | Patients | hypothetical |
| 0.117168 | 0.34658 | 0.707796 | pred_wait_1_detect_1_form4_stage_2_cancers_25_posrisk_8_negrisk_40 | Patients | hypothetical |
| 0.177685 | 0.402604 | 0.630238 | pred_wait_1_detect_2_form1_stage_1_cancers_1_posrisk_2_negrisk_1 | Patients | hypothetical |
| 0.182846 | 0.418716 | 0.657271 | pred_wait_1_detect_2_form1_stage_1_cancers_5_posrisk_2_negrisk_1 | Patients | hypothetical |
| 0.191994 | 0.441408 | 0.692158 | pred_wait_1_detect_2_form1_stage_1_cancers_10_posrisk_2_negrisk_1 | Patients | hypothetical |
| 0.246904 | 0.52136 | 0.783577 | pred_wait_1_detect_2_form1_stage_1_cancers_25_posrisk_2_negrisk_1 | Patients | hypothetical |
| 0.172999 | 0.39881 | 0.628372 | pred_wait_1_detect_2_form1_stage_1_cancers_1_posrisk_2_negrisk_5 | Patients | hypothetical |
| 0.177665 | 0.414849 | 0.655619 | pred_wait_1_detect_2_form1_stage_1_cancers_5_posrisk_2_negrisk_5 | Patients | hypothetical |
| 0.186554 | 0.437535 | 0.690041 | pred_wait_1_detect_2_form1_stage_1_cancers_10_posrisk_2_negrisk_5 | Patients | hypothetical |
| 0.240509 | 0.518117 | 0.783733 | pred_wait_1_detect_2_form1_stage_1_cancers_25_posrisk_2_negrisk_5 | Patients | hypothetical |
| 0.166163 | 0.394238 | 0.625924 | pred_wait_1_detect_2_form1_stage_1_cancers_1_posrisk_2_negrisk_10 | Patients | hypothetical |
| 0.169755 | 0.410138 | 0.652845 | pred_wait_1_detect_2_form1_stage_1_cancers_5_posrisk_2_negrisk_10 | Patients | hypothetical |
| 0.177252 | 0.432739 | 0.688226 | pred_wait_1_detect_2_form1_stage_1_cancers_10_posrisk_2_negrisk_10 | Patients | hypothetical |
| 0.228034 | 0.513809 | 0.782569 | pred_wait_1_detect_2_form1_stage_1_cancers_25_posrisk_2_negrisk_10 | Patients | hypothetical |
| 0.147697 | 0.375481 | 0.619559 | pred_wait_1_detect_2_form1_stage_1_cancers_1_posrisk_2_negrisk_40 | Patients | hypothetical |
| 0.150532 | 0.39012 | 0.646715 | pred_wait_1_detect_2_form1_stage_1_cancers_5_posrisk_2_negrisk_40 | Patients | hypothetical |
| 0.155941 | 0.411075 | 0.681777 | pred_wait_1_detect_2_form1_stage_1_cancers_10_posrisk_2_negrisk_40 | Patients | hypothetical |
| 0.198626 | 0.488042 | 0.774579 | pred_wait_1_detect_2_form1_stage_1_cancers_25_posrisk_2_negrisk_40 | Patients | hypothetical |
| 0.110715 | 0.343384 | 0.620335 | pred_wait_1_detect_2_form1_stage_1_cancers_1_posrisk_4_negrisk_1 | Patients | hypothetical |
| 0.111862 | 0.356254 | 0.647194 | pred_wait_1_detect_2_form1_stage_1_cancers_5_posrisk_4_negrisk_1 | Patients | hypothetical |
| 0.115167 | 0.374947 | 0.681878 | pred_wait_1_detect_2_form1_stage_1_cancers_10_posrisk_4_negrisk_1 | Patients | hypothetical |
| 0.147273 | 0.446215 | 0.774045 | pred_wait_1_detect_2_form1_stage_1_cancers_25_posrisk_4_negrisk_1 | Patients | hypothetical |
| 0.110715 | 0.33959 | 0.615487 | pred_wait_1_detect_2_form1_stage_1_cancers_1_posrisk_4_negrisk_5 | Patients | hypothetical |
| 0.111862 | 0.352387 | 0.643274 | pred_wait_1_detect_2_form1_stage_1_cancers_5_posrisk_4_negrisk_5 | Patients | hypothetical |
| 0.115167 | 0.371075 | 0.679174 | pred_wait_1_detect_2_form1_stage_1_cancers_10_posrisk_4_negrisk_5 | Patients | hypothetical |
| 0.147273 | 0.442972 | 0.774488 | pred_wait_1_detect_2_form1_stage_1_cancers_25_posrisk_4_negrisk_5 | Patients | hypothetical |
| 0.110715 | 0.335018 | 0.60839 | pred_wait_1_detect_2_form1_stage_1_cancers_1_posrisk_4_negrisk_10 | Patients | hypothetical |
| 0.111862 | 0.347676 | 0.636821 | pred_wait_1_detect_2_form1_stage_1_cancers_5_posrisk_4_negrisk_10 | Patients | hypothetical |
| 0.115167 | 0.366279 | 0.673792 | pred_wait_1_detect_2_form1_stage_1_cancers_10_posrisk_4_negrisk_10 | Patients | hypothetical |
| 0.147273 | 0.438664 | 0.772624 | pred_wait_1_detect_2_form1_stage_1_cancers_25_posrisk_4_negrisk_10 | Patients | hypothetical |
| 0.110618 | 0.316262 | 0.555857 | pred_wait_1_detect_2_form1_stage_1_cancers_1_posrisk_4_negrisk_40 | Patients | hypothetical |
| 0.111767 | 0.327657 | 0.579561 | pred_wait_1_detect_2_form1_stage_1_cancers_5_posrisk_4_negrisk_40 | Patients | hypothetical |
| 0.115153 | 0.344615 | 0.611993 | pred_wait_1_detect_2_form1_stage_1_cancers_10_posrisk_4_negrisk_40 | Patients | hypothetical |
| 0.147273 | 0.412897 | 0.707796 | pred_wait_1_detect_2_form1_stage_1_cancers_25_posrisk_4_negrisk_40 | Patients | hypothetical |
| 0.089891 | 0.31147 | 0.620335 | pred_wait_1_detect_2_form1_stage_1_cancers_1_posrisk_6_negrisk_1 | Patients | hypothetical |
| 0.091778 | 0.322679 | 0.647194 | pred_wait_1_detect_2_form1_stage_1_cancers_5_posrisk_6_negrisk_1 | Patients | hypothetical |
| 0.095358 | 0.338942 | 0.681878 | pred_wait_1_detect_2_form1_stage_1_cancers_10_posrisk_6_negrisk_1 | Patients | hypothetical |
| 0.120116 | 0.40078 | 0.774045 | pred_wait_1_detect_2_form1_stage_1_cancers_25_posrisk_6_negrisk_1 | Patients | hypothetical |
| 0.089891 | 0.307676 | 0.615487 | pred_wait_1_detect_2_form1_stage_1_cancers_1_posrisk_6_negrisk_5 | Patients | hypothetical |
| 0.091778 | 0.318812 | 0.643274 | pred_wait_1_detect_2_form1_stage_1_cancers_5_posrisk_6_negrisk_5 | Patients | hypothetical |
| 0.095358 | 0.335069 | 0.679174 | pred_wait_1_detect_2_form1_stage_1_cancers_10_posrisk_6_negrisk_5 | Patients | hypothetical |
| 0.120116 | 0.397537 | 0.774488 | pred_wait_1_detect_2_form1_stage_1_cancers_25_posrisk_6_negrisk_5 | Patients | hypothetical |
| 0.089891 | 0.303104 | 0.60839 | pred_wait_1_detect_2_form1_stage_1_cancers_1_posrisk_6_negrisk_10 | Patients | hypothetical |
| 0.091778 | 0.314101 | 0.636821 | pred_wait_1_detect_2_form1_stage_1_cancers_5_posrisk_6_negrisk_10 | Patients | hypothetical |
| 0.095358 | 0.330273 | 0.673792 | pred_wait_1_detect_2_form1_stage_1_cancers_10_posrisk_6_negrisk_10 | Patients | hypothetical |
| 0.120116 | 0.393229 | 0.772624 | pred_wait_1_detect_2_form1_stage_1_cancers_25_posrisk_6_negrisk_10 | Patients | hypothetical |
| 0.089891 | 0.284348 | 0.555857 | pred_wait_1_detect_2_form1_stage_1_cancers_1_posrisk_6_negrisk_40 | Patients | hypothetical |
| 0.091778 | 0.294083 | 0.579561 | pred_wait_1_detect_2_form1_stage_1_cancers_5_posrisk_6_negrisk_40 | Patients | hypothetical |
| 0.095358 | 0.308609 | 0.611993 | pred_wait_1_detect_2_form1_stage_1_cancers_10_posrisk_6_negrisk_40 | Patients | hypothetical |
| 0.120116 | 0.367462 | 0.707796 | pred_wait_1_detect_2_form1_stage_1_cancers_25_posrisk_6_negrisk_40 | Patients | hypothetical |
| 0.089238 | 0.297922 | 0.620335 | pred_wait_1_detect_2_form1_stage_1_cancers_1_posrisk_8_negrisk_1 | Patients | hypothetical |
| 0.091829 | 0.308532 | 0.647194 | pred_wait_1_detect_2_form1_stage_1_cancers_5_posrisk_8_negrisk_1 | Patients | hypothetical |
| 0.095875 | 0.323731 | 0.681878 | pred_wait_1_detect_2_form1_stage_1_cancers_10_posrisk_8_negrisk_1 | Patients | hypothetical |
| 0.117168 | 0.379898 | 0.774045 | pred_wait_1_detect_2_form1_stage_1_cancers_25_posrisk_8_negrisk_1 | Patients | hypothetical |
| 0.089238 | 0.294128 | 0.615487 | pred_wait_1_detect_2_form1_stage_1_cancers_1_posrisk_8_negrisk_5 | Patients | hypothetical |
| 0.091829 | 0.304665 | 0.643274 | pred_wait_1_detect_2_form1_stage_1_cancers_5_posrisk_8_negrisk_5 | Patients | hypothetical |
| 0.095875 | 0.319858 | 0.679174 | pred_wait_1_detect_2_form1_stage_1_cancers_10_posrisk_8_negrisk_5 | Patients | hypothetical |
| 0.117168 | 0.376655 | 0.774488 | pred_wait_1_detect_2_form1_stage_1_cancers_25_posrisk_8_negrisk_5 | Patients | hypothetical |
| 0.089238 | 0.289556 | 0.60839 | pred_wait_1_detect_2_form1_stage_1_cancers_1_posrisk_8_negrisk_10 | Patients | hypothetical |
| 0.091829 | 0.299954 | 0.636821 | pred_wait_1_detect_2_form1_stage_1_cancers_5_posrisk_8_negrisk_10 | Patients | hypothetical |
| 0.095875 | 0.315062 | 0.673792 | pred_wait_1_detect_2_form1_stage_1_cancers_10_posrisk_8_negrisk_10 | Patients | hypothetical |
| 0.117168 | 0.372347 | 0.772624 | pred_wait_1_detect_2_form1_stage_1_cancers_25_posrisk_8_negrisk_10 | Patients | hypothetical |
| 0.089238 | 0.2708 | 0.555857 | pred_wait_1_detect_2_form1_stage_1_cancers_1_posrisk_8_negrisk_40 | Patients | hypothetical |
| 0.091829 | 0.279935 | 0.579561 | pred_wait_1_detect_2_form1_stage_1_cancers_5_posrisk_8_negrisk_40 | Patients | hypothetical |
| 0.095875 | 0.293398 | 0.611993 | pred_wait_1_detect_2_form1_stage_1_cancers_10_posrisk_8_negrisk_40 | Patients | hypothetical |
| 0.117168 | 0.34658 | 0.707796 | pred_wait_1_detect_2_form1_stage_1_cancers_25_posrisk_8_negrisk_40 | Patients | hypothetical |
| 0.177685 | 0.402604 | 0.630238 | pred_wait_1_detect_2_form1_stage_2_cancers_1_posrisk_2_negrisk_1 | Patients | hypothetical |
| 0.182846 | 0.418716 | 0.657271 | pred_wait_1_detect_2_form1_stage_2_cancers_5_posrisk_2_negrisk_1 | Patients | hypothetical |
| 0.191994 | 0.441408 | 0.692158 | pred_wait_1_detect_2_form1_stage_2_cancers_10_posrisk_2_negrisk_1 | Patients | hypothetical |
| 0.246904 | 0.52136 | 0.783577 | pred_wait_1_detect_2_form1_stage_2_cancers_25_posrisk_2_negrisk_1 | Patients | hypothetical |
| 0.172999 | 0.39881 | 0.628372 | pred_wait_1_detect_2_form1_stage_2_cancers_1_posrisk_2_negrisk_5 | Patients | hypothetical |
| 0.177665 | 0.414849 | 0.655619 | pred_wait_1_detect_2_form1_stage_2_cancers_5_posrisk_2_negrisk_5 | Patients | hypothetical |
| 0.186554 | 0.437535 | 0.690041 | pred_wait_1_detect_2_form1_stage_2_cancers_10_posrisk_2_negrisk_5 | Patients | hypothetical |
| 0.240509 | 0.518117 | 0.783733 | pred_wait_1_detect_2_form1_stage_2_cancers_25_posrisk_2_negrisk_5 | Patients | hypothetical |
| 0.166163 | 0.394238 | 0.625924 | pred_wait_1_detect_2_form1_stage_2_cancers_1_posrisk_2_negrisk_10 | Patients | hypothetical |
| 0.169755 | 0.410138 | 0.652845 | pred_wait_1_detect_2_form1_stage_2_cancers_5_posrisk_2_negrisk_10 | Patients | hypothetical |
| 0.177252 | 0.432739 | 0.688226 | pred_wait_1_detect_2_form1_stage_2_cancers_10_posrisk_2_negrisk_10 | Patients | hypothetical |
| 0.228034 | 0.513809 | 0.782569 | pred_wait_1_detect_2_form1_stage_2_cancers_25_posrisk_2_negrisk_10 | Patients | hypothetical |
| 0.147697 | 0.375481 | 0.619559 | pred_wait_1_detect_2_form1_stage_2_cancers_1_posrisk_2_negrisk_40 | Patients | hypothetical |
| 0.150532 | 0.39012 | 0.646715 | pred_wait_1_detect_2_form1_stage_2_cancers_5_posrisk_2_negrisk_40 | Patients | hypothetical |
| 0.155941 | 0.411075 | 0.681777 | pred_wait_1_detect_2_form1_stage_2_cancers_10_posrisk_2_negrisk_40 | Patients | hypothetical |
| 0.198626 | 0.488042 | 0.774579 | pred_wait_1_detect_2_form1_stage_2_cancers_25_posrisk_2_negrisk_40 | Patients | hypothetical |
| 0.110715 | 0.343384 | 0.620335 | pred_wait_1_detect_2_form1_stage_2_cancers_1_posrisk_4_negrisk_1 | Patients | hypothetical |
| 0.111862 | 0.356254 | 0.647194 | pred_wait_1_detect_2_form1_stage_2_cancers_5_posrisk_4_negrisk_1 | Patients | hypothetical |
| 0.115167 | 0.374947 | 0.681878 | pred_wait_1_detect_2_form1_stage_2_cancers_10_posrisk_4_negrisk_1 | Patients | hypothetical |
| 0.147273 | 0.446215 | 0.774045 | pred_wait_1_detect_2_form1_stage_2_cancers_25_posrisk_4_negrisk_1 | Patients | hypothetical |
| 0.110715 | 0.33959 | 0.615487 | pred_wait_1_detect_2_form1_stage_2_cancers_1_posrisk_4_negrisk_5 | Patients | hypothetical |
| 0.111862 | 0.352387 | 0.643274 | pred_wait_1_detect_2_form1_stage_2_cancers_5_posrisk_4_negrisk_5 | Patients | hypothetical |
| 0.115167 | 0.371075 | 0.679174 | pred_wait_1_detect_2_form1_stage_2_cancers_10_posrisk_4_negrisk_5 | Patients | hypothetical |
| 0.147273 | 0.442972 | 0.774488 | pred_wait_1_detect_2_form1_stage_2_cancers_25_posrisk_4_negrisk_5 | Patients | hypothetical |
| 0.110715 | 0.335018 | 0.60839 | pred_wait_1_detect_2_form1_stage_2_cancers_1_posrisk_4_negrisk_10 | Patients | hypothetical |
| 0.111862 | 0.347676 | 0.636821 | pred_wait_1_detect_2_form1_stage_2_cancers_5_posrisk_4_negrisk_10 | Patients | hypothetical |
| 0.115167 | 0.366279 | 0.673792 | pred_wait_1_detect_2_form1_stage_2_cancers_10_posrisk_4_negrisk_10 | Patients | hypothetical |
| 0.147273 | 0.438664 | 0.772624 | pred_wait_1_detect_2_form1_stage_2_cancers_25_posrisk_4_negrisk_10 | Patients | hypothetical |
| 0.110618 | 0.316262 | 0.555857 | pred_wait_1_detect_2_form1_stage_2_cancers_1_posrisk_4_negrisk_40 | Patients | hypothetical |
| 0.111767 | 0.327657 | 0.579561 | pred_wait_1_detect_2_form1_stage_2_cancers_5_posrisk_4_negrisk_40 | Patients | hypothetical |
| 0.115153 | 0.344615 | 0.611993 | pred_wait_1_detect_2_form1_stage_2_cancers_10_posrisk_4_negrisk_40 | Patients | hypothetical |
| 0.147273 | 0.412897 | 0.707796 | pred_wait_1_detect_2_form1_stage_2_cancers_25_posrisk_4_negrisk_40 | Patients | hypothetical |
| 0.089891 | 0.31147 | 0.620335 | pred_wait_1_detect_2_form1_stage_2_cancers_1_posrisk_6_negrisk_1 | Patients | hypothetical |
| 0.091778 | 0.322679 | 0.647194 | pred_wait_1_detect_2_form1_stage_2_cancers_5_posrisk_6_negrisk_1 | Patients | hypothetical |
| 0.095358 | 0.338942 | 0.681878 | pred_wait_1_detect_2_form1_stage_2_cancers_10_posrisk_6_negrisk_1 | Patients | hypothetical |
| 0.120116 | 0.40078 | 0.774045 | pred_wait_1_detect_2_form1_stage_2_cancers_25_posrisk_6_negrisk_1 | Patients | hypothetical |
| 0.089891 | 0.307676 | 0.615487 | pred_wait_1_detect_2_form1_stage_2_cancers_1_posrisk_6_negrisk_5 | Patients | hypothetical |
| 0.091778 | 0.318812 | 0.643274 | pred_wait_1_detect_2_form1_stage_2_cancers_5_posrisk_6_negrisk_5 | Patients | hypothetical |
| 0.095358 | 0.335069 | 0.679174 | pred_wait_1_detect_2_form1_stage_2_cancers_10_posrisk_6_negrisk_5 | Patients | hypothetical |
| 0.120116 | 0.397537 | 0.774488 | pred_wait_1_detect_2_form1_stage_2_cancers_25_posrisk_6_negrisk_5 | Patients | hypothetical |
| 0.089891 | 0.303104 | 0.60839 | pred_wait_1_detect_2_form1_stage_2_cancers_1_posrisk_6_negrisk_10 | Patients | hypothetical |
| 0.091778 | 0.314101 | 0.636821 | pred_wait_1_detect_2_form1_stage_2_cancers_5_posrisk_6_negrisk_10 | Patients | hypothetical |
| 0.095358 | 0.330273 | 0.673792 | pred_wait_1_detect_2_form1_stage_2_cancers_10_posrisk_6_negrisk_10 | Patients | hypothetical |
| 0.120116 | 0.393229 | 0.772624 | pred_wait_1_detect_2_form1_stage_2_cancers_25_posrisk_6_negrisk_10 | Patients | hypothetical |
| 0.089891 | 0.284348 | 0.555857 | pred_wait_1_detect_2_form1_stage_2_cancers_1_posrisk_6_negrisk_40 | Patients | hypothetical |
| 0.091778 | 0.294083 | 0.579561 | pred_wait_1_detect_2_form1_stage_2_cancers_5_posrisk_6_negrisk_40 | Patients | hypothetical |
| 0.095358 | 0.308609 | 0.611993 | pred_wait_1_detect_2_form1_stage_2_cancers_10_posrisk_6_negrisk_40 | Patients | hypothetical |
| 0.120116 | 0.367462 | 0.707796 | pred_wait_1_detect_2_form1_stage_2_cancers_25_posrisk_6_negrisk_40 | Patients | hypothetical |
| 0.089238 | 0.297922 | 0.620335 | pred_wait_1_detect_2_form1_stage_2_cancers_1_posrisk_8_negrisk_1 | Patients | hypothetical |
| 0.091829 | 0.308532 | 0.647194 | pred_wait_1_detect_2_form1_stage_2_cancers_5_posrisk_8_negrisk_1 | Patients | hypothetical |
| 0.095875 | 0.323731 | 0.681878 | pred_wait_1_detect_2_form1_stage_2_cancers_10_posrisk_8_negrisk_1 | Patients | hypothetical |
| 0.117168 | 0.379898 | 0.774045 | pred_wait_1_detect_2_form1_stage_2_cancers_25_posrisk_8_negrisk_1 | Patients | hypothetical |
| 0.089238 | 0.294128 | 0.615487 | pred_wait_1_detect_2_form1_stage_2_cancers_1_posrisk_8_negrisk_5 | Patients | hypothetical |
| 0.091829 | 0.304665 | 0.643274 | pred_wait_1_detect_2_form1_stage_2_cancers_5_posrisk_8_negrisk_5 | Patients | hypothetical |
| 0.095875 | 0.319858 | 0.679174 | pred_wait_1_detect_2_form1_stage_2_cancers_10_posrisk_8_negrisk_5 | Patients | hypothetical |
| 0.117168 | 0.376655 | 0.774488 | pred_wait_1_detect_2_form1_stage_2_cancers_25_posrisk_8_negrisk_5 | Patients | hypothetical |
| 0.089238 | 0.289556 | 0.60839 | pred_wait_1_detect_2_form1_stage_2_cancers_1_posrisk_8_negrisk_10 | Patients | hypothetical |
| 0.091829 | 0.299954 | 0.636821 | pred_wait_1_detect_2_form1_stage_2_cancers_5_posrisk_8_negrisk_10 | Patients | hypothetical |
| 0.095875 | 0.315062 | 0.673792 | pred_wait_1_detect_2_form1_stage_2_cancers_10_posrisk_8_negrisk_10 | Patients | hypothetical |
| 0.117168 | 0.372347 | 0.772624 | pred_wait_1_detect_2_form1_stage_2_cancers_25_posrisk_8_negrisk_10 | Patients | hypothetical |
| 0.089238 | 0.2708 | 0.555857 | pred_wait_1_detect_2_form1_stage_2_cancers_1_posrisk_8_negrisk_40 | Patients | hypothetical |
| 0.091829 | 0.279935 | 0.579561 | pred_wait_1_detect_2_form1_stage_2_cancers_5_posrisk_8_negrisk_40 | Patients | hypothetical |
| 0.095875 | 0.293398 | 0.611993 | pred_wait_1_detect_2_form1_stage_2_cancers_10_posrisk_8_negrisk_40 | Patients | hypothetical |
| 0.117168 | 0.34658 | 0.707796 | pred_wait_1_detect_2_form1_stage_2_cancers_25_posrisk_8_negrisk_40 | Patients | hypothetical |
| 0.177685 | 0.402604 | 0.630238 | pred_wait_1_detect_2_form2_stage_1_cancers_1_posrisk_2_negrisk_1 | Patients | hypothetical |
| 0.182846 | 0.418716 | 0.657271 | pred_wait_1_detect_2_form2_stage_1_cancers_5_posrisk_2_negrisk_1 | Patients | hypothetical |
| 0.191994 | 0.441408 | 0.692158 | pred_wait_1_detect_2_form2_stage_1_cancers_10_posrisk_2_negrisk_1 | Patients | hypothetical |
| 0.246904 | 0.52136 | 0.783577 | pred_wait_1_detect_2_form2_stage_1_cancers_25_posrisk_2_negrisk_1 | Patients | hypothetical |
| 0.172999 | 0.39881 | 0.628372 | pred_wait_1_detect_2_form2_stage_1_cancers_1_posrisk_2_negrisk_5 | Patients | hypothetical |
| 0.177665 | 0.414849 | 0.655619 | pred_wait_1_detect_2_form2_stage_1_cancers_5_posrisk_2_negrisk_5 | Patients | hypothetical |
| 0.186554 | 0.437535 | 0.690041 | pred_wait_1_detect_2_form2_stage_1_cancers_10_posrisk_2_negrisk_5 | Patients | hypothetical |
| 0.240509 | 0.518117 | 0.783733 | pred_wait_1_detect_2_form2_stage_1_cancers_25_posrisk_2_negrisk_5 | Patients | hypothetical |
| 0.166163 | 0.394238 | 0.625924 | pred_wait_1_detect_2_form2_stage_1_cancers_1_posrisk_2_negrisk_10 | Patients | hypothetical |
| 0.169755 | 0.410138 | 0.652845 | pred_wait_1_detect_2_form2_stage_1_cancers_5_posrisk_2_negrisk_10 | Patients | hypothetical |
| 0.177252 | 0.432739 | 0.688226 | pred_wait_1_detect_2_form2_stage_1_cancers_10_posrisk_2_negrisk_10 | Patients | hypothetical |
| 0.228034 | 0.513809 | 0.782569 | pred_wait_1_detect_2_form2_stage_1_cancers_25_posrisk_2_negrisk_10 | Patients | hypothetical |
| 0.147697 | 0.375481 | 0.619559 | pred_wait_1_detect_2_form2_stage_1_cancers_1_posrisk_2_negrisk_40 | Patients | hypothetical |
| 0.150532 | 0.39012 | 0.646715 | pred_wait_1_detect_2_form2_stage_1_cancers_5_posrisk_2_negrisk_40 | Patients | hypothetical |
| 0.155941 | 0.411075 | 0.681777 | pred_wait_1_detect_2_form2_stage_1_cancers_10_posrisk_2_negrisk_40 | Patients | hypothetical |
| 0.198626 | 0.488042 | 0.774579 | pred_wait_1_detect_2_form2_stage_1_cancers_25_posrisk_2_negrisk_40 | Patients | hypothetical |
| 0.110715 | 0.343384 | 0.620335 | pred_wait_1_detect_2_form2_stage_1_cancers_1_posrisk_4_negrisk_1 | Patients | hypothetical |
| 0.111862 | 0.356254 | 0.647194 | pred_wait_1_detect_2_form2_stage_1_cancers_5_posrisk_4_negrisk_1 | Patients | hypothetical |
| 0.115167 | 0.374947 | 0.681878 | pred_wait_1_detect_2_form2_stage_1_cancers_10_posrisk_4_negrisk_1 | Patients | hypothetical |
| 0.147273 | 0.446215 | 0.774045 | pred_wait_1_detect_2_form2_stage_1_cancers_25_posrisk_4_negrisk_1 | Patients | hypothetical |
| 0.110715 | 0.33959 | 0.615487 | pred_wait_1_detect_2_form2_stage_1_cancers_1_posrisk_4_negrisk_5 | Patients | hypothetical |
| 0.111862 | 0.352387 | 0.643274 | pred_wait_1_detect_2_form2_stage_1_cancers_5_posrisk_4_negrisk_5 | Patients | hypothetical |
| 0.115167 | 0.371075 | 0.679174 | pred_wait_1_detect_2_form2_stage_1_cancers_10_posrisk_4_negrisk_5 | Patients | hypothetical |
| 0.147273 | 0.442972 | 0.774488 | pred_wait_1_detect_2_form2_stage_1_cancers_25_posrisk_4_negrisk_5 | Patients | hypothetical |
| 0.110715 | 0.335018 | 0.60839 | pred_wait_1_detect_2_form2_stage_1_cancers_1_posrisk_4_negrisk_10 | Patients | hypothetical |
| 0.111862 | 0.347676 | 0.636821 | pred_wait_1_detect_2_form2_stage_1_cancers_5_posrisk_4_negrisk_10 | Patients | hypothetical |
| 0.115167 | 0.366279 | 0.673792 | pred_wait_1_detect_2_form2_stage_1_cancers_10_posrisk_4_negrisk_10 | Patients | hypothetical |
| 0.147273 | 0.438664 | 0.772624 | pred_wait_1_detect_2_form2_stage_1_cancers_25_posrisk_4_negrisk_10 | Patients | hypothetical |
| 0.110618 | 0.316262 | 0.555857 | pred_wait_1_detect_2_form2_stage_1_cancers_1_posrisk_4_negrisk_40 | Patients | hypothetical |
| 0.111767 | 0.327657 | 0.579561 | pred_wait_1_detect_2_form2_stage_1_cancers_5_posrisk_4_negrisk_40 | Patients | hypothetical |
| 0.115153 | 0.344615 | 0.611993 | pred_wait_1_detect_2_form2_stage_1_cancers_10_posrisk_4_negrisk_40 | Patients | hypothetical |
| 0.147273 | 0.412897 | 0.707796 | pred_wait_1_detect_2_form2_stage_1_cancers_25_posrisk_4_negrisk_40 | Patients | hypothetical |
| 0.089891 | 0.31147 | 0.620335 | pred_wait_1_detect_2_form2_stage_1_cancers_1_posrisk_6_negrisk_1 | Patients | hypothetical |
| 0.091778 | 0.322679 | 0.647194 | pred_wait_1_detect_2_form2_stage_1_cancers_5_posrisk_6_negrisk_1 | Patients | hypothetical |
| 0.095358 | 0.338942 | 0.681878 | pred_wait_1_detect_2_form2_stage_1_cancers_10_posrisk_6_negrisk_1 | Patients | hypothetical |
| 0.120116 | 0.40078 | 0.774045 | pred_wait_1_detect_2_form2_stage_1_cancers_25_posrisk_6_negrisk_1 | Patients | hypothetical |
| 0.089891 | 0.307676 | 0.615487 | pred_wait_1_detect_2_form2_stage_1_cancers_1_posrisk_6_negrisk_5 | Patients | hypothetical |
| 0.091778 | 0.318812 | 0.643274 | pred_wait_1_detect_2_form2_stage_1_cancers_5_posrisk_6_negrisk_5 | Patients | hypothetical |
| 0.095358 | 0.335069 | 0.679174 | pred_wait_1_detect_2_form2_stage_1_cancers_10_posrisk_6_negrisk_5 | Patients | hypothetical |
| 0.120116 | 0.397537 | 0.774488 | pred_wait_1_detect_2_form2_stage_1_cancers_25_posrisk_6_negrisk_5 | Patients | hypothetical |
| 0.089891 | 0.303104 | 0.60839 | pred_wait_1_detect_2_form2_stage_1_cancers_1_posrisk_6_negrisk_10 | Patients | hypothetical |
| 0.091778 | 0.314101 | 0.636821 | pred_wait_1_detect_2_form2_stage_1_cancers_5_posrisk_6_negrisk_10 | Patients | hypothetical |
| 0.095358 | 0.330273 | 0.673792 | pred_wait_1_detect_2_form2_stage_1_cancers_10_posrisk_6_negrisk_10 | Patients | hypothetical |
| 0.120116 | 0.393229 | 0.772624 | pred_wait_1_detect_2_form2_stage_1_cancers_25_posrisk_6_negrisk_10 | Patients | hypothetical |
| 0.089891 | 0.284348 | 0.555857 | pred_wait_1_detect_2_form2_stage_1_cancers_1_posrisk_6_negrisk_40 | Patients | hypothetical |
| 0.091778 | 0.294083 | 0.579561 | pred_wait_1_detect_2_form2_stage_1_cancers_5_posrisk_6_negrisk_40 | Patients | hypothetical |
| 0.095358 | 0.308609 | 0.611993 | pred_wait_1_detect_2_form2_stage_1_cancers_10_posrisk_6_negrisk_40 | Patients | hypothetical |
| 0.120116 | 0.367462 | 0.707796 | pred_wait_1_detect_2_form2_stage_1_cancers_25_posrisk_6_negrisk_40 | Patients | hypothetical |
| 0.089238 | 0.297922 | 0.620335 | pred_wait_1_detect_2_form2_stage_1_cancers_1_posrisk_8_negrisk_1 | Patients | hypothetical |
| 0.091829 | 0.308532 | 0.647194 | pred_wait_1_detect_2_form2_stage_1_cancers_5_posrisk_8_negrisk_1 | Patients | hypothetical |
| 0.095875 | 0.323731 | 0.681878 | pred_wait_1_detect_2_form2_stage_1_cancers_10_posrisk_8_negrisk_1 | Patients | hypothetical |
| 0.117168 | 0.379898 | 0.774045 | pred_wait_1_detect_2_form2_stage_1_cancers_25_posrisk_8_negrisk_1 | Patients | hypothetical |
| 0.089238 | 0.294128 | 0.615487 | pred_wait_1_detect_2_form2_stage_1_cancers_1_posrisk_8_negrisk_5 | Patients | hypothetical |
| 0.091829 | 0.304665 | 0.643274 | pred_wait_1_detect_2_form2_stage_1_cancers_5_posrisk_8_negrisk_5 | Patients | hypothetical |
| 0.095875 | 0.319858 | 0.679174 | pred_wait_1_detect_2_form2_stage_1_cancers_10_posrisk_8_negrisk_5 | Patients | hypothetical |
| 0.117168 | 0.376655 | 0.774488 | pred_wait_1_detect_2_form2_stage_1_cancers_25_posrisk_8_negrisk_5 | Patients | hypothetical |
| 0.089238 | 0.289556 | 0.60839 | pred_wait_1_detect_2_form2_stage_1_cancers_1_posrisk_8_negrisk_10 | Patients | hypothetical |
| 0.091829 | 0.299954 | 0.636821 | pred_wait_1_detect_2_form2_stage_1_cancers_5_posrisk_8_negrisk_10 | Patients | hypothetical |
| 0.095875 | 0.315062 | 0.673792 | pred_wait_1_detect_2_form2_stage_1_cancers_10_posrisk_8_negrisk_10 | Patients | hypothetical |
| 0.117168 | 0.372347 | 0.772624 | pred_wait_1_detect_2_form2_stage_1_cancers_25_posrisk_8_negrisk_10 | Patients | hypothetical |
| 0.089238 | 0.2708 | 0.555857 | pred_wait_1_detect_2_form2_stage_1_cancers_1_posrisk_8_negrisk_40 | Patients | hypothetical |
| 0.091829 | 0.279935 | 0.579561 | pred_wait_1_detect_2_form2_stage_1_cancers_5_posrisk_8_negrisk_40 | Patients | hypothetical |
| 0.095875 | 0.293398 | 0.611993 | pred_wait_1_detect_2_form2_stage_1_cancers_10_posrisk_8_negrisk_40 | Patients | hypothetical |
| 0.117168 | 0.34658 | 0.707796 | pred_wait_1_detect_2_form2_stage_1_cancers_25_posrisk_8_negrisk_40 | Patients | hypothetical |
| 0.177685 | 0.402604 | 0.630238 | pred_wait_1_detect_2_form2_stage_2_cancers_1_posrisk_2_negrisk_1 | Patients | hypothetical |
| 0.182846 | 0.418716 | 0.657271 | pred_wait_1_detect_2_form2_stage_2_cancers_5_posrisk_2_negrisk_1 | Patients | hypothetical |
| 0.191994 | 0.441408 | 0.692158 | pred_wait_1_detect_2_form2_stage_2_cancers_10_posrisk_2_negrisk_1 | Patients | hypothetical |
| 0.246904 | 0.52136 | 0.783577 | pred_wait_1_detect_2_form2_stage_2_cancers_25_posrisk_2_negrisk_1 | Patients | hypothetical |
| 0.172999 | 0.39881 | 0.628372 | pred_wait_1_detect_2_form2_stage_2_cancers_1_posrisk_2_negrisk_5 | Patients | hypothetical |
| 0.177665 | 0.414849 | 0.655619 | pred_wait_1_detect_2_form2_stage_2_cancers_5_posrisk_2_negrisk_5 | Patients | hypothetical |
| 0.186554 | 0.437535 | 0.690041 | pred_wait_1_detect_2_form2_stage_2_cancers_10_posrisk_2_negrisk_5 | Patients | hypothetical |
| 0.240509 | 0.518117 | 0.783733 | pred_wait_1_detect_2_form2_stage_2_cancers_25_posrisk_2_negrisk_5 | Patients | hypothetical |
| 0.166163 | 0.394238 | 0.625924 | pred_wait_1_detect_2_form2_stage_2_cancers_1_posrisk_2_negrisk_10 | Patients | hypothetical |
| 0.169755 | 0.410138 | 0.652845 | pred_wait_1_detect_2_form2_stage_2_cancers_5_posrisk_2_negrisk_10 | Patients | hypothetical |
| 0.177252 | 0.432739 | 0.688226 | pred_wait_1_detect_2_form2_stage_2_cancers_10_posrisk_2_negrisk_10 | Patients | hypothetical |
| 0.228034 | 0.513809 | 0.782569 | pred_wait_1_detect_2_form2_stage_2_cancers_25_posrisk_2_negrisk_10 | Patients | hypothetical |
| 0.147697 | 0.375481 | 0.619559 | pred_wait_1_detect_2_form2_stage_2_cancers_1_posrisk_2_negrisk_40 | Patients | hypothetical |
| 0.150532 | 0.39012 | 0.646715 | pred_wait_1_detect_2_form2_stage_2_cancers_5_posrisk_2_negrisk_40 | Patients | hypothetical |
| 0.155941 | 0.411075 | 0.681777 | pred_wait_1_detect_2_form2_stage_2_cancers_10_posrisk_2_negrisk_40 | Patients | hypothetical |
| 0.198626 | 0.488042 | 0.774579 | pred_wait_1_detect_2_form2_stage_2_cancers_25_posrisk_2_negrisk_40 | Patients | hypothetical |
| 0.110715 | 0.343384 | 0.620335 | pred_wait_1_detect_2_form2_stage_2_cancers_1_posrisk_4_negrisk_1 | Patients | hypothetical |
| 0.111862 | 0.356254 | 0.647194 | pred_wait_1_detect_2_form2_stage_2_cancers_5_posrisk_4_negrisk_1 | Patients | hypothetical |
| 0.115167 | 0.374947 | 0.681878 | pred_wait_1_detect_2_form2_stage_2_cancers_10_posrisk_4_negrisk_1 | Patients | hypothetical |
| 0.147273 | 0.446215 | 0.774045 | pred_wait_1_detect_2_form2_stage_2_cancers_25_posrisk_4_negrisk_1 | Patients | hypothetical |
| 0.110715 | 0.33959 | 0.615487 | pred_wait_1_detect_2_form2_stage_2_cancers_1_posrisk_4_negrisk_5 | Patients | hypothetical |
| 0.111862 | 0.352387 | 0.643274 | pred_wait_1_detect_2_form2_stage_2_cancers_5_posrisk_4_negrisk_5 | Patients | hypothetical |
| 0.115167 | 0.371075 | 0.679174 | pred_wait_1_detect_2_form2_stage_2_cancers_10_posrisk_4_negrisk_5 | Patients | hypothetical |
| 0.147273 | 0.442972 | 0.774488 | pred_wait_1_detect_2_form2_stage_2_cancers_25_posrisk_4_negrisk_5 | Patients | hypothetical |
| 0.110715 | 0.335018 | 0.60839 | pred_wait_1_detect_2_form2_stage_2_cancers_1_posrisk_4_negrisk_10 | Patients | hypothetical |
| 0.111862 | 0.347676 | 0.636821 | pred_wait_1_detect_2_form2_stage_2_cancers_5_posrisk_4_negrisk_10 | Patients | hypothetical |
| 0.115167 | 0.366279 | 0.673792 | pred_wait_1_detect_2_form2_stage_2_cancers_10_posrisk_4_negrisk_10 | Patients | hypothetical |
| 0.147273 | 0.438664 | 0.772624 | pred_wait_1_detect_2_form2_stage_2_cancers_25_posrisk_4_negrisk_10 | Patients | hypothetical |
| 0.110618 | 0.316262 | 0.555857 | pred_wait_1_detect_2_form2_stage_2_cancers_1_posrisk_4_negrisk_40 | Patients | hypothetical |
| 0.111767 | 0.327657 | 0.579561 | pred_wait_1_detect_2_form2_stage_2_cancers_5_posrisk_4_negrisk_40 | Patients | hypothetical |
| 0.115153 | 0.344615 | 0.611993 | pred_wait_1_detect_2_form2_stage_2_cancers_10_posrisk_4_negrisk_40 | Patients | hypothetical |
| 0.147273 | 0.412897 | 0.707796 | pred_wait_1_detect_2_form2_stage_2_cancers_25_posrisk_4_negrisk_40 | Patients | hypothetical |
| 0.089891 | 0.31147 | 0.620335 | pred_wait_1_detect_2_form2_stage_2_cancers_1_posrisk_6_negrisk_1 | Patients | hypothetical |
| 0.091778 | 0.322679 | 0.647194 | pred_wait_1_detect_2_form2_stage_2_cancers_5_posrisk_6_negrisk_1 | Patients | hypothetical |
| 0.095358 | 0.338942 | 0.681878 | pred_wait_1_detect_2_form2_stage_2_cancers_10_posrisk_6_negrisk_1 | Patients | hypothetical |
| 0.120116 | 0.40078 | 0.774045 | pred_wait_1_detect_2_form2_stage_2_cancers_25_posrisk_6_negrisk_1 | Patients | hypothetical |
| 0.089891 | 0.307676 | 0.615487 | pred_wait_1_detect_2_form2_stage_2_cancers_1_posrisk_6_negrisk_5 | Patients | hypothetical |
| 0.091778 | 0.318812 | 0.643274 | pred_wait_1_detect_2_form2_stage_2_cancers_5_posrisk_6_negrisk_5 | Patients | hypothetical |
| 0.095358 | 0.335069 | 0.679174 | pred_wait_1_detect_2_form2_stage_2_cancers_10_posrisk_6_negrisk_5 | Patients | hypothetical |
| 0.120116 | 0.397537 | 0.774488 | pred_wait_1_detect_2_form2_stage_2_cancers_25_posrisk_6_negrisk_5 | Patients | hypothetical |
| 0.089891 | 0.303104 | 0.60839 | pred_wait_1_detect_2_form2_stage_2_cancers_1_posrisk_6_negrisk_10 | Patients | hypothetical |
| 0.091778 | 0.314101 | 0.636821 | pred_wait_1_detect_2_form2_stage_2_cancers_5_posrisk_6_negrisk_10 | Patients | hypothetical |
| 0.095358 | 0.330273 | 0.673792 | pred_wait_1_detect_2_form2_stage_2_cancers_10_posrisk_6_negrisk_10 | Patients | hypothetical |
| 0.120116 | 0.393229 | 0.772624 | pred_wait_1_detect_2_form2_stage_2_cancers_25_posrisk_6_negrisk_10 | Patients | hypothetical |
| 0.089891 | 0.284348 | 0.555857 | pred_wait_1_detect_2_form2_stage_2_cancers_1_posrisk_6_negrisk_40 | Patients | hypothetical |
| 0.091778 | 0.294083 | 0.579561 | pred_wait_1_detect_2_form2_stage_2_cancers_5_posrisk_6_negrisk_40 | Patients | hypothetical |
| 0.095358 | 0.308609 | 0.611993 | pred_wait_1_detect_2_form2_stage_2_cancers_10_posrisk_6_negrisk_40 | Patients | hypothetical |
| 0.120116 | 0.367462 | 0.707796 | pred_wait_1_detect_2_form2_stage_2_cancers_25_posrisk_6_negrisk_40 | Patients | hypothetical |
| 0.089238 | 0.297922 | 0.620335 | pred_wait_1_detect_2_form2_stage_2_cancers_1_posrisk_8_negrisk_1 | Patients | hypothetical |
| 0.091829 | 0.308532 | 0.647194 | pred_wait_1_detect_2_form2_stage_2_cancers_5_posrisk_8_negrisk_1 | Patients | hypothetical |
| 0.095875 | 0.323731 | 0.681878 | pred_wait_1_detect_2_form2_stage_2_cancers_10_posrisk_8_negrisk_1 | Patients | hypothetical |
| 0.117168 | 0.379898 | 0.774045 | pred_wait_1_detect_2_form2_stage_2_cancers_25_posrisk_8_negrisk_1 | Patients | hypothetical |
| 0.089238 | 0.294128 | 0.615487 | pred_wait_1_detect_2_form2_stage_2_cancers_1_posrisk_8_negrisk_5 | Patients | hypothetical |
| 0.091829 | 0.304665 | 0.643274 | pred_wait_1_detect_2_form2_stage_2_cancers_5_posrisk_8_negrisk_5 | Patients | hypothetical |
| 0.095875 | 0.319858 | 0.679174 | pred_wait_1_detect_2_form2_stage_2_cancers_10_posrisk_8_negrisk_5 | Patients | hypothetical |
| 0.117168 | 0.376655 | 0.774488 | pred_wait_1_detect_2_form2_stage_2_cancers_25_posrisk_8_negrisk_5 | Patients | hypothetical |
| 0.089238 | 0.289556 | 0.60839 | pred_wait_1_detect_2_form2_stage_2_cancers_1_posrisk_8_negrisk_10 | Patients | hypothetical |
| 0.091829 | 0.299954 | 0.636821 | pred_wait_1_detect_2_form2_stage_2_cancers_5_posrisk_8_negrisk_10 | Patients | hypothetical |
| 0.095875 | 0.315062 | 0.673792 | pred_wait_1_detect_2_form2_stage_2_cancers_10_posrisk_8_negrisk_10 | Patients | hypothetical |
| 0.117168 | 0.372347 | 0.772624 | pred_wait_1_detect_2_form2_stage_2_cancers_25_posrisk_8_negrisk_10 | Patients | hypothetical |
| 0.089238 | 0.2708 | 0.555857 | pred_wait_1_detect_2_form2_stage_2_cancers_1_posrisk_8_negrisk_40 | Patients | hypothetical |
| 0.091829 | 0.279935 | 0.579561 | pred_wait_1_detect_2_form2_stage_2_cancers_5_posrisk_8_negrisk_40 | Patients | hypothetical |
| 0.095875 | 0.293398 | 0.611993 | pred_wait_1_detect_2_form2_stage_2_cancers_10_posrisk_8_negrisk_40 | Patients | hypothetical |
| 0.117168 | 0.34658 | 0.707796 | pred_wait_1_detect_2_form2_stage_2_cancers_25_posrisk_8_negrisk_40 | Patients | hypothetical |
| 0.177685 | 0.402604 | 0.630238 | pred_wait_1_detect_2_form3_stage_1_cancers_1_posrisk_2_negrisk_1 | Patients | hypothetical |
| 0.182846 | 0.418716 | 0.657271 | pred_wait_1_detect_2_form3_stage_1_cancers_5_posrisk_2_negrisk_1 | Patients | hypothetical |
| 0.191994 | 0.441408 | 0.692158 | pred_wait_1_detect_2_form3_stage_1_cancers_10_posrisk_2_negrisk_1 | Patients | hypothetical |
| 0.246904 | 0.52136 | 0.783577 | pred_wait_1_detect_2_form3_stage_1_cancers_25_posrisk_2_negrisk_1 | Patients | hypothetical |
| 0.172999 | 0.39881 | 0.628372 | pred_wait_1_detect_2_form3_stage_1_cancers_1_posrisk_2_negrisk_5 | Patients | hypothetical |
| 0.177665 | 0.414849 | 0.655619 | pred_wait_1_detect_2_form3_stage_1_cancers_5_posrisk_2_negrisk_5 | Patients | hypothetical |
| 0.186554 | 0.437535 | 0.690041 | pred_wait_1_detect_2_form3_stage_1_cancers_10_posrisk_2_negrisk_5 | Patients | hypothetical |
| 0.240509 | 0.518117 | 0.783733 | pred_wait_1_detect_2_form3_stage_1_cancers_25_posrisk_2_negrisk_5 | Patients | hypothetical |
| 0.166163 | 0.394238 | 0.625924 | pred_wait_1_detect_2_form3_stage_1_cancers_1_posrisk_2_negrisk_10 | Patients | hypothetical |
| 0.169755 | 0.410138 | 0.652845 | pred_wait_1_detect_2_form3_stage_1_cancers_5_posrisk_2_negrisk_10 | Patients | hypothetical |
| 0.177252 | 0.432739 | 0.688226 | pred_wait_1_detect_2_form3_stage_1_cancers_10_posrisk_2_negrisk_10 | Patients | hypothetical |
| 0.228034 | 0.513809 | 0.782569 | pred_wait_1_detect_2_form3_stage_1_cancers_25_posrisk_2_negrisk_10 | Patients | hypothetical |
| 0.147697 | 0.375481 | 0.619559 | pred_wait_1_detect_2_form3_stage_1_cancers_1_posrisk_2_negrisk_40 | Patients | hypothetical |
| 0.150532 | 0.39012 | 0.646715 | pred_wait_1_detect_2_form3_stage_1_cancers_5_posrisk_2_negrisk_40 | Patients | hypothetical |
| 0.155941 | 0.411075 | 0.681777 | pred_wait_1_detect_2_form3_stage_1_cancers_10_posrisk_2_negrisk_40 | Patients | hypothetical |
| 0.198626 | 0.488042 | 0.774579 | pred_wait_1_detect_2_form3_stage_1_cancers_25_posrisk_2_negrisk_40 | Patients | hypothetical |
| 0.110715 | 0.343384 | 0.620335 | pred_wait_1_detect_2_form3_stage_1_cancers_1_posrisk_4_negrisk_1 | Patients | hypothetical |
| 0.111862 | 0.356254 | 0.647194 | pred_wait_1_detect_2_form3_stage_1_cancers_5_posrisk_4_negrisk_1 | Patients | hypothetical |
| 0.115167 | 0.374947 | 0.681878 | pred_wait_1_detect_2_form3_stage_1_cancers_10_posrisk_4_negrisk_1 | Patients | hypothetical |
| 0.147273 | 0.446215 | 0.774045 | pred_wait_1_detect_2_form3_stage_1_cancers_25_posrisk_4_negrisk_1 | Patients | hypothetical |
| 0.110715 | 0.33959 | 0.615487 | pred_wait_1_detect_2_form3_stage_1_cancers_1_posrisk_4_negrisk_5 | Patients | hypothetical |
| 0.111862 | 0.352387 | 0.643274 | pred_wait_1_detect_2_form3_stage_1_cancers_5_posrisk_4_negrisk_5 | Patients | hypothetical |
| 0.115167 | 0.371075 | 0.679174 | pred_wait_1_detect_2_form3_stage_1_cancers_10_posrisk_4_negrisk_5 | Patients | hypothetical |
| 0.147273 | 0.442972 | 0.774488 | pred_wait_1_detect_2_form3_stage_1_cancers_25_posrisk_4_negrisk_5 | Patients | hypothetical |
| 0.110715 | 0.335018 | 0.60839 | pred_wait_1_detect_2_form3_stage_1_cancers_1_posrisk_4_negrisk_10 | Patients | hypothetical |
| 0.111862 | 0.347676 | 0.636821 | pred_wait_1_detect_2_form3_stage_1_cancers_5_posrisk_4_negrisk_10 | Patients | hypothetical |
| 0.115167 | 0.366279 | 0.673792 | pred_wait_1_detect_2_form3_stage_1_cancers_10_posrisk_4_negrisk_10 | Patients | hypothetical |
| 0.147273 | 0.438664 | 0.772624 | pred_wait_1_detect_2_form3_stage_1_cancers_25_posrisk_4_negrisk_10 | Patients | hypothetical |
| 0.110618 | 0.316262 | 0.555857 | pred_wait_1_detect_2_form3_stage_1_cancers_1_posrisk_4_negrisk_40 | Patients | hypothetical |
| 0.111767 | 0.327657 | 0.579561 | pred_wait_1_detect_2_form3_stage_1_cancers_5_posrisk_4_negrisk_40 | Patients | hypothetical |
| 0.115153 | 0.344615 | 0.611993 | pred_wait_1_detect_2_form3_stage_1_cancers_10_posrisk_4_negrisk_40 | Patients | hypothetical |
| 0.147273 | 0.412897 | 0.707796 | pred_wait_1_detect_2_form3_stage_1_cancers_25_posrisk_4_negrisk_40 | Patients | hypothetical |
| 0.089891 | 0.31147 | 0.620335 | pred_wait_1_detect_2_form3_stage_1_cancers_1_posrisk_6_negrisk_1 | Patients | hypothetical |
| 0.091778 | 0.322679 | 0.647194 | pred_wait_1_detect_2_form3_stage_1_cancers_5_posrisk_6_negrisk_1 | Patients | hypothetical |
| 0.095358 | 0.338942 | 0.681878 | pred_wait_1_detect_2_form3_stage_1_cancers_10_posrisk_6_negrisk_1 | Patients | hypothetical |
| 0.120116 | 0.40078 | 0.774045 | pred_wait_1_detect_2_form3_stage_1_cancers_25_posrisk_6_negrisk_1 | Patients | hypothetical |
| 0.089891 | 0.307676 | 0.615487 | pred_wait_1_detect_2_form3_stage_1_cancers_1_posrisk_6_negrisk_5 | Patients | hypothetical |
| 0.091778 | 0.318812 | 0.643274 | pred_wait_1_detect_2_form3_stage_1_cancers_5_posrisk_6_negrisk_5 | Patients | hypothetical |
| 0.095358 | 0.335069 | 0.679174 | pred_wait_1_detect_2_form3_stage_1_cancers_10_posrisk_6_negrisk_5 | Patients | hypothetical |
| 0.120116 | 0.397537 | 0.774488 | pred_wait_1_detect_2_form3_stage_1_cancers_25_posrisk_6_negrisk_5 | Patients | hypothetical |
| 0.089891 | 0.303104 | 0.60839 | pred_wait_1_detect_2_form3_stage_1_cancers_1_posrisk_6_negrisk_10 | Patients | hypothetical |
| 0.091778 | 0.314101 | 0.636821 | pred_wait_1_detect_2_form3_stage_1_cancers_5_posrisk_6_negrisk_10 | Patients | hypothetical |
| 0.095358 | 0.330273 | 0.673792 | pred_wait_1_detect_2_form3_stage_1_cancers_10_posrisk_6_negrisk_10 | Patients | hypothetical |
| 0.120116 | 0.393229 | 0.772624 | pred_wait_1_detect_2_form3_stage_1_cancers_25_posrisk_6_negrisk_10 | Patients | hypothetical |
| 0.089891 | 0.284348 | 0.555857 | pred_wait_1_detect_2_form3_stage_1_cancers_1_posrisk_6_negrisk_40 | Patients | hypothetical |
| 0.091778 | 0.294083 | 0.579561 | pred_wait_1_detect_2_form3_stage_1_cancers_5_posrisk_6_negrisk_40 | Patients | hypothetical |
| 0.095358 | 0.308609 | 0.611993 | pred_wait_1_detect_2_form3_stage_1_cancers_10_posrisk_6_negrisk_40 | Patients | hypothetical |
| 0.120116 | 0.367462 | 0.707796 | pred_wait_1_detect_2_form3_stage_1_cancers_25_posrisk_6_negrisk_40 | Patients | hypothetical |
| 0.089238 | 0.297922 | 0.620335 | pred_wait_1_detect_2_form3_stage_1_cancers_1_posrisk_8_negrisk_1 | Patients | hypothetical |
| 0.091829 | 0.308532 | 0.647194 | pred_wait_1_detect_2_form3_stage_1_cancers_5_posrisk_8_negrisk_1 | Patients | hypothetical |
| 0.095875 | 0.323731 | 0.681878 | pred_wait_1_detect_2_form3_stage_1_cancers_10_posrisk_8_negrisk_1 | Patients | hypothetical |
| 0.117168 | 0.379898 | 0.774045 | pred_wait_1_detect_2_form3_stage_1_cancers_25_posrisk_8_negrisk_1 | Patients | hypothetical |
| 0.089238 | 0.294128 | 0.615487 | pred_wait_1_detect_2_form3_stage_1_cancers_1_posrisk_8_negrisk_5 | Patients | hypothetical |
| 0.091829 | 0.304665 | 0.643274 | pred_wait_1_detect_2_form3_stage_1_cancers_5_posrisk_8_negrisk_5 | Patients | hypothetical |
| 0.095875 | 0.319858 | 0.679174 | pred_wait_1_detect_2_form3_stage_1_cancers_10_posrisk_8_negrisk_5 | Patients | hypothetical |
| 0.117168 | 0.376655 | 0.774488 | pred_wait_1_detect_2_form3_stage_1_cancers_25_posrisk_8_negrisk_5 | Patients | hypothetical |
| 0.089238 | 0.289556 | 0.60839 | pred_wait_1_detect_2_form3_stage_1_cancers_1_posrisk_8_negrisk_10 | Patients | hypothetical |
| 0.091829 | 0.299954 | 0.636821 | pred_wait_1_detect_2_form3_stage_1_cancers_5_posrisk_8_negrisk_10 | Patients | hypothetical |
| 0.095875 | 0.315062 | 0.673792 | pred_wait_1_detect_2_form3_stage_1_cancers_10_posrisk_8_negrisk_10 | Patients | hypothetical |
| 0.117168 | 0.372347 | 0.772624 | pred_wait_1_detect_2_form3_stage_1_cancers_25_posrisk_8_negrisk_10 | Patients | hypothetical |
| 0.089238 | 0.2708 | 0.555857 | pred_wait_1_detect_2_form3_stage_1_cancers_1_posrisk_8_negrisk_40 | Patients | hypothetical |
| 0.091829 | 0.279935 | 0.579561 | pred_wait_1_detect_2_form3_stage_1_cancers_5_posrisk_8_negrisk_40 | Patients | hypothetical |
| 0.095875 | 0.293398 | 0.611993 | pred_wait_1_detect_2_form3_stage_1_cancers_10_posrisk_8_negrisk_40 | Patients | hypothetical |
| 0.117168 | 0.34658 | 0.707796 | pred_wait_1_detect_2_form3_stage_1_cancers_25_posrisk_8_negrisk_40 | Patients | hypothetical |
| 0.177685 | 0.402604 | 0.630238 | pred_wait_1_detect_2_form3_stage_2_cancers_1_posrisk_2_negrisk_1 | Patients | hypothetical |
| 0.182846 | 0.418716 | 0.657271 | pred_wait_1_detect_2_form3_stage_2_cancers_5_posrisk_2_negrisk_1 | Patients | hypothetical |
| 0.191994 | 0.441408 | 0.692158 | pred_wait_1_detect_2_form3_stage_2_cancers_10_posrisk_2_negrisk_1 | Patients | hypothetical |
| 0.246904 | 0.52136 | 0.783577 | pred_wait_1_detect_2_form3_stage_2_cancers_25_posrisk_2_negrisk_1 | Patients | hypothetical |
| 0.172999 | 0.39881 | 0.628372 | pred_wait_1_detect_2_form3_stage_2_cancers_1_posrisk_2_negrisk_5 | Patients | hypothetical |
| 0.177665 | 0.414849 | 0.655619 | pred_wait_1_detect_2_form3_stage_2_cancers_5_posrisk_2_negrisk_5 | Patients | hypothetical |
| 0.186554 | 0.437535 | 0.690041 | pred_wait_1_detect_2_form3_stage_2_cancers_10_posrisk_2_negrisk_5 | Patients | hypothetical |
| 0.240509 | 0.518117 | 0.783733 | pred_wait_1_detect_2_form3_stage_2_cancers_25_posrisk_2_negrisk_5 | Patients | hypothetical |
| 0.166163 | 0.394238 | 0.625924 | pred_wait_1_detect_2_form3_stage_2_cancers_1_posrisk_2_negrisk_10 | Patients | hypothetical |
| 0.169755 | 0.410138 | 0.652845 | pred_wait_1_detect_2_form3_stage_2_cancers_5_posrisk_2_negrisk_10 | Patients | hypothetical |
| 0.177252 | 0.432739 | 0.688226 | pred_wait_1_detect_2_form3_stage_2_cancers_10_posrisk_2_negrisk_10 | Patients | hypothetical |
| 0.228034 | 0.513809 | 0.782569 | pred_wait_1_detect_2_form3_stage_2_cancers_25_posrisk_2_negrisk_10 | Patients | hypothetical |
| 0.147697 | 0.375481 | 0.619559 | pred_wait_1_detect_2_form3_stage_2_cancers_1_posrisk_2_negrisk_40 | Patients | hypothetical |
| 0.150532 | 0.39012 | 0.646715 | pred_wait_1_detect_2_form3_stage_2_cancers_5_posrisk_2_negrisk_40 | Patients | hypothetical |
| 0.155941 | 0.411075 | 0.681777 | pred_wait_1_detect_2_form3_stage_2_cancers_10_posrisk_2_negrisk_40 | Patients | hypothetical |
| 0.198626 | 0.488042 | 0.774579 | pred_wait_1_detect_2_form3_stage_2_cancers_25_posrisk_2_negrisk_40 | Patients | hypothetical |
| 0.110715 | 0.343384 | 0.620335 | pred_wait_1_detect_2_form3_stage_2_cancers_1_posrisk_4_negrisk_1 | Patients | hypothetical |
| 0.111862 | 0.356254 | 0.647194 | pred_wait_1_detect_2_form3_stage_2_cancers_5_posrisk_4_negrisk_1 | Patients | hypothetical |
| 0.115167 | 0.374947 | 0.681878 | pred_wait_1_detect_2_form3_stage_2_cancers_10_posrisk_4_negrisk_1 | Patients | hypothetical |
| 0.147273 | 0.446215 | 0.774045 | pred_wait_1_detect_2_form3_stage_2_cancers_25_posrisk_4_negrisk_1 | Patients | hypothetical |
| 0.110715 | 0.33959 | 0.615487 | pred_wait_1_detect_2_form3_stage_2_cancers_1_posrisk_4_negrisk_5 | Patients | hypothetical |
| 0.111862 | 0.352387 | 0.643274 | pred_wait_1_detect_2_form3_stage_2_cancers_5_posrisk_4_negrisk_5 | Patients | hypothetical |
| 0.115167 | 0.371075 | 0.679174 | pred_wait_1_detect_2_form3_stage_2_cancers_10_posrisk_4_negrisk_5 | Patients | hypothetical |
| 0.147273 | 0.442972 | 0.774488 | pred_wait_1_detect_2_form3_stage_2_cancers_25_posrisk_4_negrisk_5 | Patients | hypothetical |
| 0.110715 | 0.335018 | 0.60839 | pred_wait_1_detect_2_form3_stage_2_cancers_1_posrisk_4_negrisk_10 | Patients | hypothetical |
| 0.111862 | 0.347676 | 0.636821 | pred_wait_1_detect_2_form3_stage_2_cancers_5_posrisk_4_negrisk_10 | Patients | hypothetical |
| 0.115167 | 0.366279 | 0.673792 | pred_wait_1_detect_2_form3_stage_2_cancers_10_posrisk_4_negrisk_10 | Patients | hypothetical |
| 0.147273 | 0.438664 | 0.772624 | pred_wait_1_detect_2_form3_stage_2_cancers_25_posrisk_4_negrisk_10 | Patients | hypothetical |
| 0.110618 | 0.316262 | 0.555857 | pred_wait_1_detect_2_form3_stage_2_cancers_1_posrisk_4_negrisk_40 | Patients | hypothetical |
| 0.111767 | 0.327657 | 0.579561 | pred_wait_1_detect_2_form3_stage_2_cancers_5_posrisk_4_negrisk_40 | Patients | hypothetical |
| 0.115153 | 0.344615 | 0.611993 | pred_wait_1_detect_2_form3_stage_2_cancers_10_posrisk_4_negrisk_40 | Patients | hypothetical |
| 0.147273 | 0.412897 | 0.707796 | pred_wait_1_detect_2_form3_stage_2_cancers_25_posrisk_4_negrisk_40 | Patients | hypothetical |
| 0.089891 | 0.31147 | 0.620335 | pred_wait_1_detect_2_form3_stage_2_cancers_1_posrisk_6_negrisk_1 | Patients | hypothetical |
| 0.091778 | 0.322679 | 0.647194 | pred_wait_1_detect_2_form3_stage_2_cancers_5_posrisk_6_negrisk_1 | Patients | hypothetical |
| 0.095358 | 0.338942 | 0.681878 | pred_wait_1_detect_2_form3_stage_2_cancers_10_posrisk_6_negrisk_1 | Patients | hypothetical |
| 0.120116 | 0.40078 | 0.774045 | pred_wait_1_detect_2_form3_stage_2_cancers_25_posrisk_6_negrisk_1 | Patients | hypothetical |
| 0.089891 | 0.307676 | 0.615487 | pred_wait_1_detect_2_form3_stage_2_cancers_1_posrisk_6_negrisk_5 | Patients | hypothetical |
| 0.091778 | 0.318812 | 0.643274 | pred_wait_1_detect_2_form3_stage_2_cancers_5_posrisk_6_negrisk_5 | Patients | hypothetical |
| 0.095358 | 0.335069 | 0.679174 | pred_wait_1_detect_2_form3_stage_2_cancers_10_posrisk_6_negrisk_5 | Patients | hypothetical |
| 0.120116 | 0.397537 | 0.774488 | pred_wait_1_detect_2_form3_stage_2_cancers_25_posrisk_6_negrisk_5 | Patients | hypothetical |
| 0.089891 | 0.303104 | 0.60839 | pred_wait_1_detect_2_form3_stage_2_cancers_1_posrisk_6_negrisk_10 | Patients | hypothetical |
| 0.091778 | 0.314101 | 0.636821 | pred_wait_1_detect_2_form3_stage_2_cancers_5_posrisk_6_negrisk_10 | Patients | hypothetical |
| 0.095358 | 0.330273 | 0.673792 | pred_wait_1_detect_2_form3_stage_2_cancers_10_posrisk_6_negrisk_10 | Patients | hypothetical |
| 0.120116 | 0.393229 | 0.772624 | pred_wait_1_detect_2_form3_stage_2_cancers_25_posrisk_6_negrisk_10 | Patients | hypothetical |
| 0.089891 | 0.284348 | 0.555857 | pred_wait_1_detect_2_form3_stage_2_cancers_1_posrisk_6_negrisk_40 | Patients | hypothetical |
| 0.091778 | 0.294083 | 0.579561 | pred_wait_1_detect_2_form3_stage_2_cancers_5_posrisk_6_negrisk_40 | Patients | hypothetical |
| 0.095358 | 0.308609 | 0.611993 | pred_wait_1_detect_2_form3_stage_2_cancers_10_posrisk_6_negrisk_40 | Patients | hypothetical |
| 0.120116 | 0.367462 | 0.707796 | pred_wait_1_detect_2_form3_stage_2_cancers_25_posrisk_6_negrisk_40 | Patients | hypothetical |
| 0.089238 | 0.297922 | 0.620335 | pred_wait_1_detect_2_form3_stage_2_cancers_1_posrisk_8_negrisk_1 | Patients | hypothetical |
| 0.091829 | 0.308532 | 0.647194 | pred_wait_1_detect_2_form3_stage_2_cancers_5_posrisk_8_negrisk_1 | Patients | hypothetical |
| 0.095875 | 0.323731 | 0.681878 | pred_wait_1_detect_2_form3_stage_2_cancers_10_posrisk_8_negrisk_1 | Patients | hypothetical |
| 0.117168 | 0.379898 | 0.774045 | pred_wait_1_detect_2_form3_stage_2_cancers_25_posrisk_8_negrisk_1 | Patients | hypothetical |
| 0.089238 | 0.294128 | 0.615487 | pred_wait_1_detect_2_form3_stage_2_cancers_1_posrisk_8_negrisk_5 | Patients | hypothetical |
| 0.091829 | 0.304665 | 0.643274 | pred_wait_1_detect_2_form3_stage_2_cancers_5_posrisk_8_negrisk_5 | Patients | hypothetical |
| 0.095875 | 0.319858 | 0.679174 | pred_wait_1_detect_2_form3_stage_2_cancers_10_posrisk_8_negrisk_5 | Patients | hypothetical |
| 0.117168 | 0.376655 | 0.774488 | pred_wait_1_detect_2_form3_stage_2_cancers_25_posrisk_8_negrisk_5 | Patients | hypothetical |
| 0.089238 | 0.289556 | 0.60839 | pred_wait_1_detect_2_form3_stage_2_cancers_1_posrisk_8_negrisk_10 | Patients | hypothetical |
| 0.091829 | 0.299954 | 0.636821 | pred_wait_1_detect_2_form3_stage_2_cancers_5_posrisk_8_negrisk_10 | Patients | hypothetical |
| 0.095875 | 0.315062 | 0.673792 | pred_wait_1_detect_2_form3_stage_2_cancers_10_posrisk_8_negrisk_10 | Patients | hypothetical |
| 0.117168 | 0.372347 | 0.772624 | pred_wait_1_detect_2_form3_stage_2_cancers_25_posrisk_8_negrisk_10 | Patients | hypothetical |
| 0.089238 | 0.2708 | 0.555857 | pred_wait_1_detect_2_form3_stage_2_cancers_1_posrisk_8_negrisk_40 | Patients | hypothetical |
| 0.091829 | 0.279935 | 0.579561 | pred_wait_1_detect_2_form3_stage_2_cancers_5_posrisk_8_negrisk_40 | Patients | hypothetical |
| 0.095875 | 0.293398 | 0.611993 | pred_wait_1_detect_2_form3_stage_2_cancers_10_posrisk_8_negrisk_40 | Patients | hypothetical |
| 0.117168 | 0.34658 | 0.707796 | pred_wait_1_detect_2_form3_stage_2_cancers_25_posrisk_8_negrisk_40 | Patients | hypothetical |
| 0.177685 | 0.402604 | 0.630238 | pred_wait_1_detect_2_form4_stage_1_cancers_1_posrisk_2_negrisk_1 | Patients | hypothetical |
| 0.182846 | 0.418716 | 0.657271 | pred_wait_1_detect_2_form4_stage_1_cancers_5_posrisk_2_negrisk_1 | Patients | hypothetical |
| 0.191994 | 0.441408 | 0.692158 | pred_wait_1_detect_2_form4_stage_1_cancers_10_posrisk_2_negrisk_1 | Patients | hypothetical |
| 0.246904 | 0.52136 | 0.783577 | pred_wait_1_detect_2_form4_stage_1_cancers_25_posrisk_2_negrisk_1 | Patients | hypothetical |
| 0.172999 | 0.39881 | 0.628372 | pred_wait_1_detect_2_form4_stage_1_cancers_1_posrisk_2_negrisk_5 | Patients | hypothetical |
| 0.177665 | 0.414849 | 0.655619 | pred_wait_1_detect_2_form4_stage_1_cancers_5_posrisk_2_negrisk_5 | Patients | hypothetical |
| 0.186554 | 0.437535 | 0.690041 | pred_wait_1_detect_2_form4_stage_1_cancers_10_posrisk_2_negrisk_5 | Patients | hypothetical |
| 0.240509 | 0.518117 | 0.783733 | pred_wait_1_detect_2_form4_stage_1_cancers_25_posrisk_2_negrisk_5 | Patients | hypothetical |
| 0.166163 | 0.394238 | 0.625924 | pred_wait_1_detect_2_form4_stage_1_cancers_1_posrisk_2_negrisk_10 | Patients | hypothetical |
| 0.169755 | 0.410138 | 0.652845 | pred_wait_1_detect_2_form4_stage_1_cancers_5_posrisk_2_negrisk_10 | Patients | hypothetical |
| 0.177252 | 0.432739 | 0.688226 | pred_wait_1_detect_2_form4_stage_1_cancers_10_posrisk_2_negrisk_10 | Patients | hypothetical |
| 0.228034 | 0.513809 | 0.782569 | pred_wait_1_detect_2_form4_stage_1_cancers_25_posrisk_2_negrisk_10 | Patients | hypothetical |
| 0.147697 | 0.375481 | 0.619559 | pred_wait_1_detect_2_form4_stage_1_cancers_1_posrisk_2_negrisk_40 | Patients | hypothetical |
| 0.150532 | 0.39012 | 0.646715 | pred_wait_1_detect_2_form4_stage_1_cancers_5_posrisk_2_negrisk_40 | Patients | hypothetical |
| 0.155941 | 0.411075 | 0.681777 | pred_wait_1_detect_2_form4_stage_1_cancers_10_posrisk_2_negrisk_40 | Patients | hypothetical |
| 0.198626 | 0.488042 | 0.774579 | pred_wait_1_detect_2_form4_stage_1_cancers_25_posrisk_2_negrisk_40 | Patients | hypothetical |
| 0.110715 | 0.343384 | 0.620335 | pred_wait_1_detect_2_form4_stage_1_cancers_1_posrisk_4_negrisk_1 | Patients | hypothetical |
| 0.111862 | 0.356254 | 0.647194 | pred_wait_1_detect_2_form4_stage_1_cancers_5_posrisk_4_negrisk_1 | Patients | hypothetical |
| 0.115167 | 0.374947 | 0.681878 | pred_wait_1_detect_2_form4_stage_1_cancers_10_posrisk_4_negrisk_1 | Patients | hypothetical |
| 0.147273 | 0.446215 | 0.774045 | pred_wait_1_detect_2_form4_stage_1_cancers_25_posrisk_4_negrisk_1 | Patients | hypothetical |
| 0.110715 | 0.33959 | 0.615487 | pred_wait_1_detect_2_form4_stage_1_cancers_1_posrisk_4_negrisk_5 | Patients | hypothetical |
| 0.111862 | 0.352387 | 0.643274 | pred_wait_1_detect_2_form4_stage_1_cancers_5_posrisk_4_negrisk_5 | Patients | hypothetical |
| 0.115167 | 0.371075 | 0.679174 | pred_wait_1_detect_2_form4_stage_1_cancers_10_posrisk_4_negrisk_5 | Patients | hypothetical |
| 0.147273 | 0.442972 | 0.774488 | pred_wait_1_detect_2_form4_stage_1_cancers_25_posrisk_4_negrisk_5 | Patients | hypothetical |
| 0.110715 | 0.335018 | 0.60839 | pred_wait_1_detect_2_form4_stage_1_cancers_1_posrisk_4_negrisk_10 | Patients | hypothetical |
| 0.111862 | 0.347676 | 0.636821 | pred_wait_1_detect_2_form4_stage_1_cancers_5_posrisk_4_negrisk_10 | Patients | hypothetical |
| 0.115167 | 0.366279 | 0.673792 | pred_wait_1_detect_2_form4_stage_1_cancers_10_posrisk_4_negrisk_10 | Patients | hypothetical |
| 0.147273 | 0.438664 | 0.772624 | pred_wait_1_detect_2_form4_stage_1_cancers_25_posrisk_4_negrisk_10 | Patients | hypothetical |
| 0.110618 | 0.316262 | 0.555857 | pred_wait_1_detect_2_form4_stage_1_cancers_1_posrisk_4_negrisk_40 | Patients | hypothetical |
| 0.111767 | 0.327657 | 0.579561 | pred_wait_1_detect_2_form4_stage_1_cancers_5_posrisk_4_negrisk_40 | Patients | hypothetical |
| 0.115153 | 0.344615 | 0.611993 | pred_wait_1_detect_2_form4_stage_1_cancers_10_posrisk_4_negrisk_40 | Patients | hypothetical |
| 0.147273 | 0.412897 | 0.707796 | pred_wait_1_detect_2_form4_stage_1_cancers_25_posrisk_4_negrisk_40 | Patients | hypothetical |
| 0.089891 | 0.31147 | 0.620335 | pred_wait_1_detect_2_form4_stage_1_cancers_1_posrisk_6_negrisk_1 | Patients | hypothetical |
| 0.091778 | 0.322679 | 0.647194 | pred_wait_1_detect_2_form4_stage_1_cancers_5_posrisk_6_negrisk_1 | Patients | hypothetical |
| 0.095358 | 0.338942 | 0.681878 | pred_wait_1_detect_2_form4_stage_1_cancers_10_posrisk_6_negrisk_1 | Patients | hypothetical |
| 0.120116 | 0.40078 | 0.774045 | pred_wait_1_detect_2_form4_stage_1_cancers_25_posrisk_6_negrisk_1 | Patients | hypothetical |
| 0.089891 | 0.307676 | 0.615487 | pred_wait_1_detect_2_form4_stage_1_cancers_1_posrisk_6_negrisk_5 | Patients | hypothetical |
| 0.091778 | 0.318812 | 0.643274 | pred_wait_1_detect_2_form4_stage_1_cancers_5_posrisk_6_negrisk_5 | Patients | hypothetical |
| 0.095358 | 0.335069 | 0.679174 | pred_wait_1_detect_2_form4_stage_1_cancers_10_posrisk_6_negrisk_5 | Patients | hypothetical |
| 0.120116 | 0.397537 | 0.774488 | pred_wait_1_detect_2_form4_stage_1_cancers_25_posrisk_6_negrisk_5 | Patients | hypothetical |
| 0.089891 | 0.303104 | 0.60839 | pred_wait_1_detect_2_form4_stage_1_cancers_1_posrisk_6_negrisk_10 | Patients | hypothetical |
| 0.091778 | 0.314101 | 0.636821 | pred_wait_1_detect_2_form4_stage_1_cancers_5_posrisk_6_negrisk_10 | Patients | hypothetical |
| 0.095358 | 0.330273 | 0.673792 | pred_wait_1_detect_2_form4_stage_1_cancers_10_posrisk_6_negrisk_10 | Patients | hypothetical |
| 0.120116 | 0.393229 | 0.772624 | pred_wait_1_detect_2_form4_stage_1_cancers_25_posrisk_6_negrisk_10 | Patients | hypothetical |
| 0.089891 | 0.284348 | 0.555857 | pred_wait_1_detect_2_form4_stage_1_cancers_1_posrisk_6_negrisk_40 | Patients | hypothetical |
| 0.091778 | 0.294083 | 0.579561 | pred_wait_1_detect_2_form4_stage_1_cancers_5_posrisk_6_negrisk_40 | Patients | hypothetical |
| 0.095358 | 0.308609 | 0.611993 | pred_wait_1_detect_2_form4_stage_1_cancers_10_posrisk_6_negrisk_40 | Patients | hypothetical |
| 0.120116 | 0.367462 | 0.707796 | pred_wait_1_detect_2_form4_stage_1_cancers_25_posrisk_6_negrisk_40 | Patients | hypothetical |
| 0.089238 | 0.297922 | 0.620335 | pred_wait_1_detect_2_form4_stage_1_cancers_1_posrisk_8_negrisk_1 | Patients | hypothetical |
| 0.091829 | 0.308532 | 0.647194 | pred_wait_1_detect_2_form4_stage_1_cancers_5_posrisk_8_negrisk_1 | Patients | hypothetical |
| 0.095875 | 0.323731 | 0.681878 | pred_wait_1_detect_2_form4_stage_1_cancers_10_posrisk_8_negrisk_1 | Patients | hypothetical |
| 0.117168 | 0.379898 | 0.774045 | pred_wait_1_detect_2_form4_stage_1_cancers_25_posrisk_8_negrisk_1 | Patients | hypothetical |
| 0.089238 | 0.294128 | 0.615487 | pred_wait_1_detect_2_form4_stage_1_cancers_1_posrisk_8_negrisk_5 | Patients | hypothetical |
| 0.091829 | 0.304665 | 0.643274 | pred_wait_1_detect_2_form4_stage_1_cancers_5_posrisk_8_negrisk_5 | Patients | hypothetical |
| 0.095875 | 0.319858 | 0.679174 | pred_wait_1_detect_2_form4_stage_1_cancers_10_posrisk_8_negrisk_5 | Patients | hypothetical |
| 0.117168 | 0.376655 | 0.774488 | pred_wait_1_detect_2_form4_stage_1_cancers_25_posrisk_8_negrisk_5 | Patients | hypothetical |
| 0.089238 | 0.289556 | 0.60839 | pred_wait_1_detect_2_form4_stage_1_cancers_1_posrisk_8_negrisk_10 | Patients | hypothetical |
| 0.091829 | 0.299954 | 0.636821 | pred_wait_1_detect_2_form4_stage_1_cancers_5_posrisk_8_negrisk_10 | Patients | hypothetical |
| 0.095875 | 0.315062 | 0.673792 | pred_wait_1_detect_2_form4_stage_1_cancers_10_posrisk_8_negrisk_10 | Patients | hypothetical |
| 0.117168 | 0.372347 | 0.772624 | pred_wait_1_detect_2_form4_stage_1_cancers_25_posrisk_8_negrisk_10 | Patients | hypothetical |
| 0.089238 | 0.2708 | 0.555857 | pred_wait_1_detect_2_form4_stage_1_cancers_1_posrisk_8_negrisk_40 | Patients | hypothetical |
| 0.091829 | 0.279935 | 0.579561 | pred_wait_1_detect_2_form4_stage_1_cancers_5_posrisk_8_negrisk_40 | Patients | hypothetical |
| 0.095875 | 0.293398 | 0.611993 | pred_wait_1_detect_2_form4_stage_1_cancers_10_posrisk_8_negrisk_40 | Patients | hypothetical |
| 0.117168 | 0.34658 | 0.707796 | pred_wait_1_detect_2_form4_stage_1_cancers_25_posrisk_8_negrisk_40 | Patients | hypothetical |
| 0.177685 | 0.402604 | 0.630238 | pred_wait_1_detect_2_form4_stage_2_cancers_1_posrisk_2_negrisk_1 | Patients | hypothetical |
| 0.182846 | 0.418716 | 0.657271 | pred_wait_1_detect_2_form4_stage_2_cancers_5_posrisk_2_negrisk_1 | Patients | hypothetical |
| 0.191994 | 0.441408 | 0.692158 | pred_wait_1_detect_2_form4_stage_2_cancers_10_posrisk_2_negrisk_1 | Patients | hypothetical |
| 0.246904 | 0.52136 | 0.783577 | pred_wait_1_detect_2_form4_stage_2_cancers_25_posrisk_2_negrisk_1 | Patients | hypothetical |
| 0.172999 | 0.39881 | 0.628372 | pred_wait_1_detect_2_form4_stage_2_cancers_1_posrisk_2_negrisk_5 | Patients | hypothetical |
| 0.177665 | 0.414849 | 0.655619 | pred_wait_1_detect_2_form4_stage_2_cancers_5_posrisk_2_negrisk_5 | Patients | hypothetical |
| 0.186554 | 0.437535 | 0.690041 | pred_wait_1_detect_2_form4_stage_2_cancers_10_posrisk_2_negrisk_5 | Patients | hypothetical |
| 0.240509 | 0.518117 | 0.783733 | pred_wait_1_detect_2_form4_stage_2_cancers_25_posrisk_2_negrisk_5 | Patients | hypothetical |
| 0.166163 | 0.394238 | 0.625924 | pred_wait_1_detect_2_form4_stage_2_cancers_1_posrisk_2_negrisk_10 | Patients | hypothetical |
| 0.169755 | 0.410138 | 0.652845 | pred_wait_1_detect_2_form4_stage_2_cancers_5_posrisk_2_negrisk_10 | Patients | hypothetical |
| 0.177252 | 0.432739 | 0.688226 | pred_wait_1_detect_2_form4_stage_2_cancers_10_posrisk_2_negrisk_10 | Patients | hypothetical |
| 0.228034 | 0.513809 | 0.782569 | pred_wait_1_detect_2_form4_stage_2_cancers_25_posrisk_2_negrisk_10 | Patients | hypothetical |
| 0.147697 | 0.375481 | 0.619559 | pred_wait_1_detect_2_form4_stage_2_cancers_1_posrisk_2_negrisk_40 | Patients | hypothetical |
| 0.150532 | 0.39012 | 0.646715 | pred_wait_1_detect_2_form4_stage_2_cancers_5_posrisk_2_negrisk_40 | Patients | hypothetical |
| 0.155941 | 0.411075 | 0.681777 | pred_wait_1_detect_2_form4_stage_2_cancers_10_posrisk_2_negrisk_40 | Patients | hypothetical |
| 0.198626 | 0.488042 | 0.774579 | pred_wait_1_detect_2_form4_stage_2_cancers_25_posrisk_2_negrisk_40 | Patients | hypothetical |
| 0.110715 | 0.343384 | 0.620335 | pred_wait_1_detect_2_form4_stage_2_cancers_1_posrisk_4_negrisk_1 | Patients | hypothetical |
| 0.111862 | 0.356254 | 0.647194 | pred_wait_1_detect_2_form4_stage_2_cancers_5_posrisk_4_negrisk_1 | Patients | hypothetical |
| 0.115167 | 0.374947 | 0.681878 | pred_wait_1_detect_2_form4_stage_2_cancers_10_posrisk_4_negrisk_1 | Patients | hypothetical |
| 0.147273 | 0.446215 | 0.774045 | pred_wait_1_detect_2_form4_stage_2_cancers_25_posrisk_4_negrisk_1 | Patients | hypothetical |
| 0.110715 | 0.33959 | 0.615487 | pred_wait_1_detect_2_form4_stage_2_cancers_1_posrisk_4_negrisk_5 | Patients | hypothetical |
| 0.111862 | 0.352387 | 0.643274 | pred_wait_1_detect_2_form4_stage_2_cancers_5_posrisk_4_negrisk_5 | Patients | hypothetical |
| 0.115167 | 0.371075 | 0.679174 | pred_wait_1_detect_2_form4_stage_2_cancers_10_posrisk_4_negrisk_5 | Patients | hypothetical |
| 0.147273 | 0.442972 | 0.774488 | pred_wait_1_detect_2_form4_stage_2_cancers_25_posrisk_4_negrisk_5 | Patients | hypothetical |
| 0.110715 | 0.335018 | 0.60839 | pred_wait_1_detect_2_form4_stage_2_cancers_1_posrisk_4_negrisk_10 | Patients | hypothetical |
| 0.111862 | 0.347676 | 0.636821 | pred_wait_1_detect_2_form4_stage_2_cancers_5_posrisk_4_negrisk_10 | Patients | hypothetical |
| 0.115167 | 0.366279 | 0.673792 | pred_wait_1_detect_2_form4_stage_2_cancers_10_posrisk_4_negrisk_10 | Patients | hypothetical |
| 0.147273 | 0.438664 | 0.772624 | pred_wait_1_detect_2_form4_stage_2_cancers_25_posrisk_4_negrisk_10 | Patients | hypothetical |
| 0.110618 | 0.316262 | 0.555857 | pred_wait_1_detect_2_form4_stage_2_cancers_1_posrisk_4_negrisk_40 | Patients | hypothetical |
| 0.111767 | 0.327657 | 0.579561 | pred_wait_1_detect_2_form4_stage_2_cancers_5_posrisk_4_negrisk_40 | Patients | hypothetical |
| 0.115153 | 0.344615 | 0.611993 | pred_wait_1_detect_2_form4_stage_2_cancers_10_posrisk_4_negrisk_40 | Patients | hypothetical |
| 0.147273 | 0.412897 | 0.707796 | pred_wait_1_detect_2_form4_stage_2_cancers_25_posrisk_4_negrisk_40 | Patients | hypothetical |
| 0.089891 | 0.31147 | 0.620335 | pred_wait_1_detect_2_form4_stage_2_cancers_1_posrisk_6_negrisk_1 | Patients | hypothetical |
| 0.091778 | 0.322679 | 0.647194 | pred_wait_1_detect_2_form4_stage_2_cancers_5_posrisk_6_negrisk_1 | Patients | hypothetical |
| 0.095358 | 0.338942 | 0.681878 | pred_wait_1_detect_2_form4_stage_2_cancers_10_posrisk_6_negrisk_1 | Patients | hypothetical |
| 0.120116 | 0.40078 | 0.774045 | pred_wait_1_detect_2_form4_stage_2_cancers_25_posrisk_6_negrisk_1 | Patients | hypothetical |
| 0.089891 | 0.307676 | 0.615487 | pred_wait_1_detect_2_form4_stage_2_cancers_1_posrisk_6_negrisk_5 | Patients | hypothetical |
| 0.091778 | 0.318812 | 0.643274 | pred_wait_1_detect_2_form4_stage_2_cancers_5_posrisk_6_negrisk_5 | Patients | hypothetical |
| 0.095358 | 0.335069 | 0.679174 | pred_wait_1_detect_2_form4_stage_2_cancers_10_posrisk_6_negrisk_5 | Patients | hypothetical |
| 0.120116 | 0.397537 | 0.774488 | pred_wait_1_detect_2_form4_stage_2_cancers_25_posrisk_6_negrisk_5 | Patients | hypothetical |
| 0.089891 | 0.303104 | 0.60839 | pred_wait_1_detect_2_form4_stage_2_cancers_1_posrisk_6_negrisk_10 | Patients | hypothetical |
| 0.091778 | 0.314101 | 0.636821 | pred_wait_1_detect_2_form4_stage_2_cancers_5_posrisk_6_negrisk_10 | Patients | hypothetical |
| 0.095358 | 0.330273 | 0.673792 | pred_wait_1_detect_2_form4_stage_2_cancers_10_posrisk_6_negrisk_10 | Patients | hypothetical |
| 0.120116 | 0.393229 | 0.772624 | pred_wait_1_detect_2_form4_stage_2_cancers_25_posrisk_6_negrisk_10 | Patients | hypothetical |
| 0.089891 | 0.284348 | 0.555857 | pred_wait_1_detect_2_form4_stage_2_cancers_1_posrisk_6_negrisk_40 | Patients | hypothetical |
| 0.091778 | 0.294083 | 0.579561 | pred_wait_1_detect_2_form4_stage_2_cancers_5_posrisk_6_negrisk_40 | Patients | hypothetical |
| 0.095358 | 0.308609 | 0.611993 | pred_wait_1_detect_2_form4_stage_2_cancers_10_posrisk_6_negrisk_40 | Patients | hypothetical |
| 0.120116 | 0.367462 | 0.707796 | pred_wait_1_detect_2_form4_stage_2_cancers_25_posrisk_6_negrisk_40 | Patients | hypothetical |
| 0.089238 | 0.297922 | 0.620335 | pred_wait_1_detect_2_form4_stage_2_cancers_1_posrisk_8_negrisk_1 | Patients | hypothetical |
| 0.091829 | 0.308532 | 0.647194 | pred_wait_1_detect_2_form4_stage_2_cancers_5_posrisk_8_negrisk_1 | Patients | hypothetical |
| 0.095875 | 0.323731 | 0.681878 | pred_wait_1_detect_2_form4_stage_2_cancers_10_posrisk_8_negrisk_1 | Patients | hypothetical |
| 0.117168 | 0.379898 | 0.774045 | pred_wait_1_detect_2_form4_stage_2_cancers_25_posrisk_8_negrisk_1 | Patients | hypothetical |
| 0.089238 | 0.294128 | 0.615487 | pred_wait_1_detect_2_form4_stage_2_cancers_1_posrisk_8_negrisk_5 | Patients | hypothetical |
| 0.091829 | 0.304665 | 0.643274 | pred_wait_1_detect_2_form4_stage_2_cancers_5_posrisk_8_negrisk_5 | Patients | hypothetical |
| 0.095875 | 0.319858 | 0.679174 | pred_wait_1_detect_2_form4_stage_2_cancers_10_posrisk_8_negrisk_5 | Patients | hypothetical |
| 0.117168 | 0.376655 | 0.774488 | pred_wait_1_detect_2_form4_stage_2_cancers_25_posrisk_8_negrisk_5 | Patients | hypothetical |
| 0.089238 | 0.289556 | 0.60839 | pred_wait_1_detect_2_form4_stage_2_cancers_1_posrisk_8_negrisk_10 | Patients | hypothetical |
| 0.091829 | 0.299954 | 0.636821 | pred_wait_1_detect_2_form4_stage_2_cancers_5_posrisk_8_negrisk_10 | Patients | hypothetical |
| 0.095875 | 0.315062 | 0.673792 | pred_wait_1_detect_2_form4_stage_2_cancers_10_posrisk_8_negrisk_10 | Patients | hypothetical |
| 0.117168 | 0.372347 | 0.772624 | pred_wait_1_detect_2_form4_stage_2_cancers_25_posrisk_8_negrisk_10 | Patients | hypothetical |
| 0.089238 | 0.2708 | 0.555857 | pred_wait_1_detect_2_form4_stage_2_cancers_1_posrisk_8_negrisk_40 | Patients | hypothetical |
| 0.091829 | 0.279935 | 0.579561 | pred_wait_1_detect_2_form4_stage_2_cancers_5_posrisk_8_negrisk_40 | Patients | hypothetical |
| 0.095875 | 0.293398 | 0.611993 | pred_wait_1_detect_2_form4_stage_2_cancers_10_posrisk_8_negrisk_40 | Patients | hypothetical |
| 0.117168 | 0.34658 | 0.707796 | pred_wait_1_detect_2_form4_stage_2_cancers_25_posrisk_8_negrisk_40 | Patients | hypothetical |
| 0.177685 | 0.402604 | 0.630238 | pred_wait_2_detect_1_form1_stage_1_cancers_1_posrisk_2_negrisk_1 | Patients | hypothetical |
| 0.182846 | 0.418716 | 0.657271 | pred_wait_2_detect_1_form1_stage_1_cancers_5_posrisk_2_negrisk_1 | Patients | hypothetical |
| 0.191994 | 0.441408 | 0.692158 | pred_wait_2_detect_1_form1_stage_1_cancers_10_posrisk_2_negrisk_1 | Patients | hypothetical |
| 0.246904 | 0.52136 | 0.783577 | pred_wait_2_detect_1_form1_stage_1_cancers_25_posrisk_2_negrisk_1 | Patients | hypothetical |
| 0.172999 | 0.39881 | 0.628372 | pred_wait_2_detect_1_form1_stage_1_cancers_1_posrisk_2_negrisk_5 | Patients | hypothetical |
| 0.177665 | 0.414849 | 0.655619 | pred_wait_2_detect_1_form1_stage_1_cancers_5_posrisk_2_negrisk_5 | Patients | hypothetical |
| 0.186554 | 0.437535 | 0.690041 | pred_wait_2_detect_1_form1_stage_1_cancers_10_posrisk_2_negrisk_5 | Patients | hypothetical |
| 0.240509 | 0.518117 | 0.783733 | pred_wait_2_detect_1_form1_stage_1_cancers_25_posrisk_2_negrisk_5 | Patients | hypothetical |
| 0.166163 | 0.394238 | 0.625924 | pred_wait_2_detect_1_form1_stage_1_cancers_1_posrisk_2_negrisk_10 | Patients | hypothetical |
| 0.169755 | 0.410138 | 0.652845 | pred_wait_2_detect_1_form1_stage_1_cancers_5_posrisk_2_negrisk_10 | Patients | hypothetical |
| 0.177252 | 0.432739 | 0.688226 | pred_wait_2_detect_1_form1_stage_1_cancers_10_posrisk_2_negrisk_10 | Patients | hypothetical |
| 0.228034 | 0.513809 | 0.782569 | pred_wait_2_detect_1_form1_stage_1_cancers_25_posrisk_2_negrisk_10 | Patients | hypothetical |
| 0.147697 | 0.375481 | 0.619559 | pred_wait_2_detect_1_form1_stage_1_cancers_1_posrisk_2_negrisk_40 | Patients | hypothetical |
| 0.150532 | 0.39012 | 0.646715 | pred_wait_2_detect_1_form1_stage_1_cancers_5_posrisk_2_negrisk_40 | Patients | hypothetical |
| 0.155941 | 0.411075 | 0.681777 | pred_wait_2_detect_1_form1_stage_1_cancers_10_posrisk_2_negrisk_40 | Patients | hypothetical |
| 0.198626 | 0.488042 | 0.774579 | pred_wait_2_detect_1_form1_stage_1_cancers_25_posrisk_2_negrisk_40 | Patients | hypothetical |
| 0.110715 | 0.343384 | 0.620335 | pred_wait_2_detect_1_form1_stage_1_cancers_1_posrisk_4_negrisk_1 | Patients | hypothetical |
| 0.111862 | 0.356254 | 0.647194 | pred_wait_2_detect_1_form1_stage_1_cancers_5_posrisk_4_negrisk_1 | Patients | hypothetical |
| 0.115167 | 0.374947 | 0.681878 | pred_wait_2_detect_1_form1_stage_1_cancers_10_posrisk_4_negrisk_1 | Patients | hypothetical |
| 0.147273 | 0.446215 | 0.774045 | pred_wait_2_detect_1_form1_stage_1_cancers_25_posrisk_4_negrisk_1 | Patients | hypothetical |
| 0.110715 | 0.33959 | 0.615487 | pred_wait_2_detect_1_form1_stage_1_cancers_1_posrisk_4_negrisk_5 | Patients | hypothetical |
| 0.111862 | 0.352387 | 0.643274 | pred_wait_2_detect_1_form1_stage_1_cancers_5_posrisk_4_negrisk_5 | Patients | hypothetical |
| 0.115167 | 0.371075 | 0.679174 | pred_wait_2_detect_1_form1_stage_1_cancers_10_posrisk_4_negrisk_5 | Patients | hypothetical |
| 0.147273 | 0.442972 | 0.774488 | pred_wait_2_detect_1_form1_stage_1_cancers_25_posrisk_4_negrisk_5 | Patients | hypothetical |
| 0.110715 | 0.335018 | 0.60839 | pred_wait_2_detect_1_form1_stage_1_cancers_1_posrisk_4_negrisk_10 | Patients | hypothetical |
| 0.111862 | 0.347676 | 0.636821 | pred_wait_2_detect_1_form1_stage_1_cancers_5_posrisk_4_negrisk_10 | Patients | hypothetical |
| 0.115167 | 0.366279 | 0.673792 | pred_wait_2_detect_1_form1_stage_1_cancers_10_posrisk_4_negrisk_10 | Patients | hypothetical |
| 0.147273 | 0.438664 | 0.772624 | pred_wait_2_detect_1_form1_stage_1_cancers_25_posrisk_4_negrisk_10 | Patients | hypothetical |
| 0.110618 | 0.316262 | 0.555857 | pred_wait_2_detect_1_form1_stage_1_cancers_1_posrisk_4_negrisk_40 | Patients | hypothetical |
| 0.111767 | 0.327657 | 0.579561 | pred_wait_2_detect_1_form1_stage_1_cancers_5_posrisk_4_negrisk_40 | Patients | hypothetical |
| 0.115153 | 0.344615 | 0.611993 | pred_wait_2_detect_1_form1_stage_1_cancers_10_posrisk_4_negrisk_40 | Patients | hypothetical |
| 0.147273 | 0.412897 | 0.707796 | pred_wait_2_detect_1_form1_stage_1_cancers_25_posrisk_4_negrisk_40 | Patients | hypothetical |
| 0.089891 | 0.31147 | 0.620335 | pred_wait_2_detect_1_form1_stage_1_cancers_1_posrisk_6_negrisk_1 | Patients | hypothetical |
| 0.091778 | 0.322679 | 0.647194 | pred_wait_2_detect_1_form1_stage_1_cancers_5_posrisk_6_negrisk_1 | Patients | hypothetical |
| 0.095358 | 0.338942 | 0.681878 | pred_wait_2_detect_1_form1_stage_1_cancers_10_posrisk_6_negrisk_1 | Patients | hypothetical |
| 0.120116 | 0.40078 | 0.774045 | pred_wait_2_detect_1_form1_stage_1_cancers_25_posrisk_6_negrisk_1 | Patients | hypothetical |
| 0.089891 | 0.307676 | 0.615487 | pred_wait_2_detect_1_form1_stage_1_cancers_1_posrisk_6_negrisk_5 | Patients | hypothetical |
| 0.091778 | 0.318812 | 0.643274 | pred_wait_2_detect_1_form1_stage_1_cancers_5_posrisk_6_negrisk_5 | Patients | hypothetical |
| 0.095358 | 0.335069 | 0.679174 | pred_wait_2_detect_1_form1_stage_1_cancers_10_posrisk_6_negrisk_5 | Patients | hypothetical |
| 0.120116 | 0.397537 | 0.774488 | pred_wait_2_detect_1_form1_stage_1_cancers_25_posrisk_6_negrisk_5 | Patients | hypothetical |
| 0.089891 | 0.303104 | 0.60839 | pred_wait_2_detect_1_form1_stage_1_cancers_1_posrisk_6_negrisk_10 | Patients | hypothetical |
| 0.091778 | 0.314101 | 0.636821 | pred_wait_2_detect_1_form1_stage_1_cancers_5_posrisk_6_negrisk_10 | Patients | hypothetical |
| 0.095358 | 0.330273 | 0.673792 | pred_wait_2_detect_1_form1_stage_1_cancers_10_posrisk_6_negrisk_10 | Patients | hypothetical |
| 0.120116 | 0.393229 | 0.772624 | pred_wait_2_detect_1_form1_stage_1_cancers_25_posrisk_6_negrisk_10 | Patients | hypothetical |
| 0.089891 | 0.284348 | 0.555857 | pred_wait_2_detect_1_form1_stage_1_cancers_1_posrisk_6_negrisk_40 | Patients | hypothetical |
| 0.091778 | 0.294083 | 0.579561 | pred_wait_2_detect_1_form1_stage_1_cancers_5_posrisk_6_negrisk_40 | Patients | hypothetical |
| 0.095358 | 0.308609 | 0.611993 | pred_wait_2_detect_1_form1_stage_1_cancers_10_posrisk_6_negrisk_40 | Patients | hypothetical |
| 0.120116 | 0.367462 | 0.707796 | pred_wait_2_detect_1_form1_stage_1_cancers_25_posrisk_6_negrisk_40 | Patients | hypothetical |
| 0.089238 | 0.297922 | 0.620335 | pred_wait_2_detect_1_form1_stage_1_cancers_1_posrisk_8_negrisk_1 | Patients | hypothetical |
| 0.091829 | 0.308532 | 0.647194 | pred_wait_2_detect_1_form1_stage_1_cancers_5_posrisk_8_negrisk_1 | Patients | hypothetical |
| 0.095875 | 0.323731 | 0.681878 | pred_wait_2_detect_1_form1_stage_1_cancers_10_posrisk_8_negrisk_1 | Patients | hypothetical |
| 0.117168 | 0.379898 | 0.774045 | pred_wait_2_detect_1_form1_stage_1_cancers_25_posrisk_8_negrisk_1 | Patients | hypothetical |
| 0.089238 | 0.294128 | 0.615487 | pred_wait_2_detect_1_form1_stage_1_cancers_1_posrisk_8_negrisk_5 | Patients | hypothetical |
| 0.091829 | 0.304665 | 0.643274 | pred_wait_2_detect_1_form1_stage_1_cancers_5_posrisk_8_negrisk_5 | Patients | hypothetical |
| 0.095875 | 0.319858 | 0.679174 | pred_wait_2_detect_1_form1_stage_1_cancers_10_posrisk_8_negrisk_5 | Patients | hypothetical |
| 0.117168 | 0.376655 | 0.774488 | pred_wait_2_detect_1_form1_stage_1_cancers_25_posrisk_8_negrisk_5 | Patients | hypothetical |
| 0.089238 | 0.289556 | 0.60839 | pred_wait_2_detect_1_form1_stage_1_cancers_1_posrisk_8_negrisk_10 | Patients | hypothetical |
| 0.091829 | 0.299954 | 0.636821 | pred_wait_2_detect_1_form1_stage_1_cancers_5_posrisk_8_negrisk_10 | Patients | hypothetical |
| 0.095875 | 0.315062 | 0.673792 | pred_wait_2_detect_1_form1_stage_1_cancers_10_posrisk_8_negrisk_10 | Patients | hypothetical |
| 0.117168 | 0.372347 | 0.772624 | pred_wait_2_detect_1_form1_stage_1_cancers_25_posrisk_8_negrisk_10 | Patients | hypothetical |
| 0.089238 | 0.2708 | 0.555857 | pred_wait_2_detect_1_form1_stage_1_cancers_1_posrisk_8_negrisk_40 | Patients | hypothetical |
| 0.091829 | 0.279935 | 0.579561 | pred_wait_2_detect_1_form1_stage_1_cancers_5_posrisk_8_negrisk_40 | Patients | hypothetical |
| 0.095875 | 0.293398 | 0.611993 | pred_wait_2_detect_1_form1_stage_1_cancers_10_posrisk_8_negrisk_40 | Patients | hypothetical |
| 0.117168 | 0.34658 | 0.707796 | pred_wait_2_detect_1_form1_stage_1_cancers_25_posrisk_8_negrisk_40 | Patients | hypothetical |
| 0.177685 | 0.402604 | 0.630238 | pred_wait_2_detect_1_form1_stage_2_cancers_1_posrisk_2_negrisk_1 | Patients | hypothetical |
| 0.182846 | 0.418716 | 0.657271 | pred_wait_2_detect_1_form1_stage_2_cancers_5_posrisk_2_negrisk_1 | Patients | hypothetical |
| 0.191994 | 0.441408 | 0.692158 | pred_wait_2_detect_1_form1_stage_2_cancers_10_posrisk_2_negrisk_1 | Patients | hypothetical |
| 0.246904 | 0.52136 | 0.783577 | pred_wait_2_detect_1_form1_stage_2_cancers_25_posrisk_2_negrisk_1 | Patients | hypothetical |
| 0.172999 | 0.39881 | 0.628372 | pred_wait_2_detect_1_form1_stage_2_cancers_1_posrisk_2_negrisk_5 | Patients | hypothetical |
| 0.177665 | 0.414849 | 0.655619 | pred_wait_2_detect_1_form1_stage_2_cancers_5_posrisk_2_negrisk_5 | Patients | hypothetical |
| 0.186554 | 0.437535 | 0.690041 | pred_wait_2_detect_1_form1_stage_2_cancers_10_posrisk_2_negrisk_5 | Patients | hypothetical |
| 0.240509 | 0.518117 | 0.783733 | pred_wait_2_detect_1_form1_stage_2_cancers_25_posrisk_2_negrisk_5 | Patients | hypothetical |
| 0.166163 | 0.394238 | 0.625924 | pred_wait_2_detect_1_form1_stage_2_cancers_1_posrisk_2_negrisk_10 | Patients | hypothetical |
| 0.169755 | 0.410138 | 0.652845 | pred_wait_2_detect_1_form1_stage_2_cancers_5_posrisk_2_negrisk_10 | Patients | hypothetical |
| 0.177252 | 0.432739 | 0.688226 | pred_wait_2_detect_1_form1_stage_2_cancers_10_posrisk_2_negrisk_10 | Patients | hypothetical |
| 0.228034 | 0.513809 | 0.782569 | pred_wait_2_detect_1_form1_stage_2_cancers_25_posrisk_2_negrisk_10 | Patients | hypothetical |
| 0.147697 | 0.375481 | 0.619559 | pred_wait_2_detect_1_form1_stage_2_cancers_1_posrisk_2_negrisk_40 | Patients | hypothetical |
| 0.150532 | 0.39012 | 0.646715 | pred_wait_2_detect_1_form1_stage_2_cancers_5_posrisk_2_negrisk_40 | Patients | hypothetical |
| 0.155941 | 0.411075 | 0.681777 | pred_wait_2_detect_1_form1_stage_2_cancers_10_posrisk_2_negrisk_40 | Patients | hypothetical |
| 0.198626 | 0.488042 | 0.774579 | pred_wait_2_detect_1_form1_stage_2_cancers_25_posrisk_2_negrisk_40 | Patients | hypothetical |
| 0.110715 | 0.343384 | 0.620335 | pred_wait_2_detect_1_form1_stage_2_cancers_1_posrisk_4_negrisk_1 | Patients | hypothetical |
| 0.111862 | 0.356254 | 0.647194 | pred_wait_2_detect_1_form1_stage_2_cancers_5_posrisk_4_negrisk_1 | Patients | hypothetical |
| 0.115167 | 0.374947 | 0.681878 | pred_wait_2_detect_1_form1_stage_2_cancers_10_posrisk_4_negrisk_1 | Patients | hypothetical |
| 0.147273 | 0.446215 | 0.774045 | pred_wait_2_detect_1_form1_stage_2_cancers_25_posrisk_4_negrisk_1 | Patients | hypothetical |
| 0.110715 | 0.33959 | 0.615487 | pred_wait_2_detect_1_form1_stage_2_cancers_1_posrisk_4_negrisk_5 | Patients | hypothetical |
| 0.111862 | 0.352387 | 0.643274 | pred_wait_2_detect_1_form1_stage_2_cancers_5_posrisk_4_negrisk_5 | Patients | hypothetical |
| 0.115167 | 0.371075 | 0.679174 | pred_wait_2_detect_1_form1_stage_2_cancers_10_posrisk_4_negrisk_5 | Patients | hypothetical |
| 0.147273 | 0.442972 | 0.774488 | pred_wait_2_detect_1_form1_stage_2_cancers_25_posrisk_4_negrisk_5 | Patients | hypothetical |
| 0.110715 | 0.335018 | 0.60839 | pred_wait_2_detect_1_form1_stage_2_cancers_1_posrisk_4_negrisk_10 | Patients | hypothetical |
| 0.111862 | 0.347676 | 0.636821 | pred_wait_2_detect_1_form1_stage_2_cancers_5_posrisk_4_negrisk_10 | Patients | hypothetical |
| 0.115167 | 0.366279 | 0.673792 | pred_wait_2_detect_1_form1_stage_2_cancers_10_posrisk_4_negrisk_10 | Patients | hypothetical |
| 0.147273 | 0.438664 | 0.772624 | pred_wait_2_detect_1_form1_stage_2_cancers_25_posrisk_4_negrisk_10 | Patients | hypothetical |
| 0.110618 | 0.316262 | 0.555857 | pred_wait_2_detect_1_form1_stage_2_cancers_1_posrisk_4_negrisk_40 | Patients | hypothetical |
| 0.111767 | 0.327657 | 0.579561 | pred_wait_2_detect_1_form1_stage_2_cancers_5_posrisk_4_negrisk_40 | Patients | hypothetical |
| 0.115153 | 0.344615 | 0.611993 | pred_wait_2_detect_1_form1_stage_2_cancers_10_posrisk_4_negrisk_40 | Patients | hypothetical |
| 0.147273 | 0.412897 | 0.707796 | pred_wait_2_detect_1_form1_stage_2_cancers_25_posrisk_4_negrisk_40 | Patients | hypothetical |
| 0.089891 | 0.31147 | 0.620335 | pred_wait_2_detect_1_form1_stage_2_cancers_1_posrisk_6_negrisk_1 | Patients | hypothetical |
| 0.091778 | 0.322679 | 0.647194 | pred_wait_2_detect_1_form1_stage_2_cancers_5_posrisk_6_negrisk_1 | Patients | hypothetical |
| 0.095358 | 0.338942 | 0.681878 | pred_wait_2_detect_1_form1_stage_2_cancers_10_posrisk_6_negrisk_1 | Patients | hypothetical |
| 0.120116 | 0.40078 | 0.774045 | pred_wait_2_detect_1_form1_stage_2_cancers_25_posrisk_6_negrisk_1 | Patients | hypothetical |
| 0.089891 | 0.307676 | 0.615487 | pred_wait_2_detect_1_form1_stage_2_cancers_1_posrisk_6_negrisk_5 | Patients | hypothetical |
| 0.091778 | 0.318812 | 0.643274 | pred_wait_2_detect_1_form1_stage_2_cancers_5_posrisk_6_negrisk_5 | Patients | hypothetical |
| 0.095358 | 0.335069 | 0.679174 | pred_wait_2_detect_1_form1_stage_2_cancers_10_posrisk_6_negrisk_5 | Patients | hypothetical |
| 0.120116 | 0.397537 | 0.774488 | pred_wait_2_detect_1_form1_stage_2_cancers_25_posrisk_6_negrisk_5 | Patients | hypothetical |
| 0.089891 | 0.303104 | 0.60839 | pred_wait_2_detect_1_form1_stage_2_cancers_1_posrisk_6_negrisk_10 | Patients | hypothetical |
| 0.091778 | 0.314101 | 0.636821 | pred_wait_2_detect_1_form1_stage_2_cancers_5_posrisk_6_negrisk_10 | Patients | hypothetical |
| 0.095358 | 0.330273 | 0.673792 | pred_wait_2_detect_1_form1_stage_2_cancers_10_posrisk_6_negrisk_10 | Patients | hypothetical |
| 0.120116 | 0.393229 | 0.772624 | pred_wait_2_detect_1_form1_stage_2_cancers_25_posrisk_6_negrisk_10 | Patients | hypothetical |
| 0.089891 | 0.284348 | 0.555857 | pred_wait_2_detect_1_form1_stage_2_cancers_1_posrisk_6_negrisk_40 | Patients | hypothetical |
| 0.091778 | 0.294083 | 0.579561 | pred_wait_2_detect_1_form1_stage_2_cancers_5_posrisk_6_negrisk_40 | Patients | hypothetical |
| 0.095358 | 0.308609 | 0.611993 | pred_wait_2_detect_1_form1_stage_2_cancers_10_posrisk_6_negrisk_40 | Patients | hypothetical |
| 0.120116 | 0.367462 | 0.707796 | pred_wait_2_detect_1_form1_stage_2_cancers_25_posrisk_6_negrisk_40 | Patients | hypothetical |
| 0.089238 | 0.297922 | 0.620335 | pred_wait_2_detect_1_form1_stage_2_cancers_1_posrisk_8_negrisk_1 | Patients | hypothetical |
| 0.091829 | 0.308532 | 0.647194 | pred_wait_2_detect_1_form1_stage_2_cancers_5_posrisk_8_negrisk_1 | Patients | hypothetical |
| 0.095875 | 0.323731 | 0.681878 | pred_wait_2_detect_1_form1_stage_2_cancers_10_posrisk_8_negrisk_1 | Patients | hypothetical |
| 0.117168 | 0.379898 | 0.774045 | pred_wait_2_detect_1_form1_stage_2_cancers_25_posrisk_8_negrisk_1 | Patients | hypothetical |
| 0.089238 | 0.294128 | 0.615487 | pred_wait_2_detect_1_form1_stage_2_cancers_1_posrisk_8_negrisk_5 | Patients | hypothetical |
| 0.091829 | 0.304665 | 0.643274 | pred_wait_2_detect_1_form1_stage_2_cancers_5_posrisk_8_negrisk_5 | Patients | hypothetical |
| 0.095875 | 0.319858 | 0.679174 | pred_wait_2_detect_1_form1_stage_2_cancers_10_posrisk_8_negrisk_5 | Patients | hypothetical |
| 0.117168 | 0.376655 | 0.774488 | pred_wait_2_detect_1_form1_stage_2_cancers_25_posrisk_8_negrisk_5 | Patients | hypothetical |
| 0.089238 | 0.289556 | 0.60839 | pred_wait_2_detect_1_form1_stage_2_cancers_1_posrisk_8_negrisk_10 | Patients | hypothetical |
| 0.091829 | 0.299954 | 0.636821 | pred_wait_2_detect_1_form1_stage_2_cancers_5_posrisk_8_negrisk_10 | Patients | hypothetical |
| 0.095875 | 0.315062 | 0.673792 | pred_wait_2_detect_1_form1_stage_2_cancers_10_posrisk_8_negrisk_10 | Patients | hypothetical |
| 0.117168 | 0.372347 | 0.772624 | pred_wait_2_detect_1_form1_stage_2_cancers_25_posrisk_8_negrisk_10 | Patients | hypothetical |
| 0.089238 | 0.2708 | 0.555857 | pred_wait_2_detect_1_form1_stage_2_cancers_1_posrisk_8_negrisk_40 | Patients | hypothetical |
| 0.091829 | 0.279935 | 0.579561 | pred_wait_2_detect_1_form1_stage_2_cancers_5_posrisk_8_negrisk_40 | Patients | hypothetical |
| 0.095875 | 0.293398 | 0.611993 | pred_wait_2_detect_1_form1_stage_2_cancers_10_posrisk_8_negrisk_40 | Patients | hypothetical |
| 0.117168 | 0.34658 | 0.707796 | pred_wait_2_detect_1_form1_stage_2_cancers_25_posrisk_8_negrisk_40 | Patients | hypothetical |
| 0.177685 | 0.402604 | 0.630238 | pred_wait_2_detect_1_form2_stage_1_cancers_1_posrisk_2_negrisk_1 | Patients | hypothetical |
| 0.182846 | 0.418716 | 0.657271 | pred_wait_2_detect_1_form2_stage_1_cancers_5_posrisk_2_negrisk_1 | Patients | hypothetical |
| 0.191994 | 0.441408 | 0.692158 | pred_wait_2_detect_1_form2_stage_1_cancers_10_posrisk_2_negrisk_1 | Patients | hypothetical |
| 0.246904 | 0.52136 | 0.783577 | pred_wait_2_detect_1_form2_stage_1_cancers_25_posrisk_2_negrisk_1 | Patients | hypothetical |
| 0.172999 | 0.39881 | 0.628372 | pred_wait_2_detect_1_form2_stage_1_cancers_1_posrisk_2_negrisk_5 | Patients | hypothetical |
| 0.177665 | 0.414849 | 0.655619 | pred_wait_2_detect_1_form2_stage_1_cancers_5_posrisk_2_negrisk_5 | Patients | hypothetical |
| 0.186554 | 0.437535 | 0.690041 | pred_wait_2_detect_1_form2_stage_1_cancers_10_posrisk_2_negrisk_5 | Patients | hypothetical |
| 0.240509 | 0.518117 | 0.783733 | pred_wait_2_detect_1_form2_stage_1_cancers_25_posrisk_2_negrisk_5 | Patients | hypothetical |
| 0.166163 | 0.394238 | 0.625924 | pred_wait_2_detect_1_form2_stage_1_cancers_1_posrisk_2_negrisk_10 | Patients | hypothetical |
| 0.169755 | 0.410138 | 0.652845 | pred_wait_2_detect_1_form2_stage_1_cancers_5_posrisk_2_negrisk_10 | Patients | hypothetical |
| 0.177252 | 0.432739 | 0.688226 | pred_wait_2_detect_1_form2_stage_1_cancers_10_posrisk_2_negrisk_10 | Patients | hypothetical |
| 0.228034 | 0.513809 | 0.782569 | pred_wait_2_detect_1_form2_stage_1_cancers_25_posrisk_2_negrisk_10 | Patients | hypothetical |
| 0.147697 | 0.375481 | 0.619559 | pred_wait_2_detect_1_form2_stage_1_cancers_1_posrisk_2_negrisk_40 | Patients | hypothetical |
| 0.150532 | 0.39012 | 0.646715 | pred_wait_2_detect_1_form2_stage_1_cancers_5_posrisk_2_negrisk_40 | Patients | hypothetical |
| 0.155941 | 0.411075 | 0.681777 | pred_wait_2_detect_1_form2_stage_1_cancers_10_posrisk_2_negrisk_40 | Patients | hypothetical |
| 0.198626 | 0.488042 | 0.774579 | pred_wait_2_detect_1_form2_stage_1_cancers_25_posrisk_2_negrisk_40 | Patients | hypothetical |
| 0.110715 | 0.343384 | 0.620335 | pred_wait_2_detect_1_form2_stage_1_cancers_1_posrisk_4_negrisk_1 | Patients | hypothetical |
| 0.111862 | 0.356254 | 0.647194 | pred_wait_2_detect_1_form2_stage_1_cancers_5_posrisk_4_negrisk_1 | Patients | hypothetical |
| 0.115167 | 0.374947 | 0.681878 | pred_wait_2_detect_1_form2_stage_1_cancers_10_posrisk_4_negrisk_1 | Patients | hypothetical |
| 0.147273 | 0.446215 | 0.774045 | pred_wait_2_detect_1_form2_stage_1_cancers_25_posrisk_4_negrisk_1 | Patients | hypothetical |
| 0.110715 | 0.33959 | 0.615487 | pred_wait_2_detect_1_form2_stage_1_cancers_1_posrisk_4_negrisk_5 | Patients | hypothetical |
| 0.111862 | 0.352387 | 0.643274 | pred_wait_2_detect_1_form2_stage_1_cancers_5_posrisk_4_negrisk_5 | Patients | hypothetical |
| 0.115167 | 0.371075 | 0.679174 | pred_wait_2_detect_1_form2_stage_1_cancers_10_posrisk_4_negrisk_5 | Patients | hypothetical |
| 0.147273 | 0.442972 | 0.774488 | pred_wait_2_detect_1_form2_stage_1_cancers_25_posrisk_4_negrisk_5 | Patients | hypothetical |
| 0.110715 | 0.335018 | 0.60839 | pred_wait_2_detect_1_form2_stage_1_cancers_1_posrisk_4_negrisk_10 | Patients | hypothetical |
| 0.111862 | 0.347676 | 0.636821 | pred_wait_2_detect_1_form2_stage_1_cancers_5_posrisk_4_negrisk_10 | Patients | hypothetical |
| 0.115167 | 0.366279 | 0.673792 | pred_wait_2_detect_1_form2_stage_1_cancers_10_posrisk_4_negrisk_10 | Patients | hypothetical |
| 0.147273 | 0.438664 | 0.772624 | pred_wait_2_detect_1_form2_stage_1_cancers_25_posrisk_4_negrisk_10 | Patients | hypothetical |
| 0.110618 | 0.316262 | 0.555857 | pred_wait_2_detect_1_form2_stage_1_cancers_1_posrisk_4_negrisk_40 | Patients | hypothetical |
| 0.111767 | 0.327657 | 0.579561 | pred_wait_2_detect_1_form2_stage_1_cancers_5_posrisk_4_negrisk_40 | Patients | hypothetical |
| 0.115153 | 0.344615 | 0.611993 | pred_wait_2_detect_1_form2_stage_1_cancers_10_posrisk_4_negrisk_40 | Patients | hypothetical |
| 0.147273 | 0.412897 | 0.707796 | pred_wait_2_detect_1_form2_stage_1_cancers_25_posrisk_4_negrisk_40 | Patients | hypothetical |
| 0.089891 | 0.31147 | 0.620335 | pred_wait_2_detect_1_form2_stage_1_cancers_1_posrisk_6_negrisk_1 | Patients | hypothetical |
| 0.091778 | 0.322679 | 0.647194 | pred_wait_2_detect_1_form2_stage_1_cancers_5_posrisk_6_negrisk_1 | Patients | hypothetical |
| 0.095358 | 0.338942 | 0.681878 | pred_wait_2_detect_1_form2_stage_1_cancers_10_posrisk_6_negrisk_1 | Patients | hypothetical |
| 0.120116 | 0.40078 | 0.774045 | pred_wait_2_detect_1_form2_stage_1_cancers_25_posrisk_6_negrisk_1 | Patients | hypothetical |
| 0.089891 | 0.307676 | 0.615487 | pred_wait_2_detect_1_form2_stage_1_cancers_1_posrisk_6_negrisk_5 | Patients | hypothetical |
| 0.091778 | 0.318812 | 0.643274 | pred_wait_2_detect_1_form2_stage_1_cancers_5_posrisk_6_negrisk_5 | Patients | hypothetical |
| 0.095358 | 0.335069 | 0.679174 | pred_wait_2_detect_1_form2_stage_1_cancers_10_posrisk_6_negrisk_5 | Patients | hypothetical |
| 0.120116 | 0.397537 | 0.774488 | pred_wait_2_detect_1_form2_stage_1_cancers_25_posrisk_6_negrisk_5 | Patients | hypothetical |
| 0.089891 | 0.303104 | 0.60839 | pred_wait_2_detect_1_form2_stage_1_cancers_1_posrisk_6_negrisk_10 | Patients | hypothetical |
| 0.091778 | 0.314101 | 0.636821 | pred_wait_2_detect_1_form2_stage_1_cancers_5_posrisk_6_negrisk_10 | Patients | hypothetical |
| 0.095358 | 0.330273 | 0.673792 | pred_wait_2_detect_1_form2_stage_1_cancers_10_posrisk_6_negrisk_10 | Patients | hypothetical |
| 0.120116 | 0.393229 | 0.772624 | pred_wait_2_detect_1_form2_stage_1_cancers_25_posrisk_6_negrisk_10 | Patients | hypothetical |
| 0.089891 | 0.284348 | 0.555857 | pred_wait_2_detect_1_form2_stage_1_cancers_1_posrisk_6_negrisk_40 | Patients | hypothetical |
| 0.091778 | 0.294083 | 0.579561 | pred_wait_2_detect_1_form2_stage_1_cancers_5_posrisk_6_negrisk_40 | Patients | hypothetical |
| 0.095358 | 0.308609 | 0.611993 | pred_wait_2_detect_1_form2_stage_1_cancers_10_posrisk_6_negrisk_40 | Patients | hypothetical |
| 0.120116 | 0.367462 | 0.707796 | pred_wait_2_detect_1_form2_stage_1_cancers_25_posrisk_6_negrisk_40 | Patients | hypothetical |
| 0.089238 | 0.297922 | 0.620335 | pred_wait_2_detect_1_form2_stage_1_cancers_1_posrisk_8_negrisk_1 | Patients | hypothetical |
| 0.091829 | 0.308532 | 0.647194 | pred_wait_2_detect_1_form2_stage_1_cancers_5_posrisk_8_negrisk_1 | Patients | hypothetical |
| 0.095875 | 0.323731 | 0.681878 | pred_wait_2_detect_1_form2_stage_1_cancers_10_posrisk_8_negrisk_1 | Patients | hypothetical |
| 0.117168 | 0.379898 | 0.774045 | pred_wait_2_detect_1_form2_stage_1_cancers_25_posrisk_8_negrisk_1 | Patients | hypothetical |
| 0.089238 | 0.294128 | 0.615487 | pred_wait_2_detect_1_form2_stage_1_cancers_1_posrisk_8_negrisk_5 | Patients | hypothetical |
| 0.091829 | 0.304665 | 0.643274 | pred_wait_2_detect_1_form2_stage_1_cancers_5_posrisk_8_negrisk_5 | Patients | hypothetical |
| 0.095875 | 0.319858 | 0.679174 | pred_wait_2_detect_1_form2_stage_1_cancers_10_posrisk_8_negrisk_5 | Patients | hypothetical |
| 0.117168 | 0.376655 | 0.774488 | pred_wait_2_detect_1_form2_stage_1_cancers_25_posrisk_8_negrisk_5 | Patients | hypothetical |
| 0.089238 | 0.289556 | 0.60839 | pred_wait_2_detect_1_form2_stage_1_cancers_1_posrisk_8_negrisk_10 | Patients | hypothetical |
| 0.091829 | 0.299954 | 0.636821 | pred_wait_2_detect_1_form2_stage_1_cancers_5_posrisk_8_negrisk_10 | Patients | hypothetical |
| 0.095875 | 0.315062 | 0.673792 | pred_wait_2_detect_1_form2_stage_1_cancers_10_posrisk_8_negrisk_10 | Patients | hypothetical |
| 0.117168 | 0.372347 | 0.772624 | pred_wait_2_detect_1_form2_stage_1_cancers_25_posrisk_8_negrisk_10 | Patients | hypothetical |
| 0.089238 | 0.2708 | 0.555857 | pred_wait_2_detect_1_form2_stage_1_cancers_1_posrisk_8_negrisk_40 | Patients | hypothetical |
| 0.091829 | 0.279935 | 0.579561 | pred_wait_2_detect_1_form2_stage_1_cancers_5_posrisk_8_negrisk_40 | Patients | hypothetical |
| 0.095875 | 0.293398 | 0.611993 | pred_wait_2_detect_1_form2_stage_1_cancers_10_posrisk_8_negrisk_40 | Patients | hypothetical |
| 0.117168 | 0.34658 | 0.707796 | pred_wait_2_detect_1_form2_stage_1_cancers_25_posrisk_8_negrisk_40 | Patients | hypothetical |
| 0.177685 | 0.402604 | 0.630238 | pred_wait_2_detect_1_form2_stage_2_cancers_1_posrisk_2_negrisk_1 | Patients | hypothetical |
| 0.182846 | 0.418716 | 0.657271 | pred_wait_2_detect_1_form2_stage_2_cancers_5_posrisk_2_negrisk_1 | Patients | hypothetical |
| 0.191994 | 0.441408 | 0.692158 | pred_wait_2_detect_1_form2_stage_2_cancers_10_posrisk_2_negrisk_1 | Patients | hypothetical |
| 0.246904 | 0.52136 | 0.783577 | pred_wait_2_detect_1_form2_stage_2_cancers_25_posrisk_2_negrisk_1 | Patients | hypothetical |
| 0.172999 | 0.39881 | 0.628372 | pred_wait_2_detect_1_form2_stage_2_cancers_1_posrisk_2_negrisk_5 | Patients | hypothetical |
| 0.177665 | 0.414849 | 0.655619 | pred_wait_2_detect_1_form2_stage_2_cancers_5_posrisk_2_negrisk_5 | Patients | hypothetical |
| 0.186554 | 0.437535 | 0.690041 | pred_wait_2_detect_1_form2_stage_2_cancers_10_posrisk_2_negrisk_5 | Patients | hypothetical |
| 0.240509 | 0.518117 | 0.783733 | pred_wait_2_detect_1_form2_stage_2_cancers_25_posrisk_2_negrisk_5 | Patients | hypothetical |
| 0.166163 | 0.394238 | 0.625924 | pred_wait_2_detect_1_form2_stage_2_cancers_1_posrisk_2_negrisk_10 | Patients | hypothetical |
| 0.169755 | 0.410138 | 0.652845 | pred_wait_2_detect_1_form2_stage_2_cancers_5_posrisk_2_negrisk_10 | Patients | hypothetical |
| 0.177252 | 0.432739 | 0.688226 | pred_wait_2_detect_1_form2_stage_2_cancers_10_posrisk_2_negrisk_10 | Patients | hypothetical |
| 0.228034 | 0.513809 | 0.782569 | pred_wait_2_detect_1_form2_stage_2_cancers_25_posrisk_2_negrisk_10 | Patients | hypothetical |
| 0.147697 | 0.375481 | 0.619559 | pred_wait_2_detect_1_form2_stage_2_cancers_1_posrisk_2_negrisk_40 | Patients | hypothetical |
| 0.150532 | 0.39012 | 0.646715 | pred_wait_2_detect_1_form2_stage_2_cancers_5_posrisk_2_negrisk_40 | Patients | hypothetical |
| 0.155941 | 0.411075 | 0.681777 | pred_wait_2_detect_1_form2_stage_2_cancers_10_posrisk_2_negrisk_40 | Patients | hypothetical |
| 0.198626 | 0.488042 | 0.774579 | pred_wait_2_detect_1_form2_stage_2_cancers_25_posrisk_2_negrisk_40 | Patients | hypothetical |
| 0.110715 | 0.343384 | 0.620335 | pred_wait_2_detect_1_form2_stage_2_cancers_1_posrisk_4_negrisk_1 | Patients | hypothetical |
| 0.111862 | 0.356254 | 0.647194 | pred_wait_2_detect_1_form2_stage_2_cancers_5_posrisk_4_negrisk_1 | Patients | hypothetical |
| 0.115167 | 0.374947 | 0.681878 | pred_wait_2_detect_1_form2_stage_2_cancers_10_posrisk_4_negrisk_1 | Patients | hypothetical |
| 0.147273 | 0.446215 | 0.774045 | pred_wait_2_detect_1_form2_stage_2_cancers_25_posrisk_4_negrisk_1 | Patients | hypothetical |
| 0.110715 | 0.33959 | 0.615487 | pred_wait_2_detect_1_form2_stage_2_cancers_1_posrisk_4_negrisk_5 | Patients | hypothetical |
| 0.111862 | 0.352387 | 0.643274 | pred_wait_2_detect_1_form2_stage_2_cancers_5_posrisk_4_negrisk_5 | Patients | hypothetical |
| 0.115167 | 0.371075 | 0.679174 | pred_wait_2_detect_1_form2_stage_2_cancers_10_posrisk_4_negrisk_5 | Patients | hypothetical |
| 0.147273 | 0.442972 | 0.774488 | pred_wait_2_detect_1_form2_stage_2_cancers_25_posrisk_4_negrisk_5 | Patients | hypothetical |
| 0.110715 | 0.335018 | 0.60839 | pred_wait_2_detect_1_form2_stage_2_cancers_1_posrisk_4_negrisk_10 | Patients | hypothetical |
| 0.111862 | 0.347676 | 0.636821 | pred_wait_2_detect_1_form2_stage_2_cancers_5_posrisk_4_negrisk_10 | Patients | hypothetical |
| 0.115167 | 0.366279 | 0.673792 | pred_wait_2_detect_1_form2_stage_2_cancers_10_posrisk_4_negrisk_10 | Patients | hypothetical |
| 0.147273 | 0.438664 | 0.772624 | pred_wait_2_detect_1_form2_stage_2_cancers_25_posrisk_4_negrisk_10 | Patients | hypothetical |
| 0.110618 | 0.316262 | 0.555857 | pred_wait_2_detect_1_form2_stage_2_cancers_1_posrisk_4_negrisk_40 | Patients | hypothetical |
| 0.111767 | 0.327657 | 0.579561 | pred_wait_2_detect_1_form2_stage_2_cancers_5_posrisk_4_negrisk_40 | Patients | hypothetical |
| 0.115153 | 0.344615 | 0.611993 | pred_wait_2_detect_1_form2_stage_2_cancers_10_posrisk_4_negrisk_40 | Patients | hypothetical |
| 0.147273 | 0.412897 | 0.707796 | pred_wait_2_detect_1_form2_stage_2_cancers_25_posrisk_4_negrisk_40 | Patients | hypothetical |
| 0.089891 | 0.31147 | 0.620335 | pred_wait_2_detect_1_form2_stage_2_cancers_1_posrisk_6_negrisk_1 | Patients | hypothetical |
| 0.091778 | 0.322679 | 0.647194 | pred_wait_2_detect_1_form2_stage_2_cancers_5_posrisk_6_negrisk_1 | Patients | hypothetical |
| 0.095358 | 0.338942 | 0.681878 | pred_wait_2_detect_1_form2_stage_2_cancers_10_posrisk_6_negrisk_1 | Patients | hypothetical |
| 0.120116 | 0.40078 | 0.774045 | pred_wait_2_detect_1_form2_stage_2_cancers_25_posrisk_6_negrisk_1 | Patients | hypothetical |
| 0.089891 | 0.307676 | 0.615487 | pred_wait_2_detect_1_form2_stage_2_cancers_1_posrisk_6_negrisk_5 | Patients | hypothetical |
| 0.091778 | 0.318812 | 0.643274 | pred_wait_2_detect_1_form2_stage_2_cancers_5_posrisk_6_negrisk_5 | Patients | hypothetical |
| 0.095358 | 0.335069 | 0.679174 | pred_wait_2_detect_1_form2_stage_2_cancers_10_posrisk_6_negrisk_5 | Patients | hypothetical |
| 0.120116 | 0.397537 | 0.774488 | pred_wait_2_detect_1_form2_stage_2_cancers_25_posrisk_6_negrisk_5 | Patients | hypothetical |
| 0.089891 | 0.303104 | 0.60839 | pred_wait_2_detect_1_form2_stage_2_cancers_1_posrisk_6_negrisk_10 | Patients | hypothetical |
| 0.091778 | 0.314101 | 0.636821 | pred_wait_2_detect_1_form2_stage_2_cancers_5_posrisk_6_negrisk_10 | Patients | hypothetical |
| 0.095358 | 0.330273 | 0.673792 | pred_wait_2_detect_1_form2_stage_2_cancers_10_posrisk_6_negrisk_10 | Patients | hypothetical |
| 0.120116 | 0.393229 | 0.772624 | pred_wait_2_detect_1_form2_stage_2_cancers_25_posrisk_6_negrisk_10 | Patients | hypothetical |
| 0.089891 | 0.284348 | 0.555857 | pred_wait_2_detect_1_form2_stage_2_cancers_1_posrisk_6_negrisk_40 | Patients | hypothetical |
| 0.091778 | 0.294083 | 0.579561 | pred_wait_2_detect_1_form2_stage_2_cancers_5_posrisk_6_negrisk_40 | Patients | hypothetical |
| 0.095358 | 0.308609 | 0.611993 | pred_wait_2_detect_1_form2_stage_2_cancers_10_posrisk_6_negrisk_40 | Patients | hypothetical |
| 0.120116 | 0.367462 | 0.707796 | pred_wait_2_detect_1_form2_stage_2_cancers_25_posrisk_6_negrisk_40 | Patients | hypothetical |
| 0.089238 | 0.297922 | 0.620335 | pred_wait_2_detect_1_form2_stage_2_cancers_1_posrisk_8_negrisk_1 | Patients | hypothetical |
| 0.091829 | 0.308532 | 0.647194 | pred_wait_2_detect_1_form2_stage_2_cancers_5_posrisk_8_negrisk_1 | Patients | hypothetical |
| 0.095875 | 0.323731 | 0.681878 | pred_wait_2_detect_1_form2_stage_2_cancers_10_posrisk_8_negrisk_1 | Patients | hypothetical |
| 0.117168 | 0.379898 | 0.774045 | pred_wait_2_detect_1_form2_stage_2_cancers_25_posrisk_8_negrisk_1 | Patients | hypothetical |
| 0.089238 | 0.294128 | 0.615487 | pred_wait_2_detect_1_form2_stage_2_cancers_1_posrisk_8_negrisk_5 | Patients | hypothetical |
| 0.091829 | 0.304665 | 0.643274 | pred_wait_2_detect_1_form2_stage_2_cancers_5_posrisk_8_negrisk_5 | Patients | hypothetical |
| 0.095875 | 0.319858 | 0.679174 | pred_wait_2_detect_1_form2_stage_2_cancers_10_posrisk_8_negrisk_5 | Patients | hypothetical |
| 0.117168 | 0.376655 | 0.774488 | pred_wait_2_detect_1_form2_stage_2_cancers_25_posrisk_8_negrisk_5 | Patients | hypothetical |
| 0.089238 | 0.289556 | 0.60839 | pred_wait_2_detect_1_form2_stage_2_cancers_1_posrisk_8_negrisk_10 | Patients | hypothetical |
| 0.091829 | 0.299954 | 0.636821 | pred_wait_2_detect_1_form2_stage_2_cancers_5_posrisk_8_negrisk_10 | Patients | hypothetical |
| 0.095875 | 0.315062 | 0.673792 | pred_wait_2_detect_1_form2_stage_2_cancers_10_posrisk_8_negrisk_10 | Patients | hypothetical |
| 0.117168 | 0.372347 | 0.772624 | pred_wait_2_detect_1_form2_stage_2_cancers_25_posrisk_8_negrisk_10 | Patients | hypothetical |
| 0.089238 | 0.2708 | 0.555857 | pred_wait_2_detect_1_form2_stage_2_cancers_1_posrisk_8_negrisk_40 | Patients | hypothetical |
| 0.091829 | 0.279935 | 0.579561 | pred_wait_2_detect_1_form2_stage_2_cancers_5_posrisk_8_negrisk_40 | Patients | hypothetical |
| 0.095875 | 0.293398 | 0.611993 | pred_wait_2_detect_1_form2_stage_2_cancers_10_posrisk_8_negrisk_40 | Patients | hypothetical |
| 0.117168 | 0.34658 | 0.707796 | pred_wait_2_detect_1_form2_stage_2_cancers_25_posrisk_8_negrisk_40 | Patients | hypothetical |
| 0.177685 | 0.402604 | 0.630238 | pred_wait_2_detect_1_form3_stage_1_cancers_1_posrisk_2_negrisk_1 | Patients | hypothetical |
| 0.182846 | 0.418716 | 0.657271 | pred_wait_2_detect_1_form3_stage_1_cancers_5_posrisk_2_negrisk_1 | Patients | hypothetical |
| 0.191994 | 0.441408 | 0.692158 | pred_wait_2_detect_1_form3_stage_1_cancers_10_posrisk_2_negrisk_1 | Patients | hypothetical |
| 0.246904 | 0.52136 | 0.783577 | pred_wait_2_detect_1_form3_stage_1_cancers_25_posrisk_2_negrisk_1 | Patients | hypothetical |
| 0.172999 | 0.39881 | 0.628372 | pred_wait_2_detect_1_form3_stage_1_cancers_1_posrisk_2_negrisk_5 | Patients | hypothetical |
| 0.177665 | 0.414849 | 0.655619 | pred_wait_2_detect_1_form3_stage_1_cancers_5_posrisk_2_negrisk_5 | Patients | hypothetical |
| 0.186554 | 0.437535 | 0.690041 | pred_wait_2_detect_1_form3_stage_1_cancers_10_posrisk_2_negrisk_5 | Patients | hypothetical |
| 0.240509 | 0.518117 | 0.783733 | pred_wait_2_detect_1_form3_stage_1_cancers_25_posrisk_2_negrisk_5 | Patients | hypothetical |
| 0.166163 | 0.394238 | 0.625924 | pred_wait_2_detect_1_form3_stage_1_cancers_1_posrisk_2_negrisk_10 | Patients | hypothetical |
| 0.169755 | 0.410138 | 0.652845 | pred_wait_2_detect_1_form3_stage_1_cancers_5_posrisk_2_negrisk_10 | Patients | hypothetical |
| 0.177252 | 0.432739 | 0.688226 | pred_wait_2_detect_1_form3_stage_1_cancers_10_posrisk_2_negrisk_10 | Patients | hypothetical |
| 0.228034 | 0.513809 | 0.782569 | pred_wait_2_detect_1_form3_stage_1_cancers_25_posrisk_2_negrisk_10 | Patients | hypothetical |
| 0.147697 | 0.375481 | 0.619559 | pred_wait_2_detect_1_form3_stage_1_cancers_1_posrisk_2_negrisk_40 | Patients | hypothetical |
| 0.150532 | 0.39012 | 0.646715 | pred_wait_2_detect_1_form3_stage_1_cancers_5_posrisk_2_negrisk_40 | Patients | hypothetical |
| 0.155941 | 0.411075 | 0.681777 | pred_wait_2_detect_1_form3_stage_1_cancers_10_posrisk_2_negrisk_40 | Patients | hypothetical |
| 0.198626 | 0.488042 | 0.774579 | pred_wait_2_detect_1_form3_stage_1_cancers_25_posrisk_2_negrisk_40 | Patients | hypothetical |
| 0.110715 | 0.343384 | 0.620335 | pred_wait_2_detect_1_form3_stage_1_cancers_1_posrisk_4_negrisk_1 | Patients | hypothetical |
| 0.111862 | 0.356254 | 0.647194 | pred_wait_2_detect_1_form3_stage_1_cancers_5_posrisk_4_negrisk_1 | Patients | hypothetical |
| 0.115167 | 0.374947 | 0.681878 | pred_wait_2_detect_1_form3_stage_1_cancers_10_posrisk_4_negrisk_1 | Patients | hypothetical |
| 0.147273 | 0.446215 | 0.774045 | pred_wait_2_detect_1_form3_stage_1_cancers_25_posrisk_4_negrisk_1 | Patients | hypothetical |
| 0.110715 | 0.33959 | 0.615487 | pred_wait_2_detect_1_form3_stage_1_cancers_1_posrisk_4_negrisk_5 | Patients | hypothetical |
| 0.111862 | 0.352387 | 0.643274 | pred_wait_2_detect_1_form3_stage_1_cancers_5_posrisk_4_negrisk_5 | Patients | hypothetical |
| 0.115167 | 0.371075 | 0.679174 | pred_wait_2_detect_1_form3_stage_1_cancers_10_posrisk_4_negrisk_5 | Patients | hypothetical |
| 0.147273 | 0.442972 | 0.774488 | pred_wait_2_detect_1_form3_stage_1_cancers_25_posrisk_4_negrisk_5 | Patients | hypothetical |
| 0.110715 | 0.335018 | 0.60839 | pred_wait_2_detect_1_form3_stage_1_cancers_1_posrisk_4_negrisk_10 | Patients | hypothetical |
| 0.111862 | 0.347676 | 0.636821 | pred_wait_2_detect_1_form3_stage_1_cancers_5_posrisk_4_negrisk_10 | Patients | hypothetical |
| 0.115167 | 0.366279 | 0.673792 | pred_wait_2_detect_1_form3_stage_1_cancers_10_posrisk_4_negrisk_10 | Patients | hypothetical |
| 0.147273 | 0.438664 | 0.772624 | pred_wait_2_detect_1_form3_stage_1_cancers_25_posrisk_4_negrisk_10 | Patients | hypothetical |
| 0.110618 | 0.316262 | 0.555857 | pred_wait_2_detect_1_form3_stage_1_cancers_1_posrisk_4_negrisk_40 | Patients | hypothetical |
| 0.111767 | 0.327657 | 0.579561 | pred_wait_2_detect_1_form3_stage_1_cancers_5_posrisk_4_negrisk_40 | Patients | hypothetical |
| 0.115153 | 0.344615 | 0.611993 | pred_wait_2_detect_1_form3_stage_1_cancers_10_posrisk_4_negrisk_40 | Patients | hypothetical |
| 0.147273 | 0.412897 | 0.707796 | pred_wait_2_detect_1_form3_stage_1_cancers_25_posrisk_4_negrisk_40 | Patients | hypothetical |
| 0.089891 | 0.31147 | 0.620335 | pred_wait_2_detect_1_form3_stage_1_cancers_1_posrisk_6_negrisk_1 | Patients | hypothetical |
| 0.091778 | 0.322679 | 0.647194 | pred_wait_2_detect_1_form3_stage_1_cancers_5_posrisk_6_negrisk_1 | Patients | hypothetical |
| 0.095358 | 0.338942 | 0.681878 | pred_wait_2_detect_1_form3_stage_1_cancers_10_posrisk_6_negrisk_1 | Patients | hypothetical |
| 0.120116 | 0.40078 | 0.774045 | pred_wait_2_detect_1_form3_stage_1_cancers_25_posrisk_6_negrisk_1 | Patients | hypothetical |
| 0.089891 | 0.307676 | 0.615487 | pred_wait_2_detect_1_form3_stage_1_cancers_1_posrisk_6_negrisk_5 | Patients | hypothetical |
| 0.091778 | 0.318812 | 0.643274 | pred_wait_2_detect_1_form3_stage_1_cancers_5_posrisk_6_negrisk_5 | Patients | hypothetical |
| 0.095358 | 0.335069 | 0.679174 | pred_wait_2_detect_1_form3_stage_1_cancers_10_posrisk_6_negrisk_5 | Patients | hypothetical |
| 0.120116 | 0.397537 | 0.774488 | pred_wait_2_detect_1_form3_stage_1_cancers_25_posrisk_6_negrisk_5 | Patients | hypothetical |
| 0.089891 | 0.303104 | 0.60839 | pred_wait_2_detect_1_form3_stage_1_cancers_1_posrisk_6_negrisk_10 | Patients | hypothetical |
| 0.091778 | 0.314101 | 0.636821 | pred_wait_2_detect_1_form3_stage_1_cancers_5_posrisk_6_negrisk_10 | Patients | hypothetical |
| 0.095358 | 0.330273 | 0.673792 | pred_wait_2_detect_1_form3_stage_1_cancers_10_posrisk_6_negrisk_10 | Patients | hypothetical |
| 0.120116 | 0.393229 | 0.772624 | pred_wait_2_detect_1_form3_stage_1_cancers_25_posrisk_6_negrisk_10 | Patients | hypothetical |
| 0.089891 | 0.284348 | 0.555857 | pred_wait_2_detect_1_form3_stage_1_cancers_1_posrisk_6_negrisk_40 | Patients | hypothetical |
| 0.091778 | 0.294083 | 0.579561 | pred_wait_2_detect_1_form3_stage_1_cancers_5_posrisk_6_negrisk_40 | Patients | hypothetical |
| 0.095358 | 0.308609 | 0.611993 | pred_wait_2_detect_1_form3_stage_1_cancers_10_posrisk_6_negrisk_40 | Patients | hypothetical |
| 0.120116 | 0.367462 | 0.707796 | pred_wait_2_detect_1_form3_stage_1_cancers_25_posrisk_6_negrisk_40 | Patients | hypothetical |
| 0.089238 | 0.297922 | 0.620335 | pred_wait_2_detect_1_form3_stage_1_cancers_1_posrisk_8_negrisk_1 | Patients | hypothetical |
| 0.091829 | 0.308532 | 0.647194 | pred_wait_2_detect_1_form3_stage_1_cancers_5_posrisk_8_negrisk_1 | Patients | hypothetical |
| 0.095875 | 0.323731 | 0.681878 | pred_wait_2_detect_1_form3_stage_1_cancers_10_posrisk_8_negrisk_1 | Patients | hypothetical |
| 0.117168 | 0.379898 | 0.774045 | pred_wait_2_detect_1_form3_stage_1_cancers_25_posrisk_8_negrisk_1 | Patients | hypothetical |
| 0.089238 | 0.294128 | 0.615487 | pred_wait_2_detect_1_form3_stage_1_cancers_1_posrisk_8_negrisk_5 | Patients | hypothetical |
| 0.091829 | 0.304665 | 0.643274 | pred_wait_2_detect_1_form3_stage_1_cancers_5_posrisk_8_negrisk_5 | Patients | hypothetical |
| 0.095875 | 0.319858 | 0.679174 | pred_wait_2_detect_1_form3_stage_1_cancers_10_posrisk_8_negrisk_5 | Patients | hypothetical |
| 0.117168 | 0.376655 | 0.774488 | pred_wait_2_detect_1_form3_stage_1_cancers_25_posrisk_8_negrisk_5 | Patients | hypothetical |
| 0.089238 | 0.289556 | 0.60839 | pred_wait_2_detect_1_form3_stage_1_cancers_1_posrisk_8_negrisk_10 | Patients | hypothetical |
| 0.091829 | 0.299954 | 0.636821 | pred_wait_2_detect_1_form3_stage_1_cancers_5_posrisk_8_negrisk_10 | Patients | hypothetical |
| 0.095875 | 0.315062 | 0.673792 | pred_wait_2_detect_1_form3_stage_1_cancers_10_posrisk_8_negrisk_10 | Patients | hypothetical |
| 0.117168 | 0.372347 | 0.772624 | pred_wait_2_detect_1_form3_stage_1_cancers_25_posrisk_8_negrisk_10 | Patients | hypothetical |
| 0.089238 | 0.2708 | 0.555857 | pred_wait_2_detect_1_form3_stage_1_cancers_1_posrisk_8_negrisk_40 | Patients | hypothetical |
| 0.091829 | 0.279935 | 0.579561 | pred_wait_2_detect_1_form3_stage_1_cancers_5_posrisk_8_negrisk_40 | Patients | hypothetical |
| 0.095875 | 0.293398 | 0.611993 | pred_wait_2_detect_1_form3_stage_1_cancers_10_posrisk_8_negrisk_40 | Patients | hypothetical |
| 0.117168 | 0.34658 | 0.707796 | pred_wait_2_detect_1_form3_stage_1_cancers_25_posrisk_8_negrisk_40 | Patients | hypothetical |
| 0.177685 | 0.402604 | 0.630238 | pred_wait_2_detect_1_form3_stage_2_cancers_1_posrisk_2_negrisk_1 | Patients | hypothetical |
| 0.182846 | 0.418716 | 0.657271 | pred_wait_2_detect_1_form3_stage_2_cancers_5_posrisk_2_negrisk_1 | Patients | hypothetical |
| 0.191994 | 0.441408 | 0.692158 | pred_wait_2_detect_1_form3_stage_2_cancers_10_posrisk_2_negrisk_1 | Patients | hypothetical |
| 0.246904 | 0.52136 | 0.783577 | pred_wait_2_detect_1_form3_stage_2_cancers_25_posrisk_2_negrisk_1 | Patients | hypothetical |
| 0.172999 | 0.39881 | 0.628372 | pred_wait_2_detect_1_form3_stage_2_cancers_1_posrisk_2_negrisk_5 | Patients | hypothetical |
| 0.177665 | 0.414849 | 0.655619 | pred_wait_2_detect_1_form3_stage_2_cancers_5_posrisk_2_negrisk_5 | Patients | hypothetical |
| 0.186554 | 0.437535 | 0.690041 | pred_wait_2_detect_1_form3_stage_2_cancers_10_posrisk_2_negrisk_5 | Patients | hypothetical |
| 0.240509 | 0.518117 | 0.783733 | pred_wait_2_detect_1_form3_stage_2_cancers_25_posrisk_2_negrisk_5 | Patients | hypothetical |
| 0.166163 | 0.394238 | 0.625924 | pred_wait_2_detect_1_form3_stage_2_cancers_1_posrisk_2_negrisk_10 | Patients | hypothetical |
| 0.169755 | 0.410138 | 0.652845 | pred_wait_2_detect_1_form3_stage_2_cancers_5_posrisk_2_negrisk_10 | Patients | hypothetical |
| 0.177252 | 0.432739 | 0.688226 | pred_wait_2_detect_1_form3_stage_2_cancers_10_posrisk_2_negrisk_10 | Patients | hypothetical |
| 0.228034 | 0.513809 | 0.782569 | pred_wait_2_detect_1_form3_stage_2_cancers_25_posrisk_2_negrisk_10 | Patients | hypothetical |
| 0.147697 | 0.375481 | 0.619559 | pred_wait_2_detect_1_form3_stage_2_cancers_1_posrisk_2_negrisk_40 | Patients | hypothetical |
| 0.150532 | 0.39012 | 0.646715 | pred_wait_2_detect_1_form3_stage_2_cancers_5_posrisk_2_negrisk_40 | Patients | hypothetical |
| 0.155941 | 0.411075 | 0.681777 | pred_wait_2_detect_1_form3_stage_2_cancers_10_posrisk_2_negrisk_40 | Patients | hypothetical |
| 0.198626 | 0.488042 | 0.774579 | pred_wait_2_detect_1_form3_stage_2_cancers_25_posrisk_2_negrisk_40 | Patients | hypothetical |
| 0.110715 | 0.343384 | 0.620335 | pred_wait_2_detect_1_form3_stage_2_cancers_1_posrisk_4_negrisk_1 | Patients | hypothetical |
| 0.111862 | 0.356254 | 0.647194 | pred_wait_2_detect_1_form3_stage_2_cancers_5_posrisk_4_negrisk_1 | Patients | hypothetical |
| 0.115167 | 0.374947 | 0.681878 | pred_wait_2_detect_1_form3_stage_2_cancers_10_posrisk_4_negrisk_1 | Patients | hypothetical |
| 0.147273 | 0.446215 | 0.774045 | pred_wait_2_detect_1_form3_stage_2_cancers_25_posrisk_4_negrisk_1 | Patients | hypothetical |
| 0.110715 | 0.33959 | 0.615487 | pred_wait_2_detect_1_form3_stage_2_cancers_1_posrisk_4_negrisk_5 | Patients | hypothetical |
| 0.111862 | 0.352387 | 0.643274 | pred_wait_2_detect_1_form3_stage_2_cancers_5_posrisk_4_negrisk_5 | Patients | hypothetical |
| 0.115167 | 0.371075 | 0.679174 | pred_wait_2_detect_1_form3_stage_2_cancers_10_posrisk_4_negrisk_5 | Patients | hypothetical |
| 0.147273 | 0.442972 | 0.774488 | pred_wait_2_detect_1_form3_stage_2_cancers_25_posrisk_4_negrisk_5 | Patients | hypothetical |
| 0.110715 | 0.335018 | 0.60839 | pred_wait_2_detect_1_form3_stage_2_cancers_1_posrisk_4_negrisk_10 | Patients | hypothetical |
| 0.111862 | 0.347676 | 0.636821 | pred_wait_2_detect_1_form3_stage_2_cancers_5_posrisk_4_negrisk_10 | Patients | hypothetical |
| 0.115167 | 0.366279 | 0.673792 | pred_wait_2_detect_1_form3_stage_2_cancers_10_posrisk_4_negrisk_10 | Patients | hypothetical |
| 0.147273 | 0.438664 | 0.772624 | pred_wait_2_detect_1_form3_stage_2_cancers_25_posrisk_4_negrisk_10 | Patients | hypothetical |
| 0.110618 | 0.316262 | 0.555857 | pred_wait_2_detect_1_form3_stage_2_cancers_1_posrisk_4_negrisk_40 | Patients | hypothetical |
| 0.111767 | 0.327657 | 0.579561 | pred_wait_2_detect_1_form3_stage_2_cancers_5_posrisk_4_negrisk_40 | Patients | hypothetical |
| 0.115153 | 0.344615 | 0.611993 | pred_wait_2_detect_1_form3_stage_2_cancers_10_posrisk_4_negrisk_40 | Patients | hypothetical |
| 0.147273 | 0.412897 | 0.707796 | pred_wait_2_detect_1_form3_stage_2_cancers_25_posrisk_4_negrisk_40 | Patients | hypothetical |
| 0.089891 | 0.31147 | 0.620335 | pred_wait_2_detect_1_form3_stage_2_cancers_1_posrisk_6_negrisk_1 | Patients | hypothetical |
| 0.091778 | 0.322679 | 0.647194 | pred_wait_2_detect_1_form3_stage_2_cancers_5_posrisk_6_negrisk_1 | Patients | hypothetical |
| 0.095358 | 0.338942 | 0.681878 | pred_wait_2_detect_1_form3_stage_2_cancers_10_posrisk_6_negrisk_1 | Patients | hypothetical |
| 0.120116 | 0.40078 | 0.774045 | pred_wait_2_detect_1_form3_stage_2_cancers_25_posrisk_6_negrisk_1 | Patients | hypothetical |
| 0.089891 | 0.307676 | 0.615487 | pred_wait_2_detect_1_form3_stage_2_cancers_1_posrisk_6_negrisk_5 | Patients | hypothetical |
| 0.091778 | 0.318812 | 0.643274 | pred_wait_2_detect_1_form3_stage_2_cancers_5_posrisk_6_negrisk_5 | Patients | hypothetical |
| 0.095358 | 0.335069 | 0.679174 | pred_wait_2_detect_1_form3_stage_2_cancers_10_posrisk_6_negrisk_5 | Patients | hypothetical |
| 0.120116 | 0.397537 | 0.774488 | pred_wait_2_detect_1_form3_stage_2_cancers_25_posrisk_6_negrisk_5 | Patients | hypothetical |
| 0.089891 | 0.303104 | 0.60839 | pred_wait_2_detect_1_form3_stage_2_cancers_1_posrisk_6_negrisk_10 | Patients | hypothetical |
| 0.091778 | 0.314101 | 0.636821 | pred_wait_2_detect_1_form3_stage_2_cancers_5_posrisk_6_negrisk_10 | Patients | hypothetical |
| 0.095358 | 0.330273 | 0.673792 | pred_wait_2_detect_1_form3_stage_2_cancers_10_posrisk_6_negrisk_10 | Patients | hypothetical |
| 0.120116 | 0.393229 | 0.772624 | pred_wait_2_detect_1_form3_stage_2_cancers_25_posrisk_6_negrisk_10 | Patients | hypothetical |
| 0.089891 | 0.284348 | 0.555857 | pred_wait_2_detect_1_form3_stage_2_cancers_1_posrisk_6_negrisk_40 | Patients | hypothetical |
| 0.091778 | 0.294083 | 0.579561 | pred_wait_2_detect_1_form3_stage_2_cancers_5_posrisk_6_negrisk_40 | Patients | hypothetical |
| 0.095358 | 0.308609 | 0.611993 | pred_wait_2_detect_1_form3_stage_2_cancers_10_posrisk_6_negrisk_40 | Patients | hypothetical |
| 0.120116 | 0.367462 | 0.707796 | pred_wait_2_detect_1_form3_stage_2_cancers_25_posrisk_6_negrisk_40 | Patients | hypothetical |
| 0.089238 | 0.297922 | 0.620335 | pred_wait_2_detect_1_form3_stage_2_cancers_1_posrisk_8_negrisk_1 | Patients | hypothetical |
| 0.091829 | 0.308532 | 0.647194 | pred_wait_2_detect_1_form3_stage_2_cancers_5_posrisk_8_negrisk_1 | Patients | hypothetical |
| 0.095875 | 0.323731 | 0.681878 | pred_wait_2_detect_1_form3_stage_2_cancers_10_posrisk_8_negrisk_1 | Patients | hypothetical |
| 0.117168 | 0.379898 | 0.774045 | pred_wait_2_detect_1_form3_stage_2_cancers_25_posrisk_8_negrisk_1 | Patients | hypothetical |
| 0.089238 | 0.294128 | 0.615487 | pred_wait_2_detect_1_form3_stage_2_cancers_1_posrisk_8_negrisk_5 | Patients | hypothetical |
| 0.091829 | 0.304665 | 0.643274 | pred_wait_2_detect_1_form3_stage_2_cancers_5_posrisk_8_negrisk_5 | Patients | hypothetical |
| 0.095875 | 0.319858 | 0.679174 | pred_wait_2_detect_1_form3_stage_2_cancers_10_posrisk_8_negrisk_5 | Patients | hypothetical |
| 0.117168 | 0.376655 | 0.774488 | pred_wait_2_detect_1_form3_stage_2_cancers_25_posrisk_8_negrisk_5 | Patients | hypothetical |
| 0.089238 | 0.289556 | 0.60839 | pred_wait_2_detect_1_form3_stage_2_cancers_1_posrisk_8_negrisk_10 | Patients | hypothetical |
| 0.091829 | 0.299954 | 0.636821 | pred_wait_2_detect_1_form3_stage_2_cancers_5_posrisk_8_negrisk_10 | Patients | hypothetical |
| 0.095875 | 0.315062 | 0.673792 | pred_wait_2_detect_1_form3_stage_2_cancers_10_posrisk_8_negrisk_10 | Patients | hypothetical |
| 0.117168 | 0.372347 | 0.772624 | pred_wait_2_detect_1_form3_stage_2_cancers_25_posrisk_8_negrisk_10 | Patients | hypothetical |
| 0.089238 | 0.2708 | 0.555857 | pred_wait_2_detect_1_form3_stage_2_cancers_1_posrisk_8_negrisk_40 | Patients | hypothetical |
| 0.091829 | 0.279935 | 0.579561 | pred_wait_2_detect_1_form3_stage_2_cancers_5_posrisk_8_negrisk_40 | Patients | hypothetical |
| 0.095875 | 0.293398 | 0.611993 | pred_wait_2_detect_1_form3_stage_2_cancers_10_posrisk_8_negrisk_40 | Patients | hypothetical |
| 0.117168 | 0.34658 | 0.707796 | pred_wait_2_detect_1_form3_stage_2_cancers_25_posrisk_8_negrisk_40 | Patients | hypothetical |
| 0.177685 | 0.402604 | 0.630238 | pred_wait_2_detect_1_form4_stage_1_cancers_1_posrisk_2_negrisk_1 | Patients | hypothetical |
| 0.182846 | 0.418716 | 0.657271 | pred_wait_2_detect_1_form4_stage_1_cancers_5_posrisk_2_negrisk_1 | Patients | hypothetical |
| 0.191994 | 0.441408 | 0.692158 | pred_wait_2_detect_1_form4_stage_1_cancers_10_posrisk_2_negrisk_1 | Patients | hypothetical |
| 0.246904 | 0.52136 | 0.783577 | pred_wait_2_detect_1_form4_stage_1_cancers_25_posrisk_2_negrisk_1 | Patients | hypothetical |
| 0.172999 | 0.39881 | 0.628372 | pred_wait_2_detect_1_form4_stage_1_cancers_1_posrisk_2_negrisk_5 | Patients | hypothetical |
| 0.177665 | 0.414849 | 0.655619 | pred_wait_2_detect_1_form4_stage_1_cancers_5_posrisk_2_negrisk_5 | Patients | hypothetical |
| 0.186554 | 0.437535 | 0.690041 | pred_wait_2_detect_1_form4_stage_1_cancers_10_posrisk_2_negrisk_5 | Patients | hypothetical |
| 0.240509 | 0.518117 | 0.783733 | pred_wait_2_detect_1_form4_stage_1_cancers_25_posrisk_2_negrisk_5 | Patients | hypothetical |
| 0.166163 | 0.394238 | 0.625924 | pred_wait_2_detect_1_form4_stage_1_cancers_1_posrisk_2_negrisk_10 | Patients | hypothetical |
| 0.169755 | 0.410138 | 0.652845 | pred_wait_2_detect_1_form4_stage_1_cancers_5_posrisk_2_negrisk_10 | Patients | hypothetical |
| 0.177252 | 0.432739 | 0.688226 | pred_wait_2_detect_1_form4_stage_1_cancers_10_posrisk_2_negrisk_10 | Patients | hypothetical |
| 0.228034 | 0.513809 | 0.782569 | pred_wait_2_detect_1_form4_stage_1_cancers_25_posrisk_2_negrisk_10 | Patients | hypothetical |
| 0.147697 | 0.375481 | 0.619559 | pred_wait_2_detect_1_form4_stage_1_cancers_1_posrisk_2_negrisk_40 | Patients | hypothetical |
| 0.150532 | 0.39012 | 0.646715 | pred_wait_2_detect_1_form4_stage_1_cancers_5_posrisk_2_negrisk_40 | Patients | hypothetical |
| 0.155941 | 0.411075 | 0.681777 | pred_wait_2_detect_1_form4_stage_1_cancers_10_posrisk_2_negrisk_40 | Patients | hypothetical |
| 0.198626 | 0.488042 | 0.774579 | pred_wait_2_detect_1_form4_stage_1_cancers_25_posrisk_2_negrisk_40 | Patients | hypothetical |
| 0.110715 | 0.343384 | 0.620335 | pred_wait_2_detect_1_form4_stage_1_cancers_1_posrisk_4_negrisk_1 | Patients | hypothetical |
| 0.111862 | 0.356254 | 0.647194 | pred_wait_2_detect_1_form4_stage_1_cancers_5_posrisk_4_negrisk_1 | Patients | hypothetical |
| 0.115167 | 0.374947 | 0.681878 | pred_wait_2_detect_1_form4_stage_1_cancers_10_posrisk_4_negrisk_1 | Patients | hypothetical |
| 0.147273 | 0.446215 | 0.774045 | pred_wait_2_detect_1_form4_stage_1_cancers_25_posrisk_4_negrisk_1 | Patients | hypothetical |
| 0.110715 | 0.33959 | 0.615487 | pred_wait_2_detect_1_form4_stage_1_cancers_1_posrisk_4_negrisk_5 | Patients | hypothetical |
| 0.111862 | 0.352387 | 0.643274 | pred_wait_2_detect_1_form4_stage_1_cancers_5_posrisk_4_negrisk_5 | Patients | hypothetical |
| 0.115167 | 0.371075 | 0.679174 | pred_wait_2_detect_1_form4_stage_1_cancers_10_posrisk_4_negrisk_5 | Patients | hypothetical |
| 0.147273 | 0.442972 | 0.774488 | pred_wait_2_detect_1_form4_stage_1_cancers_25_posrisk_4_negrisk_5 | Patients | hypothetical |
| 0.110715 | 0.335018 | 0.60839 | pred_wait_2_detect_1_form4_stage_1_cancers_1_posrisk_4_negrisk_10 | Patients | hypothetical |
| 0.111862 | 0.347676 | 0.636821 | pred_wait_2_detect_1_form4_stage_1_cancers_5_posrisk_4_negrisk_10 | Patients | hypothetical |
| 0.115167 | 0.366279 | 0.673792 | pred_wait_2_detect_1_form4_stage_1_cancers_10_posrisk_4_negrisk_10 | Patients | hypothetical |
| 0.147273 | 0.438664 | 0.772624 | pred_wait_2_detect_1_form4_stage_1_cancers_25_posrisk_4_negrisk_10 | Patients | hypothetical |
| 0.110618 | 0.316262 | 0.555857 | pred_wait_2_detect_1_form4_stage_1_cancers_1_posrisk_4_negrisk_40 | Patients | hypothetical |
| 0.111767 | 0.327657 | 0.579561 | pred_wait_2_detect_1_form4_stage_1_cancers_5_posrisk_4_negrisk_40 | Patients | hypothetical |
| 0.115153 | 0.344615 | 0.611993 | pred_wait_2_detect_1_form4_stage_1_cancers_10_posrisk_4_negrisk_40 | Patients | hypothetical |
| 0.147273 | 0.412897 | 0.707796 | pred_wait_2_detect_1_form4_stage_1_cancers_25_posrisk_4_negrisk_40 | Patients | hypothetical |
| 0.089891 | 0.31147 | 0.620335 | pred_wait_2_detect_1_form4_stage_1_cancers_1_posrisk_6_negrisk_1 | Patients | hypothetical |
| 0.091778 | 0.322679 | 0.647194 | pred_wait_2_detect_1_form4_stage_1_cancers_5_posrisk_6_negrisk_1 | Patients | hypothetical |
| 0.095358 | 0.338942 | 0.681878 | pred_wait_2_detect_1_form4_stage_1_cancers_10_posrisk_6_negrisk_1 | Patients | hypothetical |
| 0.120116 | 0.40078 | 0.774045 | pred_wait_2_detect_1_form4_stage_1_cancers_25_posrisk_6_negrisk_1 | Patients | hypothetical |
| 0.089891 | 0.307676 | 0.615487 | pred_wait_2_detect_1_form4_stage_1_cancers_1_posrisk_6_negrisk_5 | Patients | hypothetical |
| 0.091778 | 0.318812 | 0.643274 | pred_wait_2_detect_1_form4_stage_1_cancers_5_posrisk_6_negrisk_5 | Patients | hypothetical |
| 0.095358 | 0.335069 | 0.679174 | pred_wait_2_detect_1_form4_stage_1_cancers_10_posrisk_6_negrisk_5 | Patients | hypothetical |
| 0.120116 | 0.397537 | 0.774488 | pred_wait_2_detect_1_form4_stage_1_cancers_25_posrisk_6_negrisk_5 | Patients | hypothetical |
| 0.089891 | 0.303104 | 0.60839 | pred_wait_2_detect_1_form4_stage_1_cancers_1_posrisk_6_negrisk_10 | Patients | hypothetical |
| 0.091778 | 0.314101 | 0.636821 | pred_wait_2_detect_1_form4_stage_1_cancers_5_posrisk_6_negrisk_10 | Patients | hypothetical |
| 0.095358 | 0.330273 | 0.673792 | pred_wait_2_detect_1_form4_stage_1_cancers_10_posrisk_6_negrisk_10 | Patients | hypothetical |
| 0.120116 | 0.393229 | 0.772624 | pred_wait_2_detect_1_form4_stage_1_cancers_25_posrisk_6_negrisk_10 | Patients | hypothetical |
| 0.089891 | 0.284348 | 0.555857 | pred_wait_2_detect_1_form4_stage_1_cancers_1_posrisk_6_negrisk_40 | Patients | hypothetical |
| 0.091778 | 0.294083 | 0.579561 | pred_wait_2_detect_1_form4_stage_1_cancers_5_posrisk_6_negrisk_40 | Patients | hypothetical |
| 0.095358 | 0.308609 | 0.611993 | pred_wait_2_detect_1_form4_stage_1_cancers_10_posrisk_6_negrisk_40 | Patients | hypothetical |
| 0.120116 | 0.367462 | 0.707796 | pred_wait_2_detect_1_form4_stage_1_cancers_25_posrisk_6_negrisk_40 | Patients | hypothetical |
| 0.089238 | 0.297922 | 0.620335 | pred_wait_2_detect_1_form4_stage_1_cancers_1_posrisk_8_negrisk_1 | Patients | hypothetical |
| 0.091829 | 0.308532 | 0.647194 | pred_wait_2_detect_1_form4_stage_1_cancers_5_posrisk_8_negrisk_1 | Patients | hypothetical |
| 0.095875 | 0.323731 | 0.681878 | pred_wait_2_detect_1_form4_stage_1_cancers_10_posrisk_8_negrisk_1 | Patients | hypothetical |
| 0.117168 | 0.379898 | 0.774045 | pred_wait_2_detect_1_form4_stage_1_cancers_25_posrisk_8_negrisk_1 | Patients | hypothetical |
| 0.089238 | 0.294128 | 0.615487 | pred_wait_2_detect_1_form4_stage_1_cancers_1_posrisk_8_negrisk_5 | Patients | hypothetical |
| 0.091829 | 0.304665 | 0.643274 | pred_wait_2_detect_1_form4_stage_1_cancers_5_posrisk_8_negrisk_5 | Patients | hypothetical |
| 0.095875 | 0.319858 | 0.679174 | pred_wait_2_detect_1_form4_stage_1_cancers_10_posrisk_8_negrisk_5 | Patients | hypothetical |
| 0.117168 | 0.376655 | 0.774488 | pred_wait_2_detect_1_form4_stage_1_cancers_25_posrisk_8_negrisk_5 | Patients | hypothetical |
| 0.089238 | 0.289556 | 0.60839 | pred_wait_2_detect_1_form4_stage_1_cancers_1_posrisk_8_negrisk_10 | Patients | hypothetical |
| 0.091829 | 0.299954 | 0.636821 | pred_wait_2_detect_1_form4_stage_1_cancers_5_posrisk_8_negrisk_10 | Patients | hypothetical |
| 0.095875 | 0.315062 | 0.673792 | pred_wait_2_detect_1_form4_stage_1_cancers_10_posrisk_8_negrisk_10 | Patients | hypothetical |
| 0.117168 | 0.372347 | 0.772624 | pred_wait_2_detect_1_form4_stage_1_cancers_25_posrisk_8_negrisk_10 | Patients | hypothetical |
| 0.089238 | 0.2708 | 0.555857 | pred_wait_2_detect_1_form4_stage_1_cancers_1_posrisk_8_negrisk_40 | Patients | hypothetical |
| 0.091829 | 0.279935 | 0.579561 | pred_wait_2_detect_1_form4_stage_1_cancers_5_posrisk_8_negrisk_40 | Patients | hypothetical |
| 0.095875 | 0.293398 | 0.611993 | pred_wait_2_detect_1_form4_stage_1_cancers_10_posrisk_8_negrisk_40 | Patients | hypothetical |
| 0.117168 | 0.34658 | 0.707796 | pred_wait_2_detect_1_form4_stage_1_cancers_25_posrisk_8_negrisk_40 | Patients | hypothetical |
| 0.177685 | 0.402604 | 0.630238 | pred_wait_2_detect_1_form4_stage_2_cancers_1_posrisk_2_negrisk_1 | Patients | hypothetical |
| 0.182846 | 0.418716 | 0.657271 | pred_wait_2_detect_1_form4_stage_2_cancers_5_posrisk_2_negrisk_1 | Patients | hypothetical |
| 0.191994 | 0.441408 | 0.692158 | pred_wait_2_detect_1_form4_stage_2_cancers_10_posrisk_2_negrisk_1 | Patients | hypothetical |
| 0.246904 | 0.52136 | 0.783577 | pred_wait_2_detect_1_form4_stage_2_cancers_25_posrisk_2_negrisk_1 | Patients | hypothetical |
| 0.172999 | 0.39881 | 0.628372 | pred_wait_2_detect_1_form4_stage_2_cancers_1_posrisk_2_negrisk_5 | Patients | hypothetical |
| 0.177665 | 0.414849 | 0.655619 | pred_wait_2_detect_1_form4_stage_2_cancers_5_posrisk_2_negrisk_5 | Patients | hypothetical |
| 0.186554 | 0.437535 | 0.690041 | pred_wait_2_detect_1_form4_stage_2_cancers_10_posrisk_2_negrisk_5 | Patients | hypothetical |
| 0.240509 | 0.518117 | 0.783733 | pred_wait_2_detect_1_form4_stage_2_cancers_25_posrisk_2_negrisk_5 | Patients | hypothetical |
| 0.166163 | 0.394238 | 0.625924 | pred_wait_2_detect_1_form4_stage_2_cancers_1_posrisk_2_negrisk_10 | Patients | hypothetical |
| 0.169755 | 0.410138 | 0.652845 | pred_wait_2_detect_1_form4_stage_2_cancers_5_posrisk_2_negrisk_10 | Patients | hypothetical |
| 0.177252 | 0.432739 | 0.688226 | pred_wait_2_detect_1_form4_stage_2_cancers_10_posrisk_2_negrisk_10 | Patients | hypothetical |
| 0.228034 | 0.513809 | 0.782569 | pred_wait_2_detect_1_form4_stage_2_cancers_25_posrisk_2_negrisk_10 | Patients | hypothetical |
| 0.147697 | 0.375481 | 0.619559 | pred_wait_2_detect_1_form4_stage_2_cancers_1_posrisk_2_negrisk_40 | Patients | hypothetical |
| 0.150532 | 0.39012 | 0.646715 | pred_wait_2_detect_1_form4_stage_2_cancers_5_posrisk_2_negrisk_40 | Patients | hypothetical |
| 0.155941 | 0.411075 | 0.681777 | pred_wait_2_detect_1_form4_stage_2_cancers_10_posrisk_2_negrisk_40 | Patients | hypothetical |
| 0.198626 | 0.488042 | 0.774579 | pred_wait_2_detect_1_form4_stage_2_cancers_25_posrisk_2_negrisk_40 | Patients | hypothetical |
| 0.110715 | 0.343384 | 0.620335 | pred_wait_2_detect_1_form4_stage_2_cancers_1_posrisk_4_negrisk_1 | Patients | hypothetical |
| 0.111862 | 0.356254 | 0.647194 | pred_wait_2_detect_1_form4_stage_2_cancers_5_posrisk_4_negrisk_1 | Patients | hypothetical |
| 0.115167 | 0.374947 | 0.681878 | pred_wait_2_detect_1_form4_stage_2_cancers_10_posrisk_4_negrisk_1 | Patients | hypothetical |
| 0.147273 | 0.446215 | 0.774045 | pred_wait_2_detect_1_form4_stage_2_cancers_25_posrisk_4_negrisk_1 | Patients | hypothetical |
| 0.110715 | 0.33959 | 0.615487 | pred_wait_2_detect_1_form4_stage_2_cancers_1_posrisk_4_negrisk_5 | Patients | hypothetical |
| 0.111862 | 0.352387 | 0.643274 | pred_wait_2_detect_1_form4_stage_2_cancers_5_posrisk_4_negrisk_5 | Patients | hypothetical |
| 0.115167 | 0.371075 | 0.679174 | pred_wait_2_detect_1_form4_stage_2_cancers_10_posrisk_4_negrisk_5 | Patients | hypothetical |
| 0.147273 | 0.442972 | 0.774488 | pred_wait_2_detect_1_form4_stage_2_cancers_25_posrisk_4_negrisk_5 | Patients | hypothetical |
| 0.110715 | 0.335018 | 0.60839 | pred_wait_2_detect_1_form4_stage_2_cancers_1_posrisk_4_negrisk_10 | Patients | hypothetical |
| 0.111862 | 0.347676 | 0.636821 | pred_wait_2_detect_1_form4_stage_2_cancers_5_posrisk_4_negrisk_10 | Patients | hypothetical |
| 0.115167 | 0.366279 | 0.673792 | pred_wait_2_detect_1_form4_stage_2_cancers_10_posrisk_4_negrisk_10 | Patients | hypothetical |
| 0.147273 | 0.438664 | 0.772624 | pred_wait_2_detect_1_form4_stage_2_cancers_25_posrisk_4_negrisk_10 | Patients | hypothetical |
| 0.110618 | 0.316262 | 0.555857 | pred_wait_2_detect_1_form4_stage_2_cancers_1_posrisk_4_negrisk_40 | Patients | hypothetical |
| 0.111767 | 0.327657 | 0.579561 | pred_wait_2_detect_1_form4_stage_2_cancers_5_posrisk_4_negrisk_40 | Patients | hypothetical |
| 0.115153 | 0.344615 | 0.611993 | pred_wait_2_detect_1_form4_stage_2_cancers_10_posrisk_4_negrisk_40 | Patients | hypothetical |
| 0.147273 | 0.412897 | 0.707796 | pred_wait_2_detect_1_form4_stage_2_cancers_25_posrisk_4_negrisk_40 | Patients | hypothetical |
| 0.089891 | 0.31147 | 0.620335 | pred_wait_2_detect_1_form4_stage_2_cancers_1_posrisk_6_negrisk_1 | Patients | hypothetical |
| 0.091778 | 0.322679 | 0.647194 | pred_wait_2_detect_1_form4_stage_2_cancers_5_posrisk_6_negrisk_1 | Patients | hypothetical |
| 0.095358 | 0.338942 | 0.681878 | pred_wait_2_detect_1_form4_stage_2_cancers_10_posrisk_6_negrisk_1 | Patients | hypothetical |
| 0.120116 | 0.40078 | 0.774045 | pred_wait_2_detect_1_form4_stage_2_cancers_25_posrisk_6_negrisk_1 | Patients | hypothetical |
| 0.089891 | 0.307676 | 0.615487 | pred_wait_2_detect_1_form4_stage_2_cancers_1_posrisk_6_negrisk_5 | Patients | hypothetical |
| 0.091778 | 0.318812 | 0.643274 | pred_wait_2_detect_1_form4_stage_2_cancers_5_posrisk_6_negrisk_5 | Patients | hypothetical |
| 0.095358 | 0.335069 | 0.679174 | pred_wait_2_detect_1_form4_stage_2_cancers_10_posrisk_6_negrisk_5 | Patients | hypothetical |
| 0.120116 | 0.397537 | 0.774488 | pred_wait_2_detect_1_form4_stage_2_cancers_25_posrisk_6_negrisk_5 | Patients | hypothetical |
| 0.089891 | 0.303104 | 0.60839 | pred_wait_2_detect_1_form4_stage_2_cancers_1_posrisk_6_negrisk_10 | Patients | hypothetical |
| 0.091778 | 0.314101 | 0.636821 | pred_wait_2_detect_1_form4_stage_2_cancers_5_posrisk_6_negrisk_10 | Patients | hypothetical |
| 0.095358 | 0.330273 | 0.673792 | pred_wait_2_detect_1_form4_stage_2_cancers_10_posrisk_6_negrisk_10 | Patients | hypothetical |
| 0.120116 | 0.393229 | 0.772624 | pred_wait_2_detect_1_form4_stage_2_cancers_25_posrisk_6_negrisk_10 | Patients | hypothetical |
| 0.089891 | 0.284348 | 0.555857 | pred_wait_2_detect_1_form4_stage_2_cancers_1_posrisk_6_negrisk_40 | Patients | hypothetical |
| 0.091778 | 0.294083 | 0.579561 | pred_wait_2_detect_1_form4_stage_2_cancers_5_posrisk_6_negrisk_40 | Patients | hypothetical |
| 0.095358 | 0.308609 | 0.611993 | pred_wait_2_detect_1_form4_stage_2_cancers_10_posrisk_6_negrisk_40 | Patients | hypothetical |
| 0.120116 | 0.367462 | 0.707796 | pred_wait_2_detect_1_form4_stage_2_cancers_25_posrisk_6_negrisk_40 | Patients | hypothetical |
| 0.089238 | 0.297922 | 0.620335 | pred_wait_2_detect_1_form4_stage_2_cancers_1_posrisk_8_negrisk_1 | Patients | hypothetical |
| 0.091829 | 0.308532 | 0.647194 | pred_wait_2_detect_1_form4_stage_2_cancers_5_posrisk_8_negrisk_1 | Patients | hypothetical |
| 0.095875 | 0.323731 | 0.681878 | pred_wait_2_detect_1_form4_stage_2_cancers_10_posrisk_8_negrisk_1 | Patients | hypothetical |
| 0.117168 | 0.379898 | 0.774045 | pred_wait_2_detect_1_form4_stage_2_cancers_25_posrisk_8_negrisk_1 | Patients | hypothetical |
| 0.089238 | 0.294128 | 0.615487 | pred_wait_2_detect_1_form4_stage_2_cancers_1_posrisk_8_negrisk_5 | Patients | hypothetical |
| 0.091829 | 0.304665 | 0.643274 | pred_wait_2_detect_1_form4_stage_2_cancers_5_posrisk_8_negrisk_5 | Patients | hypothetical |
| 0.095875 | 0.319858 | 0.679174 | pred_wait_2_detect_1_form4_stage_2_cancers_10_posrisk_8_negrisk_5 | Patients | hypothetical |
| 0.117168 | 0.376655 | 0.774488 | pred_wait_2_detect_1_form4_stage_2_cancers_25_posrisk_8_negrisk_5 | Patients | hypothetical |
| 0.089238 | 0.289556 | 0.60839 | pred_wait_2_detect_1_form4_stage_2_cancers_1_posrisk_8_negrisk_10 | Patients | hypothetical |
| 0.091829 | 0.299954 | 0.636821 | pred_wait_2_detect_1_form4_stage_2_cancers_5_posrisk_8_negrisk_10 | Patients | hypothetical |
| 0.095875 | 0.315062 | 0.673792 | pred_wait_2_detect_1_form4_stage_2_cancers_10_posrisk_8_negrisk_10 | Patients | hypothetical |
| 0.117168 | 0.372347 | 0.772624 | pred_wait_2_detect_1_form4_stage_2_cancers_25_posrisk_8_negrisk_10 | Patients | hypothetical |
| 0.089238 | 0.2708 | 0.555857 | pred_wait_2_detect_1_form4_stage_2_cancers_1_posrisk_8_negrisk_40 | Patients | hypothetical |
| 0.091829 | 0.279935 | 0.579561 | pred_wait_2_detect_1_form4_stage_2_cancers_5_posrisk_8_negrisk_40 | Patients | hypothetical |
| 0.095875 | 0.293398 | 0.611993 | pred_wait_2_detect_1_form4_stage_2_cancers_10_posrisk_8_negrisk_40 | Patients | hypothetical |
| 0.117168 | 0.34658 | 0.707796 | pred_wait_2_detect_1_form4_stage_2_cancers_25_posrisk_8_negrisk_40 | Patients | hypothetical |
| 0.177685 | 0.402604 | 0.630238 | pred_wait_2_detect_2_form1_stage_1_cancers_1_posrisk_2_negrisk_1 | Patients | hypothetical |
| 0.182846 | 0.418716 | 0.657271 | pred_wait_2_detect_2_form1_stage_1_cancers_5_posrisk_2_negrisk_1 | Patients | hypothetical |
| 0.191994 | 0.441408 | 0.692158 | pred_wait_2_detect_2_form1_stage_1_cancers_10_posrisk_2_negrisk_1 | Patients | hypothetical |
| 0.246904 | 0.52136 | 0.783577 | pred_wait_2_detect_2_form1_stage_1_cancers_25_posrisk_2_negrisk_1 | Patients | hypothetical |
| 0.172999 | 0.39881 | 0.628372 | pred_wait_2_detect_2_form1_stage_1_cancers_1_posrisk_2_negrisk_5 | Patients | hypothetical |
| 0.177665 | 0.414849 | 0.655619 | pred_wait_2_detect_2_form1_stage_1_cancers_5_posrisk_2_negrisk_5 | Patients | hypothetical |
| 0.186554 | 0.437535 | 0.690041 | pred_wait_2_detect_2_form1_stage_1_cancers_10_posrisk_2_negrisk_5 | Patients | hypothetical |
| 0.240509 | 0.518117 | 0.783733 | pred_wait_2_detect_2_form1_stage_1_cancers_25_posrisk_2_negrisk_5 | Patients | hypothetical |
| 0.166163 | 0.394238 | 0.625924 | pred_wait_2_detect_2_form1_stage_1_cancers_1_posrisk_2_negrisk_10 | Patients | hypothetical |
| 0.169755 | 0.410138 | 0.652845 | pred_wait_2_detect_2_form1_stage_1_cancers_5_posrisk_2_negrisk_10 | Patients | hypothetical |
| 0.177252 | 0.432739 | 0.688226 | pred_wait_2_detect_2_form1_stage_1_cancers_10_posrisk_2_negrisk_10 | Patients | hypothetical |
| 0.228034 | 0.513809 | 0.782569 | pred_wait_2_detect_2_form1_stage_1_cancers_25_posrisk_2_negrisk_10 | Patients | hypothetical |
| 0.147697 | 0.375481 | 0.619559 | pred_wait_2_detect_2_form1_stage_1_cancers_1_posrisk_2_negrisk_40 | Patients | hypothetical |
| 0.150532 | 0.39012 | 0.646715 | pred_wait_2_detect_2_form1_stage_1_cancers_5_posrisk_2_negrisk_40 | Patients | hypothetical |
| 0.155941 | 0.411075 | 0.681777 | pred_wait_2_detect_2_form1_stage_1_cancers_10_posrisk_2_negrisk_40 | Patients | hypothetical |
| 0.198626 | 0.488042 | 0.774579 | pred_wait_2_detect_2_form1_stage_1_cancers_25_posrisk_2_negrisk_40 | Patients | hypothetical |
| 0.110715 | 0.343384 | 0.620335 | pred_wait_2_detect_2_form1_stage_1_cancers_1_posrisk_4_negrisk_1 | Patients | hypothetical |
| 0.111862 | 0.356254 | 0.647194 | pred_wait_2_detect_2_form1_stage_1_cancers_5_posrisk_4_negrisk_1 | Patients | hypothetical |
| 0.115167 | 0.374947 | 0.681878 | pred_wait_2_detect_2_form1_stage_1_cancers_10_posrisk_4_negrisk_1 | Patients | hypothetical |
| 0.147273 | 0.446215 | 0.774045 | pred_wait_2_detect_2_form1_stage_1_cancers_25_posrisk_4_negrisk_1 | Patients | hypothetical |
| 0.110715 | 0.33959 | 0.615487 | pred_wait_2_detect_2_form1_stage_1_cancers_1_posrisk_4_negrisk_5 | Patients | hypothetical |
| 0.111862 | 0.352387 | 0.643274 | pred_wait_2_detect_2_form1_stage_1_cancers_5_posrisk_4_negrisk_5 | Patients | hypothetical |
| 0.115167 | 0.371075 | 0.679174 | pred_wait_2_detect_2_form1_stage_1_cancers_10_posrisk_4_negrisk_5 | Patients | hypothetical |
| 0.147273 | 0.442972 | 0.774488 | pred_wait_2_detect_2_form1_stage_1_cancers_25_posrisk_4_negrisk_5 | Patients | hypothetical |
| 0.110715 | 0.335018 | 0.60839 | pred_wait_2_detect_2_form1_stage_1_cancers_1_posrisk_4_negrisk_10 | Patients | hypothetical |
| 0.111862 | 0.347676 | 0.636821 | pred_wait_2_detect_2_form1_stage_1_cancers_5_posrisk_4_negrisk_10 | Patients | hypothetical |
| 0.115167 | 0.366279 | 0.673792 | pred_wait_2_detect_2_form1_stage_1_cancers_10_posrisk_4_negrisk_10 | Patients | hypothetical |
| 0.147273 | 0.438664 | 0.772624 | pred_wait_2_detect_2_form1_stage_1_cancers_25_posrisk_4_negrisk_10 | Patients | hypothetical |
| 0.110618 | 0.316262 | 0.555857 | pred_wait_2_detect_2_form1_stage_1_cancers_1_posrisk_4_negrisk_40 | Patients | hypothetical |
| 0.111767 | 0.327657 | 0.579561 | pred_wait_2_detect_2_form1_stage_1_cancers_5_posrisk_4_negrisk_40 | Patients | hypothetical |
| 0.115153 | 0.344615 | 0.611993 | pred_wait_2_detect_2_form1_stage_1_cancers_10_posrisk_4_negrisk_40 | Patients | hypothetical |
| 0.147273 | 0.412897 | 0.707796 | pred_wait_2_detect_2_form1_stage_1_cancers_25_posrisk_4_negrisk_40 | Patients | hypothetical |
| 0.089891 | 0.31147 | 0.620335 | pred_wait_2_detect_2_form1_stage_1_cancers_1_posrisk_6_negrisk_1 | Patients | hypothetical |
| 0.091778 | 0.322679 | 0.647194 | pred_wait_2_detect_2_form1_stage_1_cancers_5_posrisk_6_negrisk_1 | Patients | hypothetical |
| 0.095358 | 0.338942 | 0.681878 | pred_wait_2_detect_2_form1_stage_1_cancers_10_posrisk_6_negrisk_1 | Patients | hypothetical |
| 0.120116 | 0.40078 | 0.774045 | pred_wait_2_detect_2_form1_stage_1_cancers_25_posrisk_6_negrisk_1 | Patients | hypothetical |
| 0.089891 | 0.307676 | 0.615487 | pred_wait_2_detect_2_form1_stage_1_cancers_1_posrisk_6_negrisk_5 | Patients | hypothetical |
| 0.091778 | 0.318812 | 0.643274 | pred_wait_2_detect_2_form1_stage_1_cancers_5_posrisk_6_negrisk_5 | Patients | hypothetical |
| 0.095358 | 0.335069 | 0.679174 | pred_wait_2_detect_2_form1_stage_1_cancers_10_posrisk_6_negrisk_5 | Patients | hypothetical |
| 0.120116 | 0.397537 | 0.774488 | pred_wait_2_detect_2_form1_stage_1_cancers_25_posrisk_6_negrisk_5 | Patients | hypothetical |
| 0.089891 | 0.303104 | 0.60839 | pred_wait_2_detect_2_form1_stage_1_cancers_1_posrisk_6_negrisk_10 | Patients | hypothetical |
| 0.091778 | 0.314101 | 0.636821 | pred_wait_2_detect_2_form1_stage_1_cancers_5_posrisk_6_negrisk_10 | Patients | hypothetical |
| 0.095358 | 0.330273 | 0.673792 | pred_wait_2_detect_2_form1_stage_1_cancers_10_posrisk_6_negrisk_10 | Patients | hypothetical |
| 0.120116 | 0.393229 | 0.772624 | pred_wait_2_detect_2_form1_stage_1_cancers_25_posrisk_6_negrisk_10 | Patients | hypothetical |
| 0.089891 | 0.284348 | 0.555857 | pred_wait_2_detect_2_form1_stage_1_cancers_1_posrisk_6_negrisk_40 | Patients | hypothetical |
| 0.091778 | 0.294083 | 0.579561 | pred_wait_2_detect_2_form1_stage_1_cancers_5_posrisk_6_negrisk_40 | Patients | hypothetical |
| 0.095358 | 0.308609 | 0.611993 | pred_wait_2_detect_2_form1_stage_1_cancers_10_posrisk_6_negrisk_40 | Patients | hypothetical |
| 0.120116 | 0.367462 | 0.707796 | pred_wait_2_detect_2_form1_stage_1_cancers_25_posrisk_6_negrisk_40 | Patients | hypothetical |
| 0.089238 | 0.297922 | 0.620335 | pred_wait_2_detect_2_form1_stage_1_cancers_1_posrisk_8_negrisk_1 | Patients | hypothetical |
| 0.091829 | 0.308532 | 0.647194 | pred_wait_2_detect_2_form1_stage_1_cancers_5_posrisk_8_negrisk_1 | Patients | hypothetical |
| 0.095875 | 0.323731 | 0.681878 | pred_wait_2_detect_2_form1_stage_1_cancers_10_posrisk_8_negrisk_1 | Patients | hypothetical |
| 0.117168 | 0.379898 | 0.774045 | pred_wait_2_detect_2_form1_stage_1_cancers_25_posrisk_8_negrisk_1 | Patients | hypothetical |
| 0.089238 | 0.294128 | 0.615487 | pred_wait_2_detect_2_form1_stage_1_cancers_1_posrisk_8_negrisk_5 | Patients | hypothetical |
| 0.091829 | 0.304665 | 0.643274 | pred_wait_2_detect_2_form1_stage_1_cancers_5_posrisk_8_negrisk_5 | Patients | hypothetical |
| 0.095875 | 0.319858 | 0.679174 | pred_wait_2_detect_2_form1_stage_1_cancers_10_posrisk_8_negrisk_5 | Patients | hypothetical |
| 0.117168 | 0.376655 | 0.774488 | pred_wait_2_detect_2_form1_stage_1_cancers_25_posrisk_8_negrisk_5 | Patients | hypothetical |
| 0.089238 | 0.289556 | 0.60839 | pred_wait_2_detect_2_form1_stage_1_cancers_1_posrisk_8_negrisk_10 | Patients | hypothetical |
| 0.091829 | 0.299954 | 0.636821 | pred_wait_2_detect_2_form1_stage_1_cancers_5_posrisk_8_negrisk_10 | Patients | hypothetical |
| 0.095875 | 0.315062 | 0.673792 | pred_wait_2_detect_2_form1_stage_1_cancers_10_posrisk_8_negrisk_10 | Patients | hypothetical |
| 0.117168 | 0.372347 | 0.772624 | pred_wait_2_detect_2_form1_stage_1_cancers_25_posrisk_8_negrisk_10 | Patients | hypothetical |
| 0.089238 | 0.2708 | 0.555857 | pred_wait_2_detect_2_form1_stage_1_cancers_1_posrisk_8_negrisk_40 | Patients | hypothetical |
| 0.091829 | 0.279935 | 0.579561 | pred_wait_2_detect_2_form1_stage_1_cancers_5_posrisk_8_negrisk_40 | Patients | hypothetical |
| 0.095875 | 0.293398 | 0.611993 | pred_wait_2_detect_2_form1_stage_1_cancers_10_posrisk_8_negrisk_40 | Patients | hypothetical |
| 0.117168 | 0.34658 | 0.707796 | pred_wait_2_detect_2_form1_stage_1_cancers_25_posrisk_8_negrisk_40 | Patients | hypothetical |
| 0.177685 | 0.402604 | 0.630238 | pred_wait_2_detect_2_form1_stage_2_cancers_1_posrisk_2_negrisk_1 | Patients | hypothetical |
| 0.182846 | 0.418716 | 0.657271 | pred_wait_2_detect_2_form1_stage_2_cancers_5_posrisk_2_negrisk_1 | Patients | hypothetical |
| 0.191994 | 0.441408 | 0.692158 | pred_wait_2_detect_2_form1_stage_2_cancers_10_posrisk_2_negrisk_1 | Patients | hypothetical |
| 0.246904 | 0.52136 | 0.783577 | pred_wait_2_detect_2_form1_stage_2_cancers_25_posrisk_2_negrisk_1 | Patients | hypothetical |
| 0.172999 | 0.39881 | 0.628372 | pred_wait_2_detect_2_form1_stage_2_cancers_1_posrisk_2_negrisk_5 | Patients | hypothetical |
| 0.177665 | 0.414849 | 0.655619 | pred_wait_2_detect_2_form1_stage_2_cancers_5_posrisk_2_negrisk_5 | Patients | hypothetical |
| 0.186554 | 0.437535 | 0.690041 | pred_wait_2_detect_2_form1_stage_2_cancers_10_posrisk_2_negrisk_5 | Patients | hypothetical |
| 0.240509 | 0.518117 | 0.783733 | pred_wait_2_detect_2_form1_stage_2_cancers_25_posrisk_2_negrisk_5 | Patients | hypothetical |
| 0.166163 | 0.394238 | 0.625924 | pred_wait_2_detect_2_form1_stage_2_cancers_1_posrisk_2_negrisk_10 | Patients | hypothetical |
| 0.169755 | 0.410138 | 0.652845 | pred_wait_2_detect_2_form1_stage_2_cancers_5_posrisk_2_negrisk_10 | Patients | hypothetical |
| 0.177252 | 0.432739 | 0.688226 | pred_wait_2_detect_2_form1_stage_2_cancers_10_posrisk_2_negrisk_10 | Patients | hypothetical |
| 0.228034 | 0.513809 | 0.782569 | pred_wait_2_detect_2_form1_stage_2_cancers_25_posrisk_2_negrisk_10 | Patients | hypothetical |
| 0.147697 | 0.375481 | 0.619559 | pred_wait_2_detect_2_form1_stage_2_cancers_1_posrisk_2_negrisk_40 | Patients | hypothetical |
| 0.150532 | 0.39012 | 0.646715 | pred_wait_2_detect_2_form1_stage_2_cancers_5_posrisk_2_negrisk_40 | Patients | hypothetical |
| 0.155941 | 0.411075 | 0.681777 | pred_wait_2_detect_2_form1_stage_2_cancers_10_posrisk_2_negrisk_40 | Patients | hypothetical |
| 0.198626 | 0.488042 | 0.774579 | pred_wait_2_detect_2_form1_stage_2_cancers_25_posrisk_2_negrisk_40 | Patients | hypothetical |
| 0.110715 | 0.343384 | 0.620335 | pred_wait_2_detect_2_form1_stage_2_cancers_1_posrisk_4_negrisk_1 | Patients | hypothetical |
| 0.111862 | 0.356254 | 0.647194 | pred_wait_2_detect_2_form1_stage_2_cancers_5_posrisk_4_negrisk_1 | Patients | hypothetical |
| 0.115167 | 0.374947 | 0.681878 | pred_wait_2_detect_2_form1_stage_2_cancers_10_posrisk_4_negrisk_1 | Patients | hypothetical |
| 0.147273 | 0.446215 | 0.774045 | pred_wait_2_detect_2_form1_stage_2_cancers_25_posrisk_4_negrisk_1 | Patients | hypothetical |
| 0.110715 | 0.33959 | 0.615487 | pred_wait_2_detect_2_form1_stage_2_cancers_1_posrisk_4_negrisk_5 | Patients | hypothetical |
| 0.111862 | 0.352387 | 0.643274 | pred_wait_2_detect_2_form1_stage_2_cancers_5_posrisk_4_negrisk_5 | Patients | hypothetical |
| 0.115167 | 0.371075 | 0.679174 | pred_wait_2_detect_2_form1_stage_2_cancers_10_posrisk_4_negrisk_5 | Patients | hypothetical |
| 0.147273 | 0.442972 | 0.774488 | pred_wait_2_detect_2_form1_stage_2_cancers_25_posrisk_4_negrisk_5 | Patients | hypothetical |
| 0.110715 | 0.335018 | 0.60839 | pred_wait_2_detect_2_form1_stage_2_cancers_1_posrisk_4_negrisk_10 | Patients | hypothetical |
| 0.111862 | 0.347676 | 0.636821 | pred_wait_2_detect_2_form1_stage_2_cancers_5_posrisk_4_negrisk_10 | Patients | hypothetical |
| 0.115167 | 0.366279 | 0.673792 | pred_wait_2_detect_2_form1_stage_2_cancers_10_posrisk_4_negrisk_10 | Patients | hypothetical |
| 0.147273 | 0.438664 | 0.772624 | pred_wait_2_detect_2_form1_stage_2_cancers_25_posrisk_4_negrisk_10 | Patients | hypothetical |
| 0.110618 | 0.316262 | 0.555857 | pred_wait_2_detect_2_form1_stage_2_cancers_1_posrisk_4_negrisk_40 | Patients | hypothetical |
| 0.111767 | 0.327657 | 0.579561 | pred_wait_2_detect_2_form1_stage_2_cancers_5_posrisk_4_negrisk_40 | Patients | hypothetical |
| 0.115153 | 0.344615 | 0.611993 | pred_wait_2_detect_2_form1_stage_2_cancers_10_posrisk_4_negrisk_40 | Patients | hypothetical |
| 0.147273 | 0.412897 | 0.707796 | pred_wait_2_detect_2_form1_stage_2_cancers_25_posrisk_4_negrisk_40 | Patients | hypothetical |
| 0.089891 | 0.31147 | 0.620335 | pred_wait_2_detect_2_form1_stage_2_cancers_1_posrisk_6_negrisk_1 | Patients | hypothetical |
| 0.091778 | 0.322679 | 0.647194 | pred_wait_2_detect_2_form1_stage_2_cancers_5_posrisk_6_negrisk_1 | Patients | hypothetical |
| 0.095358 | 0.338942 | 0.681878 | pred_wait_2_detect_2_form1_stage_2_cancers_10_posrisk_6_negrisk_1 | Patients | hypothetical |
| 0.120116 | 0.40078 | 0.774045 | pred_wait_2_detect_2_form1_stage_2_cancers_25_posrisk_6_negrisk_1 | Patients | hypothetical |
| 0.089891 | 0.307676 | 0.615487 | pred_wait_2_detect_2_form1_stage_2_cancers_1_posrisk_6_negrisk_5 | Patients | hypothetical |
| 0.091778 | 0.318812 | 0.643274 | pred_wait_2_detect_2_form1_stage_2_cancers_5_posrisk_6_negrisk_5 | Patients | hypothetical |
| 0.095358 | 0.335069 | 0.679174 | pred_wait_2_detect_2_form1_stage_2_cancers_10_posrisk_6_negrisk_5 | Patients | hypothetical |
| 0.120116 | 0.397537 | 0.774488 | pred_wait_2_detect_2_form1_stage_2_cancers_25_posrisk_6_negrisk_5 | Patients | hypothetical |
| 0.089891 | 0.303104 | 0.60839 | pred_wait_2_detect_2_form1_stage_2_cancers_1_posrisk_6_negrisk_10 | Patients | hypothetical |
| 0.091778 | 0.314101 | 0.636821 | pred_wait_2_detect_2_form1_stage_2_cancers_5_posrisk_6_negrisk_10 | Patients | hypothetical |
| 0.095358 | 0.330273 | 0.673792 | pred_wait_2_detect_2_form1_stage_2_cancers_10_posrisk_6_negrisk_10 | Patients | hypothetical |
| 0.120116 | 0.393229 | 0.772624 | pred_wait_2_detect_2_form1_stage_2_cancers_25_posrisk_6_negrisk_10 | Patients | hypothetical |
| 0.089891 | 0.284348 | 0.555857 | pred_wait_2_detect_2_form1_stage_2_cancers_1_posrisk_6_negrisk_40 | Patients | hypothetical |
| 0.091778 | 0.294083 | 0.579561 | pred_wait_2_detect_2_form1_stage_2_cancers_5_posrisk_6_negrisk_40 | Patients | hypothetical |
| 0.095358 | 0.308609 | 0.611993 | pred_wait_2_detect_2_form1_stage_2_cancers_10_posrisk_6_negrisk_40 | Patients | hypothetical |
| 0.120116 | 0.367462 | 0.707796 | pred_wait_2_detect_2_form1_stage_2_cancers_25_posrisk_6_negrisk_40 | Patients | hypothetical |
| 0.089238 | 0.297922 | 0.620335 | pred_wait_2_detect_2_form1_stage_2_cancers_1_posrisk_8_negrisk_1 | Patients | hypothetical |
| 0.091829 | 0.308532 | 0.647194 | pred_wait_2_detect_2_form1_stage_2_cancers_5_posrisk_8_negrisk_1 | Patients | hypothetical |
| 0.095875 | 0.323731 | 0.681878 | pred_wait_2_detect_2_form1_stage_2_cancers_10_posrisk_8_negrisk_1 | Patients | hypothetical |
| 0.117168 | 0.379898 | 0.774045 | pred_wait_2_detect_2_form1_stage_2_cancers_25_posrisk_8_negrisk_1 | Patients | hypothetical |
| 0.089238 | 0.294128 | 0.615487 | pred_wait_2_detect_2_form1_stage_2_cancers_1_posrisk_8_negrisk_5 | Patients | hypothetical |
| 0.091829 | 0.304665 | 0.643274 | pred_wait_2_detect_2_form1_stage_2_cancers_5_posrisk_8_negrisk_5 | Patients | hypothetical |
| 0.095875 | 0.319858 | 0.679174 | pred_wait_2_detect_2_form1_stage_2_cancers_10_posrisk_8_negrisk_5 | Patients | hypothetical |
| 0.117168 | 0.376655 | 0.774488 | pred_wait_2_detect_2_form1_stage_2_cancers_25_posrisk_8_negrisk_5 | Patients | hypothetical |
| 0.089238 | 0.289556 | 0.60839 | pred_wait_2_detect_2_form1_stage_2_cancers_1_posrisk_8_negrisk_10 | Patients | hypothetical |
| 0.091829 | 0.299954 | 0.636821 | pred_wait_2_detect_2_form1_stage_2_cancers_5_posrisk_8_negrisk_10 | Patients | hypothetical |
| 0.095875 | 0.315062 | 0.673792 | pred_wait_2_detect_2_form1_stage_2_cancers_10_posrisk_8_negrisk_10 | Patients | hypothetical |
| 0.117168 | 0.372347 | 0.772624 | pred_wait_2_detect_2_form1_stage_2_cancers_25_posrisk_8_negrisk_10 | Patients | hypothetical |
| 0.089238 | 0.2708 | 0.555857 | pred_wait_2_detect_2_form1_stage_2_cancers_1_posrisk_8_negrisk_40 | Patients | hypothetical |
| 0.091829 | 0.279935 | 0.579561 | pred_wait_2_detect_2_form1_stage_2_cancers_5_posrisk_8_negrisk_40 | Patients | hypothetical |
| 0.095875 | 0.293398 | 0.611993 | pred_wait_2_detect_2_form1_stage_2_cancers_10_posrisk_8_negrisk_40 | Patients | hypothetical |
| 0.117168 | 0.34658 | 0.707796 | pred_wait_2_detect_2_form1_stage_2_cancers_25_posrisk_8_negrisk_40 | Patients | hypothetical |
| 0.177685 | 0.402604 | 0.630238 | pred_wait_2_detect_2_form2_stage_1_cancers_1_posrisk_2_negrisk_1 | Patients | hypothetical |
| 0.182846 | 0.418716 | 0.657271 | pred_wait_2_detect_2_form2_stage_1_cancers_5_posrisk_2_negrisk_1 | Patients | hypothetical |
| 0.191994 | 0.441408 | 0.692158 | pred_wait_2_detect_2_form2_stage_1_cancers_10_posrisk_2_negrisk_1 | Patients | hypothetical |
| 0.246904 | 0.52136 | 0.783577 | pred_wait_2_detect_2_form2_stage_1_cancers_25_posrisk_2_negrisk_1 | Patients | hypothetical |
| 0.172999 | 0.39881 | 0.628372 | pred_wait_2_detect_2_form2_stage_1_cancers_1_posrisk_2_negrisk_5 | Patients | hypothetical |
| 0.177665 | 0.414849 | 0.655619 | pred_wait_2_detect_2_form2_stage_1_cancers_5_posrisk_2_negrisk_5 | Patients | hypothetical |
| 0.186554 | 0.437535 | 0.690041 | pred_wait_2_detect_2_form2_stage_1_cancers_10_posrisk_2_negrisk_5 | Patients | hypothetical |
| 0.240509 | 0.518117 | 0.783733 | pred_wait_2_detect_2_form2_stage_1_cancers_25_posrisk_2_negrisk_5 | Patients | hypothetical |
| 0.166163 | 0.394238 | 0.625924 | pred_wait_2_detect_2_form2_stage_1_cancers_1_posrisk_2_negrisk_10 | Patients | hypothetical |
| 0.169755 | 0.410138 | 0.652845 | pred_wait_2_detect_2_form2_stage_1_cancers_5_posrisk_2_negrisk_10 | Patients | hypothetical |
| 0.177252 | 0.432739 | 0.688226 | pred_wait_2_detect_2_form2_stage_1_cancers_10_posrisk_2_negrisk_10 | Patients | hypothetical |
| 0.228034 | 0.513809 | 0.782569 | pred_wait_2_detect_2_form2_stage_1_cancers_25_posrisk_2_negrisk_10 | Patients | hypothetical |
| 0.147697 | 0.375481 | 0.619559 | pred_wait_2_detect_2_form2_stage_1_cancers_1_posrisk_2_negrisk_40 | Patients | hypothetical |
| 0.150532 | 0.39012 | 0.646715 | pred_wait_2_detect_2_form2_stage_1_cancers_5_posrisk_2_negrisk_40 | Patients | hypothetical |
| 0.155941 | 0.411075 | 0.681777 | pred_wait_2_detect_2_form2_stage_1_cancers_10_posrisk_2_negrisk_40 | Patients | hypothetical |
| 0.198626 | 0.488042 | 0.774579 | pred_wait_2_detect_2_form2_stage_1_cancers_25_posrisk_2_negrisk_40 | Patients | hypothetical |
| 0.110715 | 0.343384 | 0.620335 | pred_wait_2_detect_2_form2_stage_1_cancers_1_posrisk_4_negrisk_1 | Patients | hypothetical |
| 0.111862 | 0.356254 | 0.647194 | pred_wait_2_detect_2_form2_stage_1_cancers_5_posrisk_4_negrisk_1 | Patients | hypothetical |
| 0.115167 | 0.374947 | 0.681878 | pred_wait_2_detect_2_form2_stage_1_cancers_10_posrisk_4_negrisk_1 | Patients | hypothetical |
| 0.147273 | 0.446215 | 0.774045 | pred_wait_2_detect_2_form2_stage_1_cancers_25_posrisk_4_negrisk_1 | Patients | hypothetical |
| 0.110715 | 0.33959 | 0.615487 | pred_wait_2_detect_2_form2_stage_1_cancers_1_posrisk_4_negrisk_5 | Patients | hypothetical |
| 0.111862 | 0.352387 | 0.643274 | pred_wait_2_detect_2_form2_stage_1_cancers_5_posrisk_4_negrisk_5 | Patients | hypothetical |
| 0.115167 | 0.371075 | 0.679174 | pred_wait_2_detect_2_form2_stage_1_cancers_10_posrisk_4_negrisk_5 | Patients | hypothetical |
| 0.147273 | 0.442972 | 0.774488 | pred_wait_2_detect_2_form2_stage_1_cancers_25_posrisk_4_negrisk_5 | Patients | hypothetical |
| 0.110715 | 0.335018 | 0.60839 | pred_wait_2_detect_2_form2_stage_1_cancers_1_posrisk_4_negrisk_10 | Patients | hypothetical |
| 0.111862 | 0.347676 | 0.636821 | pred_wait_2_detect_2_form2_stage_1_cancers_5_posrisk_4_negrisk_10 | Patients | hypothetical |
| 0.115167 | 0.366279 | 0.673792 | pred_wait_2_detect_2_form2_stage_1_cancers_10_posrisk_4_negrisk_10 | Patients | hypothetical |
| 0.147273 | 0.438664 | 0.772624 | pred_wait_2_detect_2_form2_stage_1_cancers_25_posrisk_4_negrisk_10 | Patients | hypothetical |
| 0.110618 | 0.316262 | 0.555857 | pred_wait_2_detect_2_form2_stage_1_cancers_1_posrisk_4_negrisk_40 | Patients | hypothetical |
| 0.111767 | 0.327657 | 0.579561 | pred_wait_2_detect_2_form2_stage_1_cancers_5_posrisk_4_negrisk_40 | Patients | hypothetical |
| 0.115153 | 0.344615 | 0.611993 | pred_wait_2_detect_2_form2_stage_1_cancers_10_posrisk_4_negrisk_40 | Patients | hypothetical |
| 0.147273 | 0.412897 | 0.707796 | pred_wait_2_detect_2_form2_stage_1_cancers_25_posrisk_4_negrisk_40 | Patients | hypothetical |
| 0.089891 | 0.31147 | 0.620335 | pred_wait_2_detect_2_form2_stage_1_cancers_1_posrisk_6_negrisk_1 | Patients | hypothetical |
| 0.091778 | 0.322679 | 0.647194 | pred_wait_2_detect_2_form2_stage_1_cancers_5_posrisk_6_negrisk_1 | Patients | hypothetical |
| 0.095358 | 0.338942 | 0.681878 | pred_wait_2_detect_2_form2_stage_1_cancers_10_posrisk_6_negrisk_1 | Patients | hypothetical |
| 0.120116 | 0.40078 | 0.774045 | pred_wait_2_detect_2_form2_stage_1_cancers_25_posrisk_6_negrisk_1 | Patients | hypothetical |
| 0.089891 | 0.307676 | 0.615487 | pred_wait_2_detect_2_form2_stage_1_cancers_1_posrisk_6_negrisk_5 | Patients | hypothetical |
| 0.091778 | 0.318812 | 0.643274 | pred_wait_2_detect_2_form2_stage_1_cancers_5_posrisk_6_negrisk_5 | Patients | hypothetical |
| 0.095358 | 0.335069 | 0.679174 | pred_wait_2_detect_2_form2_stage_1_cancers_10_posrisk_6_negrisk_5 | Patients | hypothetical |
| 0.120116 | 0.397537 | 0.774488 | pred_wait_2_detect_2_form2_stage_1_cancers_25_posrisk_6_negrisk_5 | Patients | hypothetical |
| 0.089891 | 0.303104 | 0.60839 | pred_wait_2_detect_2_form2_stage_1_cancers_1_posrisk_6_negrisk_10 | Patients | hypothetical |
| 0.091778 | 0.314101 | 0.636821 | pred_wait_2_detect_2_form2_stage_1_cancers_5_posrisk_6_negrisk_10 | Patients | hypothetical |
| 0.095358 | 0.330273 | 0.673792 | pred_wait_2_detect_2_form2_stage_1_cancers_10_posrisk_6_negrisk_10 | Patients | hypothetical |
| 0.120116 | 0.393229 | 0.772624 | pred_wait_2_detect_2_form2_stage_1_cancers_25_posrisk_6_negrisk_10 | Patients | hypothetical |
| 0.089891 | 0.284348 | 0.555857 | pred_wait_2_detect_2_form2_stage_1_cancers_1_posrisk_6_negrisk_40 | Patients | hypothetical |
| 0.091778 | 0.294083 | 0.579561 | pred_wait_2_detect_2_form2_stage_1_cancers_5_posrisk_6_negrisk_40 | Patients | hypothetical |
| 0.095358 | 0.308609 | 0.611993 | pred_wait_2_detect_2_form2_stage_1_cancers_10_posrisk_6_negrisk_40 | Patients | hypothetical |
| 0.120116 | 0.367462 | 0.707796 | pred_wait_2_detect_2_form2_stage_1_cancers_25_posrisk_6_negrisk_40 | Patients | hypothetical |
| 0.089238 | 0.297922 | 0.620335 | pred_wait_2_detect_2_form2_stage_1_cancers_1_posrisk_8_negrisk_1 | Patients | hypothetical |
| 0.091829 | 0.308532 | 0.647194 | pred_wait_2_detect_2_form2_stage_1_cancers_5_posrisk_8_negrisk_1 | Patients | hypothetical |
| 0.095875 | 0.323731 | 0.681878 | pred_wait_2_detect_2_form2_stage_1_cancers_10_posrisk_8_negrisk_1 | Patients | hypothetical |
| 0.117168 | 0.379898 | 0.774045 | pred_wait_2_detect_2_form2_stage_1_cancers_25_posrisk_8_negrisk_1 | Patients | hypothetical |
| 0.089238 | 0.294128 | 0.615487 | pred_wait_2_detect_2_form2_stage_1_cancers_1_posrisk_8_negrisk_5 | Patients | hypothetical |
| 0.091829 | 0.304665 | 0.643274 | pred_wait_2_detect_2_form2_stage_1_cancers_5_posrisk_8_negrisk_5 | Patients | hypothetical |
| 0.095875 | 0.319858 | 0.679174 | pred_wait_2_detect_2_form2_stage_1_cancers_10_posrisk_8_negrisk_5 | Patients | hypothetical |
| 0.117168 | 0.376655 | 0.774488 | pred_wait_2_detect_2_form2_stage_1_cancers_25_posrisk_8_negrisk_5 | Patients | hypothetical |
| 0.089238 | 0.289556 | 0.60839 | pred_wait_2_detect_2_form2_stage_1_cancers_1_posrisk_8_negrisk_10 | Patients | hypothetical |
| 0.091829 | 0.299954 | 0.636821 | pred_wait_2_detect_2_form2_stage_1_cancers_5_posrisk_8_negrisk_10 | Patients | hypothetical |
| 0.095875 | 0.315062 | 0.673792 | pred_wait_2_detect_2_form2_stage_1_cancers_10_posrisk_8_negrisk_10 | Patients | hypothetical |
| 0.117168 | 0.372347 | 0.772624 | pred_wait_2_detect_2_form2_stage_1_cancers_25_posrisk_8_negrisk_10 | Patients | hypothetical |
| 0.089238 | 0.2708 | 0.555857 | pred_wait_2_detect_2_form2_stage_1_cancers_1_posrisk_8_negrisk_40 | Patients | hypothetical |
| 0.091829 | 0.279935 | 0.579561 | pred_wait_2_detect_2_form2_stage_1_cancers_5_posrisk_8_negrisk_40 | Patients | hypothetical |
| 0.095875 | 0.293398 | 0.611993 | pred_wait_2_detect_2_form2_stage_1_cancers_10_posrisk_8_negrisk_40 | Patients | hypothetical |
| 0.117168 | 0.34658 | 0.707796 | pred_wait_2_detect_2_form2_stage_1_cancers_25_posrisk_8_negrisk_40 | Patients | hypothetical |
| 0.177685 | 0.402604 | 0.630238 | pred_wait_2_detect_2_form2_stage_2_cancers_1_posrisk_2_negrisk_1 | Patients | hypothetical |
| 0.182846 | 0.418716 | 0.657271 | pred_wait_2_detect_2_form2_stage_2_cancers_5_posrisk_2_negrisk_1 | Patients | hypothetical |
| 0.191994 | 0.441408 | 0.692158 | pred_wait_2_detect_2_form2_stage_2_cancers_10_posrisk_2_negrisk_1 | Patients | hypothetical |
| 0.246904 | 0.52136 | 0.783577 | pred_wait_2_detect_2_form2_stage_2_cancers_25_posrisk_2_negrisk_1 | Patients | hypothetical |
| 0.172999 | 0.39881 | 0.628372 | pred_wait_2_detect_2_form2_stage_2_cancers_1_posrisk_2_negrisk_5 | Patients | hypothetical |
| 0.177665 | 0.414849 | 0.655619 | pred_wait_2_detect_2_form2_stage_2_cancers_5_posrisk_2_negrisk_5 | Patients | hypothetical |
| 0.186554 | 0.437535 | 0.690041 | pred_wait_2_detect_2_form2_stage_2_cancers_10_posrisk_2_negrisk_5 | Patients | hypothetical |
| 0.240509 | 0.518117 | 0.783733 | pred_wait_2_detect_2_form2_stage_2_cancers_25_posrisk_2_negrisk_5 | Patients | hypothetical |
| 0.166163 | 0.394238 | 0.625924 | pred_wait_2_detect_2_form2_stage_2_cancers_1_posrisk_2_negrisk_10 | Patients | hypothetical |
| 0.169755 | 0.410138 | 0.652845 | pred_wait_2_detect_2_form2_stage_2_cancers_5_posrisk_2_negrisk_10 | Patients | hypothetical |
| 0.177252 | 0.432739 | 0.688226 | pred_wait_2_detect_2_form2_stage_2_cancers_10_posrisk_2_negrisk_10 | Patients | hypothetical |
| 0.228034 | 0.513809 | 0.782569 | pred_wait_2_detect_2_form2_stage_2_cancers_25_posrisk_2_negrisk_10 | Patients | hypothetical |
| 0.147697 | 0.375481 | 0.619559 | pred_wait_2_detect_2_form2_stage_2_cancers_1_posrisk_2_negrisk_40 | Patients | hypothetical |
| 0.150532 | 0.39012 | 0.646715 | pred_wait_2_detect_2_form2_stage_2_cancers_5_posrisk_2_negrisk_40 | Patients | hypothetical |
| 0.155941 | 0.411075 | 0.681777 | pred_wait_2_detect_2_form2_stage_2_cancers_10_posrisk_2_negrisk_40 | Patients | hypothetical |
| 0.198626 | 0.488042 | 0.774579 | pred_wait_2_detect_2_form2_stage_2_cancers_25_posrisk_2_negrisk_40 | Patients | hypothetical |
| 0.110715 | 0.343384 | 0.620335 | pred_wait_2_detect_2_form2_stage_2_cancers_1_posrisk_4_negrisk_1 | Patients | hypothetical |
| 0.111862 | 0.356254 | 0.647194 | pred_wait_2_detect_2_form2_stage_2_cancers_5_posrisk_4_negrisk_1 | Patients | hypothetical |
| 0.115167 | 0.374947 | 0.681878 | pred_wait_2_detect_2_form2_stage_2_cancers_10_posrisk_4_negrisk_1 | Patients | hypothetical |
| 0.147273 | 0.446215 | 0.774045 | pred_wait_2_detect_2_form2_stage_2_cancers_25_posrisk_4_negrisk_1 | Patients | hypothetical |
| 0.110715 | 0.33959 | 0.615487 | pred_wait_2_detect_2_form2_stage_2_cancers_1_posrisk_4_negrisk_5 | Patients | hypothetical |
| 0.111862 | 0.352387 | 0.643274 | pred_wait_2_detect_2_form2_stage_2_cancers_5_posrisk_4_negrisk_5 | Patients | hypothetical |
| 0.115167 | 0.371075 | 0.679174 | pred_wait_2_detect_2_form2_stage_2_cancers_10_posrisk_4_negrisk_5 | Patients | hypothetical |
| 0.147273 | 0.442972 | 0.774488 | pred_wait_2_detect_2_form2_stage_2_cancers_25_posrisk_4_negrisk_5 | Patients | hypothetical |
| 0.110715 | 0.335018 | 0.60839 | pred_wait_2_detect_2_form2_stage_2_cancers_1_posrisk_4_negrisk_10 | Patients | hypothetical |
| 0.111862 | 0.347676 | 0.636821 | pred_wait_2_detect_2_form2_stage_2_cancers_5_posrisk_4_negrisk_10 | Patients | hypothetical |
| 0.115167 | 0.366279 | 0.673792 | pred_wait_2_detect_2_form2_stage_2_cancers_10_posrisk_4_negrisk_10 | Patients | hypothetical |
| 0.147273 | 0.438664 | 0.772624 | pred_wait_2_detect_2_form2_stage_2_cancers_25_posrisk_4_negrisk_10 | Patients | hypothetical |
| 0.110618 | 0.316262 | 0.555857 | pred_wait_2_detect_2_form2_stage_2_cancers_1_posrisk_4_negrisk_40 | Patients | hypothetical |
| 0.111767 | 0.327657 | 0.579561 | pred_wait_2_detect_2_form2_stage_2_cancers_5_posrisk_4_negrisk_40 | Patients | hypothetical |
| 0.115153 | 0.344615 | 0.611993 | pred_wait_2_detect_2_form2_stage_2_cancers_10_posrisk_4_negrisk_40 | Patients | hypothetical |
| 0.147273 | 0.412897 | 0.707796 | pred_wait_2_detect_2_form2_stage_2_cancers_25_posrisk_4_negrisk_40 | Patients | hypothetical |
| 0.089891 | 0.31147 | 0.620335 | pred_wait_2_detect_2_form2_stage_2_cancers_1_posrisk_6_negrisk_1 | Patients | hypothetical |
| 0.091778 | 0.322679 | 0.647194 | pred_wait_2_detect_2_form2_stage_2_cancers_5_posrisk_6_negrisk_1 | Patients | hypothetical |
| 0.095358 | 0.338942 | 0.681878 | pred_wait_2_detect_2_form2_stage_2_cancers_10_posrisk_6_negrisk_1 | Patients | hypothetical |
| 0.120116 | 0.40078 | 0.774045 | pred_wait_2_detect_2_form2_stage_2_cancers_25_posrisk_6_negrisk_1 | Patients | hypothetical |
| 0.089891 | 0.307676 | 0.615487 | pred_wait_2_detect_2_form2_stage_2_cancers_1_posrisk_6_negrisk_5 | Patients | hypothetical |
| 0.091778 | 0.318812 | 0.643274 | pred_wait_2_detect_2_form2_stage_2_cancers_5_posrisk_6_negrisk_5 | Patients | hypothetical |
| 0.095358 | 0.335069 | 0.679174 | pred_wait_2_detect_2_form2_stage_2_cancers_10_posrisk_6_negrisk_5 | Patients | hypothetical |
| 0.120116 | 0.397537 | 0.774488 | pred_wait_2_detect_2_form2_stage_2_cancers_25_posrisk_6_negrisk_5 | Patients | hypothetical |
| 0.089891 | 0.303104 | 0.60839 | pred_wait_2_detect_2_form2_stage_2_cancers_1_posrisk_6_negrisk_10 | Patients | hypothetical |
| 0.091778 | 0.314101 | 0.636821 | pred_wait_2_detect_2_form2_stage_2_cancers_5_posrisk_6_negrisk_10 | Patients | hypothetical |
| 0.095358 | 0.330273 | 0.673792 | pred_wait_2_detect_2_form2_stage_2_cancers_10_posrisk_6_negrisk_10 | Patients | hypothetical |
| 0.120116 | 0.393229 | 0.772624 | pred_wait_2_detect_2_form2_stage_2_cancers_25_posrisk_6_negrisk_10 | Patients | hypothetical |
| 0.089891 | 0.284348 | 0.555857 | pred_wait_2_detect_2_form2_stage_2_cancers_1_posrisk_6_negrisk_40 | Patients | hypothetical |
| 0.091778 | 0.294083 | 0.579561 | pred_wait_2_detect_2_form2_stage_2_cancers_5_posrisk_6_negrisk_40 | Patients | hypothetical |
| 0.095358 | 0.308609 | 0.611993 | pred_wait_2_detect_2_form2_stage_2_cancers_10_posrisk_6_negrisk_40 | Patients | hypothetical |
| 0.120116 | 0.367462 | 0.707796 | pred_wait_2_detect_2_form2_stage_2_cancers_25_posrisk_6_negrisk_40 | Patients | hypothetical |
| 0.089238 | 0.297922 | 0.620335 | pred_wait_2_detect_2_form2_stage_2_cancers_1_posrisk_8_negrisk_1 | Patients | hypothetical |
| 0.091829 | 0.308532 | 0.647194 | pred_wait_2_detect_2_form2_stage_2_cancers_5_posrisk_8_negrisk_1 | Patients | hypothetical |
| 0.095875 | 0.323731 | 0.681878 | pred_wait_2_detect_2_form2_stage_2_cancers_10_posrisk_8_negrisk_1 | Patients | hypothetical |
| 0.117168 | 0.379898 | 0.774045 | pred_wait_2_detect_2_form2_stage_2_cancers_25_posrisk_8_negrisk_1 | Patients | hypothetical |
| 0.089238 | 0.294128 | 0.615487 | pred_wait_2_detect_2_form2_stage_2_cancers_1_posrisk_8_negrisk_5 | Patients | hypothetical |
| 0.091829 | 0.304665 | 0.643274 | pred_wait_2_detect_2_form2_stage_2_cancers_5_posrisk_8_negrisk_5 | Patients | hypothetical |
| 0.095875 | 0.319858 | 0.679174 | pred_wait_2_detect_2_form2_stage_2_cancers_10_posrisk_8_negrisk_5 | Patients | hypothetical |
| 0.117168 | 0.376655 | 0.774488 | pred_wait_2_detect_2_form2_stage_2_cancers_25_posrisk_8_negrisk_5 | Patients | hypothetical |
| 0.089238 | 0.289556 | 0.60839 | pred_wait_2_detect_2_form2_stage_2_cancers_1_posrisk_8_negrisk_10 | Patients | hypothetical |
| 0.091829 | 0.299954 | 0.636821 | pred_wait_2_detect_2_form2_stage_2_cancers_5_posrisk_8_negrisk_10 | Patients | hypothetical |
| 0.095875 | 0.315062 | 0.673792 | pred_wait_2_detect_2_form2_stage_2_cancers_10_posrisk_8_negrisk_10 | Patients | hypothetical |
| 0.117168 | 0.372347 | 0.772624 | pred_wait_2_detect_2_form2_stage_2_cancers_25_posrisk_8_negrisk_10 | Patients | hypothetical |
| 0.089238 | 0.2708 | 0.555857 | pred_wait_2_detect_2_form2_stage_2_cancers_1_posrisk_8_negrisk_40 | Patients | hypothetical |
| 0.091829 | 0.279935 | 0.579561 | pred_wait_2_detect_2_form2_stage_2_cancers_5_posrisk_8_negrisk_40 | Patients | hypothetical |
| 0.095875 | 0.293398 | 0.611993 | pred_wait_2_detect_2_form2_stage_2_cancers_10_posrisk_8_negrisk_40 | Patients | hypothetical |
| 0.117168 | 0.34658 | 0.707796 | pred_wait_2_detect_2_form2_stage_2_cancers_25_posrisk_8_negrisk_40 | Patients | hypothetical |
| 0.177685 | 0.402604 | 0.630238 | pred_wait_2_detect_2_form3_stage_1_cancers_1_posrisk_2_negrisk_1 | Patients | hypothetical |
| 0.182846 | 0.418716 | 0.657271 | pred_wait_2_detect_2_form3_stage_1_cancers_5_posrisk_2_negrisk_1 | Patients | hypothetical |
| 0.191994 | 0.441408 | 0.692158 | pred_wait_2_detect_2_form3_stage_1_cancers_10_posrisk_2_negrisk_1 | Patients | hypothetical |
| 0.246904 | 0.52136 | 0.783577 | pred_wait_2_detect_2_form3_stage_1_cancers_25_posrisk_2_negrisk_1 | Patients | hypothetical |
| 0.172999 | 0.39881 | 0.628372 | pred_wait_2_detect_2_form3_stage_1_cancers_1_posrisk_2_negrisk_5 | Patients | hypothetical |
| 0.177665 | 0.414849 | 0.655619 | pred_wait_2_detect_2_form3_stage_1_cancers_5_posrisk_2_negrisk_5 | Patients | hypothetical |
| 0.186554 | 0.437535 | 0.690041 | pred_wait_2_detect_2_form3_stage_1_cancers_10_posrisk_2_negrisk_5 | Patients | hypothetical |
| 0.240509 | 0.518117 | 0.783733 | pred_wait_2_detect_2_form3_stage_1_cancers_25_posrisk_2_negrisk_5 | Patients | hypothetical |
| 0.166163 | 0.394238 | 0.625924 | pred_wait_2_detect_2_form3_stage_1_cancers_1_posrisk_2_negrisk_10 | Patients | hypothetical |
| 0.169755 | 0.410138 | 0.652845 | pred_wait_2_detect_2_form3_stage_1_cancers_5_posrisk_2_negrisk_10 | Patients | hypothetical |
| 0.177252 | 0.432739 | 0.688226 | pred_wait_2_detect_2_form3_stage_1_cancers_10_posrisk_2_negrisk_10 | Patients | hypothetical |
| 0.228034 | 0.513809 | 0.782569 | pred_wait_2_detect_2_form3_stage_1_cancers_25_posrisk_2_negrisk_10 | Patients | hypothetical |
| 0.147697 | 0.375481 | 0.619559 | pred_wait_2_detect_2_form3_stage_1_cancers_1_posrisk_2_negrisk_40 | Patients | hypothetical |
| 0.150532 | 0.39012 | 0.646715 | pred_wait_2_detect_2_form3_stage_1_cancers_5_posrisk_2_negrisk_40 | Patients | hypothetical |
| 0.155941 | 0.411075 | 0.681777 | pred_wait_2_detect_2_form3_stage_1_cancers_10_posrisk_2_negrisk_40 | Patients | hypothetical |
| 0.198626 | 0.488042 | 0.774579 | pred_wait_2_detect_2_form3_stage_1_cancers_25_posrisk_2_negrisk_40 | Patients | hypothetical |
| 0.110715 | 0.343384 | 0.620335 | pred_wait_2_detect_2_form3_stage_1_cancers_1_posrisk_4_negrisk_1 | Patients | hypothetical |
| 0.111862 | 0.356254 | 0.647194 | pred_wait_2_detect_2_form3_stage_1_cancers_5_posrisk_4_negrisk_1 | Patients | hypothetical |
| 0.115167 | 0.374947 | 0.681878 | pred_wait_2_detect_2_form3_stage_1_cancers_10_posrisk_4_negrisk_1 | Patients | hypothetical |
| 0.147273 | 0.446215 | 0.774045 | pred_wait_2_detect_2_form3_stage_1_cancers_25_posrisk_4_negrisk_1 | Patients | hypothetical |
| 0.110715 | 0.33959 | 0.615487 | pred_wait_2_detect_2_form3_stage_1_cancers_1_posrisk_4_negrisk_5 | Patients | hypothetical |
| 0.111862 | 0.352387 | 0.643274 | pred_wait_2_detect_2_form3_stage_1_cancers_5_posrisk_4_negrisk_5 | Patients | hypothetical |
| 0.115167 | 0.371075 | 0.679174 | pred_wait_2_detect_2_form3_stage_1_cancers_10_posrisk_4_negrisk_5 | Patients | hypothetical |
| 0.147273 | 0.442972 | 0.774488 | pred_wait_2_detect_2_form3_stage_1_cancers_25_posrisk_4_negrisk_5 | Patients | hypothetical |
| 0.110715 | 0.335018 | 0.60839 | pred_wait_2_detect_2_form3_stage_1_cancers_1_posrisk_4_negrisk_10 | Patients | hypothetical |
| 0.111862 | 0.347676 | 0.636821 | pred_wait_2_detect_2_form3_stage_1_cancers_5_posrisk_4_negrisk_10 | Patients | hypothetical |
| 0.115167 | 0.366279 | 0.673792 | pred_wait_2_detect_2_form3_stage_1_cancers_10_posrisk_4_negrisk_10 | Patients | hypothetical |
| 0.147273 | 0.438664 | 0.772624 | pred_wait_2_detect_2_form3_stage_1_cancers_25_posrisk_4_negrisk_10 | Patients | hypothetical |
| 0.110618 | 0.316262 | 0.555857 | pred_wait_2_detect_2_form3_stage_1_cancers_1_posrisk_4_negrisk_40 | Patients | hypothetical |
| 0.111767 | 0.327657 | 0.579561 | pred_wait_2_detect_2_form3_stage_1_cancers_5_posrisk_4_negrisk_40 | Patients | hypothetical |
| 0.115153 | 0.344615 | 0.611993 | pred_wait_2_detect_2_form3_stage_1_cancers_10_posrisk_4_negrisk_40 | Patients | hypothetical |
| 0.147273 | 0.412897 | 0.707796 | pred_wait_2_detect_2_form3_stage_1_cancers_25_posrisk_4_negrisk_40 | Patients | hypothetical |
| 0.089891 | 0.31147 | 0.620335 | pred_wait_2_detect_2_form3_stage_1_cancers_1_posrisk_6_negrisk_1 | Patients | hypothetical |
| 0.091778 | 0.322679 | 0.647194 | pred_wait_2_detect_2_form3_stage_1_cancers_5_posrisk_6_negrisk_1 | Patients | hypothetical |
| 0.095358 | 0.338942 | 0.681878 | pred_wait_2_detect_2_form3_stage_1_cancers_10_posrisk_6_negrisk_1 | Patients | hypothetical |
| 0.120116 | 0.40078 | 0.774045 | pred_wait_2_detect_2_form3_stage_1_cancers_25_posrisk_6_negrisk_1 | Patients | hypothetical |
| 0.089891 | 0.307676 | 0.615487 | pred_wait_2_detect_2_form3_stage_1_cancers_1_posrisk_6_negrisk_5 | Patients | hypothetical |
| 0.091778 | 0.318812 | 0.643274 | pred_wait_2_detect_2_form3_stage_1_cancers_5_posrisk_6_negrisk_5 | Patients | hypothetical |
| 0.095358 | 0.335069 | 0.679174 | pred_wait_2_detect_2_form3_stage_1_cancers_10_posrisk_6_negrisk_5 | Patients | hypothetical |
| 0.120116 | 0.397537 | 0.774488 | pred_wait_2_detect_2_form3_stage_1_cancers_25_posrisk_6_negrisk_5 | Patients | hypothetical |
| 0.089891 | 0.303104 | 0.60839 | pred_wait_2_detect_2_form3_stage_1_cancers_1_posrisk_6_negrisk_10 | Patients | hypothetical |
| 0.091778 | 0.314101 | 0.636821 | pred_wait_2_detect_2_form3_stage_1_cancers_5_posrisk_6_negrisk_10 | Patients | hypothetical |
| 0.095358 | 0.330273 | 0.673792 | pred_wait_2_detect_2_form3_stage_1_cancers_10_posrisk_6_negrisk_10 | Patients | hypothetical |
| 0.120116 | 0.393229 | 0.772624 | pred_wait_2_detect_2_form3_stage_1_cancers_25_posrisk_6_negrisk_10 | Patients | hypothetical |
| 0.089891 | 0.284348 | 0.555857 | pred_wait_2_detect_2_form3_stage_1_cancers_1_posrisk_6_negrisk_40 | Patients | hypothetical |
| 0.091778 | 0.294083 | 0.579561 | pred_wait_2_detect_2_form3_stage_1_cancers_5_posrisk_6_negrisk_40 | Patients | hypothetical |
| 0.095358 | 0.308609 | 0.611993 | pred_wait_2_detect_2_form3_stage_1_cancers_10_posrisk_6_negrisk_40 | Patients | hypothetical |
| 0.120116 | 0.367462 | 0.707796 | pred_wait_2_detect_2_form3_stage_1_cancers_25_posrisk_6_negrisk_40 | Patients | hypothetical |
| 0.089238 | 0.297922 | 0.620335 | pred_wait_2_detect_2_form3_stage_1_cancers_1_posrisk_8_negrisk_1 | Patients | hypothetical |
| 0.091829 | 0.308532 | 0.647194 | pred_wait_2_detect_2_form3_stage_1_cancers_5_posrisk_8_negrisk_1 | Patients | hypothetical |
| 0.095875 | 0.323731 | 0.681878 | pred_wait_2_detect_2_form3_stage_1_cancers_10_posrisk_8_negrisk_1 | Patients | hypothetical |
| 0.117168 | 0.379898 | 0.774045 | pred_wait_2_detect_2_form3_stage_1_cancers_25_posrisk_8_negrisk_1 | Patients | hypothetical |
| 0.089238 | 0.294128 | 0.615487 | pred_wait_2_detect_2_form3_stage_1_cancers_1_posrisk_8_negrisk_5 | Patients | hypothetical |
| 0.091829 | 0.304665 | 0.643274 | pred_wait_2_detect_2_form3_stage_1_cancers_5_posrisk_8_negrisk_5 | Patients | hypothetical |
| 0.095875 | 0.319858 | 0.679174 | pred_wait_2_detect_2_form3_stage_1_cancers_10_posrisk_8_negrisk_5 | Patients | hypothetical |
| 0.117168 | 0.376655 | 0.774488 | pred_wait_2_detect_2_form3_stage_1_cancers_25_posrisk_8_negrisk_5 | Patients | hypothetical |
| 0.089238 | 0.289556 | 0.60839 | pred_wait_2_detect_2_form3_stage_1_cancers_1_posrisk_8_negrisk_10 | Patients | hypothetical |
| 0.091829 | 0.299954 | 0.636821 | pred_wait_2_detect_2_form3_stage_1_cancers_5_posrisk_8_negrisk_10 | Patients | hypothetical |
| 0.095875 | 0.315062 | 0.673792 | pred_wait_2_detect_2_form3_stage_1_cancers_10_posrisk_8_negrisk_10 | Patients | hypothetical |
| 0.117168 | 0.372347 | 0.772624 | pred_wait_2_detect_2_form3_stage_1_cancers_25_posrisk_8_negrisk_10 | Patients | hypothetical |
| 0.089238 | 0.2708 | 0.555857 | pred_wait_2_detect_2_form3_stage_1_cancers_1_posrisk_8_negrisk_40 | Patients | hypothetical |
| 0.091829 | 0.279935 | 0.579561 | pred_wait_2_detect_2_form3_stage_1_cancers_5_posrisk_8_negrisk_40 | Patients | hypothetical |
| 0.095875 | 0.293398 | 0.611993 | pred_wait_2_detect_2_form3_stage_1_cancers_10_posrisk_8_negrisk_40 | Patients | hypothetical |
| 0.117168 | 0.34658 | 0.707796 | pred_wait_2_detect_2_form3_stage_1_cancers_25_posrisk_8_negrisk_40 | Patients | hypothetical |
| 0.177685 | 0.402604 | 0.630238 | pred_wait_2_detect_2_form3_stage_2_cancers_1_posrisk_2_negrisk_1 | Patients | hypothetical |
| 0.182846 | 0.418716 | 0.657271 | pred_wait_2_detect_2_form3_stage_2_cancers_5_posrisk_2_negrisk_1 | Patients | hypothetical |
| 0.191994 | 0.441408 | 0.692158 | pred_wait_2_detect_2_form3_stage_2_cancers_10_posrisk_2_negrisk_1 | Patients | hypothetical |
| 0.246904 | 0.52136 | 0.783577 | pred_wait_2_detect_2_form3_stage_2_cancers_25_posrisk_2_negrisk_1 | Patients | hypothetical |
| 0.172999 | 0.39881 | 0.628372 | pred_wait_2_detect_2_form3_stage_2_cancers_1_posrisk_2_negrisk_5 | Patients | hypothetical |
| 0.177665 | 0.414849 | 0.655619 | pred_wait_2_detect_2_form3_stage_2_cancers_5_posrisk_2_negrisk_5 | Patients | hypothetical |
| 0.186554 | 0.437535 | 0.690041 | pred_wait_2_detect_2_form3_stage_2_cancers_10_posrisk_2_negrisk_5 | Patients | hypothetical |
| 0.240509 | 0.518117 | 0.783733 | pred_wait_2_detect_2_form3_stage_2_cancers_25_posrisk_2_negrisk_5 | Patients | hypothetical |
| 0.166163 | 0.394238 | 0.625924 | pred_wait_2_detect_2_form3_stage_2_cancers_1_posrisk_2_negrisk_10 | Patients | hypothetical |
| 0.169755 | 0.410138 | 0.652845 | pred_wait_2_detect_2_form3_stage_2_cancers_5_posrisk_2_negrisk_10 | Patients | hypothetical |
| 0.177252 | 0.432739 | 0.688226 | pred_wait_2_detect_2_form3_stage_2_cancers_10_posrisk_2_negrisk_10 | Patients | hypothetical |
| 0.228034 | 0.513809 | 0.782569 | pred_wait_2_detect_2_form3_stage_2_cancers_25_posrisk_2_negrisk_10 | Patients | hypothetical |
| 0.147697 | 0.375481 | 0.619559 | pred_wait_2_detect_2_form3_stage_2_cancers_1_posrisk_2_negrisk_40 | Patients | hypothetical |
| 0.150532 | 0.39012 | 0.646715 | pred_wait_2_detect_2_form3_stage_2_cancers_5_posrisk_2_negrisk_40 | Patients | hypothetical |
| 0.155941 | 0.411075 | 0.681777 | pred_wait_2_detect_2_form3_stage_2_cancers_10_posrisk_2_negrisk_40 | Patients | hypothetical |
| 0.198626 | 0.488042 | 0.774579 | pred_wait_2_detect_2_form3_stage_2_cancers_25_posrisk_2_negrisk_40 | Patients | hypothetical |
| 0.110715 | 0.343384 | 0.620335 | pred_wait_2_detect_2_form3_stage_2_cancers_1_posrisk_4_negrisk_1 | Patients | hypothetical |
| 0.111862 | 0.356254 | 0.647194 | pred_wait_2_detect_2_form3_stage_2_cancers_5_posrisk_4_negrisk_1 | Patients | hypothetical |
| 0.115167 | 0.374947 | 0.681878 | pred_wait_2_detect_2_form3_stage_2_cancers_10_posrisk_4_negrisk_1 | Patients | hypothetical |
| 0.147273 | 0.446215 | 0.774045 | pred_wait_2_detect_2_form3_stage_2_cancers_25_posrisk_4_negrisk_1 | Patients | hypothetical |
| 0.110715 | 0.33959 | 0.615487 | pred_wait_2_detect_2_form3_stage_2_cancers_1_posrisk_4_negrisk_5 | Patients | hypothetical |
| 0.111862 | 0.352387 | 0.643274 | pred_wait_2_detect_2_form3_stage_2_cancers_5_posrisk_4_negrisk_5 | Patients | hypothetical |
| 0.115167 | 0.371075 | 0.679174 | pred_wait_2_detect_2_form3_stage_2_cancers_10_posrisk_4_negrisk_5 | Patients | hypothetical |
| 0.147273 | 0.442972 | 0.774488 | pred_wait_2_detect_2_form3_stage_2_cancers_25_posrisk_4_negrisk_5 | Patients | hypothetical |
| 0.110715 | 0.335018 | 0.60839 | pred_wait_2_detect_2_form3_stage_2_cancers_1_posrisk_4_negrisk_10 | Patients | hypothetical |
| 0.111862 | 0.347676 | 0.636821 | pred_wait_2_detect_2_form3_stage_2_cancers_5_posrisk_4_negrisk_10 | Patients | hypothetical |
| 0.115167 | 0.366279 | 0.673792 | pred_wait_2_detect_2_form3_stage_2_cancers_10_posrisk_4_negrisk_10 | Patients | hypothetical |
| 0.147273 | 0.438664 | 0.772624 | pred_wait_2_detect_2_form3_stage_2_cancers_25_posrisk_4_negrisk_10 | Patients | hypothetical |
| 0.110618 | 0.316262 | 0.555857 | pred_wait_2_detect_2_form3_stage_2_cancers_1_posrisk_4_negrisk_40 | Patients | hypothetical |
| 0.111767 | 0.327657 | 0.579561 | pred_wait_2_detect_2_form3_stage_2_cancers_5_posrisk_4_negrisk_40 | Patients | hypothetical |
| 0.115153 | 0.344615 | 0.611993 | pred_wait_2_detect_2_form3_stage_2_cancers_10_posrisk_4_negrisk_40 | Patients | hypothetical |
| 0.147273 | 0.412897 | 0.707796 | pred_wait_2_detect_2_form3_stage_2_cancers_25_posrisk_4_negrisk_40 | Patients | hypothetical |
| 0.089891 | 0.31147 | 0.620335 | pred_wait_2_detect_2_form3_stage_2_cancers_1_posrisk_6_negrisk_1 | Patients | hypothetical |
| 0.091778 | 0.322679 | 0.647194 | pred_wait_2_detect_2_form3_stage_2_cancers_5_posrisk_6_negrisk_1 | Patients | hypothetical |
| 0.095358 | 0.338942 | 0.681878 | pred_wait_2_detect_2_form3_stage_2_cancers_10_posrisk_6_negrisk_1 | Patients | hypothetical |
| 0.120116 | 0.40078 | 0.774045 | pred_wait_2_detect_2_form3_stage_2_cancers_25_posrisk_6_negrisk_1 | Patients | hypothetical |
| 0.089891 | 0.307676 | 0.615487 | pred_wait_2_detect_2_form3_stage_2_cancers_1_posrisk_6_negrisk_5 | Patients | hypothetical |
| 0.091778 | 0.318812 | 0.643274 | pred_wait_2_detect_2_form3_stage_2_cancers_5_posrisk_6_negrisk_5 | Patients | hypothetical |
| 0.095358 | 0.335069 | 0.679174 | pred_wait_2_detect_2_form3_stage_2_cancers_10_posrisk_6_negrisk_5 | Patients | hypothetical |
| 0.120116 | 0.397537 | 0.774488 | pred_wait_2_detect_2_form3_stage_2_cancers_25_posrisk_6_negrisk_5 | Patients | hypothetical |
| 0.089891 | 0.303104 | 0.60839 | pred_wait_2_detect_2_form3_stage_2_cancers_1_posrisk_6_negrisk_10 | Patients | hypothetical |
| 0.091778 | 0.314101 | 0.636821 | pred_wait_2_detect_2_form3_stage_2_cancers_5_posrisk_6_negrisk_10 | Patients | hypothetical |
| 0.095358 | 0.330273 | 0.673792 | pred_wait_2_detect_2_form3_stage_2_cancers_10_posrisk_6_negrisk_10 | Patients | hypothetical |
| 0.120116 | 0.393229 | 0.772624 | pred_wait_2_detect_2_form3_stage_2_cancers_25_posrisk_6_negrisk_10 | Patients | hypothetical |
| 0.089891 | 0.284348 | 0.555857 | pred_wait_2_detect_2_form3_stage_2_cancers_1_posrisk_6_negrisk_40 | Patients | hypothetical |
| 0.091778 | 0.294083 | 0.579561 | pred_wait_2_detect_2_form3_stage_2_cancers_5_posrisk_6_negrisk_40 | Patients | hypothetical |
| 0.095358 | 0.308609 | 0.611993 | pred_wait_2_detect_2_form3_stage_2_cancers_10_posrisk_6_negrisk_40 | Patients | hypothetical |
| 0.120116 | 0.367462 | 0.707796 | pred_wait_2_detect_2_form3_stage_2_cancers_25_posrisk_6_negrisk_40 | Patients | hypothetical |
| 0.089238 | 0.297922 | 0.620335 | pred_wait_2_detect_2_form3_stage_2_cancers_1_posrisk_8_negrisk_1 | Patients | hypothetical |
| 0.091829 | 0.308532 | 0.647194 | pred_wait_2_detect_2_form3_stage_2_cancers_5_posrisk_8_negrisk_1 | Patients | hypothetical |
| 0.095875 | 0.323731 | 0.681878 | pred_wait_2_detect_2_form3_stage_2_cancers_10_posrisk_8_negrisk_1 | Patients | hypothetical |
| 0.117168 | 0.379898 | 0.774045 | pred_wait_2_detect_2_form3_stage_2_cancers_25_posrisk_8_negrisk_1 | Patients | hypothetical |
| 0.089238 | 0.294128 | 0.615487 | pred_wait_2_detect_2_form3_stage_2_cancers_1_posrisk_8_negrisk_5 | Patients | hypothetical |
| 0.091829 | 0.304665 | 0.643274 | pred_wait_2_detect_2_form3_stage_2_cancers_5_posrisk_8_negrisk_5 | Patients | hypothetical |
| 0.095875 | 0.319858 | 0.679174 | pred_wait_2_detect_2_form3_stage_2_cancers_10_posrisk_8_negrisk_5 | Patients | hypothetical |
| 0.117168 | 0.376655 | 0.774488 | pred_wait_2_detect_2_form3_stage_2_cancers_25_posrisk_8_negrisk_5 | Patients | hypothetical |
| 0.089238 | 0.289556 | 0.60839 | pred_wait_2_detect_2_form3_stage_2_cancers_1_posrisk_8_negrisk_10 | Patients | hypothetical |
| 0.091829 | 0.299954 | 0.636821 | pred_wait_2_detect_2_form3_stage_2_cancers_5_posrisk_8_negrisk_10 | Patients | hypothetical |
| 0.095875 | 0.315062 | 0.673792 | pred_wait_2_detect_2_form3_stage_2_cancers_10_posrisk_8_negrisk_10 | Patients | hypothetical |
| 0.117168 | 0.372347 | 0.772624 | pred_wait_2_detect_2_form3_stage_2_cancers_25_posrisk_8_negrisk_10 | Patients | hypothetical |
| 0.089238 | 0.2708 | 0.555857 | pred_wait_2_detect_2_form3_stage_2_cancers_1_posrisk_8_negrisk_40 | Patients | hypothetical |
| 0.091829 | 0.279935 | 0.579561 | pred_wait_2_detect_2_form3_stage_2_cancers_5_posrisk_8_negrisk_40 | Patients | hypothetical |
| 0.095875 | 0.293398 | 0.611993 | pred_wait_2_detect_2_form3_stage_2_cancers_10_posrisk_8_negrisk_40 | Patients | hypothetical |
| 0.117168 | 0.34658 | 0.707796 | pred_wait_2_detect_2_form3_stage_2_cancers_25_posrisk_8_negrisk_40 | Patients | hypothetical |
| 0.177685 | 0.402604 | 0.630238 | pred_wait_2_detect_2_form4_stage_1_cancers_1_posrisk_2_negrisk_1 | Patients | hypothetical |
| 0.182846 | 0.418716 | 0.657271 | pred_wait_2_detect_2_form4_stage_1_cancers_5_posrisk_2_negrisk_1 | Patients | hypothetical |
| 0.191994 | 0.441408 | 0.692158 | pred_wait_2_detect_2_form4_stage_1_cancers_10_posrisk_2_negrisk_1 | Patients | hypothetical |
| 0.246904 | 0.52136 | 0.783577 | pred_wait_2_detect_2_form4_stage_1_cancers_25_posrisk_2_negrisk_1 | Patients | hypothetical |
| 0.172999 | 0.39881 | 0.628372 | pred_wait_2_detect_2_form4_stage_1_cancers_1_posrisk_2_negrisk_5 | Patients | hypothetical |
| 0.177665 | 0.414849 | 0.655619 | pred_wait_2_detect_2_form4_stage_1_cancers_5_posrisk_2_negrisk_5 | Patients | hypothetical |
| 0.186554 | 0.437535 | 0.690041 | pred_wait_2_detect_2_form4_stage_1_cancers_10_posrisk_2_negrisk_5 | Patients | hypothetical |
| 0.240509 | 0.518117 | 0.783733 | pred_wait_2_detect_2_form4_stage_1_cancers_25_posrisk_2_negrisk_5 | Patients | hypothetical |
| 0.166163 | 0.394238 | 0.625924 | pred_wait_2_detect_2_form4_stage_1_cancers_1_posrisk_2_negrisk_10 | Patients | hypothetical |
| 0.169755 | 0.410138 | 0.652845 | pred_wait_2_detect_2_form4_stage_1_cancers_5_posrisk_2_negrisk_10 | Patients | hypothetical |
| 0.177252 | 0.432739 | 0.688226 | pred_wait_2_detect_2_form4_stage_1_cancers_10_posrisk_2_negrisk_10 | Patients | hypothetical |
| 0.228034 | 0.513809 | 0.782569 | pred_wait_2_detect_2_form4_stage_1_cancers_25_posrisk_2_negrisk_10 | Patients | hypothetical |
| 0.147697 | 0.375481 | 0.619559 | pred_wait_2_detect_2_form4_stage_1_cancers_1_posrisk_2_negrisk_40 | Patients | hypothetical |
| 0.150532 | 0.39012 | 0.646715 | pred_wait_2_detect_2_form4_stage_1_cancers_5_posrisk_2_negrisk_40 | Patients | hypothetical |
| 0.155941 | 0.411075 | 0.681777 | pred_wait_2_detect_2_form4_stage_1_cancers_10_posrisk_2_negrisk_40 | Patients | hypothetical |
| 0.198626 | 0.488042 | 0.774579 | pred_wait_2_detect_2_form4_stage_1_cancers_25_posrisk_2_negrisk_40 | Patients | hypothetical |
| 0.110715 | 0.343384 | 0.620335 | pred_wait_2_detect_2_form4_stage_1_cancers_1_posrisk_4_negrisk_1 | Patients | hypothetical |
| 0.111862 | 0.356254 | 0.647194 | pred_wait_2_detect_2_form4_stage_1_cancers_5_posrisk_4_negrisk_1 | Patients | hypothetical |
| 0.115167 | 0.374947 | 0.681878 | pred_wait_2_detect_2_form4_stage_1_cancers_10_posrisk_4_negrisk_1 | Patients | hypothetical |
| 0.147273 | 0.446215 | 0.774045 | pred_wait_2_detect_2_form4_stage_1_cancers_25_posrisk_4_negrisk_1 | Patients | hypothetical |
| 0.110715 | 0.33959 | 0.615487 | pred_wait_2_detect_2_form4_stage_1_cancers_1_posrisk_4_negrisk_5 | Patients | hypothetical |
| 0.111862 | 0.352387 | 0.643274 | pred_wait_2_detect_2_form4_stage_1_cancers_5_posrisk_4_negrisk_5 | Patients | hypothetical |
| 0.115167 | 0.371075 | 0.679174 | pred_wait_2_detect_2_form4_stage_1_cancers_10_posrisk_4_negrisk_5 | Patients | hypothetical |
| 0.147273 | 0.442972 | 0.774488 | pred_wait_2_detect_2_form4_stage_1_cancers_25_posrisk_4_negrisk_5 | Patients | hypothetical |
| 0.110715 | 0.335018 | 0.60839 | pred_wait_2_detect_2_form4_stage_1_cancers_1_posrisk_4_negrisk_10 | Patients | hypothetical |
| 0.111862 | 0.347676 | 0.636821 | pred_wait_2_detect_2_form4_stage_1_cancers_5_posrisk_4_negrisk_10 | Patients | hypothetical |
| 0.115167 | 0.366279 | 0.673792 | pred_wait_2_detect_2_form4_stage_1_cancers_10_posrisk_4_negrisk_10 | Patients | hypothetical |
| 0.147273 | 0.438664 | 0.772624 | pred_wait_2_detect_2_form4_stage_1_cancers_25_posrisk_4_negrisk_10 | Patients | hypothetical |
| 0.110618 | 0.316262 | 0.555857 | pred_wait_2_detect_2_form4_stage_1_cancers_1_posrisk_4_negrisk_40 | Patients | hypothetical |
| 0.111767 | 0.327657 | 0.579561 | pred_wait_2_detect_2_form4_stage_1_cancers_5_posrisk_4_negrisk_40 | Patients | hypothetical |
| 0.115153 | 0.344615 | 0.611993 | pred_wait_2_detect_2_form4_stage_1_cancers_10_posrisk_4_negrisk_40 | Patients | hypothetical |
| 0.147273 | 0.412897 | 0.707796 | pred_wait_2_detect_2_form4_stage_1_cancers_25_posrisk_4_negrisk_40 | Patients | hypothetical |
| 0.089891 | 0.31147 | 0.620335 | pred_wait_2_detect_2_form4_stage_1_cancers_1_posrisk_6_negrisk_1 | Patients | hypothetical |
| 0.091778 | 0.322679 | 0.647194 | pred_wait_2_detect_2_form4_stage_1_cancers_5_posrisk_6_negrisk_1 | Patients | hypothetical |
| 0.095358 | 0.338942 | 0.681878 | pred_wait_2_detect_2_form4_stage_1_cancers_10_posrisk_6_negrisk_1 | Patients | hypothetical |
| 0.120116 | 0.40078 | 0.774045 | pred_wait_2_detect_2_form4_stage_1_cancers_25_posrisk_6_negrisk_1 | Patients | hypothetical |
| 0.089891 | 0.307676 | 0.615487 | pred_wait_2_detect_2_form4_stage_1_cancers_1_posrisk_6_negrisk_5 | Patients | hypothetical |
| 0.091778 | 0.318812 | 0.643274 | pred_wait_2_detect_2_form4_stage_1_cancers_5_posrisk_6_negrisk_5 | Patients | hypothetical |
| 0.095358 | 0.335069 | 0.679174 | pred_wait_2_detect_2_form4_stage_1_cancers_10_posrisk_6_negrisk_5 | Patients | hypothetical |
| 0.120116 | 0.397537 | 0.774488 | pred_wait_2_detect_2_form4_stage_1_cancers_25_posrisk_6_negrisk_5 | Patients | hypothetical |
| 0.089891 | 0.303104 | 0.60839 | pred_wait_2_detect_2_form4_stage_1_cancers_1_posrisk_6_negrisk_10 | Patients | hypothetical |
| 0.091778 | 0.314101 | 0.636821 | pred_wait_2_detect_2_form4_stage_1_cancers_5_posrisk_6_negrisk_10 | Patients | hypothetical |
| 0.095358 | 0.330273 | 0.673792 | pred_wait_2_detect_2_form4_stage_1_cancers_10_posrisk_6_negrisk_10 | Patients | hypothetical |
| 0.120116 | 0.393229 | 0.772624 | pred_wait_2_detect_2_form4_stage_1_cancers_25_posrisk_6_negrisk_10 | Patients | hypothetical |
| 0.089891 | 0.284348 | 0.555857 | pred_wait_2_detect_2_form4_stage_1_cancers_1_posrisk_6_negrisk_40 | Patients | hypothetical |
| 0.091778 | 0.294083 | 0.579561 | pred_wait_2_detect_2_form4_stage_1_cancers_5_posrisk_6_negrisk_40 | Patients | hypothetical |
| 0.095358 | 0.308609 | 0.611993 | pred_wait_2_detect_2_form4_stage_1_cancers_10_posrisk_6_negrisk_40 | Patients | hypothetical |
| 0.120116 | 0.367462 | 0.707796 | pred_wait_2_detect_2_form4_stage_1_cancers_25_posrisk_6_negrisk_40 | Patients | hypothetical |
| 0.089238 | 0.297922 | 0.620335 | pred_wait_2_detect_2_form4_stage_1_cancers_1_posrisk_8_negrisk_1 | Patients | hypothetical |
| 0.091829 | 0.308532 | 0.647194 | pred_wait_2_detect_2_form4_stage_1_cancers_5_posrisk_8_negrisk_1 | Patients | hypothetical |
| 0.095875 | 0.323731 | 0.681878 | pred_wait_2_detect_2_form4_stage_1_cancers_10_posrisk_8_negrisk_1 | Patients | hypothetical |
| 0.117168 | 0.379898 | 0.774045 | pred_wait_2_detect_2_form4_stage_1_cancers_25_posrisk_8_negrisk_1 | Patients | hypothetical |
| 0.089238 | 0.294128 | 0.615487 | pred_wait_2_detect_2_form4_stage_1_cancers_1_posrisk_8_negrisk_5 | Patients | hypothetical |
| 0.091829 | 0.304665 | 0.643274 | pred_wait_2_detect_2_form4_stage_1_cancers_5_posrisk_8_negrisk_5 | Patients | hypothetical |
| 0.095875 | 0.319858 | 0.679174 | pred_wait_2_detect_2_form4_stage_1_cancers_10_posrisk_8_negrisk_5 | Patients | hypothetical |
| 0.117168 | 0.376655 | 0.774488 | pred_wait_2_detect_2_form4_stage_1_cancers_25_posrisk_8_negrisk_5 | Patients | hypothetical |
| 0.089238 | 0.289556 | 0.60839 | pred_wait_2_detect_2_form4_stage_1_cancers_1_posrisk_8_negrisk_10 | Patients | hypothetical |
| 0.091829 | 0.299954 | 0.636821 | pred_wait_2_detect_2_form4_stage_1_cancers_5_posrisk_8_negrisk_10 | Patients | hypothetical |
| 0.095875 | 0.315062 | 0.673792 | pred_wait_2_detect_2_form4_stage_1_cancers_10_posrisk_8_negrisk_10 | Patients | hypothetical |
| 0.117168 | 0.372347 | 0.772624 | pred_wait_2_detect_2_form4_stage_1_cancers_25_posrisk_8_negrisk_10 | Patients | hypothetical |
| 0.089238 | 0.2708 | 0.555857 | pred_wait_2_detect_2_form4_stage_1_cancers_1_posrisk_8_negrisk_40 | Patients | hypothetical |
| 0.091829 | 0.279935 | 0.579561 | pred_wait_2_detect_2_form4_stage_1_cancers_5_posrisk_8_negrisk_40 | Patients | hypothetical |
| 0.095875 | 0.293398 | 0.611993 | pred_wait_2_detect_2_form4_stage_1_cancers_10_posrisk_8_negrisk_40 | Patients | hypothetical |
| 0.117168 | 0.34658 | 0.707796 | pred_wait_2_detect_2_form4_stage_1_cancers_25_posrisk_8_negrisk_40 | Patients | hypothetical |
| 0.177685 | 0.402604 | 0.630238 | pred_wait_2_detect_2_form4_stage_2_cancers_1_posrisk_2_negrisk_1 | Patients | hypothetical |
| 0.182846 | 0.418716 | 0.657271 | pred_wait_2_detect_2_form4_stage_2_cancers_5_posrisk_2_negrisk_1 | Patients | hypothetical |
| 0.191994 | 0.441408 | 0.692158 | pred_wait_2_detect_2_form4_stage_2_cancers_10_posrisk_2_negrisk_1 | Patients | hypothetical |
| 0.246904 | 0.52136 | 0.783577 | pred_wait_2_detect_2_form4_stage_2_cancers_25_posrisk_2_negrisk_1 | Patients | hypothetical |
| 0.172999 | 0.39881 | 0.628372 | pred_wait_2_detect_2_form4_stage_2_cancers_1_posrisk_2_negrisk_5 | Patients | hypothetical |
| 0.177665 | 0.414849 | 0.655619 | pred_wait_2_detect_2_form4_stage_2_cancers_5_posrisk_2_negrisk_5 | Patients | hypothetical |
| 0.186554 | 0.437535 | 0.690041 | pred_wait_2_detect_2_form4_stage_2_cancers_10_posrisk_2_negrisk_5 | Patients | hypothetical |
| 0.240509 | 0.518117 | 0.783733 | pred_wait_2_detect_2_form4_stage_2_cancers_25_posrisk_2_negrisk_5 | Patients | hypothetical |
| 0.166163 | 0.394238 | 0.625924 | pred_wait_2_detect_2_form4_stage_2_cancers_1_posrisk_2_negrisk_10 | Patients | hypothetical |
| 0.169755 | 0.410138 | 0.652845 | pred_wait_2_detect_2_form4_stage_2_cancers_5_posrisk_2_negrisk_10 | Patients | hypothetical |
| 0.177252 | 0.432739 | 0.688226 | pred_wait_2_detect_2_form4_stage_2_cancers_10_posrisk_2_negrisk_10 | Patients | hypothetical |
| 0.228034 | 0.513809 | 0.782569 | pred_wait_2_detect_2_form4_stage_2_cancers_25_posrisk_2_negrisk_10 | Patients | hypothetical |
| 0.147697 | 0.375481 | 0.619559 | pred_wait_2_detect_2_form4_stage_2_cancers_1_posrisk_2_negrisk_40 | Patients | hypothetical |
| 0.150532 | 0.39012 | 0.646715 | pred_wait_2_detect_2_form4_stage_2_cancers_5_posrisk_2_negrisk_40 | Patients | hypothetical |
| 0.155941 | 0.411075 | 0.681777 | pred_wait_2_detect_2_form4_stage_2_cancers_10_posrisk_2_negrisk_40 | Patients | hypothetical |
| 0.198626 | 0.488042 | 0.774579 | pred_wait_2_detect_2_form4_stage_2_cancers_25_posrisk_2_negrisk_40 | Patients | hypothetical |
| 0.110715 | 0.343384 | 0.620335 | pred_wait_2_detect_2_form4_stage_2_cancers_1_posrisk_4_negrisk_1 | Patients | hypothetical |
| 0.111862 | 0.356254 | 0.647194 | pred_wait_2_detect_2_form4_stage_2_cancers_5_posrisk_4_negrisk_1 | Patients | hypothetical |
| 0.115167 | 0.374947 | 0.681878 | pred_wait_2_detect_2_form4_stage_2_cancers_10_posrisk_4_negrisk_1 | Patients | hypothetical |
| 0.147273 | 0.446215 | 0.774045 | pred_wait_2_detect_2_form4_stage_2_cancers_25_posrisk_4_negrisk_1 | Patients | hypothetical |
| 0.110715 | 0.33959 | 0.615487 | pred_wait_2_detect_2_form4_stage_2_cancers_1_posrisk_4_negrisk_5 | Patients | hypothetical |
| 0.111862 | 0.352387 | 0.643274 | pred_wait_2_detect_2_form4_stage_2_cancers_5_posrisk_4_negrisk_5 | Patients | hypothetical |
| 0.115167 | 0.371075 | 0.679174 | pred_wait_2_detect_2_form4_stage_2_cancers_10_posrisk_4_negrisk_5 | Patients | hypothetical |
| 0.147273 | 0.442972 | 0.774488 | pred_wait_2_detect_2_form4_stage_2_cancers_25_posrisk_4_negrisk_5 | Patients | hypothetical |
| 0.110715 | 0.335018 | 0.60839 | pred_wait_2_detect_2_form4_stage_2_cancers_1_posrisk_4_negrisk_10 | Patients | hypothetical |
| 0.111862 | 0.347676 | 0.636821 | pred_wait_2_detect_2_form4_stage_2_cancers_5_posrisk_4_negrisk_10 | Patients | hypothetical |
| 0.115167 | 0.366279 | 0.673792 | pred_wait_2_detect_2_form4_stage_2_cancers_10_posrisk_4_negrisk_10 | Patients | hypothetical |
| 0.147273 | 0.438664 | 0.772624 | pred_wait_2_detect_2_form4_stage_2_cancers_25_posrisk_4_negrisk_10 | Patients | hypothetical |
| 0.110618 | 0.316262 | 0.555857 | pred_wait_2_detect_2_form4_stage_2_cancers_1_posrisk_4_negrisk_40 | Patients | hypothetical |
| 0.111767 | 0.327657 | 0.579561 | pred_wait_2_detect_2_form4_stage_2_cancers_5_posrisk_4_negrisk_40 | Patients | hypothetical |
| 0.115153 | 0.344615 | 0.611993 | pred_wait_2_detect_2_form4_stage_2_cancers_10_posrisk_4_negrisk_40 | Patients | hypothetical |
| 0.147273 | 0.412897 | 0.707796 | pred_wait_2_detect_2_form4_stage_2_cancers_25_posrisk_4_negrisk_40 | Patients | hypothetical |
| 0.089891 | 0.31147 | 0.620335 | pred_wait_2_detect_2_form4_stage_2_cancers_1_posrisk_6_negrisk_1 | Patients | hypothetical |
| 0.091778 | 0.322679 | 0.647194 | pred_wait_2_detect_2_form4_stage_2_cancers_5_posrisk_6_negrisk_1 | Patients | hypothetical |
| 0.095358 | 0.338942 | 0.681878 | pred_wait_2_detect_2_form4_stage_2_cancers_10_posrisk_6_negrisk_1 | Patients | hypothetical |
| 0.120116 | 0.40078 | 0.774045 | pred_wait_2_detect_2_form4_stage_2_cancers_25_posrisk_6_negrisk_1 | Patients | hypothetical |
| 0.089891 | 0.307676 | 0.615487 | pred_wait_2_detect_2_form4_stage_2_cancers_1_posrisk_6_negrisk_5 | Patients | hypothetical |
| 0.091778 | 0.318812 | 0.643274 | pred_wait_2_detect_2_form4_stage_2_cancers_5_posrisk_6_negrisk_5 | Patients | hypothetical |
| 0.095358 | 0.335069 | 0.679174 | pred_wait_2_detect_2_form4_stage_2_cancers_10_posrisk_6_negrisk_5 | Patients | hypothetical |
| 0.120116 | 0.397537 | 0.774488 | pred_wait_2_detect_2_form4_stage_2_cancers_25_posrisk_6_negrisk_5 | Patients | hypothetical |
| 0.089891 | 0.303104 | 0.60839 | pred_wait_2_detect_2_form4_stage_2_cancers_1_posrisk_6_negrisk_10 | Patients | hypothetical |
| 0.091778 | 0.314101 | 0.636821 | pred_wait_2_detect_2_form4_stage_2_cancers_5_posrisk_6_negrisk_10 | Patients | hypothetical |
| 0.095358 | 0.330273 | 0.673792 | pred_wait_2_detect_2_form4_stage_2_cancers_10_posrisk_6_negrisk_10 | Patients | hypothetical |
| 0.120116 | 0.393229 | 0.772624 | pred_wait_2_detect_2_form4_stage_2_cancers_25_posrisk_6_negrisk_10 | Patients | hypothetical |
| 0.089891 | 0.284348 | 0.555857 | pred_wait_2_detect_2_form4_stage_2_cancers_1_posrisk_6_negrisk_40 | Patients | hypothetical |
| 0.091778 | 0.294083 | 0.579561 | pred_wait_2_detect_2_form4_stage_2_cancers_5_posrisk_6_negrisk_40 | Patients | hypothetical |
| 0.095358 | 0.308609 | 0.611993 | pred_wait_2_detect_2_form4_stage_2_cancers_10_posrisk_6_negrisk_40 | Patients | hypothetical |
| 0.120116 | 0.367462 | 0.707796 | pred_wait_2_detect_2_form4_stage_2_cancers_25_posrisk_6_negrisk_40 | Patients | hypothetical |
| 0.089238 | 0.297922 | 0.620335 | pred_wait_2_detect_2_form4_stage_2_cancers_1_posrisk_8_negrisk_1 | Patients | hypothetical |
| 0.091829 | 0.308532 | 0.647194 | pred_wait_2_detect_2_form4_stage_2_cancers_5_posrisk_8_negrisk_1 | Patients | hypothetical |
| 0.095875 | 0.323731 | 0.681878 | pred_wait_2_detect_2_form4_stage_2_cancers_10_posrisk_8_negrisk_1 | Patients | hypothetical |
| 0.117168 | 0.379898 | 0.774045 | pred_wait_2_detect_2_form4_stage_2_cancers_25_posrisk_8_negrisk_1 | Patients | hypothetical |
| 0.089238 | 0.294128 | 0.615487 | pred_wait_2_detect_2_form4_stage_2_cancers_1_posrisk_8_negrisk_5 | Patients | hypothetical |
| 0.091829 | 0.304665 | 0.643274 | pred_wait_2_detect_2_form4_stage_2_cancers_5_posrisk_8_negrisk_5 | Patients | hypothetical |
| 0.095875 | 0.319858 | 0.679174 | pred_wait_2_detect_2_form4_stage_2_cancers_10_posrisk_8_negrisk_5 | Patients | hypothetical |
| 0.117168 | 0.376655 | 0.774488 | pred_wait_2_detect_2_form4_stage_2_cancers_25_posrisk_8_negrisk_5 | Patients | hypothetical |
| 0.089238 | 0.289556 | 0.60839 | pred_wait_2_detect_2_form4_stage_2_cancers_1_posrisk_8_negrisk_10 | Patients | hypothetical |
| 0.091829 | 0.299954 | 0.636821 | pred_wait_2_detect_2_form4_stage_2_cancers_5_posrisk_8_negrisk_10 | Patients | hypothetical |
| 0.095875 | 0.315062 | 0.673792 | pred_wait_2_detect_2_form4_stage_2_cancers_10_posrisk_8_negrisk_10 | Patients | hypothetical |
| 0.117168 | 0.372347 | 0.772624 | pred_wait_2_detect_2_form4_stage_2_cancers_25_posrisk_8_negrisk_10 | Patients | hypothetical |
| 0.089238 | 0.2708 | 0.555857 | pred_wait_2_detect_2_form4_stage_2_cancers_1_posrisk_8_negrisk_40 | Patients | hypothetical |
| 0.091829 | 0.279935 | 0.579561 | pred_wait_2_detect_2_form4_stage_2_cancers_5_posrisk_8_negrisk_40 | Patients | hypothetical |
| 0.095875 | 0.293398 | 0.611993 | pred_wait_2_detect_2_form4_stage_2_cancers_10_posrisk_8_negrisk_40 | Patients | hypothetical |
| 0.117168 | 0.34658 | 0.707796 | pred_wait_2_detect_2_form4_stage_2_cancers_25_posrisk_8_negrisk_40 | Patients | hypothetical |
| 0.089238 | 0.294128 | 0.615487 | pred_wait_2_detect_1_form1_stage_2_cancers_1_posrisk_8_negrisk_5 | Patients | FIT |
| 0.098712 | 0.318872 | 0.681147 | pred_wait_1_detect_1_form1_stage_2_cancers_10_posrisk_9_negrisk_2 | Patients | CA125 |
| 0.089238 | 0.28402 | 0.461358 | pred_wait_2_detect_2_form1_stage_2_cancers_1_posrisk_8_negrisk_240 | Patients | PSA |
| 0.020422 | 0.066498 | 0.126566 | pred_wait_1_detect_1_form1_stage_1_cancers_1_posrisk_8_negrisk_40 | GPs | hypothetical |
| 0.337389 | 0.533724 | 0.725643 | pred_wait_1_detect_1_form1_stage_1_cancers_5_posrisk_2_negrisk_1 | GPs | hypothetical |
| 0.386562 | 0.590536 | 0.781397 | pred_wait_1_detect_1_form1_stage_1_cancers_10_posrisk_2_negrisk_1 | GPs | hypothetical |
| 0.56791 | 0.754595 | 0.901387 | pred_wait_1_detect_1_form1_stage_1_cancers_25_posrisk_2_negrisk_1 | GPs | hypothetical |
| 0.264107 | 0.449719 | 0.640976 | pred_wait_1_detect_1_form1_stage_1_cancers_1_posrisk_2_negrisk_5 | GPs | hypothetical |
| 0.294761 | 0.491222 | 0.689509 | pred_wait_1_detect_1_form1_stage_1_cancers_5_posrisk_2_negrisk_5 | GPs | hypothetical |
| 0.339423 | 0.547987 | 0.749008 | pred_wait_1_detect_1_form1_stage_1_cancers_10_posrisk_2_negrisk_5 | GPs | hypothetical |
| 0.519522 | 0.721161 | 0.884091 | pred_wait_1_detect_1_form1_stage_1_cancers_25_posrisk_2_negrisk_5 | GPs | hypothetical |
| 0.216659 | 0.397211 | 0.589637 | pred_wait_1_detect_1_form1_stage_1_cancers_1_posrisk_2_negrisk_10 | GPs | hypothetical |
| 0.243033 | 0.436297 | 0.63862 | pred_wait_1_detect_1_form1_stage_1_cancers_5_posrisk_2_negrisk_10 | GPs | hypothetical |
| 0.282856 | 0.491391 | 0.701892 | pred_wait_1_detect_1_form1_stage_1_cancers_10_posrisk_2_negrisk_10 | GPs | hypothetical |
| 0.45484 | 0.672423 | 0.856132 | pred_wait_1_detect_1_form1_stage_1_cancers_25_posrisk_2_negrisk_10 | GPs | hypothetical |
| 0.056062 | 0.156536 | 0.284003 | pred_wait_1_detect_1_form1_stage_1_cancers_1_posrisk_2_negrisk_40 | GPs | hypothetical |
| 0.063175 | 0.172773 | 0.312522 | pred_wait_1_detect_1_form1_stage_1_cancers_5_posrisk_2_negrisk_40 | GPs | hypothetical |
| 0.075459 | 0.198764 | 0.355387 | pred_wait_1_detect_1_form1_stage_1_cancers_10_posrisk_2_negrisk_40 | GPs | hypothetical |
| 0.148482 | 0.323884 | 0.528296 | pred_wait_1_detect_1_form1_stage_1_cancers_25_posrisk_2_negrisk_40 | GPs | hypothetical |
| 0.109547 | 0.240304 | 0.394291 | pred_wait_1_detect_1_form1_stage_1_cancers_1_posrisk_4_negrisk_1 | GPs | hypothetical |
| 0.122251 | 0.26442 | 0.429584 | pred_wait_1_detect_1_form1_stage_1_cancers_5_posrisk_4_negrisk_1 | GPs | hypothetical |
| 0.142905 | 0.300534 | 0.480386 | pred_wait_1_detect_1_form1_stage_1_cancers_10_posrisk_4_negrisk_1 | GPs | hypothetical |
| 0.250066 | 0.446788 | 0.651691 | pred_wait_1_detect_1_form1_stage_1_cancers_25_posrisk_4_negrisk_1 | GPs | hypothetical |
| 0.087428 | 0.207584 | 0.353139 | pred_wait_1_detect_1_form1_stage_1_cancers_1_posrisk_4_negrisk_5 | GPs | hypothetical |
| 0.097824 | 0.228828 | 0.385715 | pred_wait_1_detect_1_form1_stage_1_cancers_5_posrisk_4_negrisk_5 | GPs | hypothetical |
| 0.114609 | 0.261381 | 0.434085 | pred_wait_1_detect_1_form1_stage_1_cancers_10_posrisk_4_negrisk_5 | GPs | hypothetical |
| 0.209791 | 0.401397 | 0.609054 | pred_wait_1_detect_1_form1_stage_1_cancers_25_posrisk_4_negrisk_5 | GPs | hypothetical |
| 0.066127 | 0.170744 | 0.304518 | pred_wait_1_detect_1_form1_stage_1_cancers_1_posrisk_4_negrisk_10 | GPs | hypothetical |
| 0.073137 | 0.188371 | 0.332958 | pred_wait_1_detect_1_form1_stage_1_cancers_5_posrisk_4_negrisk_10 | GPs | hypothetical |
| 0.086993 | 0.216151 | 0.376289 | pred_wait_1_detect_1_form1_stage_1_cancers_10_posrisk_4_negrisk_10 | GPs | hypothetical |
| 0.16464 | 0.345036 | 0.550102 | pred_wait_1_detect_1_form1_stage_1_cancers_25_posrisk_4_negrisk_10 | GPs | hypothetical |
| 0.011934 | 0.052231 | 0.115253 | pred_wait_1_detect_1_form1_stage_1_cancers_1_posrisk_4_negrisk_40 | GPs | hypothetical |
| 0.013272 | 0.056813 | 0.124409 | pred_wait_1_detect_1_form1_stage_1_cancers_5_posrisk_4_negrisk_40 | GPs | hypothetical |
| 0.015915 | 0.064978 | 0.140378 | pred_wait_1_detect_1_form1_stage_1_cancers_10_posrisk_4_negrisk_40 | GPs | hypothetical |
| 0.036407 | 0.11642 | 0.231362 | pred_wait_1_detect_1_form1_stage_1_cancers_25_posrisk_4_negrisk_40 | GPs | hypothetical |
| 0.04662 | 0.120473 | 0.217205 | pred_wait_1_detect_1_form1_stage_1_cancers_1_posrisk_6_negrisk_1 | GPs | hypothetical |
| 0.052975 | 0.131826 | 0.233437 | pred_wait_1_detect_1_form1_stage_1_cancers_5_posrisk_6_negrisk_1 | GPs | hypothetical |
| 0.061284 | 0.14935 | 0.259542 | pred_wait_1_detect_1_form1_stage_1_cancers_10_posrisk_6_negrisk_1 | GPs | hypothetical |
| 0.109005 | 0.22944 | 0.369791 | pred_wait_1_detect_1_form1_stage_1_cancers_25_posrisk_6_negrisk_1 | GPs | hypothetical |
| 0.036111 | 0.102505 | 0.191399 | pred_wait_1_detect_1_form1_stage_1_cancers_1_posrisk_6_negrisk_5 | GPs | hypothetical |
| 0.041064 | 0.112244 | 0.20671 | pred_wait_1_detect_1_form1_stage_1_cancers_5_posrisk_6_negrisk_5 | GPs | hypothetical |
| 0.04866 | 0.127537 | 0.22989 | pred_wait_1_detect_1_form1_stage_1_cancers_10_posrisk_6_negrisk_5 | GPs | hypothetical |
| 0.088753 | 0.200367 | 0.333966 | pred_wait_1_detect_1_form1_stage_1_cancers_25_posrisk_6_negrisk_5 | GPs | hypothetical |
| 0.026075 | 0.083252 | 0.161633 | pred_wait_1_detect_1_form1_stage_1_cancers_1_posrisk_6_negrisk_10 | GPs | hypothetical |
| 0.029829 | 0.091163 | 0.176037 | pred_wait_1_detect_1_form1_stage_1_cancers_5_posrisk_6_negrisk_10 | GPs | hypothetical |
| 0.035325 | 0.103858 | 0.195946 | pred_wait_1_detect_1_form1_stage_1_cancers_10_posrisk_6_negrisk_10 | GPs | hypothetical |
| 0.068687 | 0.167477 | 0.28956 | pred_wait_1_detect_1_form1_stage_1_cancers_25_posrisk_6_negrisk_10 | GPs | hypothetical |
| 0.004109 | 0.026114 | 0.064679 | pred_wait_1_detect_1_form1_stage_1_cancers_1_posrisk_6_negrisk_40 | GPs | hypothetical |
| 0.004675 | 0.02827 | 0.068103 | pred_wait_1_detect_1_form1_stage_1_cancers_5_posrisk_6_negrisk_40 | GPs | hypothetical |
| 0.005886 | 0.032088 | 0.07588 | pred_wait_1_detect_1_form1_stage_1_cancers_10_posrisk_6_negrisk_40 | GPs | hypothetical |
| 0.013687 | 0.055808 | 0.118819 | pred_wait_1_detect_1_form1_stage_1_cancers_25_posrisk_6_negrisk_40 | GPs | hypothetical |
| 0.026531 | 0.077031 | 0.14247 | pred_wait_1_detect_1_form1_stage_1_cancers_1_posrisk_8_negrisk_1 | GPs | hypothetical |
| 0.030154 | 0.083752 | 0.151369 | pred_wait_1_detect_1_form1_stage_1_cancers_5_posrisk_8_negrisk_1 | GPs | hypothetical |
| 0.035947 | 0.093946 | 0.167053 | pred_wait_1_detect_1_form1_stage_1_cancers_10_posrisk_8_negrisk_1 | GPs | hypothetical |
| 0.062542 | 0.139674 | 0.230739 | pred_wait_1_detect_1_form1_stage_1_cancers_25_posrisk_8_negrisk_1 | GPs | hypothetical |
| 0.020422 | 0.066498 | 0.126566 | pred_wait_1_detect_1_form1_stage_1_cancers_1_posrisk_8_negrisk_5 | GPs | hypothetical |
| 0.023755 | 0.072357 | 0.134879 | pred_wait_1_detect_1_form1_stage_1_cancers_5_posrisk_8_negrisk_5 | GPs | hypothetical |
| 0.02876 | 0.081356 | 0.148523 | pred_wait_1_detect_1_form1_stage_1_cancers_10_posrisk_8_negrisk_5 | GPs | hypothetical |
| 0.052063 | 0.122804 | 0.20864 | pred_wait_1_detect_1_form1_stage_1_cancers_25_posrisk_8_negrisk_5 | GPs | hypothetical |
| 0.014617 | 0.055196 | 0.108952 | pred_wait_1_detect_1_form1_stage_1_cancers_1_posrisk_8_negrisk_10 | GPs | hypothetical |
| 0.016834 | 0.060081 | 0.117257 | pred_wait_1_detect_1_form1_stage_1_cancers_5_posrisk_8_negrisk_10 | GPs | hypothetical |
| 0.020966 | 0.067707 | 0.129194 | pred_wait_1_detect_1_form1_stage_1_cancers_10_posrisk_8_negrisk_10 | GPs | hypothetical |
| 0.040592 | 0.104061 | 0.182106 | pred_wait_1_detect_1_form1_stage_1_cancers_25_posrisk_8_negrisk_10 | GPs | hypothetical |
| 0.002123 | 0.01975 | 0.050845 | pred_wait_1_detect_1_form1_stage_1_cancers_1_posrisk_8_negrisk_40 | GPs | hypothetical |
| 0.002481 | 0.021388 | 0.053261 | pred_wait_1_detect_1_form1_stage_1_cancers_5_posrisk_8_negrisk_40 | GPs | hypothetical |
| 0.003279 | 0.024122 | 0.058531 | pred_wait_1_detect_1_form1_stage_1_cancers_10_posrisk_8_negrisk_40 | GPs | hypothetical |
| 0.008088 | 0.039314 | 0.083522 | pred_wait_1_detect_1_form1_stage_1_cancers_25_posrisk_8_negrisk_40 | GPs | hypothetical |
| 0.303987 | 0.49117 | 0.680335 | pred_wait_1_detect_1_form1_stage_2_cancers_1_posrisk_2_negrisk_1 | GPs | hypothetical |
| 0.337389 | 0.533724 | 0.725643 | pred_wait_1_detect_1_form1_stage_2_cancers_5_posrisk_2_negrisk_1 | GPs | hypothetical |
| 0.386562 | 0.590536 | 0.781397 | pred_wait_1_detect_1_form1_stage_2_cancers_10_posrisk_2_negrisk_1 | GPs | hypothetical |
| 0.56791 | 0.754595 | 0.901387 | pred_wait_1_detect_1_form1_stage_2_cancers_25_posrisk_2_negrisk_1 | GPs | hypothetical |
| 0.264107 | 0.449719 | 0.640976 | pred_wait_1_detect_1_form1_stage_2_cancers_1_posrisk_2_negrisk_5 | GPs | hypothetical |
| 0.294761 | 0.491222 | 0.689509 | pred_wait_1_detect_1_form1_stage_2_cancers_5_posrisk_2_negrisk_5 | GPs | hypothetical |
| 0.339423 | 0.547987 | 0.749008 | pred_wait_1_detect_1_form1_stage_2_cancers_10_posrisk_2_negrisk_5 | GPs | hypothetical |
| 0.519522 | 0.721161 | 0.884091 | pred_wait_1_detect_1_form1_stage_2_cancers_25_posrisk_2_negrisk_5 | GPs | hypothetical |
| 0.216659 | 0.397211 | 0.589637 | pred_wait_1_detect_1_form1_stage_2_cancers_1_posrisk_2_negrisk_10 | GPs | hypothetical |
| 0.243033 | 0.436297 | 0.63862 | pred_wait_1_detect_1_form1_stage_2_cancers_5_posrisk_2_negrisk_10 | GPs | hypothetical |
| 0.282856 | 0.491391 | 0.701892 | pred_wait_1_detect_1_form1_stage_2_cancers_10_posrisk_2_negrisk_10 | GPs | hypothetical |
| 0.45484 | 0.672423 | 0.856132 | pred_wait_1_detect_1_form1_stage_2_cancers_25_posrisk_2_negrisk_10 | GPs | hypothetical |
| 0.056062 | 0.156536 | 0.284003 | pred_wait_1_detect_1_form1_stage_2_cancers_1_posrisk_2_negrisk_40 | GPs | hypothetical |
| 0.063175 | 0.172773 | 0.312522 | pred_wait_1_detect_1_form1_stage_2_cancers_5_posrisk_2_negrisk_40 | GPs | hypothetical |
| 0.075459 | 0.198764 | 0.355387 | pred_wait_1_detect_1_form1_stage_2_cancers_10_posrisk_2_negrisk_40 | GPs | hypothetical |
| 0.148482 | 0.323884 | 0.528296 | pred_wait_1_detect_1_form1_stage_2_cancers_25_posrisk_2_negrisk_40 | GPs | hypothetical |
| 0.109547 | 0.240304 | 0.394291 | pred_wait_1_detect_1_form1_stage_2_cancers_1_posrisk_4_negrisk_1 | GPs | hypothetical |
| 0.122251 | 0.26442 | 0.429584 | pred_wait_1_detect_1_form1_stage_2_cancers_5_posrisk_4_negrisk_1 | GPs | hypothetical |
| 0.142905 | 0.300534 | 0.480386 | pred_wait_1_detect_1_form1_stage_2_cancers_10_posrisk_4_negrisk_1 | GPs | hypothetical |
| 0.250066 | 0.446788 | 0.651691 | pred_wait_1_detect_1_form1_stage_2_cancers_25_posrisk_4_negrisk_1 | GPs | hypothetical |
| 0.087428 | 0.207584 | 0.353139 | pred_wait_1_detect_1_form1_stage_2_cancers_1_posrisk_4_negrisk_5 | GPs | hypothetical |
| 0.097824 | 0.228828 | 0.385715 | pred_wait_1_detect_1_form1_stage_2_cancers_5_posrisk_4_negrisk_5 | GPs | hypothetical |
| 0.114609 | 0.261381 | 0.434085 | pred_wait_1_detect_1_form1_stage_2_cancers_10_posrisk_4_negrisk_5 | GPs | hypothetical |
| 0.209791 | 0.401397 | 0.609054 | pred_wait_1_detect_1_form1_stage_2_cancers_25_posrisk_4_negrisk_5 | GPs | hypothetical |
| 0.066127 | 0.170744 | 0.304518 | pred_wait_1_detect_1_form1_stage_2_cancers_1_posrisk_4_negrisk_10 | GPs | hypothetical |
| 0.073137 | 0.188371 | 0.332958 | pred_wait_1_detect_1_form1_stage_2_cancers_5_posrisk_4_negrisk_10 | GPs | hypothetical |
| 0.086993 | 0.216151 | 0.376289 | pred_wait_1_detect_1_form1_stage_2_cancers_10_posrisk_4_negrisk_10 | GPs | hypothetical |
| 0.16464 | 0.345036 | 0.550102 | pred_wait_1_detect_1_form1_stage_2_cancers_25_posrisk_4_negrisk_10 | GPs | hypothetical |
| 0.011934 | 0.052231 | 0.115253 | pred_wait_1_detect_1_form1_stage_2_cancers_1_posrisk_4_negrisk_40 | GPs | hypothetical |
| 0.013272 | 0.056813 | 0.124409 | pred_wait_1_detect_1_form1_stage_2_cancers_5_posrisk_4_negrisk_40 | GPs | hypothetical |
| 0.015915 | 0.064978 | 0.140378 | pred_wait_1_detect_1_form1_stage_2_cancers_10_posrisk_4_negrisk_40 | GPs | hypothetical |
| 0.036407 | 0.11642 | 0.231362 | pred_wait_1_detect_1_form1_stage_2_cancers_25_posrisk_4_negrisk_40 | GPs | hypothetical |
| 0.04662 | 0.120473 | 0.217205 | pred_wait_1_detect_1_form1_stage_2_cancers_1_posrisk_6_negrisk_1 | GPs | hypothetical |
| 0.052975 | 0.131826 | 0.233437 | pred_wait_1_detect_1_form1_stage_2_cancers_5_posrisk_6_negrisk_1 | GPs | hypothetical |
| 0.061284 | 0.14935 | 0.259542 | pred_wait_1_detect_1_form1_stage_2_cancers_10_posrisk_6_negrisk_1 | GPs | hypothetical |
| 0.109005 | 0.22944 | 0.369791 | pred_wait_1_detect_1_form1_stage_2_cancers_25_posrisk_6_negrisk_1 | GPs | hypothetical |
| 0.036111 | 0.102505 | 0.191399 | pred_wait_1_detect_1_form1_stage_2_cancers_1_posrisk_6_negrisk_5 | GPs | hypothetical |
| 0.041064 | 0.112244 | 0.20671 | pred_wait_1_detect_1_form1_stage_2_cancers_5_posrisk_6_negrisk_5 | GPs | hypothetical |
| 0.04866 | 0.127537 | 0.22989 | pred_wait_1_detect_1_form1_stage_2_cancers_10_posrisk_6_negrisk_5 | GPs | hypothetical |
| 0.088753 | 0.200367 | 0.333966 | pred_wait_1_detect_1_form1_stage_2_cancers_25_posrisk_6_negrisk_5 | GPs | hypothetical |
| 0.026075 | 0.083252 | 0.161633 | pred_wait_1_detect_1_form1_stage_2_cancers_1_posrisk_6_negrisk_10 | GPs | hypothetical |
| 0.029829 | 0.091163 | 0.176037 | pred_wait_1_detect_1_form1_stage_2_cancers_5_posrisk_6_negrisk_10 | GPs | hypothetical |
| 0.035325 | 0.103858 | 0.195946 | pred_wait_1_detect_1_form1_stage_2_cancers_10_posrisk_6_negrisk_10 | GPs | hypothetical |
| 0.068687 | 0.167477 | 0.28956 | pred_wait_1_detect_1_form1_stage_2_cancers_25_posrisk_6_negrisk_10 | GPs | hypothetical |
| 0.004109 | 0.026114 | 0.064679 | pred_wait_1_detect_1_form1_stage_2_cancers_1_posrisk_6_negrisk_40 | GPs | hypothetical |
| 0.004675 | 0.02827 | 0.068103 | pred_wait_1_detect_1_form1_stage_2_cancers_5_posrisk_6_negrisk_40 | GPs | hypothetical |
| 0.005886 | 0.032088 | 0.07588 | pred_wait_1_detect_1_form1_stage_2_cancers_10_posrisk_6_negrisk_40 | GPs | hypothetical |
| 0.013687 | 0.055808 | 0.118819 | pred_wait_1_detect_1_form1_stage_2_cancers_25_posrisk_6_negrisk_40 | GPs | hypothetical |
| 0.026531 | 0.077031 | 0.14247 | pred_wait_1_detect_1_form1_stage_2_cancers_1_posrisk_8_negrisk_1 | GPs | hypothetical |
| 0.030154 | 0.083752 | 0.151369 | pred_wait_1_detect_1_form1_stage_2_cancers_5_posrisk_8_negrisk_1 | GPs | hypothetical |
| 0.035947 | 0.093946 | 0.167053 | pred_wait_1_detect_1_form1_stage_2_cancers_10_posrisk_8_negrisk_1 | GPs | hypothetical |
| 0.062542 | 0.139674 | 0.230739 | pred_wait_1_detect_1_form1_stage_2_cancers_25_posrisk_8_negrisk_1 | GPs | hypothetical |
| 0.020422 | 0.066498 | 0.126566 | pred_wait_1_detect_1_form1_stage_2_cancers_1_posrisk_8_negrisk_5 | GPs | hypothetical |
| 0.023755 | 0.072357 | 0.134879 | pred_wait_1_detect_1_form1_stage_2_cancers_5_posrisk_8_negrisk_5 | GPs | hypothetical |
| 0.02876 | 0.081356 | 0.148523 | pred_wait_1_detect_1_form1_stage_2_cancers_10_posrisk_8_negrisk_5 | GPs | hypothetical |
| 0.052063 | 0.122804 | 0.20864 | pred_wait_1_detect_1_form1_stage_2_cancers_25_posrisk_8_negrisk_5 | GPs | hypothetical |
| 0.014617 | 0.055196 | 0.108952 | pred_wait_1_detect_1_form1_stage_2_cancers_1_posrisk_8_negrisk_10 | GPs | hypothetical |
| 0.016834 | 0.060081 | 0.117257 | pred_wait_1_detect_1_form1_stage_2_cancers_5_posrisk_8_negrisk_10 | GPs | hypothetical |
| 0.020966 | 0.067707 | 0.129194 | pred_wait_1_detect_1_form1_stage_2_cancers_10_posrisk_8_negrisk_10 | GPs | hypothetical |
| 0.040592 | 0.104061 | 0.182106 | pred_wait_1_detect_1_form1_stage_2_cancers_25_posrisk_8_negrisk_10 | GPs | hypothetical |
| 0.002123 | 0.01975 | 0.050845 | pred_wait_1_detect_1_form1_stage_2_cancers_1_posrisk_8_negrisk_40 | GPs | hypothetical |
| 0.002481 | 0.021388 | 0.053261 | pred_wait_1_detect_1_form1_stage_2_cancers_5_posrisk_8_negrisk_40 | GPs | hypothetical |
| 0.003279 | 0.024122 | 0.058531 | pred_wait_1_detect_1_form1_stage_2_cancers_10_posrisk_8_negrisk_40 | GPs | hypothetical |
| 0.008088 | 0.039314 | 0.083522 | pred_wait_1_detect_1_form1_stage_2_cancers_25_posrisk_8_negrisk_40 | GPs | hypothetical |
| 0.303987 | 0.49117 | 0.680335 | pred_wait_1_detect_1_form2_stage_1_cancers_1_posrisk_2_negrisk_1 | GPs | hypothetical |
| 0.337389 | 0.533724 | 0.725643 | pred_wait_1_detect_1_form2_stage_1_cancers_5_posrisk_2_negrisk_1 | GPs | hypothetical |
| 0.386562 | 0.590536 | 0.781397 | pred_wait_1_detect_1_form2_stage_1_cancers_10_posrisk_2_negrisk_1 | GPs | hypothetical |
| 0.56791 | 0.754595 | 0.901387 | pred_wait_1_detect_1_form2_stage_1_cancers_25_posrisk_2_negrisk_1 | GPs | hypothetical |
| 0.264107 | 0.449719 | 0.640976 | pred_wait_1_detect_1_form2_stage_1_cancers_1_posrisk_2_negrisk_5 | GPs | hypothetical |
| 0.294761 | 0.491222 | 0.689509 | pred_wait_1_detect_1_form2_stage_1_cancers_5_posrisk_2_negrisk_5 | GPs | hypothetical |
| 0.339423 | 0.547987 | 0.749008 | pred_wait_1_detect_1_form2_stage_1_cancers_10_posrisk_2_negrisk_5 | GPs | hypothetical |
| 0.519522 | 0.721161 | 0.884091 | pred_wait_1_detect_1_form2_stage_1_cancers_25_posrisk_2_negrisk_5 | GPs | hypothetical |
| 0.216659 | 0.397211 | 0.589637 | pred_wait_1_detect_1_form2_stage_1_cancers_1_posrisk_2_negrisk_10 | GPs | hypothetical |
| 0.243033 | 0.436297 | 0.63862 | pred_wait_1_detect_1_form2_stage_1_cancers_5_posrisk_2_negrisk_10 | GPs | hypothetical |
| 0.282856 | 0.491391 | 0.701892 | pred_wait_1_detect_1_form2_stage_1_cancers_10_posrisk_2_negrisk_10 | GPs | hypothetical |
| 0.45484 | 0.672423 | 0.856132 | pred_wait_1_detect_1_form2_stage_1_cancers_25_posrisk_2_negrisk_10 | GPs | hypothetical |
| 0.056062 | 0.156536 | 0.284003 | pred_wait_1_detect_1_form2_stage_1_cancers_1_posrisk_2_negrisk_40 | GPs | hypothetical |
| 0.063175 | 0.172773 | 0.312522 | pred_wait_1_detect_1_form2_stage_1_cancers_5_posrisk_2_negrisk_40 | GPs | hypothetical |
| 0.075459 | 0.198764 | 0.355387 | pred_wait_1_detect_1_form2_stage_1_cancers_10_posrisk_2_negrisk_40 | GPs | hypothetical |
| 0.148482 | 0.323884 | 0.528296 | pred_wait_1_detect_1_form2_stage_1_cancers_25_posrisk_2_negrisk_40 | GPs | hypothetical |
| 0.109547 | 0.240304 | 0.394291 | pred_wait_1_detect_1_form2_stage_1_cancers_1_posrisk_4_negrisk_1 | GPs | hypothetical |
| 0.122251 | 0.26442 | 0.429584 | pred_wait_1_detect_1_form2_stage_1_cancers_5_posrisk_4_negrisk_1 | GPs | hypothetical |
| 0.142905 | 0.300534 | 0.480386 | pred_wait_1_detect_1_form2_stage_1_cancers_10_posrisk_4_negrisk_1 | GPs | hypothetical |
| 0.250066 | 0.446788 | 0.651691 | pred_wait_1_detect_1_form2_stage_1_cancers_25_posrisk_4_negrisk_1 | GPs | hypothetical |
| 0.087428 | 0.207584 | 0.353139 | pred_wait_1_detect_1_form2_stage_1_cancers_1_posrisk_4_negrisk_5 | GPs | hypothetical |
| 0.097824 | 0.228828 | 0.385715 | pred_wait_1_detect_1_form2_stage_1_cancers_5_posrisk_4_negrisk_5 | GPs | hypothetical |
| 0.114609 | 0.261381 | 0.434085 | pred_wait_1_detect_1_form2_stage_1_cancers_10_posrisk_4_negrisk_5 | GPs | hypothetical |
| 0.209791 | 0.401397 | 0.609054 | pred_wait_1_detect_1_form2_stage_1_cancers_25_posrisk_4_negrisk_5 | GPs | hypothetical |
| 0.066127 | 0.170744 | 0.304518 | pred_wait_1_detect_1_form2_stage_1_cancers_1_posrisk_4_negrisk_10 | GPs | hypothetical |
| 0.073137 | 0.188371 | 0.332958 | pred_wait_1_detect_1_form2_stage_1_cancers_5_posrisk_4_negrisk_10 | GPs | hypothetical |
| 0.086993 | 0.216151 | 0.376289 | pred_wait_1_detect_1_form2_stage_1_cancers_10_posrisk_4_negrisk_10 | GPs | hypothetical |
| 0.16464 | 0.345036 | 0.550102 | pred_wait_1_detect_1_form2_stage_1_cancers_25_posrisk_4_negrisk_10 | GPs | hypothetical |
| 0.011934 | 0.052231 | 0.115253 | pred_wait_1_detect_1_form2_stage_1_cancers_1_posrisk_4_negrisk_40 | GPs | hypothetical |
| 0.013272 | 0.056813 | 0.124409 | pred_wait_1_detect_1_form2_stage_1_cancers_5_posrisk_4_negrisk_40 | GPs | hypothetical |
| 0.015915 | 0.064978 | 0.140378 | pred_wait_1_detect_1_form2_stage_1_cancers_10_posrisk_4_negrisk_40 | GPs | hypothetical |
| 0.036407 | 0.11642 | 0.231362 | pred_wait_1_detect_1_form2_stage_1_cancers_25_posrisk_4_negrisk_40 | GPs | hypothetical |
| 0.04662 | 0.120473 | 0.217205 | pred_wait_1_detect_1_form2_stage_1_cancers_1_posrisk_6_negrisk_1 | GPs | hypothetical |
| 0.052975 | 0.131826 | 0.233437 | pred_wait_1_detect_1_form2_stage_1_cancers_5_posrisk_6_negrisk_1 | GPs | hypothetical |
| 0.061284 | 0.14935 | 0.259542 | pred_wait_1_detect_1_form2_stage_1_cancers_10_posrisk_6_negrisk_1 | GPs | hypothetical |
| 0.109005 | 0.22944 | 0.369791 | pred_wait_1_detect_1_form2_stage_1_cancers_25_posrisk_6_negrisk_1 | GPs | hypothetical |
| 0.036111 | 0.102505 | 0.191399 | pred_wait_1_detect_1_form2_stage_1_cancers_1_posrisk_6_negrisk_5 | GPs | hypothetical |
| 0.041064 | 0.112244 | 0.20671 | pred_wait_1_detect_1_form2_stage_1_cancers_5_posrisk_6_negrisk_5 | GPs | hypothetical |
| 0.04866 | 0.127537 | 0.22989 | pred_wait_1_detect_1_form2_stage_1_cancers_10_posrisk_6_negrisk_5 | GPs | hypothetical |
| 0.088753 | 0.200367 | 0.333966 | pred_wait_1_detect_1_form2_stage_1_cancers_25_posrisk_6_negrisk_5 | GPs | hypothetical |
| 0.026075 | 0.083252 | 0.161633 | pred_wait_1_detect_1_form2_stage_1_cancers_1_posrisk_6_negrisk_10 | GPs | hypothetical |
| 0.029829 | 0.091163 | 0.176037 | pred_wait_1_detect_1_form2_stage_1_cancers_5_posrisk_6_negrisk_10 | GPs | hypothetical |
| 0.035325 | 0.103858 | 0.195946 | pred_wait_1_detect_1_form2_stage_1_cancers_10_posrisk_6_negrisk_10 | GPs | hypothetical |
| 0.068687 | 0.167477 | 0.28956 | pred_wait_1_detect_1_form2_stage_1_cancers_25_posrisk_6_negrisk_10 | GPs | hypothetical |
| 0.004109 | 0.026114 | 0.064679 | pred_wait_1_detect_1_form2_stage_1_cancers_1_posrisk_6_negrisk_40 | GPs | hypothetical |
| 0.004675 | 0.02827 | 0.068103 | pred_wait_1_detect_1_form2_stage_1_cancers_5_posrisk_6_negrisk_40 | GPs | hypothetical |
| 0.005886 | 0.032088 | 0.07588 | pred_wait_1_detect_1_form2_stage_1_cancers_10_posrisk_6_negrisk_40 | GPs | hypothetical |
| 0.013687 | 0.055808 | 0.118819 | pred_wait_1_detect_1_form2_stage_1_cancers_25_posrisk_6_negrisk_40 | GPs | hypothetical |
| 0.026531 | 0.077031 | 0.14247 | pred_wait_1_detect_1_form2_stage_1_cancers_1_posrisk_8_negrisk_1 | GPs | hypothetical |
| 0.030154 | 0.083752 | 0.151369 | pred_wait_1_detect_1_form2_stage_1_cancers_5_posrisk_8_negrisk_1 | GPs | hypothetical |
| 0.035947 | 0.093946 | 0.167053 | pred_wait_1_detect_1_form2_stage_1_cancers_10_posrisk_8_negrisk_1 | GPs | hypothetical |
| 0.062542 | 0.139674 | 0.230739 | pred_wait_1_detect_1_form2_stage_1_cancers_25_posrisk_8_negrisk_1 | GPs | hypothetical |
| 0.020422 | 0.066498 | 0.126566 | pred_wait_1_detect_1_form2_stage_1_cancers_1_posrisk_8_negrisk_5 | GPs | hypothetical |
| 0.023755 | 0.072357 | 0.134879 | pred_wait_1_detect_1_form2_stage_1_cancers_5_posrisk_8_negrisk_5 | GPs | hypothetical |
| 0.02876 | 0.081356 | 0.148523 | pred_wait_1_detect_1_form2_stage_1_cancers_10_posrisk_8_negrisk_5 | GPs | hypothetical |
| 0.052063 | 0.122804 | 0.20864 | pred_wait_1_detect_1_form2_stage_1_cancers_25_posrisk_8_negrisk_5 | GPs | hypothetical |
| 0.014617 | 0.055196 | 0.108952 | pred_wait_1_detect_1_form2_stage_1_cancers_1_posrisk_8_negrisk_10 | GPs | hypothetical |
| 0.016834 | 0.060081 | 0.117257 | pred_wait_1_detect_1_form2_stage_1_cancers_5_posrisk_8_negrisk_10 | GPs | hypothetical |
| 0.020966 | 0.067707 | 0.129194 | pred_wait_1_detect_1_form2_stage_1_cancers_10_posrisk_8_negrisk_10 | GPs | hypothetical |
| 0.040592 | 0.104061 | 0.182106 | pred_wait_1_detect_1_form2_stage_1_cancers_25_posrisk_8_negrisk_10 | GPs | hypothetical |
| 0.002123 | 0.01975 | 0.050845 | pred_wait_1_detect_1_form2_stage_1_cancers_1_posrisk_8_negrisk_40 | GPs | hypothetical |
| 0.002481 | 0.021388 | 0.053261 | pred_wait_1_detect_1_form2_stage_1_cancers_5_posrisk_8_negrisk_40 | GPs | hypothetical |
| 0.003279 | 0.024122 | 0.058531 | pred_wait_1_detect_1_form2_stage_1_cancers_10_posrisk_8_negrisk_40 | GPs | hypothetical |
| 0.008088 | 0.039314 | 0.083522 | pred_wait_1_detect_1_form2_stage_1_cancers_25_posrisk_8_negrisk_40 | GPs | hypothetical |
| 0.303987 | 0.49117 | 0.680335 | pred_wait_1_detect_1_form2_stage_2_cancers_1_posrisk_2_negrisk_1 | GPs | hypothetical |
| 0.337389 | 0.533724 | 0.725643 | pred_wait_1_detect_1_form2_stage_2_cancers_5_posrisk_2_negrisk_1 | GPs | hypothetical |
| 0.386562 | 0.590536 | 0.781397 | pred_wait_1_detect_1_form2_stage_2_cancers_10_posrisk_2_negrisk_1 | GPs | hypothetical |
| 0.56791 | 0.754595 | 0.901387 | pred_wait_1_detect_1_form2_stage_2_cancers_25_posrisk_2_negrisk_1 | GPs | hypothetical |
| 0.264107 | 0.449719 | 0.640976 | pred_wait_1_detect_1_form2_stage_2_cancers_1_posrisk_2_negrisk_5 | GPs | hypothetical |
| 0.294761 | 0.491222 | 0.689509 | pred_wait_1_detect_1_form2_stage_2_cancers_5_posrisk_2_negrisk_5 | GPs | hypothetical |
| 0.339423 | 0.547987 | 0.749008 | pred_wait_1_detect_1_form2_stage_2_cancers_10_posrisk_2_negrisk_5 | GPs | hypothetical |
| 0.519522 | 0.721161 | 0.884091 | pred_wait_1_detect_1_form2_stage_2_cancers_25_posrisk_2_negrisk_5 | GPs | hypothetical |
| 0.216659 | 0.397211 | 0.589637 | pred_wait_1_detect_1_form2_stage_2_cancers_1_posrisk_2_negrisk_10 | GPs | hypothetical |
| 0.243033 | 0.436297 | 0.63862 | pred_wait_1_detect_1_form2_stage_2_cancers_5_posrisk_2_negrisk_10 | GPs | hypothetical |
| 0.282856 | 0.491391 | 0.701892 | pred_wait_1_detect_1_form2_stage_2_cancers_10_posrisk_2_negrisk_10 | GPs | hypothetical |
| 0.45484 | 0.672423 | 0.856132 | pred_wait_1_detect_1_form2_stage_2_cancers_25_posrisk_2_negrisk_10 | GPs | hypothetical |
| 0.056062 | 0.156536 | 0.284003 | pred_wait_1_detect_1_form2_stage_2_cancers_1_posrisk_2_negrisk_40 | GPs | hypothetical |
| 0.063175 | 0.172773 | 0.312522 | pred_wait_1_detect_1_form2_stage_2_cancers_5_posrisk_2_negrisk_40 | GPs | hypothetical |
| 0.075459 | 0.198764 | 0.355387 | pred_wait_1_detect_1_form2_stage_2_cancers_10_posrisk_2_negrisk_40 | GPs | hypothetical |
| 0.148482 | 0.323884 | 0.528296 | pred_wait_1_detect_1_form2_stage_2_cancers_25_posrisk_2_negrisk_40 | GPs | hypothetical |
| 0.109547 | 0.240304 | 0.394291 | pred_wait_1_detect_1_form2_stage_2_cancers_1_posrisk_4_negrisk_1 | GPs | hypothetical |
| 0.122251 | 0.26442 | 0.429584 | pred_wait_1_detect_1_form2_stage_2_cancers_5_posrisk_4_negrisk_1 | GPs | hypothetical |
| 0.142905 | 0.300534 | 0.480386 | pred_wait_1_detect_1_form2_stage_2_cancers_10_posrisk_4_negrisk_1 | GPs | hypothetical |
| 0.250066 | 0.446788 | 0.651691 | pred_wait_1_detect_1_form2_stage_2_cancers_25_posrisk_4_negrisk_1 | GPs | hypothetical |
| 0.087428 | 0.207584 | 0.353139 | pred_wait_1_detect_1_form2_stage_2_cancers_1_posrisk_4_negrisk_5 | GPs | hypothetical |
| 0.097824 | 0.228828 | 0.385715 | pred_wait_1_detect_1_form2_stage_2_cancers_5_posrisk_4_negrisk_5 | GPs | hypothetical |
| 0.114609 | 0.261381 | 0.434085 | pred_wait_1_detect_1_form2_stage_2_cancers_10_posrisk_4_negrisk_5 | GPs | hypothetical |
| 0.209791 | 0.401397 | 0.609054 | pred_wait_1_detect_1_form2_stage_2_cancers_25_posrisk_4_negrisk_5 | GPs | hypothetical |
| 0.066127 | 0.170744 | 0.304518 | pred_wait_1_detect_1_form2_stage_2_cancers_1_posrisk_4_negrisk_10 | GPs | hypothetical |
| 0.073137 | 0.188371 | 0.332958 | pred_wait_1_detect_1_form2_stage_2_cancers_5_posrisk_4_negrisk_10 | GPs | hypothetical |
| 0.086993 | 0.216151 | 0.376289 | pred_wait_1_detect_1_form2_stage_2_cancers_10_posrisk_4_negrisk_10 | GPs | hypothetical |
| 0.16464 | 0.345036 | 0.550102 | pred_wait_1_detect_1_form2_stage_2_cancers_25_posrisk_4_negrisk_10 | GPs | hypothetical |
| 0.011934 | 0.052231 | 0.115253 | pred_wait_1_detect_1_form2_stage_2_cancers_1_posrisk_4_negrisk_40 | GPs | hypothetical |
| 0.013272 | 0.056813 | 0.124409 | pred_wait_1_detect_1_form2_stage_2_cancers_5_posrisk_4_negrisk_40 | GPs | hypothetical |
| 0.015915 | 0.064978 | 0.140378 | pred_wait_1_detect_1_form2_stage_2_cancers_10_posrisk_4_negrisk_40 | GPs | hypothetical |
| 0.036407 | 0.11642 | 0.231362 | pred_wait_1_detect_1_form2_stage_2_cancers_25_posrisk_4_negrisk_40 | GPs | hypothetical |
| 0.04662 | 0.120473 | 0.217205 | pred_wait_1_detect_1_form2_stage_2_cancers_1_posrisk_6_negrisk_1 | GPs | hypothetical |
| 0.052975 | 0.131826 | 0.233437 | pred_wait_1_detect_1_form2_stage_2_cancers_5_posrisk_6_negrisk_1 | GPs | hypothetical |
| 0.061284 | 0.14935 | 0.259542 | pred_wait_1_detect_1_form2_stage_2_cancers_10_posrisk_6_negrisk_1 | GPs | hypothetical |
| 0.109005 | 0.22944 | 0.369791 | pred_wait_1_detect_1_form2_stage_2_cancers_25_posrisk_6_negrisk_1 | GPs | hypothetical |
| 0.036111 | 0.102505 | 0.191399 | pred_wait_1_detect_1_form2_stage_2_cancers_1_posrisk_6_negrisk_5 | GPs | hypothetical |
| 0.041064 | 0.112244 | 0.20671 | pred_wait_1_detect_1_form2_stage_2_cancers_5_posrisk_6_negrisk_5 | GPs | hypothetical |
| 0.04866 | 0.127537 | 0.22989 | pred_wait_1_detect_1_form2_stage_2_cancers_10_posrisk_6_negrisk_5 | GPs | hypothetical |
| 0.088753 | 0.200367 | 0.333966 | pred_wait_1_detect_1_form2_stage_2_cancers_25_posrisk_6_negrisk_5 | GPs | hypothetical |
| 0.026075 | 0.083252 | 0.161633 | pred_wait_1_detect_1_form2_stage_2_cancers_1_posrisk_6_negrisk_10 | GPs | hypothetical |
| 0.029829 | 0.091163 | 0.176037 | pred_wait_1_detect_1_form2_stage_2_cancers_5_posrisk_6_negrisk_10 | GPs | hypothetical |
| 0.035325 | 0.103858 | 0.195946 | pred_wait_1_detect_1_form2_stage_2_cancers_10_posrisk_6_negrisk_10 | GPs | hypothetical |
| 0.068687 | 0.167477 | 0.28956 | pred_wait_1_detect_1_form2_stage_2_cancers_25_posrisk_6_negrisk_10 | GPs | hypothetical |
| 0.004109 | 0.026114 | 0.064679 | pred_wait_1_detect_1_form2_stage_2_cancers_1_posrisk_6_negrisk_40 | GPs | hypothetical |
| 0.004675 | 0.02827 | 0.068103 | pred_wait_1_detect_1_form2_stage_2_cancers_5_posrisk_6_negrisk_40 | GPs | hypothetical |
| 0.005886 | 0.032088 | 0.07588 | pred_wait_1_detect_1_form2_stage_2_cancers_10_posrisk_6_negrisk_40 | GPs | hypothetical |
| 0.013687 | 0.055808 | 0.118819 | pred_wait_1_detect_1_form2_stage_2_cancers_25_posrisk_6_negrisk_40 | GPs | hypothetical |
| 0.026531 | 0.077031 | 0.14247 | pred_wait_1_detect_1_form2_stage_2_cancers_1_posrisk_8_negrisk_1 | GPs | hypothetical |
| 0.030154 | 0.083752 | 0.151369 | pred_wait_1_detect_1_form2_stage_2_cancers_5_posrisk_8_negrisk_1 | GPs | hypothetical |
| 0.035947 | 0.093946 | 0.167053 | pred_wait_1_detect_1_form2_stage_2_cancers_10_posrisk_8_negrisk_1 | GPs | hypothetical |
| 0.062542 | 0.139674 | 0.230739 | pred_wait_1_detect_1_form2_stage_2_cancers_25_posrisk_8_negrisk_1 | GPs | hypothetical |
| 0.020422 | 0.066498 | 0.126566 | pred_wait_1_detect_1_form2_stage_2_cancers_1_posrisk_8_negrisk_5 | GPs | hypothetical |
| 0.023755 | 0.072357 | 0.134879 | pred_wait_1_detect_1_form2_stage_2_cancers_5_posrisk_8_negrisk_5 | GPs | hypothetical |
| 0.02876 | 0.081356 | 0.148523 | pred_wait_1_detect_1_form2_stage_2_cancers_10_posrisk_8_negrisk_5 | GPs | hypothetical |
| 0.052063 | 0.122804 | 0.20864 | pred_wait_1_detect_1_form2_stage_2_cancers_25_posrisk_8_negrisk_5 | GPs | hypothetical |
| 0.014617 | 0.055196 | 0.108952 | pred_wait_1_detect_1_form2_stage_2_cancers_1_posrisk_8_negrisk_10 | GPs | hypothetical |
| 0.016834 | 0.060081 | 0.117257 | pred_wait_1_detect_1_form2_stage_2_cancers_5_posrisk_8_negrisk_10 | GPs | hypothetical |
| 0.020966 | 0.067707 | 0.129194 | pred_wait_1_detect_1_form2_stage_2_cancers_10_posrisk_8_negrisk_10 | GPs | hypothetical |
| 0.040592 | 0.104061 | 0.182106 | pred_wait_1_detect_1_form2_stage_2_cancers_25_posrisk_8_negrisk_10 | GPs | hypothetical |
| 0.002123 | 0.01975 | 0.050845 | pred_wait_1_detect_1_form2_stage_2_cancers_1_posrisk_8_negrisk_40 | GPs | hypothetical |
| 0.002481 | 0.021388 | 0.053261 | pred_wait_1_detect_1_form2_stage_2_cancers_5_posrisk_8_negrisk_40 | GPs | hypothetical |
| 0.003279 | 0.024122 | 0.058531 | pred_wait_1_detect_1_form2_stage_2_cancers_10_posrisk_8_negrisk_40 | GPs | hypothetical |
| 0.008088 | 0.039314 | 0.083522 | pred_wait_1_detect_1_form2_stage_2_cancers_25_posrisk_8_negrisk_40 | GPs | hypothetical |
| 0.303987 | 0.49117 | 0.680335 | pred_wait_1_detect_1_form3_stage_1_cancers_1_posrisk_2_negrisk_1 | GPs | hypothetical |
| 0.337389 | 0.533724 | 0.725643 | pred_wait_1_detect_1_form3_stage_1_cancers_5_posrisk_2_negrisk_1 | GPs | hypothetical |
| 0.386562 | 0.590536 | 0.781397 | pred_wait_1_detect_1_form3_stage_1_cancers_10_posrisk_2_negrisk_1 | GPs | hypothetical |
| 0.56791 | 0.754595 | 0.901387 | pred_wait_1_detect_1_form3_stage_1_cancers_25_posrisk_2_negrisk_1 | GPs | hypothetical |
| 0.264107 | 0.449719 | 0.640976 | pred_wait_1_detect_1_form3_stage_1_cancers_1_posrisk_2_negrisk_5 | GPs | hypothetical |
| 0.294761 | 0.491222 | 0.689509 | pred_wait_1_detect_1_form3_stage_1_cancers_5_posrisk_2_negrisk_5 | GPs | hypothetical |
| 0.339423 | 0.547987 | 0.749008 | pred_wait_1_detect_1_form3_stage_1_cancers_10_posrisk_2_negrisk_5 | GPs | hypothetical |
| 0.519522 | 0.721161 | 0.884091 | pred_wait_1_detect_1_form3_stage_1_cancers_25_posrisk_2_negrisk_5 | GPs | hypothetical |
| 0.216659 | 0.397211 | 0.589637 | pred_wait_1_detect_1_form3_stage_1_cancers_1_posrisk_2_negrisk_10 | GPs | hypothetical |
| 0.243033 | 0.436297 | 0.63862 | pred_wait_1_detect_1_form3_stage_1_cancers_5_posrisk_2_negrisk_10 | GPs | hypothetical |
| 0.282856 | 0.491391 | 0.701892 | pred_wait_1_detect_1_form3_stage_1_cancers_10_posrisk_2_negrisk_10 | GPs | hypothetical |
| 0.45484 | 0.672423 | 0.856132 | pred_wait_1_detect_1_form3_stage_1_cancers_25_posrisk_2_negrisk_10 | GPs | hypothetical |
| 0.056062 | 0.156536 | 0.284003 | pred_wait_1_detect_1_form3_stage_1_cancers_1_posrisk_2_negrisk_40 | GPs | hypothetical |
| 0.063175 | 0.172773 | 0.312522 | pred_wait_1_detect_1_form3_stage_1_cancers_5_posrisk_2_negrisk_40 | GPs | hypothetical |
| 0.075459 | 0.198764 | 0.355387 | pred_wait_1_detect_1_form3_stage_1_cancers_10_posrisk_2_negrisk_40 | GPs | hypothetical |
| 0.148482 | 0.323884 | 0.528296 | pred_wait_1_detect_1_form3_stage_1_cancers_25_posrisk_2_negrisk_40 | GPs | hypothetical |
| 0.109547 | 0.240304 | 0.394291 | pred_wait_1_detect_1_form3_stage_1_cancers_1_posrisk_4_negrisk_1 | GPs | hypothetical |
| 0.122251 | 0.26442 | 0.429584 | pred_wait_1_detect_1_form3_stage_1_cancers_5_posrisk_4_negrisk_1 | GPs | hypothetical |
| 0.142905 | 0.300534 | 0.480386 | pred_wait_1_detect_1_form3_stage_1_cancers_10_posrisk_4_negrisk_1 | GPs | hypothetical |
| 0.250066 | 0.446788 | 0.651691 | pred_wait_1_detect_1_form3_stage_1_cancers_25_posrisk_4_negrisk_1 | GPs | hypothetical |
| 0.087428 | 0.207584 | 0.353139 | pred_wait_1_detect_1_form3_stage_1_cancers_1_posrisk_4_negrisk_5 | GPs | hypothetical |
| 0.097824 | 0.228828 | 0.385715 | pred_wait_1_detect_1_form3_stage_1_cancers_5_posrisk_4_negrisk_5 | GPs | hypothetical |
| 0.114609 | 0.261381 | 0.434085 | pred_wait_1_detect_1_form3_stage_1_cancers_10_posrisk_4_negrisk_5 | GPs | hypothetical |
| 0.209791 | 0.401397 | 0.609054 | pred_wait_1_detect_1_form3_stage_1_cancers_25_posrisk_4_negrisk_5 | GPs | hypothetical |
| 0.066127 | 0.170744 | 0.304518 | pred_wait_1_detect_1_form3_stage_1_cancers_1_posrisk_4_negrisk_10 | GPs | hypothetical |
| 0.073137 | 0.188371 | 0.332958 | pred_wait_1_detect_1_form3_stage_1_cancers_5_posrisk_4_negrisk_10 | GPs | hypothetical |
| 0.086993 | 0.216151 | 0.376289 | pred_wait_1_detect_1_form3_stage_1_cancers_10_posrisk_4_negrisk_10 | GPs | hypothetical |
| 0.16464 | 0.345036 | 0.550102 | pred_wait_1_detect_1_form3_stage_1_cancers_25_posrisk_4_negrisk_10 | GPs | hypothetical |
| 0.011934 | 0.052231 | 0.115253 | pred_wait_1_detect_1_form3_stage_1_cancers_1_posrisk_4_negrisk_40 | GPs | hypothetical |
| 0.013272 | 0.056813 | 0.124409 | pred_wait_1_detect_1_form3_stage_1_cancers_5_posrisk_4_negrisk_40 | GPs | hypothetical |
| 0.015915 | 0.064978 | 0.140378 | pred_wait_1_detect_1_form3_stage_1_cancers_10_posrisk_4_negrisk_40 | GPs | hypothetical |
| 0.036407 | 0.11642 | 0.231362 | pred_wait_1_detect_1_form3_stage_1_cancers_25_posrisk_4_negrisk_40 | GPs | hypothetical |
| 0.04662 | 0.120473 | 0.217205 | pred_wait_1_detect_1_form3_stage_1_cancers_1_posrisk_6_negrisk_1 | GPs | hypothetical |
| 0.052975 | 0.131826 | 0.233437 | pred_wait_1_detect_1_form3_stage_1_cancers_5_posrisk_6_negrisk_1 | GPs | hypothetical |
| 0.061284 | 0.14935 | 0.259542 | pred_wait_1_detect_1_form3_stage_1_cancers_10_posrisk_6_negrisk_1 | GPs | hypothetical |
| 0.109005 | 0.22944 | 0.369791 | pred_wait_1_detect_1_form3_stage_1_cancers_25_posrisk_6_negrisk_1 | GPs | hypothetical |
| 0.036111 | 0.102505 | 0.191399 | pred_wait_1_detect_1_form3_stage_1_cancers_1_posrisk_6_negrisk_5 | GPs | hypothetical |
| 0.041064 | 0.112244 | 0.20671 | pred_wait_1_detect_1_form3_stage_1_cancers_5_posrisk_6_negrisk_5 | GPs | hypothetical |
| 0.04866 | 0.127537 | 0.22989 | pred_wait_1_detect_1_form3_stage_1_cancers_10_posrisk_6_negrisk_5 | GPs | hypothetical |
| 0.088753 | 0.200367 | 0.333966 | pred_wait_1_detect_1_form3_stage_1_cancers_25_posrisk_6_negrisk_5 | GPs | hypothetical |
| 0.026075 | 0.083252 | 0.161633 | pred_wait_1_detect_1_form3_stage_1_cancers_1_posrisk_6_negrisk_10 | GPs | hypothetical |
| 0.029829 | 0.091163 | 0.176037 | pred_wait_1_detect_1_form3_stage_1_cancers_5_posrisk_6_negrisk_10 | GPs | hypothetical |
| 0.035325 | 0.103858 | 0.195946 | pred_wait_1_detect_1_form3_stage_1_cancers_10_posrisk_6_negrisk_10 | GPs | hypothetical |
| 0.068687 | 0.167477 | 0.28956 | pred_wait_1_detect_1_form3_stage_1_cancers_25_posrisk_6_negrisk_10 | GPs | hypothetical |
| 0.004109 | 0.026114 | 0.064679 | pred_wait_1_detect_1_form3_stage_1_cancers_1_posrisk_6_negrisk_40 | GPs | hypothetical |
| 0.004675 | 0.02827 | 0.068103 | pred_wait_1_detect_1_form3_stage_1_cancers_5_posrisk_6_negrisk_40 | GPs | hypothetical |
| 0.005886 | 0.032088 | 0.07588 | pred_wait_1_detect_1_form3_stage_1_cancers_10_posrisk_6_negrisk_40 | GPs | hypothetical |
| 0.013687 | 0.055808 | 0.118819 | pred_wait_1_detect_1_form3_stage_1_cancers_25_posrisk_6_negrisk_40 | GPs | hypothetical |
| 0.026531 | 0.077031 | 0.14247 | pred_wait_1_detect_1_form3_stage_1_cancers_1_posrisk_8_negrisk_1 | GPs | hypothetical |
| 0.030154 | 0.083752 | 0.151369 | pred_wait_1_detect_1_form3_stage_1_cancers_5_posrisk_8_negrisk_1 | GPs | hypothetical |
| 0.035947 | 0.093946 | 0.167053 | pred_wait_1_detect_1_form3_stage_1_cancers_10_posrisk_8_negrisk_1 | GPs | hypothetical |
| 0.062542 | 0.139674 | 0.230739 | pred_wait_1_detect_1_form3_stage_1_cancers_25_posrisk_8_negrisk_1 | GPs | hypothetical |
| 0.020422 | 0.066498 | 0.126566 | pred_wait_1_detect_1_form3_stage_1_cancers_1_posrisk_8_negrisk_5 | GPs | hypothetical |
| 0.023755 | 0.072357 | 0.134879 | pred_wait_1_detect_1_form3_stage_1_cancers_5_posrisk_8_negrisk_5 | GPs | hypothetical |
| 0.02876 | 0.081356 | 0.148523 | pred_wait_1_detect_1_form3_stage_1_cancers_10_posrisk_8_negrisk_5 | GPs | hypothetical |
| 0.052063 | 0.122804 | 0.20864 | pred_wait_1_detect_1_form3_stage_1_cancers_25_posrisk_8_negrisk_5 | GPs | hypothetical |
| 0.014617 | 0.055196 | 0.108952 | pred_wait_1_detect_1_form3_stage_1_cancers_1_posrisk_8_negrisk_10 | GPs | hypothetical |
| 0.016834 | 0.060081 | 0.117257 | pred_wait_1_detect_1_form3_stage_1_cancers_5_posrisk_8_negrisk_10 | GPs | hypothetical |
| 0.020966 | 0.067707 | 0.129194 | pred_wait_1_detect_1_form3_stage_1_cancers_10_posrisk_8_negrisk_10 | GPs | hypothetical |
| 0.040592 | 0.104061 | 0.182106 | pred_wait_1_detect_1_form3_stage_1_cancers_25_posrisk_8_negrisk_10 | GPs | hypothetical |
| 0.002123 | 0.01975 | 0.050845 | pred_wait_1_detect_1_form3_stage_1_cancers_1_posrisk_8_negrisk_40 | GPs | hypothetical |
| 0.002481 | 0.021388 | 0.053261 | pred_wait_1_detect_1_form3_stage_1_cancers_5_posrisk_8_negrisk_40 | GPs | hypothetical |
| 0.003279 | 0.024122 | 0.058531 | pred_wait_1_detect_1_form3_stage_1_cancers_10_posrisk_8_negrisk_40 | GPs | hypothetical |
| 0.008088 | 0.039314 | 0.083522 | pred_wait_1_detect_1_form3_stage_1_cancers_25_posrisk_8_negrisk_40 | GPs | hypothetical |
| 0.303987 | 0.49117 | 0.680335 | pred_wait_1_detect_1_form3_stage_2_cancers_1_posrisk_2_negrisk_1 | GPs | hypothetical |
| 0.337389 | 0.533724 | 0.725643 | pred_wait_1_detect_1_form3_stage_2_cancers_5_posrisk_2_negrisk_1 | GPs | hypothetical |
| 0.386562 | 0.590536 | 0.781397 | pred_wait_1_detect_1_form3_stage_2_cancers_10_posrisk_2_negrisk_1 | GPs | hypothetical |
| 0.56791 | 0.754595 | 0.901387 | pred_wait_1_detect_1_form3_stage_2_cancers_25_posrisk_2_negrisk_1 | GPs | hypothetical |
| 0.264107 | 0.449719 | 0.640976 | pred_wait_1_detect_1_form3_stage_2_cancers_1_posrisk_2_negrisk_5 | GPs | hypothetical |
| 0.294761 | 0.491222 | 0.689509 | pred_wait_1_detect_1_form3_stage_2_cancers_5_posrisk_2_negrisk_5 | GPs | hypothetical |
| 0.339423 | 0.547987 | 0.749008 | pred_wait_1_detect_1_form3_stage_2_cancers_10_posrisk_2_negrisk_5 | GPs | hypothetical |
| 0.519522 | 0.721161 | 0.884091 | pred_wait_1_detect_1_form3_stage_2_cancers_25_posrisk_2_negrisk_5 | GPs | hypothetical |
| 0.216659 | 0.397211 | 0.589637 | pred_wait_1_detect_1_form3_stage_2_cancers_1_posrisk_2_negrisk_10 | GPs | hypothetical |
| 0.243033 | 0.436297 | 0.63862 | pred_wait_1_detect_1_form3_stage_2_cancers_5_posrisk_2_negrisk_10 | GPs | hypothetical |
| 0.282856 | 0.491391 | 0.701892 | pred_wait_1_detect_1_form3_stage_2_cancers_10_posrisk_2_negrisk_10 | GPs | hypothetical |
| 0.45484 | 0.672423 | 0.856132 | pred_wait_1_detect_1_form3_stage_2_cancers_25_posrisk_2_negrisk_10 | GPs | hypothetical |
| 0.056062 | 0.156536 | 0.284003 | pred_wait_1_detect_1_form3_stage_2_cancers_1_posrisk_2_negrisk_40 | GPs | hypothetical |
| 0.063175 | 0.172773 | 0.312522 | pred_wait_1_detect_1_form3_stage_2_cancers_5_posrisk_2_negrisk_40 | GPs | hypothetical |
| 0.075459 | 0.198764 | 0.355387 | pred_wait_1_detect_1_form3_stage_2_cancers_10_posrisk_2_negrisk_40 | GPs | hypothetical |
| 0.148482 | 0.323884 | 0.528296 | pred_wait_1_detect_1_form3_stage_2_cancers_25_posrisk_2_negrisk_40 | GPs | hypothetical |
| 0.109547 | 0.240304 | 0.394291 | pred_wait_1_detect_1_form3_stage_2_cancers_1_posrisk_4_negrisk_1 | GPs | hypothetical |
| 0.122251 | 0.26442 | 0.429584 | pred_wait_1_detect_1_form3_stage_2_cancers_5_posrisk_4_negrisk_1 | GPs | hypothetical |
| 0.142905 | 0.300534 | 0.480386 | pred_wait_1_detect_1_form3_stage_2_cancers_10_posrisk_4_negrisk_1 | GPs | hypothetical |
| 0.250066 | 0.446788 | 0.651691 | pred_wait_1_detect_1_form3_stage_2_cancers_25_posrisk_4_negrisk_1 | GPs | hypothetical |
| 0.087428 | 0.207584 | 0.353139 | pred_wait_1_detect_1_form3_stage_2_cancers_1_posrisk_4_negrisk_5 | GPs | hypothetical |
| 0.097824 | 0.228828 | 0.385715 | pred_wait_1_detect_1_form3_stage_2_cancers_5_posrisk_4_negrisk_5 | GPs | hypothetical |
| 0.114609 | 0.261381 | 0.434085 | pred_wait_1_detect_1_form3_stage_2_cancers_10_posrisk_4_negrisk_5 | GPs | hypothetical |
| 0.209791 | 0.401397 | 0.609054 | pred_wait_1_detect_1_form3_stage_2_cancers_25_posrisk_4_negrisk_5 | GPs | hypothetical |
| 0.066127 | 0.170744 | 0.304518 | pred_wait_1_detect_1_form3_stage_2_cancers_1_posrisk_4_negrisk_10 | GPs | hypothetical |
| 0.073137 | 0.188371 | 0.332958 | pred_wait_1_detect_1_form3_stage_2_cancers_5_posrisk_4_negrisk_10 | GPs | hypothetical |
| 0.086993 | 0.216151 | 0.376289 | pred_wait_1_detect_1_form3_stage_2_cancers_10_posrisk_4_negrisk_10 | GPs | hypothetical |
| 0.16464 | 0.345036 | 0.550102 | pred_wait_1_detect_1_form3_stage_2_cancers_25_posrisk_4_negrisk_10 | GPs | hypothetical |
| 0.011934 | 0.052231 | 0.115253 | pred_wait_1_detect_1_form3_stage_2_cancers_1_posrisk_4_negrisk_40 | GPs | hypothetical |
| 0.013272 | 0.056813 | 0.124409 | pred_wait_1_detect_1_form3_stage_2_cancers_5_posrisk_4_negrisk_40 | GPs | hypothetical |
| 0.015915 | 0.064978 | 0.140378 | pred_wait_1_detect_1_form3_stage_2_cancers_10_posrisk_4_negrisk_40 | GPs | hypothetical |
| 0.036407 | 0.11642 | 0.231362 | pred_wait_1_detect_1_form3_stage_2_cancers_25_posrisk_4_negrisk_40 | GPs | hypothetical |
| 0.04662 | 0.120473 | 0.217205 | pred_wait_1_detect_1_form3_stage_2_cancers_1_posrisk_6_negrisk_1 | GPs | hypothetical |
| 0.052975 | 0.131826 | 0.233437 | pred_wait_1_detect_1_form3_stage_2_cancers_5_posrisk_6_negrisk_1 | GPs | hypothetical |
| 0.061284 | 0.14935 | 0.259542 | pred_wait_1_detect_1_form3_stage_2_cancers_10_posrisk_6_negrisk_1 | GPs | hypothetical |
| 0.109005 | 0.22944 | 0.369791 | pred_wait_1_detect_1_form3_stage_2_cancers_25_posrisk_6_negrisk_1 | GPs | hypothetical |
| 0.036111 | 0.102505 | 0.191399 | pred_wait_1_detect_1_form3_stage_2_cancers_1_posrisk_6_negrisk_5 | GPs | hypothetical |
| 0.041064 | 0.112244 | 0.20671 | pred_wait_1_detect_1_form3_stage_2_cancers_5_posrisk_6_negrisk_5 | GPs | hypothetical |
| 0.04866 | 0.127537 | 0.22989 | pred_wait_1_detect_1_form3_stage_2_cancers_10_posrisk_6_negrisk_5 | GPs | hypothetical |
| 0.088753 | 0.200367 | 0.333966 | pred_wait_1_detect_1_form3_stage_2_cancers_25_posrisk_6_negrisk_5 | GPs | hypothetical |
| 0.026075 | 0.083252 | 0.161633 | pred_wait_1_detect_1_form3_stage_2_cancers_1_posrisk_6_negrisk_10 | GPs | hypothetical |
| 0.029829 | 0.091163 | 0.176037 | pred_wait_1_detect_1_form3_stage_2_cancers_5_posrisk_6_negrisk_10 | GPs | hypothetical |
| 0.035325 | 0.103858 | 0.195946 | pred_wait_1_detect_1_form3_stage_2_cancers_10_posrisk_6_negrisk_10 | GPs | hypothetical |
| 0.068687 | 0.167477 | 0.28956 | pred_wait_1_detect_1_form3_stage_2_cancers_25_posrisk_6_negrisk_10 | GPs | hypothetical |
| 0.004109 | 0.026114 | 0.064679 | pred_wait_1_detect_1_form3_stage_2_cancers_1_posrisk_6_negrisk_40 | GPs | hypothetical |
| 0.004675 | 0.02827 | 0.068103 | pred_wait_1_detect_1_form3_stage_2_cancers_5_posrisk_6_negrisk_40 | GPs | hypothetical |
| 0.005886 | 0.032088 | 0.07588 | pred_wait_1_detect_1_form3_stage_2_cancers_10_posrisk_6_negrisk_40 | GPs | hypothetical |
| 0.013687 | 0.055808 | 0.118819 | pred_wait_1_detect_1_form3_stage_2_cancers_25_posrisk_6_negrisk_40 | GPs | hypothetical |
| 0.026531 | 0.077031 | 0.14247 | pred_wait_1_detect_1_form3_stage_2_cancers_1_posrisk_8_negrisk_1 | GPs | hypothetical |
| 0.030154 | 0.083752 | 0.151369 | pred_wait_1_detect_1_form3_stage_2_cancers_5_posrisk_8_negrisk_1 | GPs | hypothetical |
| 0.035947 | 0.093946 | 0.167053 | pred_wait_1_detect_1_form3_stage_2_cancers_10_posrisk_8_negrisk_1 | GPs | hypothetical |
| 0.062542 | 0.139674 | 0.230739 | pred_wait_1_detect_1_form3_stage_2_cancers_25_posrisk_8_negrisk_1 | GPs | hypothetical |
| 0.020422 | 0.066498 | 0.126566 | pred_wait_1_detect_1_form3_stage_2_cancers_1_posrisk_8_negrisk_5 | GPs | hypothetical |
| 0.023755 | 0.072357 | 0.134879 | pred_wait_1_detect_1_form3_stage_2_cancers_5_posrisk_8_negrisk_5 | GPs | hypothetical |
| 0.02876 | 0.081356 | 0.148523 | pred_wait_1_detect_1_form3_stage_2_cancers_10_posrisk_8_negrisk_5 | GPs | hypothetical |
| 0.052063 | 0.122804 | 0.20864 | pred_wait_1_detect_1_form3_stage_2_cancers_25_posrisk_8_negrisk_5 | GPs | hypothetical |
| 0.014617 | 0.055196 | 0.108952 | pred_wait_1_detect_1_form3_stage_2_cancers_1_posrisk_8_negrisk_10 | GPs | hypothetical |
| 0.016834 | 0.060081 | 0.117257 | pred_wait_1_detect_1_form3_stage_2_cancers_5_posrisk_8_negrisk_10 | GPs | hypothetical |
| 0.020966 | 0.067707 | 0.129194 | pred_wait_1_detect_1_form3_stage_2_cancers_10_posrisk_8_negrisk_10 | GPs | hypothetical |
| 0.040592 | 0.104061 | 0.182106 | pred_wait_1_detect_1_form3_stage_2_cancers_25_posrisk_8_negrisk_10 | GPs | hypothetical |
| 0.002123 | 0.01975 | 0.050845 | pred_wait_1_detect_1_form3_stage_2_cancers_1_posrisk_8_negrisk_40 | GPs | hypothetical |
| 0.002481 | 0.021388 | 0.053261 | pred_wait_1_detect_1_form3_stage_2_cancers_5_posrisk_8_negrisk_40 | GPs | hypothetical |
| 0.003279 | 0.024122 | 0.058531 | pred_wait_1_detect_1_form3_stage_2_cancers_10_posrisk_8_negrisk_40 | GPs | hypothetical |
| 0.008088 | 0.039314 | 0.083522 | pred_wait_1_detect_1_form3_stage_2_cancers_25_posrisk_8_negrisk_40 | GPs | hypothetical |
| 0.303987 | 0.49117 | 0.680335 | pred_wait_1_detect_1_form4_stage_1_cancers_1_posrisk_2_negrisk_1 | GPs | hypothetical |
| 0.337389 | 0.533724 | 0.725643 | pred_wait_1_detect_1_form4_stage_1_cancers_5_posrisk_2_negrisk_1 | GPs | hypothetical |
| 0.386562 | 0.590536 | 0.781397 | pred_wait_1_detect_1_form4_stage_1_cancers_10_posrisk_2_negrisk_1 | GPs | hypothetical |
| 0.56791 | 0.754595 | 0.901387 | pred_wait_1_detect_1_form4_stage_1_cancers_25_posrisk_2_negrisk_1 | GPs | hypothetical |
| 0.264107 | 0.449719 | 0.640976 | pred_wait_1_detect_1_form4_stage_1_cancers_1_posrisk_2_negrisk_5 | GPs | hypothetical |
| 0.294761 | 0.491222 | 0.689509 | pred_wait_1_detect_1_form4_stage_1_cancers_5_posrisk_2_negrisk_5 | GPs | hypothetical |
| 0.339423 | 0.547987 | 0.749008 | pred_wait_1_detect_1_form4_stage_1_cancers_10_posrisk_2_negrisk_5 | GPs | hypothetical |
| 0.519522 | 0.721161 | 0.884091 | pred_wait_1_detect_1_form4_stage_1_cancers_25_posrisk_2_negrisk_5 | GPs | hypothetical |
| 0.216659 | 0.397211 | 0.589637 | pred_wait_1_detect_1_form4_stage_1_cancers_1_posrisk_2_negrisk_10 | GPs | hypothetical |
| 0.243033 | 0.436297 | 0.63862 | pred_wait_1_detect_1_form4_stage_1_cancers_5_posrisk_2_negrisk_10 | GPs | hypothetical |
| 0.282856 | 0.491391 | 0.701892 | pred_wait_1_detect_1_form4_stage_1_cancers_10_posrisk_2_negrisk_10 | GPs | hypothetical |
| 0.45484 | 0.672423 | 0.856132 | pred_wait_1_detect_1_form4_stage_1_cancers_25_posrisk_2_negrisk_10 | GPs | hypothetical |
| 0.056062 | 0.156536 | 0.284003 | pred_wait_1_detect_1_form4_stage_1_cancers_1_posrisk_2_negrisk_40 | GPs | hypothetical |
| 0.063175 | 0.172773 | 0.312522 | pred_wait_1_detect_1_form4_stage_1_cancers_5_posrisk_2_negrisk_40 | GPs | hypothetical |
| 0.075459 | 0.198764 | 0.355387 | pred_wait_1_detect_1_form4_stage_1_cancers_10_posrisk_2_negrisk_40 | GPs | hypothetical |
| 0.148482 | 0.323884 | 0.528296 | pred_wait_1_detect_1_form4_stage_1_cancers_25_posrisk_2_negrisk_40 | GPs | hypothetical |
| 0.109547 | 0.240304 | 0.394291 | pred_wait_1_detect_1_form4_stage_1_cancers_1_posrisk_4_negrisk_1 | GPs | hypothetical |
| 0.122251 | 0.26442 | 0.429584 | pred_wait_1_detect_1_form4_stage_1_cancers_5_posrisk_4_negrisk_1 | GPs | hypothetical |
| 0.142905 | 0.300534 | 0.480386 | pred_wait_1_detect_1_form4_stage_1_cancers_10_posrisk_4_negrisk_1 | GPs | hypothetical |
| 0.250066 | 0.446788 | 0.651691 | pred_wait_1_detect_1_form4_stage_1_cancers_25_posrisk_4_negrisk_1 | GPs | hypothetical |
| 0.087428 | 0.207584 | 0.353139 | pred_wait_1_detect_1_form4_stage_1_cancers_1_posrisk_4_negrisk_5 | GPs | hypothetical |
| 0.097824 | 0.228828 | 0.385715 | pred_wait_1_detect_1_form4_stage_1_cancers_5_posrisk_4_negrisk_5 | GPs | hypothetical |
| 0.114609 | 0.261381 | 0.434085 | pred_wait_1_detect_1_form4_stage_1_cancers_10_posrisk_4_negrisk_5 | GPs | hypothetical |
| 0.209791 | 0.401397 | 0.609054 | pred_wait_1_detect_1_form4_stage_1_cancers_25_posrisk_4_negrisk_5 | GPs | hypothetical |
| 0.066127 | 0.170744 | 0.304518 | pred_wait_1_detect_1_form4_stage_1_cancers_1_posrisk_4_negrisk_10 | GPs | hypothetical |
| 0.073137 | 0.188371 | 0.332958 | pred_wait_1_detect_1_form4_stage_1_cancers_5_posrisk_4_negrisk_10 | GPs | hypothetical |
| 0.086993 | 0.216151 | 0.376289 | pred_wait_1_detect_1_form4_stage_1_cancers_10_posrisk_4_negrisk_10 | GPs | hypothetical |
| 0.16464 | 0.345036 | 0.550102 | pred_wait_1_detect_1_form4_stage_1_cancers_25_posrisk_4_negrisk_10 | GPs | hypothetical |
| 0.011934 | 0.052231 | 0.115253 | pred_wait_1_detect_1_form4_stage_1_cancers_1_posrisk_4_negrisk_40 | GPs | hypothetical |
| 0.013272 | 0.056813 | 0.124409 | pred_wait_1_detect_1_form4_stage_1_cancers_5_posrisk_4_negrisk_40 | GPs | hypothetical |
| 0.015915 | 0.064978 | 0.140378 | pred_wait_1_detect_1_form4_stage_1_cancers_10_posrisk_4_negrisk_40 | GPs | hypothetical |
| 0.036407 | 0.11642 | 0.231362 | pred_wait_1_detect_1_form4_stage_1_cancers_25_posrisk_4_negrisk_40 | GPs | hypothetical |
| 0.04662 | 0.120473 | 0.217205 | pred_wait_1_detect_1_form4_stage_1_cancers_1_posrisk_6_negrisk_1 | GPs | hypothetical |
| 0.052975 | 0.131826 | 0.233437 | pred_wait_1_detect_1_form4_stage_1_cancers_5_posrisk_6_negrisk_1 | GPs | hypothetical |
| 0.061284 | 0.14935 | 0.259542 | pred_wait_1_detect_1_form4_stage_1_cancers_10_posrisk_6_negrisk_1 | GPs | hypothetical |
| 0.109005 | 0.22944 | 0.369791 | pred_wait_1_detect_1_form4_stage_1_cancers_25_posrisk_6_negrisk_1 | GPs | hypothetical |
| 0.036111 | 0.102505 | 0.191399 | pred_wait_1_detect_1_form4_stage_1_cancers_1_posrisk_6_negrisk_5 | GPs | hypothetical |
| 0.041064 | 0.112244 | 0.20671 | pred_wait_1_detect_1_form4_stage_1_cancers_5_posrisk_6_negrisk_5 | GPs | hypothetical |
| 0.04866 | 0.127537 | 0.22989 | pred_wait_1_detect_1_form4_stage_1_cancers_10_posrisk_6_negrisk_5 | GPs | hypothetical |
| 0.088753 | 0.200367 | 0.333966 | pred_wait_1_detect_1_form4_stage_1_cancers_25_posrisk_6_negrisk_5 | GPs | hypothetical |
| 0.026075 | 0.083252 | 0.161633 | pred_wait_1_detect_1_form4_stage_1_cancers_1_posrisk_6_negrisk_10 | GPs | hypothetical |
| 0.029829 | 0.091163 | 0.176037 | pred_wait_1_detect_1_form4_stage_1_cancers_5_posrisk_6_negrisk_10 | GPs | hypothetical |
| 0.035325 | 0.103858 | 0.195946 | pred_wait_1_detect_1_form4_stage_1_cancers_10_posrisk_6_negrisk_10 | GPs | hypothetical |
| 0.068687 | 0.167477 | 0.28956 | pred_wait_1_detect_1_form4_stage_1_cancers_25_posrisk_6_negrisk_10 | GPs | hypothetical |
| 0.004109 | 0.026114 | 0.064679 | pred_wait_1_detect_1_form4_stage_1_cancers_1_posrisk_6_negrisk_40 | GPs | hypothetical |
| 0.004675 | 0.02827 | 0.068103 | pred_wait_1_detect_1_form4_stage_1_cancers_5_posrisk_6_negrisk_40 | GPs | hypothetical |
| 0.005886 | 0.032088 | 0.07588 | pred_wait_1_detect_1_form4_stage_1_cancers_10_posrisk_6_negrisk_40 | GPs | hypothetical |
| 0.013687 | 0.055808 | 0.118819 | pred_wait_1_detect_1_form4_stage_1_cancers_25_posrisk_6_negrisk_40 | GPs | hypothetical |
| 0.026531 | 0.077031 | 0.14247 | pred_wait_1_detect_1_form4_stage_1_cancers_1_posrisk_8_negrisk_1 | GPs | hypothetical |
| 0.030154 | 0.083752 | 0.151369 | pred_wait_1_detect_1_form4_stage_1_cancers_5_posrisk_8_negrisk_1 | GPs | hypothetical |
| 0.035947 | 0.093946 | 0.167053 | pred_wait_1_detect_1_form4_stage_1_cancers_10_posrisk_8_negrisk_1 | GPs | hypothetical |
| 0.062542 | 0.139674 | 0.230739 | pred_wait_1_detect_1_form4_stage_1_cancers_25_posrisk_8_negrisk_1 | GPs | hypothetical |
| 0.020422 | 0.066498 | 0.126566 | pred_wait_1_detect_1_form4_stage_1_cancers_1_posrisk_8_negrisk_5 | GPs | hypothetical |
| 0.023755 | 0.072357 | 0.134879 | pred_wait_1_detect_1_form4_stage_1_cancers_5_posrisk_8_negrisk_5 | GPs | hypothetical |
| 0.02876 | 0.081356 | 0.148523 | pred_wait_1_detect_1_form4_stage_1_cancers_10_posrisk_8_negrisk_5 | GPs | hypothetical |
| 0.052063 | 0.122804 | 0.20864 | pred_wait_1_detect_1_form4_stage_1_cancers_25_posrisk_8_negrisk_5 | GPs | hypothetical |
| 0.014617 | 0.055196 | 0.108952 | pred_wait_1_detect_1_form4_stage_1_cancers_1_posrisk_8_negrisk_10 | GPs | hypothetical |
| 0.016834 | 0.060081 | 0.117257 | pred_wait_1_detect_1_form4_stage_1_cancers_5_posrisk_8_negrisk_10 | GPs | hypothetical |
| 0.020966 | 0.067707 | 0.129194 | pred_wait_1_detect_1_form4_stage_1_cancers_10_posrisk_8_negrisk_10 | GPs | hypothetical |
| 0.040592 | 0.104061 | 0.182106 | pred_wait_1_detect_1_form4_stage_1_cancers_25_posrisk_8_negrisk_10 | GPs | hypothetical |
| 0.002123 | 0.01975 | 0.050845 | pred_wait_1_detect_1_form4_stage_1_cancers_1_posrisk_8_negrisk_40 | GPs | hypothetical |
| 0.002481 | 0.021388 | 0.053261 | pred_wait_1_detect_1_form4_stage_1_cancers_5_posrisk_8_negrisk_40 | GPs | hypothetical |
| 0.003279 | 0.024122 | 0.058531 | pred_wait_1_detect_1_form4_stage_1_cancers_10_posrisk_8_negrisk_40 | GPs | hypothetical |
| 0.008088 | 0.039314 | 0.083522 | pred_wait_1_detect_1_form4_stage_1_cancers_25_posrisk_8_negrisk_40 | GPs | hypothetical |
| 0.303987 | 0.49117 | 0.680335 | pred_wait_1_detect_1_form4_stage_2_cancers_1_posrisk_2_negrisk_1 | GPs | hypothetical |
| 0.337389 | 0.533724 | 0.725643 | pred_wait_1_detect_1_form4_stage_2_cancers_5_posrisk_2_negrisk_1 | GPs | hypothetical |
| 0.386562 | 0.590536 | 0.781397 | pred_wait_1_detect_1_form4_stage_2_cancers_10_posrisk_2_negrisk_1 | GPs | hypothetical |
| 0.56791 | 0.754595 | 0.901387 | pred_wait_1_detect_1_form4_stage_2_cancers_25_posrisk_2_negrisk_1 | GPs | hypothetical |
| 0.264107 | 0.449719 | 0.640976 | pred_wait_1_detect_1_form4_stage_2_cancers_1_posrisk_2_negrisk_5 | GPs | hypothetical |
| 0.294761 | 0.491222 | 0.689509 | pred_wait_1_detect_1_form4_stage_2_cancers_5_posrisk_2_negrisk_5 | GPs | hypothetical |
| 0.339423 | 0.547987 | 0.749008 | pred_wait_1_detect_1_form4_stage_2_cancers_10_posrisk_2_negrisk_5 | GPs | hypothetical |
| 0.519522 | 0.721161 | 0.884091 | pred_wait_1_detect_1_form4_stage_2_cancers_25_posrisk_2_negrisk_5 | GPs | hypothetical |
| 0.216659 | 0.397211 | 0.589637 | pred_wait_1_detect_1_form4_stage_2_cancers_1_posrisk_2_negrisk_10 | GPs | hypothetical |
| 0.243033 | 0.436297 | 0.63862 | pred_wait_1_detect_1_form4_stage_2_cancers_5_posrisk_2_negrisk_10 | GPs | hypothetical |
| 0.282856 | 0.491391 | 0.701892 | pred_wait_1_detect_1_form4_stage_2_cancers_10_posrisk_2_negrisk_10 | GPs | hypothetical |
| 0.45484 | 0.672423 | 0.856132 | pred_wait_1_detect_1_form4_stage_2_cancers_25_posrisk_2_negrisk_10 | GPs | hypothetical |
| 0.056062 | 0.156536 | 0.284003 | pred_wait_1_detect_1_form4_stage_2_cancers_1_posrisk_2_negrisk_40 | GPs | hypothetical |
| 0.063175 | 0.172773 | 0.312522 | pred_wait_1_detect_1_form4_stage_2_cancers_5_posrisk_2_negrisk_40 | GPs | hypothetical |
| 0.075459 | 0.198764 | 0.355387 | pred_wait_1_detect_1_form4_stage_2_cancers_10_posrisk_2_negrisk_40 | GPs | hypothetical |
| 0.148482 | 0.323884 | 0.528296 | pred_wait_1_detect_1_form4_stage_2_cancers_25_posrisk_2_negrisk_40 | GPs | hypothetical |
| 0.109547 | 0.240304 | 0.394291 | pred_wait_1_detect_1_form4_stage_2_cancers_1_posrisk_4_negrisk_1 | GPs | hypothetical |
| 0.122251 | 0.26442 | 0.429584 | pred_wait_1_detect_1_form4_stage_2_cancers_5_posrisk_4_negrisk_1 | GPs | hypothetical |
| 0.142905 | 0.300534 | 0.480386 | pred_wait_1_detect_1_form4_stage_2_cancers_10_posrisk_4_negrisk_1 | GPs | hypothetical |
| 0.250066 | 0.446788 | 0.651691 | pred_wait_1_detect_1_form4_stage_2_cancers_25_posrisk_4_negrisk_1 | GPs | hypothetical |
| 0.087428 | 0.207584 | 0.353139 | pred_wait_1_detect_1_form4_stage_2_cancers_1_posrisk_4_negrisk_5 | GPs | hypothetical |
| 0.097824 | 0.228828 | 0.385715 | pred_wait_1_detect_1_form4_stage_2_cancers_5_posrisk_4_negrisk_5 | GPs | hypothetical |
| 0.114609 | 0.261381 | 0.434085 | pred_wait_1_detect_1_form4_stage_2_cancers_10_posrisk_4_negrisk_5 | GPs | hypothetical |
| 0.209791 | 0.401397 | 0.609054 | pred_wait_1_detect_1_form4_stage_2_cancers_25_posrisk_4_negrisk_5 | GPs | hypothetical |
| 0.066127 | 0.170744 | 0.304518 | pred_wait_1_detect_1_form4_stage_2_cancers_1_posrisk_4_negrisk_10 | GPs | hypothetical |
| 0.073137 | 0.188371 | 0.332958 | pred_wait_1_detect_1_form4_stage_2_cancers_5_posrisk_4_negrisk_10 | GPs | hypothetical |
| 0.086993 | 0.216151 | 0.376289 | pred_wait_1_detect_1_form4_stage_2_cancers_10_posrisk_4_negrisk_10 | GPs | hypothetical |
| 0.16464 | 0.345036 | 0.550102 | pred_wait_1_detect_1_form4_stage_2_cancers_25_posrisk_4_negrisk_10 | GPs | hypothetical |
| 0.011934 | 0.052231 | 0.115253 | pred_wait_1_detect_1_form4_stage_2_cancers_1_posrisk_4_negrisk_40 | GPs | hypothetical |
| 0.013272 | 0.056813 | 0.124409 | pred_wait_1_detect_1_form4_stage_2_cancers_5_posrisk_4_negrisk_40 | GPs | hypothetical |
| 0.015915 | 0.064978 | 0.140378 | pred_wait_1_detect_1_form4_stage_2_cancers_10_posrisk_4_negrisk_40 | GPs | hypothetical |
| 0.036407 | 0.11642 | 0.231362 | pred_wait_1_detect_1_form4_stage_2_cancers_25_posrisk_4_negrisk_40 | GPs | hypothetical |
| 0.04662 | 0.120473 | 0.217205 | pred_wait_1_detect_1_form4_stage_2_cancers_1_posrisk_6_negrisk_1 | GPs | hypothetical |
| 0.052975 | 0.131826 | 0.233437 | pred_wait_1_detect_1_form4_stage_2_cancers_5_posrisk_6_negrisk_1 | GPs | hypothetical |
| 0.061284 | 0.14935 | 0.259542 | pred_wait_1_detect_1_form4_stage_2_cancers_10_posrisk_6_negrisk_1 | GPs | hypothetical |
| 0.109005 | 0.22944 | 0.369791 | pred_wait_1_detect_1_form4_stage_2_cancers_25_posrisk_6_negrisk_1 | GPs | hypothetical |
| 0.036111 | 0.102505 | 0.191399 | pred_wait_1_detect_1_form4_stage_2_cancers_1_posrisk_6_negrisk_5 | GPs | hypothetical |
| 0.041064 | 0.112244 | 0.20671 | pred_wait_1_detect_1_form4_stage_2_cancers_5_posrisk_6_negrisk_5 | GPs | hypothetical |
| 0.04866 | 0.127537 | 0.22989 | pred_wait_1_detect_1_form4_stage_2_cancers_10_posrisk_6_negrisk_5 | GPs | hypothetical |
| 0.088753 | 0.200367 | 0.333966 | pred_wait_1_detect_1_form4_stage_2_cancers_25_posrisk_6_negrisk_5 | GPs | hypothetical |
| 0.026075 | 0.083252 | 0.161633 | pred_wait_1_detect_1_form4_stage_2_cancers_1_posrisk_6_negrisk_10 | GPs | hypothetical |
| 0.029829 | 0.091163 | 0.176037 | pred_wait_1_detect_1_form4_stage_2_cancers_5_posrisk_6_negrisk_10 | GPs | hypothetical |
| 0.035325 | 0.103858 | 0.195946 | pred_wait_1_detect_1_form4_stage_2_cancers_10_posrisk_6_negrisk_10 | GPs | hypothetical |
| 0.068687 | 0.167477 | 0.28956 | pred_wait_1_detect_1_form4_stage_2_cancers_25_posrisk_6_negrisk_10 | GPs | hypothetical |
| 0.004109 | 0.026114 | 0.064679 | pred_wait_1_detect_1_form4_stage_2_cancers_1_posrisk_6_negrisk_40 | GPs | hypothetical |
| 0.004675 | 0.02827 | 0.068103 | pred_wait_1_detect_1_form4_stage_2_cancers_5_posrisk_6_negrisk_40 | GPs | hypothetical |
| 0.005886 | 0.032088 | 0.07588 | pred_wait_1_detect_1_form4_stage_2_cancers_10_posrisk_6_negrisk_40 | GPs | hypothetical |
| 0.013687 | 0.055808 | 0.118819 | pred_wait_1_detect_1_form4_stage_2_cancers_25_posrisk_6_negrisk_40 | GPs | hypothetical |
| 0.026531 | 0.077031 | 0.14247 | pred_wait_1_detect_1_form4_stage_2_cancers_1_posrisk_8_negrisk_1 | GPs | hypothetical |
| 0.030154 | 0.083752 | 0.151369 | pred_wait_1_detect_1_form4_stage_2_cancers_5_posrisk_8_negrisk_1 | GPs | hypothetical |
| 0.035947 | 0.093946 | 0.167053 | pred_wait_1_detect_1_form4_stage_2_cancers_10_posrisk_8_negrisk_1 | GPs | hypothetical |
| 0.062542 | 0.139674 | 0.230739 | pred_wait_1_detect_1_form4_stage_2_cancers_25_posrisk_8_negrisk_1 | GPs | hypothetical |
| 0.020422 | 0.066498 | 0.126566 | pred_wait_1_detect_1_form4_stage_2_cancers_1_posrisk_8_negrisk_5 | GPs | hypothetical |
| 0.023755 | 0.072357 | 0.134879 | pred_wait_1_detect_1_form4_stage_2_cancers_5_posrisk_8_negrisk_5 | GPs | hypothetical |
| 0.02876 | 0.081356 | 0.148523 | pred_wait_1_detect_1_form4_stage_2_cancers_10_posrisk_8_negrisk_5 | GPs | hypothetical |
| 0.052063 | 0.122804 | 0.20864 | pred_wait_1_detect_1_form4_stage_2_cancers_25_posrisk_8_negrisk_5 | GPs | hypothetical |
| 0.014617 | 0.055196 | 0.108952 | pred_wait_1_detect_1_form4_stage_2_cancers_1_posrisk_8_negrisk_10 | GPs | hypothetical |
| 0.016834 | 0.060081 | 0.117257 | pred_wait_1_detect_1_form4_stage_2_cancers_5_posrisk_8_negrisk_10 | GPs | hypothetical |
| 0.020966 | 0.067707 | 0.129194 | pred_wait_1_detect_1_form4_stage_2_cancers_10_posrisk_8_negrisk_10 | GPs | hypothetical |
| 0.040592 | 0.104061 | 0.182106 | pred_wait_1_detect_1_form4_stage_2_cancers_25_posrisk_8_negrisk_10 | GPs | hypothetical |
| 0.002123 | 0.01975 | 0.050845 | pred_wait_1_detect_1_form4_stage_2_cancers_1_posrisk_8_negrisk_40 | GPs | hypothetical |
| 0.002481 | 0.021388 | 0.053261 | pred_wait_1_detect_1_form4_stage_2_cancers_5_posrisk_8_negrisk_40 | GPs | hypothetical |
| 0.003279 | 0.024122 | 0.058531 | pred_wait_1_detect_1_form4_stage_2_cancers_10_posrisk_8_negrisk_40 | GPs | hypothetical |
| 0.008088 | 0.039314 | 0.083522 | pred_wait_1_detect_1_form4_stage_2_cancers_25_posrisk_8_negrisk_40 | GPs | hypothetical |
| 0.303987 | 0.49117 | 0.680335 | pred_wait_1_detect_2_form1_stage_1_cancers_1_posrisk_2_negrisk_1 | GPs | hypothetical |
| 0.337389 | 0.533724 | 0.725643 | pred_wait_1_detect_2_form1_stage_1_cancers_5_posrisk_2_negrisk_1 | GPs | hypothetical |
| 0.386562 | 0.590536 | 0.781397 | pred_wait_1_detect_2_form1_stage_1_cancers_10_posrisk_2_negrisk_1 | GPs | hypothetical |
| 0.56791 | 0.754595 | 0.901387 | pred_wait_1_detect_2_form1_stage_1_cancers_25_posrisk_2_negrisk_1 | GPs | hypothetical |
| 0.264107 | 0.449719 | 0.640976 | pred_wait_1_detect_2_form1_stage_1_cancers_1_posrisk_2_negrisk_5 | GPs | hypothetical |
| 0.294761 | 0.491222 | 0.689509 | pred_wait_1_detect_2_form1_stage_1_cancers_5_posrisk_2_negrisk_5 | GPs | hypothetical |
| 0.339423 | 0.547987 | 0.749008 | pred_wait_1_detect_2_form1_stage_1_cancers_10_posrisk_2_negrisk_5 | GPs | hypothetical |
| 0.519522 | 0.721161 | 0.884091 | pred_wait_1_detect_2_form1_stage_1_cancers_25_posrisk_2_negrisk_5 | GPs | hypothetical |
| 0.216659 | 0.397211 | 0.589637 | pred_wait_1_detect_2_form1_stage_1_cancers_1_posrisk_2_negrisk_10 | GPs | hypothetical |
| 0.243033 | 0.436297 | 0.63862 | pred_wait_1_detect_2_form1_stage_1_cancers_5_posrisk_2_negrisk_10 | GPs | hypothetical |
| 0.282856 | 0.491391 | 0.701892 | pred_wait_1_detect_2_form1_stage_1_cancers_10_posrisk_2_negrisk_10 | GPs | hypothetical |
| 0.45484 | 0.672423 | 0.856132 | pred_wait_1_detect_2_form1_stage_1_cancers_25_posrisk_2_negrisk_10 | GPs | hypothetical |
| 0.056062 | 0.156536 | 0.284003 | pred_wait_1_detect_2_form1_stage_1_cancers_1_posrisk_2_negrisk_40 | GPs | hypothetical |
| 0.063175 | 0.172773 | 0.312522 | pred_wait_1_detect_2_form1_stage_1_cancers_5_posrisk_2_negrisk_40 | GPs | hypothetical |
| 0.075459 | 0.198764 | 0.355387 | pred_wait_1_detect_2_form1_stage_1_cancers_10_posrisk_2_negrisk_40 | GPs | hypothetical |
| 0.148482 | 0.323884 | 0.528296 | pred_wait_1_detect_2_form1_stage_1_cancers_25_posrisk_2_negrisk_40 | GPs | hypothetical |
| 0.109547 | 0.240304 | 0.394291 | pred_wait_1_detect_2_form1_stage_1_cancers_1_posrisk_4_negrisk_1 | GPs | hypothetical |
| 0.122251 | 0.26442 | 0.429584 | pred_wait_1_detect_2_form1_stage_1_cancers_5_posrisk_4_negrisk_1 | GPs | hypothetical |
| 0.142905 | 0.300534 | 0.480386 | pred_wait_1_detect_2_form1_stage_1_cancers_10_posrisk_4_negrisk_1 | GPs | hypothetical |
| 0.250066 | 0.446788 | 0.651691 | pred_wait_1_detect_2_form1_stage_1_cancers_25_posrisk_4_negrisk_1 | GPs | hypothetical |
| 0.087428 | 0.207584 | 0.353139 | pred_wait_1_detect_2_form1_stage_1_cancers_1_posrisk_4_negrisk_5 | GPs | hypothetical |
| 0.097824 | 0.228828 | 0.385715 | pred_wait_1_detect_2_form1_stage_1_cancers_5_posrisk_4_negrisk_5 | GPs | hypothetical |
| 0.114609 | 0.261381 | 0.434085 | pred_wait_1_detect_2_form1_stage_1_cancers_10_posrisk_4_negrisk_5 | GPs | hypothetical |
| 0.209791 | 0.401397 | 0.609054 | pred_wait_1_detect_2_form1_stage_1_cancers_25_posrisk_4_negrisk_5 | GPs | hypothetical |
| 0.066127 | 0.170744 | 0.304518 | pred_wait_1_detect_2_form1_stage_1_cancers_1_posrisk_4_negrisk_10 | GPs | hypothetical |
| 0.073137 | 0.188371 | 0.332958 | pred_wait_1_detect_2_form1_stage_1_cancers_5_posrisk_4_negrisk_10 | GPs | hypothetical |
| 0.086993 | 0.216151 | 0.376289 | pred_wait_1_detect_2_form1_stage_1_cancers_10_posrisk_4_negrisk_10 | GPs | hypothetical |
| 0.16464 | 0.345036 | 0.550102 | pred_wait_1_detect_2_form1_stage_1_cancers_25_posrisk_4_negrisk_10 | GPs | hypothetical |
| 0.011934 | 0.052231 | 0.115253 | pred_wait_1_detect_2_form1_stage_1_cancers_1_posrisk_4_negrisk_40 | GPs | hypothetical |
| 0.013272 | 0.056813 | 0.124409 | pred_wait_1_detect_2_form1_stage_1_cancers_5_posrisk_4_negrisk_40 | GPs | hypothetical |
| 0.015915 | 0.064978 | 0.140378 | pred_wait_1_detect_2_form1_stage_1_cancers_10_posrisk_4_negrisk_40 | GPs | hypothetical |
| 0.036407 | 0.11642 | 0.231362 | pred_wait_1_detect_2_form1_stage_1_cancers_25_posrisk_4_negrisk_40 | GPs | hypothetical |
| 0.04662 | 0.120473 | 0.217205 | pred_wait_1_detect_2_form1_stage_1_cancers_1_posrisk_6_negrisk_1 | GPs | hypothetical |
| 0.052975 | 0.131826 | 0.233437 | pred_wait_1_detect_2_form1_stage_1_cancers_5_posrisk_6_negrisk_1 | GPs | hypothetical |
| 0.061284 | 0.14935 | 0.259542 | pred_wait_1_detect_2_form1_stage_1_cancers_10_posrisk_6_negrisk_1 | GPs | hypothetical |
| 0.109005 | 0.22944 | 0.369791 | pred_wait_1_detect_2_form1_stage_1_cancers_25_posrisk_6_negrisk_1 | GPs | hypothetical |
| 0.036111 | 0.102505 | 0.191399 | pred_wait_1_detect_2_form1_stage_1_cancers_1_posrisk_6_negrisk_5 | GPs | hypothetical |
| 0.041064 | 0.112244 | 0.20671 | pred_wait_1_detect_2_form1_stage_1_cancers_5_posrisk_6_negrisk_5 | GPs | hypothetical |
| 0.04866 | 0.127537 | 0.22989 | pred_wait_1_detect_2_form1_stage_1_cancers_10_posrisk_6_negrisk_5 | GPs | hypothetical |
| 0.088753 | 0.200367 | 0.333966 | pred_wait_1_detect_2_form1_stage_1_cancers_25_posrisk_6_negrisk_5 | GPs | hypothetical |
| 0.026075 | 0.083252 | 0.161633 | pred_wait_1_detect_2_form1_stage_1_cancers_1_posrisk_6_negrisk_10 | GPs | hypothetical |
| 0.029829 | 0.091163 | 0.176037 | pred_wait_1_detect_2_form1_stage_1_cancers_5_posrisk_6_negrisk_10 | GPs | hypothetical |
| 0.035325 | 0.103858 | 0.195946 | pred_wait_1_detect_2_form1_stage_1_cancers_10_posrisk_6_negrisk_10 | GPs | hypothetical |
| 0.068687 | 0.167477 | 0.28956 | pred_wait_1_detect_2_form1_stage_1_cancers_25_posrisk_6_negrisk_10 | GPs | hypothetical |
| 0.004109 | 0.026114 | 0.064679 | pred_wait_1_detect_2_form1_stage_1_cancers_1_posrisk_6_negrisk_40 | GPs | hypothetical |
| 0.004675 | 0.02827 | 0.068103 | pred_wait_1_detect_2_form1_stage_1_cancers_5_posrisk_6_negrisk_40 | GPs | hypothetical |
| 0.005886 | 0.032088 | 0.07588 | pred_wait_1_detect_2_form1_stage_1_cancers_10_posrisk_6_negrisk_40 | GPs | hypothetical |
| 0.013687 | 0.055808 | 0.118819 | pred_wait_1_detect_2_form1_stage_1_cancers_25_posrisk_6_negrisk_40 | GPs | hypothetical |
| 0.026531 | 0.077031 | 0.14247 | pred_wait_1_detect_2_form1_stage_1_cancers_1_posrisk_8_negrisk_1 | GPs | hypothetical |
| 0.030154 | 0.083752 | 0.151369 | pred_wait_1_detect_2_form1_stage_1_cancers_5_posrisk_8_negrisk_1 | GPs | hypothetical |
| 0.035947 | 0.093946 | 0.167053 | pred_wait_1_detect_2_form1_stage_1_cancers_10_posrisk_8_negrisk_1 | GPs | hypothetical |
| 0.062542 | 0.139674 | 0.230739 | pred_wait_1_detect_2_form1_stage_1_cancers_25_posrisk_8_negrisk_1 | GPs | hypothetical |
| 0.020422 | 0.066498 | 0.126566 | pred_wait_1_detect_2_form1_stage_1_cancers_1_posrisk_8_negrisk_5 | GPs | hypothetical |
| 0.023755 | 0.072357 | 0.134879 | pred_wait_1_detect_2_form1_stage_1_cancers_5_posrisk_8_negrisk_5 | GPs | hypothetical |
| 0.02876 | 0.081356 | 0.148523 | pred_wait_1_detect_2_form1_stage_1_cancers_10_posrisk_8_negrisk_5 | GPs | hypothetical |
| 0.052063 | 0.122804 | 0.20864 | pred_wait_1_detect_2_form1_stage_1_cancers_25_posrisk_8_negrisk_5 | GPs | hypothetical |
| 0.014617 | 0.055196 | 0.108952 | pred_wait_1_detect_2_form1_stage_1_cancers_1_posrisk_8_negrisk_10 | GPs | hypothetical |
| 0.016834 | 0.060081 | 0.117257 | pred_wait_1_detect_2_form1_stage_1_cancers_5_posrisk_8_negrisk_10 | GPs | hypothetical |
| 0.020966 | 0.067707 | 0.129194 | pred_wait_1_detect_2_form1_stage_1_cancers_10_posrisk_8_negrisk_10 | GPs | hypothetical |
| 0.040592 | 0.104061 | 0.182106 | pred_wait_1_detect_2_form1_stage_1_cancers_25_posrisk_8_negrisk_10 | GPs | hypothetical |
| 0.002123 | 0.01975 | 0.050845 | pred_wait_1_detect_2_form1_stage_1_cancers_1_posrisk_8_negrisk_40 | GPs | hypothetical |
| 0.002481 | 0.021388 | 0.053261 | pred_wait_1_detect_2_form1_stage_1_cancers_5_posrisk_8_negrisk_40 | GPs | hypothetical |
| 0.003279 | 0.024122 | 0.058531 | pred_wait_1_detect_2_form1_stage_1_cancers_10_posrisk_8_negrisk_40 | GPs | hypothetical |
| 0.008088 | 0.039314 | 0.083522 | pred_wait_1_detect_2_form1_stage_1_cancers_25_posrisk_8_negrisk_40 | GPs | hypothetical |
| 0.303987 | 0.49117 | 0.680335 | pred_wait_1_detect_2_form1_stage_2_cancers_1_posrisk_2_negrisk_1 | GPs | hypothetical |
| 0.337389 | 0.533724 | 0.725643 | pred_wait_1_detect_2_form1_stage_2_cancers_5_posrisk_2_negrisk_1 | GPs | hypothetical |
| 0.386562 | 0.590536 | 0.781397 | pred_wait_1_detect_2_form1_stage_2_cancers_10_posrisk_2_negrisk_1 | GPs | hypothetical |
| 0.56791 | 0.754595 | 0.901387 | pred_wait_1_detect_2_form1_stage_2_cancers_25_posrisk_2_negrisk_1 | GPs | hypothetical |
| 0.264107 | 0.449719 | 0.640976 | pred_wait_1_detect_2_form1_stage_2_cancers_1_posrisk_2_negrisk_5 | GPs | hypothetical |
| 0.294761 | 0.491222 | 0.689509 | pred_wait_1_detect_2_form1_stage_2_cancers_5_posrisk_2_negrisk_5 | GPs | hypothetical |
| 0.339423 | 0.547987 | 0.749008 | pred_wait_1_detect_2_form1_stage_2_cancers_10_posrisk_2_negrisk_5 | GPs | hypothetical |
| 0.519522 | 0.721161 | 0.884091 | pred_wait_1_detect_2_form1_stage_2_cancers_25_posrisk_2_negrisk_5 | GPs | hypothetical |
| 0.216659 | 0.397211 | 0.589637 | pred_wait_1_detect_2_form1_stage_2_cancers_1_posrisk_2_negrisk_10 | GPs | hypothetical |
| 0.243033 | 0.436297 | 0.63862 | pred_wait_1_detect_2_form1_stage_2_cancers_5_posrisk_2_negrisk_10 | GPs | hypothetical |
| 0.282856 | 0.491391 | 0.701892 | pred_wait_1_detect_2_form1_stage_2_cancers_10_posrisk_2_negrisk_10 | GPs | hypothetical |
| 0.45484 | 0.672423 | 0.856132 | pred_wait_1_detect_2_form1_stage_2_cancers_25_posrisk_2_negrisk_10 | GPs | hypothetical |
| 0.056062 | 0.156536 | 0.284003 | pred_wait_1_detect_2_form1_stage_2_cancers_1_posrisk_2_negrisk_40 | GPs | hypothetical |
| 0.063175 | 0.172773 | 0.312522 | pred_wait_1_detect_2_form1_stage_2_cancers_5_posrisk_2_negrisk_40 | GPs | hypothetical |
| 0.075459 | 0.198764 | 0.355387 | pred_wait_1_detect_2_form1_stage_2_cancers_10_posrisk_2_negrisk_40 | GPs | hypothetical |
| 0.148482 | 0.323884 | 0.528296 | pred_wait_1_detect_2_form1_stage_2_cancers_25_posrisk_2_negrisk_40 | GPs | hypothetical |
| 0.109547 | 0.240304 | 0.394291 | pred_wait_1_detect_2_form1_stage_2_cancers_1_posrisk_4_negrisk_1 | GPs | hypothetical |
| 0.122251 | 0.26442 | 0.429584 | pred_wait_1_detect_2_form1_stage_2_cancers_5_posrisk_4_negrisk_1 | GPs | hypothetical |
| 0.142905 | 0.300534 | 0.480386 | pred_wait_1_detect_2_form1_stage_2_cancers_10_posrisk_4_negrisk_1 | GPs | hypothetical |
| 0.250066 | 0.446788 | 0.651691 | pred_wait_1_detect_2_form1_stage_2_cancers_25_posrisk_4_negrisk_1 | GPs | hypothetical |
| 0.087428 | 0.207584 | 0.353139 | pred_wait_1_detect_2_form1_stage_2_cancers_1_posrisk_4_negrisk_5 | GPs | hypothetical |
| 0.097824 | 0.228828 | 0.385715 | pred_wait_1_detect_2_form1_stage_2_cancers_5_posrisk_4_negrisk_5 | GPs | hypothetical |
| 0.114609 | 0.261381 | 0.434085 | pred_wait_1_detect_2_form1_stage_2_cancers_10_posrisk_4_negrisk_5 | GPs | hypothetical |
| 0.209791 | 0.401397 | 0.609054 | pred_wait_1_detect_2_form1_stage_2_cancers_25_posrisk_4_negrisk_5 | GPs | hypothetical |
| 0.066127 | 0.170744 | 0.304518 | pred_wait_1_detect_2_form1_stage_2_cancers_1_posrisk_4_negrisk_10 | GPs | hypothetical |
| 0.073137 | 0.188371 | 0.332958 | pred_wait_1_detect_2_form1_stage_2_cancers_5_posrisk_4_negrisk_10 | GPs | hypothetical |
| 0.086993 | 0.216151 | 0.376289 | pred_wait_1_detect_2_form1_stage_2_cancers_10_posrisk_4_negrisk_10 | GPs | hypothetical |
| 0.16464 | 0.345036 | 0.550102 | pred_wait_1_detect_2_form1_stage_2_cancers_25_posrisk_4_negrisk_10 | GPs | hypothetical |
| 0.011934 | 0.052231 | 0.115253 | pred_wait_1_detect_2_form1_stage_2_cancers_1_posrisk_4_negrisk_40 | GPs | hypothetical |
| 0.013272 | 0.056813 | 0.124409 | pred_wait_1_detect_2_form1_stage_2_cancers_5_posrisk_4_negrisk_40 | GPs | hypothetical |
| 0.015915 | 0.064978 | 0.140378 | pred_wait_1_detect_2_form1_stage_2_cancers_10_posrisk_4_negrisk_40 | GPs | hypothetical |
| 0.036407 | 0.11642 | 0.231362 | pred_wait_1_detect_2_form1_stage_2_cancers_25_posrisk_4_negrisk_40 | GPs | hypothetical |
| 0.04662 | 0.120473 | 0.217205 | pred_wait_1_detect_2_form1_stage_2_cancers_1_posrisk_6_negrisk_1 | GPs | hypothetical |
| 0.052975 | 0.131826 | 0.233437 | pred_wait_1_detect_2_form1_stage_2_cancers_5_posrisk_6_negrisk_1 | GPs | hypothetical |
| 0.061284 | 0.14935 | 0.259542 | pred_wait_1_detect_2_form1_stage_2_cancers_10_posrisk_6_negrisk_1 | GPs | hypothetical |
| 0.109005 | 0.22944 | 0.369791 | pred_wait_1_detect_2_form1_stage_2_cancers_25_posrisk_6_negrisk_1 | GPs | hypothetical |
| 0.036111 | 0.102505 | 0.191399 | pred_wait_1_detect_2_form1_stage_2_cancers_1_posrisk_6_negrisk_5 | GPs | hypothetical |
| 0.041064 | 0.112244 | 0.20671 | pred_wait_1_detect_2_form1_stage_2_cancers_5_posrisk_6_negrisk_5 | GPs | hypothetical |
| 0.04866 | 0.127537 | 0.22989 | pred_wait_1_detect_2_form1_stage_2_cancers_10_posrisk_6_negrisk_5 | GPs | hypothetical |
| 0.088753 | 0.200367 | 0.333966 | pred_wait_1_detect_2_form1_stage_2_cancers_25_posrisk_6_negrisk_5 | GPs | hypothetical |
| 0.026075 | 0.083252 | 0.161633 | pred_wait_1_detect_2_form1_stage_2_cancers_1_posrisk_6_negrisk_10 | GPs | hypothetical |
| 0.029829 | 0.091163 | 0.176037 | pred_wait_1_detect_2_form1_stage_2_cancers_5_posrisk_6_negrisk_10 | GPs | hypothetical |
| 0.035325 | 0.103858 | 0.195946 | pred_wait_1_detect_2_form1_stage_2_cancers_10_posrisk_6_negrisk_10 | GPs | hypothetical |
| 0.068687 | 0.167477 | 0.28956 | pred_wait_1_detect_2_form1_stage_2_cancers_25_posrisk_6_negrisk_10 | GPs | hypothetical |
| 0.004109 | 0.026114 | 0.064679 | pred_wait_1_detect_2_form1_stage_2_cancers_1_posrisk_6_negrisk_40 | GPs | hypothetical |
| 0.004675 | 0.02827 | 0.068103 | pred_wait_1_detect_2_form1_stage_2_cancers_5_posrisk_6_negrisk_40 | GPs | hypothetical |
| 0.005886 | 0.032088 | 0.07588 | pred_wait_1_detect_2_form1_stage_2_cancers_10_posrisk_6_negrisk_40 | GPs | hypothetical |
| 0.013687 | 0.055808 | 0.118819 | pred_wait_1_detect_2_form1_stage_2_cancers_25_posrisk_6_negrisk_40 | GPs | hypothetical |
| 0.026531 | 0.077031 | 0.14247 | pred_wait_1_detect_2_form1_stage_2_cancers_1_posrisk_8_negrisk_1 | GPs | hypothetical |
| 0.030154 | 0.083752 | 0.151369 | pred_wait_1_detect_2_form1_stage_2_cancers_5_posrisk_8_negrisk_1 | GPs | hypothetical |
| 0.035947 | 0.093946 | 0.167053 | pred_wait_1_detect_2_form1_stage_2_cancers_10_posrisk_8_negrisk_1 | GPs | hypothetical |
| 0.062542 | 0.139674 | 0.230739 | pred_wait_1_detect_2_form1_stage_2_cancers_25_posrisk_8_negrisk_1 | GPs | hypothetical |
| 0.020422 | 0.066498 | 0.126566 | pred_wait_1_detect_2_form1_stage_2_cancers_1_posrisk_8_negrisk_5 | GPs | hypothetical |
| 0.023755 | 0.072357 | 0.134879 | pred_wait_1_detect_2_form1_stage_2_cancers_5_posrisk_8_negrisk_5 | GPs | hypothetical |
| 0.02876 | 0.081356 | 0.148523 | pred_wait_1_detect_2_form1_stage_2_cancers_10_posrisk_8_negrisk_5 | GPs | hypothetical |
| 0.052063 | 0.122804 | 0.20864 | pred_wait_1_detect_2_form1_stage_2_cancers_25_posrisk_8_negrisk_5 | GPs | hypothetical |
| 0.014617 | 0.055196 | 0.108952 | pred_wait_1_detect_2_form1_stage_2_cancers_1_posrisk_8_negrisk_10 | GPs | hypothetical |
| 0.016834 | 0.060081 | 0.117257 | pred_wait_1_detect_2_form1_stage_2_cancers_5_posrisk_8_negrisk_10 | GPs | hypothetical |
| 0.020966 | 0.067707 | 0.129194 | pred_wait_1_detect_2_form1_stage_2_cancers_10_posrisk_8_negrisk_10 | GPs | hypothetical |
| 0.040592 | 0.104061 | 0.182106 | pred_wait_1_detect_2_form1_stage_2_cancers_25_posrisk_8_negrisk_10 | GPs | hypothetical |
| 0.002123 | 0.01975 | 0.050845 | pred_wait_1_detect_2_form1_stage_2_cancers_1_posrisk_8_negrisk_40 | GPs | hypothetical |
| 0.002481 | 0.021388 | 0.053261 | pred_wait_1_detect_2_form1_stage_2_cancers_5_posrisk_8_negrisk_40 | GPs | hypothetical |
| 0.003279 | 0.024122 | 0.058531 | pred_wait_1_detect_2_form1_stage_2_cancers_10_posrisk_8_negrisk_40 | GPs | hypothetical |
| 0.008088 | 0.039314 | 0.083522 | pred_wait_1_detect_2_form1_stage_2_cancers_25_posrisk_8_negrisk_40 | GPs | hypothetical |
| 0.303987 | 0.49117 | 0.680335 | pred_wait_1_detect_2_form2_stage_1_cancers_1_posrisk_2_negrisk_1 | GPs | hypothetical |
| 0.337389 | 0.533724 | 0.725643 | pred_wait_1_detect_2_form2_stage_1_cancers_5_posrisk_2_negrisk_1 | GPs | hypothetical |
| 0.386562 | 0.590536 | 0.781397 | pred_wait_1_detect_2_form2_stage_1_cancers_10_posrisk_2_negrisk_1 | GPs | hypothetical |
| 0.56791 | 0.754595 | 0.901387 | pred_wait_1_detect_2_form2_stage_1_cancers_25_posrisk_2_negrisk_1 | GPs | hypothetical |
| 0.264107 | 0.449719 | 0.640976 | pred_wait_1_detect_2_form2_stage_1_cancers_1_posrisk_2_negrisk_5 | GPs | hypothetical |
| 0.294761 | 0.491222 | 0.689509 | pred_wait_1_detect_2_form2_stage_1_cancers_5_posrisk_2_negrisk_5 | GPs | hypothetical |
| 0.339423 | 0.547987 | 0.749008 | pred_wait_1_detect_2_form2_stage_1_cancers_10_posrisk_2_negrisk_5 | GPs | hypothetical |
| 0.519522 | 0.721161 | 0.884091 | pred_wait_1_detect_2_form2_stage_1_cancers_25_posrisk_2_negrisk_5 | GPs | hypothetical |
| 0.216659 | 0.397211 | 0.589637 | pred_wait_1_detect_2_form2_stage_1_cancers_1_posrisk_2_negrisk_10 | GPs | hypothetical |
| 0.243033 | 0.436297 | 0.63862 | pred_wait_1_detect_2_form2_stage_1_cancers_5_posrisk_2_negrisk_10 | GPs | hypothetical |
| 0.282856 | 0.491391 | 0.701892 | pred_wait_1_detect_2_form2_stage_1_cancers_10_posrisk_2_negrisk_10 | GPs | hypothetical |
| 0.45484 | 0.672423 | 0.856132 | pred_wait_1_detect_2_form2_stage_1_cancers_25_posrisk_2_negrisk_10 | GPs | hypothetical |
| 0.056062 | 0.156536 | 0.284003 | pred_wait_1_detect_2_form2_stage_1_cancers_1_posrisk_2_negrisk_40 | GPs | hypothetical |
| 0.063175 | 0.172773 | 0.312522 | pred_wait_1_detect_2_form2_stage_1_cancers_5_posrisk_2_negrisk_40 | GPs | hypothetical |
| 0.075459 | 0.198764 | 0.355387 | pred_wait_1_detect_2_form2_stage_1_cancers_10_posrisk_2_negrisk_40 | GPs | hypothetical |
| 0.148482 | 0.323884 | 0.528296 | pred_wait_1_detect_2_form2_stage_1_cancers_25_posrisk_2_negrisk_40 | GPs | hypothetical |
| 0.109547 | 0.240304 | 0.394291 | pred_wait_1_detect_2_form2_stage_1_cancers_1_posrisk_4_negrisk_1 | GPs | hypothetical |
| 0.122251 | 0.26442 | 0.429584 | pred_wait_1_detect_2_form2_stage_1_cancers_5_posrisk_4_negrisk_1 | GPs | hypothetical |
| 0.142905 | 0.300534 | 0.480386 | pred_wait_1_detect_2_form2_stage_1_cancers_10_posrisk_4_negrisk_1 | GPs | hypothetical |
| 0.250066 | 0.446788 | 0.651691 | pred_wait_1_detect_2_form2_stage_1_cancers_25_posrisk_4_negrisk_1 | GPs | hypothetical |
| 0.087428 | 0.207584 | 0.353139 | pred_wait_1_detect_2_form2_stage_1_cancers_1_posrisk_4_negrisk_5 | GPs | hypothetical |
| 0.097824 | 0.228828 | 0.385715 | pred_wait_1_detect_2_form2_stage_1_cancers_5_posrisk_4_negrisk_5 | GPs | hypothetical |
| 0.114609 | 0.261381 | 0.434085 | pred_wait_1_detect_2_form2_stage_1_cancers_10_posrisk_4_negrisk_5 | GPs | hypothetical |
| 0.209791 | 0.401397 | 0.609054 | pred_wait_1_detect_2_form2_stage_1_cancers_25_posrisk_4_negrisk_5 | GPs | hypothetical |
| 0.066127 | 0.170744 | 0.304518 | pred_wait_1_detect_2_form2_stage_1_cancers_1_posrisk_4_negrisk_10 | GPs | hypothetical |
| 0.073137 | 0.188371 | 0.332958 | pred_wait_1_detect_2_form2_stage_1_cancers_5_posrisk_4_negrisk_10 | GPs | hypothetical |
| 0.086993 | 0.216151 | 0.376289 | pred_wait_1_detect_2_form2_stage_1_cancers_10_posrisk_4_negrisk_10 | GPs | hypothetical |
| 0.16464 | 0.345036 | 0.550102 | pred_wait_1_detect_2_form2_stage_1_cancers_25_posrisk_4_negrisk_10 | GPs | hypothetical |
| 0.011934 | 0.052231 | 0.115253 | pred_wait_1_detect_2_form2_stage_1_cancers_1_posrisk_4_negrisk_40 | GPs | hypothetical |
| 0.013272 | 0.056813 | 0.124409 | pred_wait_1_detect_2_form2_stage_1_cancers_5_posrisk_4_negrisk_40 | GPs | hypothetical |
| 0.015915 | 0.064978 | 0.140378 | pred_wait_1_detect_2_form2_stage_1_cancers_10_posrisk_4_negrisk_40 | GPs | hypothetical |
| 0.036407 | 0.11642 | 0.231362 | pred_wait_1_detect_2_form2_stage_1_cancers_25_posrisk_4_negrisk_40 | GPs | hypothetical |
| 0.04662 | 0.120473 | 0.217205 | pred_wait_1_detect_2_form2_stage_1_cancers_1_posrisk_6_negrisk_1 | GPs | hypothetical |
| 0.052975 | 0.131826 | 0.233437 | pred_wait_1_detect_2_form2_stage_1_cancers_5_posrisk_6_negrisk_1 | GPs | hypothetical |
| 0.061284 | 0.14935 | 0.259542 | pred_wait_1_detect_2_form2_stage_1_cancers_10_posrisk_6_negrisk_1 | GPs | hypothetical |
| 0.109005 | 0.22944 | 0.369791 | pred_wait_1_detect_2_form2_stage_1_cancers_25_posrisk_6_negrisk_1 | GPs | hypothetical |
| 0.036111 | 0.102505 | 0.191399 | pred_wait_1_detect_2_form2_stage_1_cancers_1_posrisk_6_negrisk_5 | GPs | hypothetical |
| 0.041064 | 0.112244 | 0.20671 | pred_wait_1_detect_2_form2_stage_1_cancers_5_posrisk_6_negrisk_5 | GPs | hypothetical |
| 0.04866 | 0.127537 | 0.22989 | pred_wait_1_detect_2_form2_stage_1_cancers_10_posrisk_6_negrisk_5 | GPs | hypothetical |
| 0.088753 | 0.200367 | 0.333966 | pred_wait_1_detect_2_form2_stage_1_cancers_25_posrisk_6_negrisk_5 | GPs | hypothetical |
| 0.026075 | 0.083252 | 0.161633 | pred_wait_1_detect_2_form2_stage_1_cancers_1_posrisk_6_negrisk_10 | GPs | hypothetical |
| 0.029829 | 0.091163 | 0.176037 | pred_wait_1_detect_2_form2_stage_1_cancers_5_posrisk_6_negrisk_10 | GPs | hypothetical |
| 0.035325 | 0.103858 | 0.195946 | pred_wait_1_detect_2_form2_stage_1_cancers_10_posrisk_6_negrisk_10 | GPs | hypothetical |
| 0.068687 | 0.167477 | 0.28956 | pred_wait_1_detect_2_form2_stage_1_cancers_25_posrisk_6_negrisk_10 | GPs | hypothetical |
| 0.004109 | 0.026114 | 0.064679 | pred_wait_1_detect_2_form2_stage_1_cancers_1_posrisk_6_negrisk_40 | GPs | hypothetical |
| 0.004675 | 0.02827 | 0.068103 | pred_wait_1_detect_2_form2_stage_1_cancers_5_posrisk_6_negrisk_40 | GPs | hypothetical |
| 0.005886 | 0.032088 | 0.07588 | pred_wait_1_detect_2_form2_stage_1_cancers_10_posrisk_6_negrisk_40 | GPs | hypothetical |
| 0.013687 | 0.055808 | 0.118819 | pred_wait_1_detect_2_form2_stage_1_cancers_25_posrisk_6_negrisk_40 | GPs | hypothetical |
| 0.026531 | 0.077031 | 0.14247 | pred_wait_1_detect_2_form2_stage_1_cancers_1_posrisk_8_negrisk_1 | GPs | hypothetical |
| 0.030154 | 0.083752 | 0.151369 | pred_wait_1_detect_2_form2_stage_1_cancers_5_posrisk_8_negrisk_1 | GPs | hypothetical |
| 0.035947 | 0.093946 | 0.167053 | pred_wait_1_detect_2_form2_stage_1_cancers_10_posrisk_8_negrisk_1 | GPs | hypothetical |
| 0.062542 | 0.139674 | 0.230739 | pred_wait_1_detect_2_form2_stage_1_cancers_25_posrisk_8_negrisk_1 | GPs | hypothetical |
| 0.020422 | 0.066498 | 0.126566 | pred_wait_1_detect_2_form2_stage_1_cancers_1_posrisk_8_negrisk_5 | GPs | hypothetical |
| 0.023755 | 0.072357 | 0.134879 | pred_wait_1_detect_2_form2_stage_1_cancers_5_posrisk_8_negrisk_5 | GPs | hypothetical |
| 0.02876 | 0.081356 | 0.148523 | pred_wait_1_detect_2_form2_stage_1_cancers_10_posrisk_8_negrisk_5 | GPs | hypothetical |
| 0.052063 | 0.122804 | 0.20864 | pred_wait_1_detect_2_form2_stage_1_cancers_25_posrisk_8_negrisk_5 | GPs | hypothetical |
| 0.014617 | 0.055196 | 0.108952 | pred_wait_1_detect_2_form2_stage_1_cancers_1_posrisk_8_negrisk_10 | GPs | hypothetical |
| 0.016834 | 0.060081 | 0.117257 | pred_wait_1_detect_2_form2_stage_1_cancers_5_posrisk_8_negrisk_10 | GPs | hypothetical |
| 0.020966 | 0.067707 | 0.129194 | pred_wait_1_detect_2_form2_stage_1_cancers_10_posrisk_8_negrisk_10 | GPs | hypothetical |
| 0.040592 | 0.104061 | 0.182106 | pred_wait_1_detect_2_form2_stage_1_cancers_25_posrisk_8_negrisk_10 | GPs | hypothetical |
| 0.002123 | 0.01975 | 0.050845 | pred_wait_1_detect_2_form2_stage_1_cancers_1_posrisk_8_negrisk_40 | GPs | hypothetical |
| 0.002481 | 0.021388 | 0.053261 | pred_wait_1_detect_2_form2_stage_1_cancers_5_posrisk_8_negrisk_40 | GPs | hypothetical |
| 0.003279 | 0.024122 | 0.058531 | pred_wait_1_detect_2_form2_stage_1_cancers_10_posrisk_8_negrisk_40 | GPs | hypothetical |
| 0.008088 | 0.039314 | 0.083522 | pred_wait_1_detect_2_form2_stage_1_cancers_25_posrisk_8_negrisk_40 | GPs | hypothetical |
| 0.303987 | 0.49117 | 0.680335 | pred_wait_1_detect_2_form2_stage_2_cancers_1_posrisk_2_negrisk_1 | GPs | hypothetical |
| 0.337389 | 0.533724 | 0.725643 | pred_wait_1_detect_2_form2_stage_2_cancers_5_posrisk_2_negrisk_1 | GPs | hypothetical |
| 0.386562 | 0.590536 | 0.781397 | pred_wait_1_detect_2_form2_stage_2_cancers_10_posrisk_2_negrisk_1 | GPs | hypothetical |
| 0.56791 | 0.754595 | 0.901387 | pred_wait_1_detect_2_form2_stage_2_cancers_25_posrisk_2_negrisk_1 | GPs | hypothetical |
| 0.264107 | 0.449719 | 0.640976 | pred_wait_1_detect_2_form2_stage_2_cancers_1_posrisk_2_negrisk_5 | GPs | hypothetical |
| 0.294761 | 0.491222 | 0.689509 | pred_wait_1_detect_2_form2_stage_2_cancers_5_posrisk_2_negrisk_5 | GPs | hypothetical |
| 0.339423 | 0.547987 | 0.749008 | pred_wait_1_detect_2_form2_stage_2_cancers_10_posrisk_2_negrisk_5 | GPs | hypothetical |
| 0.519522 | 0.721161 | 0.884091 | pred_wait_1_detect_2_form2_stage_2_cancers_25_posrisk_2_negrisk_5 | GPs | hypothetical |
| 0.216659 | 0.397211 | 0.589637 | pred_wait_1_detect_2_form2_stage_2_cancers_1_posrisk_2_negrisk_10 | GPs | hypothetical |
| 0.243033 | 0.436297 | 0.63862 | pred_wait_1_detect_2_form2_stage_2_cancers_5_posrisk_2_negrisk_10 | GPs | hypothetical |
| 0.282856 | 0.491391 | 0.701892 | pred_wait_1_detect_2_form2_stage_2_cancers_10_posrisk_2_negrisk_10 | GPs | hypothetical |
| 0.45484 | 0.672423 | 0.856132 | pred_wait_1_detect_2_form2_stage_2_cancers_25_posrisk_2_negrisk_10 | GPs | hypothetical |
| 0.056062 | 0.156536 | 0.284003 | pred_wait_1_detect_2_form2_stage_2_cancers_1_posrisk_2_negrisk_40 | GPs | hypothetical |
| 0.063175 | 0.172773 | 0.312522 | pred_wait_1_detect_2_form2_stage_2_cancers_5_posrisk_2_negrisk_40 | GPs | hypothetical |
| 0.075459 | 0.198764 | 0.355387 | pred_wait_1_detect_2_form2_stage_2_cancers_10_posrisk_2_negrisk_40 | GPs | hypothetical |
| 0.148482 | 0.323884 | 0.528296 | pred_wait_1_detect_2_form2_stage_2_cancers_25_posrisk_2_negrisk_40 | GPs | hypothetical |
| 0.109547 | 0.240304 | 0.394291 | pred_wait_1_detect_2_form2_stage_2_cancers_1_posrisk_4_negrisk_1 | GPs | hypothetical |
| 0.122251 | 0.26442 | 0.429584 | pred_wait_1_detect_2_form2_stage_2_cancers_5_posrisk_4_negrisk_1 | GPs | hypothetical |
| 0.142905 | 0.300534 | 0.480386 | pred_wait_1_detect_2_form2_stage_2_cancers_10_posrisk_4_negrisk_1 | GPs | hypothetical |
| 0.250066 | 0.446788 | 0.651691 | pred_wait_1_detect_2_form2_stage_2_cancers_25_posrisk_4_negrisk_1 | GPs | hypothetical |
| 0.087428 | 0.207584 | 0.353139 | pred_wait_1_detect_2_form2_stage_2_cancers_1_posrisk_4_negrisk_5 | GPs | hypothetical |
| 0.097824 | 0.228828 | 0.385715 | pred_wait_1_detect_2_form2_stage_2_cancers_5_posrisk_4_negrisk_5 | GPs | hypothetical |
| 0.114609 | 0.261381 | 0.434085 | pred_wait_1_detect_2_form2_stage_2_cancers_10_posrisk_4_negrisk_5 | GPs | hypothetical |
| 0.209791 | 0.401397 | 0.609054 | pred_wait_1_detect_2_form2_stage_2_cancers_25_posrisk_4_negrisk_5 | GPs | hypothetical |
| 0.066127 | 0.170744 | 0.304518 | pred_wait_1_detect_2_form2_stage_2_cancers_1_posrisk_4_negrisk_10 | GPs | hypothetical |
| 0.073137 | 0.188371 | 0.332958 | pred_wait_1_detect_2_form2_stage_2_cancers_5_posrisk_4_negrisk_10 | GPs | hypothetical |
| 0.086993 | 0.216151 | 0.376289 | pred_wait_1_detect_2_form2_stage_2_cancers_10_posrisk_4_negrisk_10 | GPs | hypothetical |
| 0.16464 | 0.345036 | 0.550102 | pred_wait_1_detect_2_form2_stage_2_cancers_25_posrisk_4_negrisk_10 | GPs | hypothetical |
| 0.011934 | 0.052231 | 0.115253 | pred_wait_1_detect_2_form2_stage_2_cancers_1_posrisk_4_negrisk_40 | GPs | hypothetical |
| 0.013272 | 0.056813 | 0.124409 | pred_wait_1_detect_2_form2_stage_2_cancers_5_posrisk_4_negrisk_40 | GPs | hypothetical |
| 0.015915 | 0.064978 | 0.140378 | pred_wait_1_detect_2_form2_stage_2_cancers_10_posrisk_4_negrisk_40 | GPs | hypothetical |
| 0.036407 | 0.11642 | 0.231362 | pred_wait_1_detect_2_form2_stage_2_cancers_25_posrisk_4_negrisk_40 | GPs | hypothetical |
| 0.04662 | 0.120473 | 0.217205 | pred_wait_1_detect_2_form2_stage_2_cancers_1_posrisk_6_negrisk_1 | GPs | hypothetical |
| 0.052975 | 0.131826 | 0.233437 | pred_wait_1_detect_2_form2_stage_2_cancers_5_posrisk_6_negrisk_1 | GPs | hypothetical |
| 0.061284 | 0.14935 | 0.259542 | pred_wait_1_detect_2_form2_stage_2_cancers_10_posrisk_6_negrisk_1 | GPs | hypothetical |
| 0.109005 | 0.22944 | 0.369791 | pred_wait_1_detect_2_form2_stage_2_cancers_25_posrisk_6_negrisk_1 | GPs | hypothetical |
| 0.036111 | 0.102505 | 0.191399 | pred_wait_1_detect_2_form2_stage_2_cancers_1_posrisk_6_negrisk_5 | GPs | hypothetical |
| 0.041064 | 0.112244 | 0.20671 | pred_wait_1_detect_2_form2_stage_2_cancers_5_posrisk_6_negrisk_5 | GPs | hypothetical |
| 0.04866 | 0.127537 | 0.22989 | pred_wait_1_detect_2_form2_stage_2_cancers_10_posrisk_6_negrisk_5 | GPs | hypothetical |
| 0.088753 | 0.200367 | 0.333966 | pred_wait_1_detect_2_form2_stage_2_cancers_25_posrisk_6_negrisk_5 | GPs | hypothetical |
| 0.026075 | 0.083252 | 0.161633 | pred_wait_1_detect_2_form2_stage_2_cancers_1_posrisk_6_negrisk_10 | GPs | hypothetical |
| 0.029829 | 0.091163 | 0.176037 | pred_wait_1_detect_2_form2_stage_2_cancers_5_posrisk_6_negrisk_10 | GPs | hypothetical |
| 0.035325 | 0.103858 | 0.195946 | pred_wait_1_detect_2_form2_stage_2_cancers_10_posrisk_6_negrisk_10 | GPs | hypothetical |
| 0.068687 | 0.167477 | 0.28956 | pred_wait_1_detect_2_form2_stage_2_cancers_25_posrisk_6_negrisk_10 | GPs | hypothetical |
| 0.004109 | 0.026114 | 0.064679 | pred_wait_1_detect_2_form2_stage_2_cancers_1_posrisk_6_negrisk_40 | GPs | hypothetical |
| 0.004675 | 0.02827 | 0.068103 | pred_wait_1_detect_2_form2_stage_2_cancers_5_posrisk_6_negrisk_40 | GPs | hypothetical |
| 0.005886 | 0.032088 | 0.07588 | pred_wait_1_detect_2_form2_stage_2_cancers_10_posrisk_6_negrisk_40 | GPs | hypothetical |
| 0.013687 | 0.055808 | 0.118819 | pred_wait_1_detect_2_form2_stage_2_cancers_25_posrisk_6_negrisk_40 | GPs | hypothetical |
| 0.026531 | 0.077031 | 0.14247 | pred_wait_1_detect_2_form2_stage_2_cancers_1_posrisk_8_negrisk_1 | GPs | hypothetical |
| 0.030154 | 0.083752 | 0.151369 | pred_wait_1_detect_2_form2_stage_2_cancers_5_posrisk_8_negrisk_1 | GPs | hypothetical |
| 0.035947 | 0.093946 | 0.167053 | pred_wait_1_detect_2_form2_stage_2_cancers_10_posrisk_8_negrisk_1 | GPs | hypothetical |
| 0.062542 | 0.139674 | 0.230739 | pred_wait_1_detect_2_form2_stage_2_cancers_25_posrisk_8_negrisk_1 | GPs | hypothetical |
| 0.020422 | 0.066498 | 0.126566 | pred_wait_1_detect_2_form2_stage_2_cancers_1_posrisk_8_negrisk_5 | GPs | hypothetical |
| 0.023755 | 0.072357 | 0.134879 | pred_wait_1_detect_2_form2_stage_2_cancers_5_posrisk_8_negrisk_5 | GPs | hypothetical |
| 0.02876 | 0.081356 | 0.148523 | pred_wait_1_detect_2_form2_stage_2_cancers_10_posrisk_8_negrisk_5 | GPs | hypothetical |
| 0.052063 | 0.122804 | 0.20864 | pred_wait_1_detect_2_form2_stage_2_cancers_25_posrisk_8_negrisk_5 | GPs | hypothetical |
| 0.014617 | 0.055196 | 0.108952 | pred_wait_1_detect_2_form2_stage_2_cancers_1_posrisk_8_negrisk_10 | GPs | hypothetical |
| 0.016834 | 0.060081 | 0.117257 | pred_wait_1_detect_2_form2_stage_2_cancers_5_posrisk_8_negrisk_10 | GPs | hypothetical |
| 0.020966 | 0.067707 | 0.129194 | pred_wait_1_detect_2_form2_stage_2_cancers_10_posrisk_8_negrisk_10 | GPs | hypothetical |
| 0.040592 | 0.104061 | 0.182106 | pred_wait_1_detect_2_form2_stage_2_cancers_25_posrisk_8_negrisk_10 | GPs | hypothetical |
| 0.002123 | 0.01975 | 0.050845 | pred_wait_1_detect_2_form2_stage_2_cancers_1_posrisk_8_negrisk_40 | GPs | hypothetical |
| 0.002481 | 0.021388 | 0.053261 | pred_wait_1_detect_2_form2_stage_2_cancers_5_posrisk_8_negrisk_40 | GPs | hypothetical |
| 0.003279 | 0.024122 | 0.058531 | pred_wait_1_detect_2_form2_stage_2_cancers_10_posrisk_8_negrisk_40 | GPs | hypothetical |
| 0.008088 | 0.039314 | 0.083522 | pred_wait_1_detect_2_form2_stage_2_cancers_25_posrisk_8_negrisk_40 | GPs | hypothetical |
| 0.303987 | 0.49117 | 0.680335 | pred_wait_1_detect_2_form3_stage_1_cancers_1_posrisk_2_negrisk_1 | GPs | hypothetical |
| 0.337389 | 0.533724 | 0.725643 | pred_wait_1_detect_2_form3_stage_1_cancers_5_posrisk_2_negrisk_1 | GPs | hypothetical |
| 0.386562 | 0.590536 | 0.781397 | pred_wait_1_detect_2_form3_stage_1_cancers_10_posrisk_2_negrisk_1 | GPs | hypothetical |
| 0.56791 | 0.754595 | 0.901387 | pred_wait_1_detect_2_form3_stage_1_cancers_25_posrisk_2_negrisk_1 | GPs | hypothetical |
| 0.264107 | 0.449719 | 0.640976 | pred_wait_1_detect_2_form3_stage_1_cancers_1_posrisk_2_negrisk_5 | GPs | hypothetical |
| 0.294761 | 0.491222 | 0.689509 | pred_wait_1_detect_2_form3_stage_1_cancers_5_posrisk_2_negrisk_5 | GPs | hypothetical |
| 0.339423 | 0.547987 | 0.749008 | pred_wait_1_detect_2_form3_stage_1_cancers_10_posrisk_2_negrisk_5 | GPs | hypothetical |
| 0.519522 | 0.721161 | 0.884091 | pred_wait_1_detect_2_form3_stage_1_cancers_25_posrisk_2_negrisk_5 | GPs | hypothetical |
| 0.216659 | 0.397211 | 0.589637 | pred_wait_1_detect_2_form3_stage_1_cancers_1_posrisk_2_negrisk_10 | GPs | hypothetical |
| 0.243033 | 0.436297 | 0.63862 | pred_wait_1_detect_2_form3_stage_1_cancers_5_posrisk_2_negrisk_10 | GPs | hypothetical |
| 0.282856 | 0.491391 | 0.701892 | pred_wait_1_detect_2_form3_stage_1_cancers_10_posrisk_2_negrisk_10 | GPs | hypothetical |
| 0.45484 | 0.672423 | 0.856132 | pred_wait_1_detect_2_form3_stage_1_cancers_25_posrisk_2_negrisk_10 | GPs | hypothetical |
| 0.056062 | 0.156536 | 0.284003 | pred_wait_1_detect_2_form3_stage_1_cancers_1_posrisk_2_negrisk_40 | GPs | hypothetical |
| 0.063175 | 0.172773 | 0.312522 | pred_wait_1_detect_2_form3_stage_1_cancers_5_posrisk_2_negrisk_40 | GPs | hypothetical |
| 0.075459 | 0.198764 | 0.355387 | pred_wait_1_detect_2_form3_stage_1_cancers_10_posrisk_2_negrisk_40 | GPs | hypothetical |
| 0.148482 | 0.323884 | 0.528296 | pred_wait_1_detect_2_form3_stage_1_cancers_25_posrisk_2_negrisk_40 | GPs | hypothetical |
| 0.109547 | 0.240304 | 0.394291 | pred_wait_1_detect_2_form3_stage_1_cancers_1_posrisk_4_negrisk_1 | GPs | hypothetical |
| 0.122251 | 0.26442 | 0.429584 | pred_wait_1_detect_2_form3_stage_1_cancers_5_posrisk_4_negrisk_1 | GPs | hypothetical |
| 0.142905 | 0.300534 | 0.480386 | pred_wait_1_detect_2_form3_stage_1_cancers_10_posrisk_4_negrisk_1 | GPs | hypothetical |
| 0.250066 | 0.446788 | 0.651691 | pred_wait_1_detect_2_form3_stage_1_cancers_25_posrisk_4_negrisk_1 | GPs | hypothetical |
| 0.087428 | 0.207584 | 0.353139 | pred_wait_1_detect_2_form3_stage_1_cancers_1_posrisk_4_negrisk_5 | GPs | hypothetical |
| 0.097824 | 0.228828 | 0.385715 | pred_wait_1_detect_2_form3_stage_1_cancers_5_posrisk_4_negrisk_5 | GPs | hypothetical |
| 0.114609 | 0.261381 | 0.434085 | pred_wait_1_detect_2_form3_stage_1_cancers_10_posrisk_4_negrisk_5 | GPs | hypothetical |
| 0.209791 | 0.401397 | 0.609054 | pred_wait_1_detect_2_form3_stage_1_cancers_25_posrisk_4_negrisk_5 | GPs | hypothetical |
| 0.066127 | 0.170744 | 0.304518 | pred_wait_1_detect_2_form3_stage_1_cancers_1_posrisk_4_negrisk_10 | GPs | hypothetical |
| 0.073137 | 0.188371 | 0.332958 | pred_wait_1_detect_2_form3_stage_1_cancers_5_posrisk_4_negrisk_10 | GPs | hypothetical |
| 0.086993 | 0.216151 | 0.376289 | pred_wait_1_detect_2_form3_stage_1_cancers_10_posrisk_4_negrisk_10 | GPs | hypothetical |
| 0.16464 | 0.345036 | 0.550102 | pred_wait_1_detect_2_form3_stage_1_cancers_25_posrisk_4_negrisk_10 | GPs | hypothetical |
| 0.011934 | 0.052231 | 0.115253 | pred_wait_1_detect_2_form3_stage_1_cancers_1_posrisk_4_negrisk_40 | GPs | hypothetical |
| 0.013272 | 0.056813 | 0.124409 | pred_wait_1_detect_2_form3_stage_1_cancers_5_posrisk_4_negrisk_40 | GPs | hypothetical |
| 0.015915 | 0.064978 | 0.140378 | pred_wait_1_detect_2_form3_stage_1_cancers_10_posrisk_4_negrisk_40 | GPs | hypothetical |
| 0.036407 | 0.11642 | 0.231362 | pred_wait_1_detect_2_form3_stage_1_cancers_25_posrisk_4_negrisk_40 | GPs | hypothetical |
| 0.04662 | 0.120473 | 0.217205 | pred_wait_1_detect_2_form3_stage_1_cancers_1_posrisk_6_negrisk_1 | GPs | hypothetical |
| 0.052975 | 0.131826 | 0.233437 | pred_wait_1_detect_2_form3_stage_1_cancers_5_posrisk_6_negrisk_1 | GPs | hypothetical |
| 0.061284 | 0.14935 | 0.259542 | pred_wait_1_detect_2_form3_stage_1_cancers_10_posrisk_6_negrisk_1 | GPs | hypothetical |
| 0.109005 | 0.22944 | 0.369791 | pred_wait_1_detect_2_form3_stage_1_cancers_25_posrisk_6_negrisk_1 | GPs | hypothetical |
| 0.036111 | 0.102505 | 0.191399 | pred_wait_1_detect_2_form3_stage_1_cancers_1_posrisk_6_negrisk_5 | GPs | hypothetical |
| 0.041064 | 0.112244 | 0.20671 | pred_wait_1_detect_2_form3_stage_1_cancers_5_posrisk_6_negrisk_5 | GPs | hypothetical |
| 0.04866 | 0.127537 | 0.22989 | pred_wait_1_detect_2_form3_stage_1_cancers_10_posrisk_6_negrisk_5 | GPs | hypothetical |
| 0.088753 | 0.200367 | 0.333966 | pred_wait_1_detect_2_form3_stage_1_cancers_25_posrisk_6_negrisk_5 | GPs | hypothetical |
| 0.026075 | 0.083252 | 0.161633 | pred_wait_1_detect_2_form3_stage_1_cancers_1_posrisk_6_negrisk_10 | GPs | hypothetical |
| 0.029829 | 0.091163 | 0.176037 | pred_wait_1_detect_2_form3_stage_1_cancers_5_posrisk_6_negrisk_10 | GPs | hypothetical |
| 0.035325 | 0.103858 | 0.195946 | pred_wait_1_detect_2_form3_stage_1_cancers_10_posrisk_6_negrisk_10 | GPs | hypothetical |
| 0.068687 | 0.167477 | 0.28956 | pred_wait_1_detect_2_form3_stage_1_cancers_25_posrisk_6_negrisk_10 | GPs | hypothetical |
| 0.004109 | 0.026114 | 0.064679 | pred_wait_1_detect_2_form3_stage_1_cancers_1_posrisk_6_negrisk_40 | GPs | hypothetical |
| 0.004675 | 0.02827 | 0.068103 | pred_wait_1_detect_2_form3_stage_1_cancers_5_posrisk_6_negrisk_40 | GPs | hypothetical |
| 0.005886 | 0.032088 | 0.07588 | pred_wait_1_detect_2_form3_stage_1_cancers_10_posrisk_6_negrisk_40 | GPs | hypothetical |
| 0.013687 | 0.055808 | 0.118819 | pred_wait_1_detect_2_form3_stage_1_cancers_25_posrisk_6_negrisk_40 | GPs | hypothetical |
| 0.026531 | 0.077031 | 0.14247 | pred_wait_1_detect_2_form3_stage_1_cancers_1_posrisk_8_negrisk_1 | GPs | hypothetical |
| 0.030154 | 0.083752 | 0.151369 | pred_wait_1_detect_2_form3_stage_1_cancers_5_posrisk_8_negrisk_1 | GPs | hypothetical |
| 0.035947 | 0.093946 | 0.167053 | pred_wait_1_detect_2_form3_stage_1_cancers_10_posrisk_8_negrisk_1 | GPs | hypothetical |
| 0.062542 | 0.139674 | 0.230739 | pred_wait_1_detect_2_form3_stage_1_cancers_25_posrisk_8_negrisk_1 | GPs | hypothetical |
| 0.020422 | 0.066498 | 0.126566 | pred_wait_1_detect_2_form3_stage_1_cancers_1_posrisk_8_negrisk_5 | GPs | hypothetical |
| 0.023755 | 0.072357 | 0.134879 | pred_wait_1_detect_2_form3_stage_1_cancers_5_posrisk_8_negrisk_5 | GPs | hypothetical |
| 0.02876 | 0.081356 | 0.148523 | pred_wait_1_detect_2_form3_stage_1_cancers_10_posrisk_8_negrisk_5 | GPs | hypothetical |
| 0.052063 | 0.122804 | 0.20864 | pred_wait_1_detect_2_form3_stage_1_cancers_25_posrisk_8_negrisk_5 | GPs | hypothetical |
| 0.014617 | 0.055196 | 0.108952 | pred_wait_1_detect_2_form3_stage_1_cancers_1_posrisk_8_negrisk_10 | GPs | hypothetical |
| 0.016834 | 0.060081 | 0.117257 | pred_wait_1_detect_2_form3_stage_1_cancers_5_posrisk_8_negrisk_10 | GPs | hypothetical |
| 0.020966 | 0.067707 | 0.129194 | pred_wait_1_detect_2_form3_stage_1_cancers_10_posrisk_8_negrisk_10 | GPs | hypothetical |
| 0.040592 | 0.104061 | 0.182106 | pred_wait_1_detect_2_form3_stage_1_cancers_25_posrisk_8_negrisk_10 | GPs | hypothetical |
| 0.002123 | 0.01975 | 0.050845 | pred_wait_1_detect_2_form3_stage_1_cancers_1_posrisk_8_negrisk_40 | GPs | hypothetical |
| 0.002481 | 0.021388 | 0.053261 | pred_wait_1_detect_2_form3_stage_1_cancers_5_posrisk_8_negrisk_40 | GPs | hypothetical |
| 0.003279 | 0.024122 | 0.058531 | pred_wait_1_detect_2_form3_stage_1_cancers_10_posrisk_8_negrisk_40 | GPs | hypothetical |
| 0.008088 | 0.039314 | 0.083522 | pred_wait_1_detect_2_form3_stage_1_cancers_25_posrisk_8_negrisk_40 | GPs | hypothetical |
| 0.303987 | 0.49117 | 0.680335 | pred_wait_1_detect_2_form3_stage_2_cancers_1_posrisk_2_negrisk_1 | GPs | hypothetical |
| 0.337389 | 0.533724 | 0.725643 | pred_wait_1_detect_2_form3_stage_2_cancers_5_posrisk_2_negrisk_1 | GPs | hypothetical |
| 0.386562 | 0.590536 | 0.781397 | pred_wait_1_detect_2_form3_stage_2_cancers_10_posrisk_2_negrisk_1 | GPs | hypothetical |
| 0.56791 | 0.754595 | 0.901387 | pred_wait_1_detect_2_form3_stage_2_cancers_25_posrisk_2_negrisk_1 | GPs | hypothetical |
| 0.264107 | 0.449719 | 0.640976 | pred_wait_1_detect_2_form3_stage_2_cancers_1_posrisk_2_negrisk_5 | GPs | hypothetical |
| 0.294761 | 0.491222 | 0.689509 | pred_wait_1_detect_2_form3_stage_2_cancers_5_posrisk_2_negrisk_5 | GPs | hypothetical |
| 0.339423 | 0.547987 | 0.749008 | pred_wait_1_detect_2_form3_stage_2_cancers_10_posrisk_2_negrisk_5 | GPs | hypothetical |
| 0.519522 | 0.721161 | 0.884091 | pred_wait_1_detect_2_form3_stage_2_cancers_25_posrisk_2_negrisk_5 | GPs | hypothetical |
| 0.216659 | 0.397211 | 0.589637 | pred_wait_1_detect_2_form3_stage_2_cancers_1_posrisk_2_negrisk_10 | GPs | hypothetical |
| 0.243033 | 0.436297 | 0.63862 | pred_wait_1_detect_2_form3_stage_2_cancers_5_posrisk_2_negrisk_10 | GPs | hypothetical |
| 0.282856 | 0.491391 | 0.701892 | pred_wait_1_detect_2_form3_stage_2_cancers_10_posrisk_2_negrisk_10 | GPs | hypothetical |
| 0.45484 | 0.672423 | 0.856132 | pred_wait_1_detect_2_form3_stage_2_cancers_25_posrisk_2_negrisk_10 | GPs | hypothetical |
| 0.056062 | 0.156536 | 0.284003 | pred_wait_1_detect_2_form3_stage_2_cancers_1_posrisk_2_negrisk_40 | GPs | hypothetical |
| 0.063175 | 0.172773 | 0.312522 | pred_wait_1_detect_2_form3_stage_2_cancers_5_posrisk_2_negrisk_40 | GPs | hypothetical |
| 0.075459 | 0.198764 | 0.355387 | pred_wait_1_detect_2_form3_stage_2_cancers_10_posrisk_2_negrisk_40 | GPs | hypothetical |
| 0.148482 | 0.323884 | 0.528296 | pred_wait_1_detect_2_form3_stage_2_cancers_25_posrisk_2_negrisk_40 | GPs | hypothetical |
| 0.109547 | 0.240304 | 0.394291 | pred_wait_1_detect_2_form3_stage_2_cancers_1_posrisk_4_negrisk_1 | GPs | hypothetical |
| 0.122251 | 0.26442 | 0.429584 | pred_wait_1_detect_2_form3_stage_2_cancers_5_posrisk_4_negrisk_1 | GPs | hypothetical |
| 0.142905 | 0.300534 | 0.480386 | pred_wait_1_detect_2_form3_stage_2_cancers_10_posrisk_4_negrisk_1 | GPs | hypothetical |
| 0.250066 | 0.446788 | 0.651691 | pred_wait_1_detect_2_form3_stage_2_cancers_25_posrisk_4_negrisk_1 | GPs | hypothetical |
| 0.087428 | 0.207584 | 0.353139 | pred_wait_1_detect_2_form3_stage_2_cancers_1_posrisk_4_negrisk_5 | GPs | hypothetical |
| 0.097824 | 0.228828 | 0.385715 | pred_wait_1_detect_2_form3_stage_2_cancers_5_posrisk_4_negrisk_5 | GPs | hypothetical |
| 0.114609 | 0.261381 | 0.434085 | pred_wait_1_detect_2_form3_stage_2_cancers_10_posrisk_4_negrisk_5 | GPs | hypothetical |
| 0.209791 | 0.401397 | 0.609054 | pred_wait_1_detect_2_form3_stage_2_cancers_25_posrisk_4_negrisk_5 | GPs | hypothetical |
| 0.066127 | 0.170744 | 0.304518 | pred_wait_1_detect_2_form3_stage_2_cancers_1_posrisk_4_negrisk_10 | GPs | hypothetical |
| 0.073137 | 0.188371 | 0.332958 | pred_wait_1_detect_2_form3_stage_2_cancers_5_posrisk_4_negrisk_10 | GPs | hypothetical |
| 0.086993 | 0.216151 | 0.376289 | pred_wait_1_detect_2_form3_stage_2_cancers_10_posrisk_4_negrisk_10 | GPs | hypothetical |
| 0.16464 | 0.345036 | 0.550102 | pred_wait_1_detect_2_form3_stage_2_cancers_25_posrisk_4_negrisk_10 | GPs | hypothetical |
| 0.011934 | 0.052231 | 0.115253 | pred_wait_1_detect_2_form3_stage_2_cancers_1_posrisk_4_negrisk_40 | GPs | hypothetical |
| 0.013272 | 0.056813 | 0.124409 | pred_wait_1_detect_2_form3_stage_2_cancers_5_posrisk_4_negrisk_40 | GPs | hypothetical |
| 0.015915 | 0.064978 | 0.140378 | pred_wait_1_detect_2_form3_stage_2_cancers_10_posrisk_4_negrisk_40 | GPs | hypothetical |
| 0.036407 | 0.11642 | 0.231362 | pred_wait_1_detect_2_form3_stage_2_cancers_25_posrisk_4_negrisk_40 | GPs | hypothetical |
| 0.04662 | 0.120473 | 0.217205 | pred_wait_1_detect_2_form3_stage_2_cancers_1_posrisk_6_negrisk_1 | GPs | hypothetical |
| 0.052975 | 0.131826 | 0.233437 | pred_wait_1_detect_2_form3_stage_2_cancers_5_posrisk_6_negrisk_1 | GPs | hypothetical |
| 0.061284 | 0.14935 | 0.259542 | pred_wait_1_detect_2_form3_stage_2_cancers_10_posrisk_6_negrisk_1 | GPs | hypothetical |
| 0.109005 | 0.22944 | 0.369791 | pred_wait_1_detect_2_form3_stage_2_cancers_25_posrisk_6_negrisk_1 | GPs | hypothetical |
| 0.036111 | 0.102505 | 0.191399 | pred_wait_1_detect_2_form3_stage_2_cancers_1_posrisk_6_negrisk_5 | GPs | hypothetical |
| 0.041064 | 0.112244 | 0.20671 | pred_wait_1_detect_2_form3_stage_2_cancers_5_posrisk_6_negrisk_5 | GPs | hypothetical |
| 0.04866 | 0.127537 | 0.22989 | pred_wait_1_detect_2_form3_stage_2_cancers_10_posrisk_6_negrisk_5 | GPs | hypothetical |
| 0.088753 | 0.200367 | 0.333966 | pred_wait_1_detect_2_form3_stage_2_cancers_25_posrisk_6_negrisk_5 | GPs | hypothetical |
| 0.026075 | 0.083252 | 0.161633 | pred_wait_1_detect_2_form3_stage_2_cancers_1_posrisk_6_negrisk_10 | GPs | hypothetical |
| 0.029829 | 0.091163 | 0.176037 | pred_wait_1_detect_2_form3_stage_2_cancers_5_posrisk_6_negrisk_10 | GPs | hypothetical |
| 0.035325 | 0.103858 | 0.195946 | pred_wait_1_detect_2_form3_stage_2_cancers_10_posrisk_6_negrisk_10 | GPs | hypothetical |
| 0.068687 | 0.167477 | 0.28956 | pred_wait_1_detect_2_form3_stage_2_cancers_25_posrisk_6_negrisk_10 | GPs | hypothetical |
| 0.004109 | 0.026114 | 0.064679 | pred_wait_1_detect_2_form3_stage_2_cancers_1_posrisk_6_negrisk_40 | GPs | hypothetical |
| 0.004675 | 0.02827 | 0.068103 | pred_wait_1_detect_2_form3_stage_2_cancers_5_posrisk_6_negrisk_40 | GPs | hypothetical |
| 0.005886 | 0.032088 | 0.07588 | pred_wait_1_detect_2_form3_stage_2_cancers_10_posrisk_6_negrisk_40 | GPs | hypothetical |
| 0.013687 | 0.055808 | 0.118819 | pred_wait_1_detect_2_form3_stage_2_cancers_25_posrisk_6_negrisk_40 | GPs | hypothetical |
| 0.026531 | 0.077031 | 0.14247 | pred_wait_1_detect_2_form3_stage_2_cancers_1_posrisk_8_negrisk_1 | GPs | hypothetical |
| 0.030154 | 0.083752 | 0.151369 | pred_wait_1_detect_2_form3_stage_2_cancers_5_posrisk_8_negrisk_1 | GPs | hypothetical |
| 0.035947 | 0.093946 | 0.167053 | pred_wait_1_detect_2_form3_stage_2_cancers_10_posrisk_8_negrisk_1 | GPs | hypothetical |
| 0.062542 | 0.139674 | 0.230739 | pred_wait_1_detect_2_form3_stage_2_cancers_25_posrisk_8_negrisk_1 | GPs | hypothetical |
| 0.020422 | 0.066498 | 0.126566 | pred_wait_1_detect_2_form3_stage_2_cancers_1_posrisk_8_negrisk_5 | GPs | hypothetical |
| 0.023755 | 0.072357 | 0.134879 | pred_wait_1_detect_2_form3_stage_2_cancers_5_posrisk_8_negrisk_5 | GPs | hypothetical |
| 0.02876 | 0.081356 | 0.148523 | pred_wait_1_detect_2_form3_stage_2_cancers_10_posrisk_8_negrisk_5 | GPs | hypothetical |
| 0.052063 | 0.122804 | 0.20864 | pred_wait_1_detect_2_form3_stage_2_cancers_25_posrisk_8_negrisk_5 | GPs | hypothetical |
| 0.014617 | 0.055196 | 0.108952 | pred_wait_1_detect_2_form3_stage_2_cancers_1_posrisk_8_negrisk_10 | GPs | hypothetical |
| 0.016834 | 0.060081 | 0.117257 | pred_wait_1_detect_2_form3_stage_2_cancers_5_posrisk_8_negrisk_10 | GPs | hypothetical |
| 0.020966 | 0.067707 | 0.129194 | pred_wait_1_detect_2_form3_stage_2_cancers_10_posrisk_8_negrisk_10 | GPs | hypothetical |
| 0.040592 | 0.104061 | 0.182106 | pred_wait_1_detect_2_form3_stage_2_cancers_25_posrisk_8_negrisk_10 | GPs | hypothetical |
| 0.002123 | 0.01975 | 0.050845 | pred_wait_1_detect_2_form3_stage_2_cancers_1_posrisk_8_negrisk_40 | GPs | hypothetical |
| 0.002481 | 0.021388 | 0.053261 | pred_wait_1_detect_2_form3_stage_2_cancers_5_posrisk_8_negrisk_40 | GPs | hypothetical |
| 0.003279 | 0.024122 | 0.058531 | pred_wait_1_detect_2_form3_stage_2_cancers_10_posrisk_8_negrisk_40 | GPs | hypothetical |
| 0.008088 | 0.039314 | 0.083522 | pred_wait_1_detect_2_form3_stage_2_cancers_25_posrisk_8_negrisk_40 | GPs | hypothetical |
| 0.303987 | 0.49117 | 0.680335 | pred_wait_1_detect_2_form4_stage_1_cancers_1_posrisk_2_negrisk_1 | GPs | hypothetical |
| 0.337389 | 0.533724 | 0.725643 | pred_wait_1_detect_2_form4_stage_1_cancers_5_posrisk_2_negrisk_1 | GPs | hypothetical |
| 0.386562 | 0.590536 | 0.781397 | pred_wait_1_detect_2_form4_stage_1_cancers_10_posrisk_2_negrisk_1 | GPs | hypothetical |
| 0.56791 | 0.754595 | 0.901387 | pred_wait_1_detect_2_form4_stage_1_cancers_25_posrisk_2_negrisk_1 | GPs | hypothetical |
| 0.264107 | 0.449719 | 0.640976 | pred_wait_1_detect_2_form4_stage_1_cancers_1_posrisk_2_negrisk_5 | GPs | hypothetical |
| 0.294761 | 0.491222 | 0.689509 | pred_wait_1_detect_2_form4_stage_1_cancers_5_posrisk_2_negrisk_5 | GPs | hypothetical |
| 0.339423 | 0.547987 | 0.749008 | pred_wait_1_detect_2_form4_stage_1_cancers_10_posrisk_2_negrisk_5 | GPs | hypothetical |
| 0.519522 | 0.721161 | 0.884091 | pred_wait_1_detect_2_form4_stage_1_cancers_25_posrisk_2_negrisk_5 | GPs | hypothetical |
| 0.216659 | 0.397211 | 0.589637 | pred_wait_1_detect_2_form4_stage_1_cancers_1_posrisk_2_negrisk_10 | GPs | hypothetical |
| 0.243033 | 0.436297 | 0.63862 | pred_wait_1_detect_2_form4_stage_1_cancers_5_posrisk_2_negrisk_10 | GPs | hypothetical |
| 0.282856 | 0.491391 | 0.701892 | pred_wait_1_detect_2_form4_stage_1_cancers_10_posrisk_2_negrisk_10 | GPs | hypothetical |
| 0.45484 | 0.672423 | 0.856132 | pred_wait_1_detect_2_form4_stage_1_cancers_25_posrisk_2_negrisk_10 | GPs | hypothetical |
| 0.056062 | 0.156536 | 0.284003 | pred_wait_1_detect_2_form4_stage_1_cancers_1_posrisk_2_negrisk_40 | GPs | hypothetical |
| 0.063175 | 0.172773 | 0.312522 | pred_wait_1_detect_2_form4_stage_1_cancers_5_posrisk_2_negrisk_40 | GPs | hypothetical |
| 0.075459 | 0.198764 | 0.355387 | pred_wait_1_detect_2_form4_stage_1_cancers_10_posrisk_2_negrisk_40 | GPs | hypothetical |
| 0.148482 | 0.323884 | 0.528296 | pred_wait_1_detect_2_form4_stage_1_cancers_25_posrisk_2_negrisk_40 | GPs | hypothetical |
| 0.109547 | 0.240304 | 0.394291 | pred_wait_1_detect_2_form4_stage_1_cancers_1_posrisk_4_negrisk_1 | GPs | hypothetical |
| 0.122251 | 0.26442 | 0.429584 | pred_wait_1_detect_2_form4_stage_1_cancers_5_posrisk_4_negrisk_1 | GPs | hypothetical |
| 0.142905 | 0.300534 | 0.480386 | pred_wait_1_detect_2_form4_stage_1_cancers_10_posrisk_4_negrisk_1 | GPs | hypothetical |
| 0.250066 | 0.446788 | 0.651691 | pred_wait_1_detect_2_form4_stage_1_cancers_25_posrisk_4_negrisk_1 | GPs | hypothetical |
| 0.087428 | 0.207584 | 0.353139 | pred_wait_1_detect_2_form4_stage_1_cancers_1_posrisk_4_negrisk_5 | GPs | hypothetical |
| 0.097824 | 0.228828 | 0.385715 | pred_wait_1_detect_2_form4_stage_1_cancers_5_posrisk_4_negrisk_5 | GPs | hypothetical |
| 0.114609 | 0.261381 | 0.434085 | pred_wait_1_detect_2_form4_stage_1_cancers_10_posrisk_4_negrisk_5 | GPs | hypothetical |
| 0.209791 | 0.401397 | 0.609054 | pred_wait_1_detect_2_form4_stage_1_cancers_25_posrisk_4_negrisk_5 | GPs | hypothetical |
| 0.066127 | 0.170744 | 0.304518 | pred_wait_1_detect_2_form4_stage_1_cancers_1_posrisk_4_negrisk_10 | GPs | hypothetical |
| 0.073137 | 0.188371 | 0.332958 | pred_wait_1_detect_2_form4_stage_1_cancers_5_posrisk_4_negrisk_10 | GPs | hypothetical |
| 0.086993 | 0.216151 | 0.376289 | pred_wait_1_detect_2_form4_stage_1_cancers_10_posrisk_4_negrisk_10 | GPs | hypothetical |
| 0.16464 | 0.345036 | 0.550102 | pred_wait_1_detect_2_form4_stage_1_cancers_25_posrisk_4_negrisk_10 | GPs | hypothetical |
| 0.011934 | 0.052231 | 0.115253 | pred_wait_1_detect_2_form4_stage_1_cancers_1_posrisk_4_negrisk_40 | GPs | hypothetical |
| 0.013272 | 0.056813 | 0.124409 | pred_wait_1_detect_2_form4_stage_1_cancers_5_posrisk_4_negrisk_40 | GPs | hypothetical |
| 0.015915 | 0.064978 | 0.140378 | pred_wait_1_detect_2_form4_stage_1_cancers_10_posrisk_4_negrisk_40 | GPs | hypothetical |
| 0.036407 | 0.11642 | 0.231362 | pred_wait_1_detect_2_form4_stage_1_cancers_25_posrisk_4_negrisk_40 | GPs | hypothetical |
| 0.04662 | 0.120473 | 0.217205 | pred_wait_1_detect_2_form4_stage_1_cancers_1_posrisk_6_negrisk_1 | GPs | hypothetical |
| 0.052975 | 0.131826 | 0.233437 | pred_wait_1_detect_2_form4_stage_1_cancers_5_posrisk_6_negrisk_1 | GPs | hypothetical |
| 0.061284 | 0.14935 | 0.259542 | pred_wait_1_detect_2_form4_stage_1_cancers_10_posrisk_6_negrisk_1 | GPs | hypothetical |
| 0.109005 | 0.22944 | 0.369791 | pred_wait_1_detect_2_form4_stage_1_cancers_25_posrisk_6_negrisk_1 | GPs | hypothetical |
| 0.036111 | 0.102505 | 0.191399 | pred_wait_1_detect_2_form4_stage_1_cancers_1_posrisk_6_negrisk_5 | GPs | hypothetical |
| 0.041064 | 0.112244 | 0.20671 | pred_wait_1_detect_2_form4_stage_1_cancers_5_posrisk_6_negrisk_5 | GPs | hypothetical |
| 0.04866 | 0.127537 | 0.22989 | pred_wait_1_detect_2_form4_stage_1_cancers_10_posrisk_6_negrisk_5 | GPs | hypothetical |
| 0.088753 | 0.200367 | 0.333966 | pred_wait_1_detect_2_form4_stage_1_cancers_25_posrisk_6_negrisk_5 | GPs | hypothetical |
| 0.026075 | 0.083252 | 0.161633 | pred_wait_1_detect_2_form4_stage_1_cancers_1_posrisk_6_negrisk_10 | GPs | hypothetical |
| 0.029829 | 0.091163 | 0.176037 | pred_wait_1_detect_2_form4_stage_1_cancers_5_posrisk_6_negrisk_10 | GPs | hypothetical |
| 0.035325 | 0.103858 | 0.195946 | pred_wait_1_detect_2_form4_stage_1_cancers_10_posrisk_6_negrisk_10 | GPs | hypothetical |
| 0.068687 | 0.167477 | 0.28956 | pred_wait_1_detect_2_form4_stage_1_cancers_25_posrisk_6_negrisk_10 | GPs | hypothetical |
| 0.004109 | 0.026114 | 0.064679 | pred_wait_1_detect_2_form4_stage_1_cancers_1_posrisk_6_negrisk_40 | GPs | hypothetical |
| 0.004675 | 0.02827 | 0.068103 | pred_wait_1_detect_2_form4_stage_1_cancers_5_posrisk_6_negrisk_40 | GPs | hypothetical |
| 0.005886 | 0.032088 | 0.07588 | pred_wait_1_detect_2_form4_stage_1_cancers_10_posrisk_6_negrisk_40 | GPs | hypothetical |
| 0.013687 | 0.055808 | 0.118819 | pred_wait_1_detect_2_form4_stage_1_cancers_25_posrisk_6_negrisk_40 | GPs | hypothetical |
| 0.026531 | 0.077031 | 0.14247 | pred_wait_1_detect_2_form4_stage_1_cancers_1_posrisk_8_negrisk_1 | GPs | hypothetical |
| 0.030154 | 0.083752 | 0.151369 | pred_wait_1_detect_2_form4_stage_1_cancers_5_posrisk_8_negrisk_1 | GPs | hypothetical |
| 0.035947 | 0.093946 | 0.167053 | pred_wait_1_detect_2_form4_stage_1_cancers_10_posrisk_8_negrisk_1 | GPs | hypothetical |
| 0.062542 | 0.139674 | 0.230739 | pred_wait_1_detect_2_form4_stage_1_cancers_25_posrisk_8_negrisk_1 | GPs | hypothetical |
| 0.020422 | 0.066498 | 0.126566 | pred_wait_1_detect_2_form4_stage_1_cancers_1_posrisk_8_negrisk_5 | GPs | hypothetical |
| 0.023755 | 0.072357 | 0.134879 | pred_wait_1_detect_2_form4_stage_1_cancers_5_posrisk_8_negrisk_5 | GPs | hypothetical |
| 0.02876 | 0.081356 | 0.148523 | pred_wait_1_detect_2_form4_stage_1_cancers_10_posrisk_8_negrisk_5 | GPs | hypothetical |
| 0.052063 | 0.122804 | 0.20864 | pred_wait_1_detect_2_form4_stage_1_cancers_25_posrisk_8_negrisk_5 | GPs | hypothetical |
| 0.014617 | 0.055196 | 0.108952 | pred_wait_1_detect_2_form4_stage_1_cancers_1_posrisk_8_negrisk_10 | GPs | hypothetical |
| 0.016834 | 0.060081 | 0.117257 | pred_wait_1_detect_2_form4_stage_1_cancers_5_posrisk_8_negrisk_10 | GPs | hypothetical |
| 0.020966 | 0.067707 | 0.129194 | pred_wait_1_detect_2_form4_stage_1_cancers_10_posrisk_8_negrisk_10 | GPs | hypothetical |
| 0.040592 | 0.104061 | 0.182106 | pred_wait_1_detect_2_form4_stage_1_cancers_25_posrisk_8_negrisk_10 | GPs | hypothetical |
| 0.002123 | 0.01975 | 0.050845 | pred_wait_1_detect_2_form4_stage_1_cancers_1_posrisk_8_negrisk_40 | GPs | hypothetical |
| 0.002481 | 0.021388 | 0.053261 | pred_wait_1_detect_2_form4_stage_1_cancers_5_posrisk_8_negrisk_40 | GPs | hypothetical |
| 0.003279 | 0.024122 | 0.058531 | pred_wait_1_detect_2_form4_stage_1_cancers_10_posrisk_8_negrisk_40 | GPs | hypothetical |
| 0.008088 | 0.039314 | 0.083522 | pred_wait_1_detect_2_form4_stage_1_cancers_25_posrisk_8_negrisk_40 | GPs | hypothetical |
| 0.303987 | 0.49117 | 0.680335 | pred_wait_1_detect_2_form4_stage_2_cancers_1_posrisk_2_negrisk_1 | GPs | hypothetical |
| 0.337389 | 0.533724 | 0.725643 | pred_wait_1_detect_2_form4_stage_2_cancers_5_posrisk_2_negrisk_1 | GPs | hypothetical |
| 0.386562 | 0.590536 | 0.781397 | pred_wait_1_detect_2_form4_stage_2_cancers_10_posrisk_2_negrisk_1 | GPs | hypothetical |
| 0.56791 | 0.754595 | 0.901387 | pred_wait_1_detect_2_form4_stage_2_cancers_25_posrisk_2_negrisk_1 | GPs | hypothetical |
| 0.264107 | 0.449719 | 0.640976 | pred_wait_1_detect_2_form4_stage_2_cancers_1_posrisk_2_negrisk_5 | GPs | hypothetical |
| 0.294761 | 0.491222 | 0.689509 | pred_wait_1_detect_2_form4_stage_2_cancers_5_posrisk_2_negrisk_5 | GPs | hypothetical |
| 0.339423 | 0.547987 | 0.749008 | pred_wait_1_detect_2_form4_stage_2_cancers_10_posrisk_2_negrisk_5 | GPs | hypothetical |
| 0.519522 | 0.721161 | 0.884091 | pred_wait_1_detect_2_form4_stage_2_cancers_25_posrisk_2_negrisk_5 | GPs | hypothetical |
| 0.216659 | 0.397211 | 0.589637 | pred_wait_1_detect_2_form4_stage_2_cancers_1_posrisk_2_negrisk_10 | GPs | hypothetical |
| 0.243033 | 0.436297 | 0.63862 | pred_wait_1_detect_2_form4_stage_2_cancers_5_posrisk_2_negrisk_10 | GPs | hypothetical |
| 0.282856 | 0.491391 | 0.701892 | pred_wait_1_detect_2_form4_stage_2_cancers_10_posrisk_2_negrisk_10 | GPs | hypothetical |
| 0.45484 | 0.672423 | 0.856132 | pred_wait_1_detect_2_form4_stage_2_cancers_25_posrisk_2_negrisk_10 | GPs | hypothetical |
| 0.056062 | 0.156536 | 0.284003 | pred_wait_1_detect_2_form4_stage_2_cancers_1_posrisk_2_negrisk_40 | GPs | hypothetical |
| 0.063175 | 0.172773 | 0.312522 | pred_wait_1_detect_2_form4_stage_2_cancers_5_posrisk_2_negrisk_40 | GPs | hypothetical |
| 0.075459 | 0.198764 | 0.355387 | pred_wait_1_detect_2_form4_stage_2_cancers_10_posrisk_2_negrisk_40 | GPs | hypothetical |
| 0.148482 | 0.323884 | 0.528296 | pred_wait_1_detect_2_form4_stage_2_cancers_25_posrisk_2_negrisk_40 | GPs | hypothetical |
| 0.109547 | 0.240304 | 0.394291 | pred_wait_1_detect_2_form4_stage_2_cancers_1_posrisk_4_negrisk_1 | GPs | hypothetical |
| 0.122251 | 0.26442 | 0.429584 | pred_wait_1_detect_2_form4_stage_2_cancers_5_posrisk_4_negrisk_1 | GPs | hypothetical |
| 0.142905 | 0.300534 | 0.480386 | pred_wait_1_detect_2_form4_stage_2_cancers_10_posrisk_4_negrisk_1 | GPs | hypothetical |
| 0.250066 | 0.446788 | 0.651691 | pred_wait_1_detect_2_form4_stage_2_cancers_25_posrisk_4_negrisk_1 | GPs | hypothetical |
| 0.087428 | 0.207584 | 0.353139 | pred_wait_1_detect_2_form4_stage_2_cancers_1_posrisk_4_negrisk_5 | GPs | hypothetical |
| 0.097824 | 0.228828 | 0.385715 | pred_wait_1_detect_2_form4_stage_2_cancers_5_posrisk_4_negrisk_5 | GPs | hypothetical |
| 0.114609 | 0.261381 | 0.434085 | pred_wait_1_detect_2_form4_stage_2_cancers_10_posrisk_4_negrisk_5 | GPs | hypothetical |
| 0.209791 | 0.401397 | 0.609054 | pred_wait_1_detect_2_form4_stage_2_cancers_25_posrisk_4_negrisk_5 | GPs | hypothetical |
| 0.066127 | 0.170744 | 0.304518 | pred_wait_1_detect_2_form4_stage_2_cancers_1_posrisk_4_negrisk_10 | GPs | hypothetical |
| 0.073137 | 0.188371 | 0.332958 | pred_wait_1_detect_2_form4_stage_2_cancers_5_posrisk_4_negrisk_10 | GPs | hypothetical |
| 0.086993 | 0.216151 | 0.376289 | pred_wait_1_detect_2_form4_stage_2_cancers_10_posrisk_4_negrisk_10 | GPs | hypothetical |
| 0.16464 | 0.345036 | 0.550102 | pred_wait_1_detect_2_form4_stage_2_cancers_25_posrisk_4_negrisk_10 | GPs | hypothetical |
| 0.011934 | 0.052231 | 0.115253 | pred_wait_1_detect_2_form4_stage_2_cancers_1_posrisk_4_negrisk_40 | GPs | hypothetical |
| 0.013272 | 0.056813 | 0.124409 | pred_wait_1_detect_2_form4_stage_2_cancers_5_posrisk_4_negrisk_40 | GPs | hypothetical |
| 0.015915 | 0.064978 | 0.140378 | pred_wait_1_detect_2_form4_stage_2_cancers_10_posrisk_4_negrisk_40 | GPs | hypothetical |
| 0.036407 | 0.11642 | 0.231362 | pred_wait_1_detect_2_form4_stage_2_cancers_25_posrisk_4_negrisk_40 | GPs | hypothetical |
| 0.04662 | 0.120473 | 0.217205 | pred_wait_1_detect_2_form4_stage_2_cancers_1_posrisk_6_negrisk_1 | GPs | hypothetical |
| 0.052975 | 0.131826 | 0.233437 | pred_wait_1_detect_2_form4_stage_2_cancers_5_posrisk_6_negrisk_1 | GPs | hypothetical |
| 0.061284 | 0.14935 | 0.259542 | pred_wait_1_detect_2_form4_stage_2_cancers_10_posrisk_6_negrisk_1 | GPs | hypothetical |
| 0.109005 | 0.22944 | 0.369791 | pred_wait_1_detect_2_form4_stage_2_cancers_25_posrisk_6_negrisk_1 | GPs | hypothetical |
| 0.036111 | 0.102505 | 0.191399 | pred_wait_1_detect_2_form4_stage_2_cancers_1_posrisk_6_negrisk_5 | GPs | hypothetical |
| 0.041064 | 0.112244 | 0.20671 | pred_wait_1_detect_2_form4_stage_2_cancers_5_posrisk_6_negrisk_5 | GPs | hypothetical |
| 0.04866 | 0.127537 | 0.22989 | pred_wait_1_detect_2_form4_stage_2_cancers_10_posrisk_6_negrisk_5 | GPs | hypothetical |
| 0.088753 | 0.200367 | 0.333966 | pred_wait_1_detect_2_form4_stage_2_cancers_25_posrisk_6_negrisk_5 | GPs | hypothetical |
| 0.026075 | 0.083252 | 0.161633 | pred_wait_1_detect_2_form4_stage_2_cancers_1_posrisk_6_negrisk_10 | GPs | hypothetical |
| 0.029829 | 0.091163 | 0.176037 | pred_wait_1_detect_2_form4_stage_2_cancers_5_posrisk_6_negrisk_10 | GPs | hypothetical |
| 0.035325 | 0.103858 | 0.195946 | pred_wait_1_detect_2_form4_stage_2_cancers_10_posrisk_6_negrisk_10 | GPs | hypothetical |
| 0.068687 | 0.167477 | 0.28956 | pred_wait_1_detect_2_form4_stage_2_cancers_25_posrisk_6_negrisk_10 | GPs | hypothetical |
| 0.004109 | 0.026114 | 0.064679 | pred_wait_1_detect_2_form4_stage_2_cancers_1_posrisk_6_negrisk_40 | GPs | hypothetical |
| 0.004675 | 0.02827 | 0.068103 | pred_wait_1_detect_2_form4_stage_2_cancers_5_posrisk_6_negrisk_40 | GPs | hypothetical |
| 0.005886 | 0.032088 | 0.07588 | pred_wait_1_detect_2_form4_stage_2_cancers_10_posrisk_6_negrisk_40 | GPs | hypothetical |
| 0.013687 | 0.055808 | 0.118819 | pred_wait_1_detect_2_form4_stage_2_cancers_25_posrisk_6_negrisk_40 | GPs | hypothetical |
| 0.026531 | 0.077031 | 0.14247 | pred_wait_1_detect_2_form4_stage_2_cancers_1_posrisk_8_negrisk_1 | GPs | hypothetical |
| 0.030154 | 0.083752 | 0.151369 | pred_wait_1_detect_2_form4_stage_2_cancers_5_posrisk_8_negrisk_1 | GPs | hypothetical |
| 0.035947 | 0.093946 | 0.167053 | pred_wait_1_detect_2_form4_stage_2_cancers_10_posrisk_8_negrisk_1 | GPs | hypothetical |
| 0.062542 | 0.139674 | 0.230739 | pred_wait_1_detect_2_form4_stage_2_cancers_25_posrisk_8_negrisk_1 | GPs | hypothetical |
| 0.020422 | 0.066498 | 0.126566 | pred_wait_1_detect_2_form4_stage_2_cancers_1_posrisk_8_negrisk_5 | GPs | hypothetical |
| 0.023755 | 0.072357 | 0.134879 | pred_wait_1_detect_2_form4_stage_2_cancers_5_posrisk_8_negrisk_5 | GPs | hypothetical |
| 0.02876 | 0.081356 | 0.148523 | pred_wait_1_detect_2_form4_stage_2_cancers_10_posrisk_8_negrisk_5 | GPs | hypothetical |
| 0.052063 | 0.122804 | 0.20864 | pred_wait_1_detect_2_form4_stage_2_cancers_25_posrisk_8_negrisk_5 | GPs | hypothetical |
| 0.014617 | 0.055196 | 0.108952 | pred_wait_1_detect_2_form4_stage_2_cancers_1_posrisk_8_negrisk_10 | GPs | hypothetical |
| 0.016834 | 0.060081 | 0.117257 | pred_wait_1_detect_2_form4_stage_2_cancers_5_posrisk_8_negrisk_10 | GPs | hypothetical |
| 0.020966 | 0.067707 | 0.129194 | pred_wait_1_detect_2_form4_stage_2_cancers_10_posrisk_8_negrisk_10 | GPs | hypothetical |
| 0.040592 | 0.104061 | 0.182106 | pred_wait_1_detect_2_form4_stage_2_cancers_25_posrisk_8_negrisk_10 | GPs | hypothetical |
| 0.002123 | 0.01975 | 0.050845 | pred_wait_1_detect_2_form4_stage_2_cancers_1_posrisk_8_negrisk_40 | GPs | hypothetical |
| 0.002481 | 0.021388 | 0.053261 | pred_wait_1_detect_2_form4_stage_2_cancers_5_posrisk_8_negrisk_40 | GPs | hypothetical |
| 0.003279 | 0.024122 | 0.058531 | pred_wait_1_detect_2_form4_stage_2_cancers_10_posrisk_8_negrisk_40 | GPs | hypothetical |
| 0.008088 | 0.039314 | 0.083522 | pred_wait_1_detect_2_form4_stage_2_cancers_25_posrisk_8_negrisk_40 | GPs | hypothetical |
| 0.303987 | 0.49117 | 0.680335 | pred_wait_2_detect_1_form1_stage_1_cancers_1_posrisk_2_negrisk_1 | GPs | hypothetical |
| 0.337389 | 0.533724 | 0.725643 | pred_wait_2_detect_1_form1_stage_1_cancers_5_posrisk_2_negrisk_1 | GPs | hypothetical |
| 0.386562 | 0.590536 | 0.781397 | pred_wait_2_detect_1_form1_stage_1_cancers_10_posrisk_2_negrisk_1 | GPs | hypothetical |
| 0.56791 | 0.754595 | 0.901387 | pred_wait_2_detect_1_form1_stage_1_cancers_25_posrisk_2_negrisk_1 | GPs | hypothetical |
| 0.264107 | 0.449719 | 0.640976 | pred_wait_2_detect_1_form1_stage_1_cancers_1_posrisk_2_negrisk_5 | GPs | hypothetical |
| 0.294761 | 0.491222 | 0.689509 | pred_wait_2_detect_1_form1_stage_1_cancers_5_posrisk_2_negrisk_5 | GPs | hypothetical |
| 0.339423 | 0.547987 | 0.749008 | pred_wait_2_detect_1_form1_stage_1_cancers_10_posrisk_2_negrisk_5 | GPs | hypothetical |
| 0.519522 | 0.721161 | 0.884091 | pred_wait_2_detect_1_form1_stage_1_cancers_25_posrisk_2_negrisk_5 | GPs | hypothetical |
| 0.216659 | 0.397211 | 0.589637 | pred_wait_2_detect_1_form1_stage_1_cancers_1_posrisk_2_negrisk_10 | GPs | hypothetical |
| 0.243033 | 0.436297 | 0.63862 | pred_wait_2_detect_1_form1_stage_1_cancers_5_posrisk_2_negrisk_10 | GPs | hypothetical |
| 0.282856 | 0.491391 | 0.701892 | pred_wait_2_detect_1_form1_stage_1_cancers_10_posrisk_2_negrisk_10 | GPs | hypothetical |
| 0.45484 | 0.672423 | 0.856132 | pred_wait_2_detect_1_form1_stage_1_cancers_25_posrisk_2_negrisk_10 | GPs | hypothetical |
| 0.056062 | 0.156536 | 0.284003 | pred_wait_2_detect_1_form1_stage_1_cancers_1_posrisk_2_negrisk_40 | GPs | hypothetical |
| 0.063175 | 0.172773 | 0.312522 | pred_wait_2_detect_1_form1_stage_1_cancers_5_posrisk_2_negrisk_40 | GPs | hypothetical |
| 0.075459 | 0.198764 | 0.355387 | pred_wait_2_detect_1_form1_stage_1_cancers_10_posrisk_2_negrisk_40 | GPs | hypothetical |
| 0.148482 | 0.323884 | 0.528296 | pred_wait_2_detect_1_form1_stage_1_cancers_25_posrisk_2_negrisk_40 | GPs | hypothetical |
| 0.109547 | 0.240304 | 0.394291 | pred_wait_2_detect_1_form1_stage_1_cancers_1_posrisk_4_negrisk_1 | GPs | hypothetical |
| 0.122251 | 0.26442 | 0.429584 | pred_wait_2_detect_1_form1_stage_1_cancers_5_posrisk_4_negrisk_1 | GPs | hypothetical |
| 0.142905 | 0.300534 | 0.480386 | pred_wait_2_detect_1_form1_stage_1_cancers_10_posrisk_4_negrisk_1 | GPs | hypothetical |
| 0.250066 | 0.446788 | 0.651691 | pred_wait_2_detect_1_form1_stage_1_cancers_25_posrisk_4_negrisk_1 | GPs | hypothetical |
| 0.087428 | 0.207584 | 0.353139 | pred_wait_2_detect_1_form1_stage_1_cancers_1_posrisk_4_negrisk_5 | GPs | hypothetical |
| 0.097824 | 0.228828 | 0.385715 | pred_wait_2_detect_1_form1_stage_1_cancers_5_posrisk_4_negrisk_5 | GPs | hypothetical |
| 0.114609 | 0.261381 | 0.434085 | pred_wait_2_detect_1_form1_stage_1_cancers_10_posrisk_4_negrisk_5 | GPs | hypothetical |
| 0.209791 | 0.401397 | 0.609054 | pred_wait_2_detect_1_form1_stage_1_cancers_25_posrisk_4_negrisk_5 | GPs | hypothetical |
| 0.066127 | 0.170744 | 0.304518 | pred_wait_2_detect_1_form1_stage_1_cancers_1_posrisk_4_negrisk_10 | GPs | hypothetical |
| 0.073137 | 0.188371 | 0.332958 | pred_wait_2_detect_1_form1_stage_1_cancers_5_posrisk_4_negrisk_10 | GPs | hypothetical |
| 0.086993 | 0.216151 | 0.376289 | pred_wait_2_detect_1_form1_stage_1_cancers_10_posrisk_4_negrisk_10 | GPs | hypothetical |
| 0.16464 | 0.345036 | 0.550102 | pred_wait_2_detect_1_form1_stage_1_cancers_25_posrisk_4_negrisk_10 | GPs | hypothetical |
| 0.011934 | 0.052231 | 0.115253 | pred_wait_2_detect_1_form1_stage_1_cancers_1_posrisk_4_negrisk_40 | GPs | hypothetical |
| 0.013272 | 0.056813 | 0.124409 | pred_wait_2_detect_1_form1_stage_1_cancers_5_posrisk_4_negrisk_40 | GPs | hypothetical |
| 0.015915 | 0.064978 | 0.140378 | pred_wait_2_detect_1_form1_stage_1_cancers_10_posrisk_4_negrisk_40 | GPs | hypothetical |
| 0.036407 | 0.11642 | 0.231362 | pred_wait_2_detect_1_form1_stage_1_cancers_25_posrisk_4_negrisk_40 | GPs | hypothetical |
| 0.04662 | 0.120473 | 0.217205 | pred_wait_2_detect_1_form1_stage_1_cancers_1_posrisk_6_negrisk_1 | GPs | hypothetical |
| 0.052975 | 0.131826 | 0.233437 | pred_wait_2_detect_1_form1_stage_1_cancers_5_posrisk_6_negrisk_1 | GPs | hypothetical |
| 0.061284 | 0.14935 | 0.259542 | pred_wait_2_detect_1_form1_stage_1_cancers_10_posrisk_6_negrisk_1 | GPs | hypothetical |
| 0.109005 | 0.22944 | 0.369791 | pred_wait_2_detect_1_form1_stage_1_cancers_25_posrisk_6_negrisk_1 | GPs | hypothetical |
| 0.036111 | 0.102505 | 0.191399 | pred_wait_2_detect_1_form1_stage_1_cancers_1_posrisk_6_negrisk_5 | GPs | hypothetical |
| 0.041064 | 0.112244 | 0.20671 | pred_wait_2_detect_1_form1_stage_1_cancers_5_posrisk_6_negrisk_5 | GPs | hypothetical |
| 0.04866 | 0.127537 | 0.22989 | pred_wait_2_detect_1_form1_stage_1_cancers_10_posrisk_6_negrisk_5 | GPs | hypothetical |
| 0.088753 | 0.200367 | 0.333966 | pred_wait_2_detect_1_form1_stage_1_cancers_25_posrisk_6_negrisk_5 | GPs | hypothetical |
| 0.026075 | 0.083252 | 0.161633 | pred_wait_2_detect_1_form1_stage_1_cancers_1_posrisk_6_negrisk_10 | GPs | hypothetical |
| 0.029829 | 0.091163 | 0.176037 | pred_wait_2_detect_1_form1_stage_1_cancers_5_posrisk_6_negrisk_10 | GPs | hypothetical |
| 0.035325 | 0.103858 | 0.195946 | pred_wait_2_detect_1_form1_stage_1_cancers_10_posrisk_6_negrisk_10 | GPs | hypothetical |
| 0.068687 | 0.167477 | 0.28956 | pred_wait_2_detect_1_form1_stage_1_cancers_25_posrisk_6_negrisk_10 | GPs | hypothetical |
| 0.004109 | 0.026114 | 0.064679 | pred_wait_2_detect_1_form1_stage_1_cancers_1_posrisk_6_negrisk_40 | GPs | hypothetical |
| 0.004675 | 0.02827 | 0.068103 | pred_wait_2_detect_1_form1_stage_1_cancers_5_posrisk_6_negrisk_40 | GPs | hypothetical |
| 0.005886 | 0.032088 | 0.07588 | pred_wait_2_detect_1_form1_stage_1_cancers_10_posrisk_6_negrisk_40 | GPs | hypothetical |
| 0.013687 | 0.055808 | 0.118819 | pred_wait_2_detect_1_form1_stage_1_cancers_25_posrisk_6_negrisk_40 | GPs | hypothetical |
| 0.026531 | 0.077031 | 0.14247 | pred_wait_2_detect_1_form1_stage_1_cancers_1_posrisk_8_negrisk_1 | GPs | hypothetical |
| 0.030154 | 0.083752 | 0.151369 | pred_wait_2_detect_1_form1_stage_1_cancers_5_posrisk_8_negrisk_1 | GPs | hypothetical |
| 0.035947 | 0.093946 | 0.167053 | pred_wait_2_detect_1_form1_stage_1_cancers_10_posrisk_8_negrisk_1 | GPs | hypothetical |
| 0.062542 | 0.139674 | 0.230739 | pred_wait_2_detect_1_form1_stage_1_cancers_25_posrisk_8_negrisk_1 | GPs | hypothetical |
| 0.020422 | 0.066498 | 0.126566 | pred_wait_2_detect_1_form1_stage_1_cancers_1_posrisk_8_negrisk_5 | GPs | hypothetical |
| 0.023755 | 0.072357 | 0.134879 | pred_wait_2_detect_1_form1_stage_1_cancers_5_posrisk_8_negrisk_5 | GPs | hypothetical |
| 0.02876 | 0.081356 | 0.148523 | pred_wait_2_detect_1_form1_stage_1_cancers_10_posrisk_8_negrisk_5 | GPs | hypothetical |
| 0.052063 | 0.122804 | 0.20864 | pred_wait_2_detect_1_form1_stage_1_cancers_25_posrisk_8_negrisk_5 | GPs | hypothetical |
| 0.014617 | 0.055196 | 0.108952 | pred_wait_2_detect_1_form1_stage_1_cancers_1_posrisk_8_negrisk_10 | GPs | hypothetical |
| 0.016834 | 0.060081 | 0.117257 | pred_wait_2_detect_1_form1_stage_1_cancers_5_posrisk_8_negrisk_10 | GPs | hypothetical |
| 0.020966 | 0.067707 | 0.129194 | pred_wait_2_detect_1_form1_stage_1_cancers_10_posrisk_8_negrisk_10 | GPs | hypothetical |
| 0.040592 | 0.104061 | 0.182106 | pred_wait_2_detect_1_form1_stage_1_cancers_25_posrisk_8_negrisk_10 | GPs | hypothetical |
| 0.002123 | 0.01975 | 0.050845 | pred_wait_2_detect_1_form1_stage_1_cancers_1_posrisk_8_negrisk_40 | GPs | hypothetical |
| 0.002481 | 0.021388 | 0.053261 | pred_wait_2_detect_1_form1_stage_1_cancers_5_posrisk_8_negrisk_40 | GPs | hypothetical |
| 0.003279 | 0.024122 | 0.058531 | pred_wait_2_detect_1_form1_stage_1_cancers_10_posrisk_8_negrisk_40 | GPs | hypothetical |
| 0.008088 | 0.039314 | 0.083522 | pred_wait_2_detect_1_form1_stage_1_cancers_25_posrisk_8_negrisk_40 | GPs | hypothetical |
| 0.303987 | 0.49117 | 0.680335 | pred_wait_2_detect_1_form1_stage_2_cancers_1_posrisk_2_negrisk_1 | GPs | hypothetical |
| 0.337389 | 0.533724 | 0.725643 | pred_wait_2_detect_1_form1_stage_2_cancers_5_posrisk_2_negrisk_1 | GPs | hypothetical |
| 0.386562 | 0.590536 | 0.781397 | pred_wait_2_detect_1_form1_stage_2_cancers_10_posrisk_2_negrisk_1 | GPs | hypothetical |
| 0.56791 | 0.754595 | 0.901387 | pred_wait_2_detect_1_form1_stage_2_cancers_25_posrisk_2_negrisk_1 | GPs | hypothetical |
| 0.264107 | 0.449719 | 0.640976 | pred_wait_2_detect_1_form1_stage_2_cancers_1_posrisk_2_negrisk_5 | GPs | hypothetical |
| 0.294761 | 0.491222 | 0.689509 | pred_wait_2_detect_1_form1_stage_2_cancers_5_posrisk_2_negrisk_5 | GPs | hypothetical |
| 0.339423 | 0.547987 | 0.749008 | pred_wait_2_detect_1_form1_stage_2_cancers_10_posrisk_2_negrisk_5 | GPs | hypothetical |
| 0.519522 | 0.721161 | 0.884091 | pred_wait_2_detect_1_form1_stage_2_cancers_25_posrisk_2_negrisk_5 | GPs | hypothetical |
| 0.216659 | 0.397211 | 0.589637 | pred_wait_2_detect_1_form1_stage_2_cancers_1_posrisk_2_negrisk_10 | GPs | hypothetical |
| 0.243033 | 0.436297 | 0.63862 | pred_wait_2_detect_1_form1_stage_2_cancers_5_posrisk_2_negrisk_10 | GPs | hypothetical |
| 0.282856 | 0.491391 | 0.701892 | pred_wait_2_detect_1_form1_stage_2_cancers_10_posrisk_2_negrisk_10 | GPs | hypothetical |
| 0.45484 | 0.672423 | 0.856132 | pred_wait_2_detect_1_form1_stage_2_cancers_25_posrisk_2_negrisk_10 | GPs | hypothetical |
| 0.056062 | 0.156536 | 0.284003 | pred_wait_2_detect_1_form1_stage_2_cancers_1_posrisk_2_negrisk_40 | GPs | hypothetical |
| 0.063175 | 0.172773 | 0.312522 | pred_wait_2_detect_1_form1_stage_2_cancers_5_posrisk_2_negrisk_40 | GPs | hypothetical |
| 0.075459 | 0.198764 | 0.355387 | pred_wait_2_detect_1_form1_stage_2_cancers_10_posrisk_2_negrisk_40 | GPs | hypothetical |
| 0.148482 | 0.323884 | 0.528296 | pred_wait_2_detect_1_form1_stage_2_cancers_25_posrisk_2_negrisk_40 | GPs | hypothetical |
| 0.109547 | 0.240304 | 0.394291 | pred_wait_2_detect_1_form1_stage_2_cancers_1_posrisk_4_negrisk_1 | GPs | hypothetical |
| 0.122251 | 0.26442 | 0.429584 | pred_wait_2_detect_1_form1_stage_2_cancers_5_posrisk_4_negrisk_1 | GPs | hypothetical |
| 0.142905 | 0.300534 | 0.480386 | pred_wait_2_detect_1_form1_stage_2_cancers_10_posrisk_4_negrisk_1 | GPs | hypothetical |
| 0.250066 | 0.446788 | 0.651691 | pred_wait_2_detect_1_form1_stage_2_cancers_25_posrisk_4_negrisk_1 | GPs | hypothetical |
| 0.087428 | 0.207584 | 0.353139 | pred_wait_2_detect_1_form1_stage_2_cancers_1_posrisk_4_negrisk_5 | GPs | hypothetical |
| 0.097824 | 0.228828 | 0.385715 | pred_wait_2_detect_1_form1_stage_2_cancers_5_posrisk_4_negrisk_5 | GPs | hypothetical |
| 0.114609 | 0.261381 | 0.434085 | pred_wait_2_detect_1_form1_stage_2_cancers_10_posrisk_4_negrisk_5 | GPs | hypothetical |
| 0.209791 | 0.401397 | 0.609054 | pred_wait_2_detect_1_form1_stage_2_cancers_25_posrisk_4_negrisk_5 | GPs | hypothetical |
| 0.066127 | 0.170744 | 0.304518 | pred_wait_2_detect_1_form1_stage_2_cancers_1_posrisk_4_negrisk_10 | GPs | hypothetical |
| 0.073137 | 0.188371 | 0.332958 | pred_wait_2_detect_1_form1_stage_2_cancers_5_posrisk_4_negrisk_10 | GPs | hypothetical |
| 0.086993 | 0.216151 | 0.376289 | pred_wait_2_detect_1_form1_stage_2_cancers_10_posrisk_4_negrisk_10 | GPs | hypothetical |
| 0.16464 | 0.345036 | 0.550102 | pred_wait_2_detect_1_form1_stage_2_cancers_25_posrisk_4_negrisk_10 | GPs | hypothetical |
| 0.011934 | 0.052231 | 0.115253 | pred_wait_2_detect_1_form1_stage_2_cancers_1_posrisk_4_negrisk_40 | GPs | hypothetical |
| 0.013272 | 0.056813 | 0.124409 | pred_wait_2_detect_1_form1_stage_2_cancers_5_posrisk_4_negrisk_40 | GPs | hypothetical |
| 0.015915 | 0.064978 | 0.140378 | pred_wait_2_detect_1_form1_stage_2_cancers_10_posrisk_4_negrisk_40 | GPs | hypothetical |
| 0.036407 | 0.11642 | 0.231362 | pred_wait_2_detect_1_form1_stage_2_cancers_25_posrisk_4_negrisk_40 | GPs | hypothetical |
| 0.04662 | 0.120473 | 0.217205 | pred_wait_2_detect_1_form1_stage_2_cancers_1_posrisk_6_negrisk_1 | GPs | hypothetical |
| 0.052975 | 0.131826 | 0.233437 | pred_wait_2_detect_1_form1_stage_2_cancers_5_posrisk_6_negrisk_1 | GPs | hypothetical |
| 0.061284 | 0.14935 | 0.259542 | pred_wait_2_detect_1_form1_stage_2_cancers_10_posrisk_6_negrisk_1 | GPs | hypothetical |
| 0.109005 | 0.22944 | 0.369791 | pred_wait_2_detect_1_form1_stage_2_cancers_25_posrisk_6_negrisk_1 | GPs | hypothetical |
| 0.036111 | 0.102505 | 0.191399 | pred_wait_2_detect_1_form1_stage_2_cancers_1_posrisk_6_negrisk_5 | GPs | hypothetical |
| 0.041064 | 0.112244 | 0.20671 | pred_wait_2_detect_1_form1_stage_2_cancers_5_posrisk_6_negrisk_5 | GPs | hypothetical |
| 0.04866 | 0.127537 | 0.22989 | pred_wait_2_detect_1_form1_stage_2_cancers_10_posrisk_6_negrisk_5 | GPs | hypothetical |
| 0.088753 | 0.200367 | 0.333966 | pred_wait_2_detect_1_form1_stage_2_cancers_25_posrisk_6_negrisk_5 | GPs | hypothetical |
| 0.026075 | 0.083252 | 0.161633 | pred_wait_2_detect_1_form1_stage_2_cancers_1_posrisk_6_negrisk_10 | GPs | hypothetical |
| 0.029829 | 0.091163 | 0.176037 | pred_wait_2_detect_1_form1_stage_2_cancers_5_posrisk_6_negrisk_10 | GPs | hypothetical |
| 0.035325 | 0.103858 | 0.195946 | pred_wait_2_detect_1_form1_stage_2_cancers_10_posrisk_6_negrisk_10 | GPs | hypothetical |
| 0.068687 | 0.167477 | 0.28956 | pred_wait_2_detect_1_form1_stage_2_cancers_25_posrisk_6_negrisk_10 | GPs | hypothetical |
| 0.004109 | 0.026114 | 0.064679 | pred_wait_2_detect_1_form1_stage_2_cancers_1_posrisk_6_negrisk_40 | GPs | hypothetical |
| 0.004675 | 0.02827 | 0.068103 | pred_wait_2_detect_1_form1_stage_2_cancers_5_posrisk_6_negrisk_40 | GPs | hypothetical |
| 0.005886 | 0.032088 | 0.07588 | pred_wait_2_detect_1_form1_stage_2_cancers_10_posrisk_6_negrisk_40 | GPs | hypothetical |
| 0.013687 | 0.055808 | 0.118819 | pred_wait_2_detect_1_form1_stage_2_cancers_25_posrisk_6_negrisk_40 | GPs | hypothetical |
| 0.026531 | 0.077031 | 0.14247 | pred_wait_2_detect_1_form1_stage_2_cancers_1_posrisk_8_negrisk_1 | GPs | hypothetical |
| 0.030154 | 0.083752 | 0.151369 | pred_wait_2_detect_1_form1_stage_2_cancers_5_posrisk_8_negrisk_1 | GPs | hypothetical |
| 0.035947 | 0.093946 | 0.167053 | pred_wait_2_detect_1_form1_stage_2_cancers_10_posrisk_8_negrisk_1 | GPs | hypothetical |
| 0.062542 | 0.139674 | 0.230739 | pred_wait_2_detect_1_form1_stage_2_cancers_25_posrisk_8_negrisk_1 | GPs | hypothetical |
| 0.020422 | 0.066498 | 0.126566 | pred_wait_2_detect_1_form1_stage_2_cancers_1_posrisk_8_negrisk_5 | GPs | hypothetical |
| 0.023755 | 0.072357 | 0.134879 | pred_wait_2_detect_1_form1_stage_2_cancers_5_posrisk_8_negrisk_5 | GPs | hypothetical |
| 0.02876 | 0.081356 | 0.148523 | pred_wait_2_detect_1_form1_stage_2_cancers_10_posrisk_8_negrisk_5 | GPs | hypothetical |
| 0.052063 | 0.122804 | 0.20864 | pred_wait_2_detect_1_form1_stage_2_cancers_25_posrisk_8_negrisk_5 | GPs | hypothetical |
| 0.014617 | 0.055196 | 0.108952 | pred_wait_2_detect_1_form1_stage_2_cancers_1_posrisk_8_negrisk_10 | GPs | hypothetical |
| 0.016834 | 0.060081 | 0.117257 | pred_wait_2_detect_1_form1_stage_2_cancers_5_posrisk_8_negrisk_10 | GPs | hypothetical |
| 0.020966 | 0.067707 | 0.129194 | pred_wait_2_detect_1_form1_stage_2_cancers_10_posrisk_8_negrisk_10 | GPs | hypothetical |
| 0.040592 | 0.104061 | 0.182106 | pred_wait_2_detect_1_form1_stage_2_cancers_25_posrisk_8_negrisk_10 | GPs | hypothetical |
| 0.002123 | 0.01975 | 0.050845 | pred_wait_2_detect_1_form1_stage_2_cancers_1_posrisk_8_negrisk_40 | GPs | hypothetical |
| 0.002481 | 0.021388 | 0.053261 | pred_wait_2_detect_1_form1_stage_2_cancers_5_posrisk_8_negrisk_40 | GPs | hypothetical |
| 0.003279 | 0.024122 | 0.058531 | pred_wait_2_detect_1_form1_stage_2_cancers_10_posrisk_8_negrisk_40 | GPs | hypothetical |
| 0.008088 | 0.039314 | 0.083522 | pred_wait_2_detect_1_form1_stage_2_cancers_25_posrisk_8_negrisk_40 | GPs | hypothetical |
| 0.303987 | 0.49117 | 0.680335 | pred_wait_2_detect_1_form2_stage_1_cancers_1_posrisk_2_negrisk_1 | GPs | hypothetical |
| 0.337389 | 0.533724 | 0.725643 | pred_wait_2_detect_1_form2_stage_1_cancers_5_posrisk_2_negrisk_1 | GPs | hypothetical |
| 0.386562 | 0.590536 | 0.781397 | pred_wait_2_detect_1_form2_stage_1_cancers_10_posrisk_2_negrisk_1 | GPs | hypothetical |
| 0.56791 | 0.754595 | 0.901387 | pred_wait_2_detect_1_form2_stage_1_cancers_25_posrisk_2_negrisk_1 | GPs | hypothetical |
| 0.264107 | 0.449719 | 0.640976 | pred_wait_2_detect_1_form2_stage_1_cancers_1_posrisk_2_negrisk_5 | GPs | hypothetical |
| 0.294761 | 0.491222 | 0.689509 | pred_wait_2_detect_1_form2_stage_1_cancers_5_posrisk_2_negrisk_5 | GPs | hypothetical |
| 0.339423 | 0.547987 | 0.749008 | pred_wait_2_detect_1_form2_stage_1_cancers_10_posrisk_2_negrisk_5 | GPs | hypothetical |
| 0.519522 | 0.721161 | 0.884091 | pred_wait_2_detect_1_form2_stage_1_cancers_25_posrisk_2_negrisk_5 | GPs | hypothetical |
| 0.216659 | 0.397211 | 0.589637 | pred_wait_2_detect_1_form2_stage_1_cancers_1_posrisk_2_negrisk_10 | GPs | hypothetical |
| 0.243033 | 0.436297 | 0.63862 | pred_wait_2_detect_1_form2_stage_1_cancers_5_posrisk_2_negrisk_10 | GPs | hypothetical |
| 0.282856 | 0.491391 | 0.701892 | pred_wait_2_detect_1_form2_stage_1_cancers_10_posrisk_2_negrisk_10 | GPs | hypothetical |
| 0.45484 | 0.672423 | 0.856132 | pred_wait_2_detect_1_form2_stage_1_cancers_25_posrisk_2_negrisk_10 | GPs | hypothetical |
| 0.056062 | 0.156536 | 0.284003 | pred_wait_2_detect_1_form2_stage_1_cancers_1_posrisk_2_negrisk_40 | GPs | hypothetical |
| 0.063175 | 0.172773 | 0.312522 | pred_wait_2_detect_1_form2_stage_1_cancers_5_posrisk_2_negrisk_40 | GPs | hypothetical |
| 0.075459 | 0.198764 | 0.355387 | pred_wait_2_detect_1_form2_stage_1_cancers_10_posrisk_2_negrisk_40 | GPs | hypothetical |
| 0.148482 | 0.323884 | 0.528296 | pred_wait_2_detect_1_form2_stage_1_cancers_25_posrisk_2_negrisk_40 | GPs | hypothetical |
| 0.109547 | 0.240304 | 0.394291 | pred_wait_2_detect_1_form2_stage_1_cancers_1_posrisk_4_negrisk_1 | GPs | hypothetical |
| 0.122251 | 0.26442 | 0.429584 | pred_wait_2_detect_1_form2_stage_1_cancers_5_posrisk_4_negrisk_1 | GPs | hypothetical |
| 0.142905 | 0.300534 | 0.480386 | pred_wait_2_detect_1_form2_stage_1_cancers_10_posrisk_4_negrisk_1 | GPs | hypothetical |
| 0.250066 | 0.446788 | 0.651691 | pred_wait_2_detect_1_form2_stage_1_cancers_25_posrisk_4_negrisk_1 | GPs | hypothetical |
| 0.087428 | 0.207584 | 0.353139 | pred_wait_2_detect_1_form2_stage_1_cancers_1_posrisk_4_negrisk_5 | GPs | hypothetical |
| 0.097824 | 0.228828 | 0.385715 | pred_wait_2_detect_1_form2_stage_1_cancers_5_posrisk_4_negrisk_5 | GPs | hypothetical |
| 0.114609 | 0.261381 | 0.434085 | pred_wait_2_detect_1_form2_stage_1_cancers_10_posrisk_4_negrisk_5 | GPs | hypothetical |
| 0.209791 | 0.401397 | 0.609054 | pred_wait_2_detect_1_form2_stage_1_cancers_25_posrisk_4_negrisk_5 | GPs | hypothetical |
| 0.066127 | 0.170744 | 0.304518 | pred_wait_2_detect_1_form2_stage_1_cancers_1_posrisk_4_negrisk_10 | GPs | hypothetical |
| 0.073137 | 0.188371 | 0.332958 | pred_wait_2_detect_1_form2_stage_1_cancers_5_posrisk_4_negrisk_10 | GPs | hypothetical |
| 0.086993 | 0.216151 | 0.376289 | pred_wait_2_detect_1_form2_stage_1_cancers_10_posrisk_4_negrisk_10 | GPs | hypothetical |
| 0.16464 | 0.345036 | 0.550102 | pred_wait_2_detect_1_form2_stage_1_cancers_25_posrisk_4_negrisk_10 | GPs | hypothetical |
| 0.011934 | 0.052231 | 0.115253 | pred_wait_2_detect_1_form2_stage_1_cancers_1_posrisk_4_negrisk_40 | GPs | hypothetical |
| 0.013272 | 0.056813 | 0.124409 | pred_wait_2_detect_1_form2_stage_1_cancers_5_posrisk_4_negrisk_40 | GPs | hypothetical |
| 0.015915 | 0.064978 | 0.140378 | pred_wait_2_detect_1_form2_stage_1_cancers_10_posrisk_4_negrisk_40 | GPs | hypothetical |
| 0.036407 | 0.11642 | 0.231362 | pred_wait_2_detect_1_form2_stage_1_cancers_25_posrisk_4_negrisk_40 | GPs | hypothetical |
| 0.04662 | 0.120473 | 0.217205 | pred_wait_2_detect_1_form2_stage_1_cancers_1_posrisk_6_negrisk_1 | GPs | hypothetical |
| 0.052975 | 0.131826 | 0.233437 | pred_wait_2_detect_1_form2_stage_1_cancers_5_posrisk_6_negrisk_1 | GPs | hypothetical |
| 0.061284 | 0.14935 | 0.259542 | pred_wait_2_detect_1_form2_stage_1_cancers_10_posrisk_6_negrisk_1 | GPs | hypothetical |
| 0.109005 | 0.22944 | 0.369791 | pred_wait_2_detect_1_form2_stage_1_cancers_25_posrisk_6_negrisk_1 | GPs | hypothetical |
| 0.036111 | 0.102505 | 0.191399 | pred_wait_2_detect_1_form2_stage_1_cancers_1_posrisk_6_negrisk_5 | GPs | hypothetical |
| 0.041064 | 0.112244 | 0.20671 | pred_wait_2_detect_1_form2_stage_1_cancers_5_posrisk_6_negrisk_5 | GPs | hypothetical |
| 0.04866 | 0.127537 | 0.22989 | pred_wait_2_detect_1_form2_stage_1_cancers_10_posrisk_6_negrisk_5 | GPs | hypothetical |
| 0.088753 | 0.200367 | 0.333966 | pred_wait_2_detect_1_form2_stage_1_cancers_25_posrisk_6_negrisk_5 | GPs | hypothetical |
| 0.026075 | 0.083252 | 0.161633 | pred_wait_2_detect_1_form2_stage_1_cancers_1_posrisk_6_negrisk_10 | GPs | hypothetical |
| 0.029829 | 0.091163 | 0.176037 | pred_wait_2_detect_1_form2_stage_1_cancers_5_posrisk_6_negrisk_10 | GPs | hypothetical |
| 0.035325 | 0.103858 | 0.195946 | pred_wait_2_detect_1_form2_stage_1_cancers_10_posrisk_6_negrisk_10 | GPs | hypothetical |
| 0.068687 | 0.167477 | 0.28956 | pred_wait_2_detect_1_form2_stage_1_cancers_25_posrisk_6_negrisk_10 | GPs | hypothetical |
| 0.004109 | 0.026114 | 0.064679 | pred_wait_2_detect_1_form2_stage_1_cancers_1_posrisk_6_negrisk_40 | GPs | hypothetical |
| 0.004675 | 0.02827 | 0.068103 | pred_wait_2_detect_1_form2_stage_1_cancers_5_posrisk_6_negrisk_40 | GPs | hypothetical |
| 0.005886 | 0.032088 | 0.07588 | pred_wait_2_detect_1_form2_stage_1_cancers_10_posrisk_6_negrisk_40 | GPs | hypothetical |
| 0.013687 | 0.055808 | 0.118819 | pred_wait_2_detect_1_form2_stage_1_cancers_25_posrisk_6_negrisk_40 | GPs | hypothetical |
| 0.026531 | 0.077031 | 0.14247 | pred_wait_2_detect_1_form2_stage_1_cancers_1_posrisk_8_negrisk_1 | GPs | hypothetical |
| 0.030154 | 0.083752 | 0.151369 | pred_wait_2_detect_1_form2_stage_1_cancers_5_posrisk_8_negrisk_1 | GPs | hypothetical |
| 0.035947 | 0.093946 | 0.167053 | pred_wait_2_detect_1_form2_stage_1_cancers_10_posrisk_8_negrisk_1 | GPs | hypothetical |
| 0.062542 | 0.139674 | 0.230739 | pred_wait_2_detect_1_form2_stage_1_cancers_25_posrisk_8_negrisk_1 | GPs | hypothetical |
| 0.020422 | 0.066498 | 0.126566 | pred_wait_2_detect_1_form2_stage_1_cancers_1_posrisk_8_negrisk_5 | GPs | hypothetical |
| 0.023755 | 0.072357 | 0.134879 | pred_wait_2_detect_1_form2_stage_1_cancers_5_posrisk_8_negrisk_5 | GPs | hypothetical |
| 0.02876 | 0.081356 | 0.148523 | pred_wait_2_detect_1_form2_stage_1_cancers_10_posrisk_8_negrisk_5 | GPs | hypothetical |
| 0.052063 | 0.122804 | 0.20864 | pred_wait_2_detect_1_form2_stage_1_cancers_25_posrisk_8_negrisk_5 | GPs | hypothetical |
| 0.014617 | 0.055196 | 0.108952 | pred_wait_2_detect_1_form2_stage_1_cancers_1_posrisk_8_negrisk_10 | GPs | hypothetical |
| 0.016834 | 0.060081 | 0.117257 | pred_wait_2_detect_1_form2_stage_1_cancers_5_posrisk_8_negrisk_10 | GPs | hypothetical |
| 0.020966 | 0.067707 | 0.129194 | pred_wait_2_detect_1_form2_stage_1_cancers_10_posrisk_8_negrisk_10 | GPs | hypothetical |
| 0.040592 | 0.104061 | 0.182106 | pred_wait_2_detect_1_form2_stage_1_cancers_25_posrisk_8_negrisk_10 | GPs | hypothetical |
| 0.002123 | 0.01975 | 0.050845 | pred_wait_2_detect_1_form2_stage_1_cancers_1_posrisk_8_negrisk_40 | GPs | hypothetical |
| 0.002481 | 0.021388 | 0.053261 | pred_wait_2_detect_1_form2_stage_1_cancers_5_posrisk_8_negrisk_40 | GPs | hypothetical |
| 0.003279 | 0.024122 | 0.058531 | pred_wait_2_detect_1_form2_stage_1_cancers_10_posrisk_8_negrisk_40 | GPs | hypothetical |
| 0.008088 | 0.039314 | 0.083522 | pred_wait_2_detect_1_form2_stage_1_cancers_25_posrisk_8_negrisk_40 | GPs | hypothetical |
| 0.303987 | 0.49117 | 0.680335 | pred_wait_2_detect_1_form2_stage_2_cancers_1_posrisk_2_negrisk_1 | GPs | hypothetical |
| 0.337389 | 0.533724 | 0.725643 | pred_wait_2_detect_1_form2_stage_2_cancers_5_posrisk_2_negrisk_1 | GPs | hypothetical |
| 0.386562 | 0.590536 | 0.781397 | pred_wait_2_detect_1_form2_stage_2_cancers_10_posrisk_2_negrisk_1 | GPs | hypothetical |
| 0.56791 | 0.754595 | 0.901387 | pred_wait_2_detect_1_form2_stage_2_cancers_25_posrisk_2_negrisk_1 | GPs | hypothetical |
| 0.264107 | 0.449719 | 0.640976 | pred_wait_2_detect_1_form2_stage_2_cancers_1_posrisk_2_negrisk_5 | GPs | hypothetical |
| 0.294761 | 0.491222 | 0.689509 | pred_wait_2_detect_1_form2_stage_2_cancers_5_posrisk_2_negrisk_5 | GPs | hypothetical |
| 0.339423 | 0.547987 | 0.749008 | pred_wait_2_detect_1_form2_stage_2_cancers_10_posrisk_2_negrisk_5 | GPs | hypothetical |
| 0.519522 | 0.721161 | 0.884091 | pred_wait_2_detect_1_form2_stage_2_cancers_25_posrisk_2_negrisk_5 | GPs | hypothetical |
| 0.216659 | 0.397211 | 0.589637 | pred_wait_2_detect_1_form2_stage_2_cancers_1_posrisk_2_negrisk_10 | GPs | hypothetical |
| 0.243033 | 0.436297 | 0.63862 | pred_wait_2_detect_1_form2_stage_2_cancers_5_posrisk_2_negrisk_10 | GPs | hypothetical |
| 0.282856 | 0.491391 | 0.701892 | pred_wait_2_detect_1_form2_stage_2_cancers_10_posrisk_2_negrisk_10 | GPs | hypothetical |
| 0.45484 | 0.672423 | 0.856132 | pred_wait_2_detect_1_form2_stage_2_cancers_25_posrisk_2_negrisk_10 | GPs | hypothetical |
| 0.056062 | 0.156536 | 0.284003 | pred_wait_2_detect_1_form2_stage_2_cancers_1_posrisk_2_negrisk_40 | GPs | hypothetical |
| 0.063175 | 0.172773 | 0.312522 | pred_wait_2_detect_1_form2_stage_2_cancers_5_posrisk_2_negrisk_40 | GPs | hypothetical |
| 0.075459 | 0.198764 | 0.355387 | pred_wait_2_detect_1_form2_stage_2_cancers_10_posrisk_2_negrisk_40 | GPs | hypothetical |
| 0.148482 | 0.323884 | 0.528296 | pred_wait_2_detect_1_form2_stage_2_cancers_25_posrisk_2_negrisk_40 | GPs | hypothetical |
| 0.109547 | 0.240304 | 0.394291 | pred_wait_2_detect_1_form2_stage_2_cancers_1_posrisk_4_negrisk_1 | GPs | hypothetical |
| 0.122251 | 0.26442 | 0.429584 | pred_wait_2_detect_1_form2_stage_2_cancers_5_posrisk_4_negrisk_1 | GPs | hypothetical |
| 0.142905 | 0.300534 | 0.480386 | pred_wait_2_detect_1_form2_stage_2_cancers_10_posrisk_4_negrisk_1 | GPs | hypothetical |
| 0.250066 | 0.446788 | 0.651691 | pred_wait_2_detect_1_form2_stage_2_cancers_25_posrisk_4_negrisk_1 | GPs | hypothetical |
| 0.087428 | 0.207584 | 0.353139 | pred_wait_2_detect_1_form2_stage_2_cancers_1_posrisk_4_negrisk_5 | GPs | hypothetical |
| 0.097824 | 0.228828 | 0.385715 | pred_wait_2_detect_1_form2_stage_2_cancers_5_posrisk_4_negrisk_5 | GPs | hypothetical |
| 0.114609 | 0.261381 | 0.434085 | pred_wait_2_detect_1_form2_stage_2_cancers_10_posrisk_4_negrisk_5 | GPs | hypothetical |
| 0.209791 | 0.401397 | 0.609054 | pred_wait_2_detect_1_form2_stage_2_cancers_25_posrisk_4_negrisk_5 | GPs | hypothetical |
| 0.066127 | 0.170744 | 0.304518 | pred_wait_2_detect_1_form2_stage_2_cancers_1_posrisk_4_negrisk_10 | GPs | hypothetical |
| 0.073137 | 0.188371 | 0.332958 | pred_wait_2_detect_1_form2_stage_2_cancers_5_posrisk_4_negrisk_10 | GPs | hypothetical |
| 0.086993 | 0.216151 | 0.376289 | pred_wait_2_detect_1_form2_stage_2_cancers_10_posrisk_4_negrisk_10 | GPs | hypothetical |
| 0.16464 | 0.345036 | 0.550102 | pred_wait_2_detect_1_form2_stage_2_cancers_25_posrisk_4_negrisk_10 | GPs | hypothetical |
| 0.011934 | 0.052231 | 0.115253 | pred_wait_2_detect_1_form2_stage_2_cancers_1_posrisk_4_negrisk_40 | GPs | hypothetical |
| 0.013272 | 0.056813 | 0.124409 | pred_wait_2_detect_1_form2_stage_2_cancers_5_posrisk_4_negrisk_40 | GPs | hypothetical |
| 0.015915 | 0.064978 | 0.140378 | pred_wait_2_detect_1_form2_stage_2_cancers_10_posrisk_4_negrisk_40 | GPs | hypothetical |
| 0.036407 | 0.11642 | 0.231362 | pred_wait_2_detect_1_form2_stage_2_cancers_25_posrisk_4_negrisk_40 | GPs | hypothetical |
| 0.04662 | 0.120473 | 0.217205 | pred_wait_2_detect_1_form2_stage_2_cancers_1_posrisk_6_negrisk_1 | GPs | hypothetical |
| 0.052975 | 0.131826 | 0.233437 | pred_wait_2_detect_1_form2_stage_2_cancers_5_posrisk_6_negrisk_1 | GPs | hypothetical |
| 0.061284 | 0.14935 | 0.259542 | pred_wait_2_detect_1_form2_stage_2_cancers_10_posrisk_6_negrisk_1 | GPs | hypothetical |
| 0.109005 | 0.22944 | 0.369791 | pred_wait_2_detect_1_form2_stage_2_cancers_25_posrisk_6_negrisk_1 | GPs | hypothetical |
| 0.036111 | 0.102505 | 0.191399 | pred_wait_2_detect_1_form2_stage_2_cancers_1_posrisk_6_negrisk_5 | GPs | hypothetical |
| 0.041064 | 0.112244 | 0.20671 | pred_wait_2_detect_1_form2_stage_2_cancers_5_posrisk_6_negrisk_5 | GPs | hypothetical |
| 0.04866 | 0.127537 | 0.22989 | pred_wait_2_detect_1_form2_stage_2_cancers_10_posrisk_6_negrisk_5 | GPs | hypothetical |
| 0.088753 | 0.200367 | 0.333966 | pred_wait_2_detect_1_form2_stage_2_cancers_25_posrisk_6_negrisk_5 | GPs | hypothetical |
| 0.026075 | 0.083252 | 0.161633 | pred_wait_2_detect_1_form2_stage_2_cancers_1_posrisk_6_negrisk_10 | GPs | hypothetical |
| 0.029829 | 0.091163 | 0.176037 | pred_wait_2_detect_1_form2_stage_2_cancers_5_posrisk_6_negrisk_10 | GPs | hypothetical |
| 0.035325 | 0.103858 | 0.195946 | pred_wait_2_detect_1_form2_stage_2_cancers_10_posrisk_6_negrisk_10 | GPs | hypothetical |
| 0.068687 | 0.167477 | 0.28956 | pred_wait_2_detect_1_form2_stage_2_cancers_25_posrisk_6_negrisk_10 | GPs | hypothetical |
| 0.004109 | 0.026114 | 0.064679 | pred_wait_2_detect_1_form2_stage_2_cancers_1_posrisk_6_negrisk_40 | GPs | hypothetical |
| 0.004675 | 0.02827 | 0.068103 | pred_wait_2_detect_1_form2_stage_2_cancers_5_posrisk_6_negrisk_40 | GPs | hypothetical |
| 0.005886 | 0.032088 | 0.07588 | pred_wait_2_detect_1_form2_stage_2_cancers_10_posrisk_6_negrisk_40 | GPs | hypothetical |
| 0.013687 | 0.055808 | 0.118819 | pred_wait_2_detect_1_form2_stage_2_cancers_25_posrisk_6_negrisk_40 | GPs | hypothetical |
| 0.026531 | 0.077031 | 0.14247 | pred_wait_2_detect_1_form2_stage_2_cancers_1_posrisk_8_negrisk_1 | GPs | hypothetical |
| 0.030154 | 0.083752 | 0.151369 | pred_wait_2_detect_1_form2_stage_2_cancers_5_posrisk_8_negrisk_1 | GPs | hypothetical |
| 0.035947 | 0.093946 | 0.167053 | pred_wait_2_detect_1_form2_stage_2_cancers_10_posrisk_8_negrisk_1 | GPs | hypothetical |
| 0.062542 | 0.139674 | 0.230739 | pred_wait_2_detect_1_form2_stage_2_cancers_25_posrisk_8_negrisk_1 | GPs | hypothetical |
| 0.020422 | 0.066498 | 0.126566 | pred_wait_2_detect_1_form2_stage_2_cancers_1_posrisk_8_negrisk_5 | GPs | hypothetical |
| 0.023755 | 0.072357 | 0.134879 | pred_wait_2_detect_1_form2_stage_2_cancers_5_posrisk_8_negrisk_5 | GPs | hypothetical |
| 0.02876 | 0.081356 | 0.148523 | pred_wait_2_detect_1_form2_stage_2_cancers_10_posrisk_8_negrisk_5 | GPs | hypothetical |
| 0.052063 | 0.122804 | 0.20864 | pred_wait_2_detect_1_form2_stage_2_cancers_25_posrisk_8_negrisk_5 | GPs | hypothetical |
| 0.014617 | 0.055196 | 0.108952 | pred_wait_2_detect_1_form2_stage_2_cancers_1_posrisk_8_negrisk_10 | GPs | hypothetical |
| 0.016834 | 0.060081 | 0.117257 | pred_wait_2_detect_1_form2_stage_2_cancers_5_posrisk_8_negrisk_10 | GPs | hypothetical |
| 0.020966 | 0.067707 | 0.129194 | pred_wait_2_detect_1_form2_stage_2_cancers_10_posrisk_8_negrisk_10 | GPs | hypothetical |
| 0.040592 | 0.104061 | 0.182106 | pred_wait_2_detect_1_form2_stage_2_cancers_25_posrisk_8_negrisk_10 | GPs | hypothetical |
| 0.002123 | 0.01975 | 0.050845 | pred_wait_2_detect_1_form2_stage_2_cancers_1_posrisk_8_negrisk_40 | GPs | hypothetical |
| 0.002481 | 0.021388 | 0.053261 | pred_wait_2_detect_1_form2_stage_2_cancers_5_posrisk_8_negrisk_40 | GPs | hypothetical |
| 0.003279 | 0.024122 | 0.058531 | pred_wait_2_detect_1_form2_stage_2_cancers_10_posrisk_8_negrisk_40 | GPs | hypothetical |
| 0.008088 | 0.039314 | 0.083522 | pred_wait_2_detect_1_form2_stage_2_cancers_25_posrisk_8_negrisk_40 | GPs | hypothetical |
| 0.303987 | 0.49117 | 0.680335 | pred_wait_2_detect_1_form3_stage_1_cancers_1_posrisk_2_negrisk_1 | GPs | hypothetical |
| 0.337389 | 0.533724 | 0.725643 | pred_wait_2_detect_1_form3_stage_1_cancers_5_posrisk_2_negrisk_1 | GPs | hypothetical |
| 0.386562 | 0.590536 | 0.781397 | pred_wait_2_detect_1_form3_stage_1_cancers_10_posrisk_2_negrisk_1 | GPs | hypothetical |
| 0.56791 | 0.754595 | 0.901387 | pred_wait_2_detect_1_form3_stage_1_cancers_25_posrisk_2_negrisk_1 | GPs | hypothetical |
| 0.264107 | 0.449719 | 0.640976 | pred_wait_2_detect_1_form3_stage_1_cancers_1_posrisk_2_negrisk_5 | GPs | hypothetical |
| 0.294761 | 0.491222 | 0.689509 | pred_wait_2_detect_1_form3_stage_1_cancers_5_posrisk_2_negrisk_5 | GPs | hypothetical |
| 0.339423 | 0.547987 | 0.749008 | pred_wait_2_detect_1_form3_stage_1_cancers_10_posrisk_2_negrisk_5 | GPs | hypothetical |
| 0.519522 | 0.721161 | 0.884091 | pred_wait_2_detect_1_form3_stage_1_cancers_25_posrisk_2_negrisk_5 | GPs | hypothetical |
| 0.216659 | 0.397211 | 0.589637 | pred_wait_2_detect_1_form3_stage_1_cancers_1_posrisk_2_negrisk_10 | GPs | hypothetical |
| 0.243033 | 0.436297 | 0.63862 | pred_wait_2_detect_1_form3_stage_1_cancers_5_posrisk_2_negrisk_10 | GPs | hypothetical |
| 0.282856 | 0.491391 | 0.701892 | pred_wait_2_detect_1_form3_stage_1_cancers_10_posrisk_2_negrisk_10 | GPs | hypothetical |
| 0.45484 | 0.672423 | 0.856132 | pred_wait_2_detect_1_form3_stage_1_cancers_25_posrisk_2_negrisk_10 | GPs | hypothetical |
| 0.056062 | 0.156536 | 0.284003 | pred_wait_2_detect_1_form3_stage_1_cancers_1_posrisk_2_negrisk_40 | GPs | hypothetical |
| 0.063175 | 0.172773 | 0.312522 | pred_wait_2_detect_1_form3_stage_1_cancers_5_posrisk_2_negrisk_40 | GPs | hypothetical |
| 0.075459 | 0.198764 | 0.355387 | pred_wait_2_detect_1_form3_stage_1_cancers_10_posrisk_2_negrisk_40 | GPs | hypothetical |
| 0.148482 | 0.323884 | 0.528296 | pred_wait_2_detect_1_form3_stage_1_cancers_25_posrisk_2_negrisk_40 | GPs | hypothetical |
| 0.109547 | 0.240304 | 0.394291 | pred_wait_2_detect_1_form3_stage_1_cancers_1_posrisk_4_negrisk_1 | GPs | hypothetical |
| 0.122251 | 0.26442 | 0.429584 | pred_wait_2_detect_1_form3_stage_1_cancers_5_posrisk_4_negrisk_1 | GPs | hypothetical |
| 0.142905 | 0.300534 | 0.480386 | pred_wait_2_detect_1_form3_stage_1_cancers_10_posrisk_4_negrisk_1 | GPs | hypothetical |
| 0.250066 | 0.446788 | 0.651691 | pred_wait_2_detect_1_form3_stage_1_cancers_25_posrisk_4_negrisk_1 | GPs | hypothetical |
| 0.087428 | 0.207584 | 0.353139 | pred_wait_2_detect_1_form3_stage_1_cancers_1_posrisk_4_negrisk_5 | GPs | hypothetical |
| 0.097824 | 0.228828 | 0.385715 | pred_wait_2_detect_1_form3_stage_1_cancers_5_posrisk_4_negrisk_5 | GPs | hypothetical |
| 0.114609 | 0.261381 | 0.434085 | pred_wait_2_detect_1_form3_stage_1_cancers_10_posrisk_4_negrisk_5 | GPs | hypothetical |
| 0.209791 | 0.401397 | 0.609054 | pred_wait_2_detect_1_form3_stage_1_cancers_25_posrisk_4_negrisk_5 | GPs | hypothetical |
| 0.066127 | 0.170744 | 0.304518 | pred_wait_2_detect_1_form3_stage_1_cancers_1_posrisk_4_negrisk_10 | GPs | hypothetical |
| 0.073137 | 0.188371 | 0.332958 | pred_wait_2_detect_1_form3_stage_1_cancers_5_posrisk_4_negrisk_10 | GPs | hypothetical |
| 0.086993 | 0.216151 | 0.376289 | pred_wait_2_detect_1_form3_stage_1_cancers_10_posrisk_4_negrisk_10 | GPs | hypothetical |
| 0.16464 | 0.345036 | 0.550102 | pred_wait_2_detect_1_form3_stage_1_cancers_25_posrisk_4_negrisk_10 | GPs | hypothetical |
| 0.011934 | 0.052231 | 0.115253 | pred_wait_2_detect_1_form3_stage_1_cancers_1_posrisk_4_negrisk_40 | GPs | hypothetical |
| 0.013272 | 0.056813 | 0.124409 | pred_wait_2_detect_1_form3_stage_1_cancers_5_posrisk_4_negrisk_40 | GPs | hypothetical |
| 0.015915 | 0.064978 | 0.140378 | pred_wait_2_detect_1_form3_stage_1_cancers_10_posrisk_4_negrisk_40 | GPs | hypothetical |
| 0.036407 | 0.11642 | 0.231362 | pred_wait_2_detect_1_form3_stage_1_cancers_25_posrisk_4_negrisk_40 | GPs | hypothetical |
| 0.04662 | 0.120473 | 0.217205 | pred_wait_2_detect_1_form3_stage_1_cancers_1_posrisk_6_negrisk_1 | GPs | hypothetical |
| 0.052975 | 0.131826 | 0.233437 | pred_wait_2_detect_1_form3_stage_1_cancers_5_posrisk_6_negrisk_1 | GPs | hypothetical |
| 0.061284 | 0.14935 | 0.259542 | pred_wait_2_detect_1_form3_stage_1_cancers_10_posrisk_6_negrisk_1 | GPs | hypothetical |
| 0.109005 | 0.22944 | 0.369791 | pred_wait_2_detect_1_form3_stage_1_cancers_25_posrisk_6_negrisk_1 | GPs | hypothetical |
| 0.036111 | 0.102505 | 0.191399 | pred_wait_2_detect_1_form3_stage_1_cancers_1_posrisk_6_negrisk_5 | GPs | hypothetical |
| 0.041064 | 0.112244 | 0.20671 | pred_wait_2_detect_1_form3_stage_1_cancers_5_posrisk_6_negrisk_5 | GPs | hypothetical |
| 0.04866 | 0.127537 | 0.22989 | pred_wait_2_detect_1_form3_stage_1_cancers_10_posrisk_6_negrisk_5 | GPs | hypothetical |
| 0.088753 | 0.200367 | 0.333966 | pred_wait_2_detect_1_form3_stage_1_cancers_25_posrisk_6_negrisk_5 | GPs | hypothetical |
| 0.026075 | 0.083252 | 0.161633 | pred_wait_2_detect_1_form3_stage_1_cancers_1_posrisk_6_negrisk_10 | GPs | hypothetical |
| 0.029829 | 0.091163 | 0.176037 | pred_wait_2_detect_1_form3_stage_1_cancers_5_posrisk_6_negrisk_10 | GPs | hypothetical |
| 0.035325 | 0.103858 | 0.195946 | pred_wait_2_detect_1_form3_stage_1_cancers_10_posrisk_6_negrisk_10 | GPs | hypothetical |
| 0.068687 | 0.167477 | 0.28956 | pred_wait_2_detect_1_form3_stage_1_cancers_25_posrisk_6_negrisk_10 | GPs | hypothetical |
| 0.004109 | 0.026114 | 0.064679 | pred_wait_2_detect_1_form3_stage_1_cancers_1_posrisk_6_negrisk_40 | GPs | hypothetical |
| 0.004675 | 0.02827 | 0.068103 | pred_wait_2_detect_1_form3_stage_1_cancers_5_posrisk_6_negrisk_40 | GPs | hypothetical |
| 0.005886 | 0.032088 | 0.07588 | pred_wait_2_detect_1_form3_stage_1_cancers_10_posrisk_6_negrisk_40 | GPs | hypothetical |
| 0.013687 | 0.055808 | 0.118819 | pred_wait_2_detect_1_form3_stage_1_cancers_25_posrisk_6_negrisk_40 | GPs | hypothetical |
| 0.026531 | 0.077031 | 0.14247 | pred_wait_2_detect_1_form3_stage_1_cancers_1_posrisk_8_negrisk_1 | GPs | hypothetical |
| 0.030154 | 0.083752 | 0.151369 | pred_wait_2_detect_1_form3_stage_1_cancers_5_posrisk_8_negrisk_1 | GPs | hypothetical |
| 0.035947 | 0.093946 | 0.167053 | pred_wait_2_detect_1_form3_stage_1_cancers_10_posrisk_8_negrisk_1 | GPs | hypothetical |
| 0.062542 | 0.139674 | 0.230739 | pred_wait_2_detect_1_form3_stage_1_cancers_25_posrisk_8_negrisk_1 | GPs | hypothetical |
| 0.020422 | 0.066498 | 0.126566 | pred_wait_2_detect_1_form3_stage_1_cancers_1_posrisk_8_negrisk_5 | GPs | hypothetical |
| 0.023755 | 0.072357 | 0.134879 | pred_wait_2_detect_1_form3_stage_1_cancers_5_posrisk_8_negrisk_5 | GPs | hypothetical |
| 0.02876 | 0.081356 | 0.148523 | pred_wait_2_detect_1_form3_stage_1_cancers_10_posrisk_8_negrisk_5 | GPs | hypothetical |
| 0.052063 | 0.122804 | 0.20864 | pred_wait_2_detect_1_form3_stage_1_cancers_25_posrisk_8_negrisk_5 | GPs | hypothetical |
| 0.014617 | 0.055196 | 0.108952 | pred_wait_2_detect_1_form3_stage_1_cancers_1_posrisk_8_negrisk_10 | GPs | hypothetical |
| 0.016834 | 0.060081 | 0.117257 | pred_wait_2_detect_1_form3_stage_1_cancers_5_posrisk_8_negrisk_10 | GPs | hypothetical |
| 0.020966 | 0.067707 | 0.129194 | pred_wait_2_detect_1_form3_stage_1_cancers_10_posrisk_8_negrisk_10 | GPs | hypothetical |
| 0.040592 | 0.104061 | 0.182106 | pred_wait_2_detect_1_form3_stage_1_cancers_25_posrisk_8_negrisk_10 | GPs | hypothetical |
| 0.002123 | 0.01975 | 0.050845 | pred_wait_2_detect_1_form3_stage_1_cancers_1_posrisk_8_negrisk_40 | GPs | hypothetical |
| 0.002481 | 0.021388 | 0.053261 | pred_wait_2_detect_1_form3_stage_1_cancers_5_posrisk_8_negrisk_40 | GPs | hypothetical |
| 0.003279 | 0.024122 | 0.058531 | pred_wait_2_detect_1_form3_stage_1_cancers_10_posrisk_8_negrisk_40 | GPs | hypothetical |
| 0.008088 | 0.039314 | 0.083522 | pred_wait_2_detect_1_form3_stage_1_cancers_25_posrisk_8_negrisk_40 | GPs | hypothetical |
| 0.303987 | 0.49117 | 0.680335 | pred_wait_2_detect_1_form3_stage_2_cancers_1_posrisk_2_negrisk_1 | GPs | hypothetical |
| 0.337389 | 0.533724 | 0.725643 | pred_wait_2_detect_1_form3_stage_2_cancers_5_posrisk_2_negrisk_1 | GPs | hypothetical |
| 0.386562 | 0.590536 | 0.781397 | pred_wait_2_detect_1_form3_stage_2_cancers_10_posrisk_2_negrisk_1 | GPs | hypothetical |
| 0.56791 | 0.754595 | 0.901387 | pred_wait_2_detect_1_form3_stage_2_cancers_25_posrisk_2_negrisk_1 | GPs | hypothetical |
| 0.264107 | 0.449719 | 0.640976 | pred_wait_2_detect_1_form3_stage_2_cancers_1_posrisk_2_negrisk_5 | GPs | hypothetical |
| 0.294761 | 0.491222 | 0.689509 | pred_wait_2_detect_1_form3_stage_2_cancers_5_posrisk_2_negrisk_5 | GPs | hypothetical |
| 0.339423 | 0.547987 | 0.749008 | pred_wait_2_detect_1_form3_stage_2_cancers_10_posrisk_2_negrisk_5 | GPs | hypothetical |
| 0.519522 | 0.721161 | 0.884091 | pred_wait_2_detect_1_form3_stage_2_cancers_25_posrisk_2_negrisk_5 | GPs | hypothetical |
| 0.216659 | 0.397211 | 0.589637 | pred_wait_2_detect_1_form3_stage_2_cancers_1_posrisk_2_negrisk_10 | GPs | hypothetical |
| 0.243033 | 0.436297 | 0.63862 | pred_wait_2_detect_1_form3_stage_2_cancers_5_posrisk_2_negrisk_10 | GPs | hypothetical |
| 0.282856 | 0.491391 | 0.701892 | pred_wait_2_detect_1_form3_stage_2_cancers_10_posrisk_2_negrisk_10 | GPs | hypothetical |
| 0.45484 | 0.672423 | 0.856132 | pred_wait_2_detect_1_form3_stage_2_cancers_25_posrisk_2_negrisk_10 | GPs | hypothetical |
| 0.056062 | 0.156536 | 0.284003 | pred_wait_2_detect_1_form3_stage_2_cancers_1_posrisk_2_negrisk_40 | GPs | hypothetical |
| 0.063175 | 0.172773 | 0.312522 | pred_wait_2_detect_1_form3_stage_2_cancers_5_posrisk_2_negrisk_40 | GPs | hypothetical |
| 0.075459 | 0.198764 | 0.355387 | pred_wait_2_detect_1_form3_stage_2_cancers_10_posrisk_2_negrisk_40 | GPs | hypothetical |
| 0.148482 | 0.323884 | 0.528296 | pred_wait_2_detect_1_form3_stage_2_cancers_25_posrisk_2_negrisk_40 | GPs | hypothetical |
| 0.109547 | 0.240304 | 0.394291 | pred_wait_2_detect_1_form3_stage_2_cancers_1_posrisk_4_negrisk_1 | GPs | hypothetical |
| 0.122251 | 0.26442 | 0.429584 | pred_wait_2_detect_1_form3_stage_2_cancers_5_posrisk_4_negrisk_1 | GPs | hypothetical |
| 0.142905 | 0.300534 | 0.480386 | pred_wait_2_detect_1_form3_stage_2_cancers_10_posrisk_4_negrisk_1 | GPs | hypothetical |
| 0.250066 | 0.446788 | 0.651691 | pred_wait_2_detect_1_form3_stage_2_cancers_25_posrisk_4_negrisk_1 | GPs | hypothetical |
| 0.087428 | 0.207584 | 0.353139 | pred_wait_2_detect_1_form3_stage_2_cancers_1_posrisk_4_negrisk_5 | GPs | hypothetical |
| 0.097824 | 0.228828 | 0.385715 | pred_wait_2_detect_1_form3_stage_2_cancers_5_posrisk_4_negrisk_5 | GPs | hypothetical |
| 0.114609 | 0.261381 | 0.434085 | pred_wait_2_detect_1_form3_stage_2_cancers_10_posrisk_4_negrisk_5 | GPs | hypothetical |
| 0.209791 | 0.401397 | 0.609054 | pred_wait_2_detect_1_form3_stage_2_cancers_25_posrisk_4_negrisk_5 | GPs | hypothetical |
| 0.066127 | 0.170744 | 0.304518 | pred_wait_2_detect_1_form3_stage_2_cancers_1_posrisk_4_negrisk_10 | GPs | hypothetical |
| 0.073137 | 0.188371 | 0.332958 | pred_wait_2_detect_1_form3_stage_2_cancers_5_posrisk_4_negrisk_10 | GPs | hypothetical |
| 0.086993 | 0.216151 | 0.376289 | pred_wait_2_detect_1_form3_stage_2_cancers_10_posrisk_4_negrisk_10 | GPs | hypothetical |
| 0.16464 | 0.345036 | 0.550102 | pred_wait_2_detect_1_form3_stage_2_cancers_25_posrisk_4_negrisk_10 | GPs | hypothetical |
| 0.011934 | 0.052231 | 0.115253 | pred_wait_2_detect_1_form3_stage_2_cancers_1_posrisk_4_negrisk_40 | GPs | hypothetical |
| 0.013272 | 0.056813 | 0.124409 | pred_wait_2_detect_1_form3_stage_2_cancers_5_posrisk_4_negrisk_40 | GPs | hypothetical |
| 0.015915 | 0.064978 | 0.140378 | pred_wait_2_detect_1_form3_stage_2_cancers_10_posrisk_4_negrisk_40 | GPs | hypothetical |
| 0.036407 | 0.11642 | 0.231362 | pred_wait_2_detect_1_form3_stage_2_cancers_25_posrisk_4_negrisk_40 | GPs | hypothetical |
| 0.04662 | 0.120473 | 0.217205 | pred_wait_2_detect_1_form3_stage_2_cancers_1_posrisk_6_negrisk_1 | GPs | hypothetical |
| 0.052975 | 0.131826 | 0.233437 | pred_wait_2_detect_1_form3_stage_2_cancers_5_posrisk_6_negrisk_1 | GPs | hypothetical |
| 0.061284 | 0.14935 | 0.259542 | pred_wait_2_detect_1_form3_stage_2_cancers_10_posrisk_6_negrisk_1 | GPs | hypothetical |
| 0.109005 | 0.22944 | 0.369791 | pred_wait_2_detect_1_form3_stage_2_cancers_25_posrisk_6_negrisk_1 | GPs | hypothetical |
| 0.036111 | 0.102505 | 0.191399 | pred_wait_2_detect_1_form3_stage_2_cancers_1_posrisk_6_negrisk_5 | GPs | hypothetical |
| 0.041064 | 0.112244 | 0.20671 | pred_wait_2_detect_1_form3_stage_2_cancers_5_posrisk_6_negrisk_5 | GPs | hypothetical |
| 0.04866 | 0.127537 | 0.22989 | pred_wait_2_detect_1_form3_stage_2_cancers_10_posrisk_6_negrisk_5 | GPs | hypothetical |
| 0.088753 | 0.200367 | 0.333966 | pred_wait_2_detect_1_form3_stage_2_cancers_25_posrisk_6_negrisk_5 | GPs | hypothetical |
| 0.026075 | 0.083252 | 0.161633 | pred_wait_2_detect_1_form3_stage_2_cancers_1_posrisk_6_negrisk_10 | GPs | hypothetical |
| 0.029829 | 0.091163 | 0.176037 | pred_wait_2_detect_1_form3_stage_2_cancers_5_posrisk_6_negrisk_10 | GPs | hypothetical |
| 0.035325 | 0.103858 | 0.195946 | pred_wait_2_detect_1_form3_stage_2_cancers_10_posrisk_6_negrisk_10 | GPs | hypothetical |
| 0.068687 | 0.167477 | 0.28956 | pred_wait_2_detect_1_form3_stage_2_cancers_25_posrisk_6_negrisk_10 | GPs | hypothetical |
| 0.004109 | 0.026114 | 0.064679 | pred_wait_2_detect_1_form3_stage_2_cancers_1_posrisk_6_negrisk_40 | GPs | hypothetical |
| 0.004675 | 0.02827 | 0.068103 | pred_wait_2_detect_1_form3_stage_2_cancers_5_posrisk_6_negrisk_40 | GPs | hypothetical |
| 0.005886 | 0.032088 | 0.07588 | pred_wait_2_detect_1_form3_stage_2_cancers_10_posrisk_6_negrisk_40 | GPs | hypothetical |
| 0.013687 | 0.055808 | 0.118819 | pred_wait_2_detect_1_form3_stage_2_cancers_25_posrisk_6_negrisk_40 | GPs | hypothetical |
| 0.026531 | 0.077031 | 0.14247 | pred_wait_2_detect_1_form3_stage_2_cancers_1_posrisk_8_negrisk_1 | GPs | hypothetical |
| 0.030154 | 0.083752 | 0.151369 | pred_wait_2_detect_1_form3_stage_2_cancers_5_posrisk_8_negrisk_1 | GPs | hypothetical |
| 0.035947 | 0.093946 | 0.167053 | pred_wait_2_detect_1_form3_stage_2_cancers_10_posrisk_8_negrisk_1 | GPs | hypothetical |
| 0.062542 | 0.139674 | 0.230739 | pred_wait_2_detect_1_form3_stage_2_cancers_25_posrisk_8_negrisk_1 | GPs | hypothetical |
| 0.020422 | 0.066498 | 0.126566 | pred_wait_2_detect_1_form3_stage_2_cancers_1_posrisk_8_negrisk_5 | GPs | hypothetical |
| 0.023755 | 0.072357 | 0.134879 | pred_wait_2_detect_1_form3_stage_2_cancers_5_posrisk_8_negrisk_5 | GPs | hypothetical |
| 0.02876 | 0.081356 | 0.148523 | pred_wait_2_detect_1_form3_stage_2_cancers_10_posrisk_8_negrisk_5 | GPs | hypothetical |
| 0.052063 | 0.122804 | 0.20864 | pred_wait_2_detect_1_form3_stage_2_cancers_25_posrisk_8_negrisk_5 | GPs | hypothetical |
| 0.014617 | 0.055196 | 0.108952 | pred_wait_2_detect_1_form3_stage_2_cancers_1_posrisk_8_negrisk_10 | GPs | hypothetical |
| 0.016834 | 0.060081 | 0.117257 | pred_wait_2_detect_1_form3_stage_2_cancers_5_posrisk_8_negrisk_10 | GPs | hypothetical |
| 0.020966 | 0.067707 | 0.129194 | pred_wait_2_detect_1_form3_stage_2_cancers_10_posrisk_8_negrisk_10 | GPs | hypothetical |
| 0.040592 | 0.104061 | 0.182106 | pred_wait_2_detect_1_form3_stage_2_cancers_25_posrisk_8_negrisk_10 | GPs | hypothetical |
| 0.002123 | 0.01975 | 0.050845 | pred_wait_2_detect_1_form3_stage_2_cancers_1_posrisk_8_negrisk_40 | GPs | hypothetical |
| 0.002481 | 0.021388 | 0.053261 | pred_wait_2_detect_1_form3_stage_2_cancers_5_posrisk_8_negrisk_40 | GPs | hypothetical |
| 0.003279 | 0.024122 | 0.058531 | pred_wait_2_detect_1_form3_stage_2_cancers_10_posrisk_8_negrisk_40 | GPs | hypothetical |
| 0.008088 | 0.039314 | 0.083522 | pred_wait_2_detect_1_form3_stage_2_cancers_25_posrisk_8_negrisk_40 | GPs | hypothetical |
| 0.303987 | 0.49117 | 0.680335 | pred_wait_2_detect_1_form4_stage_1_cancers_1_posrisk_2_negrisk_1 | GPs | hypothetical |
| 0.337389 | 0.533724 | 0.725643 | pred_wait_2_detect_1_form4_stage_1_cancers_5_posrisk_2_negrisk_1 | GPs | hypothetical |
| 0.386562 | 0.590536 | 0.781397 | pred_wait_2_detect_1_form4_stage_1_cancers_10_posrisk_2_negrisk_1 | GPs | hypothetical |
| 0.56791 | 0.754595 | 0.901387 | pred_wait_2_detect_1_form4_stage_1_cancers_25_posrisk_2_negrisk_1 | GPs | hypothetical |
| 0.264107 | 0.449719 | 0.640976 | pred_wait_2_detect_1_form4_stage_1_cancers_1_posrisk_2_negrisk_5 | GPs | hypothetical |
| 0.294761 | 0.491222 | 0.689509 | pred_wait_2_detect_1_form4_stage_1_cancers_5_posrisk_2_negrisk_5 | GPs | hypothetical |
| 0.339423 | 0.547987 | 0.749008 | pred_wait_2_detect_1_form4_stage_1_cancers_10_posrisk_2_negrisk_5 | GPs | hypothetical |
| 0.519522 | 0.721161 | 0.884091 | pred_wait_2_detect_1_form4_stage_1_cancers_25_posrisk_2_negrisk_5 | GPs | hypothetical |
| 0.216659 | 0.397211 | 0.589637 | pred_wait_2_detect_1_form4_stage_1_cancers_1_posrisk_2_negrisk_10 | GPs | hypothetical |
| 0.243033 | 0.436297 | 0.63862 | pred_wait_2_detect_1_form4_stage_1_cancers_5_posrisk_2_negrisk_10 | GPs | hypothetical |
| 0.282856 | 0.491391 | 0.701892 | pred_wait_2_detect_1_form4_stage_1_cancers_10_posrisk_2_negrisk_10 | GPs | hypothetical |
| 0.45484 | 0.672423 | 0.856132 | pred_wait_2_detect_1_form4_stage_1_cancers_25_posrisk_2_negrisk_10 | GPs | hypothetical |
| 0.056062 | 0.156536 | 0.284003 | pred_wait_2_detect_1_form4_stage_1_cancers_1_posrisk_2_negrisk_40 | GPs | hypothetical |
| 0.063175 | 0.172773 | 0.312522 | pred_wait_2_detect_1_form4_stage_1_cancers_5_posrisk_2_negrisk_40 | GPs | hypothetical |
| 0.075459 | 0.198764 | 0.355387 | pred_wait_2_detect_1_form4_stage_1_cancers_10_posrisk_2_negrisk_40 | GPs | hypothetical |
| 0.148482 | 0.323884 | 0.528296 | pred_wait_2_detect_1_form4_stage_1_cancers_25_posrisk_2_negrisk_40 | GPs | hypothetical |
| 0.109547 | 0.240304 | 0.394291 | pred_wait_2_detect_1_form4_stage_1_cancers_1_posrisk_4_negrisk_1 | GPs | hypothetical |
| 0.122251 | 0.26442 | 0.429584 | pred_wait_2_detect_1_form4_stage_1_cancers_5_posrisk_4_negrisk_1 | GPs | hypothetical |
| 0.142905 | 0.300534 | 0.480386 | pred_wait_2_detect_1_form4_stage_1_cancers_10_posrisk_4_negrisk_1 | GPs | hypothetical |
| 0.250066 | 0.446788 | 0.651691 | pred_wait_2_detect_1_form4_stage_1_cancers_25_posrisk_4_negrisk_1 | GPs | hypothetical |
| 0.087428 | 0.207584 | 0.353139 | pred_wait_2_detect_1_form4_stage_1_cancers_1_posrisk_4_negrisk_5 | GPs | hypothetical |
| 0.097824 | 0.228828 | 0.385715 | pred_wait_2_detect_1_form4_stage_1_cancers_5_posrisk_4_negrisk_5 | GPs | hypothetical |
| 0.114609 | 0.261381 | 0.434085 | pred_wait_2_detect_1_form4_stage_1_cancers_10_posrisk_4_negrisk_5 | GPs | hypothetical |
| 0.209791 | 0.401397 | 0.609054 | pred_wait_2_detect_1_form4_stage_1_cancers_25_posrisk_4_negrisk_5 | GPs | hypothetical |
| 0.066127 | 0.170744 | 0.304518 | pred_wait_2_detect_1_form4_stage_1_cancers_1_posrisk_4_negrisk_10 | GPs | hypothetical |
| 0.073137 | 0.188371 | 0.332958 | pred_wait_2_detect_1_form4_stage_1_cancers_5_posrisk_4_negrisk_10 | GPs | hypothetical |
| 0.086993 | 0.216151 | 0.376289 | pred_wait_2_detect_1_form4_stage_1_cancers_10_posrisk_4_negrisk_10 | GPs | hypothetical |
| 0.16464 | 0.345036 | 0.550102 | pred_wait_2_detect_1_form4_stage_1_cancers_25_posrisk_4_negrisk_10 | GPs | hypothetical |
| 0.011934 | 0.052231 | 0.115253 | pred_wait_2_detect_1_form4_stage_1_cancers_1_posrisk_4_negrisk_40 | GPs | hypothetical |
| 0.013272 | 0.056813 | 0.124409 | pred_wait_2_detect_1_form4_stage_1_cancers_5_posrisk_4_negrisk_40 | GPs | hypothetical |
| 0.015915 | 0.064978 | 0.140378 | pred_wait_2_detect_1_form4_stage_1_cancers_10_posrisk_4_negrisk_40 | GPs | hypothetical |
| 0.036407 | 0.11642 | 0.231362 | pred_wait_2_detect_1_form4_stage_1_cancers_25_posrisk_4_negrisk_40 | GPs | hypothetical |
| 0.04662 | 0.120473 | 0.217205 | pred_wait_2_detect_1_form4_stage_1_cancers_1_posrisk_6_negrisk_1 | GPs | hypothetical |
| 0.052975 | 0.131826 | 0.233437 | pred_wait_2_detect_1_form4_stage_1_cancers_5_posrisk_6_negrisk_1 | GPs | hypothetical |
| 0.061284 | 0.14935 | 0.259542 | pred_wait_2_detect_1_form4_stage_1_cancers_10_posrisk_6_negrisk_1 | GPs | hypothetical |
| 0.109005 | 0.22944 | 0.369791 | pred_wait_2_detect_1_form4_stage_1_cancers_25_posrisk_6_negrisk_1 | GPs | hypothetical |
| 0.036111 | 0.102505 | 0.191399 | pred_wait_2_detect_1_form4_stage_1_cancers_1_posrisk_6_negrisk_5 | GPs | hypothetical |
| 0.041064 | 0.112244 | 0.20671 | pred_wait_2_detect_1_form4_stage_1_cancers_5_posrisk_6_negrisk_5 | GPs | hypothetical |
| 0.04866 | 0.127537 | 0.22989 | pred_wait_2_detect_1_form4_stage_1_cancers_10_posrisk_6_negrisk_5 | GPs | hypothetical |
| 0.088753 | 0.200367 | 0.333966 | pred_wait_2_detect_1_form4_stage_1_cancers_25_posrisk_6_negrisk_5 | GPs | hypothetical |
| 0.026075 | 0.083252 | 0.161633 | pred_wait_2_detect_1_form4_stage_1_cancers_1_posrisk_6_negrisk_10 | GPs | hypothetical |
| 0.029829 | 0.091163 | 0.176037 | pred_wait_2_detect_1_form4_stage_1_cancers_5_posrisk_6_negrisk_10 | GPs | hypothetical |
| 0.035325 | 0.103858 | 0.195946 | pred_wait_2_detect_1_form4_stage_1_cancers_10_posrisk_6_negrisk_10 | GPs | hypothetical |
| 0.068687 | 0.167477 | 0.28956 | pred_wait_2_detect_1_form4_stage_1_cancers_25_posrisk_6_negrisk_10 | GPs | hypothetical |
| 0.004109 | 0.026114 | 0.064679 | pred_wait_2_detect_1_form4_stage_1_cancers_1_posrisk_6_negrisk_40 | GPs | hypothetical |
| 0.004675 | 0.02827 | 0.068103 | pred_wait_2_detect_1_form4_stage_1_cancers_5_posrisk_6_negrisk_40 | GPs | hypothetical |
| 0.005886 | 0.032088 | 0.07588 | pred_wait_2_detect_1_form4_stage_1_cancers_10_posrisk_6_negrisk_40 | GPs | hypothetical |
| 0.013687 | 0.055808 | 0.118819 | pred_wait_2_detect_1_form4_stage_1_cancers_25_posrisk_6_negrisk_40 | GPs | hypothetical |
| 0.026531 | 0.077031 | 0.14247 | pred_wait_2_detect_1_form4_stage_1_cancers_1_posrisk_8_negrisk_1 | GPs | hypothetical |
| 0.030154 | 0.083752 | 0.151369 | pred_wait_2_detect_1_form4_stage_1_cancers_5_posrisk_8_negrisk_1 | GPs | hypothetical |
| 0.035947 | 0.093946 | 0.167053 | pred_wait_2_detect_1_form4_stage_1_cancers_10_posrisk_8_negrisk_1 | GPs | hypothetical |
| 0.062542 | 0.139674 | 0.230739 | pred_wait_2_detect_1_form4_stage_1_cancers_25_posrisk_8_negrisk_1 | GPs | hypothetical |
| 0.020422 | 0.066498 | 0.126566 | pred_wait_2_detect_1_form4_stage_1_cancers_1_posrisk_8_negrisk_5 | GPs | hypothetical |
| 0.023755 | 0.072357 | 0.134879 | pred_wait_2_detect_1_form4_stage_1_cancers_5_posrisk_8_negrisk_5 | GPs | hypothetical |
| 0.02876 | 0.081356 | 0.148523 | pred_wait_2_detect_1_form4_stage_1_cancers_10_posrisk_8_negrisk_5 | GPs | hypothetical |
| 0.052063 | 0.122804 | 0.20864 | pred_wait_2_detect_1_form4_stage_1_cancers_25_posrisk_8_negrisk_5 | GPs | hypothetical |
| 0.014617 | 0.055196 | 0.108952 | pred_wait_2_detect_1_form4_stage_1_cancers_1_posrisk_8_negrisk_10 | GPs | hypothetical |
| 0.016834 | 0.060081 | 0.117257 | pred_wait_2_detect_1_form4_stage_1_cancers_5_posrisk_8_negrisk_10 | GPs | hypothetical |
| 0.020966 | 0.067707 | 0.129194 | pred_wait_2_detect_1_form4_stage_1_cancers_10_posrisk_8_negrisk_10 | GPs | hypothetical |
| 0.040592 | 0.104061 | 0.182106 | pred_wait_2_detect_1_form4_stage_1_cancers_25_posrisk_8_negrisk_10 | GPs | hypothetical |
| 0.002123 | 0.01975 | 0.050845 | pred_wait_2_detect_1_form4_stage_1_cancers_1_posrisk_8_negrisk_40 | GPs | hypothetical |
| 0.002481 | 0.021388 | 0.053261 | pred_wait_2_detect_1_form4_stage_1_cancers_5_posrisk_8_negrisk_40 | GPs | hypothetical |
| 0.003279 | 0.024122 | 0.058531 | pred_wait_2_detect_1_form4_stage_1_cancers_10_posrisk_8_negrisk_40 | GPs | hypothetical |
| 0.008088 | 0.039314 | 0.083522 | pred_wait_2_detect_1_form4_stage_1_cancers_25_posrisk_8_negrisk_40 | GPs | hypothetical |
| 0.303987 | 0.49117 | 0.680335 | pred_wait_2_detect_1_form4_stage_2_cancers_1_posrisk_2_negrisk_1 | GPs | hypothetical |
| 0.337389 | 0.533724 | 0.725643 | pred_wait_2_detect_1_form4_stage_2_cancers_5_posrisk_2_negrisk_1 | GPs | hypothetical |
| 0.386562 | 0.590536 | 0.781397 | pred_wait_2_detect_1_form4_stage_2_cancers_10_posrisk_2_negrisk_1 | GPs | hypothetical |
| 0.56791 | 0.754595 | 0.901387 | pred_wait_2_detect_1_form4_stage_2_cancers_25_posrisk_2_negrisk_1 | GPs | hypothetical |
| 0.264107 | 0.449719 | 0.640976 | pred_wait_2_detect_1_form4_stage_2_cancers_1_posrisk_2_negrisk_5 | GPs | hypothetical |
| 0.294761 | 0.491222 | 0.689509 | pred_wait_2_detect_1_form4_stage_2_cancers_5_posrisk_2_negrisk_5 | GPs | hypothetical |
| 0.339423 | 0.547987 | 0.749008 | pred_wait_2_detect_1_form4_stage_2_cancers_10_posrisk_2_negrisk_5 | GPs | hypothetical |
| 0.519522 | 0.721161 | 0.884091 | pred_wait_2_detect_1_form4_stage_2_cancers_25_posrisk_2_negrisk_5 | GPs | hypothetical |
| 0.216659 | 0.397211 | 0.589637 | pred_wait_2_detect_1_form4_stage_2_cancers_1_posrisk_2_negrisk_10 | GPs | hypothetical |
| 0.243033 | 0.436297 | 0.63862 | pred_wait_2_detect_1_form4_stage_2_cancers_5_posrisk_2_negrisk_10 | GPs | hypothetical |
| 0.282856 | 0.491391 | 0.701892 | pred_wait_2_detect_1_form4_stage_2_cancers_10_posrisk_2_negrisk_10 | GPs | hypothetical |
| 0.45484 | 0.672423 | 0.856132 | pred_wait_2_detect_1_form4_stage_2_cancers_25_posrisk_2_negrisk_10 | GPs | hypothetical |
| 0.056062 | 0.156536 | 0.284003 | pred_wait_2_detect_1_form4_stage_2_cancers_1_posrisk_2_negrisk_40 | GPs | hypothetical |
| 0.063175 | 0.172773 | 0.312522 | pred_wait_2_detect_1_form4_stage_2_cancers_5_posrisk_2_negrisk_40 | GPs | hypothetical |
| 0.075459 | 0.198764 | 0.355387 | pred_wait_2_detect_1_form4_stage_2_cancers_10_posrisk_2_negrisk_40 | GPs | hypothetical |
| 0.148482 | 0.323884 | 0.528296 | pred_wait_2_detect_1_form4_stage_2_cancers_25_posrisk_2_negrisk_40 | GPs | hypothetical |
| 0.109547 | 0.240304 | 0.394291 | pred_wait_2_detect_1_form4_stage_2_cancers_1_posrisk_4_negrisk_1 | GPs | hypothetical |
| 0.122251 | 0.26442 | 0.429584 | pred_wait_2_detect_1_form4_stage_2_cancers_5_posrisk_4_negrisk_1 | GPs | hypothetical |
| 0.142905 | 0.300534 | 0.480386 | pred_wait_2_detect_1_form4_stage_2_cancers_10_posrisk_4_negrisk_1 | GPs | hypothetical |
| 0.250066 | 0.446788 | 0.651691 | pred_wait_2_detect_1_form4_stage_2_cancers_25_posrisk_4_negrisk_1 | GPs | hypothetical |
| 0.087428 | 0.207584 | 0.353139 | pred_wait_2_detect_1_form4_stage_2_cancers_1_posrisk_4_negrisk_5 | GPs | hypothetical |
| 0.097824 | 0.228828 | 0.385715 | pred_wait_2_detect_1_form4_stage_2_cancers_5_posrisk_4_negrisk_5 | GPs | hypothetical |
| 0.114609 | 0.261381 | 0.434085 | pred_wait_2_detect_1_form4_stage_2_cancers_10_posrisk_4_negrisk_5 | GPs | hypothetical |
| 0.209791 | 0.401397 | 0.609054 | pred_wait_2_detect_1_form4_stage_2_cancers_25_posrisk_4_negrisk_5 | GPs | hypothetical |
| 0.066127 | 0.170744 | 0.304518 | pred_wait_2_detect_1_form4_stage_2_cancers_1_posrisk_4_negrisk_10 | GPs | hypothetical |
| 0.073137 | 0.188371 | 0.332958 | pred_wait_2_detect_1_form4_stage_2_cancers_5_posrisk_4_negrisk_10 | GPs | hypothetical |
| 0.086993 | 0.216151 | 0.376289 | pred_wait_2_detect_1_form4_stage_2_cancers_10_posrisk_4_negrisk_10 | GPs | hypothetical |
| 0.16464 | 0.345036 | 0.550102 | pred_wait_2_detect_1_form4_stage_2_cancers_25_posrisk_4_negrisk_10 | GPs | hypothetical |
| 0.011934 | 0.052231 | 0.115253 | pred_wait_2_detect_1_form4_stage_2_cancers_1_posrisk_4_negrisk_40 | GPs | hypothetical |
| 0.013272 | 0.056813 | 0.124409 | pred_wait_2_detect_1_form4_stage_2_cancers_5_posrisk_4_negrisk_40 | GPs | hypothetical |
| 0.015915 | 0.064978 | 0.140378 | pred_wait_2_detect_1_form4_stage_2_cancers_10_posrisk_4_negrisk_40 | GPs | hypothetical |
| 0.036407 | 0.11642 | 0.231362 | pred_wait_2_detect_1_form4_stage_2_cancers_25_posrisk_4_negrisk_40 | GPs | hypothetical |
| 0.04662 | 0.120473 | 0.217205 | pred_wait_2_detect_1_form4_stage_2_cancers_1_posrisk_6_negrisk_1 | GPs | hypothetical |
| 0.052975 | 0.131826 | 0.233437 | pred_wait_2_detect_1_form4_stage_2_cancers_5_posrisk_6_negrisk_1 | GPs | hypothetical |
| 0.061284 | 0.14935 | 0.259542 | pred_wait_2_detect_1_form4_stage_2_cancers_10_posrisk_6_negrisk_1 | GPs | hypothetical |
| 0.109005 | 0.22944 | 0.369791 | pred_wait_2_detect_1_form4_stage_2_cancers_25_posrisk_6_negrisk_1 | GPs | hypothetical |
| 0.036111 | 0.102505 | 0.191399 | pred_wait_2_detect_1_form4_stage_2_cancers_1_posrisk_6_negrisk_5 | GPs | hypothetical |
| 0.041064 | 0.112244 | 0.20671 | pred_wait_2_detect_1_form4_stage_2_cancers_5_posrisk_6_negrisk_5 | GPs | hypothetical |
| 0.04866 | 0.127537 | 0.22989 | pred_wait_2_detect_1_form4_stage_2_cancers_10_posrisk_6_negrisk_5 | GPs | hypothetical |
| 0.088753 | 0.200367 | 0.333966 | pred_wait_2_detect_1_form4_stage_2_cancers_25_posrisk_6_negrisk_5 | GPs | hypothetical |
| 0.026075 | 0.083252 | 0.161633 | pred_wait_2_detect_1_form4_stage_2_cancers_1_posrisk_6_negrisk_10 | GPs | hypothetical |
| 0.029829 | 0.091163 | 0.176037 | pred_wait_2_detect_1_form4_stage_2_cancers_5_posrisk_6_negrisk_10 | GPs | hypothetical |
| 0.035325 | 0.103858 | 0.195946 | pred_wait_2_detect_1_form4_stage_2_cancers_10_posrisk_6_negrisk_10 | GPs | hypothetical |
| 0.068687 | 0.167477 | 0.28956 | pred_wait_2_detect_1_form4_stage_2_cancers_25_posrisk_6_negrisk_10 | GPs | hypothetical |
| 0.004109 | 0.026114 | 0.064679 | pred_wait_2_detect_1_form4_stage_2_cancers_1_posrisk_6_negrisk_40 | GPs | hypothetical |
| 0.004675 | 0.02827 | 0.068103 | pred_wait_2_detect_1_form4_stage_2_cancers_5_posrisk_6_negrisk_40 | GPs | hypothetical |
| 0.005886 | 0.032088 | 0.07588 | pred_wait_2_detect_1_form4_stage_2_cancers_10_posrisk_6_negrisk_40 | GPs | hypothetical |
| 0.013687 | 0.055808 | 0.118819 | pred_wait_2_detect_1_form4_stage_2_cancers_25_posrisk_6_negrisk_40 | GPs | hypothetical |
| 0.026531 | 0.077031 | 0.14247 | pred_wait_2_detect_1_form4_stage_2_cancers_1_posrisk_8_negrisk_1 | GPs | hypothetical |
| 0.030154 | 0.083752 | 0.151369 | pred_wait_2_detect_1_form4_stage_2_cancers_5_posrisk_8_negrisk_1 | GPs | hypothetical |
| 0.035947 | 0.093946 | 0.167053 | pred_wait_2_detect_1_form4_stage_2_cancers_10_posrisk_8_negrisk_1 | GPs | hypothetical |
| 0.062542 | 0.139674 | 0.230739 | pred_wait_2_detect_1_form4_stage_2_cancers_25_posrisk_8_negrisk_1 | GPs | hypothetical |
| 0.020422 | 0.066498 | 0.126566 | pred_wait_2_detect_1_form4_stage_2_cancers_1_posrisk_8_negrisk_5 | GPs | hypothetical |
| 0.023755 | 0.072357 | 0.134879 | pred_wait_2_detect_1_form4_stage_2_cancers_5_posrisk_8_negrisk_5 | GPs | hypothetical |
| 0.02876 | 0.081356 | 0.148523 | pred_wait_2_detect_1_form4_stage_2_cancers_10_posrisk_8_negrisk_5 | GPs | hypothetical |
| 0.052063 | 0.122804 | 0.20864 | pred_wait_2_detect_1_form4_stage_2_cancers_25_posrisk_8_negrisk_5 | GPs | hypothetical |
| 0.014617 | 0.055196 | 0.108952 | pred_wait_2_detect_1_form4_stage_2_cancers_1_posrisk_8_negrisk_10 | GPs | hypothetical |
| 0.016834 | 0.060081 | 0.117257 | pred_wait_2_detect_1_form4_stage_2_cancers_5_posrisk_8_negrisk_10 | GPs | hypothetical |
| 0.020966 | 0.067707 | 0.129194 | pred_wait_2_detect_1_form4_stage_2_cancers_10_posrisk_8_negrisk_10 | GPs | hypothetical |
| 0.040592 | 0.104061 | 0.182106 | pred_wait_2_detect_1_form4_stage_2_cancers_25_posrisk_8_negrisk_10 | GPs | hypothetical |
| 0.002123 | 0.01975 | 0.050845 | pred_wait_2_detect_1_form4_stage_2_cancers_1_posrisk_8_negrisk_40 | GPs | hypothetical |
| 0.002481 | 0.021388 | 0.053261 | pred_wait_2_detect_1_form4_stage_2_cancers_5_posrisk_8_negrisk_40 | GPs | hypothetical |
| 0.003279 | 0.024122 | 0.058531 | pred_wait_2_detect_1_form4_stage_2_cancers_10_posrisk_8_negrisk_40 | GPs | hypothetical |
| 0.008088 | 0.039314 | 0.083522 | pred_wait_2_detect_1_form4_stage_2_cancers_25_posrisk_8_negrisk_40 | GPs | hypothetical |
| 0.303987 | 0.49117 | 0.680335 | pred_wait_2_detect_2_form1_stage_1_cancers_1_posrisk_2_negrisk_1 | GPs | hypothetical |
| 0.337389 | 0.533724 | 0.725643 | pred_wait_2_detect_2_form1_stage_1_cancers_5_posrisk_2_negrisk_1 | GPs | hypothetical |
| 0.386562 | 0.590536 | 0.781397 | pred_wait_2_detect_2_form1_stage_1_cancers_10_posrisk_2_negrisk_1 | GPs | hypothetical |
| 0.56791 | 0.754595 | 0.901387 | pred_wait_2_detect_2_form1_stage_1_cancers_25_posrisk_2_negrisk_1 | GPs | hypothetical |
| 0.264107 | 0.449719 | 0.640976 | pred_wait_2_detect_2_form1_stage_1_cancers_1_posrisk_2_negrisk_5 | GPs | hypothetical |
| 0.294761 | 0.491222 | 0.689509 | pred_wait_2_detect_2_form1_stage_1_cancers_5_posrisk_2_negrisk_5 | GPs | hypothetical |
| 0.339423 | 0.547987 | 0.749008 | pred_wait_2_detect_2_form1_stage_1_cancers_10_posrisk_2_negrisk_5 | GPs | hypothetical |
| 0.519522 | 0.721161 | 0.884091 | pred_wait_2_detect_2_form1_stage_1_cancers_25_posrisk_2_negrisk_5 | GPs | hypothetical |
| 0.216659 | 0.397211 | 0.589637 | pred_wait_2_detect_2_form1_stage_1_cancers_1_posrisk_2_negrisk_10 | GPs | hypothetical |
| 0.243033 | 0.436297 | 0.63862 | pred_wait_2_detect_2_form1_stage_1_cancers_5_posrisk_2_negrisk_10 | GPs | hypothetical |
| 0.282856 | 0.491391 | 0.701892 | pred_wait_2_detect_2_form1_stage_1_cancers_10_posrisk_2_negrisk_10 | GPs | hypothetical |
| 0.45484 | 0.672423 | 0.856132 | pred_wait_2_detect_2_form1_stage_1_cancers_25_posrisk_2_negrisk_10 | GPs | hypothetical |
| 0.056062 | 0.156536 | 0.284003 | pred_wait_2_detect_2_form1_stage_1_cancers_1_posrisk_2_negrisk_40 | GPs | hypothetical |
| 0.063175 | 0.172773 | 0.312522 | pred_wait_2_detect_2_form1_stage_1_cancers_5_posrisk_2_negrisk_40 | GPs | hypothetical |
| 0.075459 | 0.198764 | 0.355387 | pred_wait_2_detect_2_form1_stage_1_cancers_10_posrisk_2_negrisk_40 | GPs | hypothetical |
| 0.148482 | 0.323884 | 0.528296 | pred_wait_2_detect_2_form1_stage_1_cancers_25_posrisk_2_negrisk_40 | GPs | hypothetical |
| 0.109547 | 0.240304 | 0.394291 | pred_wait_2_detect_2_form1_stage_1_cancers_1_posrisk_4_negrisk_1 | GPs | hypothetical |
| 0.122251 | 0.26442 | 0.429584 | pred_wait_2_detect_2_form1_stage_1_cancers_5_posrisk_4_negrisk_1 | GPs | hypothetical |
| 0.142905 | 0.300534 | 0.480386 | pred_wait_2_detect_2_form1_stage_1_cancers_10_posrisk_4_negrisk_1 | GPs | hypothetical |
| 0.250066 | 0.446788 | 0.651691 | pred_wait_2_detect_2_form1_stage_1_cancers_25_posrisk_4_negrisk_1 | GPs | hypothetical |
| 0.087428 | 0.207584 | 0.353139 | pred_wait_2_detect_2_form1_stage_1_cancers_1_posrisk_4_negrisk_5 | GPs | hypothetical |
| 0.097824 | 0.228828 | 0.385715 | pred_wait_2_detect_2_form1_stage_1_cancers_5_posrisk_4_negrisk_5 | GPs | hypothetical |
| 0.114609 | 0.261381 | 0.434085 | pred_wait_2_detect_2_form1_stage_1_cancers_10_posrisk_4_negrisk_5 | GPs | hypothetical |
| 0.209791 | 0.401397 | 0.609054 | pred_wait_2_detect_2_form1_stage_1_cancers_25_posrisk_4_negrisk_5 | GPs | hypothetical |
| 0.066127 | 0.170744 | 0.304518 | pred_wait_2_detect_2_form1_stage_1_cancers_1_posrisk_4_negrisk_10 | GPs | hypothetical |
| 0.073137 | 0.188371 | 0.332958 | pred_wait_2_detect_2_form1_stage_1_cancers_5_posrisk_4_negrisk_10 | GPs | hypothetical |
| 0.086993 | 0.216151 | 0.376289 | pred_wait_2_detect_2_form1_stage_1_cancers_10_posrisk_4_negrisk_10 | GPs | hypothetical |
| 0.16464 | 0.345036 | 0.550102 | pred_wait_2_detect_2_form1_stage_1_cancers_25_posrisk_4_negrisk_10 | GPs | hypothetical |
| 0.011934 | 0.052231 | 0.115253 | pred_wait_2_detect_2_form1_stage_1_cancers_1_posrisk_4_negrisk_40 | GPs | hypothetical |
| 0.013272 | 0.056813 | 0.124409 | pred_wait_2_detect_2_form1_stage_1_cancers_5_posrisk_4_negrisk_40 | GPs | hypothetical |
| 0.015915 | 0.064978 | 0.140378 | pred_wait_2_detect_2_form1_stage_1_cancers_10_posrisk_4_negrisk_40 | GPs | hypothetical |
| 0.036407 | 0.11642 | 0.231362 | pred_wait_2_detect_2_form1_stage_1_cancers_25_posrisk_4_negrisk_40 | GPs | hypothetical |
| 0.04662 | 0.120473 | 0.217205 | pred_wait_2_detect_2_form1_stage_1_cancers_1_posrisk_6_negrisk_1 | GPs | hypothetical |
| 0.052975 | 0.131826 | 0.233437 | pred_wait_2_detect_2_form1_stage_1_cancers_5_posrisk_6_negrisk_1 | GPs | hypothetical |
| 0.061284 | 0.14935 | 0.259542 | pred_wait_2_detect_2_form1_stage_1_cancers_10_posrisk_6_negrisk_1 | GPs | hypothetical |
| 0.109005 | 0.22944 | 0.369791 | pred_wait_2_detect_2_form1_stage_1_cancers_25_posrisk_6_negrisk_1 | GPs | hypothetical |
| 0.036111 | 0.102505 | 0.191399 | pred_wait_2_detect_2_form1_stage_1_cancers_1_posrisk_6_negrisk_5 | GPs | hypothetical |
| 0.041064 | 0.112244 | 0.20671 | pred_wait_2_detect_2_form1_stage_1_cancers_5_posrisk_6_negrisk_5 | GPs | hypothetical |
| 0.04866 | 0.127537 | 0.22989 | pred_wait_2_detect_2_form1_stage_1_cancers_10_posrisk_6_negrisk_5 | GPs | hypothetical |
| 0.088753 | 0.200367 | 0.333966 | pred_wait_2_detect_2_form1_stage_1_cancers_25_posrisk_6_negrisk_5 | GPs | hypothetical |
| 0.026075 | 0.083252 | 0.161633 | pred_wait_2_detect_2_form1_stage_1_cancers_1_posrisk_6_negrisk_10 | GPs | hypothetical |
| 0.029829 | 0.091163 | 0.176037 | pred_wait_2_detect_2_form1_stage_1_cancers_5_posrisk_6_negrisk_10 | GPs | hypothetical |
| 0.035325 | 0.103858 | 0.195946 | pred_wait_2_detect_2_form1_stage_1_cancers_10_posrisk_6_negrisk_10 | GPs | hypothetical |
| 0.068687 | 0.167477 | 0.28956 | pred_wait_2_detect_2_form1_stage_1_cancers_25_posrisk_6_negrisk_10 | GPs | hypothetical |
| 0.004109 | 0.026114 | 0.064679 | pred_wait_2_detect_2_form1_stage_1_cancers_1_posrisk_6_negrisk_40 | GPs | hypothetical |
| 0.004675 | 0.02827 | 0.068103 | pred_wait_2_detect_2_form1_stage_1_cancers_5_posrisk_6_negrisk_40 | GPs | hypothetical |
| 0.005886 | 0.032088 | 0.07588 | pred_wait_2_detect_2_form1_stage_1_cancers_10_posrisk_6_negrisk_40 | GPs | hypothetical |
| 0.013687 | 0.055808 | 0.118819 | pred_wait_2_detect_2_form1_stage_1_cancers_25_posrisk_6_negrisk_40 | GPs | hypothetical |
| 0.026531 | 0.077031 | 0.14247 | pred_wait_2_detect_2_form1_stage_1_cancers_1_posrisk_8_negrisk_1 | GPs | hypothetical |
| 0.030154 | 0.083752 | 0.151369 | pred_wait_2_detect_2_form1_stage_1_cancers_5_posrisk_8_negrisk_1 | GPs | hypothetical |
| 0.035947 | 0.093946 | 0.167053 | pred_wait_2_detect_2_form1_stage_1_cancers_10_posrisk_8_negrisk_1 | GPs | hypothetical |
| 0.062542 | 0.139674 | 0.230739 | pred_wait_2_detect_2_form1_stage_1_cancers_25_posrisk_8_negrisk_1 | GPs | hypothetical |
| 0.020422 | 0.066498 | 0.126566 | pred_wait_2_detect_2_form1_stage_1_cancers_1_posrisk_8_negrisk_5 | GPs | hypothetical |
| 0.023755 | 0.072357 | 0.134879 | pred_wait_2_detect_2_form1_stage_1_cancers_5_posrisk_8_negrisk_5 | GPs | hypothetical |
| 0.02876 | 0.081356 | 0.148523 | pred_wait_2_detect_2_form1_stage_1_cancers_10_posrisk_8_negrisk_5 | GPs | hypothetical |
| 0.052063 | 0.122804 | 0.20864 | pred_wait_2_detect_2_form1_stage_1_cancers_25_posrisk_8_negrisk_5 | GPs | hypothetical |
| 0.014617 | 0.055196 | 0.108952 | pred_wait_2_detect_2_form1_stage_1_cancers_1_posrisk_8_negrisk_10 | GPs | hypothetical |
| 0.016834 | 0.060081 | 0.117257 | pred_wait_2_detect_2_form1_stage_1_cancers_5_posrisk_8_negrisk_10 | GPs | hypothetical |
| 0.020966 | 0.067707 | 0.129194 | pred_wait_2_detect_2_form1_stage_1_cancers_10_posrisk_8_negrisk_10 | GPs | hypothetical |
| 0.040592 | 0.104061 | 0.182106 | pred_wait_2_detect_2_form1_stage_1_cancers_25_posrisk_8_negrisk_10 | GPs | hypothetical |
| 0.002123 | 0.01975 | 0.050845 | pred_wait_2_detect_2_form1_stage_1_cancers_1_posrisk_8_negrisk_40 | GPs | hypothetical |
| 0.002481 | 0.021388 | 0.053261 | pred_wait_2_detect_2_form1_stage_1_cancers_5_posrisk_8_negrisk_40 | GPs | hypothetical |
| 0.003279 | 0.024122 | 0.058531 | pred_wait_2_detect_2_form1_stage_1_cancers_10_posrisk_8_negrisk_40 | GPs | hypothetical |
| 0.008088 | 0.039314 | 0.083522 | pred_wait_2_detect_2_form1_stage_1_cancers_25_posrisk_8_negrisk_40 | GPs | hypothetical |
| 0.303987 | 0.49117 | 0.680335 | pred_wait_2_detect_2_form1_stage_2_cancers_1_posrisk_2_negrisk_1 | GPs | hypothetical |
| 0.337389 | 0.533724 | 0.725643 | pred_wait_2_detect_2_form1_stage_2_cancers_5_posrisk_2_negrisk_1 | GPs | hypothetical |
| 0.386562 | 0.590536 | 0.781397 | pred_wait_2_detect_2_form1_stage_2_cancers_10_posrisk_2_negrisk_1 | GPs | hypothetical |
| 0.56791 | 0.754595 | 0.901387 | pred_wait_2_detect_2_form1_stage_2_cancers_25_posrisk_2_negrisk_1 | GPs | hypothetical |
| 0.264107 | 0.449719 | 0.640976 | pred_wait_2_detect_2_form1_stage_2_cancers_1_posrisk_2_negrisk_5 | GPs | hypothetical |
| 0.294761 | 0.491222 | 0.689509 | pred_wait_2_detect_2_form1_stage_2_cancers_5_posrisk_2_negrisk_5 | GPs | hypothetical |
| 0.339423 | 0.547987 | 0.749008 | pred_wait_2_detect_2_form1_stage_2_cancers_10_posrisk_2_negrisk_5 | GPs | hypothetical |
| 0.519522 | 0.721161 | 0.884091 | pred_wait_2_detect_2_form1_stage_2_cancers_25_posrisk_2_negrisk_5 | GPs | hypothetical |
| 0.216659 | 0.397211 | 0.589637 | pred_wait_2_detect_2_form1_stage_2_cancers_1_posrisk_2_negrisk_10 | GPs | hypothetical |
| 0.243033 | 0.436297 | 0.63862 | pred_wait_2_detect_2_form1_stage_2_cancers_5_posrisk_2_negrisk_10 | GPs | hypothetical |
| 0.282856 | 0.491391 | 0.701892 | pred_wait_2_detect_2_form1_stage_2_cancers_10_posrisk_2_negrisk_10 | GPs | hypothetical |
| 0.45484 | 0.672423 | 0.856132 | pred_wait_2_detect_2_form1_stage_2_cancers_25_posrisk_2_negrisk_10 | GPs | hypothetical |
| 0.056062 | 0.156536 | 0.284003 | pred_wait_2_detect_2_form1_stage_2_cancers_1_posrisk_2_negrisk_40 | GPs | hypothetical |
| 0.063175 | 0.172773 | 0.312522 | pred_wait_2_detect_2_form1_stage_2_cancers_5_posrisk_2_negrisk_40 | GPs | hypothetical |
| 0.075459 | 0.198764 | 0.355387 | pred_wait_2_detect_2_form1_stage_2_cancers_10_posrisk_2_negrisk_40 | GPs | hypothetical |
| 0.148482 | 0.323884 | 0.528296 | pred_wait_2_detect_2_form1_stage_2_cancers_25_posrisk_2_negrisk_40 | GPs | hypothetical |
| 0.109547 | 0.240304 | 0.394291 | pred_wait_2_detect_2_form1_stage_2_cancers_1_posrisk_4_negrisk_1 | GPs | hypothetical |
| 0.122251 | 0.26442 | 0.429584 | pred_wait_2_detect_2_form1_stage_2_cancers_5_posrisk_4_negrisk_1 | GPs | hypothetical |
| 0.142905 | 0.300534 | 0.480386 | pred_wait_2_detect_2_form1_stage_2_cancers_10_posrisk_4_negrisk_1 | GPs | hypothetical |
| 0.250066 | 0.446788 | 0.651691 | pred_wait_2_detect_2_form1_stage_2_cancers_25_posrisk_4_negrisk_1 | GPs | hypothetical |
| 0.087428 | 0.207584 | 0.353139 | pred_wait_2_detect_2_form1_stage_2_cancers_1_posrisk_4_negrisk_5 | GPs | hypothetical |
| 0.097824 | 0.228828 | 0.385715 | pred_wait_2_detect_2_form1_stage_2_cancers_5_posrisk_4_negrisk_5 | GPs | hypothetical |
| 0.114609 | 0.261381 | 0.434085 | pred_wait_2_detect_2_form1_stage_2_cancers_10_posrisk_4_negrisk_5 | GPs | hypothetical |
| 0.209791 | 0.401397 | 0.609054 | pred_wait_2_detect_2_form1_stage_2_cancers_25_posrisk_4_negrisk_5 | GPs | hypothetical |
| 0.066127 | 0.170744 | 0.304518 | pred_wait_2_detect_2_form1_stage_2_cancers_1_posrisk_4_negrisk_10 | GPs | hypothetical |
| 0.073137 | 0.188371 | 0.332958 | pred_wait_2_detect_2_form1_stage_2_cancers_5_posrisk_4_negrisk_10 | GPs | hypothetical |
| 0.086993 | 0.216151 | 0.376289 | pred_wait_2_detect_2_form1_stage_2_cancers_10_posrisk_4_negrisk_10 | GPs | hypothetical |
| 0.16464 | 0.345036 | 0.550102 | pred_wait_2_detect_2_form1_stage_2_cancers_25_posrisk_4_negrisk_10 | GPs | hypothetical |
| 0.011934 | 0.052231 | 0.115253 | pred_wait_2_detect_2_form1_stage_2_cancers_1_posrisk_4_negrisk_40 | GPs | hypothetical |
| 0.013272 | 0.056813 | 0.124409 | pred_wait_2_detect_2_form1_stage_2_cancers_5_posrisk_4_negrisk_40 | GPs | hypothetical |
| 0.015915 | 0.064978 | 0.140378 | pred_wait_2_detect_2_form1_stage_2_cancers_10_posrisk_4_negrisk_40 | GPs | hypothetical |
| 0.036407 | 0.11642 | 0.231362 | pred_wait_2_detect_2_form1_stage_2_cancers_25_posrisk_4_negrisk_40 | GPs | hypothetical |
| 0.04662 | 0.120473 | 0.217205 | pred_wait_2_detect_2_form1_stage_2_cancers_1_posrisk_6_negrisk_1 | GPs | hypothetical |
| 0.052975 | 0.131826 | 0.233437 | pred_wait_2_detect_2_form1_stage_2_cancers_5_posrisk_6_negrisk_1 | GPs | hypothetical |
| 0.061284 | 0.14935 | 0.259542 | pred_wait_2_detect_2_form1_stage_2_cancers_10_posrisk_6_negrisk_1 | GPs | hypothetical |
| 0.109005 | 0.22944 | 0.369791 | pred_wait_2_detect_2_form1_stage_2_cancers_25_posrisk_6_negrisk_1 | GPs | hypothetical |
| 0.036111 | 0.102505 | 0.191399 | pred_wait_2_detect_2_form1_stage_2_cancers_1_posrisk_6_negrisk_5 | GPs | hypothetical |
| 0.041064 | 0.112244 | 0.20671 | pred_wait_2_detect_2_form1_stage_2_cancers_5_posrisk_6_negrisk_5 | GPs | hypothetical |
| 0.04866 | 0.127537 | 0.22989 | pred_wait_2_detect_2_form1_stage_2_cancers_10_posrisk_6_negrisk_5 | GPs | hypothetical |
| 0.088753 | 0.200367 | 0.333966 | pred_wait_2_detect_2_form1_stage_2_cancers_25_posrisk_6_negrisk_5 | GPs | hypothetical |
| 0.026075 | 0.083252 | 0.161633 | pred_wait_2_detect_2_form1_stage_2_cancers_1_posrisk_6_negrisk_10 | GPs | hypothetical |
| 0.029829 | 0.091163 | 0.176037 | pred_wait_2_detect_2_form1_stage_2_cancers_5_posrisk_6_negrisk_10 | GPs | hypothetical |
| 0.035325 | 0.103858 | 0.195946 | pred_wait_2_detect_2_form1_stage_2_cancers_10_posrisk_6_negrisk_10 | GPs | hypothetical |
| 0.068687 | 0.167477 | 0.28956 | pred_wait_2_detect_2_form1_stage_2_cancers_25_posrisk_6_negrisk_10 | GPs | hypothetical |
| 0.004109 | 0.026114 | 0.064679 | pred_wait_2_detect_2_form1_stage_2_cancers_1_posrisk_6_negrisk_40 | GPs | hypothetical |
| 0.004675 | 0.02827 | 0.068103 | pred_wait_2_detect_2_form1_stage_2_cancers_5_posrisk_6_negrisk_40 | GPs | hypothetical |
| 0.005886 | 0.032088 | 0.07588 | pred_wait_2_detect_2_form1_stage_2_cancers_10_posrisk_6_negrisk_40 | GPs | hypothetical |
| 0.013687 | 0.055808 | 0.118819 | pred_wait_2_detect_2_form1_stage_2_cancers_25_posrisk_6_negrisk_40 | GPs | hypothetical |
| 0.026531 | 0.077031 | 0.14247 | pred_wait_2_detect_2_form1_stage_2_cancers_1_posrisk_8_negrisk_1 | GPs | hypothetical |
| 0.030154 | 0.083752 | 0.151369 | pred_wait_2_detect_2_form1_stage_2_cancers_5_posrisk_8_negrisk_1 | GPs | hypothetical |
| 0.035947 | 0.093946 | 0.167053 | pred_wait_2_detect_2_form1_stage_2_cancers_10_posrisk_8_negrisk_1 | GPs | hypothetical |
| 0.062542 | 0.139674 | 0.230739 | pred_wait_2_detect_2_form1_stage_2_cancers_25_posrisk_8_negrisk_1 | GPs | hypothetical |
| 0.020422 | 0.066498 | 0.126566 | pred_wait_2_detect_2_form1_stage_2_cancers_1_posrisk_8_negrisk_5 | GPs | hypothetical |
| 0.023755 | 0.072357 | 0.134879 | pred_wait_2_detect_2_form1_stage_2_cancers_5_posrisk_8_negrisk_5 | GPs | hypothetical |
| 0.02876 | 0.081356 | 0.148523 | pred_wait_2_detect_2_form1_stage_2_cancers_10_posrisk_8_negrisk_5 | GPs | hypothetical |
| 0.052063 | 0.122804 | 0.20864 | pred_wait_2_detect_2_form1_stage_2_cancers_25_posrisk_8_negrisk_5 | GPs | hypothetical |
| 0.014617 | 0.055196 | 0.108952 | pred_wait_2_detect_2_form1_stage_2_cancers_1_posrisk_8_negrisk_10 | GPs | hypothetical |
| 0.016834 | 0.060081 | 0.117257 | pred_wait_2_detect_2_form1_stage_2_cancers_5_posrisk_8_negrisk_10 | GPs | hypothetical |
| 0.020966 | 0.067707 | 0.129194 | pred_wait_2_detect_2_form1_stage_2_cancers_10_posrisk_8_negrisk_10 | GPs | hypothetical |
| 0.040592 | 0.104061 | 0.182106 | pred_wait_2_detect_2_form1_stage_2_cancers_25_posrisk_8_negrisk_10 | GPs | hypothetical |
| 0.002123 | 0.01975 | 0.050845 | pred_wait_2_detect_2_form1_stage_2_cancers_1_posrisk_8_negrisk_40 | GPs | hypothetical |
| 0.002481 | 0.021388 | 0.053261 | pred_wait_2_detect_2_form1_stage_2_cancers_5_posrisk_8_negrisk_40 | GPs | hypothetical |
| 0.003279 | 0.024122 | 0.058531 | pred_wait_2_detect_2_form1_stage_2_cancers_10_posrisk_8_negrisk_40 | GPs | hypothetical |
| 0.008088 | 0.039314 | 0.083522 | pred_wait_2_detect_2_form1_stage_2_cancers_25_posrisk_8_negrisk_40 | GPs | hypothetical |
| 0.303987 | 0.49117 | 0.680335 | pred_wait_2_detect_2_form2_stage_1_cancers_1_posrisk_2_negrisk_1 | GPs | hypothetical |
| 0.337389 | 0.533724 | 0.725643 | pred_wait_2_detect_2_form2_stage_1_cancers_5_posrisk_2_negrisk_1 | GPs | hypothetical |
| 0.386562 | 0.590536 | 0.781397 | pred_wait_2_detect_2_form2_stage_1_cancers_10_posrisk_2_negrisk_1 | GPs | hypothetical |
| 0.56791 | 0.754595 | 0.901387 | pred_wait_2_detect_2_form2_stage_1_cancers_25_posrisk_2_negrisk_1 | GPs | hypothetical |
| 0.264107 | 0.449719 | 0.640976 | pred_wait_2_detect_2_form2_stage_1_cancers_1_posrisk_2_negrisk_5 | GPs | hypothetical |
| 0.294761 | 0.491222 | 0.689509 | pred_wait_2_detect_2_form2_stage_1_cancers_5_posrisk_2_negrisk_5 | GPs | hypothetical |
| 0.339423 | 0.547987 | 0.749008 | pred_wait_2_detect_2_form2_stage_1_cancers_10_posrisk_2_negrisk_5 | GPs | hypothetical |
| 0.519522 | 0.721161 | 0.884091 | pred_wait_2_detect_2_form2_stage_1_cancers_25_posrisk_2_negrisk_5 | GPs | hypothetical |
| 0.216659 | 0.397211 | 0.589637 | pred_wait_2_detect_2_form2_stage_1_cancers_1_posrisk_2_negrisk_10 | GPs | hypothetical |
| 0.243033 | 0.436297 | 0.63862 | pred_wait_2_detect_2_form2_stage_1_cancers_5_posrisk_2_negrisk_10 | GPs | hypothetical |
| 0.282856 | 0.491391 | 0.701892 | pred_wait_2_detect_2_form2_stage_1_cancers_10_posrisk_2_negrisk_10 | GPs | hypothetical |
| 0.45484 | 0.672423 | 0.856132 | pred_wait_2_detect_2_form2_stage_1_cancers_25_posrisk_2_negrisk_10 | GPs | hypothetical |
| 0.056062 | 0.156536 | 0.284003 | pred_wait_2_detect_2_form2_stage_1_cancers_1_posrisk_2_negrisk_40 | GPs | hypothetical |
| 0.063175 | 0.172773 | 0.312522 | pred_wait_2_detect_2_form2_stage_1_cancers_5_posrisk_2_negrisk_40 | GPs | hypothetical |
| 0.075459 | 0.198764 | 0.355387 | pred_wait_2_detect_2_form2_stage_1_cancers_10_posrisk_2_negrisk_40 | GPs | hypothetical |
| 0.148482 | 0.323884 | 0.528296 | pred_wait_2_detect_2_form2_stage_1_cancers_25_posrisk_2_negrisk_40 | GPs | hypothetical |
| 0.109547 | 0.240304 | 0.394291 | pred_wait_2_detect_2_form2_stage_1_cancers_1_posrisk_4_negrisk_1 | GPs | hypothetical |
| 0.122251 | 0.26442 | 0.429584 | pred_wait_2_detect_2_form2_stage_1_cancers_5_posrisk_4_negrisk_1 | GPs | hypothetical |
| 0.142905 | 0.300534 | 0.480386 | pred_wait_2_detect_2_form2_stage_1_cancers_10_posrisk_4_negrisk_1 | GPs | hypothetical |
| 0.250066 | 0.446788 | 0.651691 | pred_wait_2_detect_2_form2_stage_1_cancers_25_posrisk_4_negrisk_1 | GPs | hypothetical |
| 0.087428 | 0.207584 | 0.353139 | pred_wait_2_detect_2_form2_stage_1_cancers_1_posrisk_4_negrisk_5 | GPs | hypothetical |
| 0.097824 | 0.228828 | 0.385715 | pred_wait_2_detect_2_form2_stage_1_cancers_5_posrisk_4_negrisk_5 | GPs | hypothetical |
| 0.114609 | 0.261381 | 0.434085 | pred_wait_2_detect_2_form2_stage_1_cancers_10_posrisk_4_negrisk_5 | GPs | hypothetical |
| 0.209791 | 0.401397 | 0.609054 | pred_wait_2_detect_2_form2_stage_1_cancers_25_posrisk_4_negrisk_5 | GPs | hypothetical |
| 0.066127 | 0.170744 | 0.304518 | pred_wait_2_detect_2_form2_stage_1_cancers_1_posrisk_4_negrisk_10 | GPs | hypothetical |
| 0.073137 | 0.188371 | 0.332958 | pred_wait_2_detect_2_form2_stage_1_cancers_5_posrisk_4_negrisk_10 | GPs | hypothetical |
| 0.086993 | 0.216151 | 0.376289 | pred_wait_2_detect_2_form2_stage_1_cancers_10_posrisk_4_negrisk_10 | GPs | hypothetical |
| 0.16464 | 0.345036 | 0.550102 | pred_wait_2_detect_2_form2_stage_1_cancers_25_posrisk_4_negrisk_10 | GPs | hypothetical |
| 0.011934 | 0.052231 | 0.115253 | pred_wait_2_detect_2_form2_stage_1_cancers_1_posrisk_4_negrisk_40 | GPs | hypothetical |
| 0.013272 | 0.056813 | 0.124409 | pred_wait_2_detect_2_form2_stage_1_cancers_5_posrisk_4_negrisk_40 | GPs | hypothetical |
| 0.015915 | 0.064978 | 0.140378 | pred_wait_2_detect_2_form2_stage_1_cancers_10_posrisk_4_negrisk_40 | GPs | hypothetical |
| 0.036407 | 0.11642 | 0.231362 | pred_wait_2_detect_2_form2_stage_1_cancers_25_posrisk_4_negrisk_40 | GPs | hypothetical |
| 0.04662 | 0.120473 | 0.217205 | pred_wait_2_detect_2_form2_stage_1_cancers_1_posrisk_6_negrisk_1 | GPs | hypothetical |
| 0.052975 | 0.131826 | 0.233437 | pred_wait_2_detect_2_form2_stage_1_cancers_5_posrisk_6_negrisk_1 | GPs | hypothetical |
| 0.061284 | 0.14935 | 0.259542 | pred_wait_2_detect_2_form2_stage_1_cancers_10_posrisk_6_negrisk_1 | GPs | hypothetical |
| 0.109005 | 0.22944 | 0.369791 | pred_wait_2_detect_2_form2_stage_1_cancers_25_posrisk_6_negrisk_1 | GPs | hypothetical |
| 0.036111 | 0.102505 | 0.191399 | pred_wait_2_detect_2_form2_stage_1_cancers_1_posrisk_6_negrisk_5 | GPs | hypothetical |
| 0.041064 | 0.112244 | 0.20671 | pred_wait_2_detect_2_form2_stage_1_cancers_5_posrisk_6_negrisk_5 | GPs | hypothetical |
| 0.04866 | 0.127537 | 0.22989 | pred_wait_2_detect_2_form2_stage_1_cancers_10_posrisk_6_negrisk_5 | GPs | hypothetical |
| 0.088753 | 0.200367 | 0.333966 | pred_wait_2_detect_2_form2_stage_1_cancers_25_posrisk_6_negrisk_5 | GPs | hypothetical |
| 0.026075 | 0.083252 | 0.161633 | pred_wait_2_detect_2_form2_stage_1_cancers_1_posrisk_6_negrisk_10 | GPs | hypothetical |
| 0.029829 | 0.091163 | 0.176037 | pred_wait_2_detect_2_form2_stage_1_cancers_5_posrisk_6_negrisk_10 | GPs | hypothetical |
| 0.035325 | 0.103858 | 0.195946 | pred_wait_2_detect_2_form2_stage_1_cancers_10_posrisk_6_negrisk_10 | GPs | hypothetical |
| 0.068687 | 0.167477 | 0.28956 | pred_wait_2_detect_2_form2_stage_1_cancers_25_posrisk_6_negrisk_10 | GPs | hypothetical |
| 0.004109 | 0.026114 | 0.064679 | pred_wait_2_detect_2_form2_stage_1_cancers_1_posrisk_6_negrisk_40 | GPs | hypothetical |
| 0.004675 | 0.02827 | 0.068103 | pred_wait_2_detect_2_form2_stage_1_cancers_5_posrisk_6_negrisk_40 | GPs | hypothetical |
| 0.005886 | 0.032088 | 0.07588 | pred_wait_2_detect_2_form2_stage_1_cancers_10_posrisk_6_negrisk_40 | GPs | hypothetical |
| 0.013687 | 0.055808 | 0.118819 | pred_wait_2_detect_2_form2_stage_1_cancers_25_posrisk_6_negrisk_40 | GPs | hypothetical |
| 0.026531 | 0.077031 | 0.14247 | pred_wait_2_detect_2_form2_stage_1_cancers_1_posrisk_8_negrisk_1 | GPs | hypothetical |
| 0.030154 | 0.083752 | 0.151369 | pred_wait_2_detect_2_form2_stage_1_cancers_5_posrisk_8_negrisk_1 | GPs | hypothetical |
| 0.035947 | 0.093946 | 0.167053 | pred_wait_2_detect_2_form2_stage_1_cancers_10_posrisk_8_negrisk_1 | GPs | hypothetical |
| 0.062542 | 0.139674 | 0.230739 | pred_wait_2_detect_2_form2_stage_1_cancers_25_posrisk_8_negrisk_1 | GPs | hypothetical |
| 0.020422 | 0.066498 | 0.126566 | pred_wait_2_detect_2_form2_stage_1_cancers_1_posrisk_8_negrisk_5 | GPs | hypothetical |
| 0.023755 | 0.072357 | 0.134879 | pred_wait_2_detect_2_form2_stage_1_cancers_5_posrisk_8_negrisk_5 | GPs | hypothetical |
| 0.02876 | 0.081356 | 0.148523 | pred_wait_2_detect_2_form2_stage_1_cancers_10_posrisk_8_negrisk_5 | GPs | hypothetical |
| 0.052063 | 0.122804 | 0.20864 | pred_wait_2_detect_2_form2_stage_1_cancers_25_posrisk_8_negrisk_5 | GPs | hypothetical |
| 0.014617 | 0.055196 | 0.108952 | pred_wait_2_detect_2_form2_stage_1_cancers_1_posrisk_8_negrisk_10 | GPs | hypothetical |
| 0.016834 | 0.060081 | 0.117257 | pred_wait_2_detect_2_form2_stage_1_cancers_5_posrisk_8_negrisk_10 | GPs | hypothetical |
| 0.020966 | 0.067707 | 0.129194 | pred_wait_2_detect_2_form2_stage_1_cancers_10_posrisk_8_negrisk_10 | GPs | hypothetical |
| 0.040592 | 0.104061 | 0.182106 | pred_wait_2_detect_2_form2_stage_1_cancers_25_posrisk_8_negrisk_10 | GPs | hypothetical |
| 0.002123 | 0.01975 | 0.050845 | pred_wait_2_detect_2_form2_stage_1_cancers_1_posrisk_8_negrisk_40 | GPs | hypothetical |
| 0.002481 | 0.021388 | 0.053261 | pred_wait_2_detect_2_form2_stage_1_cancers_5_posrisk_8_negrisk_40 | GPs | hypothetical |
| 0.003279 | 0.024122 | 0.058531 | pred_wait_2_detect_2_form2_stage_1_cancers_10_posrisk_8_negrisk_40 | GPs | hypothetical |
| 0.008088 | 0.039314 | 0.083522 | pred_wait_2_detect_2_form2_stage_1_cancers_25_posrisk_8_negrisk_40 | GPs | hypothetical |
| 0.303987 | 0.49117 | 0.680335 | pred_wait_2_detect_2_form2_stage_2_cancers_1_posrisk_2_negrisk_1 | GPs | hypothetical |
| 0.337389 | 0.533724 | 0.725643 | pred_wait_2_detect_2_form2_stage_2_cancers_5_posrisk_2_negrisk_1 | GPs | hypothetical |
| 0.386562 | 0.590536 | 0.781397 | pred_wait_2_detect_2_form2_stage_2_cancers_10_posrisk_2_negrisk_1 | GPs | hypothetical |
| 0.56791 | 0.754595 | 0.901387 | pred_wait_2_detect_2_form2_stage_2_cancers_25_posrisk_2_negrisk_1 | GPs | hypothetical |
| 0.264107 | 0.449719 | 0.640976 | pred_wait_2_detect_2_form2_stage_2_cancers_1_posrisk_2_negrisk_5 | GPs | hypothetical |
| 0.294761 | 0.491222 | 0.689509 | pred_wait_2_detect_2_form2_stage_2_cancers_5_posrisk_2_negrisk_5 | GPs | hypothetical |
| 0.339423 | 0.547987 | 0.749008 | pred_wait_2_detect_2_form2_stage_2_cancers_10_posrisk_2_negrisk_5 | GPs | hypothetical |
| 0.519522 | 0.721161 | 0.884091 | pred_wait_2_detect_2_form2_stage_2_cancers_25_posrisk_2_negrisk_5 | GPs | hypothetical |
| 0.216659 | 0.397211 | 0.589637 | pred_wait_2_detect_2_form2_stage_2_cancers_1_posrisk_2_negrisk_10 | GPs | hypothetical |
| 0.243033 | 0.436297 | 0.63862 | pred_wait_2_detect_2_form2_stage_2_cancers_5_posrisk_2_negrisk_10 | GPs | hypothetical |
| 0.282856 | 0.491391 | 0.701892 | pred_wait_2_detect_2_form2_stage_2_cancers_10_posrisk_2_negrisk_10 | GPs | hypothetical |
| 0.45484 | 0.672423 | 0.856132 | pred_wait_2_detect_2_form2_stage_2_cancers_25_posrisk_2_negrisk_10 | GPs | hypothetical |
| 0.056062 | 0.156536 | 0.284003 | pred_wait_2_detect_2_form2_stage_2_cancers_1_posrisk_2_negrisk_40 | GPs | hypothetical |
| 0.063175 | 0.172773 | 0.312522 | pred_wait_2_detect_2_form2_stage_2_cancers_5_posrisk_2_negrisk_40 | GPs | hypothetical |
| 0.075459 | 0.198764 | 0.355387 | pred_wait_2_detect_2_form2_stage_2_cancers_10_posrisk_2_negrisk_40 | GPs | hypothetical |
| 0.148482 | 0.323884 | 0.528296 | pred_wait_2_detect_2_form2_stage_2_cancers_25_posrisk_2_negrisk_40 | GPs | hypothetical |
| 0.109547 | 0.240304 | 0.394291 | pred_wait_2_detect_2_form2_stage_2_cancers_1_posrisk_4_negrisk_1 | GPs | hypothetical |
| 0.122251 | 0.26442 | 0.429584 | pred_wait_2_detect_2_form2_stage_2_cancers_5_posrisk_4_negrisk_1 | GPs | hypothetical |
| 0.142905 | 0.300534 | 0.480386 | pred_wait_2_detect_2_form2_stage_2_cancers_10_posrisk_4_negrisk_1 | GPs | hypothetical |
| 0.250066 | 0.446788 | 0.651691 | pred_wait_2_detect_2_form2_stage_2_cancers_25_posrisk_4_negrisk_1 | GPs | hypothetical |
| 0.087428 | 0.207584 | 0.353139 | pred_wait_2_detect_2_form2_stage_2_cancers_1_posrisk_4_negrisk_5 | GPs | hypothetical |
| 0.097824 | 0.228828 | 0.385715 | pred_wait_2_detect_2_form2_stage_2_cancers_5_posrisk_4_negrisk_5 | GPs | hypothetical |
| 0.114609 | 0.261381 | 0.434085 | pred_wait_2_detect_2_form2_stage_2_cancers_10_posrisk_4_negrisk_5 | GPs | hypothetical |
| 0.209791 | 0.401397 | 0.609054 | pred_wait_2_detect_2_form2_stage_2_cancers_25_posrisk_4_negrisk_5 | GPs | hypothetical |
| 0.066127 | 0.170744 | 0.304518 | pred_wait_2_detect_2_form2_stage_2_cancers_1_posrisk_4_negrisk_10 | GPs | hypothetical |
| 0.073137 | 0.188371 | 0.332958 | pred_wait_2_detect_2_form2_stage_2_cancers_5_posrisk_4_negrisk_10 | GPs | hypothetical |
| 0.086993 | 0.216151 | 0.376289 | pred_wait_2_detect_2_form2_stage_2_cancers_10_posrisk_4_negrisk_10 | GPs | hypothetical |
| 0.16464 | 0.345036 | 0.550102 | pred_wait_2_detect_2_form2_stage_2_cancers_25_posrisk_4_negrisk_10 | GPs | hypothetical |
| 0.011934 | 0.052231 | 0.115253 | pred_wait_2_detect_2_form2_stage_2_cancers_1_posrisk_4_negrisk_40 | GPs | hypothetical |
| 0.013272 | 0.056813 | 0.124409 | pred_wait_2_detect_2_form2_stage_2_cancers_5_posrisk_4_negrisk_40 | GPs | hypothetical |
| 0.015915 | 0.064978 | 0.140378 | pred_wait_2_detect_2_form2_stage_2_cancers_10_posrisk_4_negrisk_40 | GPs | hypothetical |
| 0.036407 | 0.11642 | 0.231362 | pred_wait_2_detect_2_form2_stage_2_cancers_25_posrisk_4_negrisk_40 | GPs | hypothetical |
| 0.04662 | 0.120473 | 0.217205 | pred_wait_2_detect_2_form2_stage_2_cancers_1_posrisk_6_negrisk_1 | GPs | hypothetical |
| 0.052975 | 0.131826 | 0.233437 | pred_wait_2_detect_2_form2_stage_2_cancers_5_posrisk_6_negrisk_1 | GPs | hypothetical |
| 0.061284 | 0.14935 | 0.259542 | pred_wait_2_detect_2_form2_stage_2_cancers_10_posrisk_6_negrisk_1 | GPs | hypothetical |
| 0.109005 | 0.22944 | 0.369791 | pred_wait_2_detect_2_form2_stage_2_cancers_25_posrisk_6_negrisk_1 | GPs | hypothetical |
| 0.036111 | 0.102505 | 0.191399 | pred_wait_2_detect_2_form2_stage_2_cancers_1_posrisk_6_negrisk_5 | GPs | hypothetical |
| 0.041064 | 0.112244 | 0.20671 | pred_wait_2_detect_2_form2_stage_2_cancers_5_posrisk_6_negrisk_5 | GPs | hypothetical |
| 0.04866 | 0.127537 | 0.22989 | pred_wait_2_detect_2_form2_stage_2_cancers_10_posrisk_6_negrisk_5 | GPs | hypothetical |
| 0.088753 | 0.200367 | 0.333966 | pred_wait_2_detect_2_form2_stage_2_cancers_25_posrisk_6_negrisk_5 | GPs | hypothetical |
| 0.026075 | 0.083252 | 0.161633 | pred_wait_2_detect_2_form2_stage_2_cancers_1_posrisk_6_negrisk_10 | GPs | hypothetical |
| 0.029829 | 0.091163 | 0.176037 | pred_wait_2_detect_2_form2_stage_2_cancers_5_posrisk_6_negrisk_10 | GPs | hypothetical |
| 0.035325 | 0.103858 | 0.195946 | pred_wait_2_detect_2_form2_stage_2_cancers_10_posrisk_6_negrisk_10 | GPs | hypothetical |
| 0.068687 | 0.167477 | 0.28956 | pred_wait_2_detect_2_form2_stage_2_cancers_25_posrisk_6_negrisk_10 | GPs | hypothetical |
| 0.004109 | 0.026114 | 0.064679 | pred_wait_2_detect_2_form2_stage_2_cancers_1_posrisk_6_negrisk_40 | GPs | hypothetical |
| 0.004675 | 0.02827 | 0.068103 | pred_wait_2_detect_2_form2_stage_2_cancers_5_posrisk_6_negrisk_40 | GPs | hypothetical |
| 0.005886 | 0.032088 | 0.07588 | pred_wait_2_detect_2_form2_stage_2_cancers_10_posrisk_6_negrisk_40 | GPs | hypothetical |
| 0.013687 | 0.055808 | 0.118819 | pred_wait_2_detect_2_form2_stage_2_cancers_25_posrisk_6_negrisk_40 | GPs | hypothetical |
| 0.026531 | 0.077031 | 0.14247 | pred_wait_2_detect_2_form2_stage_2_cancers_1_posrisk_8_negrisk_1 | GPs | hypothetical |
| 0.030154 | 0.083752 | 0.151369 | pred_wait_2_detect_2_form2_stage_2_cancers_5_posrisk_8_negrisk_1 | GPs | hypothetical |
| 0.035947 | 0.093946 | 0.167053 | pred_wait_2_detect_2_form2_stage_2_cancers_10_posrisk_8_negrisk_1 | GPs | hypothetical |
| 0.062542 | 0.139674 | 0.230739 | pred_wait_2_detect_2_form2_stage_2_cancers_25_posrisk_8_negrisk_1 | GPs | hypothetical |
| 0.020422 | 0.066498 | 0.126566 | pred_wait_2_detect_2_form2_stage_2_cancers_1_posrisk_8_negrisk_5 | GPs | hypothetical |
| 0.023755 | 0.072357 | 0.134879 | pred_wait_2_detect_2_form2_stage_2_cancers_5_posrisk_8_negrisk_5 | GPs | hypothetical |
| 0.02876 | 0.081356 | 0.148523 | pred_wait_2_detect_2_form2_stage_2_cancers_10_posrisk_8_negrisk_5 | GPs | hypothetical |
| 0.052063 | 0.122804 | 0.20864 | pred_wait_2_detect_2_form2_stage_2_cancers_25_posrisk_8_negrisk_5 | GPs | hypothetical |
| 0.014617 | 0.055196 | 0.108952 | pred_wait_2_detect_2_form2_stage_2_cancers_1_posrisk_8_negrisk_10 | GPs | hypothetical |
| 0.016834 | 0.060081 | 0.117257 | pred_wait_2_detect_2_form2_stage_2_cancers_5_posrisk_8_negrisk_10 | GPs | hypothetical |
| 0.020966 | 0.067707 | 0.129194 | pred_wait_2_detect_2_form2_stage_2_cancers_10_posrisk_8_negrisk_10 | GPs | hypothetical |
| 0.040592 | 0.104061 | 0.182106 | pred_wait_2_detect_2_form2_stage_2_cancers_25_posrisk_8_negrisk_10 | GPs | hypothetical |
| 0.002123 | 0.01975 | 0.050845 | pred_wait_2_detect_2_form2_stage_2_cancers_1_posrisk_8_negrisk_40 | GPs | hypothetical |
| 0.002481 | 0.021388 | 0.053261 | pred_wait_2_detect_2_form2_stage_2_cancers_5_posrisk_8_negrisk_40 | GPs | hypothetical |
| 0.003279 | 0.024122 | 0.058531 | pred_wait_2_detect_2_form2_stage_2_cancers_10_posrisk_8_negrisk_40 | GPs | hypothetical |
| 0.008088 | 0.039314 | 0.083522 | pred_wait_2_detect_2_form2_stage_2_cancers_25_posrisk_8_negrisk_40 | GPs | hypothetical |
| 0.303987 | 0.49117 | 0.680335 | pred_wait_2_detect_2_form3_stage_1_cancers_1_posrisk_2_negrisk_1 | GPs | hypothetical |
| 0.337389 | 0.533724 | 0.725643 | pred_wait_2_detect_2_form3_stage_1_cancers_5_posrisk_2_negrisk_1 | GPs | hypothetical |
| 0.386562 | 0.590536 | 0.781397 | pred_wait_2_detect_2_form3_stage_1_cancers_10_posrisk_2_negrisk_1 | GPs | hypothetical |
| 0.56791 | 0.754595 | 0.901387 | pred_wait_2_detect_2_form3_stage_1_cancers_25_posrisk_2_negrisk_1 | GPs | hypothetical |
| 0.264107 | 0.449719 | 0.640976 | pred_wait_2_detect_2_form3_stage_1_cancers_1_posrisk_2_negrisk_5 | GPs | hypothetical |
| 0.294761 | 0.491222 | 0.689509 | pred_wait_2_detect_2_form3_stage_1_cancers_5_posrisk_2_negrisk_5 | GPs | hypothetical |
| 0.339423 | 0.547987 | 0.749008 | pred_wait_2_detect_2_form3_stage_1_cancers_10_posrisk_2_negrisk_5 | GPs | hypothetical |
| 0.519522 | 0.721161 | 0.884091 | pred_wait_2_detect_2_form3_stage_1_cancers_25_posrisk_2_negrisk_5 | GPs | hypothetical |
| 0.216659 | 0.397211 | 0.589637 | pred_wait_2_detect_2_form3_stage_1_cancers_1_posrisk_2_negrisk_10 | GPs | hypothetical |
| 0.243033 | 0.436297 | 0.63862 | pred_wait_2_detect_2_form3_stage_1_cancers_5_posrisk_2_negrisk_10 | GPs | hypothetical |
| 0.282856 | 0.491391 | 0.701892 | pred_wait_2_detect_2_form3_stage_1_cancers_10_posrisk_2_negrisk_10 | GPs | hypothetical |
| 0.45484 | 0.672423 | 0.856132 | pred_wait_2_detect_2_form3_stage_1_cancers_25_posrisk_2_negrisk_10 | GPs | hypothetical |
| 0.056062 | 0.156536 | 0.284003 | pred_wait_2_detect_2_form3_stage_1_cancers_1_posrisk_2_negrisk_40 | GPs | hypothetical |
| 0.063175 | 0.172773 | 0.312522 | pred_wait_2_detect_2_form3_stage_1_cancers_5_posrisk_2_negrisk_40 | GPs | hypothetical |
| 0.075459 | 0.198764 | 0.355387 | pred_wait_2_detect_2_form3_stage_1_cancers_10_posrisk_2_negrisk_40 | GPs | hypothetical |
| 0.148482 | 0.323884 | 0.528296 | pred_wait_2_detect_2_form3_stage_1_cancers_25_posrisk_2_negrisk_40 | GPs | hypothetical |
| 0.109547 | 0.240304 | 0.394291 | pred_wait_2_detect_2_form3_stage_1_cancers_1_posrisk_4_negrisk_1 | GPs | hypothetical |
| 0.122251 | 0.26442 | 0.429584 | pred_wait_2_detect_2_form3_stage_1_cancers_5_posrisk_4_negrisk_1 | GPs | hypothetical |
| 0.142905 | 0.300534 | 0.480386 | pred_wait_2_detect_2_form3_stage_1_cancers_10_posrisk_4_negrisk_1 | GPs | hypothetical |
| 0.250066 | 0.446788 | 0.651691 | pred_wait_2_detect_2_form3_stage_1_cancers_25_posrisk_4_negrisk_1 | GPs | hypothetical |
| 0.087428 | 0.207584 | 0.353139 | pred_wait_2_detect_2_form3_stage_1_cancers_1_posrisk_4_negrisk_5 | GPs | hypothetical |
| 0.097824 | 0.228828 | 0.385715 | pred_wait_2_detect_2_form3_stage_1_cancers_5_posrisk_4_negrisk_5 | GPs | hypothetical |
| 0.114609 | 0.261381 | 0.434085 | pred_wait_2_detect_2_form3_stage_1_cancers_10_posrisk_4_negrisk_5 | GPs | hypothetical |
| 0.209791 | 0.401397 | 0.609054 | pred_wait_2_detect_2_form3_stage_1_cancers_25_posrisk_4_negrisk_5 | GPs | hypothetical |
| 0.066127 | 0.170744 | 0.304518 | pred_wait_2_detect_2_form3_stage_1_cancers_1_posrisk_4_negrisk_10 | GPs | hypothetical |
| 0.073137 | 0.188371 | 0.332958 | pred_wait_2_detect_2_form3_stage_1_cancers_5_posrisk_4_negrisk_10 | GPs | hypothetical |
| 0.086993 | 0.216151 | 0.376289 | pred_wait_2_detect_2_form3_stage_1_cancers_10_posrisk_4_negrisk_10 | GPs | hypothetical |
| 0.16464 | 0.345036 | 0.550102 | pred_wait_2_detect_2_form3_stage_1_cancers_25_posrisk_4_negrisk_10 | GPs | hypothetical |
| 0.011934 | 0.052231 | 0.115253 | pred_wait_2_detect_2_form3_stage_1_cancers_1_posrisk_4_negrisk_40 | GPs | hypothetical |
| 0.013272 | 0.056813 | 0.124409 | pred_wait_2_detect_2_form3_stage_1_cancers_5_posrisk_4_negrisk_40 | GPs | hypothetical |
| 0.015915 | 0.064978 | 0.140378 | pred_wait_2_detect_2_form3_stage_1_cancers_10_posrisk_4_negrisk_40 | GPs | hypothetical |
| 0.036407 | 0.11642 | 0.231362 | pred_wait_2_detect_2_form3_stage_1_cancers_25_posrisk_4_negrisk_40 | GPs | hypothetical |
| 0.04662 | 0.120473 | 0.217205 | pred_wait_2_detect_2_form3_stage_1_cancers_1_posrisk_6_negrisk_1 | GPs | hypothetical |
| 0.052975 | 0.131826 | 0.233437 | pred_wait_2_detect_2_form3_stage_1_cancers_5_posrisk_6_negrisk_1 | GPs | hypothetical |
| 0.061284 | 0.14935 | 0.259542 | pred_wait_2_detect_2_form3_stage_1_cancers_10_posrisk_6_negrisk_1 | GPs | hypothetical |
| 0.109005 | 0.22944 | 0.369791 | pred_wait_2_detect_2_form3_stage_1_cancers_25_posrisk_6_negrisk_1 | GPs | hypothetical |
| 0.036111 | 0.102505 | 0.191399 | pred_wait_2_detect_2_form3_stage_1_cancers_1_posrisk_6_negrisk_5 | GPs | hypothetical |
| 0.041064 | 0.112244 | 0.20671 | pred_wait_2_detect_2_form3_stage_1_cancers_5_posrisk_6_negrisk_5 | GPs | hypothetical |
| 0.04866 | 0.127537 | 0.22989 | pred_wait_2_detect_2_form3_stage_1_cancers_10_posrisk_6_negrisk_5 | GPs | hypothetical |
| 0.088753 | 0.200367 | 0.333966 | pred_wait_2_detect_2_form3_stage_1_cancers_25_posrisk_6_negrisk_5 | GPs | hypothetical |
| 0.026075 | 0.083252 | 0.161633 | pred_wait_2_detect_2_form3_stage_1_cancers_1_posrisk_6_negrisk_10 | GPs | hypothetical |
| 0.029829 | 0.091163 | 0.176037 | pred_wait_2_detect_2_form3_stage_1_cancers_5_posrisk_6_negrisk_10 | GPs | hypothetical |
| 0.035325 | 0.103858 | 0.195946 | pred_wait_2_detect_2_form3_stage_1_cancers_10_posrisk_6_negrisk_10 | GPs | hypothetical |
| 0.068687 | 0.167477 | 0.28956 | pred_wait_2_detect_2_form3_stage_1_cancers_25_posrisk_6_negrisk_10 | GPs | hypothetical |
| 0.004109 | 0.026114 | 0.064679 | pred_wait_2_detect_2_form3_stage_1_cancers_1_posrisk_6_negrisk_40 | GPs | hypothetical |
| 0.004675 | 0.02827 | 0.068103 | pred_wait_2_detect_2_form3_stage_1_cancers_5_posrisk_6_negrisk_40 | GPs | hypothetical |
| 0.005886 | 0.032088 | 0.07588 | pred_wait_2_detect_2_form3_stage_1_cancers_10_posrisk_6_negrisk_40 | GPs | hypothetical |
| 0.013687 | 0.055808 | 0.118819 | pred_wait_2_detect_2_form3_stage_1_cancers_25_posrisk_6_negrisk_40 | GPs | hypothetical |
| 0.026531 | 0.077031 | 0.14247 | pred_wait_2_detect_2_form3_stage_1_cancers_1_posrisk_8_negrisk_1 | GPs | hypothetical |
| 0.030154 | 0.083752 | 0.151369 | pred_wait_2_detect_2_form3_stage_1_cancers_5_posrisk_8_negrisk_1 | GPs | hypothetical |
| 0.035947 | 0.093946 | 0.167053 | pred_wait_2_detect_2_form3_stage_1_cancers_10_posrisk_8_negrisk_1 | GPs | hypothetical |
| 0.062542 | 0.139674 | 0.230739 | pred_wait_2_detect_2_form3_stage_1_cancers_25_posrisk_8_negrisk_1 | GPs | hypothetical |
| 0.020422 | 0.066498 | 0.126566 | pred_wait_2_detect_2_form3_stage_1_cancers_1_posrisk_8_negrisk_5 | GPs | hypothetical |
| 0.023755 | 0.072357 | 0.134879 | pred_wait_2_detect_2_form3_stage_1_cancers_5_posrisk_8_negrisk_5 | GPs | hypothetical |
| 0.02876 | 0.081356 | 0.148523 | pred_wait_2_detect_2_form3_stage_1_cancers_10_posrisk_8_negrisk_5 | GPs | hypothetical |
| 0.052063 | 0.122804 | 0.20864 | pred_wait_2_detect_2_form3_stage_1_cancers_25_posrisk_8_negrisk_5 | GPs | hypothetical |
| 0.014617 | 0.055196 | 0.108952 | pred_wait_2_detect_2_form3_stage_1_cancers_1_posrisk_8_negrisk_10 | GPs | hypothetical |
| 0.016834 | 0.060081 | 0.117257 | pred_wait_2_detect_2_form3_stage_1_cancers_5_posrisk_8_negrisk_10 | GPs | hypothetical |
| 0.020966 | 0.067707 | 0.129194 | pred_wait_2_detect_2_form3_stage_1_cancers_10_posrisk_8_negrisk_10 | GPs | hypothetical |
| 0.040592 | 0.104061 | 0.182106 | pred_wait_2_detect_2_form3_stage_1_cancers_25_posrisk_8_negrisk_10 | GPs | hypothetical |
| 0.002123 | 0.01975 | 0.050845 | pred_wait_2_detect_2_form3_stage_1_cancers_1_posrisk_8_negrisk_40 | GPs | hypothetical |
| 0.002481 | 0.021388 | 0.053261 | pred_wait_2_detect_2_form3_stage_1_cancers_5_posrisk_8_negrisk_40 | GPs | hypothetical |
| 0.003279 | 0.024122 | 0.058531 | pred_wait_2_detect_2_form3_stage_1_cancers_10_posrisk_8_negrisk_40 | GPs | hypothetical |
| 0.008088 | 0.039314 | 0.083522 | pred_wait_2_detect_2_form3_stage_1_cancers_25_posrisk_8_negrisk_40 | GPs | hypothetical |
| 0.303987 | 0.49117 | 0.680335 | pred_wait_2_detect_2_form3_stage_2_cancers_1_posrisk_2_negrisk_1 | GPs | hypothetical |
| 0.337389 | 0.533724 | 0.725643 | pred_wait_2_detect_2_form3_stage_2_cancers_5_posrisk_2_negrisk_1 | GPs | hypothetical |
| 0.386562 | 0.590536 | 0.781397 | pred_wait_2_detect_2_form3_stage_2_cancers_10_posrisk_2_negrisk_1 | GPs | hypothetical |
| 0.56791 | 0.754595 | 0.901387 | pred_wait_2_detect_2_form3_stage_2_cancers_25_posrisk_2_negrisk_1 | GPs | hypothetical |
| 0.264107 | 0.449719 | 0.640976 | pred_wait_2_detect_2_form3_stage_2_cancers_1_posrisk_2_negrisk_5 | GPs | hypothetical |
| 0.294761 | 0.491222 | 0.689509 | pred_wait_2_detect_2_form3_stage_2_cancers_5_posrisk_2_negrisk_5 | GPs | hypothetical |
| 0.339423 | 0.547987 | 0.749008 | pred_wait_2_detect_2_form3_stage_2_cancers_10_posrisk_2_negrisk_5 | GPs | hypothetical |
| 0.519522 | 0.721161 | 0.884091 | pred_wait_2_detect_2_form3_stage_2_cancers_25_posrisk_2_negrisk_5 | GPs | hypothetical |
| 0.216659 | 0.397211 | 0.589637 | pred_wait_2_detect_2_form3_stage_2_cancers_1_posrisk_2_negrisk_10 | GPs | hypothetical |
| 0.243033 | 0.436297 | 0.63862 | pred_wait_2_detect_2_form3_stage_2_cancers_5_posrisk_2_negrisk_10 | GPs | hypothetical |
| 0.282856 | 0.491391 | 0.701892 | pred_wait_2_detect_2_form3_stage_2_cancers_10_posrisk_2_negrisk_10 | GPs | hypothetical |
| 0.45484 | 0.672423 | 0.856132 | pred_wait_2_detect_2_form3_stage_2_cancers_25_posrisk_2_negrisk_10 | GPs | hypothetical |
| 0.056062 | 0.156536 | 0.284003 | pred_wait_2_detect_2_form3_stage_2_cancers_1_posrisk_2_negrisk_40 | GPs | hypothetical |
| 0.063175 | 0.172773 | 0.312522 | pred_wait_2_detect_2_form3_stage_2_cancers_5_posrisk_2_negrisk_40 | GPs | hypothetical |
| 0.075459 | 0.198764 | 0.355387 | pred_wait_2_detect_2_form3_stage_2_cancers_10_posrisk_2_negrisk_40 | GPs | hypothetical |
| 0.148482 | 0.323884 | 0.528296 | pred_wait_2_detect_2_form3_stage_2_cancers_25_posrisk_2_negrisk_40 | GPs | hypothetical |
| 0.109547 | 0.240304 | 0.394291 | pred_wait_2_detect_2_form3_stage_2_cancers_1_posrisk_4_negrisk_1 | GPs | hypothetical |
| 0.122251 | 0.26442 | 0.429584 | pred_wait_2_detect_2_form3_stage_2_cancers_5_posrisk_4_negrisk_1 | GPs | hypothetical |
| 0.142905 | 0.300534 | 0.480386 | pred_wait_2_detect_2_form3_stage_2_cancers_10_posrisk_4_negrisk_1 | GPs | hypothetical |
| 0.250066 | 0.446788 | 0.651691 | pred_wait_2_detect_2_form3_stage_2_cancers_25_posrisk_4_negrisk_1 | GPs | hypothetical |
| 0.087428 | 0.207584 | 0.353139 | pred_wait_2_detect_2_form3_stage_2_cancers_1_posrisk_4_negrisk_5 | GPs | hypothetical |
| 0.097824 | 0.228828 | 0.385715 | pred_wait_2_detect_2_form3_stage_2_cancers_5_posrisk_4_negrisk_5 | GPs | hypothetical |
| 0.114609 | 0.261381 | 0.434085 | pred_wait_2_detect_2_form3_stage_2_cancers_10_posrisk_4_negrisk_5 | GPs | hypothetical |
| 0.209791 | 0.401397 | 0.609054 | pred_wait_2_detect_2_form3_stage_2_cancers_25_posrisk_4_negrisk_5 | GPs | hypothetical |
| 0.066127 | 0.170744 | 0.304518 | pred_wait_2_detect_2_form3_stage_2_cancers_1_posrisk_4_negrisk_10 | GPs | hypothetical |
| 0.073137 | 0.188371 | 0.332958 | pred_wait_2_detect_2_form3_stage_2_cancers_5_posrisk_4_negrisk_10 | GPs | hypothetical |
| 0.086993 | 0.216151 | 0.376289 | pred_wait_2_detect_2_form3_stage_2_cancers_10_posrisk_4_negrisk_10 | GPs | hypothetical |
| 0.16464 | 0.345036 | 0.550102 | pred_wait_2_detect_2_form3_stage_2_cancers_25_posrisk_4_negrisk_10 | GPs | hypothetical |
| 0.011934 | 0.052231 | 0.115253 | pred_wait_2_detect_2_form3_stage_2_cancers_1_posrisk_4_negrisk_40 | GPs | hypothetical |
| 0.013272 | 0.056813 | 0.124409 | pred_wait_2_detect_2_form3_stage_2_cancers_5_posrisk_4_negrisk_40 | GPs | hypothetical |
| 0.015915 | 0.064978 | 0.140378 | pred_wait_2_detect_2_form3_stage_2_cancers_10_posrisk_4_negrisk_40 | GPs | hypothetical |
| 0.036407 | 0.11642 | 0.231362 | pred_wait_2_detect_2_form3_stage_2_cancers_25_posrisk_4_negrisk_40 | GPs | hypothetical |
| 0.04662 | 0.120473 | 0.217205 | pred_wait_2_detect_2_form3_stage_2_cancers_1_posrisk_6_negrisk_1 | GPs | hypothetical |
| 0.052975 | 0.131826 | 0.233437 | pred_wait_2_detect_2_form3_stage_2_cancers_5_posrisk_6_negrisk_1 | GPs | hypothetical |
| 0.061284 | 0.14935 | 0.259542 | pred_wait_2_detect_2_form3_stage_2_cancers_10_posrisk_6_negrisk_1 | GPs | hypothetical |
| 0.109005 | 0.22944 | 0.369791 | pred_wait_2_detect_2_form3_stage_2_cancers_25_posrisk_6_negrisk_1 | GPs | hypothetical |
| 0.036111 | 0.102505 | 0.191399 | pred_wait_2_detect_2_form3_stage_2_cancers_1_posrisk_6_negrisk_5 | GPs | hypothetical |
| 0.041064 | 0.112244 | 0.20671 | pred_wait_2_detect_2_form3_stage_2_cancers_5_posrisk_6_negrisk_5 | GPs | hypothetical |
| 0.04866 | 0.127537 | 0.22989 | pred_wait_2_detect_2_form3_stage_2_cancers_10_posrisk_6_negrisk_5 | GPs | hypothetical |
| 0.088753 | 0.200367 | 0.333966 | pred_wait_2_detect_2_form3_stage_2_cancers_25_posrisk_6_negrisk_5 | GPs | hypothetical |
| 0.026075 | 0.083252 | 0.161633 | pred_wait_2_detect_2_form3_stage_2_cancers_1_posrisk_6_negrisk_10 | GPs | hypothetical |
| 0.029829 | 0.091163 | 0.176037 | pred_wait_2_detect_2_form3_stage_2_cancers_5_posrisk_6_negrisk_10 | GPs | hypothetical |
| 0.035325 | 0.103858 | 0.195946 | pred_wait_2_detect_2_form3_stage_2_cancers_10_posrisk_6_negrisk_10 | GPs | hypothetical |
| 0.068687 | 0.167477 | 0.28956 | pred_wait_2_detect_2_form3_stage_2_cancers_25_posrisk_6_negrisk_10 | GPs | hypothetical |
| 0.004109 | 0.026114 | 0.064679 | pred_wait_2_detect_2_form3_stage_2_cancers_1_posrisk_6_negrisk_40 | GPs | hypothetical |
| 0.004675 | 0.02827 | 0.068103 | pred_wait_2_detect_2_form3_stage_2_cancers_5_posrisk_6_negrisk_40 | GPs | hypothetical |
| 0.005886 | 0.032088 | 0.07588 | pred_wait_2_detect_2_form3_stage_2_cancers_10_posrisk_6_negrisk_40 | GPs | hypothetical |
| 0.013687 | 0.055808 | 0.118819 | pred_wait_2_detect_2_form3_stage_2_cancers_25_posrisk_6_negrisk_40 | GPs | hypothetical |
| 0.026531 | 0.077031 | 0.14247 | pred_wait_2_detect_2_form3_stage_2_cancers_1_posrisk_8_negrisk_1 | GPs | hypothetical |
| 0.030154 | 0.083752 | 0.151369 | pred_wait_2_detect_2_form3_stage_2_cancers_5_posrisk_8_negrisk_1 | GPs | hypothetical |
| 0.035947 | 0.093946 | 0.167053 | pred_wait_2_detect_2_form3_stage_2_cancers_10_posrisk_8_negrisk_1 | GPs | hypothetical |
| 0.062542 | 0.139674 | 0.230739 | pred_wait_2_detect_2_form3_stage_2_cancers_25_posrisk_8_negrisk_1 | GPs | hypothetical |
| 0.020422 | 0.066498 | 0.126566 | pred_wait_2_detect_2_form3_stage_2_cancers_1_posrisk_8_negrisk_5 | GPs | hypothetical |
| 0.023755 | 0.072357 | 0.134879 | pred_wait_2_detect_2_form3_stage_2_cancers_5_posrisk_8_negrisk_5 | GPs | hypothetical |
| 0.02876 | 0.081356 | 0.148523 | pred_wait_2_detect_2_form3_stage_2_cancers_10_posrisk_8_negrisk_5 | GPs | hypothetical |
| 0.052063 | 0.122804 | 0.20864 | pred_wait_2_detect_2_form3_stage_2_cancers_25_posrisk_8_negrisk_5 | GPs | hypothetical |
| 0.014617 | 0.055196 | 0.108952 | pred_wait_2_detect_2_form3_stage_2_cancers_1_posrisk_8_negrisk_10 | GPs | hypothetical |
| 0.016834 | 0.060081 | 0.117257 | pred_wait_2_detect_2_form3_stage_2_cancers_5_posrisk_8_negrisk_10 | GPs | hypothetical |
| 0.020966 | 0.067707 | 0.129194 | pred_wait_2_detect_2_form3_stage_2_cancers_10_posrisk_8_negrisk_10 | GPs | hypothetical |
| 0.040592 | 0.104061 | 0.182106 | pred_wait_2_detect_2_form3_stage_2_cancers_25_posrisk_8_negrisk_10 | GPs | hypothetical |
| 0.002123 | 0.01975 | 0.050845 | pred_wait_2_detect_2_form3_stage_2_cancers_1_posrisk_8_negrisk_40 | GPs | hypothetical |
| 0.002481 | 0.021388 | 0.053261 | pred_wait_2_detect_2_form3_stage_2_cancers_5_posrisk_8_negrisk_40 | GPs | hypothetical |
| 0.003279 | 0.024122 | 0.058531 | pred_wait_2_detect_2_form3_stage_2_cancers_10_posrisk_8_negrisk_40 | GPs | hypothetical |
| 0.008088 | 0.039314 | 0.083522 | pred_wait_2_detect_2_form3_stage_2_cancers_25_posrisk_8_negrisk_40 | GPs | hypothetical |
| 0.303987 | 0.49117 | 0.680335 | pred_wait_2_detect_2_form4_stage_1_cancers_1_posrisk_2_negrisk_1 | GPs | hypothetical |
| 0.337389 | 0.533724 | 0.725643 | pred_wait_2_detect_2_form4_stage_1_cancers_5_posrisk_2_negrisk_1 | GPs | hypothetical |
| 0.386562 | 0.590536 | 0.781397 | pred_wait_2_detect_2_form4_stage_1_cancers_10_posrisk_2_negrisk_1 | GPs | hypothetical |
| 0.56791 | 0.754595 | 0.901387 | pred_wait_2_detect_2_form4_stage_1_cancers_25_posrisk_2_negrisk_1 | GPs | hypothetical |
| 0.264107 | 0.449719 | 0.640976 | pred_wait_2_detect_2_form4_stage_1_cancers_1_posrisk_2_negrisk_5 | GPs | hypothetical |
| 0.294761 | 0.491222 | 0.689509 | pred_wait_2_detect_2_form4_stage_1_cancers_5_posrisk_2_negrisk_5 | GPs | hypothetical |
| 0.339423 | 0.547987 | 0.749008 | pred_wait_2_detect_2_form4_stage_1_cancers_10_posrisk_2_negrisk_5 | GPs | hypothetical |
| 0.519522 | 0.721161 | 0.884091 | pred_wait_2_detect_2_form4_stage_1_cancers_25_posrisk_2_negrisk_5 | GPs | hypothetical |
| 0.216659 | 0.397211 | 0.589637 | pred_wait_2_detect_2_form4_stage_1_cancers_1_posrisk_2_negrisk_10 | GPs | hypothetical |
| 0.243033 | 0.436297 | 0.63862 | pred_wait_2_detect_2_form4_stage_1_cancers_5_posrisk_2_negrisk_10 | GPs | hypothetical |
| 0.282856 | 0.491391 | 0.701892 | pred_wait_2_detect_2_form4_stage_1_cancers_10_posrisk_2_negrisk_10 | GPs | hypothetical |
| 0.45484 | 0.672423 | 0.856132 | pred_wait_2_detect_2_form4_stage_1_cancers_25_posrisk_2_negrisk_10 | GPs | hypothetical |
| 0.056062 | 0.156536 | 0.284003 | pred_wait_2_detect_2_form4_stage_1_cancers_1_posrisk_2_negrisk_40 | GPs | hypothetical |
| 0.063175 | 0.172773 | 0.312522 | pred_wait_2_detect_2_form4_stage_1_cancers_5_posrisk_2_negrisk_40 | GPs | hypothetical |
| 0.075459 | 0.198764 | 0.355387 | pred_wait_2_detect_2_form4_stage_1_cancers_10_posrisk_2_negrisk_40 | GPs | hypothetical |
| 0.148482 | 0.323884 | 0.528296 | pred_wait_2_detect_2_form4_stage_1_cancers_25_posrisk_2_negrisk_40 | GPs | hypothetical |
| 0.109547 | 0.240304 | 0.394291 | pred_wait_2_detect_2_form4_stage_1_cancers_1_posrisk_4_negrisk_1 | GPs | hypothetical |
| 0.122251 | 0.26442 | 0.429584 | pred_wait_2_detect_2_form4_stage_1_cancers_5_posrisk_4_negrisk_1 | GPs | hypothetical |
| 0.142905 | 0.300534 | 0.480386 | pred_wait_2_detect_2_form4_stage_1_cancers_10_posrisk_4_negrisk_1 | GPs | hypothetical |
| 0.250066 | 0.446788 | 0.651691 | pred_wait_2_detect_2_form4_stage_1_cancers_25_posrisk_4_negrisk_1 | GPs | hypothetical |
| 0.087428 | 0.207584 | 0.353139 | pred_wait_2_detect_2_form4_stage_1_cancers_1_posrisk_4_negrisk_5 | GPs | hypothetical |
| 0.097824 | 0.228828 | 0.385715 | pred_wait_2_detect_2_form4_stage_1_cancers_5_posrisk_4_negrisk_5 | GPs | hypothetical |
| 0.114609 | 0.261381 | 0.434085 | pred_wait_2_detect_2_form4_stage_1_cancers_10_posrisk_4_negrisk_5 | GPs | hypothetical |
| 0.209791 | 0.401397 | 0.609054 | pred_wait_2_detect_2_form4_stage_1_cancers_25_posrisk_4_negrisk_5 | GPs | hypothetical |
| 0.066127 | 0.170744 | 0.304518 | pred_wait_2_detect_2_form4_stage_1_cancers_1_posrisk_4_negrisk_10 | GPs | hypothetical |
| 0.073137 | 0.188371 | 0.332958 | pred_wait_2_detect_2_form4_stage_1_cancers_5_posrisk_4_negrisk_10 | GPs | hypothetical |
| 0.086993 | 0.216151 | 0.376289 | pred_wait_2_detect_2_form4_stage_1_cancers_10_posrisk_4_negrisk_10 | GPs | hypothetical |
| 0.16464 | 0.345036 | 0.550102 | pred_wait_2_detect_2_form4_stage_1_cancers_25_posrisk_4_negrisk_10 | GPs | hypothetical |
| 0.011934 | 0.052231 | 0.115253 | pred_wait_2_detect_2_form4_stage_1_cancers_1_posrisk_4_negrisk_40 | GPs | hypothetical |
| 0.013272 | 0.056813 | 0.124409 | pred_wait_2_detect_2_form4_stage_1_cancers_5_posrisk_4_negrisk_40 | GPs | hypothetical |
| 0.015915 | 0.064978 | 0.140378 | pred_wait_2_detect_2_form4_stage_1_cancers_10_posrisk_4_negrisk_40 | GPs | hypothetical |
| 0.036407 | 0.11642 | 0.231362 | pred_wait_2_detect_2_form4_stage_1_cancers_25_posrisk_4_negrisk_40 | GPs | hypothetical |
| 0.04662 | 0.120473 | 0.217205 | pred_wait_2_detect_2_form4_stage_1_cancers_1_posrisk_6_negrisk_1 | GPs | hypothetical |
| 0.052975 | 0.131826 | 0.233437 | pred_wait_2_detect_2_form4_stage_1_cancers_5_posrisk_6_negrisk_1 | GPs | hypothetical |
| 0.061284 | 0.14935 | 0.259542 | pred_wait_2_detect_2_form4_stage_1_cancers_10_posrisk_6_negrisk_1 | GPs | hypothetical |
| 0.109005 | 0.22944 | 0.369791 | pred_wait_2_detect_2_form4_stage_1_cancers_25_posrisk_6_negrisk_1 | GPs | hypothetical |
| 0.036111 | 0.102505 | 0.191399 | pred_wait_2_detect_2_form4_stage_1_cancers_1_posrisk_6_negrisk_5 | GPs | hypothetical |
| 0.041064 | 0.112244 | 0.20671 | pred_wait_2_detect_2_form4_stage_1_cancers_5_posrisk_6_negrisk_5 | GPs | hypothetical |
| 0.04866 | 0.127537 | 0.22989 | pred_wait_2_detect_2_form4_stage_1_cancers_10_posrisk_6_negrisk_5 | GPs | hypothetical |
| 0.088753 | 0.200367 | 0.333966 | pred_wait_2_detect_2_form4_stage_1_cancers_25_posrisk_6_negrisk_5 | GPs | hypothetical |
| 0.026075 | 0.083252 | 0.161633 | pred_wait_2_detect_2_form4_stage_1_cancers_1_posrisk_6_negrisk_10 | GPs | hypothetical |
| 0.029829 | 0.091163 | 0.176037 | pred_wait_2_detect_2_form4_stage_1_cancers_5_posrisk_6_negrisk_10 | GPs | hypothetical |
| 0.035325 | 0.103858 | 0.195946 | pred_wait_2_detect_2_form4_stage_1_cancers_10_posrisk_6_negrisk_10 | GPs | hypothetical |
| 0.068687 | 0.167477 | 0.28956 | pred_wait_2_detect_2_form4_stage_1_cancers_25_posrisk_6_negrisk_10 | GPs | hypothetical |
| 0.004109 | 0.026114 | 0.064679 | pred_wait_2_detect_2_form4_stage_1_cancers_1_posrisk_6_negrisk_40 | GPs | hypothetical |
| 0.004675 | 0.02827 | 0.068103 | pred_wait_2_detect_2_form4_stage_1_cancers_5_posrisk_6_negrisk_40 | GPs | hypothetical |
| 0.005886 | 0.032088 | 0.07588 | pred_wait_2_detect_2_form4_stage_1_cancers_10_posrisk_6_negrisk_40 | GPs | hypothetical |
| 0.013687 | 0.055808 | 0.118819 | pred_wait_2_detect_2_form4_stage_1_cancers_25_posrisk_6_negrisk_40 | GPs | hypothetical |
| 0.026531 | 0.077031 | 0.14247 | pred_wait_2_detect_2_form4_stage_1_cancers_1_posrisk_8_negrisk_1 | GPs | hypothetical |
| 0.030154 | 0.083752 | 0.151369 | pred_wait_2_detect_2_form4_stage_1_cancers_5_posrisk_8_negrisk_1 | GPs | hypothetical |
| 0.035947 | 0.093946 | 0.167053 | pred_wait_2_detect_2_form4_stage_1_cancers_10_posrisk_8_negrisk_1 | GPs | hypothetical |
| 0.062542 | 0.139674 | 0.230739 | pred_wait_2_detect_2_form4_stage_1_cancers_25_posrisk_8_negrisk_1 | GPs | hypothetical |
| 0.020422 | 0.066498 | 0.126566 | pred_wait_2_detect_2_form4_stage_1_cancers_1_posrisk_8_negrisk_5 | GPs | hypothetical |
| 0.023755 | 0.072357 | 0.134879 | pred_wait_2_detect_2_form4_stage_1_cancers_5_posrisk_8_negrisk_5 | GPs | hypothetical |
| 0.02876 | 0.081356 | 0.148523 | pred_wait_2_detect_2_form4_stage_1_cancers_10_posrisk_8_negrisk_5 | GPs | hypothetical |
| 0.052063 | 0.122804 | 0.20864 | pred_wait_2_detect_2_form4_stage_1_cancers_25_posrisk_8_negrisk_5 | GPs | hypothetical |
| 0.014617 | 0.055196 | 0.108952 | pred_wait_2_detect_2_form4_stage_1_cancers_1_posrisk_8_negrisk_10 | GPs | hypothetical |
| 0.016834 | 0.060081 | 0.117257 | pred_wait_2_detect_2_form4_stage_1_cancers_5_posrisk_8_negrisk_10 | GPs | hypothetical |
| 0.020966 | 0.067707 | 0.129194 | pred_wait_2_detect_2_form4_stage_1_cancers_10_posrisk_8_negrisk_10 | GPs | hypothetical |
| 0.040592 | 0.104061 | 0.182106 | pred_wait_2_detect_2_form4_stage_1_cancers_25_posrisk_8_negrisk_10 | GPs | hypothetical |
| 0.002123 | 0.01975 | 0.050845 | pred_wait_2_detect_2_form4_stage_1_cancers_1_posrisk_8_negrisk_40 | GPs | hypothetical |
| 0.002481 | 0.021388 | 0.053261 | pred_wait_2_detect_2_form4_stage_1_cancers_5_posrisk_8_negrisk_40 | GPs | hypothetical |
| 0.003279 | 0.024122 | 0.058531 | pred_wait_2_detect_2_form4_stage_1_cancers_10_posrisk_8_negrisk_40 | GPs | hypothetical |
| 0.008088 | 0.039314 | 0.083522 | pred_wait_2_detect_2_form4_stage_1_cancers_25_posrisk_8_negrisk_40 | GPs | hypothetical |
| 0.303987 | 0.49117 | 0.680335 | pred_wait_2_detect_2_form4_stage_2_cancers_1_posrisk_2_negrisk_1 | GPs | hypothetical |
| 0.337389 | 0.533724 | 0.725643 | pred_wait_2_detect_2_form4_stage_2_cancers_5_posrisk_2_negrisk_1 | GPs | hypothetical |
| 0.386562 | 0.590536 | 0.781397 | pred_wait_2_detect_2_form4_stage_2_cancers_10_posrisk_2_negrisk_1 | GPs | hypothetical |
| 0.56791 | 0.754595 | 0.901387 | pred_wait_2_detect_2_form4_stage_2_cancers_25_posrisk_2_negrisk_1 | GPs | hypothetical |
| 0.264107 | 0.449719 | 0.640976 | pred_wait_2_detect_2_form4_stage_2_cancers_1_posrisk_2_negrisk_5 | GPs | hypothetical |
| 0.294761 | 0.491222 | 0.689509 | pred_wait_2_detect_2_form4_stage_2_cancers_5_posrisk_2_negrisk_5 | GPs | hypothetical |
| 0.339423 | 0.547987 | 0.749008 | pred_wait_2_detect_2_form4_stage_2_cancers_10_posrisk_2_negrisk_5 | GPs | hypothetical |
| 0.519522 | 0.721161 | 0.884091 | pred_wait_2_detect_2_form4_stage_2_cancers_25_posrisk_2_negrisk_5 | GPs | hypothetical |
| 0.216659 | 0.397211 | 0.589637 | pred_wait_2_detect_2_form4_stage_2_cancers_1_posrisk_2_negrisk_10 | GPs | hypothetical |
| 0.243033 | 0.436297 | 0.63862 | pred_wait_2_detect_2_form4_stage_2_cancers_5_posrisk_2_negrisk_10 | GPs | hypothetical |
| 0.282856 | 0.491391 | 0.701892 | pred_wait_2_detect_2_form4_stage_2_cancers_10_posrisk_2_negrisk_10 | GPs | hypothetical |
| 0.45484 | 0.672423 | 0.856132 | pred_wait_2_detect_2_form4_stage_2_cancers_25_posrisk_2_negrisk_10 | GPs | hypothetical |
| 0.056062 | 0.156536 | 0.284003 | pred_wait_2_detect_2_form4_stage_2_cancers_1_posrisk_2_negrisk_40 | GPs | hypothetical |
| 0.063175 | 0.172773 | 0.312522 | pred_wait_2_detect_2_form4_stage_2_cancers_5_posrisk_2_negrisk_40 | GPs | hypothetical |
| 0.075459 | 0.198764 | 0.355387 | pred_wait_2_detect_2_form4_stage_2_cancers_10_posrisk_2_negrisk_40 | GPs | hypothetical |
| 0.148482 | 0.323884 | 0.528296 | pred_wait_2_detect_2_form4_stage_2_cancers_25_posrisk_2_negrisk_40 | GPs | hypothetical |
| 0.109547 | 0.240304 | 0.394291 | pred_wait_2_detect_2_form4_stage_2_cancers_1_posrisk_4_negrisk_1 | GPs | hypothetical |
| 0.122251 | 0.26442 | 0.429584 | pred_wait_2_detect_2_form4_stage_2_cancers_5_posrisk_4_negrisk_1 | GPs | hypothetical |
| 0.142905 | 0.300534 | 0.480386 | pred_wait_2_detect_2_form4_stage_2_cancers_10_posrisk_4_negrisk_1 | GPs | hypothetical |
| 0.250066 | 0.446788 | 0.651691 | pred_wait_2_detect_2_form4_stage_2_cancers_25_posrisk_4_negrisk_1 | GPs | hypothetical |
| 0.087428 | 0.207584 | 0.353139 | pred_wait_2_detect_2_form4_stage_2_cancers_1_posrisk_4_negrisk_5 | GPs | hypothetical |
| 0.097824 | 0.228828 | 0.385715 | pred_wait_2_detect_2_form4_stage_2_cancers_5_posrisk_4_negrisk_5 | GPs | hypothetical |
| 0.114609 | 0.261381 | 0.434085 | pred_wait_2_detect_2_form4_stage_2_cancers_10_posrisk_4_negrisk_5 | GPs | hypothetical |
| 0.209791 | 0.401397 | 0.609054 | pred_wait_2_detect_2_form4_stage_2_cancers_25_posrisk_4_negrisk_5 | GPs | hypothetical |
| 0.066127 | 0.170744 | 0.304518 | pred_wait_2_detect_2_form4_stage_2_cancers_1_posrisk_4_negrisk_10 | GPs | hypothetical |
| 0.073137 | 0.188371 | 0.332958 | pred_wait_2_detect_2_form4_stage_2_cancers_5_posrisk_4_negrisk_10 | GPs | hypothetical |
| 0.086993 | 0.216151 | 0.376289 | pred_wait_2_detect_2_form4_stage_2_cancers_10_posrisk_4_negrisk_10 | GPs | hypothetical |
| 0.16464 | 0.345036 | 0.550102 | pred_wait_2_detect_2_form4_stage_2_cancers_25_posrisk_4_negrisk_10 | GPs | hypothetical |
| 0.011934 | 0.052231 | 0.115253 | pred_wait_2_detect_2_form4_stage_2_cancers_1_posrisk_4_negrisk_40 | GPs | hypothetical |
| 0.013272 | 0.056813 | 0.124409 | pred_wait_2_detect_2_form4_stage_2_cancers_5_posrisk_4_negrisk_40 | GPs | hypothetical |
| 0.015915 | 0.064978 | 0.140378 | pred_wait_2_detect_2_form4_stage_2_cancers_10_posrisk_4_negrisk_40 | GPs | hypothetical |
| 0.036407 | 0.11642 | 0.231362 | pred_wait_2_detect_2_form4_stage_2_cancers_25_posrisk_4_negrisk_40 | GPs | hypothetical |
| 0.04662 | 0.120473 | 0.217205 | pred_wait_2_detect_2_form4_stage_2_cancers_1_posrisk_6_negrisk_1 | GPs | hypothetical |
| 0.052975 | 0.131826 | 0.233437 | pred_wait_2_detect_2_form4_stage_2_cancers_5_posrisk_6_negrisk_1 | GPs | hypothetical |
| 0.061284 | 0.14935 | 0.259542 | pred_wait_2_detect_2_form4_stage_2_cancers_10_posrisk_6_negrisk_1 | GPs | hypothetical |
| 0.109005 | 0.22944 | 0.369791 | pred_wait_2_detect_2_form4_stage_2_cancers_25_posrisk_6_negrisk_1 | GPs | hypothetical |
| 0.036111 | 0.102505 | 0.191399 | pred_wait_2_detect_2_form4_stage_2_cancers_1_posrisk_6_negrisk_5 | GPs | hypothetical |
| 0.041064 | 0.112244 | 0.20671 | pred_wait_2_detect_2_form4_stage_2_cancers_5_posrisk_6_negrisk_5 | GPs | hypothetical |
| 0.04866 | 0.127537 | 0.22989 | pred_wait_2_detect_2_form4_stage_2_cancers_10_posrisk_6_negrisk_5 | GPs | hypothetical |
| 0.088753 | 0.200367 | 0.333966 | pred_wait_2_detect_2_form4_stage_2_cancers_25_posrisk_6_negrisk_5 | GPs | hypothetical |
| 0.026075 | 0.083252 | 0.161633 | pred_wait_2_detect_2_form4_stage_2_cancers_1_posrisk_6_negrisk_10 | GPs | hypothetical |
| 0.029829 | 0.091163 | 0.176037 | pred_wait_2_detect_2_form4_stage_2_cancers_5_posrisk_6_negrisk_10 | GPs | hypothetical |
| 0.035325 | 0.103858 | 0.195946 | pred_wait_2_detect_2_form4_stage_2_cancers_10_posrisk_6_negrisk_10 | GPs | hypothetical |
| 0.068687 | 0.167477 | 0.28956 | pred_wait_2_detect_2_form4_stage_2_cancers_25_posrisk_6_negrisk_10 | GPs | hypothetical |
| 0.004109 | 0.026114 | 0.064679 | pred_wait_2_detect_2_form4_stage_2_cancers_1_posrisk_6_negrisk_40 | GPs | hypothetical |
| 0.004675 | 0.02827 | 0.068103 | pred_wait_2_detect_2_form4_stage_2_cancers_5_posrisk_6_negrisk_40 | GPs | hypothetical |
| 0.005886 | 0.032088 | 0.07588 | pred_wait_2_detect_2_form4_stage_2_cancers_10_posrisk_6_negrisk_40 | GPs | hypothetical |
| 0.013687 | 0.055808 | 0.118819 | pred_wait_2_detect_2_form4_stage_2_cancers_25_posrisk_6_negrisk_40 | GPs | hypothetical |
| 0.026531 | 0.077031 | 0.14247 | pred_wait_2_detect_2_form4_stage_2_cancers_1_posrisk_8_negrisk_1 | GPs | hypothetical |
| 0.030154 | 0.083752 | 0.151369 | pred_wait_2_detect_2_form4_stage_2_cancers_5_posrisk_8_negrisk_1 | GPs | hypothetical |
| 0.035947 | 0.093946 | 0.167053 | pred_wait_2_detect_2_form4_stage_2_cancers_10_posrisk_8_negrisk_1 | GPs | hypothetical |
| 0.062542 | 0.139674 | 0.230739 | pred_wait_2_detect_2_form4_stage_2_cancers_25_posrisk_8_negrisk_1 | GPs | hypothetical |
| 0.020422 | 0.066498 | 0.126566 | pred_wait_2_detect_2_form4_stage_2_cancers_1_posrisk_8_negrisk_5 | GPs | hypothetical |
| 0.023755 | 0.072357 | 0.134879 | pred_wait_2_detect_2_form4_stage_2_cancers_5_posrisk_8_negrisk_5 | GPs | hypothetical |
| 0.02876 | 0.081356 | 0.148523 | pred_wait_2_detect_2_form4_stage_2_cancers_10_posrisk_8_negrisk_5 | GPs | hypothetical |
| 0.052063 | 0.122804 | 0.20864 | pred_wait_2_detect_2_form4_stage_2_cancers_25_posrisk_8_negrisk_5 | GPs | hypothetical |
| 0.014617 | 0.055196 | 0.108952 | pred_wait_2_detect_2_form4_stage_2_cancers_1_posrisk_8_negrisk_10 | GPs | hypothetical |
| 0.016834 | 0.060081 | 0.117257 | pred_wait_2_detect_2_form4_stage_2_cancers_5_posrisk_8_negrisk_10 | GPs | hypothetical |
| 0.020966 | 0.067707 | 0.129194 | pred_wait_2_detect_2_form4_stage_2_cancers_10_posrisk_8_negrisk_10 | GPs | hypothetical |
| 0.040592 | 0.104061 | 0.182106 | pred_wait_2_detect_2_form4_stage_2_cancers_25_posrisk_8_negrisk_10 | GPs | hypothetical |
| 0.002123 | 0.01975 | 0.050845 | pred_wait_2_detect_2_form4_stage_2_cancers_1_posrisk_8_negrisk_40 | GPs | hypothetical |
| 0.002481 | 0.021388 | 0.053261 | pred_wait_2_detect_2_form4_stage_2_cancers_5_posrisk_8_negrisk_40 | GPs | hypothetical |
| 0.003279 | 0.024122 | 0.058531 | pred_wait_2_detect_2_form4_stage_2_cancers_10_posrisk_8_negrisk_40 | GPs | hypothetical |
| 0.008088 | 0.039314 | 0.083522 | pred_wait_2_detect_2_form4_stage_2_cancers_25_posrisk_8_negrisk_40 | GPs | hypothetical |
| 0.020422 | 0.066498 | 0.126566 | pred_wait_2_detect_1_form1_stage_2_cancers_1_posrisk_8_negrisk_5 | GPs | FIT |
| 0.027839 | 0.077804 | 0.139658 | pred_wait_1_detect_1_form1_stage_2_cancers_10_posrisk_9_negrisk_2 | GPs | CA125 |
| 4.63E-05 | 0.014598 | 0.040777 | pred_wait_2_detect_2_form1_stage_2_cancers_1_posrisk_8_negrisk_240 | GPs | PSA |
